# Selective, Transition Metal‐free 1,2‐Diboration of Alkyl Halides, Tosylates, and Alcohols

**DOI:** 10.1002/chem.202200480

**Published:** 2022-03-19

**Authors:** Mingming Huang, Jiefeng Hu, Shasha Shi, Alexandra Friedrich, Johannes Krebs, Stephen A. Westcott, Udo Radius, Todd B. Marder

**Affiliations:** ^1^ Institut für Anorganische Chemie and Institute for Sustainable Chemistry & Catalysis with Boron Julius-Maximilians-Universität Würzburg Am Hubland 97074 Würzburg Germany; ^2^ School of Chemistry and Molecular Engineering Nanjing Tech University Nanjing 211816 China; ^3^ Department of Chemistry & Biochemistry Mount Allison University Sackville NB E4L 1G8 Canada

**Keywords:** 1,2-diboration, boron; boronate, 1,2-diborylalkanes, metal-free

## Abstract

Defunctionalization of readily available feedstocks to provide alkenes for the synthesis of multifunctional molecules represents an extremely useful process in organic synthesis. Herein, we describe a transition metal‐free, simple and efficient strategy to access alkyl 1,2‐bis(boronate esters) via regio‐ and diastereoselective diboration of secondary and tertiary alkyl halides (Br, Cl, I), tosylates, and alcohols. Control experiments demonstrated that the key to this high reactivity and selectivity is the addition of a combination of potassium iodide and *N*,*N*‐dimethylacetamide (DMA). The practicality and industrial potential of this transformation are demonstrated by its operational simplicity, wide functional group tolerance, and the late‐stage modification of complex molecules. From a drug discovery perspective, this synthetic method offers control of the position of diversification and diastereoselectivity in complex ring scaffolds, which would be especially useful in a lead optimization program.

## Graphical abstract

A transition metal‐free, simple and efficient strategy to access alkyl 1,2‐bis(boronate esters) via regio‐ and diastereoselective diboration of readily available secondary and tertiary alkyl halides (Br, Cl, I), tosylates, and alcohols was developed. The use of KI and DMA is critical to the methodology, which circumvents the regio‐ and diastereoselectivity problems. The transformation showed a broad substrate scope (75 examples), and practicality for the late‐stage modification of natural products.

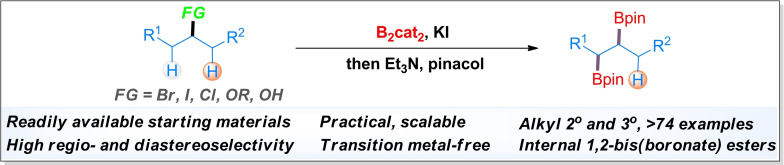

## Introduction

**Figure 1:**
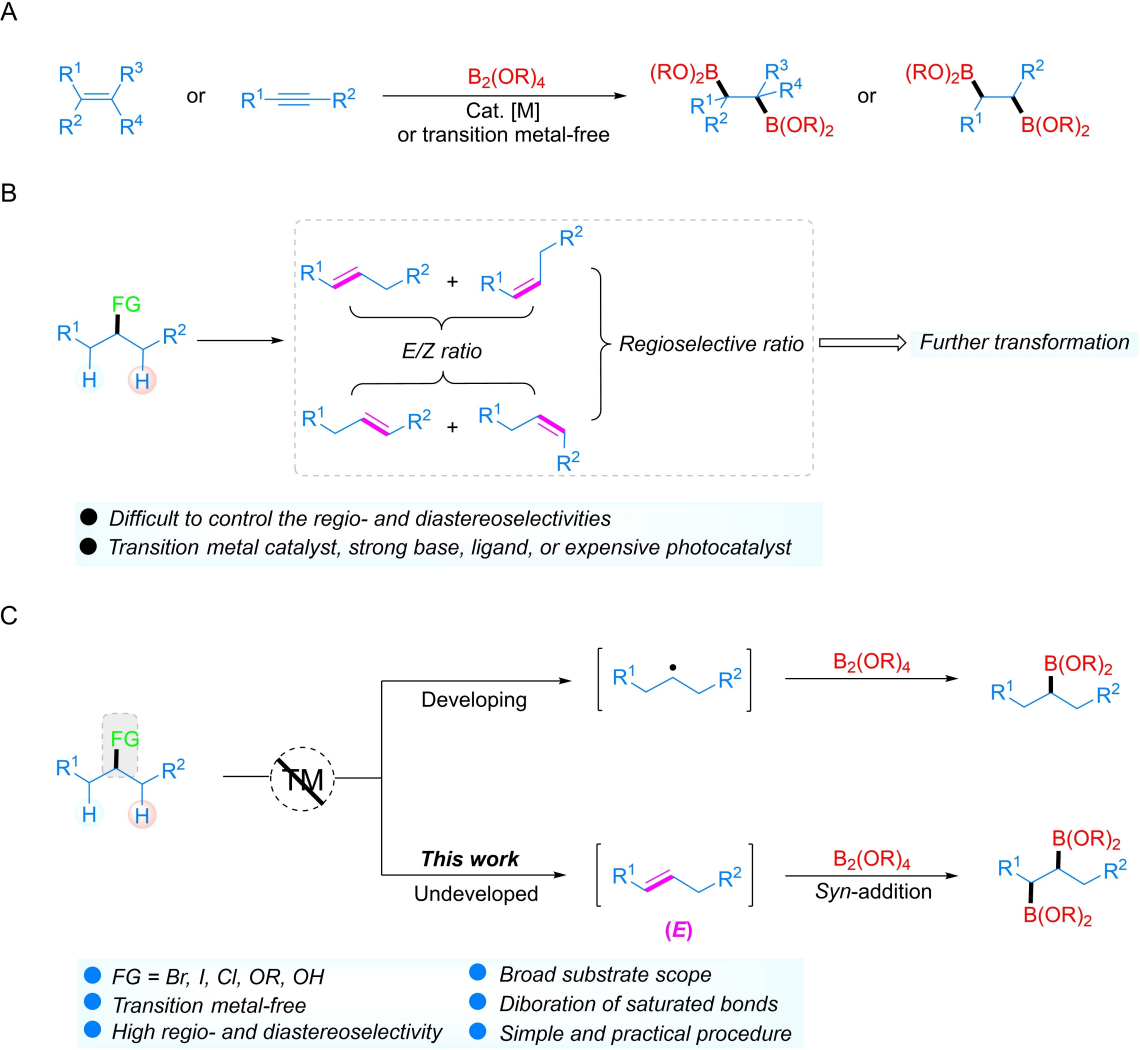
(A) Diboration of unsaturated bonds. (B) Challenges in olefin synthesis via defunctionalization processes. (C) This work: direct site‐selective diboration of alkyl (pseudo)halides and alcohols.

Alkylboronates play an important role in synthetic chemistry, materials science and drug discovery.^[1]^ They are easy to handle due to their good air and moisture stability, and can be readily employed to form carbon‐carbon and carbon‐heteroatom bonds and converted to various functional groups under mild reaction conditions.^[2]^ The early approach to generate alkyl boronates typically focused on transmetalation using organolithium or Grignard reagents,^[3]^ or the classical hydroboration of olefins,^[4]^ which was followed by the development of metal‐catalyzed olefin hydroboration.^[5]^ Notably, the diboration of alkenes has attracted much attention because 1,2‐bis(boronate esters) are emerging as important synthetic intermediates for preparing 1,2‐difunctional compounds.^[6]^ In addition, the boryl moieties in different environments in a 1,2‐bis(boronate ester) can be differentiated and converted selectively, allowing the synthesis of a wide variety of complex molecules.^[7]^ From the emergence of diboron(4) compounds, the addition of diboron tetrachloride to ethylene was reported by Schlesinger.^[8]^ Compared with diboron tetrahalide species, diboron(4) esters are much easier to handle, and are now commercially available in ton quantities.^[9]^ Shortly after the initial report on the diboration of alkynes with bis(pinacolato)diboron (B_2_pin_2_) by Miyaura and Suzuki in 1993,^[10]^ the first examples of the 1,2‐diboration of alkenes using rhodium and gold catalysts were reported by Baker, Westcott, Marder and co‐workers.^[11]^ Subsequently, transition metal‐catalyzed syntheses of 1,2‐diborylalkanes from terminal or internal alkenes and alkynes have been widely reported by Marder,^[12]^ Fernández,^[13]^ Yun,^[14]^ and others,^[15]^ and enantioselective diboration^[12b]^ was subsequently developed by Morken^[16]^ and Hoveyda^[17]^ (Figure 1A). Recently, Fernández and co‐workers employed a Lewis acid‐base adduct,^[18]^ formed from an alkoxide and a diboron(4) reagent, which enabled the first transition metal‐free 1,2‐diboration of nonactivated alkenes.^[19]^ Furthermore, other transition metal‐free protocols have been developed for this 1,2‐diboration process, such as amine‐catalyzed or mediated,[^20a^, ^20b^] hydroxyl‐directed,^[20c]^ carbohydrate‐catalyzed enantioselective 1,2‐diboration,^[20d]^ unidirectional homologation of diborylmethane,^[20e]^ and the reductive diboration of aryl alkenes with Na dispersion.^[20f]^ Later, this strategy was also employed by Song's group to the base‐catalyzed, selective diboration of alkynes in the presence of MeOH.^[21]^

As the most prevalent and readily available alkyl source, alkyl halides, alcohols, and their derivatives have recently been utilized in borylation reactions catalyzed by transition metals.[^22^, ^23^] Despite the rapid development of these methods, diboration of these substrates is extremely rare. In 2019, Fu and co‐workers developed a nickel‐catalyzed vicinal diboration of alkyl bromides for the synthesis of 1,2‐diborylalkanes; however, the products obtained were limited to terminal 1,2‐bis(boronate esters).^[23m]^ With increasing attention to sustainable chemistry, transition metal‐free protocols[^19e^, ^24^, ^25^, ^26^, ^27^] have emerged as practical tools for the direct conversion of alkyl (pseudo)halides to alkyl boronates. The Studer,^[28]^ Melchiorre,^[29]^ and Jiao^[30]^ groups independently reported the metal‐free radical borylation of primary and secondary or benzylic alkyl halides using B_2_cat_2_ under blue LED irradiation, providing a broad range of alkylboronate esters. This protocol was later extended by Mo, et al.^[31]^ to the thermal borylation of primary alkyl iodides using B_2_pin_2_. Interestingly, deoxygenative monoborylations of secondary and tertiary alcohol derivatives, including xanthates, thionocarbamates, and methyl oxalate esters, using B_2_cat_2_ in DMF were disclosed by Studer^[32]^ and Aggarwal.^[33]^ Surprisingly, despite much effort on the metal‐free borylation of alkyl precursors (including Br, I, and alcohol derivatives) (see above): 1) the transition metal‐free direct borylation of unactivated alkyl chlorides and alcohols remains an unsolved problem, as most such reactions require transition metals;^[23]^ and 2) almost all metal‐free borylations of alkyl precursors focus on monoborylation. To the best of our knowledge, the metal‐free diboration of alkyl halides and alcohols has not been achieved to date.

Defunctionalization of readily available feedstocks has emerged as one of the most valuable strategies for the generation of alkenes.^[34]^ However, most of these transformations require a transition metal catalyst, a ligand, a strong base, or an expensive photocatalyst. Additionally, it is difficult to the control of regio‐ and stereoselectivities of the reactions; thus, four isomers of the alkenes were generally obtained restricting the selectivity of further transformations (Figure 1B). Therefore, we set as a goal the development of practical methods to solve these challenges. Given the easy accessibility and abundance of alkyl halides (including iodides, bromides and chlorides) and alcohols, we report, herein, the transition metal‐free diboration of alkyl halides, tosylates, and alcohols as a highly selective synthetic approach to useful 1,2‐bis(boronate esters) (Figure 1C).

## Results and Discussion

We set out to explore the possibility of metal‐free diboration using the secondary alkyl tosylate (**1 a)** as a model substrate (Table 1). As commonly used sources of diboron reagent in borylation reactions, bis(pinacolato)diboron (B_2_pin_2_), bis(neopentyl glycolato)diboron (B_2_neop_2_), and tetrahydroxydiboron (B_2_(OH)_4_) failed to afford any product in DMA at 80 °C for 12 h, whereas when bis(catecholato)diboron (B_2_cat_2_) was applied to this reaction, only trace amounts of 1,2‐bisboronate pinacol ester were detected by GC‐MS after transesterification of the crude catechol boronate ester with pinacol in Et_3_N (entries 1–4). When the reaction time was extended to 72 h, 1,2‐bisboronate pinacol ester regioisomers (**1 b**/**1 b’**=58/42) were obtained in 18 % yield (entry 5). By screening solvents such as DMF, 1,4‐dioxane, MeCN, and toluene (entries 6–9), we found that only an amide‐based solvent was effective for this transformation. To improve the reactivity, we next tested a variety of bases or other additives. In view of the fact that strong bases can promote radical borylation,^31^ we tested some strong bases, including LiO^*t*^Bu and NaOMe, which were not efficient for our diboration reaction, and the monoboration product was also not detected in our system (entries 10 and 11). Neutral Lewis base additives, 4‐PhPy and PPh_3_, proved ineffective (entries 12 and 13). As the addition of KOAc showed a slightly increase in reactivity (entry 14), other alkali salts were screened (entries 15–17). Thus, upon addition of a stoichiometric amount of NaI to the reaction at 80 °C, a 62 % yield of 1,2‐diborylalkanes was obtained with the ratio of the internal and terminal borylation products **1 b** and **1 b’**, respectively, being 77 : 23 (entry 16). Interestingly, KI showed a dramatically increased yield and excellent regioselectivity of **1 b**/**1 b’** (entry 18). When a catalytic amount of KI was employed, we observed no change in reactivity, but a decrease in its selectivity (see Table S1 in the Supporting Information). Further study showed that a slight temperature increase (90 °C) provided a higher isolated yield (95 %), rr=95 : 5, dr>10 : 1 (entry 19). This result suggested that KI could serve as an effective additive to promote the yield significantly with competitive regioselectivity.

**Table 1:** Optimization of the reaction conditions.^[a]^

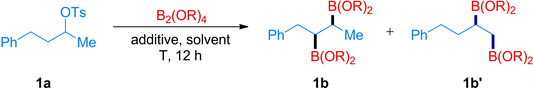						
entry	B_2_(OR)_4_	solvent	additive	temperature [°C]	Yield [%]^[b]^	**1 b**/**1 b’** ^[c]^
1	B_2_pin_2_	DMA	–	80	0	–
2	B_2_neop_2_	DMA	–	80	0	–
3	B_2_(OH)_4_	DMA	–	80	0	–
4	B_2_cat_2_	DMA	–	80	trace	–
5^[d]^	B_2_cat_2_	DMA	–	80	18	58/42
6^[d]^	B_2_cat_2_	DMF	–	80	10	55/45
7	B_2_cat_2_	1,4‐dioxane	–	80	0	–
8	B_2_cat_2_	MeCN	–	80	0	–
9	B_2_cat_2_	toluene	–	80	0	–
10	B_2_cat_2_	DMA	LiO^*t*^Bu	80	27	54/46
11	B_2_cat_2_	DMA	NaOMe	80	trace	–
12	B_2_cat_2_	DMA	4‐PhPy	80	0	–
13	B_2_cat_2_	DMA	PPh_3_	80	0	–
14	B_2_cat_2_	DMA	KOAc	80	24	57/43
15	B_2_cat_2_	DMA	KCl	80	27	61/39
16^[d]^	B_2_cat_2_	DMA	NaI	80	62	77/23
17	B_2_cat_2_	DMA	TBAI	80	39	71/28
18	B_2_cat_2_	DMA	KI	80	84	94/6
19	B_2_cat_2_	DMA	KI	90	95	95/5

[a] Reaction conditions: alkyl tosylates **1 a** (0.3 mmol, 1.0 equiv.), B_2_(OR)_4_ (2.5 equiv.), additive (1.0 equiv.), solvent (1.0 mL), 12 h, under argon; then pinacol (0.9 mmol), Et_3_N (1.0 mL), rt, 1 h. [b] Isolated yield of **1 b** and **1 b’** after chromatographic workup. [c] The ratio of **1 b** and **1 b’** was determined from the crude reaction mixture by GC‐MS analysis vs. a calibrated internal standard and are averages of two runs. [d] 80 °C, 72 h. 4‐PhPy = 4‐Phenylpyridine. DMA = *N,N*‐Dimethylacetamide. DMF = *N,N*‐Dimethylformamide.

**Scheme 1:**
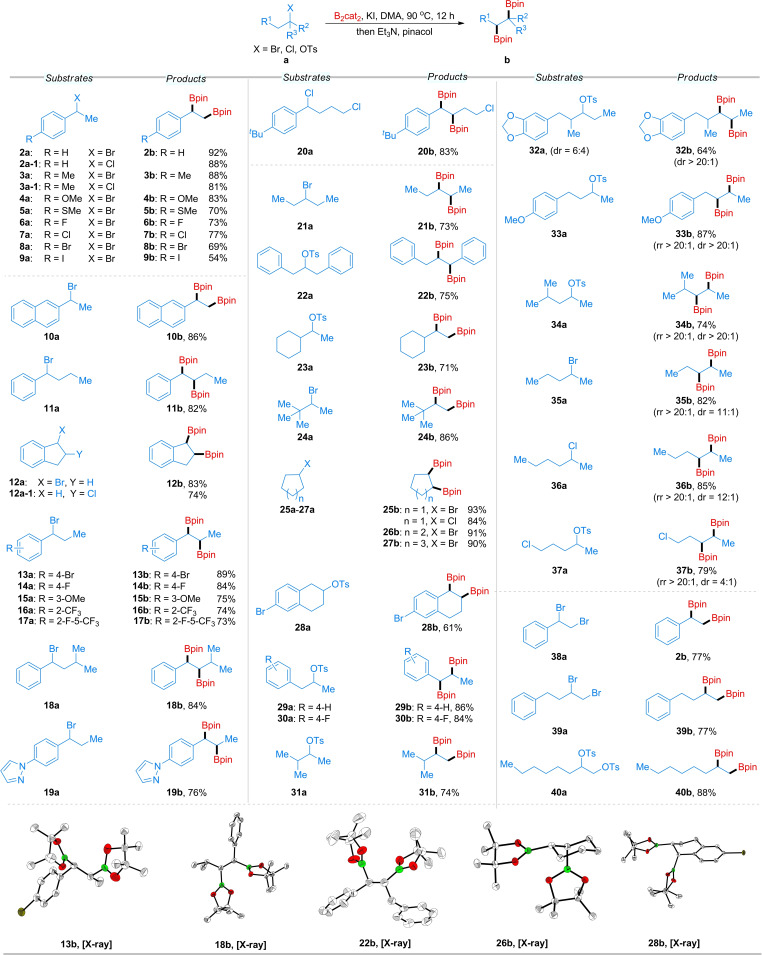
Diboration of secondary alkyl halides and tosylates.^[a]^ [a] All reaction were conducted on 0.3 mmol scale; isolated yield after chromatography; rr's were determined by GC‐MS analysis of the crude reaction mixture vs. a calibrated internal standard and are averages of two runs, and dr's were determined by ^1^H NMR spectroscopic analysis of the crude reaction mixture vs. a calibrated internal standard and are averages of two runs.

With the optimal reaction conditions in hand, we proceeded to investigate the substrate scope of this transformation using various alkyl halides and tosylates. As shown in Scheme 1, secondary alkyl halides and tosylates could easily be diborylated with good to excellent yields. Thus, 1‐(bromoethyl)arenes (**2 a**–**5 a**), bearing a *para*‐Me, OMe, or SMe substituent, were converted into the corresponding alkylboronate esters in excellent isolated yields (**2 b**–**5 b**). Halide substituents (F, Cl, Br and I) on the benzene rings of the substrates were compatible with the diboration reaction, exhibiting selective cleavage of the alkyl C−Br bond over the aryl C−X bond (**6 b**–**9 b**). We also investigated the compatibility of alkyl chlorides, as examples of the borylation of these substrates remain rare and usually require transition metals. Strikingly, the diboration of alkyl chlorides (**2 a‐1** and **3 a‐1**) preceded well under our conditions, giving the corresponding diboration products. Diboration of 2‐(1‐bromoethyl)naphthalene **10 a** was also successful, and **10 b** was obtained in 86 % yield. A series of other linear and cyclic benzyl bromides also worked well to generate the internal 1,2‐bisboronate pinacol esters bearing two stereocenters (**11 a**–**19 a**). Regarding the effect of steric hindrance, substituents in the alkyl branch did not affect the reaction efficiency (**11 a**, **18 a**). *Para‐*, *meta‐*, and even *ortho*‐substituted (1‐bromopropyl)arenes **13 a**–**17 a** performed well to deliver the corresponding diborylated products. Substrate **19 a** containing an *N*‐heterocycle was also compatible under standard conditions. The relative stereochemistries of the racemic diborylated products were assessed by analogy with single‐crystal X‐ray diffraction studies performed on **13 b** and **18 b**. Similar to the previously reported transition‐metal‐free nucleophilic 1,2‐diborations of non‐activated olefins, which occurs in a *syn* fashion,[^19^, ^20^] our racemic diborylated products were formed in a *syn*‐configuration. Notably, substrate **20 a**, containing both a primary and a secondary chloro group, also exhibited excellent chemoselectivity giving an 83 % yield of **20 b**. The synthesis of products bearing two secondary alkyl boronate esters were also performed in 73 and 75 % yields, respectively, from symmetrical substrates **21 a** and **22 a**. It is worth noting the regioselective transformations of 1‐cyclohexylethanol derivative **23 a** and hindered halide **24 a** to the corresponding sole products in 71 and 86 % yields, respectively. Similarly to **12 b**, cyclopentyl, cyclohexyl, and cycloheptyl halides (bromides and chlorides) reacted efficiently with B_2_cat_2_ to produce vicinal diboronates with a *syn*‐configuration (**25 b**–**27 b**). The stereochemistry of **26 b** was determined by single‐crystal X‐ray diffraction (Scheme 1). For the unsymmetrical six‐membered ring substrate **28 a**, *cis*‐diboronate **28 b** was obtained with complete regioselectivity, with the second boryl moiety at the benzylic site. The *syn* stereochemistry and site‐selectivity of **28 b** was determined by single crystal X‐ray diffraction.

Another series of unsymmetrical substrates **29 a**–**36 a** was also studied to examine site‐selectivity under our reaction conditions. Among them, for substrates possessing two alternative β sites (internal or terminal sites) (**29 a**, **30 a** and **33 a**–**36 a**), the second Bpin moiety was preferentially incorporated at the internal position to generate the internal 1,2‐bisboronate pinacol ester with excellent regio‐ and diastereoselectivities. Interestingly, when the internal position was substituted with a methyl group, such as in **31 a** and **32 a**, or was a cyclohexyl ring (i. e., a tertiary carbon) as in **23 a**, the second boryl group was installed at the terminal position. In these cases, this suggests that the site of deprotonation in the alkene‐forming HX elimination step is influenced by steric effects. In addition to unactivated secondary alkyl chloride **36 a**, which can efficiently produce internal 1,2‐bis(boronate esters), substrate **37 a** with both primary alkyl chloride and secondary alkyl tosylate sites exhibited similar reactivity, high regioselectivity, and good stereoselectivity. Interestingly, using 1,2‐dibromo (**38 a**, **39 a**) or 1,2‐ditosyl (**40 a**) substrates, we obtained chemoselective diborylated products. This process also provides an efficient method to control the reaction sites for 1,2‐difunctionalization. The corresponding alkenes were observed in the absence of B_2_cat_2_ when 1,2‐dibromo (**38 a**, **39 a**) or 1,2‐ditosyl (**40 a**) substrates were employed under the standard conditions.^[35]^

**Scheme 2:**
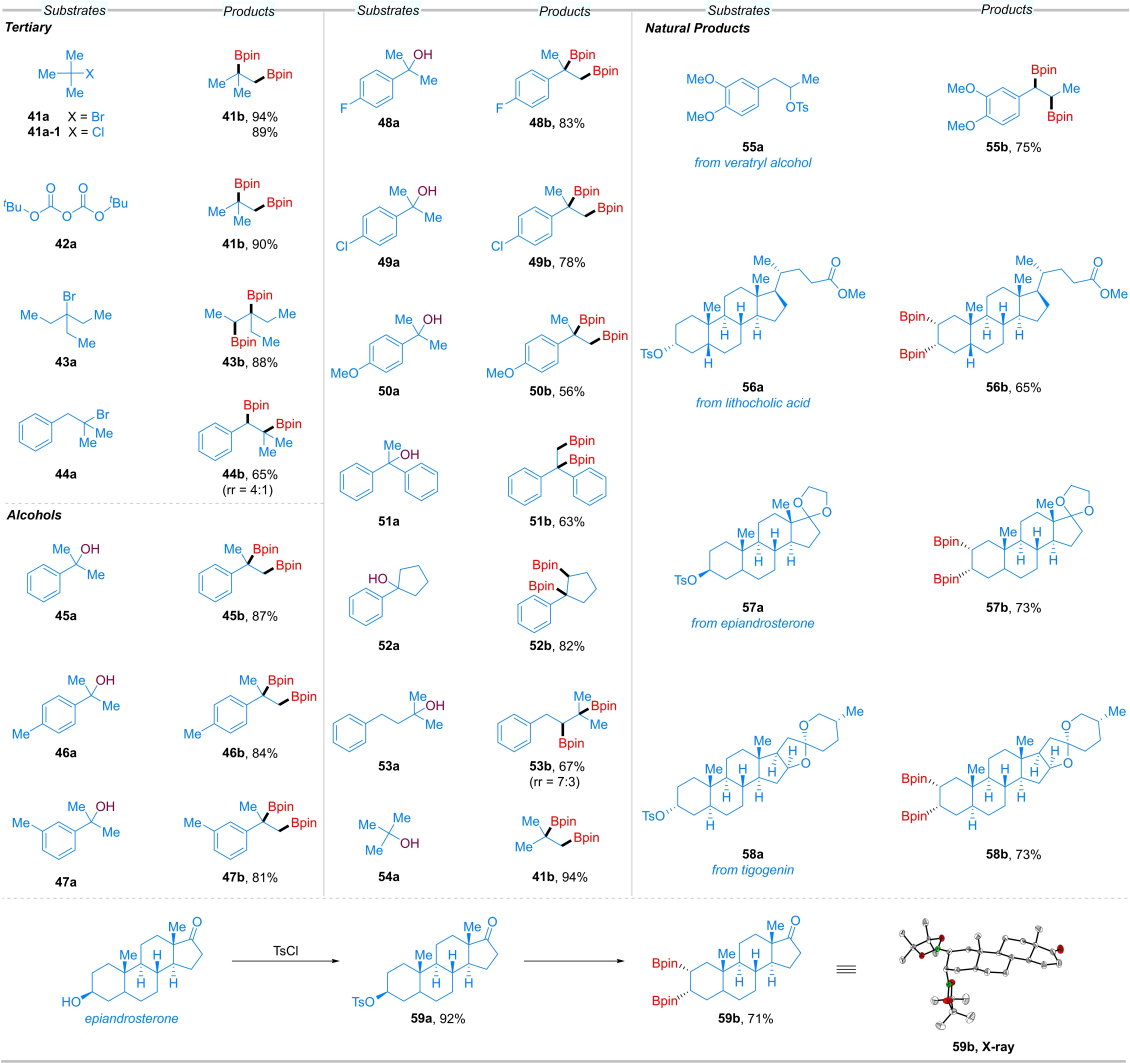
Diboration of tertiary (pseudo)halides, alcohols and natural product derivatives.

Furthermore, various *tert*‐alkyl substrates and complex natural products were examined (Scheme 2). The simple and commercially available *tert*‐butyl halides (Br and Cl) (**41 a** and **41 a‐1**) and Boc_2_O (**42 a**) both afforded diborylated compound **41 b** in 89–94 % yields. A slightly more sterically hindered *tert*‐alkyl bromide **43 a** also performed well. Substrate **44 a** showed good regioselectivity (rr=4 : 1) to afford the internal diboronated products.

In light of the above results, we wondered whether our diboration reaction could be applied directly to alkyl alcohols. In fact, a variety of benzyl *tert*‐alkyl alcohols possessing Me, F, Cl, and OMe groups were well tolerated, giving good yields of **45 b**–**50 b** in all cases. The more sterically hindered substrate **51 a** did not interfere with productive C−B bond formation (**51 b**). The internal cyclic product **52 b** was also obtained from the corresponding tertiary alcohol, and **53 b**, along with another terminal regioisomer, was obtained from alcohol **53 a** under our conditions. Intriguingly, *tert*‐butanol **54 a** smoothly afforded the diborylated product in 94 % yield.

The site‐selective diboration of complex molecules is highly relevant to late‐stage functionalization in total synthesis and drug discovery. Thus, veratryl alcohol derivative **55 a** was subjected to our reaction conditions, delivering **55 b** in 75 % yield. Functional groups such as esters, acetals and ethers, as found in lithocholic acid (**56 a**), epiandrosterone (**57 a**), and tigogenin (**58 a**), respectively, were tolerated, and excellent regio‐ and stereocontrol was achieved. The second boronate ester moieties are incorporated at the less sterically hindered sites to generate **56 b**–**59 b** as single *syn* regioisomers. The relative stereochemistries of **56 b**–**59 b** were assessed by analogy with single crystal X‐ray diffraction studies of **59 b**, which showed that **59 b** was the only isolated stereoisomer present in the solid‐state. From the viewpoint of drug development, this synthetic technique provides control over the selectivity for diversification and diastereoselectivity in the reactions of natural products, which should be crucial for lead optimization processes.

**Scheme 3:**
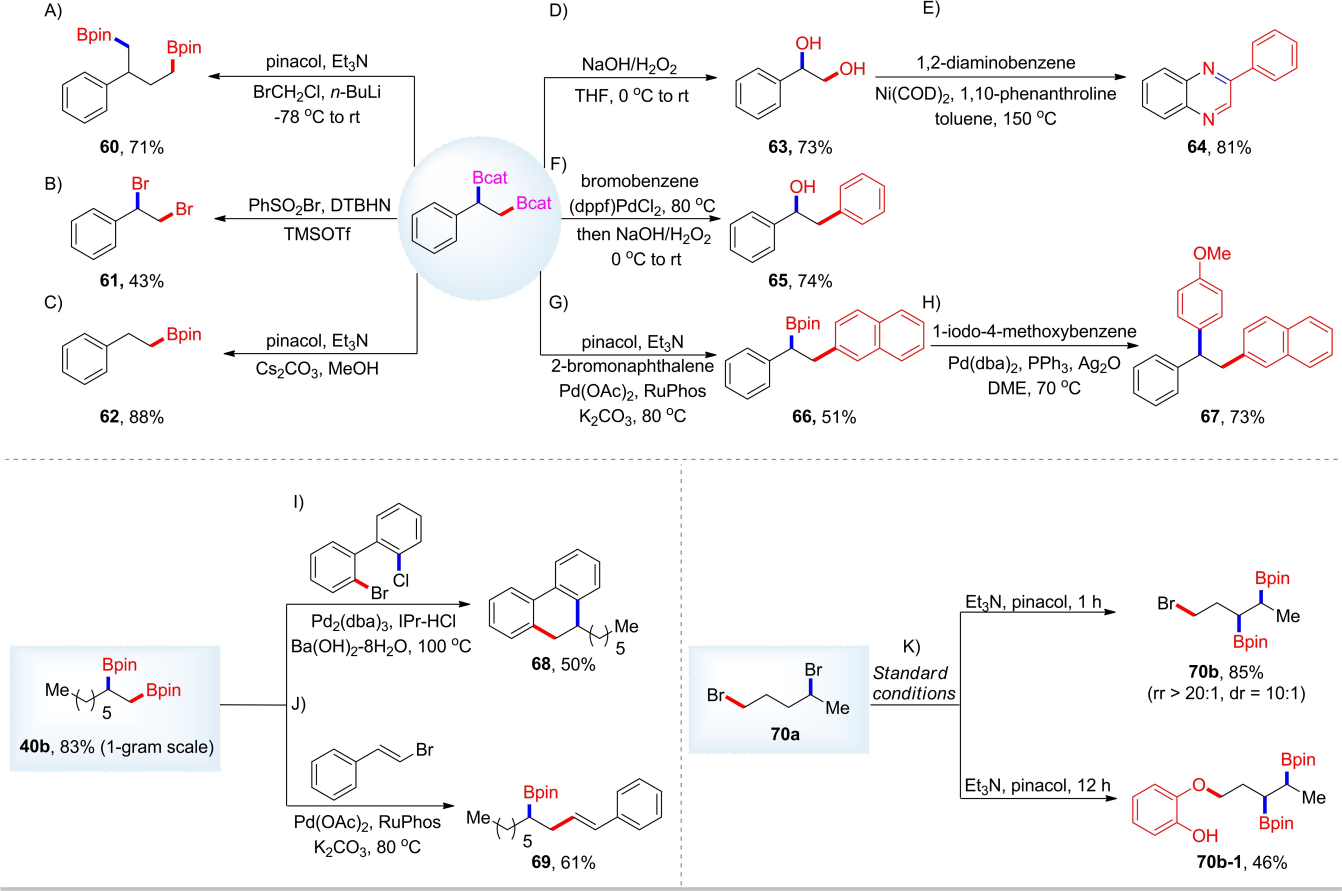
Applications of the 1,2‐diborylalkane products. DTBHN, *trans*‐Di‐*t*‐butylhyponitrite. DME, Dimethoxyethane.

To demonstrate further the synthetic value of this strategy, we carried out a series of reactions of the 1,2‐diborylalkane products (Scheme 3). Subjection to standard Matteson homologation and deboronative bromination^[36]^ gave the corresponding difunctionalization products **60** and **61**, respectively, in 71 and 43 % yields. We were also particularly interested in whether selective functionalization of either the primary or the secondary boronate ester could be achieved. Selective protodeboronation occurred at the benzylic position, which eventually led to homobenzylic boronate **62**.^[7g]^ Quinoxaline **64** was prepared readily from 1,2‐diaminobenzene and 1,2‐diol **63**,^[37]^ which was formed under standard oxidation conditions. Alternatively, the 1,2‐diboronate ester can be reacted, in situ, with bromobenzene in a one‐pot reaction wherein the less hindered C−B bond participates in the Suzuki‐Miyaura cross‐coupling.^[7a]^ Then, the remaining C−B bond was oxidized to produce **65** during the reaction workup. For **2 b**, the primary linear C−B bond could be coupled with an aryl bromide in the presence of Pd(OAc)_2_ and RuPhos to give **66**. The remaining branched, secondary C−B bond was available for further cross‐coupling under Ag_2_O‐promoted conditions to deliver compound **67**.^[7f]^ According to recent work from the Morandi group,^[6e]^ we conducted the stereospecific cascade Suzuki‐Miyaura annulation of alkyl 1,2‐bisboronate ester **40 b** with 2‐bromo‐2’‐chloro biaryl giving rise to 9,10‐dihydrophenanthrene **68**. The secondary alkylboronate ester **69** could also be prepared from **40 b** by reaction with (*E*)‐(2‐bromovinyl)benzene. Regarding chemoselectivity, an intramolecular competition experiment with secondary and primary alkyl bromide sites showed that the former functionality was more reactive. Thus, diboration of **70 a** smoothly provided **70 b** in 85 % yield with excellent regioselectivity, and the primary alkyl bromide moiety was retained. Interestingly, after adding pinacol and trimethylamine to the reaction with stirring for 12 h, we isolated the catechol‐containing product (**70 b‐1**) in which the catechol moiety had displaced the bromide at the primary alkyl site.

**Scheme 4:**
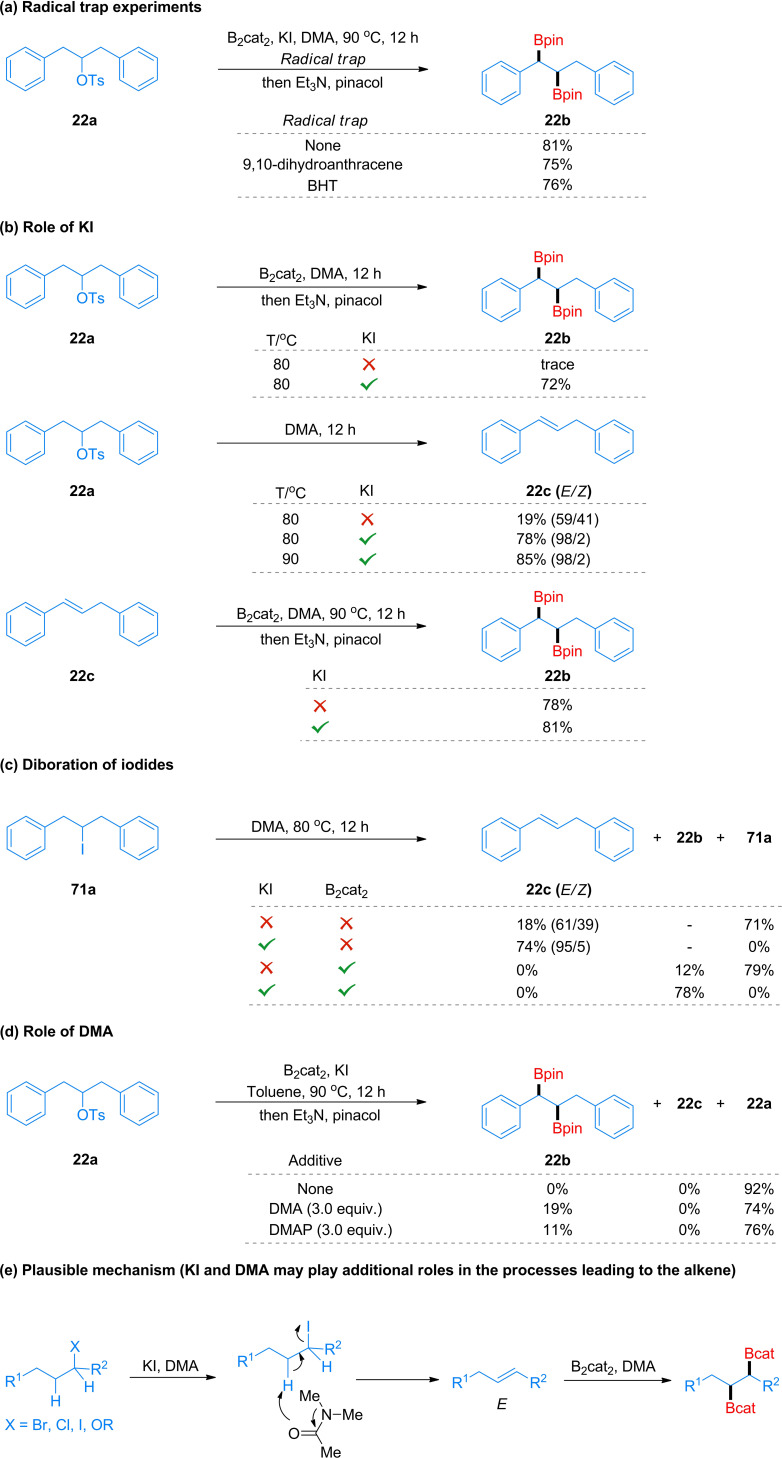
Mechanistic studies.

We next conducted several experiments to explore the mechanism of this diboration process. Firstly, when 9,10‐dihydroanthracene or butylated hydroxytoluene (BHT) were added as radical traps, the yield of diboryl product **22 b** remained largely unaffected (Scheme 4a). This result implies that radical process may not be involved. Secondly, without the addition of KI, only a trace amount of diborylated product **22 b** was detected, and **22 a** was almost completely recovered (Scheme 4b). Clearly, KI plays a crucial role for the generation of the diborylated products. When tosylate **22 a** was reacted in DMA without addition of B_2_cat_2_, olefin **22 c** was obtained in 19 % yield with low stereoselectivity. Upon addition of KI, olefin **22 c** was isolated in 78 % yield with an excellent *E/Z* ratio at 80 °C. The results also implied that alkyl alkenes might be the active intermediates of this transformation. Immediately afterwards, **22 c** could be converted into **22 b** in similarly high yields regardless of the presence or absence of KI under the standard conditions. In order to confirm whether the process undergoes replacement of the OTs in **22 a** by iodide, we synthesized alkyl iodide (**71 a**), which was subjected to our conditions (Scheme 4c). In the absence of B_2_cat_2_, KI promoted the formation of *E*‐alkene (**22 c**). When KI and B_2_cat_2_ were added to the reaction at the same time, the yield of the target product increased significantly. When KI was replaced with KOTf for the conversion of **71 a**, the corresponding alkene **22 c** was also obtained in good yield in the absence of B_2_cat_2_. Thus, K+ and I‐ both assist in this reaction. These results indicated that the presence of KI enhanced both the reactivity and regioselectivity and diastereoselectivity of the reaction. However, this does not rule out the possibility of initial iodide exchange taking place. Finally, we explored the role of DMA in this system (Scheme 4d). Only an amide solvent afforded the desired product, which was in accordance with literature reports, suggesting the weak complexation of B_2_cat_2_ with DMA.[^25^, ^26^] However, 4‐dimethylaminopydidine (DMAP) was somewhat effective as a base additive, indicating that a nitrogen base can promote the diboration process to some extent.[^20a^, ^38^] Based on the above experiments, we propose a plausible mechanism (Scheme 4e). Alkyl (pseudo)halides are initially dehydrohalogenated to form alkenes with high selectivity using a combination of KI and DMA. Subsequently, the alkenes undergo *syn*‐selective diboration[^19a^, ^20a^] with DMA‐activated B_2_cat_2_, providing the target product. For the alkyl tertiary alcohol **53 a**, the corresponding alkene was also formed in good yield in the presence of KI. For tertiary substrates, S_N_2 exchange of X^−^ by I^−^ is not possible, and it is possible that the K^+^ cation also plays a role, with the DMA acting as the base in the deprotonation.

## Conclusions

We have developed a direct and selective diboration of alkyl halides, tosylates and alcohols, thus efficiently synthesizing 1,2‐bis(boronate esters). The use of KI and DMA is critical to the methodology, which circumvents the regio‐ and diastereoselectivity problems. The method shows a broad substrate scope with high yields and selectivities, and practicality for the late‐stage modification of natural molecules. Experimental studies of the reaction mechanism of the selective diborylation process were also carried out. Given how widespread halogen and hydroxyl groups are, we anticipate that this approach will simplify the preparation of diborylalkane targets for research in chemistry, materials, bioactive compounds, and other applications.

## Crystal structures

Deposition Numbers 2119847 (for **13 b**), 2119849 (for **18 b**), 2119873 (for **22 b**), 2119851 (for **26 b**), 2119856 (for **28 b**), 2119852 (for **59 b**) contain the supplementary crystallographic data for this paper. These data are provided free of charge by the joint Cambridge Crystallographic Data Centre and Fachinformationszentrum Karlsruhe Access Structures service.

## Conflict of interest

The authors declare no conflict of interest.

1

## Supporting information

As a service to our authors and readers, this journal provides supporting information supplied by the authors. Such materials are peer reviewed and may be re‐organized for online delivery, but are not copy‐edited or typeset. Technical support issues arising from supporting information (other than missing files) should be addressed to the authors.

Supporting Information

## Data Availability

The data that support the findings of this study are available in the supplementary material of this article.
