# Supplementary material for: Selective, Transition Metal‐free 1,2‐Diboration of Alkyl Halides, Tosylates, and Alcohols
Source: Chemistry. 2022 Mar 19;28(24):e202200480. doi: 10.1002/chem.202200480 (PMC9314653; doi:10.1002/chem.202200480)
Supplement: Supplementary file 1 — Supporting Information [file CHEM-28-0-s001.pdf]

Chemistry–A European Journal

Supporting Information

Selective, Transition Metal-free 1,2-Diboration of Alkyl Halides, Tosylates, and Alcohols

Mingming Huang, Jiefeng Hu,* Shasha Shi, Alexandra Friedrich, Johannes Krebs, Stephen A. Westcott, Udo Radius,* and Todd B. Marder*

Supporting Information

Table of Contents

1 Experimental Section 1

1.1 General Considerations 1

1.2 Optimization of the Reaction Conditions 3

1.3 Preparation of Substrates 5

1.4 Details of the Diboration of Alkyl Halides, Tosylates, and Alcohols 27

1.5 Application of 1,2-Diborylalkane 55

2 Preliminary Mechanistic Investigations 68

3 Single-Crystal X-ray Diffraction Analysis 73

4 References 80

5 NMR Spectra 83

1 Experimental Section

1.1 General Considerations

All reactions and subsequent manipulations were performed under an argon atmosphere using standard Schlenk techniques or in a glovebox (Innovative Technology Inc. and Braun Uni Lab). All reactions were carried out in oven-dried glassware. Reagent grade solvents (Fisher Scientific and J.T. Baker) were nitrogen saturated and were dried and deoxygenated using an Innovative Technology Inc. Pure-Solv 400 Solvent Purification System, and further deoxygenated using the freeze-pump-thaw method. C_6D_6 and $CDCl_3$ were purchased from Sigma-Aldrich. The diboron reagents B_2pin_2, B_2neop_2, and $B_2(OH)_4$, were a generous gift from AllyChem Co. Ltd. All other reagents were purchased from Sigma-Aldrich or ABCR.

NMR spectra were recorded at 298 K using Bruker Avance 300 (^{1}H, 300 MHz; ^{13}C, 75 MHz, ^{11}B, 96 MHz), Bruker DPX-400 (^{1}H, 400 MHz; ^{13}C, 100 MHz, ^{11}B, 128 MHz; ^{19}F, 376 MHz), or Bruker Avance 500 (^{1}H, 500 MHz; ^{13}C, 125 MHz, ^{11}B, 160 MHz; ^{19}F, 470 MHz) spectrometers. ^{1}H NMR chemical shifts are reported relative to TMS and were referenced via residual proton resonances of the corresponding deuterated solvent ($CDCl_3$: 7.26 ppm; C_6D_6: 7.16 ppm) whereas ^{13}C{^{1}H} NMR spectra are reported relative to TMS using the natural-abundance carbon resonances ($CDCl_3$: 77.16 ppm, C_6D_6: 128.0 ppm). ^{11}B and ^{19}F NMR chemical shifts are reported relative to external BF_3•OEt_3 or $CFCl_3$, respectively. Coupling constants are given in Hertz. Elemental analyses were performed in the microanalytical laboratory of the Institute of Inorganic Chemistry, Universität Würzburg, using an Elementar vario micro cube instrument. Automated flash chromatography was performed using a Biotage® Isolera Four system, on silica gel (Biotage SNAP cartridge KP-Sil 10 g and KP-Sil 25 g). Commercially available, precoated TLC plates (Polygram® Sil G/UV254) were purchased from Machery-Nagel. The removal of solvent was performed on a rotary evaporator *in vacuo* at a maximum temperature of 30 °C. GC-MS analyses were performed using a Thermo Fisher Scientific Trace 1310 gas chromatograph (column: TG-SQC 5% phenyl methyl siloxane, 15 m, Ø 0.25 mm, film 0.25 μm; injector: 250 °C; oven: 40 °C (2 min), 40 °C to 280 °C; carrier gas: He (1.2 mL min^{-1}) or an Agilent 7890A gas chromatograph (column: HP-5MS 5% phenyl methyl siloxane, 30 m, Ø 0.25 mm, film 0.25 μm; injector: 250 °C; oven: 40 °C (2 min), 40 °C to 280 °C (20 °C min^{-1}); carrier gas: He (1.2 mL min^{-1}) equipped with an Agilent 5975C inert MSD with triple-axis detector operating in EI mode and an Agilent 7693A series auto sampler/injector. High-resolution

mass spectra were obtained using a Thermo Scientific Exactive Plus spectrometer equipped with an Orbitrap Mass Analyzer. Measurements were accomplished using an ASAP/APCI source with a corona needle, and a carrier-gas (N_2) temperature of 250 °C.

1.2 Optimization of the Reaction Conditions

General procedure of optimization for Table S1. In an argon-filled glovebox, the alkyl tosylate **1a** (0.3 mmol, 1.0 equiv.), dissolved in solvent (1 mL), was added to a 10 mL thick-walled reaction tube equipped with a magnetic stirring bar. Additive and the boron source were added. The reaction mixture was stirred at indicated temperature for 12 h, then a solution of pinacol (106 mg, 0.9 mmol, 3.0 equiv.) in Et_3N (1 mL) was added to the reaction mixture, which was stirred at room temperature for 1 h. Then diluted with Et_2O (2 mL) and filtered through a pad of Celite (Ø 3 mm x 8 mm). The solvent was evaporated under reduced pressure and *n*-dodecane was added as an internal standard and the crude reaction mixture was analyzed by GC-MS.

Table S1: Optimization of the reaction conditions.[a]

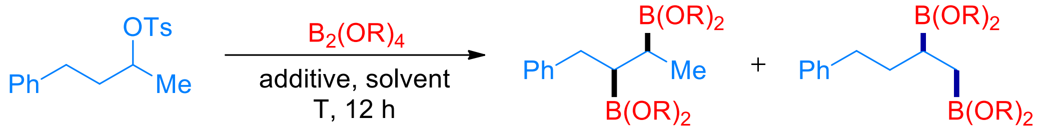

1a **1b** **1b'**

entry	$B_2(OR)_4$	solvent	additive	temperature (°C)	yield (%)[b]	**1b/1b'**[c]
1	B_2pin_2	DMA	-	80	0	-
2	B_2neop_2	DMA	-	80	0	-
3	$B_2(OH)_4$	DMA	-	80	0	-
4	B_2cat_2	DMA	-	80	trace	-
5[d]	B_2cat_2	DMA	-	80	18	58/42
6[d]	B_2cat_2	DMF	-	80	10	55/45
7	B_2cat_2	1,4-dioxane	-	80	0	-
8	B_2cat_2	MeCN	-	80	0	-
9	B_2cat_2	toluene	-	80	0	-
10	B_2cat_2	DMA	LiO^{t}Bu	80	27	54/46
11	B_2cat_2	DMA	NaOMe	80	trace	-
12	B_2cat_2	DMA	4-PhPy	80	0	-
13	B_2cat_2	DMA	PPh_3	80	0	-
14	B_2cat_2	DMA	KOAc	80	24	57/43
15	B_2cat_2	DMA	KCl	80	27	61/39
16	B_2cat_2	DMA	NaI	80	62	77/23
17	B_2cat_2	DMA	TBAI	80	39	71/28

18	B_2cat_2	DMA	KF	80	28	66/34
19	B_2cat_2	DMA	I_2	80	80	85/15
20	B_2cat_2	DMA	KI	80	84	94/6
21	B_2cat_2	DMA	KI	90	95	95/5
22[e]	B_2cat_2	DMA	KI	90	89	68/32

[a] Reaction conditions, unless otherwise stated: alkyl tosylate **1a** (0.3 mmol, 1.0 equiv.), $B_2(OR)_4$ (2.5 equiv.), additive (1.0 equiv.) in solvent (1.0 mL) was stirred for 12 h at indicated temperature under Ar; then, pinacol (0.9 mmol) and Et_3N (1.0 mL) were added and the reaction was stirred for 1 h at rt. [b] Isolated yield after chromatographic workup. [c] The ratio of **1b** and **1b'** was determined of the crude reaction mixture by GC-MS analysis *vs* a calibrated internal standard and are averages of two runs. [d] The reaction was stirred at 80 °C for 72 h. 4-PhPy, 4-Phenylpyridine. DMA, *N,N*-Dimethylacetamide. DMF, *N,N*-Dimethylformamide. [e] 10 mol% KI was employed.

1.3 Preparation of Substrates

General procedure 1: Preparation of secondary alkyl tosylates

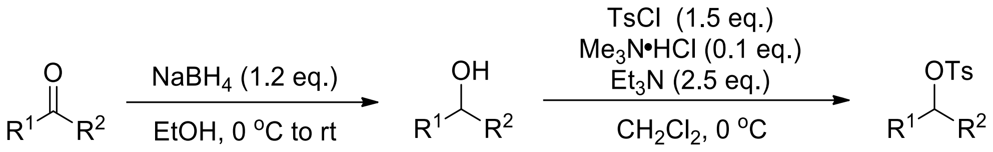

To a solution of ketone (3.0 mmol, 1.0 equiv.) in EtOH (9.0 mL) was added $NaBH_4$ (136.8 mg, 3.6 mmol, 1.2 equiv.) at 0 °C and the reaction mixture was stirred at room temperature for 2 h. The reaction was then quenched with water, diluted with CH_2Cl_2, and extracted with CH_2Cl_2 three times. The combined organic layer was washed by brine, dried over Na_2SO_4, filtered, and concentrated under reduced pressure to afford the corresponding alcohol, which was directly used in the next step without further purification.

Tosylates were synthesized according to reported literature.[1] A glass tube was charged with *p*-toluene sulfonyl chloride (857.9 mg, 4.5 mmol, 1.5 equiv.) and trimethylamine hydrochloride (28.7 mg, 0.3 mmol, 0.1 equiv.) in CH_2Cl_2 (1 M with respect to the alcohol). Triethylamine (1.04 mL, 7.5 mmol, 2.5 equiv.) was added dropwise at 0 °C to the solution. To the resulting mixture was added a solution of the alcohol obtained above (3.0 mmol, 1.0 equiv.) in CH_2Cl_2 (1 M), and the mixture was then stirred at 0 °C for 2 h. The reaction was quenched by addition of *N*,*N*-dimethylethylenediamine (0.49 mL, 4.5 mmol, 1.5 equiv.) and stirred for 10 min. The reaction was mixed with water and extracted 3 times with CH_2Cl_2. The organic layer was washed sequentially with 1 M HCl, saturated aqueous Na_2CO_3 and brine. The combined organic layer was dried over Na_2SO_4, and filtered through a pad of Celite (Ø 3 mm x 8 mm). The product was isolated by flash column chromatography.

4-Phenylbutan-2-yl 4-methylbenzenesulfonate 1a

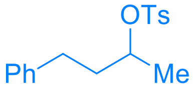

According to **General procedure 1** with 4-phenylbutan-2-one (444.6 mg, 3.0 mmol, 1.0 equiv.), the reaction mixture was purified by column chromatography on silica gel (hexane/EtOAc = 10/1) to yield the product **1a** as a white solid (785.4 mg, 2.58 mmol, 86% yield over two steps). **^{1}H NMR** (400 MHz, $CDCl_3$): δ = 7.78 (d, J = 8 Hz, 2H), 7.31 (d, J = 8 Hz, 2H), 7.26 – 7.16 (m, 3H), 7.05 (d, J = 7 Hz, 2H), 4.69 – 4.58 (m, 1H), 2.65 – 2.47 (m, 2H), 2.43 (s, 3H), 1.99 – 1.73 (m, 2H), 1.29 (d, J = 6 Hz, 3H). **^{13}C{^{1}H} NMR** (100 MHz,

$CDCl_3$): δ = 144.6, 141.0, 134.6, 129.9, 128.6, 128.4, 127.9, 126.2, 80.0, 38.3, 31.3, 21.8, 21.0. **HRMS-ASAP** (m/z): Calculated (found) for $C_{17}H_{21}O_3S$ $[M+H]^+$ 305.1206 (305.1201).

The spectroscopic data for **1a** match those reported in the literature.[1]

1,3-Diphenylpropan-2-yl 4-methylbenzenesulfonate 22a

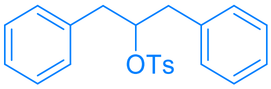

According to **General procedure 1** with 1,3-diphenylpropan-2-one (630.8 mg, 3.0 mmol, 1.0 equiv.), the reaction mixture was purified by column chromatography on silica gel (hexane/EtOAc = 10/1) to yield the product **22a** as a white solid (802.6 mg, 2.19 mmol, 73% yield over two steps). **^{1}H NMR** (400 MHz, $CDCl_3$): δ = 7.41 – 7.38 (m, 2H), 7.22 – 7.18 (m, 6H), 7.07 – 7.05 (m, 6H), 4.85 – 4.79 (m, 1H), 2.99 – 2.89 (m, 4H), 2.37 (s, 3H). **^{13}C{^{1}H} NMR** (100 MHz, $CDCl_3$): δ = 144.1, 136.4, 133.4, 129.7, 129.6, 128.6, 127.6, 126.8, 84.7, 40.6, 21.7. **HRMS-ASAP** (m/z): Calculated (found) for $C_{22}H_{23}O_3S$ $[M+H]^+$ 367.1362 (367.1358).

The spectroscopic data for **22a** match those reported in the literature.[1]

1-Cyclohexylethyl 4-methylbenzenesulfonate 23a

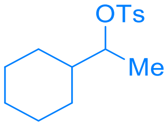

According to **General procedure 1** with 1-cyclohexylethanone (378.6 mg, 3.0 mmol, 1.0 equiv.), the reaction mixture was purified by column chromatography on silica gel (hexane/EtOAc = 10/1) to yield the product **23a** as a white solid (601.5 mg, 2.13 mmol, 71% yield over two steps). **^{1}H NMR** (400 MHz, $CDCl_3$): δ = 7.78 (d, J = 8 Hz, 2H), 7.32 (d, J = 8 Hz, 2H), 4.47 – 4.41 (m, 1H), 2.44 (s, 3H), 1.73 – 1.55 (m, 6H), 1.49 – 1.41 (m, 1H), 1.19 (d, J = 6 Hz, 3H), 1.17 – 0.80 (m, 4H). **^{13}C{^{1}H} NMR** (100 MHz, $CDCl_3$): δ = 144.5, 134.7, 129.8, 127.8, 84.7, 43.2, 28.3, 28.1, 26.3, 26.0, 25.9, 21.8, 17.9. **HRMS-ASAP** (m/z): Calculated (found) for $C_{15}H_{23}O_3S$ $[M+H]^+$ 283.1362 (283.1659).

The spectroscopic data for **23a** match those reported in the literature.[1]

6-Bromo-1,2,3,4-tetrahydronaphthalen-2-yl 4-methylbenzenesulfonate 28a

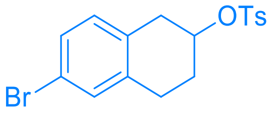

According to **General procedure 1** with 6-bromo-3,4-dihydronaphthalen-2(1*H*)-one (675.2 mg, 3.0 mmol, 1.0 equiv.), the reaction mixture was purified by column chromatography on silica gel (hexane/EtOAc = 10/1) to yield the product **28a** as a white solid (823.6 mg, 2.16 mmol, 72% yield over two steps). **^{1}H NMR** (300 MHz, $CDCl_3$): δ = 7.80 (d, J = 8 Hz, 2H), 7.34 (d, J = 8 Hz, 2H), 7.23 – 7.19 (m, 2H), 6.85 – 6.83 (m, 1H), 4.97 – 4.90 (m, 1H), 3.00 – 2.68 (m, 4H), 2.46 (s, 3H), 2.06 – 1.96 (m, 2H). **^{13}C{^{1}H} NMR** (75 MHz, $CDCl_3$): δ = 144.9, 137.3, 134.5, 131.49, 131.48, 130.9, 130.0, 129.3, 127.8, 120.1, 34.8, 28.2, 25.8, 21.8. **HRMS-ASAP** (m/z): Calculated (found) for $C_{17}H_{18}BrO_3S$ $[M+H]^+$ 381.0155 (381.0151). **Anal.** for $C_{17}H_{17}BrO_3S$ calcd: C, 53.55; H, 4.49; S, 8.41. found: C, 53.46; H, 4.36; S, 8.46.

1-Phenylpropan-2-yl 4-methylbenzenesulfonate 29a

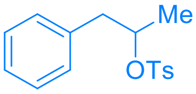

According to **General procedure 1** with 1-phenylpropan-2-one (402.5 mg, 3.0 mmol, 1.0 equiv.), the reaction mixture was purified by column chromatography on silica gel (hexane/EtOAc = 10/1) to yield the product **29a** as a white solid (740.5 mg, 2.55 mmol, 85% yield over two steps). **^{1}H NMR** (400 MHz, $CDCl_3$): δ = 7.61 (d, J = 8 Hz, 2H), 7.23 – 7.18 (m, 5H), 7.04 – 7.02 (m, 2H), 4.77 – 4.69 (m, 1H), 2.91 (dd, J = 7, 14 Hz, 1H), 2.78 (dd, J = 7, 14 Hz, 1H), 2.41 (s, 3H), 1.30 (d, J = 6 Hz, 3H). **^{13}C{^{1}H} NMR** (100 MHz, $CDCl_3$): δ = 144.4, 136.4, 134.0, 129.8, 129.6, 128.5, 127.7, 126.8, 80.8, 43.0, 21.7, 20.7. **HRMS-ASAP** (m/z): Calculated (found) for $C_{16}H_{19}O_3S$ $[M+H]^+$ 291.1049 (291.1047).

The spectroscopic data for **29a** match those reported in the literature.[2]

1-(4-Fluorophenyl)propan-2-yl 4-methylbenzenesulfonate 30a

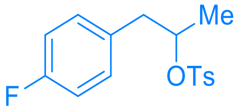

According to **General procedure 1** with 1-(4-fluorophenyl)propan-2-one (456.5 mg, 3.0 mmol, 1.0 equiv.), the reaction mixture was purified by column chromatography on silica gel (hexane/EtOAc = 10/1) to yield the product **30a** as a white solid (721.6 mg, 2.34 mmol, 78% yield over two steps). **^{1}H NMR** (400 MHz, $CDCl_3$): δ = 7.56 (d, J = 8 Hz, 2H), 7.20 (d, J = 8 Hz, 2H), 6.99 – 6.94 (m, 2H), 6.86 – 6.80 (m, 2H), 4.72 – 4.64 (m, 1H), 2.84 (dd, J = 7, 14 Hz, 1H), 2.76 (dd, J = 7, 14 Hz, 1H), 2.42 (s, 3H), 1.33 (d, J = 6 Hz, 3H). **^{13}C{^{1}H} NMR** (100 MHz, $CDCl_3$): δ = 161.9 (d, J = 244 Hz), 144.5, 133.9, 132.1 (d, J = 3 Hz), 130.9 (d, J = 8 Hz), 129.7, 127.7, 115.3 (d, J = 21 Hz), 80.8 (d, J = 2 Hz), 42.2, 21.7, 20.9. **^{19}F{^{1}H} NMR** (376 MHz, $CDCl_3$): δ = -116.2 (s). **HRMS-ASAP** (m/z): Calculated (found) for $C_{16}H_{18}FO_3S$ $[M+H]^+$ 309.0955 (309.0951).

The spectroscopic data for **30a** match those reported in the literature.[2]

3-Methylbutan-2-yl 4-methylbenzenesulfonate 31a

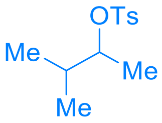

According to **General procedure 1** with 3-methylbutan-2-one (258.4 mg, 3.0 mmol, 1.0 equiv.), the reaction mixture was purified by column chromatography on silica gel (hexane/EtOAc = 20/1) to yield the product **31a** as a white solid (625.2 mg, 2.58 mmol, 86% yield over two steps). **^{1}H NMR** (400 MHz, $CDCl_3$): δ = 7.79 (d, J = 8 Hz, 2H), 7.33 (d, J = 8 Hz, 2H), 4.48 – 4.42 (m, 1H), 2.44 (s, 3H), 1.83 – 1.75 (m, 1H), 1.20 (d, J = 6 Hz, 3H), 0.83 (dd, J = 5, 7 Hz, 6H). **^{13}C{^{1}H} NMR** (100 MHz, $CDCl_3$): δ = 144.5, 134.7, 129.8, 127.8, 85.0, 33.4, 21.8, 17.9, 17.7, 17.5. **HRMS-ASAP** (m/z): Calculated (found) for $C_{12}H_{19}O_3S$ $[M+H]^+$ 243.1049 (243.1045). **Anal.** for $C_{12}H_{18}O_3S$ calcd: C, 59.47; H, 7.49; S, 13.23. found: C, 59.54; H, 7.41; S, 13.37.

1-(Benzo[*d*][1,3]dioxol-5-yl)-2-methylpentan-3-yl 4-methylbenzenesulfonate 32a

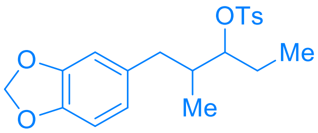

According to **General procedure 1** with 1-(benzo[*d*][1,3]dioxol-5-yl)-2-methylpentan-3-one (660.8 mg, 3.0 mmol, 1.0 equiv.), the reaction mixture was purified by column

chromatography on silica gel (hexane/EtOAc = 10/1) to yield the product **32a** as a white solid (835.8 mg, 2.22 mmol, 74% yield over two steps). **^{1}H NMR** (500 MHz, $CDCl_3$): δ = 7.82 (d, J = 8 Hz, 2H), 7.34 (d, J = 8 Hz, 2H), 6.70 (d, J = 8 Hz, 1H), 6.50 – 6.49 (m, 2H), 5.93 (dd, J = 2, 5 Hz, 2H), 4.56 – 4.53 (m, 1H), 2.68 (dd, J = 5, 14 Hz, 1H), 2.45 (s, 3H), 2.21 – 2.18 (m, 1H), 2.02 – 1.98 (m, 1H), 1.69 – 1.64 (m, 2H), 0.84 (q, J = 7 Hz, 6H). **^{13}C{^{1}H} NMR** (125 MHz, $CDCl_3$): δ = 147.7, 146.0, 144.6, 135.2, 134.1, 129.9, 127.9, 122.1, 109.4, 108.2, 101.0, 89.1, 38.6, 38.3, 24.0, 21.8, 14.3, 10.1. **HRMS-ASAP** (m/z): Calculated (found) for $C_{20}H_{25}O_5S$ $[M+H]^+$ 377.1417 (377.1412). **Anal.** for $C_{20}H_{24}O_5S$ calcd: C, 63.81; H, 6.43; S, 8.52. found: C, 63.92; H, 6.40; S, 8.59.

4-(4-Methoxyphenyl)butan-2-yl 4-methylbenzenesulfonate 33a

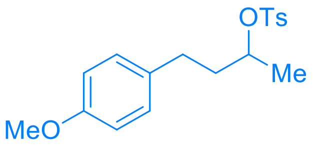

According to **General procedure 1** with 4-(4-methoxyphenyl)butan-2-one (534.7 mg, 3.0 mmol, 1.0 equiv.), the reaction mixture was purified by column chromatography on silica gel (hexane/EtOAc = 10/1) to yield the product **33a** as a white solid (822.7 mg, 2.46 mmol, 82% yield over two steps). **^{1}H NMR** (400 MHz, $CDCl_3$): δ = 7.81 – 7.78 (m, 2H), 7.34 – 7.32 (m, 2H), 6.99 – 6.96 (m, 2H), 6.81 – 6.77 (m, 2H), 4.66 – 4.59 (m, 1H), 3.78 (s, 3H), 2.58 – 2.51 (m, 1H), 2.47 – 2.40 (m, 4H), 1.94 – 1.85 (m, 1H), 1.80 – 1.72 (m, 1H), 1.29 (d, J = 6 Hz, 3H). **^{13}C{^{1}H} NMR** (100 MHz, $CDCl_3$): δ = 158.0, 144.6, 134.5, 133.0, 129.9, 129.3, 127.9, 113.9, 80.1, 55.4, 38.5, 30.4, 21.8, 21.0. **HRMS-ASAP** (m/z): Calculated (found) for $C_{18}H_{23}O_4S$ $[M+H]^+$ 335.1312 (335.1310).

The spectroscopic data for **33a** match those reported in the literature.[1]

4-Methylpentan-2-yl 4-methylbenzenesulfonate 34a

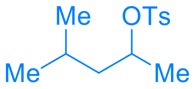

According to **General procedure 1** with 4-methylpentan-2-ol (306.5 mg, 3.0 mmol, 1.0 equiv.), the reaction mixture was purified by column chromatography on silica gel (hexane/EtOAc = 10/1) to yield the product **34a** as a white solid (707.6 mg, 2.76 mmol, 92% yield). **^{1}H NMR** (300 MHz, $CDCl_3$): δ = 7.80 (d, J = 8 Hz, 2H), 7.33 (d, J = 8 Hz, 2H), 4.73 –

4.63 (m, 1H), 2.44 (s, 3H), 1.62 – 1.52 (m, 2H), 1.31 – 1.20 (m, 4H), 0.81 (d, J = 6 Hz, 3H), 0.75 (d, J = 6 Hz, 3H). **^{13}C{^{1}H} NMR** (75 MHz, $CDCl_3$): δ = 144.5, 134.8, 129.8, 127.9, 79.3, 46.0, 24.4, 22.9, 22.1, 21.8, 21.5. **HRMS-ASAP** (m/z): Calculated (found) for $C_{13}H_{21}O_3S$ $[M+H]^+$ 257.1206 (257.1201).

The spectroscopic data for **34a** match those reported in the literature.[3]

5-Chloropentan-2-yl 4-methylbenzenesulfonate 37a

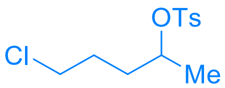

According to **General procedure 1** with 5-chloropentan-2-one (361.7 mg, 3.0 mmol, 1.0 equiv.), the reaction mixture was purified by column chromatography on silica gel (hexane/EtOAc = 10/1) to yield the product **37a** as a white solid (622.7 mg, 2.25 mmol, 75% yield over two steps). **^{1}H NMR** (400 MHz, $CDCl_3$): δ = 7.78 (d, J = 8 Hz, 2H), 7.33 (d, J = 8 Hz, 2H), 4.66 – 4.59 (m, 1H), 3.49 – 3.40 (m, 2H), 2.43 (s, 3H), 1.81 – 1.63 (m, 4H), 1.24 (d, J = 6 Hz, 3H). **^{13}C{^{1}H} NMR** (100 MHz, $CDCl_3$): δ = 144.8, 134.3, 129.9, 127.8, 79.5, 44.5, 33.8, 27.9, 21.7, 21.0. **HRMS-ASAP** (m/z): Calculated (found) for $C_{12}H_{18}ClO_3S$ $[M+H]^+$ 277.0660 (277.0654).

The spectroscopic data for **37a** match those reported in the literature.[1]

1-(3,4-Dimethoxyphenyl)propan-2-yl 4-methylbenzenesulfonate 55a

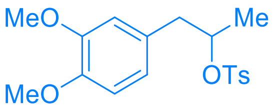

According to **General procedure 1** with 1-(3,4-dimethoxyphenyl)propan-2-one (582.7 mg, 3.0 mmol, 1.0 equiv.), the reaction mixture was purified by column chromatography on silica gel (hexane/EtOAc = 5/1) to yield the product **55a** as a white solid (735.9 mg, 2.10 mmol, 70% yield over two steps). **^{1}H NMR** (300 MHz, $CDCl_3$): δ = 7.56 (d, J = 8 Hz, 2H), 7.18 (d, J = 8 Hz, 2H), 6.68 (d, J = 8 Hz, 1H), 6.58 (dd, J = 2, 8 Hz, 1H), 6.464 – 6.458 (m, 1H), 4.75 – 4.64 (m, 1H), 3.85 (s, 3H), 3.75 (s, 3H), 2.86 – 2.68 (m, 2H), 2.40 (s, 3H), 1.34 (d, J = 6 Hz, 3H). **^{13}C{^{1}H} NMR** (75 MHz, $CDCl_3$): δ = 148.8, 148.0, 144.4, 134.1, 129.6, 129.0, 127.7, 121.6, 112.4, 111.1, 81.1, 55.9, 55.7, 42.7, 21.7, 21.9. **HRMS-ASAP** (m/z): Calculated

(found) for $C_{18}H_{22}O_5S$ [M] 350.1182 (350.1175). **Anal.** for $C_{18}H_{22}O_5S$ calcd: C, 61.69; H, 6.33; S, 9.15. found: C, 61.54; H, 6.42; S, 9.16.

The synthesis of substrate 56a

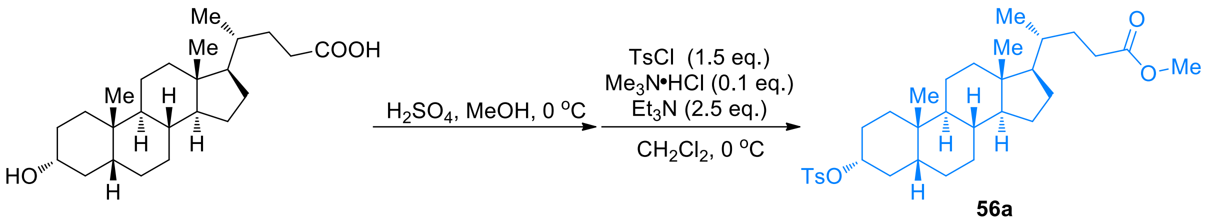

56a

Conc. H_2SO_4 (0.50 mL) was added in portions to a solution of lithocholic acid (1.13 g, 3 mmol, 1.0 equiv.) in MeOH (12 mL). The reaction mixture was stirred for 24 h at 70 °C. Afterwards, the reaction mixture was neutralized with diluted solution of $NaHCO_3$ and the aqueous layer was extracted three times with CH_2Cl_2 and the combined organic layers were washed with brine, dried over Na_2SO_4 and concentrated under reduced pressure. Then, a glass tube was charged with *p*-toluene sulfonyl chloride (857.9 mg, 4.5 mmol, 1.5 equiv.) and trimethylamine hydrochloride (28.7 mg, 0.3 mmol, 0.1 equiv.) in CH_2Cl_2 (1 M with respect to the alcohol). Triethylamine (1.04 mL, 7.5 mmol, 2.5 equiv.) was added dropwise at 0 °C to the solution. To the resulting mixture was added a solution of the alcohol obtained above (3.0 mmol, 1.0 equiv.) in CH_2Cl_2 (1 M), and the mixture was then stirred at 0 °C for 2 h. The reaction was quenched by addition of *N*,*N*-dimethylethylenediamine (0.49 mL, 4.5 mmol, 1.5 equiv.) and stirred for 10 min. The reaction was mixed with water and extracted 3 times with CH_2Cl_2. The organic layer was washed sequentially with 1 M HCl, saturated aqueous Na_2CO_3 and brine. The combined organic layer was dried over Na_2SO_4, and filtered through a pad of Celite (Ø 3 mm x 8 mm). The reaction mixture was purified by flash column chromatography on silica gel (hexane/EtOAc = 90/10) to yield the product **56a** as a white solid (1.03 mg, 1.89 mmol, 63% yield over two steps).

(*R*)-methyl 4-((3*R*,5*R*,8*R*,9*S*,10*S*,13*R*,14*S*,17*R*)-10,13-dimethyl-3-(tosyloxy)hexadecahydro-1*H*-cyclopenta[*a*]phenanthren-17-yl)pentanoate 56a

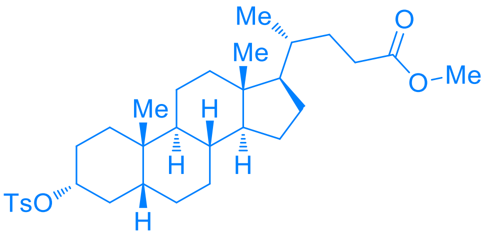

^{1}H NMR (300 MHz, $CDCl_3$): δ = 7.78 (d, J = 8 Hz, 2H), 7.31 (d, J = 8 Hz, 2H), 4.50 – 4.39 (m, 1H), 3.65 (s, 3H), 2.43 (s, 3H), 2.37 – 2.15 (m, 2H), 2.03 – 0.94 (m, 26H), 0.90– 0.87 (m, 6H), 0.61 (s, 3H). **^{13}C{^{1}H} NMR** (75 MHz, $CDCl_3$): δ = 174.8, 144.4, 134.9, 129.8, 127.7, 83.3, 56.5, 56.0, 51.6, 42.8, 42.2, 40.4, 40.1, 35.8, 35.4, 35.1, 34.4, 33.2, 31.14, 31.1, 28.3, 27.7, 26.9, 26.3, 24.3, 23.2, 21.7, 20.9, 18.4, 12.1. **HRMS-ASAP** (m/z): Calculated (found) for $C_{32}H_{49}O_5S$ $[M+H]^+$ 545.3295 (545.3288).

The spectroscopic data for **56a** match those reported in the literature.[4]

(3*S*,8*R*,9*S*,10*S*,13*S*,14*S*)-10,13-Dimethylhexadecahydrospiro[cyclopenta[*a*]phenanthrene-17,2'-[1,3]dioxolan]-3-yl 4-methylbenzenesulfonate 57a

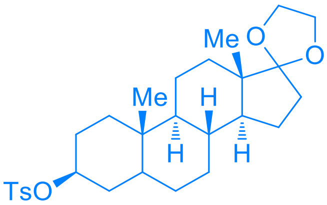

According to **General procedure 1** with (3*S*,8*R*,9*S*,10*S*,13*S*,14*S*)-10,13-dimethylhexadecahydrospiro[cyclopenta[*a*]phenanthrene-17,2'-[1,3]dioxolan]-3-ol (1.003 g, 3.0 mmol, 1.0 equiv.), the reaction mixture was purified by column chromatography on silica gel (hexane/EtOAc = 90/10) to yield the product **57a** as a white solid (1.085 g, 2.22 mmol, 74% yield). **^{1}H NMR** (500 MHz, $CDCl_3$): δ = 7.78 (d, J = 8 Hz, 2H), 7.32 (d, J = 8 Hz, 2H), 4.49 – 4.46 (m, 1H), 4.43 – 4.35 (m, 1H), 4.24 – 4.20 (m, 1H), 4.14 – 4.09 (m, 1H), 3.98 – 3.87 (m, 2H), 2.44 (s, 3H), 2.08 – 2.00 (m, 1H), 1.98 – 1.92 (m, 1H), 1.79 – 1.44 (m, 10H), 1.37 – 1.13 (m, 6H), 0.94 – 0.86 (m, 2H), 0.84 (s, 3H), 0.76 (s, 3H), 0.66 – 0.60 (m, 1H). **^{13}C{^{1}H} NMR** (125 MHz, $CDCl_3$): δ = 144.5, 134.8, 129.9, 127.7, 116.9, 82.4, 66.6, 66.3, 55.5, 53.8, 48.4, 45.6, 44.8, 36.8, 35.5, 35.4, 35.2, 34.9, 31.0, 30.6, 28.4, 28.2, 21.8, 20.4, 14.8, 12.2. **HRMS-**

ASAP (m/z): Calculated (found) for $C_{28}H_{41}O_5S$ $[M+H]^+$ 489.2669 (489.2663). **Anal.** for $C_{28}H_{40}O_5S$ calcd: C, 68.82; H, 8.25; S, 6.56. found: C, 68.67; H, 8.11; S, 6.74.

<u>(2a*S*,2'*R*,4*R*,5'*R*,6a*S*,6b*S*,8a*S*,8b*R*,11a*S*,12a*S*,12b*R*)-5',6a,8a-Trimethyldocosahydrospiro[naphtho[2',1':4,5]indeno[2,1-*b*]furan-10,2'-pyran]-4-yl 4-methylbenzenesulfonate 58a</u>

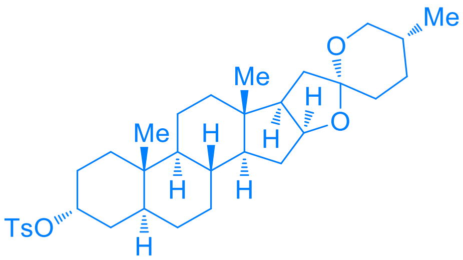

According to **General procedure 1** with (2a*S*,2'*R*,4*R*,5'*R*,6a*S*,6b*S*,8a*S*,8b*R*,11a*S*,12a*S*,12b*R*)-5',6a,8a-trimethyldocosahydrospiro[naphtho[2',1':4,5]indeno[2,1-*b*]furan-10,2'-pyran]-4-ol (1.208 g, 3.0 mmol, 1.0 equiv.), the reaction mixture was purified by column chromatography on silica gel (hexane/EtOAc = 90/10) to yield the product **58a** as a white solid (1.186 g, 2.13 mmol, 71% yield). **^{1}H NMR** (300 MHz, $CDCl_3$): δ = 7.78 (d, J = 8 Hz, 2H), 7.32 (d, J = 8 Hz, 2H), 4.46 – 4.33 (m, 2H), 3.49 – 3.32 (m, 2H), 2.44 (s, 3H), 1.99 – 1.91 (m, 1H), 1.89 – 1.82 (m, 1H), 1.76 – 1.40 (m, 13H), 1.28 – 1.00 (m, 7H), 0.95 (d, J = 7 Hz, 3H), 0.91 – 0.83 (m, 2H), 0.79 – 0.77 (m, 6H), 0.74 (s, 3H), 0.64 – 0.55 (m, 1H). **^{13}C{^{1}H} NMR** (75 MHz, $CDCl_3$): δ = 144.5, 134.9, 129.9, 127.7, 109.4, 82.6, 80.9, 67.0, 62.3, 56.3, 54.2, 44.9, 41.7, 40.7, 40.1, 36.9, 35.5, 35.1, 35.0, 32.2, 31.9, 31.5, 30.4, 28.9, 28.5, 21.8, 21.1, 17.3, 16.6, 14.6, 12.3. **HRMS-ASAP** (m/z): Calculated (found) for $C_{33}H_{49}O_5S$ $[M+H]^+$ 557.3295 (557.3287). **Anal.** for $C_{33}H_{48}O_5S$ calcd: C, 71.18; H, 8.69; S, 5.76. found: C, 71.26; H, 8.68; S, 5.89.

<u>(3*S*,8*R*,9*S*,10*S*,13*S*,14*S*)-10,13-Dimethyl-17-oxohexadecahydro-1*H*-cyclopenta[*a*]phenanthren-3-yl 4-methylbenzenesulfonate 59a</u>

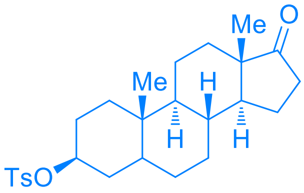

According to **General procedure 1** with (3*S*,8*R*,9*S*,10*S*,13*S*,14*S*)-3-hydroxy-10,13-dimethyltetradecahydro-1*H*-cyclopenta[*a*]phenanthren-17(2*H*)-one (871.3 mg, 3.0 mmol, 1.0

equiv.), the reaction mixture was purified by column chromatography on silica gel (hexane/EtOAc = 90/10) to yield the product **59a** as a white solid (973.7 mg, 2.19 mmol, 73% yield). **^{1}H NMR** (300 MHz, $CDCl_3$): δ = 7.78 (d, J = 8 Hz, 2H), 7.32 (d, J = 8 Hz, 2H), 4.46 – 4.35 (m, 1H), 2.47 – 2.38 (m, 4H), 2.11 – 1.99 (m, 1H), 1.95 – 1.86 (m, 1H), 1.79 – 1.44 (m, 10H), 1.30 – 1.04 (m, 6H), 0.99 – 0.88 (m, 2H), 0.83 (s, 3H), 0.80 (s, 3H), 0.68 – 0.60 (m, 1H). **^{13}C{^{1}H} NMR** (75 MHz, $CDCl_3$): δ = 221.2, 144.5, 134.8, 129.9, 127.7, 82.3, 54.3, 51.5, 47.9, 44.9, 36.8, 35.9, 35.5, 35.1, 34.9, 31.6, 30.8, 28.4, 28.2, 21.9, 21.8, 20.6, 13.9, 12.2. **HRMS-ASAP** (m/z): Calculated (found) for $C_{26}H_{37}O_4S$ $[M+H]^+$ 445.2407 (445.2401). **Anal.** for $C_{26}H_{36}O_4S$ calcd: C, 70.23; H, 8.16; S, 7.21. found: C, 70.11; H, 8.23; S, 7.37.

The spectroscopic data for **59a** match those reported in the literature.[5]

General procedure 2: Preparation of secondary alkyl bromides

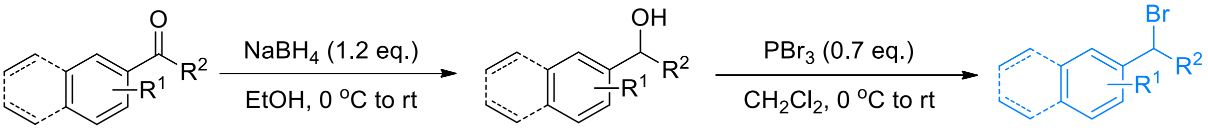

To a solution of ketone (3.0 mmol, 1.0 equiv.) in EtOH (9.0 mL) was added $NaBH_4$ (136.8 mg, 3.6 mmol, 1.2 equiv.) at 0 °C and the reaction mixture was stirred at room temperature for 2 h. The reaction was then quenched with water, diluted with CH_2Cl_2, and extracted with CH_2Cl_2 three times. The combined organic layer was washed by brine, dried over Na_2SO_4, filtered, and concentrated under reduced pressure to afford the corresponding alcohol, which was directly used in the next step without further purification.

To a solution of the residue obtained above (if it is a commercially available alcohol, skip the above step and use it directly in this step) in CH_2Cl_2 (9.0 mL) was added PBr_3 (0.20 mL, 2.1 mmol, 0.70 equiv.) under argon at 0 °C and the resulting reaction mixture was stirred at room temperature overnight. The reaction was then quenched with water and extracted 3 times with CH_2Cl_2. The organic layer was washed with brine, and then dried over Na_2SO_4, and filtered through a pad of Celite (Ø 3 mm x 8 mm), concentrated under reduced pressure to afford the corresponding crude alkyl bromide, which was directly used in the next step without further purification or stored in a refrigerator. (The product readily decomposed in air or on silica gel).

1-(1-Bromoethyl)-4-methylbenzene 3a

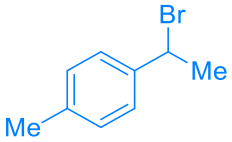

According to **General procedure 2** with 1-(p-tolyl)ethanone (402.5 mg, 3.0 mmol, 1.0 equiv.), **3a** was obtained as a yellow oil (513.7 mg, 2.58 mmol, 86% crude yield over two steps). **^{1}H NMR** (400 MHz, $CDCl_3$): δ = 7.35 (d, J = 8 Hz, 2H), 7.17 (d, J = 8 Hz, 2H), 5.23 (q, J = 7 Hz, 1H), 2.36 (s, 3H), 2.06 (d, J = 7 Hz, 3H). **^{13}C{^{1}H} NMR** (100 MHz, $CDCl_3$): δ = 140.5, 138.4, 129.5, 126.8, 50.0, 26.9, 21.3. **HRMS-ASAP** (m/z): Calculated (found) for $C_9H_{12}Br$ $[M+H]^+$ 199.0117 (199.0114).

The spectroscopic data for **3a** match those reported in the literature.[6]

1-(1-Bromoethyl)-4-methoxybenzene 4a

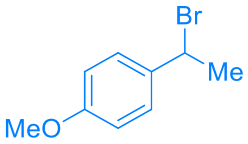

According to **General procedure 2** with 1-(4-methoxyphenyl)ethanol (456.6 mg, 3.0 mmol, 1.0 equiv.), **4a** was obtained as a yellow oil (529.1 mg, 2.46 mmol, 82% yield). **^{1}H NMR** (300 MHz, $CDCl_3$): δ = 7.38 (d, J = 9 Hz, 2H), 6.87 (d, J = 9 Hz, 2H), 5.26 (q, J = 7 Hz, 1H), 3.81 (s, 3H), 2.05 (d, J = 7 Hz, 3H). **^{13}C{^{1}H} NMR** (75 MHz, $CDCl_3$): δ = 159.6, 135.6, 128.2, 114.1, 55.5, 50.2, 27.0. **HRMS-ASAP** (m/z): Calculated (found) for $C_9H_{12}BrO$ $[M+H]^+$ 215.0066 (215.0061).

The spectroscopic data for **4a** match those reported in the literature.[7]

(4-(1-Bromoethyl)phenyl)(methyl)sulfane 5a

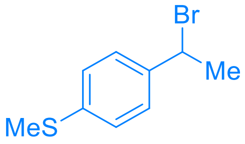

According to **General procedure 2** with 1-(4-(methylthio)phenyl)ethanone (498.7 mg, 3.0 mmol, 1.0 equiv.), **5a** was obtained as a white solid (610.2 mg, 2.64 mmol, 88% crude yield over two steps). **^{1}H NMR** (400 MHz, $CDCl_3$): δ = 7.37 – 7.34 (m, 2H), 7.23 – 7.19 (m, 2H),

5.21 (q, J = 7 Hz, 1H), 2.48 (s, 3H), 2.03 (d, J = 7 Hz, 3H). **^{13}C{^{1}H} NMR** (100 MHz, $CDCl_3$): δ = 140.0, 139.1, 127.4, 126.5, 49.6, 26.8, 15.7. **HRMS-ASAP** (m/z): Calculated (found) for $C_9H_{12}BrS$ $[M+H]^+$ 230.9838 (230.9832). **Anal.** for $C_9H_{11}BrS$ calcd: C, 46.76; H, 4.80; S, 13.87. found: C, 46.71; H, 4.91; S, 13.86.

1-(1-Bromoethyl)-4-chlorobenzene 7a

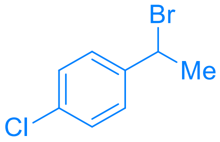

According to **General procedure 2** with 1-(4-chlorophenyl)ethanone (463.8 mg, 3.0 mmol, 1.0 equiv.), **7a** was obtained as a yellow oil (533.4 mg, 2.43 mmol, 81% crude yield over two steps). **^{1}H NMR** (400 MHz, $CDCl_3$): δ = 7.40 – 7.36 (m, 2H), 7.33 – 7.30 (m, 2H), 5.18 (q, J = 7 Hz, 1H), 2.03 (d, J = 7 Hz, 3H). **^{13}C{^{1}H} NMR** (100 MHz, $CDCl_3$): δ = 141.8, 134.1, 128.9, 128.3, 48.4, 26.8. **HRMS-ASAP** (m/z): Calculated (found) for C_8H_9BrCl $[M+H]^+$ 218.9571 (218.9567).

The spectroscopic data for **7a** match those reported in the literature.[7]

1-Bromo-4-(1-bromoethyl)benzene 8a

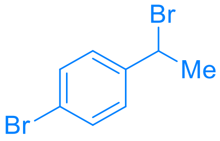

According to **General procedure 2** with 1-(4-bromophenyl)ethanone (597.1 mg, 3.0 mmol, 1.0 equiv.), **8a** was obtained as a yellow oil (681.0 mg, 2.58 mmol, 86% crude yield over two steps). **^{1}H NMR** (400 MHz, $CDCl_3$): δ = 7.49 – 7.45 (m, 2H), 7.33 – 7.30 (m, 2H), 5.15 (q, J = 7 Hz, 1H), 2.02 (d, J = 7 Hz, 3H). **^{13}C{^{1}H} NMR** (100 MHz, $CDCl_3$): δ = 142.4, 132.0, 128.6, 122.3, 48.4, 26.8. **HRMS-ASAP** (m/z): Calculated (found) for $C_8H_9Br_2$ $[M+H]^+$ 262.9066 (262.9061).

The spectroscopic data for **8a** match those reported in the literature.[8]

1-(1-Bromoethyl)-4-iodobenzene 9a

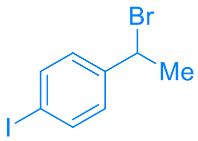

According to **General procedure 2** with 1-(4-iodophenyl)ethanone (738.1 mg, 3.0 mmol, 1.0 equiv.), **9a** was obtained as a yellow oil (932.9 mg, 2.34 mmol, 78% crude yield over two steps). **^{1}H NMR** (300 MHz, $CDCl_3$): δ = 7.68 (d, J = 8 Hz, 2H), 7.18 (d, J = 8 Hz, 2H), 5.13 (q, J = 7 Hz, 1H), 2.01 (d, J = 7 Hz, 3H). **^{13}C{^{1}H} NMR** (75 MHz, $CDCl_3$): δ = 143.1, 137.9, 128.8, 94.0, 48.4, 26.8. **HRMS-ASAP** (m/z): Calculated (found) for C_8H_9BrI $[M+H]^+$ 310.8927 (310.8923).

The spectroscopic data for **9a** match those reported in the literature.[9]

2-(1-Bromoethyl)naphthalene 10a

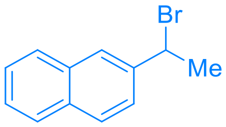

According to **General procedure 2** with 1-(naphthalen-2-yl)ethanone (510.6 mg, 3.0 mmol, 1.0 equiv.), **10a** was obtained as a white solid (557.2 mg, 2.37 mmol, 79% crude yield over two steps). **^{1}H NMR** (400 MHz, $CDCl_3$): δ = 7.87 – 7.81 (m, 4H), 7.61 (dd, J = 8, 2 Hz, 1H), 7.53 – 7.48 (m, 2H), 5.41 (q, J = 7 Hz, 1H), 2.16 (d, J = 7 Hz, 3H). **^{13}C{^{1}H} NMR** (100 MHz, $CDCl_3$): δ = 140.5, 133.3, 133.1, 128.8, 128.2, 127.8, 126.62, 126.60, 125.3, 125.2, 50.2, 26.8. **HRMS-ASAP** (m/z): Calculated (found) for $C_{12}H_{12}Br$ $[M+H]^+$ 235.0117 (235.0114).

The spectroscopic data for **10a** match those reported in the literature.[6]

(1-Bromobutyl)benzene 11a

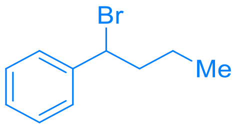

According to **General procedure 2** with 1-phenylbutan-1-one (444.6 mg, 3.0 mmol, 1.0 equiv.), **11a** was obtained as a colorless oil (537.0 mg, 2.52 mmol, 84% crude yield over two steps). **^{1}H NMR** (400 MHz, $CDCl_3$): δ = 7.43 – 7.40 (m, 2H), 7.38 – 7.34 (m, 2H), 7.32 – 7.28 (m, 1H), 5.00 (t, J = 7 Hz, 1H), 2.35 – 2.26 (m, 1H), 2.18 – 2.09 (m, 1H), 1.58 – 1.48 (m,

1H), 1.41 – 1.31 (m, 1H), 0.96 (t, J = 7 Hz, 3H). **^{13}C{^{1}H} NMR** (100 MHz, $CDCl_3$): δ = 142.4, 128.8, 128.4, 127.4, 55.6, 42.1, 21.6, 13.5. **HRMS-ASAP** (m/z): Calculated (found) for $C_{10}H_{14}Br$ $[M+H]^+$ 213.0273 (213.0269).

The spectroscopic data for **11a** match those reported in the literature.[6]

1-Bromo-4-(1-bromopropyl)benzene 13a

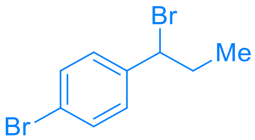

According to **General procedure 2** with 1-(4-bromophenyl)propan-1-one (639.2, 3.0 mmol, 1.0 equiv.), **13a** was obtained as a colorless oil (683.8 mg, 2.46 mmol, 82% crude yield over two steps). **^{1}H NMR** (400 MHz, $CDCl_3$): δ = 7.49 – 7.45 (m, 2H), 7.28 – 7.25 (m, 2H), 4.82 (t, J = 7 Hz, 1H), 2.32 – 2.21 (m, 1H), 2.18 – 2.07 (m, 1H), 0.99 (t, J = 7 Hz, 3H). **^{13}C{^{1}H} NMR** (100 MHz, $CDCl_3$): δ = 141.3, 131.9, 129.1, 122.2, 56.3, 33.3, 13.1. **HRMS-ASAP** (m/z): Calculated (found) for $C_9H_{11}Br_2$ $[M+H]^+$ 276.9222 (276.9218).

The spectroscopic data for **13a** match those reported in the literature.[6]

1-(1-Bromopropyl)-4-fluorobenzene 14a

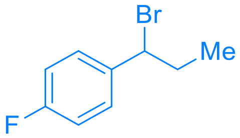

According to **General procedure 2** with 1-(4-fluorophenyl)propan-1-one (456.5 mg, 3.0 mmol, 1.0 equiv.), **14a** was obtained as a colorless oil (566.6 mg, 2.61 mmol, 87% crude yield over two steps). **^{1}H NMR** (400 MHz, $CDCl_3$): δ = 7.40 – 7.35 (m, 2H), 7.06 – 7.00 (m, 2H), 4.88 (t, J = 7 Hz, 1H), 2.34 – 2.23 (m, 1H), 2.20 – 2.09 (m, 1H), 1.00 (t, J = 7 Hz, 3H). **^{13}C{^{1}H} NMR** (100 MHz, $CDCl_3$): δ = 162.4 (d, J = 246 Hz), 138.2 (d, J = 3 Hz), 129.1 (d, J = 8 Hz), 115.7 (d, J = 21 Hz), 56.6, 33.6, 13.1. **^{19}F{^{1}H} NMR** (376 MHz, $CDCl_3$): δ = -116.2 (s). **HRMS-ASAP** (m/z): Calculated (found) for $C_9H_{11}BrF$ $[M+H]^+$ 217.0023 (217.0018).

The spectroscopic data for **14a** match those reported in the literature.[10]

1-(1-Bromopropyl)-3-methoxybenzene 15a

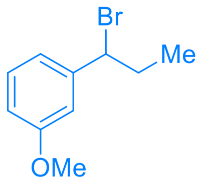

According to **General procedure 2** with 1-(3-methoxyphenyl)propan-1-one (492.6 mg, 3.0 mmol, 1.0 equiv.), **15a** was obtained as a colorless oil (556.7 mg, 2.43 mmol, 81% crude yield over two steps). **^{1}H NMR** (400 MHz, $CDCl_3$): δ = 7.27 (t, J = 8 Hz, 1H), 6.99 (d, J = 8 Hz, 1H), 6.96 – 6.95 (m, 1H), 6.85 – 6.83 (m, 1H), 4.86 (t, J = 8 Hz, 1H), 3.83 (s, 3H), 2.35 – 2.24 (m, 1H), 2.23 – 2.12 (m, 1H), 1.02 (t, J = 7 Hz, 3H). **^{13}C{^{1}H} NMR** (100 MHz, $CDCl_3$): δ = 159.8, 143.7, 129.8, 119.7, 113.8, 113.1, 57.6, 55.4, 33.4, 13.1. **HRMS-ASAP** (m/z): Calculated (found) for $C_{10}H_{14}Br$ $[M+H]^+$ 229.0223 (229.0220).

The spectroscopic data for **15a** match those reported in the literature.[6]

1-(1-Bromopropyl)-2-(trifluoromethyl)benzene 16a

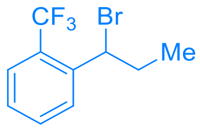

According to **General procedure 2** with 1-(2-(trifluoromethyl)phenyl)propan-1-one (606.5 mg, 3.0 mmol, 1.0 equiv.), **16a** was obtained as a colorless oil (577.0 mg, 2.16 mmol, 72% crude yield over two steps). **^{1}H NMR** (500 MHz, $CDCl_3$): δ = 7.71 (d, J = 8 Hz, 1H), 7.63 – 7.56 (m, 2H), 7.40 (t, J = 7 Hz, 1H), 5.65 – 5.61 (m, 1H), 1.93 – 1.81 (m, 2H), 0.99 (t, J = 8 Hz, 3H). **^{13}C{^{1}H} NMR** (125 MHz, $CDCl_3$): δ = 139.6 (q, J = 2 Hz), 132.4, 128.3, 128.2 126.8 (q, J = 30 Hz), 125.6 (q, J = 6 Hz), 125.2 (q, J = 272 Hz), 75.9 (q, J = 3 Hz), 32.2, 10.1. **^{19}F{^{1}H} NMR** (470 MHz, $CDCl_3$): δ = -58.1 (s). **HRMS-ASAP** (m/z): Calculated (found) for $C_{10}H_{11}BrF_3$ $[M+H]^+$ 266.9991 (266.9983). **Anal.** for $C_{10}H_{10}BrF_3$ calcd: C, 44.97; H, 3.77. found: C, 44.84; H, 3.82.

1-(4-(1-Bromopropyl)phenyl)-1*H*-pyrazole 19a

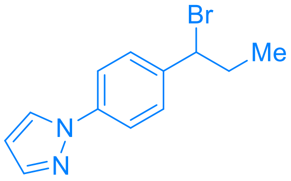

According to **General procedure 2** with 1-(4-(1*H*-pyrazol-1-yl)phenyl)propan-1-one (600.7 mg, 3.0 mmol, 1.0 equiv.), **19a** was obtained as a colorless oil (588.6 mg, 2.22 mmol, 74% crude yield over two steps). **^{1}H NMR** (300 MHz, $CDCl_3$): δ = 7.96 (d, J = 3 Hz, 1H), 7.80 (d, J = 2 Hz, 1H), 7.70 (d, J = 9 Hz, 2H), 7.49 (d, J = 9 Hz, 2H), 6.52 (t, J = 2 Hz, 1H), 4.90 (t, J = 7 Hz, 1H), 2.38 – 2.25 (m, 1H), 2.23 – 2.10 (m, 1H), 1.01 (t, J = 7 Hz, 3H). **^{13}C{^{1}H} NMR** (75 MHz, $CDCl_3$): δ = 141.1, 140.6, 139.0, 128.7, 127.1, 119.8, 108.1, 56.5, 33.3, 13.1. **HRMS-ASAP** (m/z): Calculated (found) for $C_{12}H_{14}BrN_2$ $[M+H]^+$ 265.0335 (265.0331).

The spectroscopic data for **19a** match those reported in the literature.[6]

General procedure 3: Preparation of alkyl chlorides

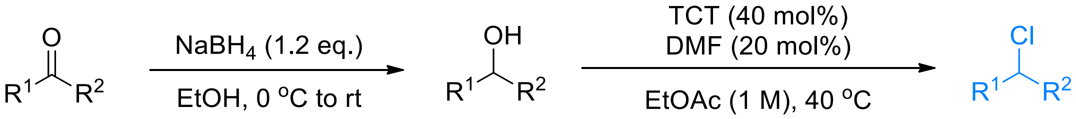

Alkyl chlorides were synthesized according to reported literature.[11a] To a solution of ketone (3.0 mmol, 1.0 equiv.) in EtOH (9.0 mL) was added $NaBH_4$ (136.8 mg, 3.6 mmol, 1.2 equiv.) at 0 °C and the reaction mixture was stirred at room temperature for 2 h. The reaction was then quenched with water, diluted with CH_2Cl_2, and extracted with CH_2Cl_2 three times. The combined organic layer was washed by brine, dried over Na_2SO_4, filtered, and concentrated under reduced pressure to afford the corresponding alcohol, which was directly used in the next step without further purification.

To a solution of the residue obtained above (if it is a commercially available alcohol, skip the above step and use it directly in this step) in EtOAc (3.0 mL) was added DMF (44.0 mg, 0.6 mmol, 20 mol%). Next, cyanuric chloride (TCT) (221.3 mg, 1.2 mmol, 40 mol%) was added in one portion and the resulting reaction mixture was stirred at 0 °C for 6 h. After completion of reaction, the reaction mixture was filtered through a pad of Celite (Ø 3 mm x 8 mm), concentrated under reduced pressure and the residue was purified by column chromatography on silica gel to provide the corresponding chlorides.

2-Chloro-2,3-dihydro-1*H*-indene 12a-1

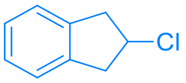

According to **General procedure 3** with 1*H*-inden-2(3*H*)-one (396.5 mg, 3.0 mmol, 1.0 equiv.), the reaction mixture was purified by column chromatography on silica gel (hexane/EtOAc = 98/2) to yield the product **12a-1** as a colorless oil (325.0 mg, 213 mmol, 71% yield over two steps). **^{1}H NMR** (300 MHz, $CDCl_3$): δ = 7.29 – 7.21 (m, 4H), 4.78 – 4.71 (m, 1H), 3.50 – 3.42 (m, 2H), 3.27 – 3.20 (m, 2H). **^{13}C{^{1}H} NMR** (75 MHz, $CDCl_3$): δ = 140.3, 127.1, 124.7, 59.3, 44.0. **HRMS-ASAP** (m/z): Calculated (found) for $C_9H_{10}Cl$ $[M+H]^+$ 153.0466 (153.0461).

The spectroscopic data for **12-1** match those reported in the literature.[11b]

1-(*tert*-Butyl)-4-(1,4-dichlorobutyl)benzene 20a

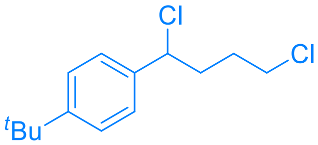

According to **General procedure 3** with 1-(4-(*tert*-butyl)phenyl)-4-chlorobutan-1-one (716.3 mg, 3.0 mmol, 1.0 equiv.), the reaction mixture was purified by column chromatography on silica gel (hexane/EtOAc = 98/2) to yield the product **20a** as a colorless oil (482.1 mg, 1.86 mmol, 62% yield over two steps). **^{1}H NMR** (300 MHz, $CDCl_3$): δ = 7.39 (d, J = 8 Hz, 2H), 7.32 (d, J = 8 Hz, 2H), 4.89 (t, J = 7 Hz, 1H), 3.60 – 3.56 (m, 2H), 2.29 – 2.21 (m, 2H), 2.10 – 1.96 (m, 1H), 1.92 – 1.78 (m, 1H), 1.33 (s, 9H). **^{13}C{^{1}H} NMR** (75 MHz, $CDCl_3$): δ = 151.6, 138.5, 126.7, 125.8, 63.0, 44.4, 37.3, 34.8, 31.4, 30.2. **HRMS-ASAP** (m/z): Calculated (found) for $C_{14}H_{21}Cl_2$ $[M+H]^+$ 259.1015 (259.1008). **Anal.** for $C_{14}H_{20}Cl_2$ calcd: C, 64.87; H, 7.78. found: C, 64.95; H, 7.74.

Octane-1,2-diyl bis(4-methylbenzenesulfonate) 40a

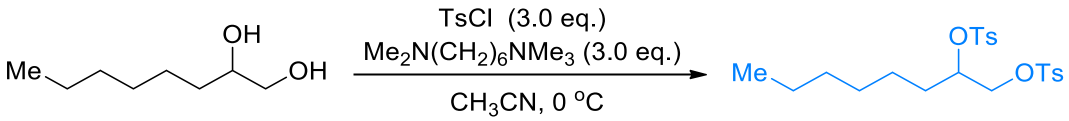

Tosylates were synthesized according to reported literature.[12] *p*-Toluene sulfonyl chloride (857.9 mg, 4.5 mmol, 3.0 equiv.) in CH_3CN (1.5 mL) was added to a stirred solution of the octane-1,2-diol (219.3 mg, 1.5 mmol, 1.0 equiv.) and $Me_2N(CH_2)_6NMe_2$ (775.4 mg, 4.5 mmol, 3.0 equiv.) in CH_3CN (1.5 mL) at 0 °C for 1 h. The reaction was quenched by addition of *N*,*N*-dimethylethylenediamine (0.49 mL, 4.5 mmol, 1.5 equiv.) and stirred for 10 min. The reaction was mixed with water and extracted 3 times with CH_2Cl_2. The organic layer was washed sequentially with 1 M HCl, saturated aqueous Na_2CO_3 and brine. The combined organic layer was dried over Na_2SO_4, and filtered through a pad of Celite (Ø 3 mm x 8 mm). The reaction mixture was purified by column chromatography on silica gel (hexane/EtOAc = 10/1) to yield the product **40a** as a white solid (392.0 mg, 1.305 mmol, 87% yield).

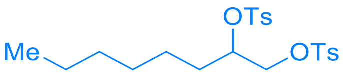

^{1}H NMR (400 MHz, $CDCl_3$): δ = 7.74 – 7.69 (m, 4H), 7.34 – 7.30 (m, 4H), 4.60 – 4.55 (m, 1H), 4.06 – 3.99 (m, 2H), 2.45 (d, J = 4 Hz, 6H), 1.63 – 1.56 (m, 2H), 1.25 – 1.04 (m, 8H), 0.84 (t, J = 7 Hz, 3H). **^{13}C{^{1}H} NMR** (100 MHz, $CDCl_3$): δ = 145.3, 145.1, 133.6, 132.4, 130.1, 129.9, 128.1, 128.0, 79.0, 69.5, 31.6, 31.1, 28.8, 24.5, 22.5, 21.80, 21.78, 14.1. **HRMS-ASAP** (m/z): Calculated (found) for $C_{22}H_{31}O_6S_2$ $[M+H]^+$ 455.1557 (455.1555).

The spectroscopic data for **40a** match those reported in the literature.[12]

General procedure 4: Preparation of tertiary alkyl bromides.

To a solution of alcohol (3.0 mmol, 1.0 equiv.) in CH_2Cl_2 was added LiBr (521 mg, 6.0 mmol, 2.0 equiv.) in 48 wt% aqueous HBr at 0 °C and the reaction mixture was stirred at room temperature for overnight. The reaction was then diluted with Et_2O, washed with water, saturated $NaHCO_3$, and brine, dried over Na_2SO_4, filtered, and concentrated under reduced pressure. The residue was purified by column chromatography to afford the product.

<u>3-Bromo-3-ethylpentane 43a</u>

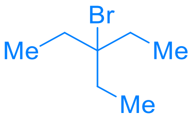

According to **General procedure 4** with 3-ethylpentan-3-ol (348.6 mg, 3.0 mmol, 1.0 equiv.), **43a** was obtained as a colorless oil (472.8 mg, 2.64 mmol, 88% yield). ^{1}H NMR (400 MHz, $CDCl_3$): δ = 1.87 (q, J = 7 Hz, 6H), 0.97 (t, J = 7 Hz, 9H). **^{13}C{^{1}H} NMR** (100 MHz, $CDCl_3$): δ = 81.8, 34.3. 9.9. **HRMS-ASAP** (m/z): Calculated (found) for $C_7H_{16}Br$ $[M+H]^+$ 179.0430 (179.0425).

The spectroscopic data for **43a** match those reported in the literature.[13]

__**(2-Bromo-2-methylpropyl)benzene 44a**__

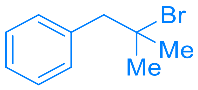

According to **General procedure 4** with 2-methyl-1-phenylpropan-2-ol (450.7 mg, 3.0 mmol, 1.0 equiv.), **44a** was obtained as a colorless oil (581.8 mg, 2.73 mmol, 91% yield). ^{1}H NMR (400 MHz, $CDCl_3$): δ = 7.36 – 7.27 (m, 4H), 3.22 (s, 2H), 1.78 (s, 6H). **^{13}C{^{1}H} NMR** (100 MHz, $CDCl_3$): δ = 137.2, 130.9, 128.1, 127.0, 66.7, 53.3, 33.9. **HRMS-ASAP** (m/z): Calculated (found) for $C_{10}H_{14}Br$ $[M+H]^+$ 213.0273 (213.0268).

The spectroscopic data for **44a** match those reported in the literature.[13]

General procedure 5:

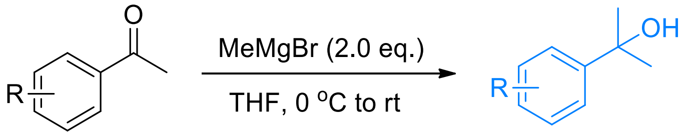

To a solution of ketone (3.0 mmol, 1.0 equiv.) in anhydrous THF (3.0 mL) was slowly added MeMgBr (1.0 M in THF, 2.0 equiv.) at 0 °C under argon atmosphere. Then the reaction mixture was warmed up to room temperature and stirred until the ketone was completely consumed (monitored by TLC). The reaction was quenched by 3.0 M HCl and extracted with CH_2Cl_2 three times. The combined organic layer was dried over Na_2SO_4, filtered, and concentrated under reduced pressure and the residue was purified by column chromatography on silica gel to provide the corresponding product.

2-(*p*-Tolyl)propan-2-ol 46a

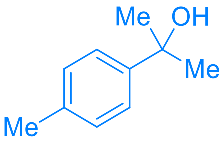

According to **General procedure 5** with 1-(*p*-tolyl)ethanone (402.6 mg, 3.0 mmol, 1.0 equiv.), the reaction mixture was purified by column chromatography on silica gel (hexane/EtOAc = 10/1) to yield the product **46a** as a colorless oil (374.0 mg, 2.49 mmol, 83% yield). **^{1}H NMR** (300 MHz, $CDCl_3$): δ = 7.39 (d, J = 8 Hz, 2H), 7.16 (d, J = 8 Hz, 2H), 2.35 (s, 3H), 1.71 (s, 1H), 1.58 (s, 6H). **^{13}C{^{1}H} NMR** (75 MHz, $CDCl_3$): δ = 146.4, 136.4, 129.0, 124.4, 72.5, 31.9, 21.1. **HRMS-ASAP** (m/z): Calculated (found) for $C_{10}H_{15}O$ $[M+H]^+$ 151.1117 (151.1114).

The spectroscopic data for **46a** match those reported in the literature.[14]

2-(*m*-Tolyl)propan-2-ol 47a

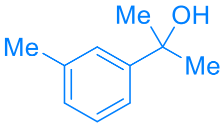

According to **General procedure 5** with 1-(*m*-tolyl)ethanone (402.6 mg, 3.0 mmol, 1.0 equiv.), the reaction mixture was purified by column chromatography on silica gel (hexane/EtOAc = 10/1) to yield the product **47a** as a colorless oil (328.9 mg, 2.19 mmol, 73% yield). **^{1}H NMR** (300 MHz, $CDCl_3$): δ = 7.32 – 7.21 (m, 3H), 7.09 – 7.05 (m, 1H), 2.37 (s, 3H), 1.73 – 1.66 (m, 1H), 1.58 (s, 6H). **^{13}C{^{1}H} NMR** (75 MHz, $CDCl_3$): δ = 149.2, 137.9, 128.3, 127.6, 125.3, 121.5, 72.6, 31.9, 21.7. **HRMS-ASAP** (m/z): Calculated (found) for $C_{10}H_{15}O$ $[M+H]^+$ 151.1117 (151.1115).

The spectroscopic data for **47a** match those reported in the literature.[15]

2-(4-Fluorophenyl)propan-2-ol 48a

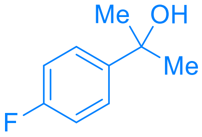

According to **General procedure 5** with 1-(4-fluorophenyl)ethanone (414.4 mg, 3.0 mmol, 1.0 equiv.), the reaction mixture was purified by column chromatography on silica gel

(hexane/EtOAc = 10/1) to yield the product **48a** as a colorless oil (328.4 mg, 2.13 mmol, 71% yield). **^{1}H NMR** (500 MHz, $CDCl_3$): δ = 7.47 – 7.44 (m, 2H), 7.03 – 6.99 (m, 2H), 1.68 (s, 1H), 1.57 (s, 6H). **^{13}C{^{1}H} NMR** (125 MHz, $CDCl_3$): δ = 161.8 (d, J = 242 Hz), 145.0 (d, J = 4 Hz), 126.3 (d, J = 8 Hz), 115.0 (d, J = 21 Hz), 72.4, 32.0. **^{19}F{^{1}H} NMR** (470 MHz, $CDCl_3$): δ = -116.8 (m). **HRMS-ASAP** (m/z): Calculated (found) for $C_9H_{12}FO$ $[M+H]^+$ 155.0867 (155.0861).

The spectroscopic data for **48a** match those reported in the literature.[16]

2-(4-Chlorophenyl)propan-2-ol 49a

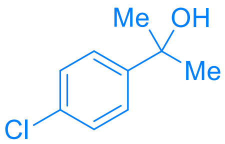

According to **General procedure 5** with 1-(4-chlorophenyl)ethanone (463.8 mg, 3.0 mmol, 1.0 equiv.), the reaction mixture was purified by column chromatography on silica gel (hexane/EtOAc = 10/1) to yield the product **49a** as a colorless oil (404.0 mg, 2.37 mmol, 79% yield). **^{1}H NMR** (300 MHz, $CDCl_3$): δ = 7.42 (d, J = 9 Hz, 2H), 7.30 (d, J = 9 Hz, 2H), 1.69 (s, 1H), 1.57 (s, 6H). **^{13}C{^{1}H} NMR** (75 MHz, $CDCl_3$): δ = 147.7, 132.6, 128.4, 126.1, 72.4, 31.9. **HRMS-ASAP** (m/z): Calculated (found) for $C_9H_{12}ClO$ $[M+H]^+$ 171.0571 (171.0568).

The spectroscopic data for **49a** match those reported in the literature.[16]

2-(4-Methoxyphenyl)propan-2-ol 50a

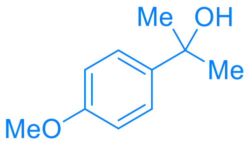

According to **General procedure 5** with 1-(4-methoxyphenyl)ethanone (450.5 mg, 3.0 mmol, 1.0 equiv.), the reaction mixture was purified by column chromatography on silica gel (hexane/EtOAc = 10/1) to yield the product **50a** as a colorless oil (349.0 mg, 2.10 mmol, 70% yield). **^{1}H NMR** (300 MHz, $CDCl_3$): δ = 7.41 (d, J = 9 Hz, 2H), 6.87 (d, J = 9 Hz, 2H), 3.80 (s, 3H), 1.73 (s, 1H), 1.57 (s, 6H). **^{13}C{^{1}H} NMR** (75 MHz, $CDCl_3$): δ = 158.5, 141.5, 125.7, 113.6, 72.3, 55.4, 31.9. **HRMS-ASAP** (m/z): Calculated (found) for $C_{10}H_{15}O_2$ $[M+H]^+$ 167.1067 (167.1064).

The spectroscopic data for **50a** match those reported in the literature.[14]

1,1-Diphenylethanol 51a

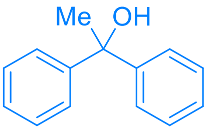

According to **General procedure 5** with benzophenone (546.7 mg, 3.0 mmol, 1.0 equiv.), the reaction mixture was purified by column chromatography on silica gel (hexane/EtOAc = 10/1) to yield the product **51a** as a white solid (487.7 mg, 2.46 mmol, 82% yield). **^{1}H NMR** (300 MHz, $CDCl_3$): δ = 7.44 (d, J = 7 Hz, 4H), 7.34 (t, J = 7 Hz, 4H), 7.26 (t, J = 7 Hz, 2H), 2.24 (s, 1H), 1.97 (s, 3H). **^{13}C{^{1}H} NMR** (75 MHz, $CDCl_3$): δ = 148.1, 128.3, 127.1, 126.0, 76.3, 31.0. **HRMS-ASAP** (m/z): Calculated (found) for $C_{14}H_{15}O$ $[M+H]^+$ 199.1117 (199.1112).

The spectroscopic data for **51a** match those reported in the literature.[17]

1-Phenylcyclopentanol 52a

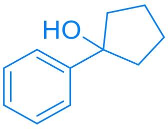

To a solution of cyclopentanone (3.0 mmol, 1.0 equiv.) in anhydrous THF (3.0 mL) was slowly added PhMgBr (1.0 M in THF, 2.0 equiv.) at 0 °C under argon atmosphere. Then the reaction mixture was warmed up to room temperature and stirred until the ketone was completely consumed (monitored by TLC). The reaction was quenched by 3.0 M HCl and extracted with CH_2Cl_2 three times. The combined organic layer was dried over Na_2SO_4, filtered, and concentrated under reduced pressure and the residue was purified by column chromatography on silica gel to provide the corresponding product **52a** as a white solid (408.8 mg, 2.52 mmol, 84% yield). **^{1}H NMR** (300 MHz, C_6D_6): δ = 7.41 – 7.37 (m, 2H), 7.23 – 7.18 (m, 2H), 7.13 – 7.07 (m, 1H), 1.92 – 1.75 (m, 6H), 1.64 – 1.57 (m, 2H), 1.00 (s, 1H). **^{13}C{^{1}H} NMR** (75 MHz, C_6D_6): δ = 147.8, 129.7, 126.8, 125.4, 83.1, 42.3, 24.2. **HRMS-ASAP** (m/z): Calculated (found) for $C_{11}H_{15}O$ $[M+H]^+$ 163.1117 (163.1114).

The spectroscopic data for **52a** match those reported in the literature.[18]

1.4 Details of the Diboration of Alkyl Halides, Tosylates, and Alcohols

General procedure 6:

In an argon-filled glovebox, alkyl halides, tosylates, or alcohols (0.3 mmol, 1.0 equiv.) in DMA (1 mL) were added to a 10 mL thick-walled reaction tube equipped with a magnetic stirring bar. KI (49.8 mg, 0.3 mmol, 1.0 equiv.) and B_2cat_2 (178 mg, 0.75 mmol, 2.5 equiv.) were added. The reaction mixture was stirred at 90 °C for 12 h, then a solution of pinacol (106 mg, 0.9 mmol, 3.0 equiv.) in Et_3N (1 mL) was added to the reaction mixture, which was stirred at room temperature for 1 h. Then water was added, and the reaction mixture was extracted with EtOAc three times. The combined organic layer was dried over Na_2SO_4, filtered, and concentrated under reduced pressure and the residue was purified by column chromatography on silica gel to provide the corresponding product.

2,2'-(1-Phenylbutane-2,3-diyl)bis(4,4,5,5-tetramethyl-1,3,2-dioxaborolane) 1b

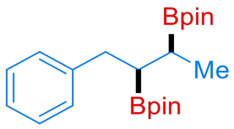

According to **General procedure 6** with 4-phenylbutan-2-yl 4-methylbenzenesulfonate **1a** (91.3 mg, 0.3 mmol, 1.0 equiv.), the reaction mixture was purified by column chromatography on silica gel (hexane/EtOAc = 97/3) to yield the product **1b** as a colorless oil (99.6 mg, 258 μmol, 86% yield). **^{1}H NMR** (500 MHz, $CDCl_3$): δ = 7.25 – 7.19 (m, 4H), 7.13 – 7.10 (m, 1H), 2.77 – 2.76 (m, 2H), 1.45 – 1.41 (m, 1H), 1.26 (s, 12H), 1.19 – 1.16 (m, 1H), 1.14 (s, 6H), 1.12 (s, 6H), 1.03 (d, J = 8 Hz, 3H). **^{13}C{^{1}H} NMR** (125 MHz, $CDCl_3$): δ = 143.2, 129.2, 128.0, 125.5, 83.0, 82.9, 36.0, 25.1, 25.0, 24.91, 24.88, 15.1. **^{11}B{^{1}H} NMR** (160 MHz, $CDCl_3$): δ = 34.3. **HRMS-ASAP** (m/z): Calculated (found) for $C_{22}H_{37}B_2O_4$ $[M+H]^+$ 387.2872 (387.2866). **Anal.** for $C_{22}H_{36}B_2O_4$ calcd: C, 68.43; H, 9.40. found: C, 68.56; H, 9.39.

2,2'-(1-Phenylethane-1,2-diyl)bis(4,4,5,5-tetramethyl-1,3,2-dioxaborolane) 2b

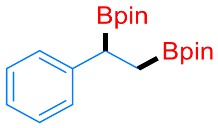

According to **General procedure 6** with (1-bromoethyl)benzene **2a** (55.5 mg, 0.3 mmol, 1.0 equiv.), the reaction mixture was purified by column chromatography on silica gel (hexane/EtOAc = 97/3) to yield the product **2b** as a colorless oil (98.8 mg, 276 µmol, 92% yield). **^{1}H NMR** (500 MHz, $CDCl_3$): δ = 7.23 – 7.22 (m, 4H), 7.13 – 7.07 (m, 1H), 2.52 (dd, *J* = 6, 11 Hz, 1H), 1.38 (dd, *J* = 11, 16 Hz, 1H), 1.20 (s, 12H), 1.19 (s, 6H), 1.17 (s, 6H), 1.11 (dd, *J* = 6, 16 Hz, 1H). **^{13}C{^{1}H} NMR** (125 MHz, $CDCl_3$): δ = 145.5, 128.3, 128.1, 125.0, 83.3, 83.2, 25.1, 24.84, 24.81, 24.6. **^{11}B{^{1}H} NMR** (160 MHz, $CDCl_3$): δ = 33.8. **HRMS-ASAP** (m/z): Calculated (found) for $C_{20}H_{33}B_2O_4$ $[M+H]^+$ 359.2559 (359.2556).

The spectroscopic data for **2b** match those reported in the literature.[19]

2,2'-(1-(*p*-Tolyl)ethane-1,2-diyl)bis(4,4,5,5-tetramethyl-1,3,2-dioxaborolane) 3b

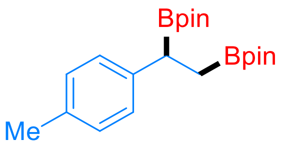

According to **General procedure 6** with 1-(1-bromoethyl)-4-methylbenzene **3a** (59.7 mg, 0.3 mmol, 1.0 equiv.), the reaction mixture was purified by column chromatography on silica gel (hexane/EtOAc = 97/3) to yield the product **3b** as a colorless oil (98.2 mg, 264 µmol, 88% yield). **^{1}H NMR** (400 MHz, $CDCl_3$): δ = 7.13 – 7.10 (m, 2H), 7.05 – 7.03 (m, 2H), 2.47 (dd, *J* = 6, 11 Hz, 1H), 2.28 (s, 3H), 1.36 (dd, *J* = 11, 16 Hz, 1H), 1.22 (s, 12H), 1.20 (s, 6H), 1.18 (s, 6H), 1.08 (dd, *J* = 6, 16 Hz, 1H). **^{13}C{^{1}H} NMR** (100 MHz, $CDCl_3$): δ = 142.4, 134.3, 129.0, 127.8, 83.2, 83.1, 25.1, 24.8, 24.7, 24.6, 21.1. **^{11}B{^{1}H} NMR** (128 MHz, $CDCl_3$): δ = 33.6. **HRMS-ASAP** (m/z): Calculated (found) for $C_{21}H_{35}B_2O_4$ $[M+H]^+$ 373.2716 (373.2712).

The spectroscopic data for **3b** match those reported in the literature.[19]

2,2'-(1-(4-Methoxyphenyl)ethane-1,2-diyl)bis(4,4,5,5-tetramethyl-1,3,2-dioxaborolane) 4b

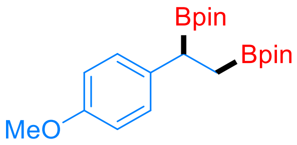

According to **General procedure 6** with 1-(1-bromoethyl)-4-methoxybenzene **4a** (64.5 mg, 0.3 mmol, 1.0 equiv.), the reaction mixture was purified by column chromatography on silica

gel (hexane/EtOAc = 97/3) to yield the product **4b** as a colorless oil (96.6 mg, 249 μmol, 83% yield). **^{1}H NMR** (400 MHz, $CDCl_3$): δ = 7.15 – 7.12 (m, 2H), 6.80 – 6.76 (m, 2H), 3.76 (s, 3H), 2.45 (dd, J = 6, 11 Hz, 1H), 1.33 (dd, J = 11, 16 Hz, 1H), 1.20 (s, 12H), 1.19 (s, 6H), 1.17 (s, 6H), 1.07 (dd, J = 6, 16 Hz, 1H). **^{13}C{^{1}H} NMR** (100 MHz, $CDCl_3$): δ = 157.2, 137.4, 128.8, 113.7, 83.3, 83.1, 55.3, 25.1, 24.82, 24.78, 24.6. **^{11}B{^{1}H} NMR** (128 MHz, $CDCl_3$): δ = 33.5. **HRMS-ASAP** (m/z): Calculated (found) for $C_{21}H_{35}B_2O_5$ $[M+H]^+$ 389.2665 (389.2661).

The spectroscopic data for **4b** match those reported in the literature.[20]

2,2'-(1-(4-(Methylthio)phenyl)ethane-1,2-diyl)bis(4,4,5,5-tetramethyl-1,3,2-dioxaborolane) 5b

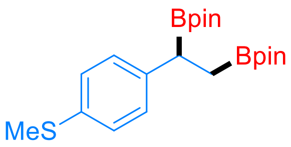

According to **General procedure 6** with (4-(1-bromoethyl)phenyl)(methyl)sulfane **5a** (69.3 mg, 0.3 mmol, 1.0 equiv.), the reaction mixture was purified by column chromatography on silica gel (hexane/EtOAc = 97/3) to yield the product **5b** as a colorless oil (84.9 mg, 210 μmol, 70% yield). **^{1}H NMR** (400 MHz, $CDCl_3$): δ = 7.15 (m, 3H), 2.47 (dd, J = 6, 11 Hz, 1H), 2.44 (s, 3H), 1.34 (dd, J = 11, 16 Hz, 1H), 1.20 (s, 12H), 1.18 (s, 6H), 1.17 (s, 6H), 1.07 (dd, J = 6, 16 Hz, 1H). **^{13}C{^{1}H} NMR** (100 MHz, $CDCl_3$): δ = 142.8, 134.0, 128.6, 127.2, 83.4, 83.2, 25.1, 24.82, 24.77, 24.6. **^{11}B{^{1}H} NMR** (128 MHz, $CDCl_3$): δ = 32.4. **HRMS-ASAP** (m/z): Calculated (found) for $C_{21}H_{35}B_2O_4S$ $[M+H]^+$ 405.2437 (405.2433). **Anal.** for $C_{21}H_{34}B_2O_4S$ calcd: C, 62.40; H, 8.48; S, 7.93. found: C, 62.29; H, 8.56; S, 7.98.

2,2'-(1-(4-Fluorophenyl)ethane-1,2-diyl)bis(4,4,5,5-tetramethyl-1,3,2-dioxaborolane) 6b

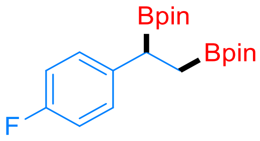

According to **General procedure 6** with 1-(1-bromoethyl)-4-fluorobenzene **6a** (60.9 mg, 0.3 mmol, 1.0 equiv.), the reaction mixture was purified by column chromatography on silica gel (hexane/EtOAc = 97/3) to yield the product **6b** as a colorless oil (82.4 mg, 219 μmol, 73% yield). **^{1}H NMR** (400 MHz, $CDCl_3$): δ = 7.17 – 7.13 (m, 2H), 6.93 – 6.87 (m, 2H), 2.49 (dd, J

= 6, 11 Hz, 1H), 1.32 (dd, J = 11, 16 Hz, 1H), 1.184 (s, 12H), 1.176 (s, 6H), 1.16 (s, 6H), 1.08 (dd, J = 6, 16 Hz, 1H). **^{13}C{^{1}H} NMR** (100 MHz, $CDCl_3$): δ = 160.9 (d, J = 240 Hz), 141.0 (d, J = 3 Hz), 129.2 (d, J = 8 Hz), 114.9 (d, J = 21 Hz), 83.4, 83.2, 25.1, 25.0, 24.8, 24.6. **^{19}F{^{1}H} NMR** (376 MHz, $CDCl_3$): δ = -119.3 (s). **^{11}B{^{1}H} NMR** (128 MHz, $CDCl_3$): δ = 33.3. **HRMS-ASAP** (m/z): Calculated (found) for $C_{20}H_{32}B_2FO_4$ $[M+H]^+$ 377.2465 (377.2459).

The spectroscopic data for **6b** match those reported in the literature.[19]

2,2'-(1-(4-Chlorophenyl)ethane-1,2-diyl)bis(4,4,5,5-tetramethyl-1,3,2-dioxaborolane) 7b

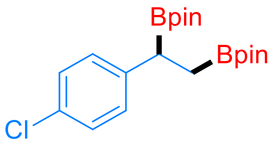

According to **General procedure 6** with 1-(1-bromoethyl)-4-chlorobenzene **7a** (65.9 mg, 0.3 mmol, 1.0 equiv.), the reaction mixture was purified by column chromatography on silica gel (hexane/EtOAc = 97/3) to yield the product **7b** as a colorless oil (90.7 mg, 231 µmol, 77% yield). **^{1}H NMR** (400 MHz, $CDCl_3$): δ = 7.21 – 7.17 (m, 2H), 7.16 – 7.13 (m, 2H), 2.49 (dd, J = 6, 11 Hz, 1H), 1.34 (dd, J = 11, 16 Hz, 1H), 1.20 (s, 6H), 1.19 (s, 6H), 1.18 (s, 6H), 1.17 (s, 6H), 1.07 (dd, J = 6, 16 Hz, 1H). **^{13}C{^{1}H} NMR** (100 MHz, $CDCl_3$): δ = 144.0, 130.6, 129.4, 128.3, 83.5, 83.3, 25.1, 24.80, 24.77, 24.6. **^{11}B{^{1}H} NMR** (128 MHz, $CDCl_3$): δ = 33.0. **HRMS-ASAP** (m/z): Calculated (found) for $C_{20}H_{32}B_2ClO_4$ $[M+H]^+$ 393.2170 (393.2168).

The spectroscopic data for **7b** match those reported in the literature.[21]

2,2'-(1-(4-Bromophenyl)ethane-1,2-diyl)bis(4,4,5,5-tetramethyl-1,3,2-dioxaborolane) 8b

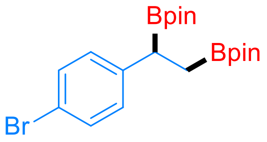

According to **General procedure 6** with 1-bromo-4-(1-bromoethyl)benzene **8a** (79.2 mg, 0.3 mmol, 1.0 equiv.), the reaction mixture was purified by column chromatography on silica gel (hexane/EtOAc = 97/3) to yield the product **8b** as a colorless oil (90.5 mg, 207 µmol, 69% yield). **^{1}H NMR** (400 MHz, $CDCl_3$): δ = 7.35 – 7.32 (m, 2H), 7.11 – 7.07 (m, 2H), 2.47 (dd, J = 6, 11 Hz, 1H), 1.33 (dd, J = 11, 16 Hz, 1H), 1.20 (s, 6H), 1.19 (s, 6H), 1.18 (s, 6H), 1.17 (s, 6H), 1.07 (dd, J = 6, 16 Hz, 1H). **^{13}C{^{1}H} NMR** (100 MHz, $CDCl_3$): δ = 144.5, 131.2, 129.8,

118.7, 83.5, 83.2, 25.1, 24.8, 24.7, 24.6. **^{11}B{^{1}H} NMR** (128 MHz, $CDCl_3$): δ = 32.7. **HRMS-ASAP** (m/z): Calculated (found) for $C_{20}H_{32}B_2BrO_4$ $[M+H]^+$ 437.1665 (437.1661).

The spectroscopic data for **8b** match those reported in the literature.[21]

2,2'-(1-(4-Iodophenyl)ethane-1,2-diyl)bis(4,4,5,5-tetramethyl-1,3,2-dioxaborolane) 9b

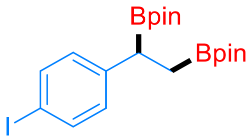

According to **General procedure 6** with 1-(1-bromoethyl)-4-iodobenzene **9a** (93.3 mg, 0.3 mmol, 1.0 equiv.), the reaction mixture was purified by column chromatography on silica gel (hexane/EtOAc = 97/3) to yield the product **9b** as a colorless oil (78.4 mg, 162 µmol, 54% yield). **^{1}H NMR** (400 MHz, $CDCl_3$): δ = 7.55 – 7.51 (m, 2H), 6.99 – 6.96 (m, 2H), 2.46 (dd, J = 6, 11 Hz, 1H), 1.33 (dd, J = 11, 16 Hz, 1H), 1.20 (s, 6H), 1.19 (s, 6H), 1.18 (s, 6H), 1.17 (s, 6H), 1.06 (dd, J = 6, 16 Hz, 1H). **^{13}C{^{1}H} NMR** (100 MHz, $CDCl_3$): δ = 145.3, 137.2, 130.2, 90.0, 83.5, 83.3, 25.1, 24.81, 24.78, 24.6. **^{11}B{^{1}H} NMR** (128 MHz, $CDCl_3$): δ = 33.3. **HRMS-ASAP** (m/z): Calculated (found) for $C_{20}H_{32}B_2IO_4$ $[M+H]^+$ 485.1526 (485.1521). **Anal.** for $C_{20}H_{31}B_2IO_4$ calcd: C, 49.63; H, 6.46. found: C, 49.71; H, 6.61.

2,2'-(1-(Naphthalen-2-yl)ethane-1,2-diyl)bis(4,4,5,5-tetramethyl-1,3,2-dioxaborolane) 10b

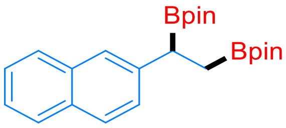

According to **General procedure 6** with 2-(1-bromoethyl)naphthalene **10a** (70.5 mg, 0.3 mmol, 1.0 equiv.), the reaction mixture was purified by column chromatography on silica gel (hexane/EtOAc = 97/3) to yield the product **10b** as a colorless oil (105.3 mg, 258 µmol, 86% yield). **^{1}H NMR** (400 MHz, $CDCl_3$): δ = 7.78 – 7.71 (m, 3H), 7.66 (s, 1H), 7.43 – 7.35 (m, 3H), 2.71 (dd, J = 6, 11 Hz, 1H), 1.50 (dd, J = 11, 16 Hz, 1H), 1.21 (dd, J = 6, 16 Hz, 1H), 1.21 (s, 12H), 1.20 (s, 6H), 1.18 (s, 6H). **^{13}C{^{1}H} NMR** (100 MHz, $CDCl_3$): δ = 143.1, 133.9, 131.8, 127.64, 127.61, 127.59, 127.5, 125.6, 125.5, 124.8, 83.4, 83.2, 25.1, 24.83, 24.77, 24.6. **^{11}B{^{1}H} NMR** (128 MHz, $CDCl_3$): δ = 33.0. **HRMS-ASAP** (m/z): Calculated (found) for $C_{24}H_{35}B_2O_4$ $[M+H]^+$ 409.2716 (409.2712).

The spectroscopic data for **10b** match those reported in the literature.[19]

2,2'-(1-Phenylbutane-1,2-diyl)bis(4,4,5,5-tetramethyl-1,3,2-dioxaborolane) 11b

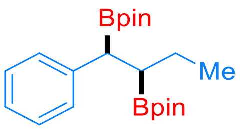

According to **General procedure 6** with (1-bromobutyl)benzene **11a** (63.9 mg, 0.3 mmol, 1.0 equiv.), the reaction mixture was purified by column chromatography on silica gel (hexane/EtOAc = 97/3) to yield the product **11b** as a colorless oil (95.0 mg, 246 µmol, 82% yield). **^{1}H NMR** (400 MHz, $CDCl_3$): δ = 7.24 – 7.16 (m, 4H), 7.12 – 7.08 (m, 1H), 2.40 (d, J = 13 Hz, 1H), 1.60 – 1.54 (m, 1H), 1.33 – 1.29 (m, 1H), 1.27 (s, 12H), 1.15 (s, 6H), 1.14 (s, 6H), 1.11 – 1.08 (m, 1H), 0.76 (t, J = 7 Hz, 3H). **^{13}C{^{1}H} NMR** (100 MHz, $CDCl_3$): δ = 142.9, 128.9, 128.2, 125.0, 83.2, 25.3, 25.1, 24.8, 24.3, 21.8, 12.5. **^{11}B{^{1}H} NMR** (128 MHz, $CDCl_3$): δ = 33.7. **HRMS-ASAP** (m/z): Calculated (found) for $C_{22}H_{37}B_2O_4$ $[M+H]^+$ 387.2872 (387.2866). **Anal.** for $C_{22}H_{36}B_2O_4$ calcd: C, 68.43; H, 9.40. found: C, 68.33; H, 9.52.

2,2'-(2,3-Dihydro-1*H*-indene-1,2-diyl)bis(4,4,5,5-tetramethyl-1,3,2-dioxaborolane) 12b

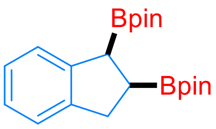

According to **General procedure 6** with 1-bromo-2,3-dihydro-1*H*-indene **12a** (59.1 mg, 0.3 mmol, 1.0 equiv.) or 2-chloro-2,3-dihydro-1*H*-indene **12a-1** (45.8 mg, 0.3 mmol, 1.0 equiv.), the reaction mixture was purified by column chromatography on silica gel (hexane/EtOAc = 97/3) to yield the product **12b** as a colorless oil (92.2 mg, 249 µmol, 83% yield from Br or 82.1 mg, 222 µmol, 74% yield from Cl). **^{1}H NMR** (400 MHz, $CDCl_3$): δ = 7.25 – 7.23 (m, 1H), 7.19 – 7.17 (m, 1H), 7.09 – 7.02 (m, 2H), 3.10 – 2.95 (m, 2H), 2.89 (d, J = 9 Hz, 1H), 2.02 – 1.95 (m, 1H), 1.27 (s, 12H), 1.17 (s, 6H), 1.09 (s, 6H). **^{13}C{^{1}H} NMR** (100 MHz, $CDCl_3$): δ = 146.2, 144.5, 125.8, 125.2, 124.1, 124.0, 83.3, 83.2, 35.0, 25.4, 25.0, 24.6, 24.3. **^{11}B{^{1}H} NMR** (160 MHz, $CDCl_3$): δ = 32.7. **HRMS-ASAP** (m/z): Calculated (found) for $C_{21}H_{33}B_2O_4$ $[M+H]^+$ 371.2559 (371.2556).

The spectroscopic data for **12b** match those reported in the literature.[22]

2,2'-(1-(4-Bromophenyl)propane-1,2-diyl)bis(4,4,5,5-tetramethyl-1,3,2-dioxaborolane) 13b

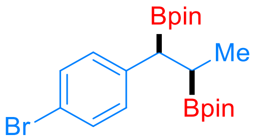

According to **General procedure 6** with 1-bromo-4-(1-bromopropyl)benzene **13a** (83.4 mg, 0.3 mmol, 1.0 equiv.), the reaction mixture was purified by column chromatography on silica gel (hexane/EtOAc = 97/3) to yield the product **13b** as a colorless oil (120.4 mg, 267 µmol, 89% yield). **^{1}H NMR** (300 MHz, $CDCl_3$): δ = 7.33 (d, J = 8 Hz, 2H), 7.04 (d, J = 8 Hz, 2H), 2.19 (d, J = 12 Hz, 1H), 1.48 (dd, J = 8, 12 Hz, 1H), 1.24 (s, 12H), 1.16 (s, 6H), 1.15 (s, 6H), 0.73 (d, J = 7 Hz, 3H). **^{13}C{^{1}H} NMR** (75 MHz, $CDCl_3$): δ = 142.2, 131.2, 130.8, 118.8, 83.3, 83.2, 25.1, 25.0, 24.7, 24.4, 14.7. **^{11}B{^{1}H} NMR** (96 MHz, $CDCl_3$): δ = 33.1. **HRMS-ASAP** (m/z): Calculated (found) for $C_{21}H_{34}B_2BrO_4$ $[M+H]^+$ 451.1821 (451.1815). **Anal.** for $C_{21}H_{33}B_2BrO_4$ calcd: C, 55.92; H, 7.38. found: C, 55.84; H, 7.52.

2,2'-(1-(4-Fluorophenyl)propane-1,2-diyl)bis(4,4,5,5-tetramethyl-1,3,2-dioxaborolane) 14b

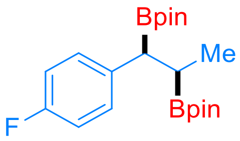

According to **General procedure 6** with 1-(1-bromopropyl)-4-fluorobenzene **14a** (65.1 mg, 0.3 mmol, 1.0 equiv.), the reaction mixture was purified by column chromatography on silica gel (hexane/EtOAc = 97/3) to yield the product **14b** as a colorless oil (98.3 mg, 252 µmol, 84% yield). **^{1}H NMR** (400 MHz, $CDCl_3$): δ = 7.12 – 7.09 (m, 2H), 6.93 – 6.88 (m, 2H), 2.20 (d, J = 12 Hz, 1H), 1.48 (dd, J = 8, 12 Hz, 1H), 1.24 (s, 12H), 1.16 (s, 6H), 1.15 (s, 6H), 0.73 (d, J = 7 Hz, 3H). **^{13}C{^{1}H} NMR** (100 MHz, $CDCl_3$): δ = 161.0 (d, J = 240 Hz), 138.5 (d, J = 3 Hz), 130.2 (d, J = 8 Hz), 114.9 (d, J = 21 Hz), 83.3, 83.2, 25.1, 25.0, 24.7, 24.4. **^{19}F{^{1}H} NMR** (376 MHz, $CDCl_3$): δ = -119.0 (s). **^{11}B{^{1}H} NMR** (128 MHz, $CDCl_3$): δ = 33.4. **HRMS-ASAP** (m/z): Calculated (found) for $C_{21}H_{34}B_2FO_4$ $[M+H]^+$ 391.2622 (391.2617). **Anal.** for $C_{21}H_{33}B_2FO_4$ calcd: C, 64.66; H, 8.53. found: C, 64.68; H, 8.41.

2,2'-(1-(3-Methoxyphenyl)propane-1,2-diyl)bis(4,4,5,5-tetramethyl-1,3,2-dioxaborolane) 15b

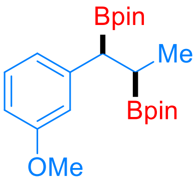

According to **General procedure 6** with 1-(1-bromopropyl)-3-methoxybenzene **15a** (68.7 mg, 0.3 mmol, 1.0 equiv.), the reaction mixture was purified by column chromatography on silica gel (hexane/EtOAc = 97/3) to yield the product **15b** as a colorless oil (90.5 mg, 225 μmol, 75% yield). **^{1}H NMR** (400 MHz, $CDCl_3$): δ = 7.14 (t, J = 8 Hz, 1H), 6.77 – 6.73 (m, 2H), 6.68 – 6.65 (m, 1H), 3.77 (s, 3H), 2.19 (d, J = 12 Hz, 1H), 1.52 (dd, J = 8, 12 Hz, 1H), 1.25 (s, 12H), 1.18 (s, 6H), 1.17 (s, 6H), 0.75 (d, J = 7 Hz, 3H). **^{13}C{^{1}H} NMR** (100 MHz, $CDCl_3$): δ = 159.6, 144.7, 129.1, 121.7, 114.3, 110.9, 83.3, 83.2, 55.2, 25.2, 25.1, 24.7, 24.4, 14.7. **^{11}B{^{1}H} NMR** (128 MHz, $CDCl_3$): δ = 33.0. **HRMS-ASAP** (m/z): Calculated (found) for $C_{22}H_{37}B_2O_5$ $[M+H]^+$ 403.2822 (403.2815). **Anal.** for $C_{22}H_{36}B_2O_5$ calcd: C, 65.71; H, 9.02. found: C, 65.84; H, 8.91.

2,2'-(1-(2-(Trifluoromethyl)phenyl)propane-1,2-diyl)bis(4,4,5,5-tetramethyl-1,3,2-dioxaborolane) 16b

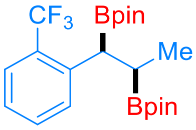

According to **General procedure 6** with 1-(1-bromopropyl)-2-(trifluoromethyl)benzene **16a** (80.1 mg, 0.3 mmol, 1.0 equiv.), the reaction mixture was purified by column chromatography on silica gel (hexane/EtOAc = 97/3) to yield the product **16b** as a colorless oil (97.7 mg, 222 μmol, 74% yield). **^{1}H NMR** (500 MHz, $CDCl_3$): δ = 7.57 (d, J = 8 Hz, 1H), 7.45 – 7.40 (m, 2H), 7.20 – 7.16 (m, 1H), 2.73 (d, J = 12 Hz, 1H), 1.63 – 1.56 (m, 1H), 1.26 (s, 12H), 1.16 (s, 6H), 1.14 (s, 6H), 0.71 (d, J = 8 Hz, 3H). **^{13}C{^{1}H} NMR** (125 MHz, $CDCl_3$): δ = 143.2, 131.5, 129.9, 129.6 (q, J = 29 Hz), 125.7 (q, J = 6 Hz), 124.78 (q, J = 272 Hz), 124.82, 83.3, 83.2, 25.1, 24.9, 24.7, 24.5, 14.6. **^{19}F{^{1}H} NMR** (470 MHz, $CDCl_3$): δ = -58.1 (s). **^{11}B{^{1}H} NMR** (160 MHz, $CDCl_3$): δ = 34.0. **HRMS-ASAP** (m/z): Calculated (found) for $C_{22}H_{34}B_2F_3O_4$ $[M+H]^+$ 441.1196 (441.1191). **Anal.** for $C_{22}H_{33}B_2F_3O_4$ calcd: C, 60.04; H, 7.56. found: C, 60.11; H, 7.43.

2,2'-(1-(2-Fluoro-5-(trifluoromethyl)phenyl)propane-1,2-diyl)bis(4,4,5,5-tetramethyl-1,3,2-dioxaborolane) 17b

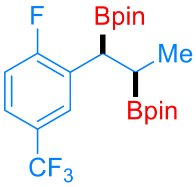

According to **General procedure 6** with 2-(1-bromopropyl)-1-fluoro-4-(trifluoromethyl)benzene **17a** (85.5 mg, 0.3 mmol, 1.0 equiv.), the reaction mixture was purified by column chromatography on silica gel (hexane/EtOAc = 97/3) to yield the product **17b** as a white solid (100.3 mg, 219 μmol, 73% yield). **^{1}H NMR** (500 MHz, $CDCl_3$): δ = 7.53 (dd, J = 7, 2 Hz, 1H), 7.39 – 7.36 (m, 1H), 7.06 (t, J = 9 Hz, 1H), 2.58 (d, J = 12 Hz, 1H), 1.55 – 1.48 (m, 1H), 1.26 (s, 6H), 1.25 (s, 6H), 1.21 (s, 6H), 1.20 (s, 6H), 0.78 (d, J = 8 Hz, 3H). **^{13}C{^{1}H} NMR** (125 MHz, $CDCl_3$): δ = 163.3 (dd, J = 2, 248 Hz), 131.6 (d, J = 18 Hz), 128.8 (q, J = 4 Hz), 126.3 (q, J = 32 Hz), 124.3 (q, J = 4 Hz), 124.2 (q, J = 270 Hz), 115.6 (d, J = 25 Hz), 25.1, 25.0, 24.7, 24.5, 14.9. **^{19}F{^{1}H} NMR** (470 MHz, $CDCl_3$): δ = -61.9 (q), -111.0 (s). **^{11}B{^{1}H} NMR** (160 MHz, $CDCl_3$): δ = 33.7. **HRMS-ASAP** (m/z): Calculated (found) for $C_{22}H_{33}B_2F_4O_4$ $[M+H]^+$ 459.2496 (459.2491). **Anal.** for $C_{22}H_{32}B_2F_4O_4$ calcd: C, 57.68; H, 7.04. found: C, 57.61; H, 7.22.

2,2'-(3-Methyl-1-phenylbutane-1,2-diyl)bis(4,4,5,5-tetramethyl-1,3,2-dioxaborolane) 18b

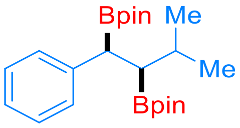

According to **General procedure 6** with (1-bromo-3-methylbutyl)benzene **18a** (68.1 mg, 0.3 mmol, 1.0 equiv.), the reaction mixture was purified by column chromatography on silica gel (hexane/EtOAc = 97/3) to yield the product **18b** as a colorless oil (100.8 mg, 252 μmol, 84% yield). **^{1}H NMR** (500 MHz, $CDCl_3$): δ = 7.25 – 7.15 (m, 4H), 7.12 – 7.06 (m, 1H), 2.52 (d, J = 13 Hz, 1H), 1.61 (dd, J = 3, 13 Hz, 1H), 1.44 – 1.38 (m, 1H), 1.28 (s, 12H), 1.14 (s, 6H), 1.13 (s, 6H), 0.92 (d, J = 7 Hz, 3H), 0.77 (d, J = 7 Hz, 3H). **^{13}C{^{1}H} NMR** (125 MHz, $CDCl_3$): δ = 143.2, 128.8, 128.3, 125.0, 83.2, 83.1, 26.8, 25.7, 25.2, 24.8, 24.2, 23.5, 18.6. **^{11}B{^{1}H} NMR** (160 MHz, $CDCl_3$): δ = 33.6. **HRMS-ASAP** (m/z): Calculated (found) for $C_{23}H_{39}B_2O_4$

[M+H]+ 401.3029 (401.3022). **Anal.** for $C_{23}H_{38}B_2O_4$ calcd: C, 69.03; H, 9.57. found: C, 69.14; H, 9.53.

1-(4-(1,2-Bis(4,4,5,5-tetramethyl-1,3,2-dioxaborolan-2-yl)propyl)phenyl)-1*H*-pyrazole 19b

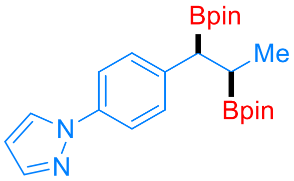

According to **General procedure 6** with 1-(4-(1-bromopropyl)phenyl)-1*H*-pyrazole **19a** (79.5 mg, 0.3 mmol, 1.0 equiv.), the reaction mixture was purified by column chromatography on silica gel (hexane/EtOAc = 90/10) to yield the product **19b** as a colorless oil (100.0 mg, 228 μmol, 76% yield). **^{1}H NMR** (500 MHz, $CDCl_3$): δ = 7.88 (d, J = 2 Hz, 1H), 7.70 (d, J = 2 Hz, 1H), 7.55 (d, J = 9 Hz, 2H), 7.26 (d, J = 9 Hz, 2H), 6.43 (t, J = 2 Hz, 1H), 2.28 (d, J = 12 Hz, 1H), 1.55 (dd, J = 8, 12 Hz, 1H), 1.26 (s, 12H), 1.18 (s, 6H), 1.16 (s, 6H), 0.78 (d, J = 8 Hz, 3H). **^{13}C{^{1}H} NMR** (125 MHz, $CDCl_3$): δ = 141.8, 140.6, 137.7, 129.9, 126.9, 119.4, 107.3, 83.4, 83.3, 25.2, 25.1, 24.7, 24.4, 14.7. **^{11}B{^{1}H} NMR** (160 MHz, $CDCl_3$): δ = 33.3. **HRMS-ASAP** (m/z): Calculated (found) for $C_{24}H_{37}B_2N_2O_4$ [M+H]+ 439.2934 (439.2931). **Anal.** for $C_{24}H_{36}$ $B_2N_2O_4$ calcd: C, 65.79; H, 8.28; N, 6.39. found: C, 65.88; H, 8.21; N, 6.33.

2,2'-(1-(4-(*tert*-Butyl)phenyl)-4-chlorobutane-1,2-diyl)bis(4,4,5,5-tetramethyl-1,3,2-dioxaborolane) 20b

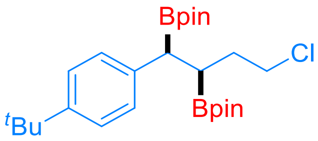

According to **General procedure 6** with 1-(*tert*-butyl)-4-(1,4-dichlorobutyl)benzene **20a** (77.8 mg, 0.3 mmol, 1.0 equiv.), the reaction mixture was purified by column chromatography on silica gel (hexane/EtOAc = 97/3) to yield the product **20b** as a colorless oil (118.7 mg, 249 μmol, 83% yield). **^{1}H NMR** (500 MHz, $CDCl_3$): δ = 7.23 (d, J = 8 Hz, 2H), 7.08 (d, J = 8 Hz, 2H), 3.53 – 3.48 (m, 1H), 3.38 – 3.32 (m, 1H), 2.33 – 2.30 (m, 1H), 1.73 – 1.64 (m, 2H), 1.35 – 1.31 (m, 1H), 1.28 (s, 9H), 1.26 (s, 12H), 1.16 (s, 6H), 1.15 (s, 6H). **^{13}C{^{1}H} NMR** (125 MHz, $CDCl_3$): δ = 148.1, 138.6, 128.5, 125.3, 83.5, 83.4, 44.6, 34.4, 32.9,

31.6, 25.3, 25.1, 24.8, 24.4. **^{11}B{^{1}H} NMR** (160 MHz, $CDCl_3$): δ = 33.6. **HRMS-ASAP** (m/z): Calculated (found) for $C_{26}H_{44}B_2ClO_4$ [M+H]$^+$ 477.3109 (477.3099). **Anal.** for $C_{26}H_{43}B_2ClO_4$ calcd: C, 65.51; H, 9.09. found: C, 65.43; H, 9.12.

2,2'-(Pentane-2,3-diyl)bis(4,4,5,5-tetramethyl-1,3,2-dioxaborolane) 21b

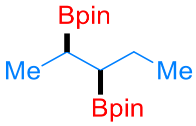

According to **General procedure 6** with 3-bromopentane **21a** (45.3 mg, 0.3 mmol, 1.0 equiv.), the reaction mixture was purified by column chromatography on silica gel (hexane/EtOAc = 98/2) to yield the product **21b** as a colorless oil (71.0 mg, 219 μmol, 73% yield). **^{1}H NMR** (500 MHz, $CDCl_3$): δ = 1.56 – 1.47 (m, 1H), 1.43 – 1.33 (m, 1H), 1.23 – 1.21 (m, 24H), 1.18 – 1.14 (m, 1H), 1.01 – 0.96 (m, 1H), 0.95 (d, J = 8 Hz, 3H), 0.87 (t, J = 8 Hz, 3H). **^{13}C{^{1}H} NMR** (125 MHz, $CDCl_3$): δ = 82.9, 82.8, 25.2, 25.1, 24.83, 24.76, 22.4, 14.5, 13.8. **^{11}B{^{1}H} NMR** (160 MHz, $CDCl_3$): δ = 34.6. **HRMS-ASAP** (m/z): Calculated (found) for $C_{17}H_{35}B_2O_4$ [M+H]$^+$ 325.2716 (325.2710). **Anal.** for $C_{17}H_{34}B_2O_4$ calcd: C, 63.01; H, 10.57. found: C, 62.94; H, 10.66.

2,2'-(1,3-Diphenylpropane-1,2-diyl)bis(4,4,5,5-tetramethyl-1,3,2-dioxaborolane) 22b

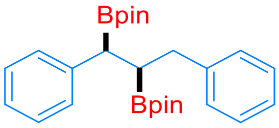

According to **General procedure 6** with 1,3-diphenylpropan-2-yl 4-methylbenzenesulfonate **22a** (109.9 mg, 0.3 mmol, 1.0 equiv.), the reaction mixture was purified by column chromatography on silica gel (hexane/EtOAc = 97/3) to yield the product **22b** as a colorless oil (100.8 mg, 225 μmol, 75% yield). **^{1}H NMR** (400 MHz, $CDCl_3$): δ = 7.28 – 7.24 (m, 4H), 7.19 – 7.07 (m, 6H), 2.64 (dd, J = 5, 14 Hz, 1H), 2.43 (t, J = 12 Hz, 2H), 2.02 – 1.93 (m, 1H), 1.16 (s, 12H), 1.15 (s, 6H), 1.10 (s, 6H). **^{13}C{^{1}H} NMR** (100 MHz, $CDCl_3$): δ = 142.6, 142.2, 129.3, 129.1, 128.3, 127.8, 125.5, 125.3, 83.3, 83.2, 35.8, 25.1, 25.0, 24.9, 24.5. **^{11}B{^{1}H} NMR** (128 MHz, $CDCl_3$): δ = 33.4. **HRMS-ASAP** (m/z): Calculated (found) for $C_{27}H_{39}B_2O_4$ [M+H]$^+$ 449.3029 (449.3021). **Anal.** for $C_{27}H_{38}B_2O_4$ calcd: C, 72.35; H, 8.55. found: C, 72.38; H, 8.67.

2,2'-(1-Cyclohexylethane-1,2-diyl)bis(4,4,5,5-tetramethyl-1,3,2-dioxaborolane) 23b

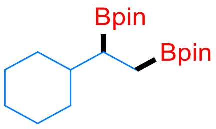

According to **General procedure 6** with 1-cyclohexylethyl 4-methylbenzenesulfonate **23a** (84.7 mg, 0.3 mmol, 1.0 equiv.), the reaction mixture was purified by column chromatography on silica gel (hexane/EtOAc = 97/3) to yield the product **23b** as a colorless oil (77.6 mg, 213 μmol, 71% yield). **^{1}H NMR** (400 MHz, $CDCl_3$): δ = 1.98 (d, J = 12 Hz, 1H), 1.90 (d, J = 12 Hz, 1H), 1.65 – 1.61 (m, 2H), 1.41 – 1.29 (m, 1H), 1.25 (s, 12H), 1.23 (s, 12H), 1.18 – 0.93 (m, 9H). **^{13}C{^{1}H} NMR** (100 MHz, $CDCl_3$): δ = 83.0, 82.8, 35.4, 34.1, 26.9, 25.9, 25.6, 25.3, 25.1, 24.9, 24.8, 11.9. **^{11}B{^{1}H} NMR** (128 MHz, $CDCl_3$): δ = 34.1. **HRMS-ASAP** (m/z): Calculated (found) for $C_{20}H_{39}B_2O_4$ $[M+H]^+$ 365.3029 (365.3026).

The spectroscopic data for **23b** match those reported in the literature.[22]

2,2'-(3,3-Dimethylbutane-1,2-diyl)bis(4,4,5,5-tetramethyl-1,3,2-dioxaborolane) 24b

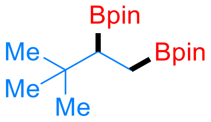

According to **General procedure 6** with 3-bromo-2,2-dimethylbutane **24a** (49.5 mg, 0.3 mmol, 1.0 equiv.), the reaction mixture was purified by column chromatography on silica gel (hexane/EtOAc = 98/2) to yield the product **24b** as a colorless oil (87.2 mg, 258 μmol, 86% yield). **^{1}H NMR** (500 MHz, $CDCl_3$): δ = 1.24 (s, 12H), 1.21 (s, 6H), 1.20 (s, 6H), 0.95 (dd, J = 4, 12 Hz, 1H), 0.90 (s, 9H), 0.84 – 0.77 (m, 2H). **^{13}C{^{1}H} NMR** (125 MHz, $CDCl_3$): δ = 82.9, 82.8, 32.5, 29.2, 25.3, 25.2, 25.0, 24.8. **^{11}B{^{1}H} NMR** (160 MHz, $CDCl_3$): δ = 34.3. **HRMS-ASAP** (m/z): Calculated (found) for $C_{18}H_{37}B_2O_4$ $[M+H]^+$ 339.2872 (339.2870).

The spectroscopic data for **24b** match those reported in the literature.[23]

1,2-Bis(4,4,5,5-tetramethyl-1,3,2-dioxaborolan-2-yl)cyclopentane 25b

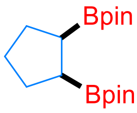

According to **General procedure 6** with bromocyclopentane **25a** (44.7 mg, 0.3 mmol, 1.0 equiv.) or chlorocyclopentane **25a-1** (31.4 mg, 0.3 mmol, 1.0 equiv.), the reaction mixture was purified by column chromatography on silica gel (hexane/EtOAc = 97/3) to yield the product **25b** as a colorless oil (89.9 mg, 279 µmol, 93% yield from Br) or (81.2 mg, 252 µmol, 84% yield from Cl). **^{1}H NMR** (300 MHz, $CDCl_3$): δ = 1.76 – 1.50 (m, 6H), 1.44 – 1.37 (m, 2H), 1.23 (s, 24H). **^{13}C{^{1}H} NMR** (75 MHz, $CDCl_3$): δ = 82.9, 28.8, 25.8, 25.0, 24.9. **^{11}B{^{1}H} NMR** (96 MHz, $CDCl_3$): δ = 34.0. **HRMS-ASAP** (m/z): Calculated (found) for $C_{17}H_{33}B_2O_4$ $[M+H]^+$ 323.2559 (323,2552).

The spectroscopic data for **25b** match those reported in the literature.[24]

1,2-Bis(4,4,5,5-tetramethyl-1,3,2-dioxaborolan-2-yl)cyclohexane 26b

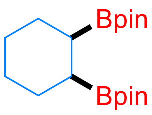

According to **General procedure 6** with bromocyclohexane **26a** (48.9 mg, 0.3 mmol, 1.0 equiv.), the reaction mixture was purified by column chromatography on silica gel (hexane/EtOAc = 97/3) to yield the product **26b** as a colorless oil (91.7 mg, 273 µmol, 91% yield). **^{1}H NMR** (500 MHz, $CDCl_3$): δ = 1.65 – 1.52 (m, 5H), 1.47 – 1.35 (m, 5H), 1.231 (s, 12H), 1.228 (s, 12H). **^{13}C{^{1}H} NMR** (125 MHz, $CDCl_3$): δ = 82.9, 28.2, 27.0, 25.1, 25.0. **^{11}B{^{1}H} NMR** (160 MHz, $CDCl_3$): δ = 34.3. **HRMS-ASAP** (m/z): Calculated (found) for $C_{18}H_{35}B_2O_4$ $[M+H]^+$ 337.2716 (337.2711).

The spectroscopic data for **26b** match those reported in the literature.[25]

1,2-Bis(4,4,5,5-tetramethyl-1,3,2-dioxaborolan-2-yl)cycloheptane 27b

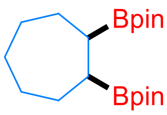

According to **General procedure 6** with bromocycloheptane **27a** (53.1 mg, 0.3 mmol, 1.0 equiv.), the reaction mixture was purified by column chromatography on silica gel (hexane/EtOAc = 97/3) to yield the product **27b** as a colorless oil (94.5 mg, 270 µmol, 90% yield). **^{1}H NMR** (500 MHz, $CDCl_3$): δ = 1.81 – 1.75 (m, 2H), 1.69 – 1.62 (m, 2H), 1.55 – 1.42 (m, 6H), 1.35 – 1.33 (m, 2H), 1.228 (s, 12H), 1.226 (s, 12H). **^{13}C{^{1}H} NMR** (125 MHz,

$CDCl_3$): δ = 82.9, 30.6, 28.5, 27.9, 25.00, 24.97. **$^{11}B\{^1H\}$ NMR** (160 MHz, $CDCl_3$): δ = 34.5. **HRMS-ASAP** (m/z): Calculated (found) for $C_{19}H_{37}B_2O_4$ $[M+H]^+$ 351.2872 (351.2866).

The spectroscopic data for **27b** match those reported in the literature.[25]

2,2'-(1,2,3,4-Tetrahydronaphthalene-1,2-diyl)bis(4,4,5,5-tetramethyl-1,3,2-dioxaborolane) 28b

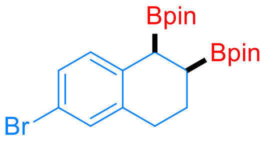

According to **General procedure 6** with 6-bromo-1,2,3,4-tetrahydronaphthalen-2-yl 4-methylbenzenesulfonate **28a** (114.4 mg, 0.3 mmol, 1.0 equiv.), the reaction mixture was purified by column chromatography on silica gel (hexane/EtOAc = 97/3) to yield the product **28b** as a colorless oil (84.7 mg, 183 µmol, 61% yield). **^1H NMR** (500 MHz, $CDCl_3$): δ = 7.16 – 7.12 (m, 2H), 7.00 (d, J = 8 Hz, 1H), 2.75 – 2.73 (m, 1H), 2.64 – 2.63 (m, 1H), 2.01 – 1.96 (m, 1H), 1.94 – 1.85 (m, 1H), 1.33 – 1.30 (m, 1H), 1.27 (s, 6H), 1.26 (s, 6H), 1.24 – 1.20 (m, 1H), 1.16 (s, 6H), 1.15 (s, 6H). **$^{13}C\{^1H\}$ NMR** (125 MHz, $CDCl_3$): δ = 139.0, 138.5, 132.0, 130.8, 128.0, 118.1, 83.4, 83.3, 30.0, 25.3, 25.0, 24.8, 24.7, 22.6. **$^{11}B\{^1H\}$ NMR** (160 MHz, $CDCl_3$): δ = 33.3. **HRMS-ASAP** (m/z): Calculated (found) for $C_{22}H_{34}B_2BrO_4$ $[M+H]^+$463.1821 (463.1814). **Anal.** for $C_{22}H_{33}B_2BrO_4$ calcd: C, 57.07; H, 7.18. found: C, 57.13; H, 7.26.

2,2'-(1-Phenylpropane-1,2-diyl)bis(4,4,5,5-tetramethyl-1,3,2-dioxaborolane) 29b

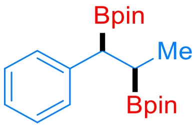

According to **General procedure 6** with 1-phenylpropan-2-yl 4-methylbenzenesulfonate **29a** (87.1 mg, 0.3 mmol, 1.0 equiv.), the reaction mixture was purified by column chromatography on silica gel (hexane/EtOAc = 97/3) to yield the product **29b** as a colorless oil (96.0 mg, 258 µmol, 86% yield). **^1H NMR** (400 MHz, $CDCl_3$): δ = 7.23 (t, J = 7 Hz, 2H), 7.16 (d, J = 7 Hz, 2H), 7.11 (t, J = 7 Hz, 1H), 2.22 (d, J = 12 Hz, 1H), 1.53 (dd, J = 8, 12 Hz, 1H), 1.26 (s, 12H), 1.17 (s, 6H), 1.15 (s, 6H), 0.74 (d, J = 7 Hz, 3H). **$^{13}C\{^1H\}$ NMR** (100 MHz, $CDCl_3$): δ = 143.0, 129.1, 128.2, 125.1, 83.3, 83.2, 25.2, 25.0, 24.7, 24.4, 14.7.

^{11}**B{^{1}H} NMR** (128 MHz, $CDCl_3$): δ = 33.8. **HRMS-ASAP** (m/z): Calculated (found) for $C_{21}H_{35}B_2O_4$ $[M+H]^+$ 373.2716 (373.2710).

The spectroscopic data for **29b** match those reported in the literature.[26]

2,2'-(1-(4-Fluorophenyl)propane-1,2-diyl)bis(4,4,5,5-tetramethyl-1,3,2-dioxaborolane) 30b

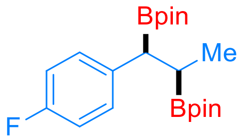

According to **General procedure 6** with 1-(4-fluorophenyl)propan-2-yl 4-methylbenzenesulfonate **30a** (92.5 mg, 0.3 mmol, 1.0 equiv.), the reaction mixture was purified by column chromatography on silica gel (hexane/EtOAc = 97/3) to yield the product **30b** as a colorless oil (98.3 mg, 252 μmol, 84% yield). ^{1}**H NMR** (400 MHz, $CDCl_3$): δ = 7.12 – 7.09 (m, 2H), 6.93 – 6.88 (m, 2H), 2.20 (d, J = 12 Hz, 1H), 1.48 (dd, J = 8, 12 Hz, 1H), 1.24 (s, 12H), 1.16 (s, 6H), 1.15 (s, 6H), 0.73 (d, J = 7 Hz, 3H). ^{13}**C{^{1}H} NMR** (100 MHz, $CDCl_3$): δ = 161.0 (d, J = 240 Hz), 138.5 (d, J = 3 Hz), 130.2 (d, J = 8 Hz), 114.9 (d, J = 21 Hz), 83.3, 83.2, 25.1, 25.0, 24.7, 24.4. ^{19}**F{^{1}H} NMR** (376 MHz, $CDCl_3$): δ = -119.0 (s). ^{11}**B{^{1}H} NMR** (128 MHz, $CDCl_3$): δ = 33.4. **HRMS-ASAP** (m/z): Calculated (found) for $C_{21}H_{34}B_2FO_4$ $[M+H]^+$ 391.2622 (391.2617). **Anal.** for $C_{21}H_{33}B_2FO_4$ calcd: C, 64.66; H, 8.53. found: C, 64.68; H, 8.41.

2,2'-(3-Methylbutane-1,2-diyl)bis(4,4,5,5-tetramethyl-1,3,2-dioxaborolane) 31b

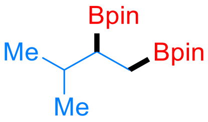

According to **General procedure 6** with 3-methylbutan-2-yl 4-methylbenzenesulfonate **31a** (72.7 mg, 0.3 mmol, 1.0 equiv.), the reaction mixture was purified by column chromatography on silica gel (hexane/EtOAc = 98/2) to yield the product **31b** as a colorless oil (71.9 mg, 222 μmol, 74% yield). ^{1}**H NMR** (400 MHz, $CDCl_3$): δ = 1.25 – 1.23 (m, 1H), 1.22 (s, 12H), 1.21 (s, 12H), 1.07 (dd, J = 7, 15 Hz, 1H), 0.94 (s, 3H), 0.93 (s, 3H), 0.93 – 0.91 (m, 2H). ^{13}**C{^{1}H} NMR** (100 MHz, $CDCl_3$): δ = 82.8, 82.8, 25.0, 24.94, 24.87, 24.8, 23.7, 23.4, 11.1. ^{11}**B{^{1}H} NMR** (128 MHz, $CDCl_3$): δ = 33.9. **HRMS-ASAP** (m/z): Calculated

(found) for $C_{17}H_{35}B_2O_4$ $[M+H]^+$ 325.2716 (325.2708). **Anal.** for $C_{17}H_{34}B_2O_4$ calcd: C, 63.01; H, 10.57. found: C, 62.92; H, 10.70.

2,2'-(5-(Benzo[*d*][1,3]dioxol-5-yl)-4-methylpentane-2,3-diyl)bis(4,4,5,5-tetramethyl-1,3,2-dioxaborolane) 32b

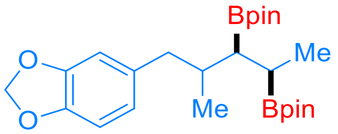

According to **General procedure 6** with 1-(benzo[*d*][1,3]dioxol-5-yl)-2-methylpentan-3-yl 4-methylbenzenesulfonate **32a** (112.9 mg, 0.3 mmol, 1.0 equiv.), the reaction mixture was purified by column chromatography on silica gel (hexane/EtOAc = 97/3) to yield the product **32b** as a colorless oil (88.0 mg, 192 µmol, 64% yield). **^{1}H NMR** (500 MHz, $CDCl_3$): δ = 6.82 (s, 1H), 6.70 (d, J = 8 Hz, 1H), 6.66 (d, J = 8 Hz, 1H), 5.88 (s, 2H), 2.90 (d, J = 14 Hz, 1H), 2.46 (d, J = 14 Hz, 1H), 1.63 – 1.58 (m, 1H), 1.50 – 1.43 (m, 1H), 1.28 (s, 6H), 1.27 (s, 6H), 1.22 (s, 6H), 1.18 (s, 6H), 0.93 (dd, J = 4, 12 Hz, 1H), 0.90 – 0.87 (m, 6H). **^{13}C{^{1}H} NMR** (125 MHz, $CDCl_3$): δ = 146.9, 145.4, 135.2, 123.5, 111.2, 107.5, 100.6, 83.2, 83.0, 42.8, 25.23, 25.15, 25.14, 25.11, 21.6, 20.2, 14.9. **^{11}B{^{1}H} NMR** (160 MHz, $CDCl_3$): δ = 34.3. **HRMS-ASAP** (m/z): Calculated (found) for $C_{25}H_{41}B_2O_6$ $[M+H]^+$ 459.3084 (459.3077). **Anal.** for $C_{25}H_{40}B_2O_6$ calcd: C, 65.53; H, 8.80. found: C, 65.67; H, 8.78.

2,2'-(1-(4-Methoxyphenyl)butane-2,3-diyl)bis(4,4,5,5-tetramethyl-1,3,2-dioxaborolane) 33b

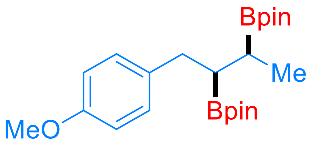

According to **General procedure 6** with 4-(4-methoxyphenyl)butan-2-yl 4-methylbenzenesulfonate **33a** (100.3 mg, 0.3 mmol, 1.0 equiv.), the reaction mixture was purified by column chromatography on silica gel (hexane/EtOAc = 97/3) to yield the product **33b** as a colorless oil (108.6 mg, 261 µmol, 87% yield). **^{1}H NMR** (400 MHz, $CDCl_3$): δ = 7.15 (d, J = 8 Hz, 2H), 6.77 (d, J = 8 Hz, 2H), 3.76 (s, 3H), 2.71 – 2.69 (m, 2H), 1.64 – 1.63 (m, 1H), 1.40 – 1.35 (m, 1H), 1.25 (s, 12H), 1.15 (s, 6H), 1.13 (s, 6H), 1.02 (d, J = 7 Hz, 3H). **^{13}C{^{1}H} NMR** (100 MHz, $CDCl_3$): δ = 157.6, 135.3, 130.1, 113.4, 83.0, 82.9, 55.4, 35.0, 25.1,

25.0, 24.9, 24.8, 15.1. **^{11}B{^{1}H} NMR** (128 MHz, $CDCl_3$): δ = 33.7. **HRMS-ASAP** (m/z): Calculated (found) for $C_{23}H_{39}B_2O_5$ $[M+H]^+$ 417.2978 (417.2972). **Anal.** for $C_{23}H_{38}B_2O_5$ calcd: C, 66.38; H, 9.20. found: C, 66.41; H, 9.29.

2,2'-(4-Methylpentane-2,3-diyl)bis(4,4,5,5-tetramethyl-1,3,2-dioxaborolane) 34b

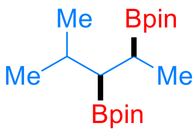

According to **General procedure 6** with 4-methylpentan-2-yl 4-methylbenzenesulfonate **34a** (76.9 mg, 0.3 mmol, 1.0 equiv.), the reaction mixture was purified by column chromatography on silica gel (hexane/EtOAc = 98/2) to yield the product **34b** as a colorless oil (82.2 mg, 243 μmol, 81% yield). **^{1}H NMR** (500 MHz, $CDCl_3$): δ = 1.96 – 1.90 (m, 1H), 1.27 (s, 6H), 1.26 (s, 6H), 1.25 (s, 6H), 1.24 (s, 6H), 1.23 – 1.21 (m, 1H), 0.97 (dd, J = 4, 8 Hz, 6H), 0.91 – 090 (m, 1H), 0.89 (d, J = 7 Hz, 3H). **^{13}C{^{1}H} NMR** (125 MHz, $CDCl_3$): δ = 82.83, 82.79, 26.8, 25.3, 25.2, 25.0, 24.7, 23.2, 21.2, 14.8. **^{11}B{^{1}H} NMR** (160 MHz, $CDCl_3$): δ = 34.3. **HRMS-ASAP** (m/z): Calculated (found) for $C_{18}H_{37}B_2O_4$ $[M+H]^+$ 339.2872 (339.2870). **Anal.** for $C_{18}H_{36}B_2O_4$ calcd: C, 63.94; H, 10.73. found: C, 63.87; H, 10.84.

2,2'-(Pentane-2,3-diyl)bis(4,4,5,5-tetramethyl-1,3,2-dioxaborolane) 35b

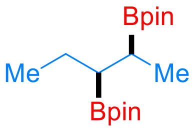

According to **General procedure 6** with 2-bromopentane **35a** (45.3 mg, 0.3 mmol, 1.0 equiv.), the reaction mixture was purified by column chromatography on silica gel (hexane/EtOAc = 98/2) to yield the product **34b** as a colorless oil (79.7 mg, 246 μmol, 82% yield). **^{1}H NMR** (500 MHz, $CDCl_3$): δ = 1.56 – 1.47 (m, 1H), 1.44 – 1.34 (m, 1H), 1.23 – 1.21 (m, 24H), 1.18 – 1.14 (m, 1H), 1.05 – 0.98 (m, 1H), 0.96 – 0.93 (m, 3H), 0.87 (t, J = 8 Hz, 3H). **^{13}C{^{1}H} NMR** (125 MHz, $CDCl_3$): δ = 82.9, 82.8, 25.2, 25.1, 24.83, 24.77, 22.4, 14.5, 13.8. **^{11}B{^{1}H} NMR** (160 MHz, $CDCl_3$): δ = 34.6. **HRMS-ASAP** (m/z): Calculated (found) for $C_{17}H_{35}B_2O_4$ $[M+H]^+$ 325.2716 (325.2712). **Anal.** for $C_{17}H_{34}B_2O_4$ calcd: C, 63.01; H, 10.57. found: C, 62.94; H, 10.66.

__**2,2'-(Hexane-2,3-diyl)bis(4,4,5,5-tetramethyl-1,3,2-dioxaborolane) 36b**__

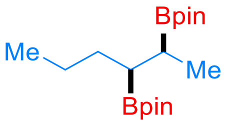

According to **General procedure 6** with 2-chlorohexane **36a** (36.2 mg, 0.3 mmol, 1.0 equiv.), the reaction mixture was purified by column chromatography on silica gel (hexane/EtOAc = 98/2) to yield the product **36b** as a colorless oil (86.2 mg, 255 μmol, 85% yield). **^{1}H NMR** (500 MHz, $CDCl_3$): δ = 1.51 – 1.44 (m, 1H), 1.37 – 1.28 (m, 2H), 1.27 – 1.24 (m, 1H), 1.22 – 1.21 (m, 24H), 1.18 – 1.13 (m, 1H), 1.10 – 1.04 (m, 1H), 0.87 (d, *J* = 8 Hz, 3H), 0.87 (t, *J* = 8 Hz, 3H). **^{13}C{^{1}H} NMR** (125 MHz, $CDCl_3$): δ = 82.8, 82.8, 31.9, 25.14, 25.10, 24.84, 24.76, 22.6, 14.7, 14.5. **^{11}B{^{1}H} NMR** (160 MHz, $CDCl_3$): δ = 34.6. **HRMS-ASAP** (m/z): Calculated (found) for $C_{18}H_{37}B_2O_4$ $[M+H]^+$ 339.2872 (339.2863). **Anal.** for $C_{18}H_{36}B_2O_4$ calcd: C, 63.94; H, 10.73. found: C, 63.83; H, 10.89.

__**2,2'-(5-Chloropentane-2,3-diyl)bis(4,4,5,5-tetramethyl-1,3,2-dioxaborolane) 37b**__

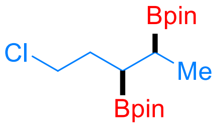

According to **General procedure 6** with 5-chloropentan-2-yl 4-methylbenzenesulfonate **37a** (36.2 mg, 0.3 mmol, 1.0 equiv.), the reaction mixture was purified by column chromatography on silica gel (hexane/EtOAc = 98/2) to yield the product **37b** as a colorless oil (85.0 mg, 237 μmol, 79% yield). **^{1}H NMR** (500 MHz, $CDCl_3$): δ = 3.65 – 3.60 (m, 1H), 3.55 – 3.50 (m, 1H), 2.05 – 1.96 (m, 1H), 1.84 – 1.74 (m, 1H), 1.30 – 1.25 (m, 1H), 1.23 – 1.22 (m, 24H), 1.20 – 1.17 (m, 1H), 0.99 (d, *J* = 7 Hz, 3H). **^{13}C{^{1}H} NMR** (125 MHz, $CDCl_3$): δ = 83.2, 83.1, 45.4, 33.2, 25.11, 25.07, 24.8, 24.8, 14.7. **^{11}B{^{1}H} NMR** (160 MHz, $CDCl_3$): δ = 34.3. **HRMS-ASAP** (m/z): Calculated (found) for $C_{17}H_{34}B_2ClO_4$ $[M+H]^+$ 359.2326 (359.2321). **Anal.** for $C_{17}H_{33}$ B_2ClO_4 calcd: C, 56.95; H, 9.28. found: C, 57.03; H, 9.14.

__**2,2'-(4-Phenylbutane-1,2-diyl)bis(4,4,5,5-tetramethyl-1,3,2-dioxaborolane) 39b**__

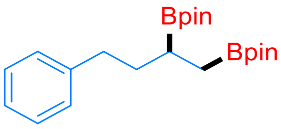

According to **General procedure 6** with (3,4-dibromobutyl)benzene **39a** (87.6 mg, 0.3 mmol, 1.0 equiv.), the reaction mixture was purified by column chromatography on silica gel (hexane/EtOAc = 97/3) to yield the product **39b** as a colorless oil (84.6 mg, 219 µmol, 73% yield). **^{1}H NMR** (500 MHz, $CDCl_3$): δ = 7.27 – 7.23 (m, 2H), 7.19 – 7.13 (m, 3H), 2.62 (t, J = 8 Hz, 2H), 1.82 – 1.75 (m, 1H), 1.67 – 1.60 (m, 1H), 1.25 (s, 12H), 1.23 (s, 12H), 1.21 – 1.18 (m, 1H), 0.97 – 0.85 (m, 2H). **^{13}C{^{1}H} NMR** (125 MHz, $CDCl_3$): δ = 143.5, 128.6, 128.3, 125.6, 83.0, 36.1, 35.5, 25.1, 25.0, 24.94, 24.92, 18.5, 12.7. **^{11}B{^{1}H} NMR** (160 MHz, $CDCl_3$): δ = 34.3. **HRMS-ASAP** (m/z): Calculated (found) for $C_{22}H_{37}B_2O_4$ $[M+H]^+$ 387.2872 (387.2865).

The spectroscopic data for **39b** match those reported in the literature.[27]

2,2'-(Octane-1,2-diyl)bis(4,4,5,5-tetramethyl-1,3,2-dioxaborolane) 40b

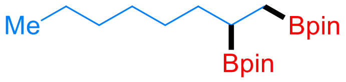

According to **General procedure 6** with octane-1,2-diyl bis(4-methylbenzenesulfonate) **40a** (136.4 mg, 0.3 mmol, 1.0 equiv.), the reaction mixture was purified by column chromatography on silica gel (hexane/EtOAc = 98/2) to yield the product **40b** as a colorless oil (96.7 mg, 264 µmol, 88% yield). **^{1}H NMR** (400 MHz, $CDCl_3$): δ = 1.46 – 1.37 (m, 1H), 1.32 – 1.24 (m, 8H), 1.22 (s, 12H), 1.21 (s, 12H), 1.13 – 1.06 (m, 2H), 0.89 – 0.79 (m, 5H). **^{13}C{^{1}H} NMR** (100 MHz, $CDCl_3$): δ = 82.93, 82.86, 34.0, 32.0, 29.7, 29.0, 25.03, 24.96, 24.89, 24.86, 22.8, 14.3. **^{11}B{^{1}H} NMR** (128 MHz, $CDCl_3$): δ = 33.4. **HRMS-ASAP** (m/z): Calculated (found) for $C_{20}H_{41}B_2O_4$ $[M+H]^+$ 367.3185 (367.3180).

The spectroscopic data for **40b** match those reported in the literature.[25]

2,2'-(2-Methylpropane-1,2-diyl)bis(4,4,5,5-tetramethyl-1,3,2-dioxaborolane) 41b

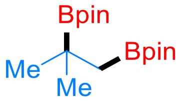

According to **General procedure 6** with 2-bromo-2-methylpropane **41a** (41.1 mg, 0.3 mmol, 1.0 equiv.), or 2-chloro-2-methylpropane **41a-1** (27.8 mg, 0.3 mmol, 1.0 equiv.), or di-*tert*-butyl dicarbonate **42a** (65.5 mg, 0.3 mmol, 1.0 equiv.), the reaction mixture was purified by column chromatography on silica gel (hexane/EtOAc = 97/3) to yield the product **41b** as a

colorless oil (from **41a**: 87.4 mg, 282 μmol, 94% yield; from **41a-1**: 82.8 mg, 267 μmol, 89% yield; from **42a**: 83.7 mg, 270 μmol, 90% yield). **^{1}H NMR** (400 MHz, $CDCl_3$): δ = 1.22 (d, J = 7 Hz, 24H), 0.97 (s, 6H), 0.81 (s, 2H). **^{13}C{^{1}H} NMR** (100 MHz, $CDCl_3$): δ = 83.0, 82.9, 27.6, 25.0, 24.8. **^{11}B{^{1}H} NMR** (128 MHz, $CDCl_3$): δ = 33.7. **HRMS-ASAP** (m/z): Calculated (found) for $C_{16}H_{33}B_2O_4$ $[M+H]^+$ 311.2559 (311.2551).

The spectroscopic data for **41b** match those reported in the literature.[25]

2,2'-(3-Ethylpentane-2,3-diyl)bis(4,4,5,5-tetramethyl-1,3,2-dioxaborolane) 43b

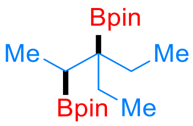

According to **General procedure 6** with 3-bromo-3-ethylpentane **43a** (53.7 mg, 0.3 mmol, 1.0 equiv.), the reaction mixture was purified by column chromatography on silica gel (hexane/EtOAc = 97/3) to yield the product **43b** as a colorless oil (92.9 mg, 264 μmol, 88% yield). **^{1}H NMR** (400 MHz, $CDCl_3$): δ = 1.59 – 1.32 (m, 4H), 1.23 (s, 12H), 1.223 (s, 6H), 1.219 (s, 6H), 1.13 (dd, J = 8, 16 Hz, 3H), 0.91 (d, J = 8 Hz, 3H), 0.82 – 0.75 (m, 6H). **^{13}C{^{1}H} NMR** (100 MHz, $CDCl_3$): δ = 82.8, 82.7, 26.3, 25.3, 25.1, 24.9, 24.8, 24.3, 11.6, 9.7, 8.8. **^{11}B{^{1}H} NMR** (128 MHz, $CDCl_3$): δ = 34.4. **HRMS-ASAP** (m/z): Calculated (found) for $C_{19}H_{39}B_2O_4$ $[M+H]^+$ 353.3029 (353.3026). **Anal.** for $C_{19}H_{38}B_2O_4$ calcd: C, 64.81; H, 10.88. found: C, 64.77; H, 10.97.

2,2'-(2-Methyl-1-phenylpropane-1,2-diyl)bis(4,4,5,5-tetramethyl-1,3,2-dioxaborolane) 44b

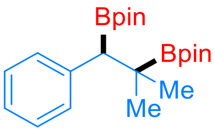

According to **General procedure 6** with (2-bromo-2-methylpropyl)benzene **44a** (63.9 mg, 0.3 mmol, 1.0 equiv.), the reaction mixture was purified by column chromatography on silica gel (hexane/EtOAc = 97/3) to yield the product **44b** as a colorless oil (75.3 mg, 195 μmol, 65% yield). **^{1}H NMR** (500 MHz, $CDCl_3$): δ = 7.23 – 7.18 (m, 4H), 7.15 – 7.12 (m, 1H), 2.47 (s, 1H), 1.24 (s, 6H), 1.23 (s, 6H), 1.22 (s, 12H), 0.98 (s, 3H), 0.83 (s, 3H). **^{13}C{^{1}H} NMR** (125 MHz, $CDCl_3$): δ = 140.5, 131.7, 127.4, 125.3, 83.3, 83.1, 29.9, 25.1, 25.0, 24.8, 24.7,

24.6, 22.9, 14.3. **^{11}B{^{1}H} NMR** (160 MHz, $CDCl_3$): δ = 34.0. **HRMS-ASAP** (m/z): Calculated (found) for $C_{22}H_{37}B_2O_4$ $[M+H]^+$ 387.2872 (387.2866). **Anal.** for $C_{22}H_{36}B_2O_4$ calcd: C, 68.43; H, 9.40. found: C, 68.35; H, 9.42.

2,2'-(2-Phenylpropane-1,2-diyl)bis(4,4,5,5-tetramethyl-1,3,2-dioxaborolane) 45b

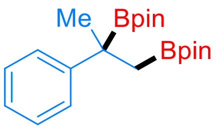

According to **General procedure 6** with 2-phenylpropan-2-ol **45a** (40.8 mg, 0.3 mmol, 1.0 equiv.), the reaction mixture was purified by column chromatography on silica gel (hexane/EtOAc = 97/3) to yield the product **45b** as a colorless oil (97.1 mg, 261 µmol, 87% yield). **^{1}H NMR** (500 MHz, $CDCl_3$): δ = 7.40 – 7.37 (m, 2H), 7.27 – 7.23 (m, 2H), 7.12 – 7.08 (m, 1H), 1.48 (d, J = 16 Hz, 1H), 1.41 (s, 3H), 1.211 (s, 6H), 1.207 (s, 6H), 1.20 (s, 6H), 1.18 (s, 6H), 1.14 (d, J = 16 Hz, 1H). **^{13}C{^{1}H} NMR** (125 MHz, $CDCl_3$): δ = 149.1, 127.9, 126.4, 124.8, 83.3, 83.0, 25.1, 24.70, 24.68, 24.6, 24.4. **^{11}B{^{1}H} NMR** (160 MHz, $CDCl_3$): δ = 33.9. **HRMS-ASAP** (m/z): Calculated (found) for $C_{21}H_{35}B_2O_4$ $[M+H]^+$ 373.2716 (373.2711).

The spectroscopic data for **45b** match those reported in the literature.[28]

2,2'-(2-(*p*-Tolyl)propane-1,2-diyl)bis(4,4,5,5-tetramethyl-1,3,2-dioxaborolane) 46b

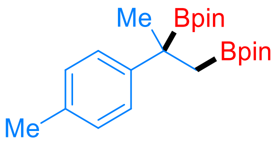

According to **General procedure 6** with 2-(*p*-tolyl)propan-2-ol **46a** (45.1 mg, 0.3 mmol, 1.0 equiv.), the reaction mixture was purified by column chromatography on silica gel (hexane/EtOAc = 97/3) to yield the product **46b** as a colorless oil (97.3 mg, 252 µmol, 84% yield). **^{1}H NMR** (500 MHz, $CDCl_3$): δ = 7.27 (d, J = 8 Hz, 2H), 7.06 (d, J = 8 Hz, 2H), 2.29 (s, 3H), 1.47 (d, J = 16 Hz, 1H), 1.38 (s, 3H), 1.22 (s, 6H), 1.21 (s, 6H), 1.20 (s, 6H), 1.18 (s, 6H), 1.10 (d, J = 16 Hz, 1H). **^{13}C{^{1}H} NMR** (125 MHz, $CDCl_3$): δ = 146.3, 134.2, 128.8, 126.4, 83.3, 83.1, 25.3, 25.0, 24.9, 24.7, 24.6, 21.0. **^{11}B{^{1}H} NMR** (160 MHz, $CDCl_3$): δ = 34.0. **HRMS-ASAP** (m/z): Calculated (found) for $C_{22}H_{37}B_2O_4$ $[M+H]^+$ 387.2872 (387.2866).

The spectroscopic data for **46b** match those reported in the literature.[28]

2,2'-(2-(*m*-Tolyl)propane-1,2-diyl)bis(4,4,5,5-tetramethyl-1,3,2-dioxaborolane) 47b

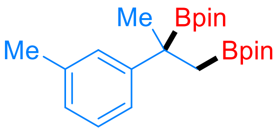

According to **General procedure 6** with 2-(*m*-tolyl)propan-2-ol **47a** (45.1 mg, 0.3 mmol, 1.0 equiv.), the reaction mixture was purified by column chromatography on silica gel (hexane/EtOAc = 97/3) to yield the product **47b** as a colorless oil (93.8 mg, 243 µmol, 81% yield). **^{1}H NMR** (500 MHz, $CDCl_3$): δ = 7.21 – 7.17 (m, 2H), 7.14 (t, J = 8 Hz, 1H), 6.93 – 6.91 (m, 1H), 2.31 (s, 3H), 1.47 (d, J = 16 Hz, 1H), 1.39 (s, 3H), 1.221 (s, 6H), 1.216 (s, 6H), 1.21 (s, 6H), 1.19 (s, 6H), 1.13 (d, J = 16 Hz, 1H). **^{13}C{^{1}H} NMR** (125 MHz, $CDCl_3$): δ = 149.2, 137.3, 127.9, 127.3, 125.7, 123.7, 83.4, 83.1, 25.2, 24.84, 24.83, 24.7, 24.6, 21.8. **^{11}B{^{1}H} NMR** (160 MHz, $CDCl_3$): δ = 33.9. **HRMS-ASAP** (m/z): Calculated (found) for $C_{22}H_{37}B_2O_4$ $[M+H]^+$ 387.2872 (387.2866).

The spectroscopic data for **47b** match those reported in the literature.[28]

2,2'-(2-(4-Fluorophenyl)propane-1,2-diyl)bis(4,4,5,5-tetramethyl-1,3,2-dioxaborolane) 48b

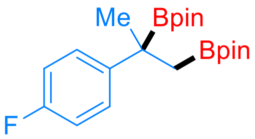

According to **General procedure 6** with 2-(4-fluorophenyl)propan-2-ol **48a** (46.3 mg, 0.3 mmol, 1.0 equiv.), the reaction mixture was purified by column chromatography on silica gel (hexane/EtOAc = 97/3) to yield the product **48b** as a colorless oil (97.1 mg, 249 µmol, 83% yield). **^{1}H NMR** (500 MHz, $CDCl_3$): δ = 7.35 – 7.31 (m, 2H), 6.95 – 6.91 (m, 2H), 1.42 (d, J = 16 Hz, 1H), 1.38 (s, 3H), 1.20 (s, 6H), 1.19 (s, 12H), 1.18 (s, 6H), 1.13 (d, J = 16 Hz, 1H). **^{13}C{^{1}H} NMR** (125 MHz, $CDCl_3$): δ = 160.8 (d, J = 240 Hz), 144.9 (d, J = 3 Hz), 128.0 (d, J = 8 Hz), 114.6 (d, J = 20 Hz), 83.5, 83.2, 25.2, 25.0, 24.8, 24.7, 24.6. **^{11}B{^{1}H} NMR** (160 MHz, $CDCl_3$): δ = 33.8. **^{19}F{^{1}H} NMR** (470 MHz, $CDCl_3$): δ = -119.6 (m). **HRMS-ASAP** (m/z): Calculated (found) for $C_{21}H_{34}B_2FO_4$ $[M+H]^+$ 391.2622 (391.2615). **Anal.** for $C_{21}H_{33}B_2FO_4$ calcd: C, 64.66; H, 8.53. found: C, 64.53; H, 8.66.

The spectroscopic data for **48b** match those reported in the literature.[28]

2,2'-(2-(4-Chlorophenyl)propane-1,2-diyl)bis(4,4,5,5-tetramethyl-1,3,2-dioxaborolane) 49b

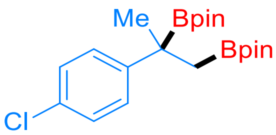

According to **General procedure 6** with 2-(4-chlorophenyl)propan-2-ol **49a** (51.2 mg, 0.3 mmol, 1.0 equiv.), the reaction mixture was purified by column chromatography on silica gel (hexane/EtOAc = 97/3) to yield the product **49b** as a colorless oil (95.1 mg, 234 µmol, 78% yield). **^{1}H NMR** (500 MHz, $CDCl_3$): δ = 7.31 (d, J = 9 Hz, 2H), 7.21 (d, J = 9 Hz, 2H), 1.42 (d, J = 16 Hz, 1H), 1.37 (s, 3H), 1.20 (s, 6H), 1.195 (s, 6H), 1.19 (s, 6H), 1.18 (s, 6H), 1.12 (d, J = 16 Hz, 1H). **^{13}C{^{1}H} NMR** (125 MHz, $CDCl_3$): δ = 147.9, 130.7, 128.1, 128.0, 83.5, 83.2, 25.2, 24.82, 24.78, 24.7, 24.6. **^{11}B{^{1}H} NMR** (160 MHz, $CDCl_3$): δ = 33.7. **HRMS-ASAP** (m/z): Calculated (found) for $C_{21}H_{34}B_2ClO_4$ $[M+H]^+$ 407.2326 (407.2322). **Anal.** for $C_{21}H_{33}B_2ClO_4$ calcd: C, 62.04; H, 8.18. found: C, 62.68; H, 8.19.

The spectroscopic data for **49b** match those reported in the literature.[28]

2,2'-(2-(4-Methoxyphenyl)propane-1,2-diyl)bis(4,4,5,5-tetramethyl-1,3,2-dioxaborolane) 50b

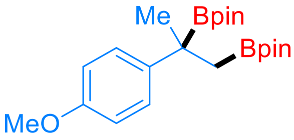

According to **General procedure 6** with 2-(4-methoxyphenyl)propan-2-ol **50a** (49.9 mg, 0.3 mmol, 1.0 equiv.), the reaction mixture was purified by column chromatography on silica gel (hexane/EtOAc = 97/3) to yield the product **50b** as a colorless oil (67.6 mg, 168 µmol, 56% yield). **^{1}H NMR** (500 MHz, $CDCl_3$): δ = 7.30 (d, J = 9 Hz, 2H), 6.81 (d, J = 9 Hz, 2H), 3.77 (s, 3H), 1.45 (d, J = 16 Hz, 1H), 1.37 (s, 3H), 1.210 (s, 6H), 1.206 (s, 6H), 1.19 (s, 6H), 1.18 (s, 6H), 1.10 (d, J = 16 Hz, 1H). **^{13}C{^{1}H} NMR** (125 MHz, $CDCl_3$): δ = 157.1, 141.4, 127.5, 113.4, 83.3, 83.1, 55.3, 25.2, 25.0, 24.8, 24.7, 24.6. **^{11}B{^{1}H} NMR** (160 MHz, $CDCl_3$): δ = 33.9. **HRMS-ASAP** (m/z): Calculated (found) for $C_{22}H_{37}B_2O_5$ $[M+H]^+$ 403.2822 (403.2813). **Anal.** for $C_{22}H_{36}B_2O_5$ calcd: C, 65.71; H, 9.02. found: C, 65.58; H, 9.14.

The spectroscopic data for **50b** match those reported in the literature.[28]

2,2'-(1,1-Diphenylethane-1,2-diyl)bis(4,4,5,5-tetramethyl-1,3,2-dioxaborolane) 51b

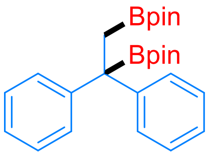

According to **General procedure 6** with 1,1-diphenylethanol **51a** (59.5 mg, 0.3 mmol, 1.0 equiv.), the reaction mixture was purified by column chromatography on silica gel (hexane/EtOAc = 97/3) to yield the product **51b** as a colorless oil (82.1 mg, 189 μmol, 63% yield). **^{1}H NMR** (500 MHz, $CDCl_3$): δ = 7.35 – 7.33 (m, 4H), 7.21 – 7.17 (m, 4H), 7.11 – 7.07 (m, 2H), 1.73 (s, 2H), 1.16 (s, 12H), 1.05 (s, 12H). **^{13}C{^{1}H} NMR** (125 MHz, $CDCl_3$): δ = 148.3, 129.4, 127.7, 125.2, 83.6, 83.1, 24.9, 24.5. **^{11}B{^{1}H} NMR** (160 MHz, $CDCl_3$): δ = 33.3. **HRMS-ASAP** (m/z): Calculated (found) for $C_{26}H_{37}B_2O_4$ $[M+H]^+$ 435.2872 (435.2865). **Anal.** for $C_{26}H_{36}B_2O_4$ calcd: C, 71.92; H, 8.36. found: C, 71.75; H, 8.47.

2,2'-(1-Phenylcyclopentane-1,2-diyl)bis(4,4,5,5-tetramethyl-1,3,2-dioxaborolane) 52b

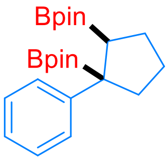

According to **General procedure 6** with 1-phenylcyclopentanol **52a** (48.7 mg, 0.3 mmol, 1.0 equiv.), the reaction mixture was purified by column chromatography on silica gel (hexane/EtOAc = 97/3) to yield the product **52b** as a colorless oil (98.0 mg, 246 μmol, 82% yield). **^{1}H NMR** (500 MHz, $CDCl_3$): δ = 7.41 – 7.38 (m, 2H), 7.25 – 7.21 (m, 2H), 7.11 – 7.07 (m, 1H), 2.25 – 2.20 (m, 1H), 198 – 1.93 (m, 1H), 1.84 – 1.73 (m, 2H), 1.70 – 1.60 (m, 2H), 1.26 (s, 6H), 1.25 (s, 6H), 1.17 (s, 6H), 1.14 (s, 6H), 0.89 (t, J = 7 Hz, 1H). **^{13}C{^{1}H} NMR** (125 MHz, $CDCl_3$): δ = 147.5, 127.8, 127.4, 124.7, 83.3, 83.0, 36.5, 27.7, 25.2, 24.8, 24.7, 24.5, 24.0. **^{11}B{^{1}H} NMR** (160 MHz, $CDCl_3$): δ = 34.2. **HRMS-ASAP** (m/z): Calculated (found) for $C_{23}H_{37}B_2O_4$ $[M+H]^+$ 399.2872 (399.2865). **Anal.** for $C_{26}H_{36}B_2O_4$ calcd: C, 69.38; H, 9.11. found: C, 69.31; H, 9.24.

2,2'-(3-Methyl-1-phenylbutane-2,3-diyl)bis(4,4,5,5-tetramethyl-1,3,2-dioxaborolane) 53b

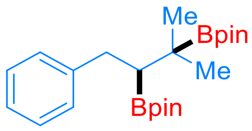

According to **General procedure 6** with 2-methyl-4-phenylbutan-2-ol **53a** (49.3 mg, 0.3 mmol, 1.0 equiv.), the reaction mixture was purified by column chromatography on silica gel (hexane/EtOAc = 97/3) to yield the product **53b** as a colorless oil (80.4 mg, 201 μmol, 67% yield). **^{1}H NMR** (500 MHz, $CDCl_3$): δ = 7.25 – 7.18 (m, 4H), 7.12 – 7.08 (m, 1H), 2.72 – 2.70 (m, 1H), 1.50 (dd, J = 6, 11 Hz, 1H), 1.25 (s, 12H), 1.08 (s, 6H), 1.06 (s, 3H), 1.04 (s, 3H), 1.01 (s, 6H), 0.88 (t, J = 7 Hz, 1H). **^{13}C{^{1}H} NMR** (125 MHz, $CDCl_3$): δ = 143.4, 129.3, 128.0, 125.5, 83.1, 82.9, 35.1, 25.5, 25.2, 25.1, 24.9, 24.8, 23.1, 22.5, 14.2. **^{11}B{^{1}H} NMR** (160 MHz, $CDCl_3$): δ = 34.6. **HRMS-ASAP** (m/z): Calculated (found) for $C_{23}H_{39}B_2O_4$ $[M+H]^+$ 401.3029 (401.3022). **Anal.** for $C_{23}H_{38}B_2O_4$ calcd: C, 69.03; H, 9.57. found: C, 68.95; H, 9.55.

2,2'-(1-(3,4-Dimethoxyphenyl)propane-1,2-diyl)bis(4,4,5,5-tetramethyl-1,3,2-dioxaborolane) 55b

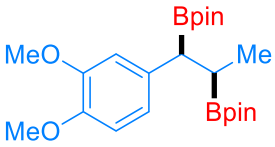

According to **General procedure 6** with 1-(3,4-dimethoxyphenyl)propan-2-yl 4-methylbenzenesulfonate **55a** (105.1 mg, 0.3 mmol, 1.0 equiv.), the reaction mixture was purified by column chromatography on silica gel (hexane/EtOAc = 97/3) to yield the product **55b** as a colorless oil (97.2 mg, 225 μmol, 75% yield). **^{1}H NMR** (300 MHz, $CDCl_3$): δ = 6.74 – 6.68 (m, 3H), 3.84 (s, 3H), 3.83 (s, 3H), 2.13 (d, J = 12 Hz, 1H), 1.47 (dq, J = 7, 12 Hz, 1H), 1.25 (s, 12H), 1.18 (s, 6H), 1.17 (s, 6H), 0.76 (d, J = 7 Hz, 3H). **^{13}C{^{1}H} NMR** (75 MHz, $CDCl_3$): δ = 148.7, 146.7, 135.5, 121.2, 111.9, 111.1, 83.23, 83.16, 55.9, 55.8, 25.2, 24.7, 24.4, 14.7. **^{11}B{^{1}H} NMR** (96 MHz, $CDCl_3$): δ = 33.0. **HRMS-ASAP** (m/z): Calculated (found) for $C_{23}H_{38}B_2O_6$ $[M]^+$ 432.2849 (432.2843). **Anal.** for $C_{23}H_{38}B_2O_4$ calcd: C, 63.92; H, 8.86. found: C, 64.04; H, 8.95.

(*R*)-Methyl 4-((2*R*,3*S*,5*R*,8*R*,9*S*,10*S*,13*R*,14*S*,17*R*)-10,13-dimethyl-2,3-bis(4,4,5,5-tetramethyl-1,3,2-dioxaborolan-2-yl)hexadecahydro-1*H*-cyclopenta[*a*]phenanthren-17-yl)pentanoate 56b

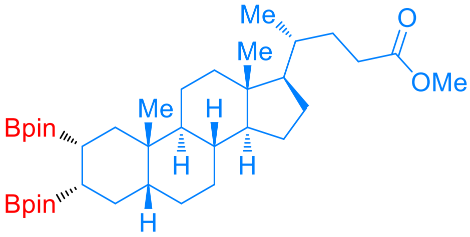

According to **General procedure 6** with (*R*)-methyl 4-((3*R*,5*R*,8*R*,9*S*,10*S*,13*R*,14*S*,17*R*)-10,13-dimethyl-3-(tosyloxy)hexadecahydro-1*H*-cyclopenta[*a*]phenanthren-17-yl)pentanoate **56a** (163.4 mg, 0.3 mmol, 1.0 equiv.), the reaction mixture was purified by column chromatography on silica gel (hexane/EtOAc = 90/10) to yield the product **56b** as a white solid (122.2 mg, 195 µmol, 65% yield). **^{1}H NMR** (500 MHz, $CDCl_3$): δ = 3.65 (s, 3H), 2.37 – 2.31 (m, 1H), 2.24 – 2.17 (m, 1H), 1.97 – 1.91 (m, 2H), 1.86 – 1.74 (m, 4H), 1.66 – 0.84 (m, 50H), 0.62 (s, 3H). **^{13}C{^{1}H} NMR** (125 MHz, $CDCl_3$): δ = 175.0, 83.1, 82.7, 56.8, 56.2, 51.6, 42.9, 42.7, 40.5, 40.2, 37.0, 36.1, 35.5, 35.4, 31.21, 31.18, 29.5, 28.4, 27.9, 26.7, 25.2, 25.0, 24.7, 24.6, 24.4, 20.9, 18.4, 12.2. **^{11}B{^{1}H} NMR** (160 MHz, $CDCl_3$): δ = 34.0. **HRMS-ASAP** (m/z): Calculated (found) for $C_{37}H_{65}B_2O_6$ $[M+H]^+$ 627.4962 (627.4951). **Anal.** for $C_{37}H_{64}B_2O_6$ calcd: C, 70.93; H, 10.30. found: C, 70.87; H, 10.43.

2,2'-((2*R*,3*S*,8*R*,9*S*,10*S*,13*S*,14*S*)-10,13-Dimethylhexadecahydrospiro[cyclopenta[*a*]phenanthrene-17,2'-[1,3]dioxolane]-2,3-diyl)bis(4,4,5,5-tetramethyl-1,3,2-dioxaborolane) 57b

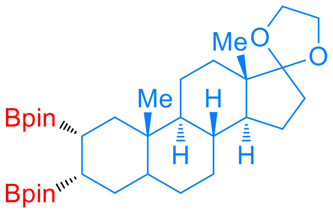

According to **General procedure 6** with (3*S*,8*R*,9*S*,10*S*,13*S*,14*S*)-10,13-dimethylhexadecahydrospiro[cyclopenta[*a*]phenanthrene-17,2'-[1,3]dioxolan]-3-yl 4-methylbenzenesulfonate **57a** (146.6 mg, 0.3 mmol, 1.0 equiv.), the reaction mixture was purified by column chromatography on silica gel (hexane/EtOAc = 90/10) to yield the product **57b** as a white solid (124.9 mg, 219 µmol, 73% yield). **^{1}H NMR** (500 MHz, $CDCl_3$): δ = 4.49 (dd, *J* = 4, 11 Hz, 1H), 4.24 – 4.20 (m, 1H), 4.14 – 4.09 (m, 1H), 3.95 – 3.89 (m, 1H), 2.10 –

2.02 (m, 1H), 1.98 – 1.93 (m, 1H), 1.74 – 1.65 (m, 4H), 1.57 – 1.52 (m, 2H), 1.49 – 1.46 (m, 1H), 1.43 – 1.40 (m, 2H), 1.35 – 1.06 (m, 30H), 0.98 – 0.83 (m, 7H), 0.74 (S, 3H), 0.69 – 0.64 (m, 1H). **^{13}C{^{1}H} NMR** (125 MHz, $CDCl_3$): δ = 117.1, 83.1, 82.7, 66.6, 66.2, 56.0, 54.8, 48.9, 46.4, 45.8, 38.4, 36.4, 35.63, 35.6, 31.5, 31.3, 30.8, 28.9, 25.14, 25.08, 24.9, 24.8, 20.1, 14.9, 12.3. **^{11}B{^{1}H} NMR** (160 MHz, $CDCl_3$): δ = 34.2. **HRMS-ASAP** (m/z): Calculated (found) for $C_{33}H_{57}B_2O_6$ $[M+H]^+$ 571.4336 (571.4331). **Anal.** for $C_{33}H_{56}B_2O_6$ calcd: C, 69.48; H, 9.90. found: C, 69.70; H, 9.86.

2,2'-((2a*S*,2'*R*,4*S*,5*R*,5'*R*,6a*S*,6b*S*,8a*S*,8b*R*,11a*S*,12a*S*,12b*R*)-5',6a,8a-Trimethyldocosahydrospiro[naphtho[2',1':4,5]indeno[2,1-*b*]furan-10,2'-pyran]-4,5-diyl)bis(4,4,5,5-tetramethyl-1,3,2-dioxaborolane) 58b

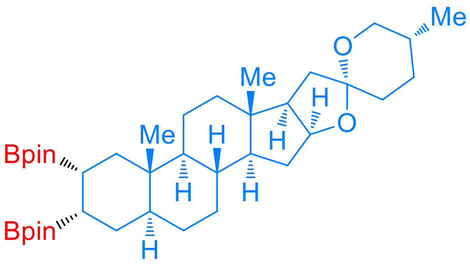

According to **General procedure 6** with (2a*S*,2'*R*,4*R*,5'*R*,6a*S*,6b*S*,8a*S*,8b*R*,11a*S*,12a*S*,12b*R*)-5',6a,8a-trimethyldocosahydrospiro[naphtho[2',1':4,5]indeno[2,1-*b*]furan-10,2'-pyran]-4-yl 4-methylbenzenesulfonate **58a** (167.0 mg, 0.3 mmol, 1.0 equiv.), the reaction mixture was purified by column chromatography on silica gel (hexane/EtOAc = 90/10) to yield the product **58b** as a white solid (140.0 mg, 219 µmol, 73% yield). **^{1}H NMR** (400 MHz, $CDCl_3$): δ = 4.40 – 4.33 (m, 1H), 3.49 – 3.44 (m, 1H), 3.36 (d, J = 11 Hz, 1H), 2.31 – 2.17 (m, 1H), 1.99 – 1.91 (m, 1H), 1.88 – 1.81 (m, 1H), 1.76 – 1.41 (m, 10H), 1.25 – .075 (m, 48H). **^{13}C{^{1}H} NMR** (100 MHz, $CDCl_3$): δ = 109.4, 83.1, 82.7, 81.1, 67.0, 62.4, 56.7, 55.2, 46.4, 41.8, 40.7, 40.2, 38.5, 36.5, 35.6, 32.6, 31.9, 31.6, 30.5, 29.3, 29.0, 25.2, 25.1, 24.9, 24.8, 20.8, 17.3, 16.7, 14.7, 12.4. **^{11}B{^{1}H} NMR** (128 MHz, $CDCl_3$): δ = 33.6. **HRMS-ASAP** (m/z): Calculated (found) for $C_{38}H_{65}B_2O_6$ $[M+H]^+$ 639.4962 (639.4954). **Anal.** for $C_{38}H_{64}B_2O_6$ calcd: C, 71.48; H, 10.10. found: C, 71.55; H, 10.03.

(2*R*,3*S*,8*R*,9*S*,10*S*,13*S*,14*S*)-10,13-Dimethyl-2,3-bis(4,4,5,5-tetramethyl-1,3,2-dioxaborolan-2-yl)tetradecahydro-1*H*-cyclopenta[*a*]phenanthren-17(2*H*)-one 59b

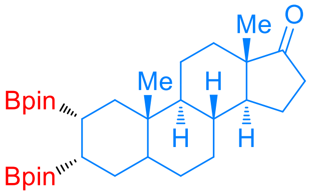

According to **General procedure 6** with (3*S*,8*R*,9*S*,10*S*,13*S*,14*S*)-10,13-dimethyl-17-oxohexadecahydro-1*H*-cyclopenta[*a*]phenanthren-3-yl 4-methylbenzenesulfonate **59a** (133.3 mg, 0.3 mmol, 1.0 equiv.), the reaction mixture was purified by column chromatography on silica gel (hexane/EtOAc = 90/10) to yield the product **59b** as a white solid (112.1 mg, 213 μmol, 71% yield). **^{1}H NMR** (500 MHz, $CDCl_3$): δ = 2.41 (dd, J = 9, 19 Hz, 1H), 2.06 – 1.99 (m, 1H), 1.92 – 1.87 (m, 1H), 1.80 – 1.67 (m, 4H), 1.56 – 1.43 (m, 5H), 1.30 – 1.21 (m, 29H), 1.11 – 0.61 (m, 11H). **^{13}C{^{1}H} NMR** (125 MHz, $CDCl_3$): δ = 221.9, 83.1, 82.7, 55.3, 51.7, 48.0, 46.4, 38.5, 36.6, 36.0, 35.5, 31.7, 31.4, 31.3, 29.0, 25.14, 25.10, 24.9, 24.7, 22.8, 21.9, 20.3, 14.2, 14.0, 12.3. **^{11}B{^{1}H} NMR** (160 MHz, $CDCl_3$): δ = 34.3. **HRMS-ASAP** (m/z): Calculated (found) for $C_{31}H_{53}B_2O_5$ $[M+H]^+$ 527.4074 (527.4066). **Anal.** for $C_{31}H_{52}B_2O_5$ calcd: C, 70.74; H, 9.96. found: C, 70.82; H, 9.84.

1.5 Application of 1,2-Diborylalkane

Procedure A:

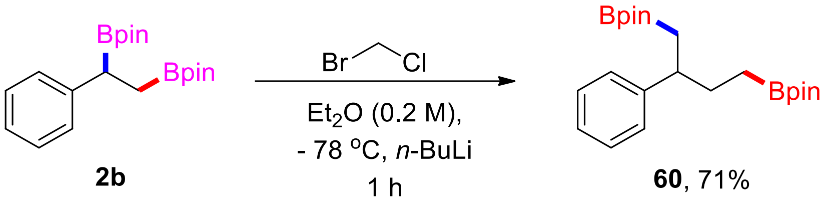

To a solution of the 1,2-bis(boronic ester) **2b** (71.6 mg, 0.2 mmol, 1.0 equiv.) in anhydrous Et_2O (0.2 M) was added bromochloromethane (155 mg, 1.2 mmol, 6.0 equiv.) under argon. The reaction mixture was cooled to -78 °C. nBuLi (2.5 M in hexane, 5.0 equiv.) was added dropwise to the reaction mixture at -78 °C. The reaction mixture was stirred at -78 °C for 20 min and the resulting reaction mixture was stirred at room temperature for 1 h. The reaction was then diluted with water and extracted 3 times with Et_2O. The organic layer was washed with brine, and then dried over Na_2SO_4, and filtered through a pad of Celite (Ø 3 mm x 8 mm), concentrated under reduced pressure and the residue was purified by flash column chromatography on silica gel to provide double homologation product **60**.

2,2'-(2-Phenylbutane-1,4-diyl)bis(4,4,5,5-tetramethyl-1,3,2-dioxaborolane) 60

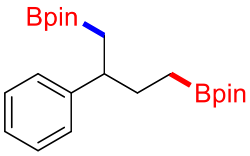

The reaction mixture was purified by column chromatography on silica gel (hexane/EtOAc = 97/3) to yield the product **60** as a colorless oil (54.8 mg, 142 μmol, 71% yield). **^{1}H NMR** (300 MHz, $CDCl_3$): δ = 7.25 – 7.08 (m, 5H), 2.79 – 2.69 (m, 1H), 1.77 – 1.55 (m, 2H), 1.27 – 1.24 (m, 2H), 1.20 (s, 12H), 1.06 (s, 6H), 1.05 (s, 6H), 0.68 – 0.59 (m, 2H). **^{13}C{^{1}H} NMR** (75 MHz, $CDCl_3$): δ = 147.2, 128.1, 127.8, 125.8, 83.0, 82.9, 44.1, 34.1, 25.0, 24.9, 24.8, 24.7. **^{11}B{^{1}H} NMR** (96 MHz, $CDCl_3$): δ = 33.1. **HRMS-ASAP** (m/z): Calculated (found) for $C_{22}H_{37}B_2O_4$ $[M+H]^+$ 387.2872 (387.2863).

The spectroscopic data for **60** match those reported in the literature.[29]

Procedure B:

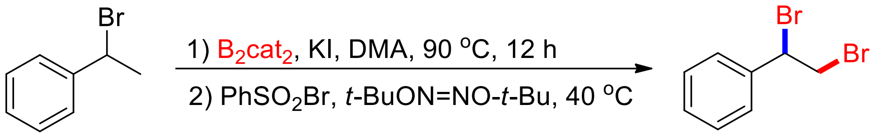

Based on the procedure described previously in literature,[30] in an argon-filled glovebox, (1-bromoethyl)benzene **2a** (55.5 mg, 0.3 mmol, 1.0 equiv.) in DMA (1 mL) were added to a 10 mL thick-walled reaction tube equipped with a magnetic stirring bar. KI (49.8 mg, 0.3 mmol, 1.0 equiv.) and B_2cat_2 (178 mg, 0.75 mmol, 2.5 equiv.) were added. The reaction mixture was stirred at 90 °C for 12 h. MeOH (58 μL, 1.44 mmol) was added at 0 °C and the solution was stirred for 15 min at room temperature. $PhSO_2Br$ (1.2 mmol) and di-*tert*-butyl hyponitrite (3.4 mg, 0.02 mmol) was added every 1h and the solution was warmed to 40 °C for 3 h. The crude product was purified by flash column chromatography on silica gel to provide the product **61**.

(1,2-Dibromoethyl)benzene 61

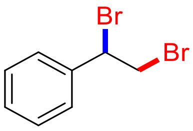

The reaction mixture was purified by column chromatography on silica gel (hexane/EtOAc = 98/2) to yield the product **61** as a colorless oil (34.1 mg, 129 μmol, 43% yield). **^{1}H NMR** (400 MHz, $CDCl_3$): δ = 7.44 – 7.34 (m, 5H), 5.16 (dd, J = 5, 8 Hz, 1H), 4.12 – 4.02 (m, 2H). **^{13}C{^{1}H} NMR** (100 MHz, $CDCl_3$): δ = 138.7, 129.3, 129.0, 127.8, 51.0, 35.2. **HRMS-ASAP** (m/z): Calculated (found) for $C_8H_9Br_2$ $[M+H]^+$ 262.9066 (262.9061).

The spectroscopic data for **61** match those reported in the literature.[31]

Procedure C:

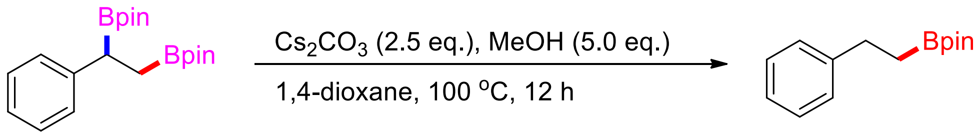

2b **62**, 88%

Based on the procedure described previously in literature,[32] to a solution of the 1,2-bis(boronic ester) **2b** (71.6 mg, 0.2 mmol, 1.0 equiv.) in anhydrous 1,4-dioxane (0.2 M), Cs_2CO_3 (163 mg, 0.5 mmol, 2.5 equiv.) and MeOH (41 µL, 1.0 mmol, 5.0 equiv.) were added under argon. The reaction mixture was stirred at 100 °C for 12 h. The reaction was then diluted with ethyl acetate, and filtered through a pad of Celite (Ø 3 mm x 8 mm), concentrated under reduced pressure and the residue was purified by flash column chromatography on silica gel to provide the product **62**.

4,4,5,5-Tetramethyl-2-phenethyl-1,3,2-dioxaborolane 62

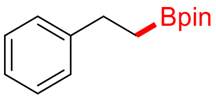

The reaction mixture was purified by column chromatography on silica gel (hexane/EtOAc = 98/2) to yield the product **62** as a colorless oil (40.8 mg, 176 µmol, 88% yield). **^{1}H NMR** (500 MHz, $CDCl_3$): δ = 7.28 – 7.21 (m, 4H), 7.17 – 7.14 (m, 1H), 2.75 (t, J = 8 Hz, 2H), 1.22 (s, 12H), 1.15 (t, J = 8 Hz, 2H). **^{13}C{^{1}H} NMR** (125 MHz, $CDCl_3$): δ = 144.5, 128.3, 128.1, 125.6, 83.2, 30.1, 24.9. **^{11}B{^{1}H} NMR** (160 MHz, $CDCl_3$): δ = 33.9. **HRMS-ASAP** (m/z): Calculated (found) for $C_{14}H_{22}BO_2$ $[M+H]^+$ 233.1707 (233.1703).

The spectroscopic data for **62** match those reported in the literature.[32]

Procedure D:

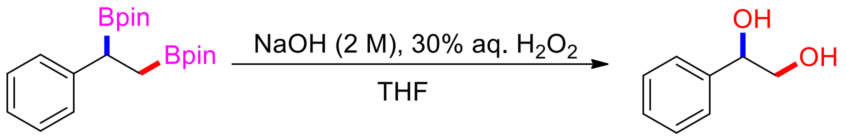

2b **63**, 73%

A premixed solution of 2 M aq. NaOH/30% aq. H_2O_2 (2:1, 3 mL) was added dropwise to a solution of the 1,2-bis(boronic ester) **2b** (71.6 mg, 0.2 mmol, 1.0 equiv.) in THF (2 mL) at 0 °C. The reaction mixture was warmed to room temperature and stirred for 4 h. The reaction was then diluted with water (2 mL) and Et_2O (2 mL), and extracted 3 times with Et_2O. The organic layer was washed with brine, and then dried over Na_2SO_4, and filtered through a pad of Celite (Ø 3 mm x 8 mm), concentrated under reduced pressure and the residue was purified by column chromatography to provide diol **63**.

1-Phenylethane-1,2-diol 63

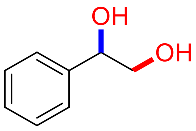

The reaction mixture was purified by column chromatography on silica gel (hexane/EtOAc = 1/2) to yield the product **63** as a white solid (20.2 mg, 146 μmol, 73% yield). **^{1}H NMR** (300 MHz, $CDCl_3$): δ = 7.37 – 7.27 (m, 5H), 4.82 (dd, J = 4, 8 Hz, 1H), 3.76 (dd, J = 4, 11 Hz, 1H), 3.66 (dd, J = 8, 11 Hz, 1H), 2.44 – 2.36 (m, 2H). **^{13}C{^{1}H} NMR** (75 MHz, $CDCl_3$): δ = 140.6, 128.7, 128.2, 126.2, 74.8, 68.2. **HRMS-ASAP** (m/z): Calculated (found) for $C_8H_{11}O_2$ $[M+H]^+$ 139.0754 (139.0745).

The spectroscopic data for **63** match those reported in the literature.[33]

Procedure E:

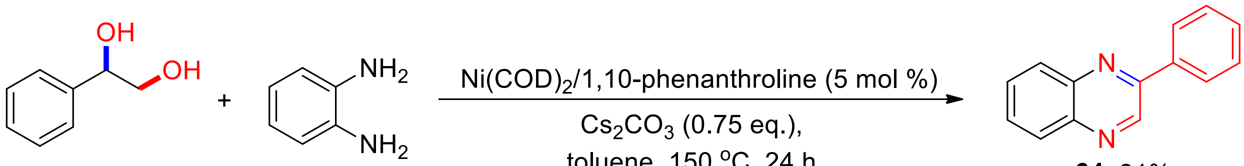

Based on the procedure described previously in literature,[34] 1,2-diamine (54.1 mg, 0.5 mmol, 1.0 equiv.), vicinal diol **63** (54.1 mg, 1.0 mmol, 2.0 equiv.), Ni(COD)$_2$ (6.9 mg, 5 mol %), 1,10-phenanthroline (4.5 mg, 5 mol %), Cs_2CO_3 (122.2 mg, 0.375 mmol, 0.75 equiv.), and 2 mL of toluene were taken in a pressure tube, and the tube was sealed under an argon atmosphere. The reaction mixture was stirred at 150 °C for 24 h. The reaction was then diluted with water (8 mL), and extracted 3 times with ethyl acetate, and filtered through a pad of Celite (Ø 3 mm x 8 mm), concentrated under reduced pressure and the residue was purified by column chromatography on silica gel to provide the quinoxaline **64**.

2-Phenylquinoxaline 64

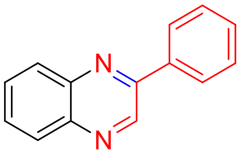

The reaction mixture was purified by column chromatography on silica gel (hexane/EtOAc = 10/1) to yield the product **64** as a yellow solid (83.5 mg, 405 μmol, 81% yield). **^{1}H NMR** (300 MHz, $CDCl_3$): δ = 9.34 (s, 1H), 8.22 – 8.13 (m, 4H), 7.83 – 7.73 (m, 2H), 7.61 – 7.53 (m, 3H). **^{13}C{^{1}H} NMR** (75 MHz, $CDCl_3$): δ = 152.1, 143.1, 142.5, 141.3, 136.7, 130.6, 130.5, 129.9, 129.7, 129.4, 129.0, 127.7. **HRMS-ASAP** (m/z): Calculated (found) for $C_{14}H_{11}N_2$ $[M+H]^+$ 207.0917 (207.0911).

The spectroscopic data for **64** match those reported in the literature.[34]

Procedure F:

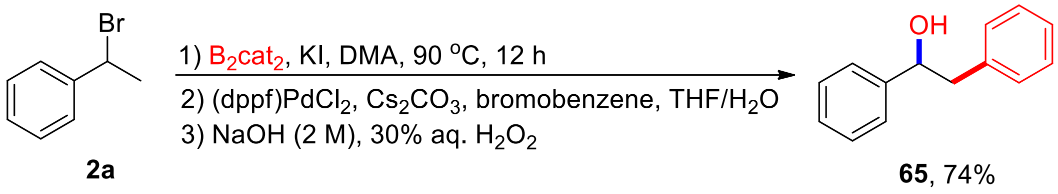

Based on the procedure described previously in literature,[35] in an argon-filled glovebox, (1-bromoethyl)benzene **2a** (55.5 mg, 0.3 mmol, 1.0 equiv.) in DMA (1 mL) were added to a 10 mL thick-walled reaction tube equipped with a magnetic stirring bar. KI (49.8 mg, 0.3 mmol, 1.0 equiv.) and B_2cat_2 (178 mg, 0.75 mmol, 2.5 equiv.) were added. The reaction mixture was stirred at 90 °C for 12 h. After completion of reaction, DMA was removed in vacuo. (dppf)$PdCl_2$ (22 mg, 10 mol%), Cs_2CO_3 (293 mg, 0.9 mmol, 3.0 equiv.), bromobenzene (94.2 mg, 0.6 mmol, 2.0 equiv.) and THF (2 mL) were added and the solution was stirred for 1 min. 200 μL of deoxygenated water was added to the mixture and the solution was heated to 80 °C for 18 h. After this time, a premixed solution of 2 M aq. NaOH/30% aq. H_2O_2 (2:1, 4.5 mL) was added dropwise to the mixture at 0 °C. The reaction mixture was warmed to room temperature and stirred for 6 h. The reaction was then diluted with H_2O (2 mL) and Et_2O (2 mL), and extracted 3 times with Et_2O. The organic layer was washed with brine, and then dried over Na_2SO_4, and filtered through a pad of Celite (Ø 3 mm x 8 mm), concentrated under reduced pressure and the residue was purified by column chromatography to provide **65**.

1,2-Diphenylethanol 65

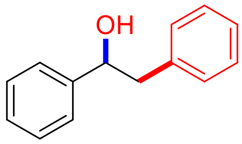

The reaction mixture was purified by column chromatography on silica gel (hexane/EtOAc = 10/1) to yield the product **65** as a white solid (44 mg, 222 μmol, 74% yield). **^{1}H NMR** (300 MHz, $CDCl_3$): δ = 7.38 – 7.19 (m, 10H), 4.91 (d, J = 5, 8 Hz, 1H), 3.10 – 2.96 (m, 2H), 1.93 – 1.89 (m, 1H). **$^{13}C\{^1H\}$ NMR** (75 MHz, $CDCl_3$): δ = 143.9, 138.2, 129.6, 128.6, 128.5, 127.7, 126.7, 126.0, 75.5, 46.2. **HRMS-ASAP** (m/z): Calculated (found) for $C_{14}H_{15}O$ $[M+H]^+$ 199.1117 (199.1115).

The spectroscopic data for **65** match those reported in the literature.[35]

Procedure G:

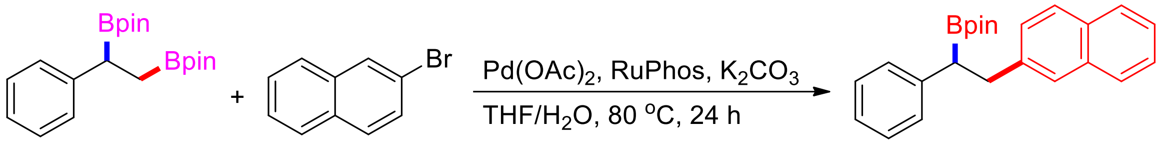

Based on the procedure described previously in literature,[36] in an argon-filled glovebox, 1,2-bis(boronic ester) **2b** (143.2 mg, 0.4 mmol, 1.0 equiv.), 2-bromonaphthalene (99.4 mg, 0.48 mmol, 1.2 equiv.), $Pd(OAc)_2$ (9 mg, 0.04 mmol, 0.1 equiv.), Ruphos (46.7 mg, 0.1 mmol, 0.25 equiv.) and K_2CO_3 (110.6 mg, 0.8 mmol, 2.0 equiv.) were weighed into a 10 mL thick-walled reaction tube and THF (2 mL) was added. Degassed water was added (20:1, organic:water) and the reaction mixture was stirred at 80 °C for 24 h. After completion of reaction, the reaction mixture was filtered through a plug of silica, washed through with EtOAc and concentrated under reduced pressure and the residue was purified by column chromatography to provide **66**.

4,4,5,5-Tetramethyl-2-(2-(naphthalen-2-yl)-1-phenylethyl)-1,3,2-dioxaborolane 66

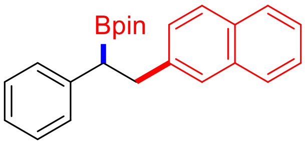

The reaction mixture was purified by column chromatography on silica gel (hexane/EtOAc = 97/3) to yield the product **66** as a colorless oil (73.1 mg, 204 μmol, 51% yield). **^{1}H NMR** (500 MHz, $CDCl_3$): δ = 7.78 – 7.76 (m, 1H), 7.74 – 7.70 (m, 2H), 7.64 – 7.63 (m, 1H), 7.43 – 7.38 (m, 2H), 7.33 (dd, J = 2, 9 Hz, 1H), 7.29 – 7.24 (m, 4H), 7.16 – 7.13 (m, 1H), 3.34 (dd, J = 10, 14 Hz, 1H), 3.12 (dd, J = 7, 14 Hz, 1H), 2.79 (dd, J = 7, 10 Hz, 1H), 1.09 (d, J = 7 Hz, 12H). **^{13}C{^{1}H} NMR** (125 MHz, $CDCl_3$): δ = 142.7, 139.5, 133.6, 132.1, 128.6, 128.5, 127.9, 127.7, 127.6, 127.0, 125.8, 125.6, 125.1, 83.6, 39.1, 24.71, 24.68. **^{11}B{^{1}H} NMR** (160 MHz, $CDCl_3$): δ = 33.0. **HRMS-ASAP** (m/z): Calculated (found) for $C_{24}H_{28}BO_2$ $[M+H]^+$ 359.2177 (359.2171).

The spectroscopic data for **66** match those reported in the literature.[36]

Procedure H:

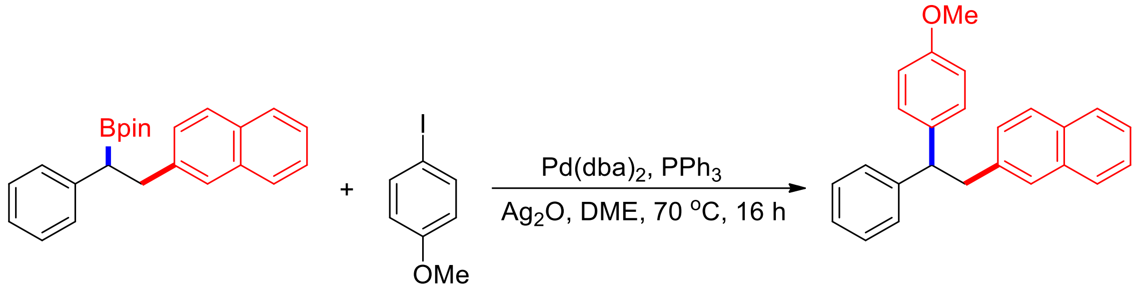

66 **67**, 73%

Based on the procedure described previously in literature,[36] in an argon-filled glovebox, boronic ester **66** (71.6 mg, 0.2 mmol, 1.0 equiv.), 1-iodo-4-methoxybenzene (70.2 mg, 0.3 mmol, 1.5 equiv.), $Pd(dba)_2$ (9 mg, 0.016 mmol, 0.08 equiv.), PPh_3 (33.6 mg, 0.128 mmol, 0.64 equiv.) and Ag_2O (69.5 mg, 0.3 mmol, 1.5 equiv.) were weighed into a 10 mL thick-walled reaction tube and dimethoxyethane (DME) (2 mL) was added. The reaction mixture was stirred at 70 °C for 16 h. After completion of reaction, the reaction mixture was filtered through a plug of silica, washed through with EtOAc and concentrated under reduced pressure and the residue was purified by column chromatography to provide **67**.

2-(2-(4-Methoxyphenyl)-2-phenylethyl)naphthalene 67

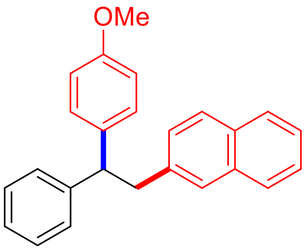

The reaction mixture was purified by column chromatography on silica gel (hexane/EtOAc = 98/2) to yield the product **67** as a colorless oil (49.4 mg, 146 μmol, 73% yield). **^{1}H NMR** (500 MHz, $CDCl_3$): δ = 7.77 – 7.75 (m, 1H), 7.69 – 7.66 (m, 2H), 7.45 – 7.39 (m, 3H), 7.25 – 7.22 (m, 4H), 7.17 – 7.14 (m, 4H), 6.79 (d, J = 8 Hz, 2H), 4.31 (t, J = 8 Hz, 1H), 3.76 (s, 3H), 3.50 (d, J = 8 Hz, 2H). **^{13}C{^{1}H} NMR** (125 MHz, $CDCl_3$): δ = 158.1, 145.0, 138.1, 136.7, 133.6, 132.1, 129.1, 128.5, 128.1, 127.88, 127.86, 127.67, 127.65, 127.6, 126.3, 125.8, 125.3, 113.9, 55.3, 52.3, 42.6. **HRMS-ASAP** (m/z): Calculated (found) for $C_{25}H_{23}O$ $[M+H]^+$ 339.1743 (339.1737).

The spectroscopic data for **67** match those reported in the literature.[36]

Procedure I:

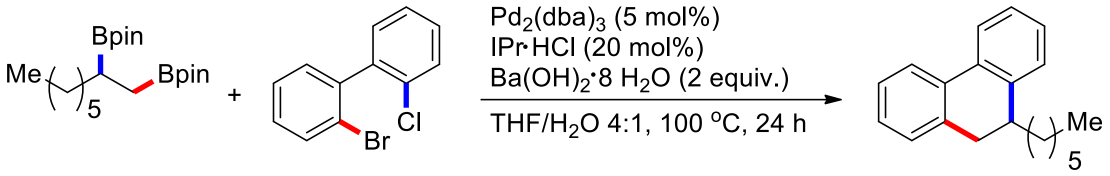

40b **68**, 50%

Based on the procedure described previously in literature,[37] in an argon-filled glovebox, boronic ester **40b** (183 mg, 0.5 mmol, 1.0 equiv.), 2-bromo-2'-chloro biaryl (133.8 mg, 0.5 mmol, 1.0 equiv.), $Pd_2(dba)_3$ (23 mg, 0.025 mmol, 5 mol%), IPr•HCl (42.7 mg, 0.1 mmol, 20 mol%) and $Ba(OH)_2$•$8H_2O$ (315.5 mg, 1.0 mmol, 2.0 equiv.) were weighed into a 50 mL Schlenk flask and THF (10 mL)/water (2.5 mL) was added. The reaction mixture was stirred at 100 °C for 24 h. The reaction was then diluted with water (5 mL) and MTBE (10 mL). The organic phase was extracted 3 times with MTBE, and then dried over Na_2SO_4, and filtered through a pad of Celite (Ø 3 mm x 8 mm), concentrated under reduced pressure and the residue was purified by column chromatography to provide compound **68**.

2-Bromo-2'-chloro biaryl

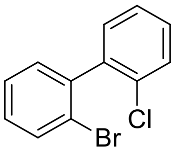

The synthesis was performed according to Morandi *et al.*[37] To a solution of 1-bromo-2-chlorobenzene (2.35 mL, 20 mmol, 2.0 equiv.) in THF (40 mL) was added a hexane solution of *n*-butyllithium (4.0 mL, 2.5 M, 10 mmol, 1.0 equiv.) slowly under argon at -78 °C and the resulting reaction mixture was stirred at room temperature for 4 h. The reaction was quenched by aqueous HCl solution (25 mL, 2M). The organic layer was extracted with DCM, washed with brine, and then dried over Na_2SO_4, and filtered through a pad of Celite (Ø 3 mm x 8 mm), concentrated under reduced pressure and the residue was purified by column chromatography to provide the product. **^{1}H NMR** (300 MHz, $CDCl_3$): δ = 7.71 – 7.67 (m, 1H), 7.51 – 7.48 (m, 1H), 7.42 – 7.33 (m, 3H), 7.29 – 7.24 (m, 3H). **^{13}C{^{1}H} NMR** (75 MHz, $CDCl_3$): δ = 140.6, 140.2, 133.5, 132.7, 131.22, 131.20, 129.6, 129.5, 129.4, 127.3, 126.6, 123.8. **HRMS-ASAP** (m/z): Calculated (found) for $C_{12}H_9BrCl$ $[M+H]^+$ 266.9571 (266.9566).

The spectroscopic data for **2-Bromo-2'-chloro biaryl** match those reported in the literature.[37]

9-Hexyl-9,10-dihydrophenanthrene 68

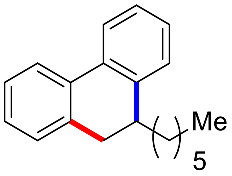

The reaction mixture was purified by column chromatography on silica gel (hexane/EtOAc = 98/2) to yield the product **68** as a colorless oil (66 mg, 250 µmol, 50% yield). **^{1}H NMR** (500 MHz, $CDCl_3$): δ = 7.75 (t, J = 8 Hz, 2H), 7.32 – 7.28 (m, 2H), 7.26 – 7.20 (m, 4H), 3.10 (dd, J = 5, 15 Hz, 1H), 2.86 – 2.81 (m, 1H), 2.78 (dd, J = 5, 15 Hz, 1H), 1.41 – 1.21 (m, 10H), 0.85 (t, J = 7 Hz, 3H). **^{13}C{^{1}H} NMR** (125 MHz, $CDCl_3$): δ = 141.5, 135.7, 134.2, 133.6, 129.1, 128.3, 127.6, 127.4, 127.0, 126.9, 124.1, 123.5, 38.7, 34.1, 33.5, 32.0, 29.5, 27.7, 22.8, 14.2. **HRMS-ASAP** (m/z): Calculated (found) for $C_{20}H_{25}$ $[M+H]^+$ 265.1951 (265.1943).

The spectroscopic data for **68** match those reported in the literature.[37]

Procedure J:

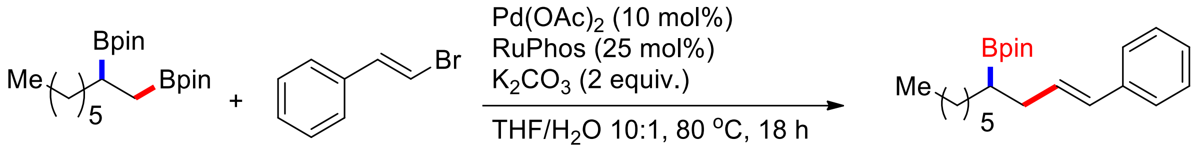

In an argon-filled glovebox, boronic ester **40b** (183 mg, 0.5 mmol, 1.0 equiv.), (*E*)-(2-bromovinyl)benzene (109.8 mg, 0.6 mmol, 1.2 equiv.), $Pd(OAc)_2$ (11.2 mg, 0.05 mmol, 10 mol%), RuPhos (58.3 mg, 0.125 mmol, 25 mol%) and K_2CO_3 (138.2 mg, 1.0 mmol, 2.0 equiv.) were weighed into a 10 mL Schlenk flask and THF (5 mL)/water (0.5 mL) was added. The reaction mixture was stirred at 80 °C for 18 h. The reaction was then diluted with water (5 mL) and MTBE (10 mL). The organic phase was extracted 3 times with MTBE, and then dried over Na_2SO_4, and filtered through a pad of Celite (Ø 3 mm x 8 mm), concentrated under reduced pressure and the residue was purified by column chromatography to provide compound **69**.

(*E*)-4,4,5,5-Tetramethyl-2-(1-phenyldec-1-en-4-yl)-1,3,2-dioxaborolane 69

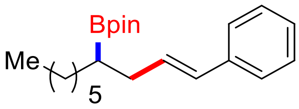

The reaction mixture was purified by column chromatography on silica gel (hexane/EtOAc = 98/2) to yield the product **69** as a colorless oil (104.4 mg, 305 μmol, 61% yield). **^{1}H NMR** (500 MHz, $CDCl_3$): δ = 7.33 – 7.27 (m, 4H), 7.19 – 7.16 (m, 1H), 6.39 (d, *J* = 16 Hz, 1H), 6.26 – 6.20 (m, 1H), 2.33 – 2.28 (m, 1H), 1.49 – 1.25 (m, 12H), 1.22 (s, 12H), 1.22 (s, 12H), 0.88 (t, *J* = 7 Hz, 3H). **^{13}C{^{1}H} NMR** (125 MHz, $CDCl_3$): δ = 138.1, 131.0, 130.2, 128.5, 126.8, 126.1, 83.1, 34.9, 32.0, 31.2, 29.7, 29.3, 25.0, 24.96, 22.8, 14.2. **^{11}B{^{1}H} NMR** (160 MHz, $CDCl_3$): δ = 34.4. **HRMS-ASAP** (m/z): Calculated (found) for $C_{22}H_{36}BO_2$ $[M+H]^+$ 343.2803 (343.2796). **Anal.** for $C_{22}H_{35}BO_2$ calcd: C, 77.19; H, 10.31. found: C, 77.32; H, 10.26.

Procedure K:

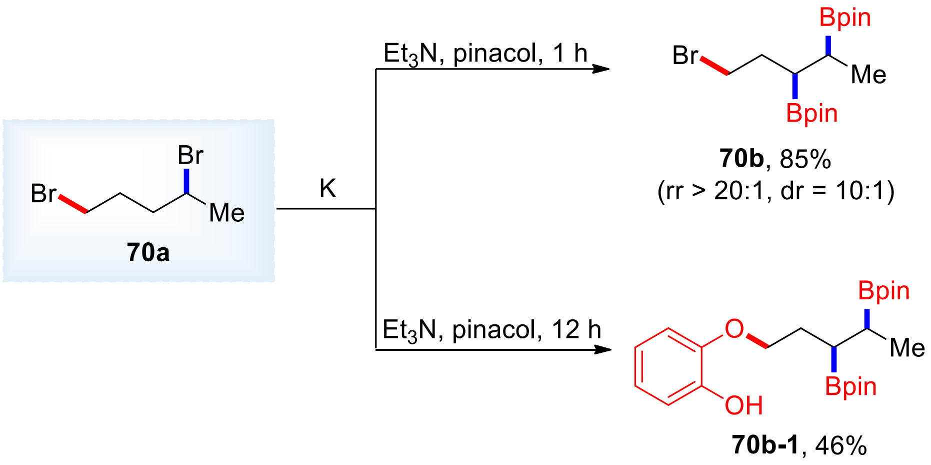

In an argon-filled glovebox, 1,4-dibromopentane (69.0 mg, 0.3 mmol, 1.0 equiv.) in DMA (1 mL) were added to a 10 mL thick-walled reaction tube equipped with a magnetic stirring bar. KI (49.8 mg, 0.3 mmol, 1.0 equiv.) and B_2cat_2 (178 mg, 0.75 mmol, 2.5 equiv.) were added. The reaction mixture was stirred at 90 °C for 12 h.

Then a solution of pinacol (106 mg, 0.9 mmol, 3.0 equiv.) in Et_3N (1 mL) was added to the reaction mixture, which was stirred at room temperature for 1 h or 12 h. Then water was added, and the reaction mixture was extracted with EtOAc three times. The combined organic layer was dried over Na_2SO_4, filtered, and concentrated under reduced pressure and the residue was purified by column chromatography on silica gel to provide product **70b** or **70b-1**.

2,2'-(5-Bromopentane-2,3-diyl)bis(4,4,5,5-tetramethyl-1,3,2-dioxaborolane) 70b

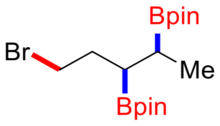

The reaction mixture was purified by column chromatography on silica gel (hexane/EtOAc = 98/2) to yield the product **70b** as a colorless oil (102.7 mg, 255 μmol, 85% yield). **^{1}H NMR** (400 MHz, $CDCl_3$): δ = 3.54 – 3.46 (m, 1H), 3.44 – 3.36 (m, 1H), 2.16 – 2.01 (m, 1H), 1.94 – 1.79 (m, 1H), 1.32 – 1.25 (m, 1H), 1.22 (m, 24H), 1.19 – 1.16 (m, 1H), 0.99 – 0.95 (m, 3H). **^{13}C{^{1}H} NMR** (100 MHz, $CDCl_3$): δ = 83.2, 83.1, 34.3, 33.5, 25.1, 25.06, 24.8, 24.8, 14.7. **^{11}B{^{1}H} NMR** (128 MHz, $CDCl_3$): δ = 33.9. **HRMS-ASAP** (m/z): Calculated (found) for $C_{17}H_{34}B_2BrO_4$ $[M+H]^+$ 403.1821 (403.1817). **Anal.** $C_{17}H_{33}B_2BrO_4$ calcd: C, 50.67; H, 8.25. found: C, 50.73; H, 8.12.

2-((3,4-Bis(4,4,5,5-tetramethyl-1,3,2-dioxaborolan-2-yl)pentyl)oxy)phenol 70b-1

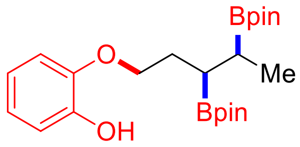

The reaction mixture was purified by column chromatography on silica gel (hexane/EtOAc = 4/1) to yield the product **70b-1** as a white solid (59.6 mg, 138 μmol, 46% yield). **^{1}H NMR** (500 MHz, $CDCl_3$): δ = 6.92 – 6.90 (m, 1H), 6.87 – 6.82 (m, 2H), 6.80 – 6.77 (m, 1H), 6.69 (s, 1H), 4.09 – 4.04 (m, 1H), 4.03 – 3.99 (m, 1H), 2.06 – 1.99 (m, 1H), 1.83 – 1.77 (m, 1H), 1.35 – 1.30 (m, 2H), 1.25 – 1.24 (m, 24H), 1.00 (d, J = 8 Hz, 3H). **^{13}C{^{1}H} NMR** (125 MHz, $CDCl_3$): δ = 146.7, 146.6, 121.5, 119.8, 114.8, 112.8, 83.4, 83.2, 69.9, 29.5, 25.1, 25.0, 24.8, 24.7, 14.5. **^{11}B{^{1}H} NMR** (160 MHz, $CDCl_3$): δ = 34.5. **HRMS-ASAP** (m/z): Calculated (found) for $C_{23}H_{39}B_2O_6$ [M+H]$^+$ 433.2927 (433.2921). **Anal.** $C_{23}H_{38}B_2O_6$ calcd: C, 63.92; H, 8.86. found: C, 63.85; H, 8.97.

2 Preliminary Mechanistic Investigations

a) Radical trap experiments

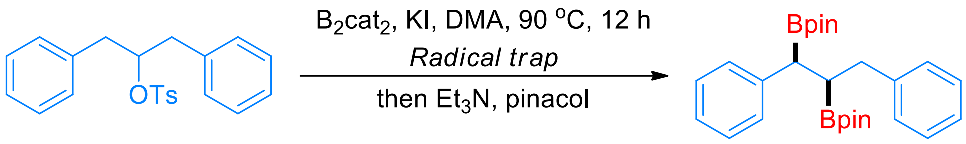

22a

Radical trap	**22b**
None	81%
9,10-dihydroanthracene	75%
BHT	76%

In an argon-filled glovebox, 1,3-diphenylpropan-2-yl 4-methylbenzenesulfonate **22a** (109.9 mg, 0.3 mmol, 1.0 equiv.) in DMA (1 mL) were added to a 10 mL thick-walled reaction tube equipped with a magnetic stirring bar. KI (49.8 mg, 0.3 mmol, 1.0 equiv.), B_2cat_2 (178 mg, 0.75 mmol, 2.5 equiv.), and radical trap [9,10-dihydroanthracene (108.2 mg, 0.6 mmol, 2.0 equiv.) or BHT (132.2 mg, 0.6 mmol, 2.0 equiv.)] were added. The reaction mixture was stirred at 90 °C for 12 h. Then a solution of pinacol (106 mg, 0.9 mmol, 3.0 equiv.) in Et_3N (1 mL) was added to the reaction mixture, which was stirred at room temperature for 1 h. Then water was added, and the reaction mixture was extracted with EtOAc three times. The combined organic layer was dried over Na_2SO_4, filtered, and concentrated under reduced pressure and the residue was purified by column chromatography on silica gel to provide product **22b** in 75% or 76% yield, respectively.

b) Role of KI

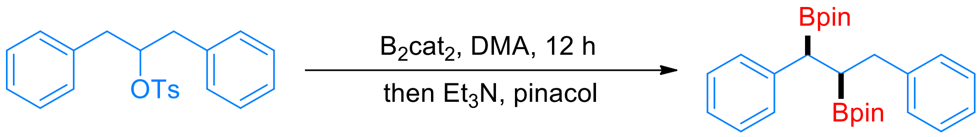

22a → **22b**

T/°C	KI	**22b**
80	✗	trace
80	✓	72%

In an argon-filled glovebox, 1,3-diphenylpropan-2-yl 4-methylbenzenesulfonate **22a** (109.9 mg, 0.3 mmol, 1.0 equiv.) in DMA (1 mL) were added to a 10 mL thick-walled reaction tube equipped with a magnetic stirring bar. B_2cat_2 (178 mg, 0.75 mmol, 2.5 equiv.) was added with or without addition of KI (49.8 mg, 0.3 mmol, 1.0 equiv.). The reaction mixture was stirred at 80 °C for 12 h. Then a solution of pinacol (106 mg, 0.9 mmol, 3.0 equiv.) in Et_3N (1 mL) was added to the reaction mixture, which was stirred at room temperature for 1 h. Then water was added, and the reaction mixture was extracted with EtOAc three times. The combined organic layer was dried over Na_2SO_4, filtered, and concentrated under reduced pressure, *n*-dodecane was added as an internal standard and the crude reaction mixture was analyzed by GC-MS. Trace amount of the diborylated product **22b** was observed by GC-MS without the addition of KI. However, 72% yield of **22b** was obtained with the addition of KI. It suggested KI played a crucial role for the generation of diborylated products.

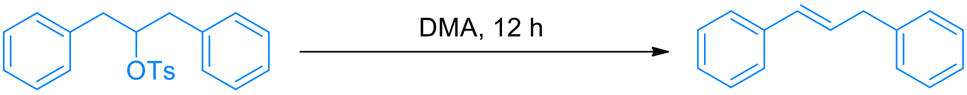

22a → **22c**

T/°C	KI	**22c**	*E/Z*
80	✗	19%	59/41
80	✓	78%	98/2
90	✓	85%	98/2

In an argon-filled glovebox, 1,3-diphenylpropan-2-yl 4-methylbenzenesulfonate **22a** (109.9 mg, 0.3 mmol, 1.0 equiv.) in DMA (1 mL) were added to a 10 mL thick-walled reaction tube equipped with a magnetic stirring bar. The reaction mixture was stirred at indicated temperature for 12 h. Then the reaction mixture was purified by column chromatography on silica gel to provide product olefin **22c**. 1) The olefin **22c** was obtained in only 19% yield with an average stereoselectivity. 2) Upon addition of KI, the olefin **22c** was isolated in 78%

yield with excellent *E/Z* ratio at 80 °C. The results implied that alkyl alkenes might be the active intermediates of this transformation.

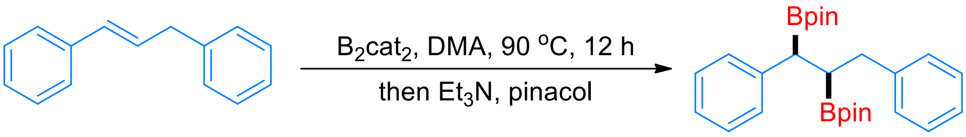

22c

KI	**22b**
✗	78%
✓	81%

In an argon-filled glovebox, olefin **22c** (58.3 mg, 0.3 mmol, 1.0 equiv.) in DMA (1 mL) were added to a 10 mL thick-walled reaction tube equipped with a magnetic stirring bar. B_2cat_2 (178 mg, 0.75 mmol, 2.5 equiv.) was added with or without addition of KI (49.8 mg, 0.3 mmol, 1.0 equiv.). The reaction mixture was stirred at 90 °C for 12 h. Then a solution of pinacol (106 mg, 0.9 mmol, 3.0 equiv.) in Et_3N (1 mL) was added to the reaction mixture, which was stirred at room temperature for 1 h. Then water was added, and the reaction mixture was extracted with EtOAc three times. The combined organic layer was dried over Na_2SO_4, filtered, and concentrated under reduced pressure and the residue was purified by column chromatography on silica gel to provide diborylated product **22b** in 78% or 81% yield, respectively. **22b** was obtained in similar high yields regardless of the presence or absence of KI under the standard conditions.

(*E*)-Prop-1-ene-1,3-diyldibenzene 22c

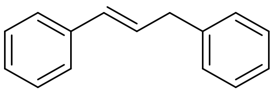

^{1}H NMR (500 MHz, $CDCl_3$): δ = 7.39 – 7.20 (m, 10H), 6.49 – 6.35 (m, 2H), 3.57 (d, J = 7 Hz, 2H). **^{13}C{^{1}H} NMR** (125 MHz, $CDCl_3$): δ = 140.3, 137.6, 131.2, 129.4, 128.8, 128.64, 128.63, 127.2, 126.32, 126.26, 39.5. **HRMS-ASAP** (m/z): Calculated (found) for $C_{15}H_{15}$ $[M+H]^+$ 195.1168 (195.1165).

The spectroscopic data for **22c** match those reported in the literature.[38]

c) Diboration of iodides

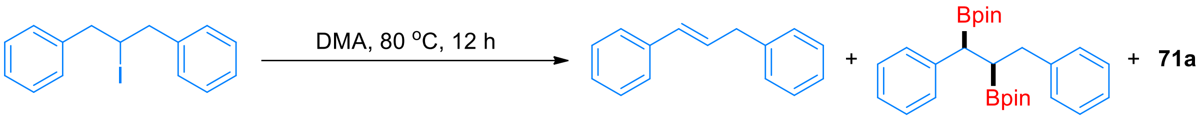

71a 22c 22b

KI	B_2cat_2	22c	22b	71a
✗	✗	18% (*E*/*Z*, 61/39)	-	71%
✓	✗	74% (*E*/*Z*, 95/5)	-	0%
✗	✓	0%	12%	79%
✓	✓	0%	78%	0%
KOTf	✗	71% (*E*/*Z*, 91/9)	-	0%

In order to confirm whether the process undergoes iodine replacement (the replacement of iodine in KI with OTs in **22a**), the alkyl iodide (**71a**) was subjected to standard conditions.

In the absence of B_2cat_2, we observed a similar phenomenon, and KI promoted the formation of *E*-alkenes (**22c**).

When KI and B_2cat_2 were added into the reaction at the same time, the yield of the target product increased sharply.

These results indicated that the presence of KI enhanced both the reactivity and regioselectivity and diastereoselectivity of the reaction. However, this does not rule out the possibility of initial iodide exchange taking place.

d) Role of DMA

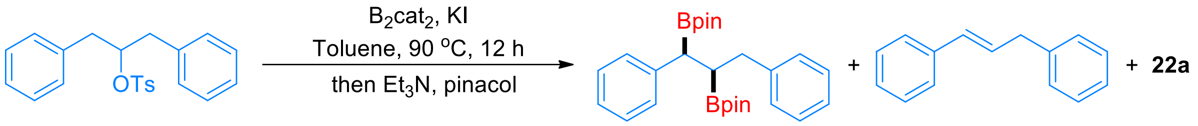

22a

Additive	22b	22c	
None	0%	0%	92%
DMA (3.0 equiv.)	19%	0%	74%
DMAP (3.0 equiv.)	11%	0%	76%

In an argon-filled glovebox, 1,3-diphenylpropan-2-yl 4-methylbenzenesulfonate **22a** (109.9 mg, 0.3 mmol, 1.0 equiv.) in toluene (1 mL) were added to a 10 mL thick-walled reaction tube equipped with a magnetic stirring bar. KI (49.8 mg, 0.3 mmol, 1.0 equiv.) and B_2cat_2 (178 mg, 0.75 mmol, 2.5 equiv.), were added. The reaction mixture was stirred at 90 °C for 12 h. Then a solution of pinacol (106 mg, 0.9 mmol, 3.0 equiv.) in Et_3N (1 mL) was added to the reaction mixture, which was stirred at room temperature for 1 h. Then water was added, and the reaction mixture was extracted with EtOAc three times. The combined organic layer was dried over Na_2SO_4, filtered, and concentrated under reduced pressure, *n*-dodecane was added as an internal standard and the crude reaction mixture was analyzed by GC-MS. The diborylated product **22b** was not observed by GC-MS. When DMA (3.0 equiv.) or DMAP (3.0 equiv.) was added to the reaction mixture, the diborylated product **22b** was generated.

3 Single-Crystal X-ray Diffraction Analysis

A crystal suitable for single-crystal X-ray diffraction was selected, coated in perfluoropolyether oil, and mounted on a microloop. Diffraction data of **13b**, **18b**, **22b**, **26b**, **28b**, and **59b** were collected on a RIGAKU OXFORD DIFFRACTION XTALAB Synergy-S, Dualflex, four-circle diffractometer with a semiconductor HPA-detector (HyPix-6000) and micro-focus sealed X-ray tubes providing multi-layer mirror monochromated Cu-K_α radiation and, for compound **18b**, Mo-K_α radiation The crystals were cooled at 100 K, or 173 K for compound **22b**, using an Oxford Cryostream 800 low-temperature device. The images were processed and corrected for Lorentz-polarization effects and absorption as implemented in the CrysAlis^{Pro} software. The structures were solved using the intrinsic phasing method (SHELXT)[39] and Fourier expansion technique. All non-hydrogen atoms were refined in anisotropic approximation, with hydrogen atoms 'riding' on idealised positions by full-matrix least squares against F^2 of all data, using SHELXL software[40] and the SHELXLE graphical user interface[41]. Disordered moieties were refined using bond lengths restraints and displacement parameter restraints. Diamond[42] software was used for graphical representation. Crystal data and experimental details are listed in Tables S2 and S3; full structural information has been deposited with Cambridge Crystallographic Data Centre. CCDC-2119847 (**13b**), 2119849 (**18b**), 2119873 (**22b**), 2119851 (**26b**), 2119856 (**28b**), and 2119852 (**59b**). These data can be obtained free of charge from The Cambridge Crystallographic Data Centre *via* www.ccdc.cam.ac.uk/data_request/cif.

Table S2: Single-crystal X-ray diffraction data and refinement details of **13b**, **18b**, and **22b**.

Data	**13b**	**18b**	**22b**
CCDC number	2119847	2119849	2119873
Empirical formula	$C_{21}H_{33}B_2BrO_4$	$C_{23}H_{38}B_2O_4$	$C_{27}H_{38}B_2O_4$
Formula weight / g·mol^{-1}	451.00	400.15	448.19
T / K	100(2)	100(2)	173(2)
Radiation, λ / Å	Cu-K$_\alpha$ 1.54184	Mo-K$_\alpha$ 0.71073	Cu-K$_\alpha$ 1.54184
Crystal size / mm³	0.057×0.097×0.329	0.155×0.298×0.543	0.465×0.230×0.214
Crystal color, habit	Colorless needle	Colorless plate	Colorless block
μ / mm^{-1}	2.604	0.073	0.564
Crystal system	Monoclinic	Monoclinic	Monoclinic
Space group	$P2_1/c$	$P2_1/n$	$P2_1/c$
a / Å	9.04840(10)	6.6217(2)	20.4392(3)
b / Å	21.9005(4)	40.7610(13)	9.80950(10)
c / Å	11.9134(2)	9.0118(3)	26.8903(3)
α / °	90	90	90
β / °	102.4160(10)	104.716(4)	99.0130(10)
γ / °	90	90	90
Volume / Å^3	2305.60(6)	2352.55(15)	5324.89(11)
Z	4	4	8
ρ_{calc} / g·cm^{-3}	1.299	1.130	1.118
F(000)	944	872	1936
θ range / °	4.037 – 74.503	1.998 – 26.370	2.189 – 80.217
Reflections collected	24717	17800	50427
Unique reflections	4722	4817	11417
Parameters / restraints	560 / 661	532 / 1032	1054 / 1920
GooF on F^2	1.065	1.097	1.056
R_1 [I>2σ (I)]	0.0402	0.0712	0.0719
wR^2 (all data)	0.1098	0.1685	0.1782
Max. / min. residual electron density / e·Å^{-3}	0.525 / –0.426	0.419 / –0.247	0.284 / –0.275

Table S3: Single-crystal X-ray diffraction data and refinement details of **26b**, **28b**, and **59b**.

Data	**26b**	**28b**	**59b**
CCDC number	2119851	2119856	2119852
Empirical formula	$C_{18}H_{34}B_2O_4$	$C_{22}H_{33}B_2BrO_4$	$C_{31}H_{52}B_2O_5$
Formula weight / g·mol^{-1}	336.07	463.01	526.34
T / K	100(2)	100(2)	100(2)
Radiation, λ / Å	Cu-K$_\alpha$ 1.54184	Cu-K$_\alpha$ 1.54184	Mo-K$_\alpha$ 0.71073
Crystal size / mm^3	0.021×0.104×0.202	0.122×0.240×0.334	0.157×0.321×0.403
Crystal color, habit	Colorless plate	Colorless plate	Colorless block
μ / mm^{-1}	0.609	2.601	0.073
Crystal system	Triclinic	Monoclinic	Monoclinic
Space group	$P\bar{1}$	$P2_1$	$P2_1$
a / Å	9.7006(2)	12.98450(10)	14.0122(4)
b / Å	10.0093(3)	12.24660(10)	7.93797(17)
c / Å	11.9545(2)	14.83570(10)	14.5453(4)
α / °	72.544(2)	90	90
β / °	68.499(2)	100.1580(10)	106.337(3)
γ / °	65.208(3)	90	90
Volume / Å^3	965.61(5)	2322.13(3)	1552.53(7)
Z	2	4	2
ρ_{calc} / g·cm^{-3}	1.156	1.324	1.126
F(000)	368	968	576
θ range / °	4.035 – 74.478	3.026 – 74.491	2.380 – 26.370
Reflections collected	20282	45749	31892
Unique reflections	3946	9474	6338
Parameters / restraints	225 / 0	539 / 1	430 / 229
GooF on F^2	1.060	1.041	1.044
R_1 [I>2σ (I)]	0.0483	0.0231	0.0406
wR^2 (all data)	0.1407	0.0582	0.1070
Max. / min. residual electron density / e·Å^{-3}	0.511 / –0.302	0.205 / –0.361	0.348 / –0.302

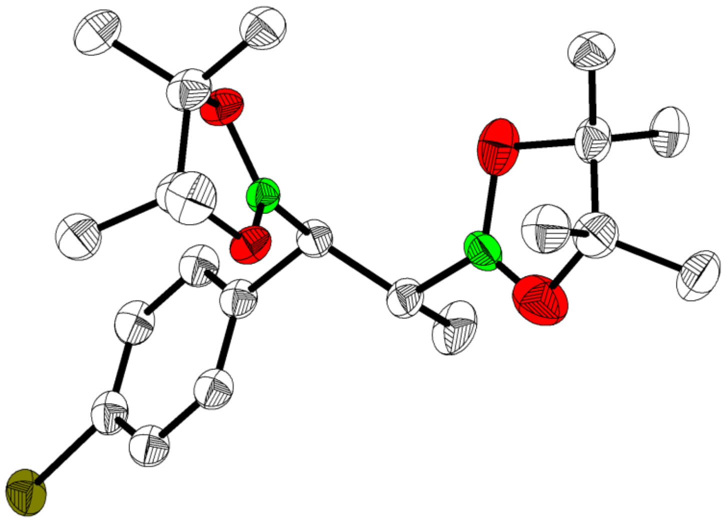

Figure S1. The solid-state molecular structure of **13b** determined by single-crystal X-ray diffraction at 100 K. All ellipsoids are drawn at the 50% probability level, and H atoms are omitted for clarity. The molecule is completely disordered with its enantiomer and only the major part (66%) is shown here. As the crystal structure is centrosymmetric, both enantiomers are present in equal amounts. Atom colors: carbon (white), boron (green), oxygen (red), bromium (brown).

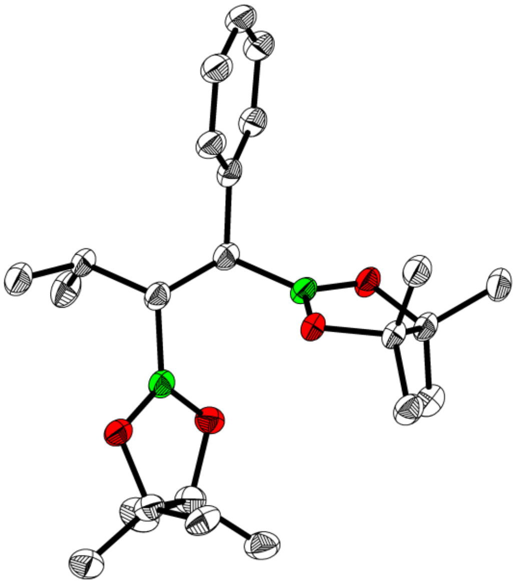

Figure S2. The solid-state molecular structure of **18b** determined by single-crystal X-ray diffraction at 100 K. All ellipsoids are drawn at the 50% probability level, and H atoms are omitted for clarity. The molecule is completely disordered with its enantiomer and only the major part (93%) is shown here. Atom colors: carbon (white), boron (green), oxygen (red).

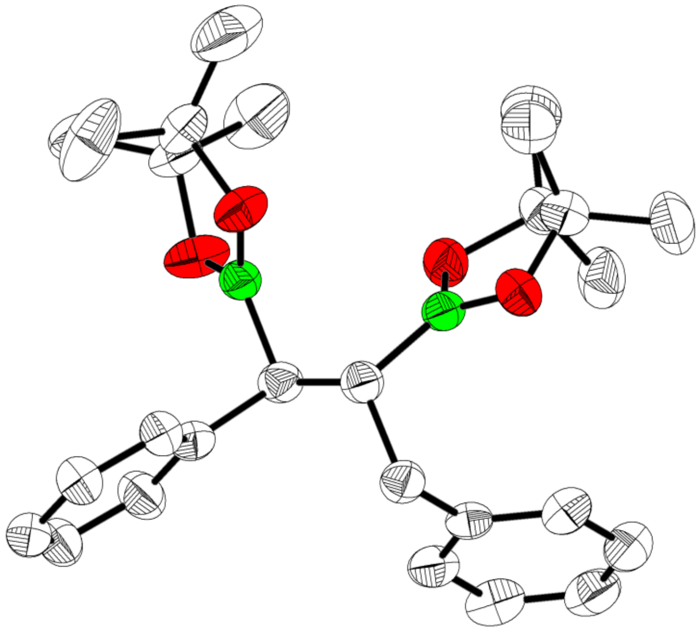

Figure S3. The solid-state molecular structure of **22b** determined by single-crystal X-ray diffraction at 173 K. All ellipsoids are drawn at the 50% probability level, and H atoms are omitted for clarity. The unit cell contains two symmetry-independent molecules and they are enantiomers. The molecules are completely disordered with their enantiomers and only the major part (72%) of one of the molecules is shown here. Both enantiomers are present in equal amounts. Atom colors: carbon (white), boron (green), oxygen (red).

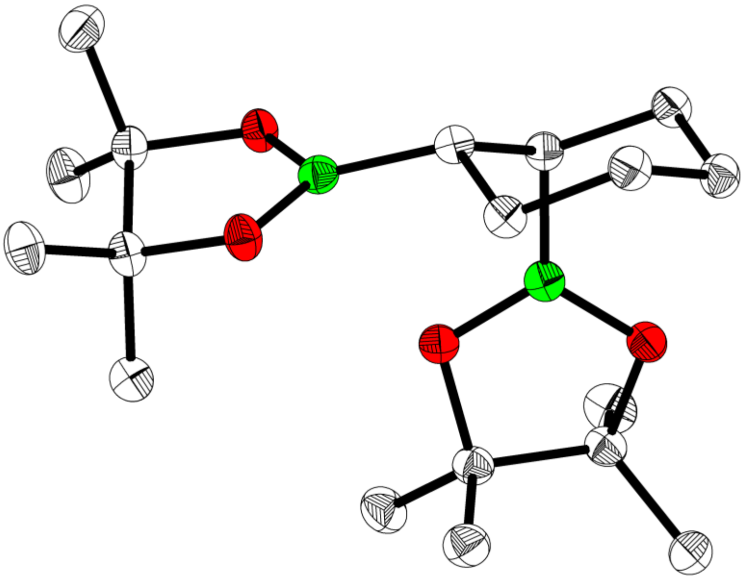

Figure S4. The solid-state molecular structure of **26b** determined by single-crystal X-ray diffraction at 100 K. All ellipsoids are drawn at the 50% probability level, and H atoms are

omitted for clarity. As the crystal structure is centrosymmetric, both enantiomers are present. Atom colors: carbon (white), boron (green), oxygen (red).

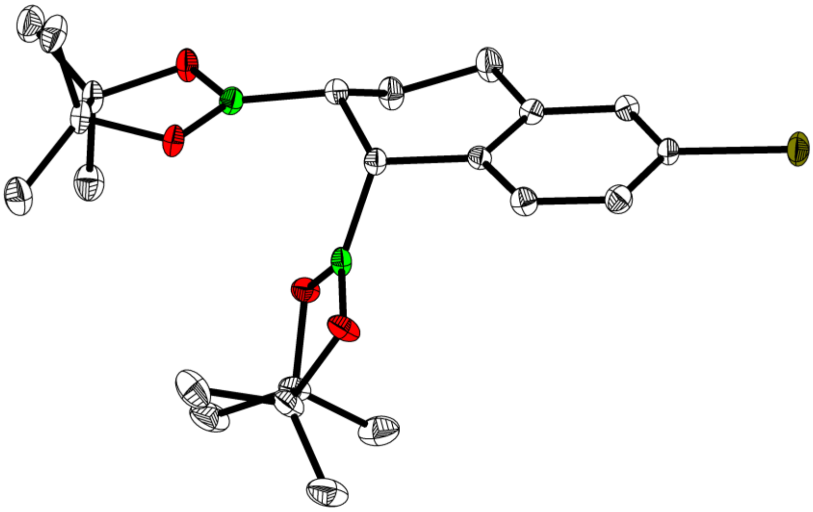

Figure S5. The solid-state molecular structure of **28b** determined by single-crystal X-ray diffraction at 100 K. All ellipsoids are drawn at the 50% probability level, and H atoms are omitted for clarity. Two symmetry-independent molecules are present in the unit cell (space group $P2_1$) and they are enantiomers. Atom colors: carbon (white), boron (green), oxygen (red).

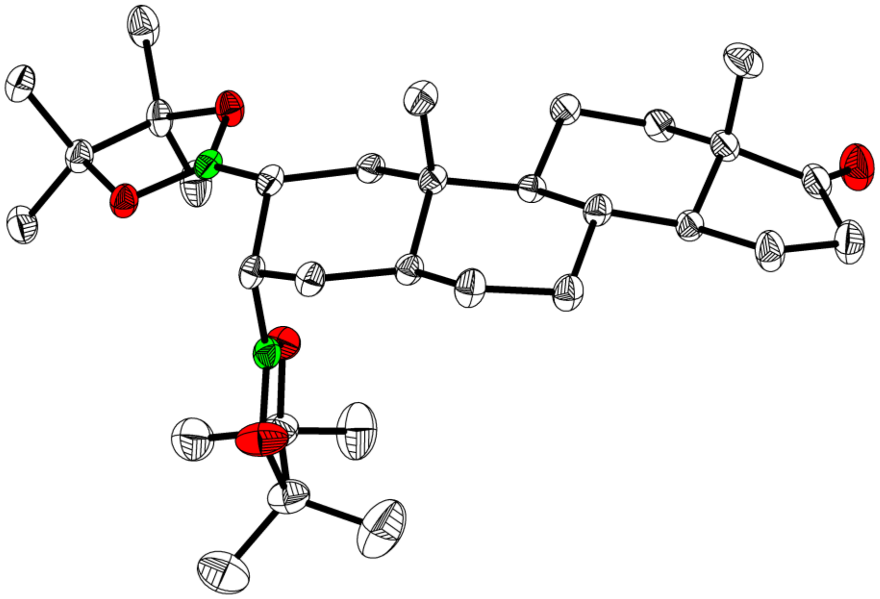

Figure S6. The solid-state molecular structure of **59b** determined by single-crystal X-ray diffraction at 100 K. All ellipsoids are drawn at the 50% probability level, and H atoms are

omitted for clarity. One of the Bpin groups is disordered and only the major part (73%) is shown here. The crystal structure is non-centrosymmetric (space group $P2_1$) and only a single enantiomer is present. Atom colors: carbon (white), boron (green), oxygen (red).

4 References

[1] J. E. R. Pena, E. J. Alexanian, *Org. Lett.* **2017**, *19*, 4413−4415.

[2] T. Taguri, M. Yamamoto, T. Fujii, Y. Muraki, T. Ando, *Eur. J. Org. Chem.* **2013**, 6924−6933.

[3] W. Liu, H. Li, H. Qin, W. Zhao, C. Zhou, S. Jiang, C. Yang, *Chem. Res. Chin. Univ.* **2017**, *33*, 213−220.

[4] V. Sepe, B. Renga, C. Festa, C. FInamore, D. Masullo, A. Carino, S. Cipriani, E. Distrutti, S. Fiorucci, A. Zampella, *Steroids* **2016**, *105*, 59−67.

[5] D. D. Bume, C. R. Pitts, F. Ghorbani, S. A. Harry, J. N. Capilato, M. A. Siegler, T. Lectka, *Chem. Sci.* **2017**, *8*, 6918−6923.

[6] X.-Y. Dong, Y.-F. Zhang, C.-L. Ma, Q.-S. Gu, F.-L. Wang, Z.-L. Li, S.-P. Jiang, X.-Y. Liu, *Nat. Chem.* **2019**, *11*, 1158−1166.

[7] C. Li, Y. Zhang, Q. Sun, T. Gu, H. Peng, W. Tang, *J. Am. Chem. Soc.* **2016**, *138*, 10774−10777.

[8] J. Holz, C. Pfeffer, H. Zuo, D. Beierlein, G. Richter, E. Klemm, R. Peters, *Angew. Chem. Int. Ed.* **2019**, *58*, 10330−10334; *Angew. Chem.* **2019**, *131*, 10437−10442.

[9] T. Hou, P. Lu, P. Li, *Tetrahedron Lett.* **2016**, *57*, 2273−2276.

[10] X.-L. Su, L. Ye, J.-J. Chen, X.-D. Liu, S.-P. Jiang, F.-L. Wang, L. Liu, C.-J. Yang, X.-Y. Chang, Z.-L. Li, Q.-S. Gu, X.-Y. Liu, *Angew. Chem. Int. Ed.* **2021**, *60*, 380−384; *Angew. Chem.* **20121**, *133*, 384−388.

[11] a) P. H. Huy, I. Filbrich, *Chem. Eur. J.* **2018**, *24*, 7410−7416; b) A. Xia, X. Xie, H. Chen, J. Zhao, C. Zhang, Y. Liu, *Org. Lett.* **2018**, *20*, 7735−7739.

[12] Y. Yoshida, K. Shimonishi, Y. Sakakura, S. Okada, N. Aso, Y. Tanabe, *Synthesis* **1999**, *9*, 1633−1636.

[13] X. Wang, S. Wang, W. Xue, H. Gong, *J. Am. Chem. Soc.* **2015**, *137*, 11562−11565.

[14] Y. Yamamoto, H. Hasegawa, H. Yamataka, *J. Org. Chem.* **2011**, *76*, 4652−4660.

[15] F. Xu, X.-Y. Qian, Y.-J. Li, H.-C. Xu, *Org. Lett.* **2017**, *19*, 6332−6335.

[16] B. V. Ramulu, L. Mahendar, G. Satyanarayana, *Asian J. Org. Chem.* **2016**, *5*, 207−212.

[17] C. Berini, O. Navarro, *Chem. Commun.* **2012**, *48*, 1538−1540.

[18] M. O. Ganiu, A. H. Cleveland, J. L. Paul, R. Kartika, *Org. Lett.* **2019**, *21*, 5611−5615.

[19] Y. Zhang, X. Zhao, C. Bi, W. Lu, M. Song, D. Wang, G. Qing, *Green Chem.* **2021**, *23*, 1691−1699.

[20] A. Fawcett, D. Nitsch, M. Ali, J. M. Bateman, E. L. Myers, V. K. Aggarwal, *Angew. Chem. Int. Ed.* **2016**, *55*, 14663−14667; *Angew. Chem.* **2016**, *128*, 14883−14887
[21] F. Alonso, Y. Moglie, L. Pastor-Pérez, A. Sepúlveda-Escribano, *ChemCatChem* **2014**, *6*, 857−865.
[22] A. Bonet, C. Sole, H. Gulyás, E. Fernández, *Org. Biomol. Chem.* **2012**, *10*, 6621−6623.
[23] G. Gao, J. Yan, K. Yang, F. Chen, Q. Song, *Green Chem.* **2017**, *19*, 3997−4001.
[24] C. Pubill-Ulldemolins, M. Poyatos, C. Bo, E. Fernández, *Dalton Trans.* **2013**, *42*, 746−752.
[25] X.-X. Wang, L. Li, T. J. Gong, B. Xiao, X. Lu, Y. Fu, *Org. Lett.* **2019**, *21*, 4298−4302.
[26] L. Yan, J. P. Morken, *Org. Lett.* **2019**, *21*, 3760−3763.
[27] J. Hu, Y. Zhao, Z. Shi, *Nat. Catal.* **2018**, *1*, 860−869.
[28] D. J. Blair, D. Tanini, J. M. Bateman, H. K. Scott, E. L. Myers, V. K. Aggarwal, *Chem. Sci.* **2017**, *8*, 2898−2903.
[29] Y. Lou, J. Qiu, K. Yang, F. Zhang, C. Wang, Q. Song, *Org. Lett.* **2021**, *23*, 4564−4569.
[30] E. Andre-Joyaux, A. Kuzovlev, N. D. C. Tappin, P. Renaud, *Angew. Chem. Int. Ed.* **2020**, *59*, 13859−13864; *Angew. Chem.* **2020**, *132*, 13963−13968.
[31] G. K. Dewkar, S. V. Narina, A. Sudalai, *Org. Lett.* **2003**, *5*, 4501−4504.
[32] K. Yang, Q. Song, *Green Chem.* **2016**, *18*, 932−936.
[33] S. Das, T. Asefa, *ACS Catal.* **2011**, *1*, 502−510.
[34] S. Shee, D. Panja, S. Kundu, *J. Org. Chem.* **2020**, *85*, 2775−2784.
[35] S. P. Miller, J. B. Morgan, F. J. Nepveux V, J. P. Morken, *Org. Lett.* **2004**, *6*, 131−133.
[36] C. M. Crudden, C. Ziebenhaus, J. P. G. Rygus, K. Ghozati, P. J. Unsworth, M. Nambo, S. Voth, M. Hutchinson, V. S. Laberge, Y. Maekawa, D. Imao, *Nat. Commun.* **2016**, *7*, 11065−11071
[37] S. Willems, G. Toupalas, J. C. Reisenbauer, B. Morandi, *Chem. Commun.* **2021**, *57*, 3909−3912.
[38] Z. Lu, Q. Zheng, S. Yang, C. Qian, Y. Shen, T. Tu, *ACS Catal.* **2021**, *11*, 10796−10801.
[39] G. M. Sheldrick, *Acta Crystallogr.* **2015**, *A71*, 3−8.
[40] G. M. Sheldrick, *Acta Crystallogr.* **2015**, *C71*, 3−8.
[41] C. B. Hübschle, G. M. Sheldrick, B. Dittrich, *J. Appl. Crystallogr.* **2011**, *44*, 1281−1284.
[42] K. Brandenburg, Diamond (version 4.4.0), Crystal and Molecular Structure Visualization, Crystal Impact H. Putz & K. Brandenburg GbR, Bonn (Germany), **2017**.

[43] a) F. Furche, R. Ahlrichs, C. Hättig, W. Klopper, M. Sierka, F. Weigend, *Comput. Mol. Sci.* **2014**, *4,* 91−100; b) R. Ahlrichs, M. Bär, M. Häser, H. Horn, C. Kölmel, *Chem. Phys. Lett.* **1989**, *162,* 165−169.

[44] a) M. Häser, R. Ahlrichs, *J. Comput. Chem.* **1989**, *10*, 104−111; b) O. Treutler, R. Ahlrichs, *J. Chem. Phys.* **1995**, *102,* 346−354; c) M. Arnim, R. Ahlrichs, *J. Comp. Chem.* **1998**, *19*, 1746−1757; d) F. Weigend, *Phys. Chem. Chem. Phys.* **2002**, *4*, 4285−4291; e) M. Sierka, A. Hogekamp, R. Ahlrichs, *J. Chem. Phys.* **2003**, *118*, 9136−9148.

[45] Y. Zhao, D. G. Truhlar, *Theor. Chem. Acc.* **2008**, *120*, 215−241.

[46] a) A. Schäfer, H. Horn, R. Ahlrichs, *J. Chem. Phys.* **1992**, *97*, 2571−2577; b) A. Schäfer, C. Huber, R. Ahlrichs, *J. Chem. Phys.* **1994**, *100,* 5829−5835; c) K. Eichkorn, O. Treutler, H. Öhm, M. Häser, R. Ahlrichs, *Chem. Phys. Letters* **1995**, *242*, 652−660; d) K. Eichkorn, F. Weigend, O. Treutler, R. Ahlrichs, *Theor. Chem. Acc.* **1997**, *97,* 119−124; e) F. Weigend, *Phys. Chem. Chem. Phys.* **2006**, *8,* 1057−1065; f) F. Weigend, R. Ahlrichs, *Phys. Chem. Chem. Phys.* **2005**, *7,* 3297−3305.

5 NMR Spectra

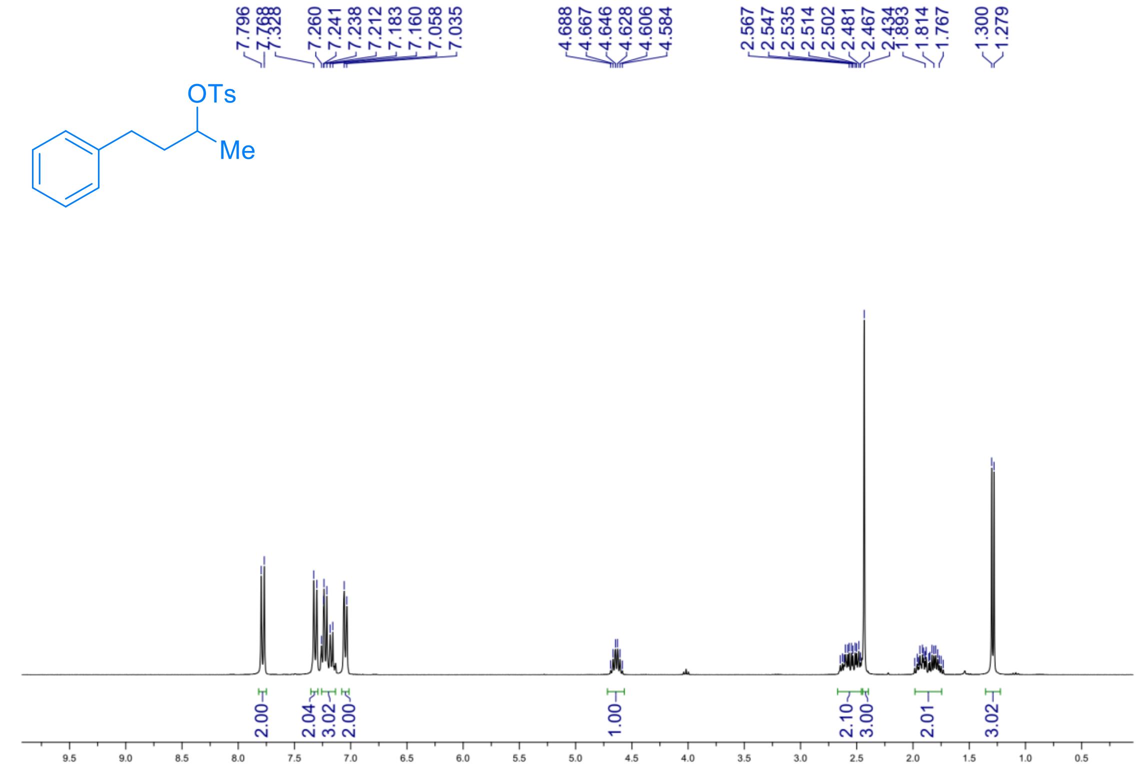

^{1}H NMR spectrum of compound **1a** in $CDCl_3$ (400 MHz).

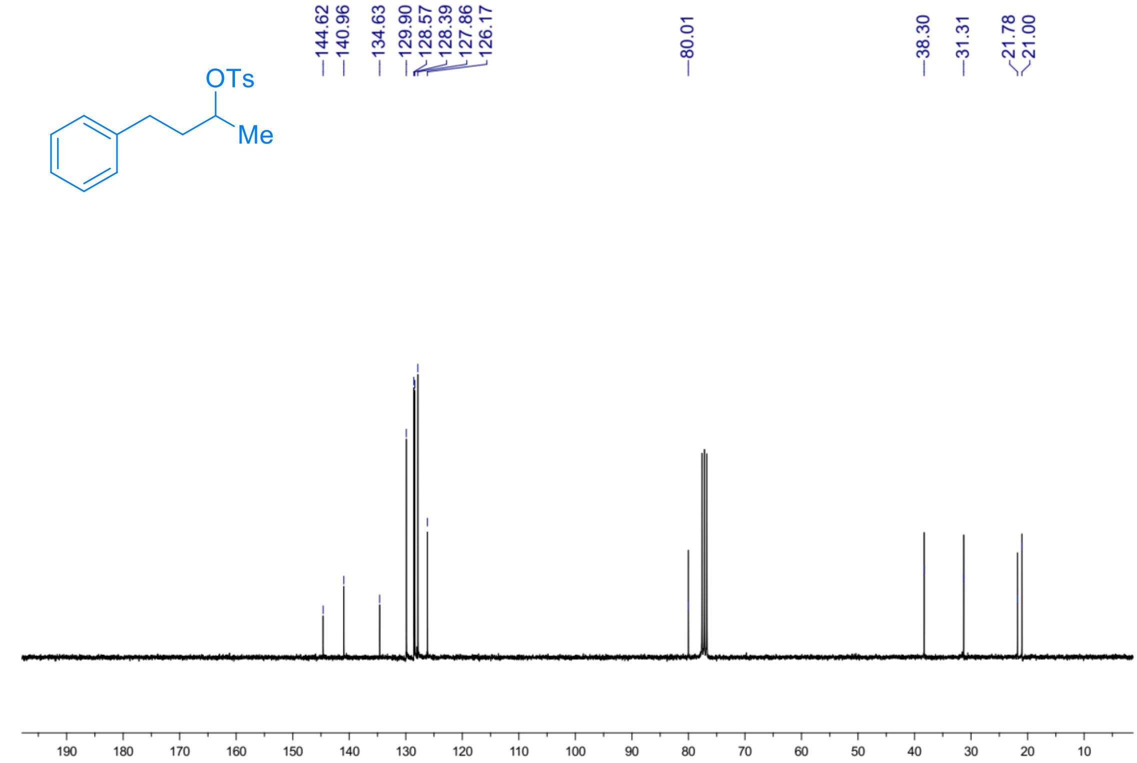

$^{13}C\{^1H\}$ NMR spectrum of compound **1a** in $CDCl_3$ (100 MHz).

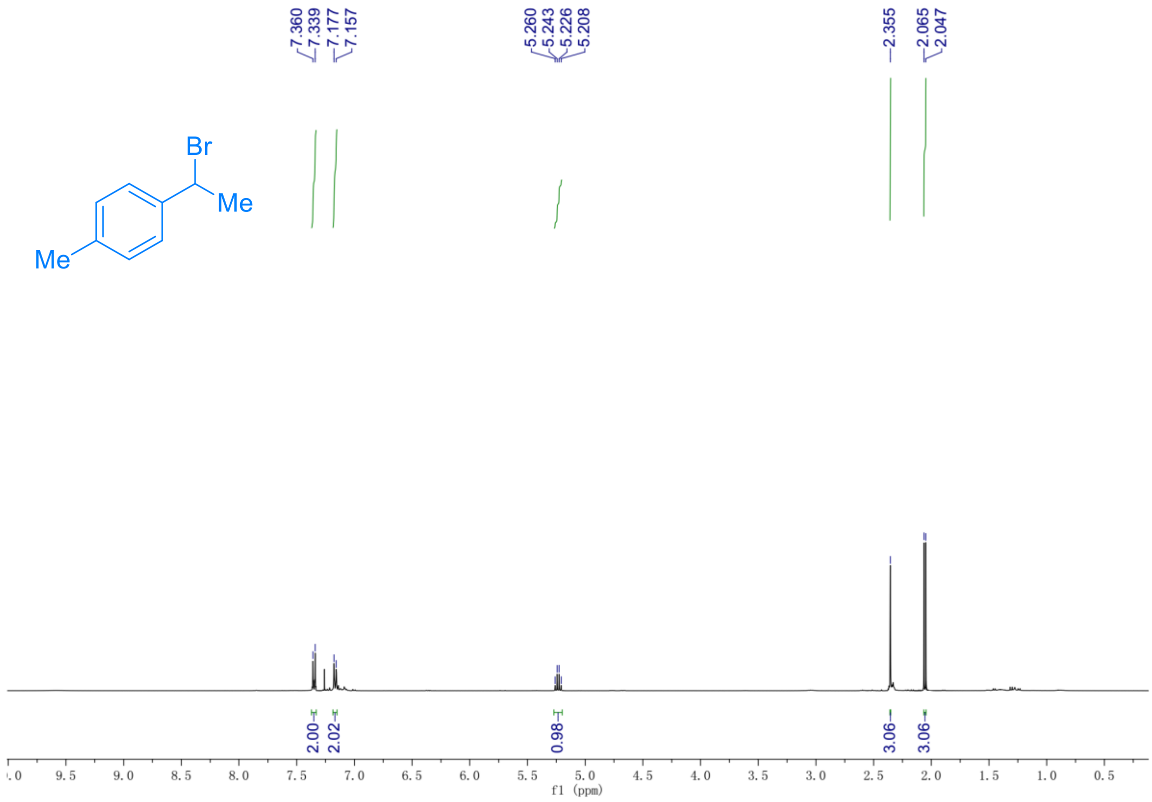

^{1}H NMR spectrum of compound **3a** in $CDCl_3$ (400 MHz).

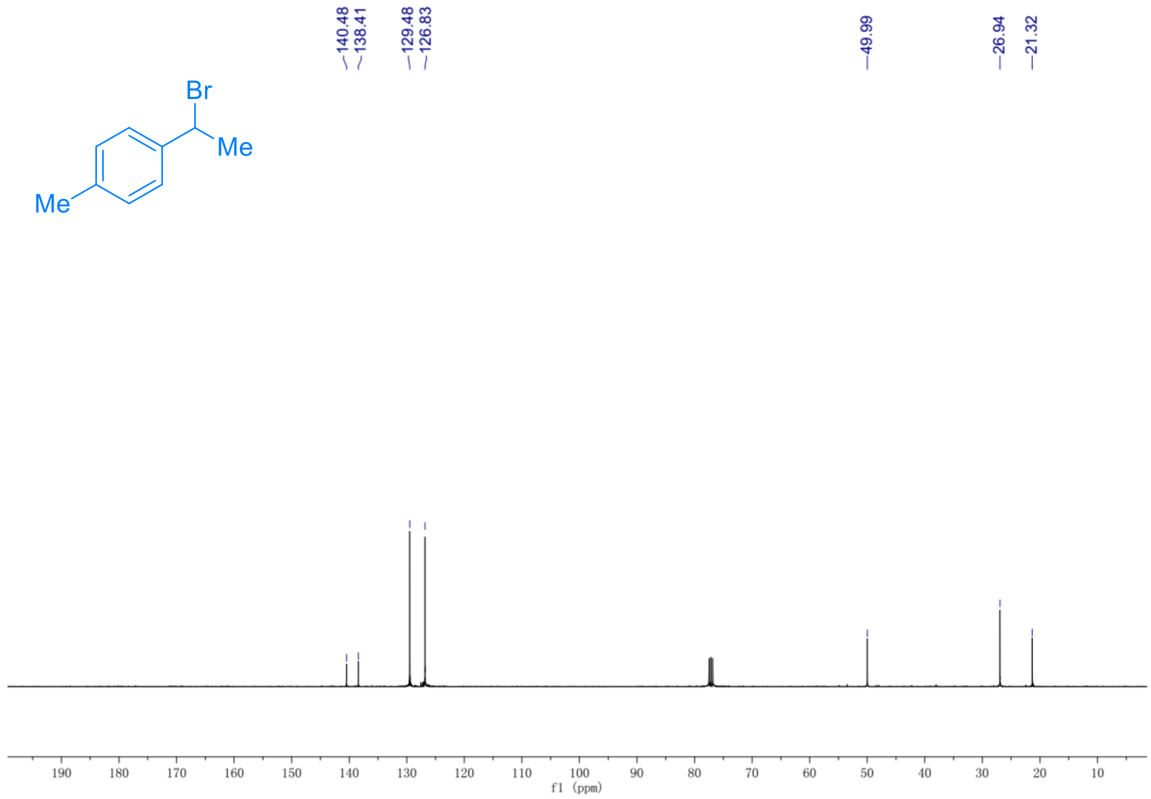

^{13}C{^{1}H} NMR spectrum of compound **3a** in $CDCl_3$ (100 MHz).

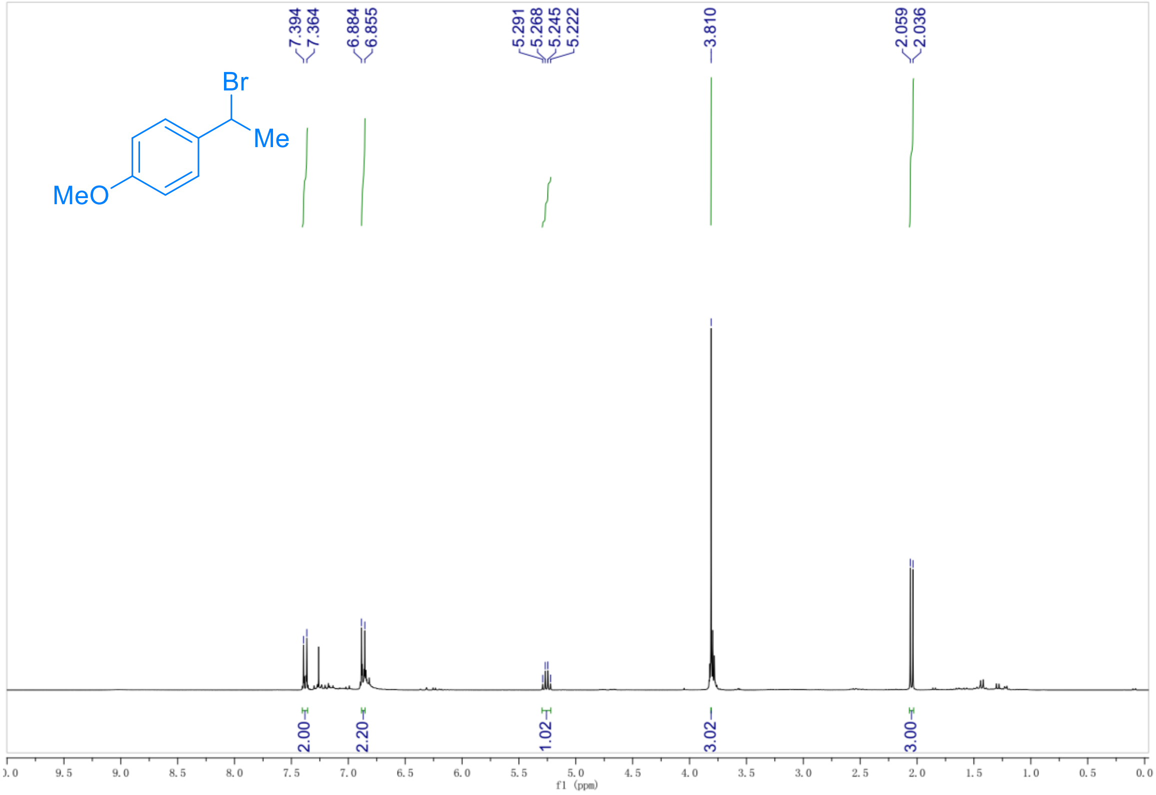

^{1}H NMR spectrum of compound **4a** in $CDCl_3$ (300 MHz).

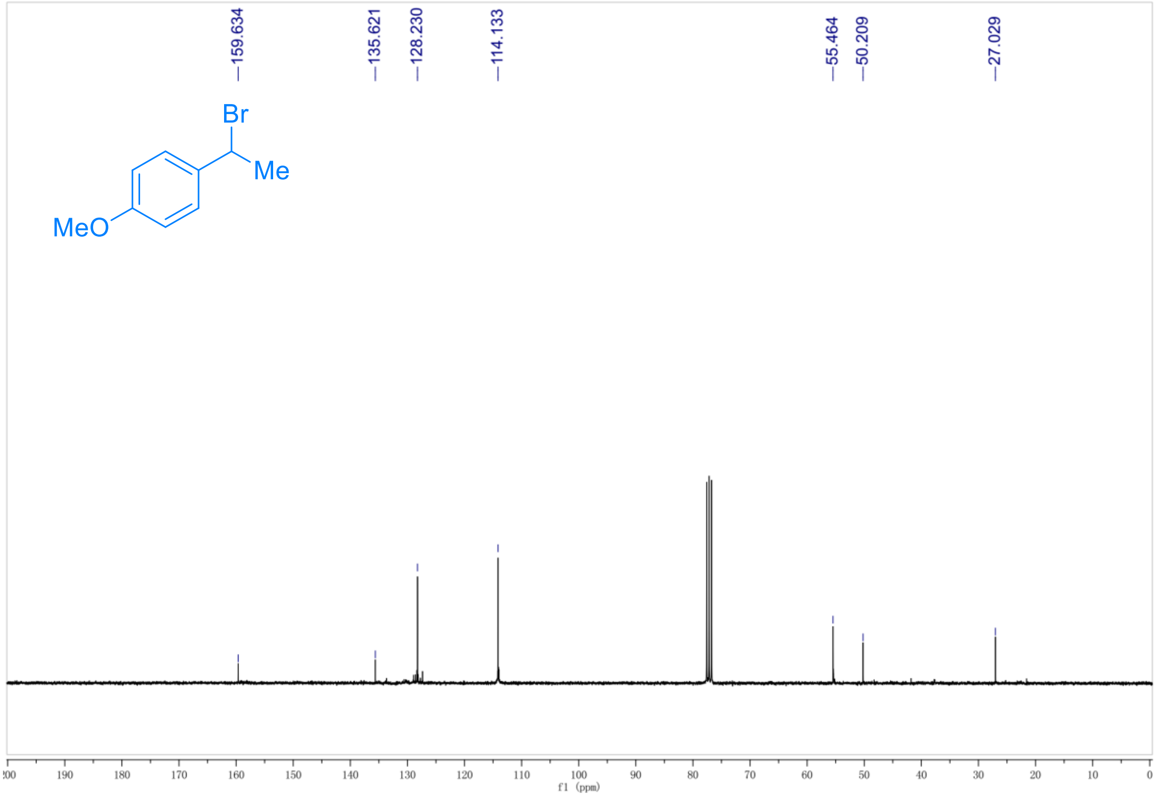

^{13}C{^{1}H} NMR spectrum of compound **4a** in $CDCl_3$ (75 MHz).

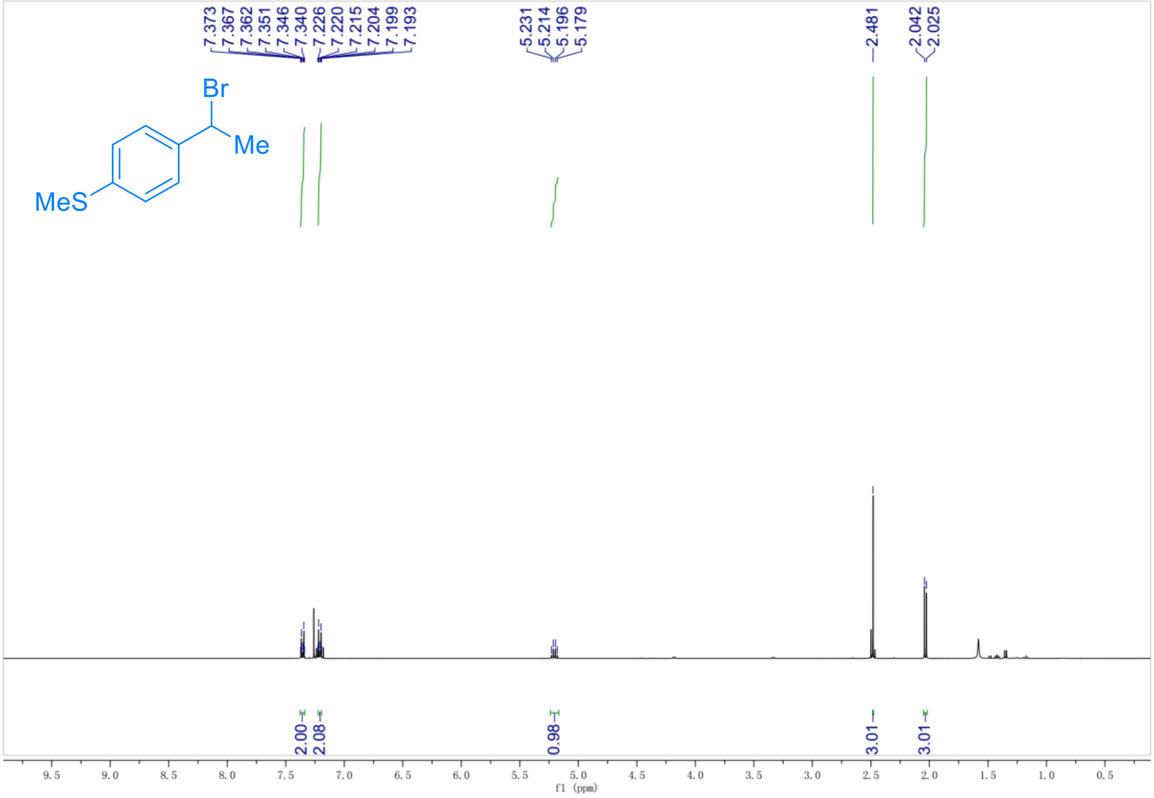

^{1}H NMR spectrum of compound **5a** in $CDCl_3$ (400 MHz).

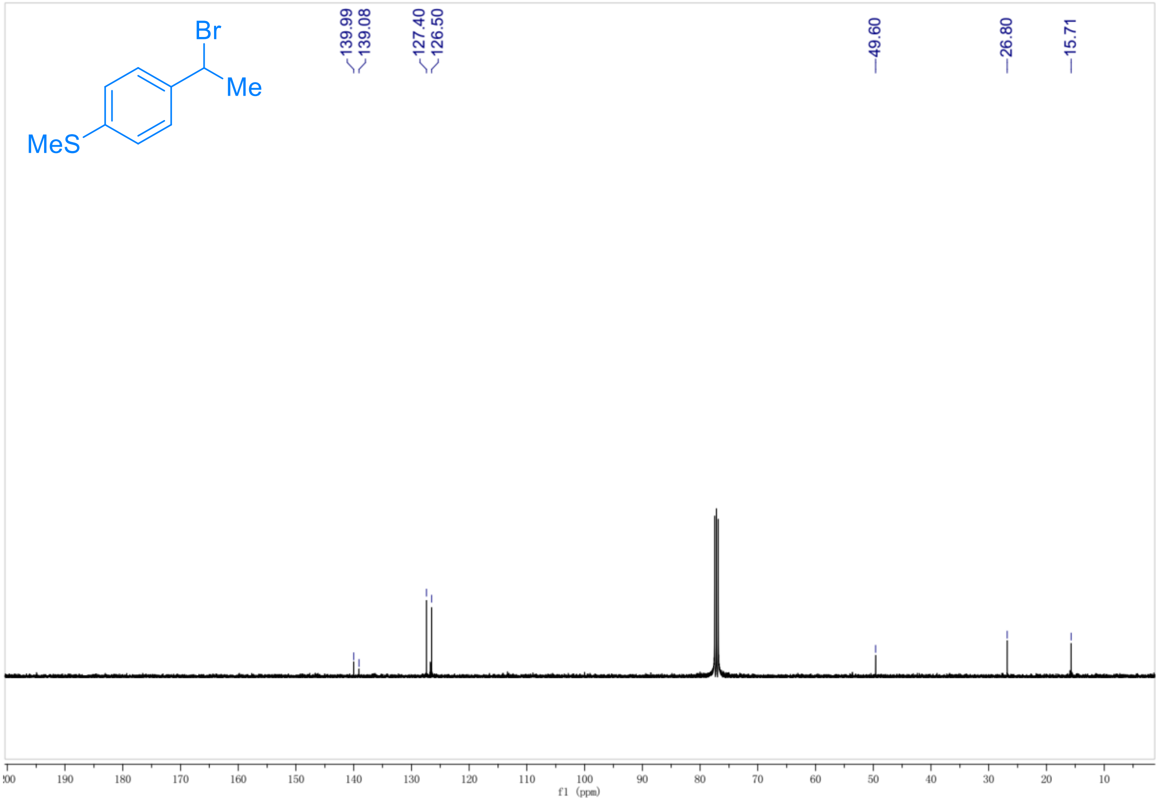

^{13}C{^{1}H} NMR spectrum of compound **5a** in $CDCl_3$ (100 MHz).

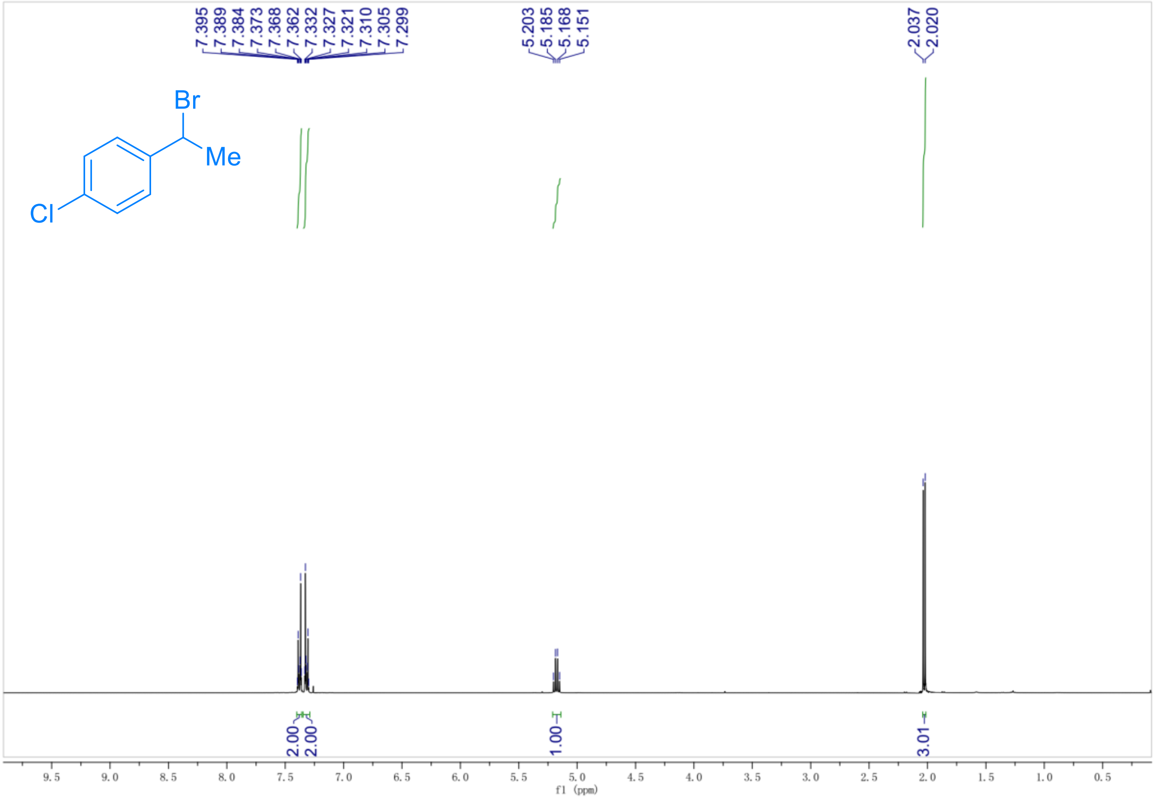

^{1}H NMR spectrum of compound **7a** in $CDCl_3$ (400 MHz).

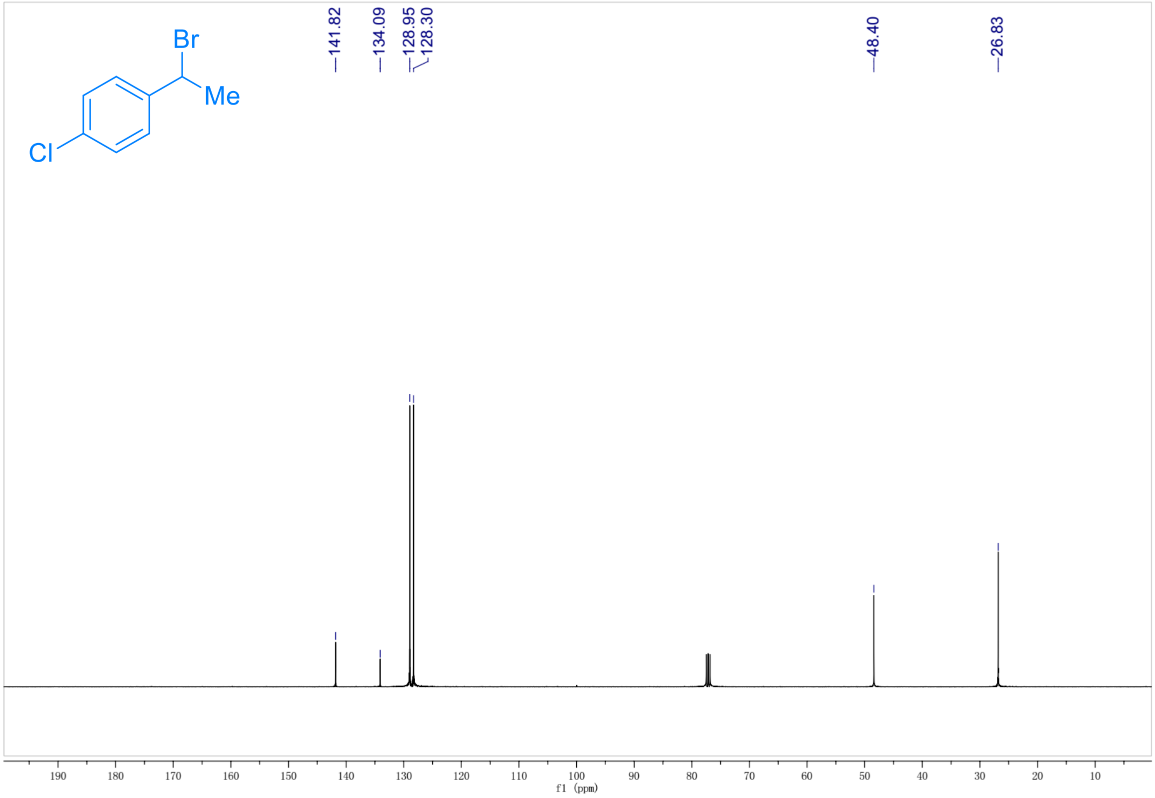

^{13}C{^{1}H} NMR spectrum of compound **7a** in $CDCl_3$ (100 MHz).

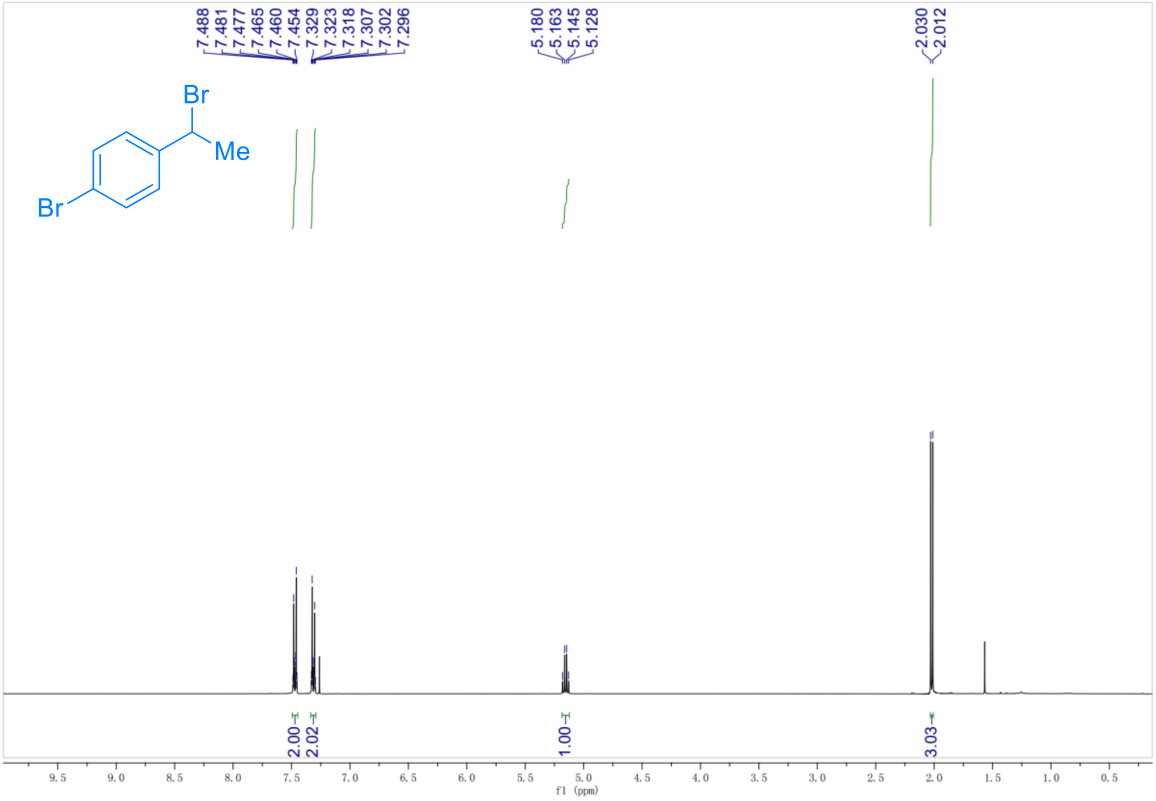

^{1}H NMR spectrum of compound **8a** in $CDCl_3$ (400 MHz).

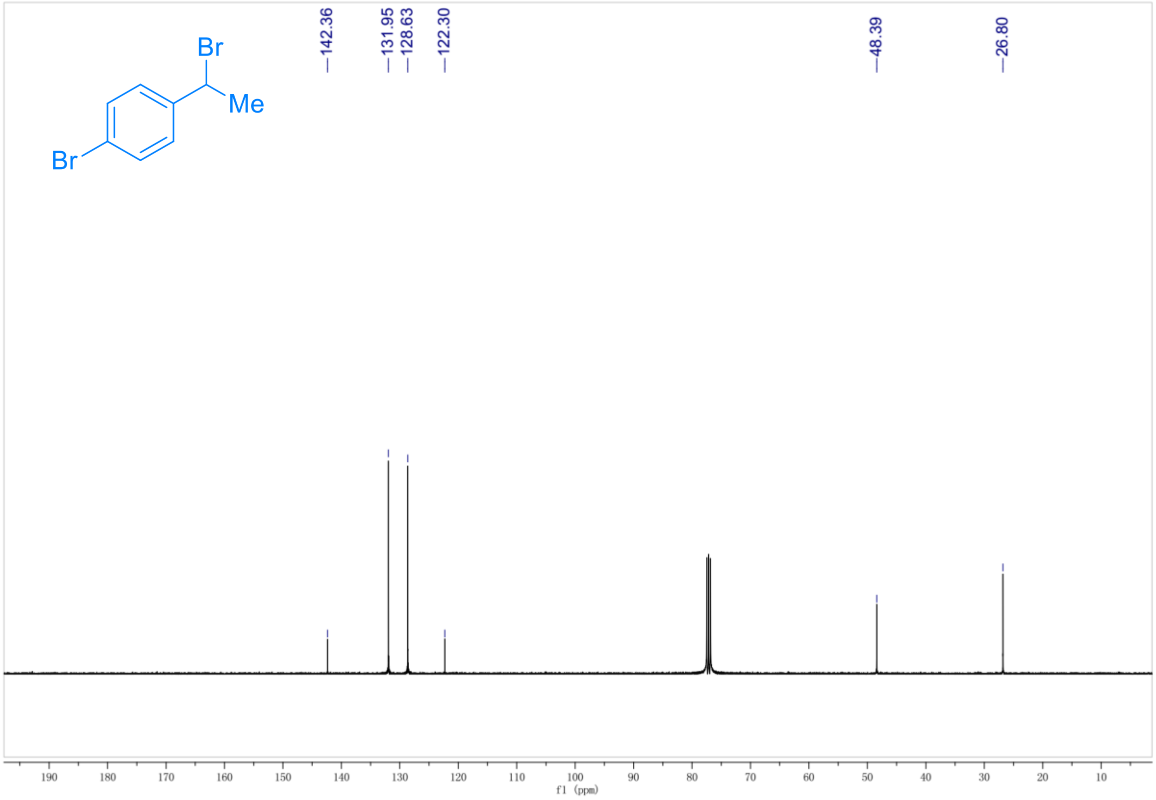

^{13}C{^{1}H} NMR spectrum of compound **8a** in $CDCl_3$ (100 MHz).

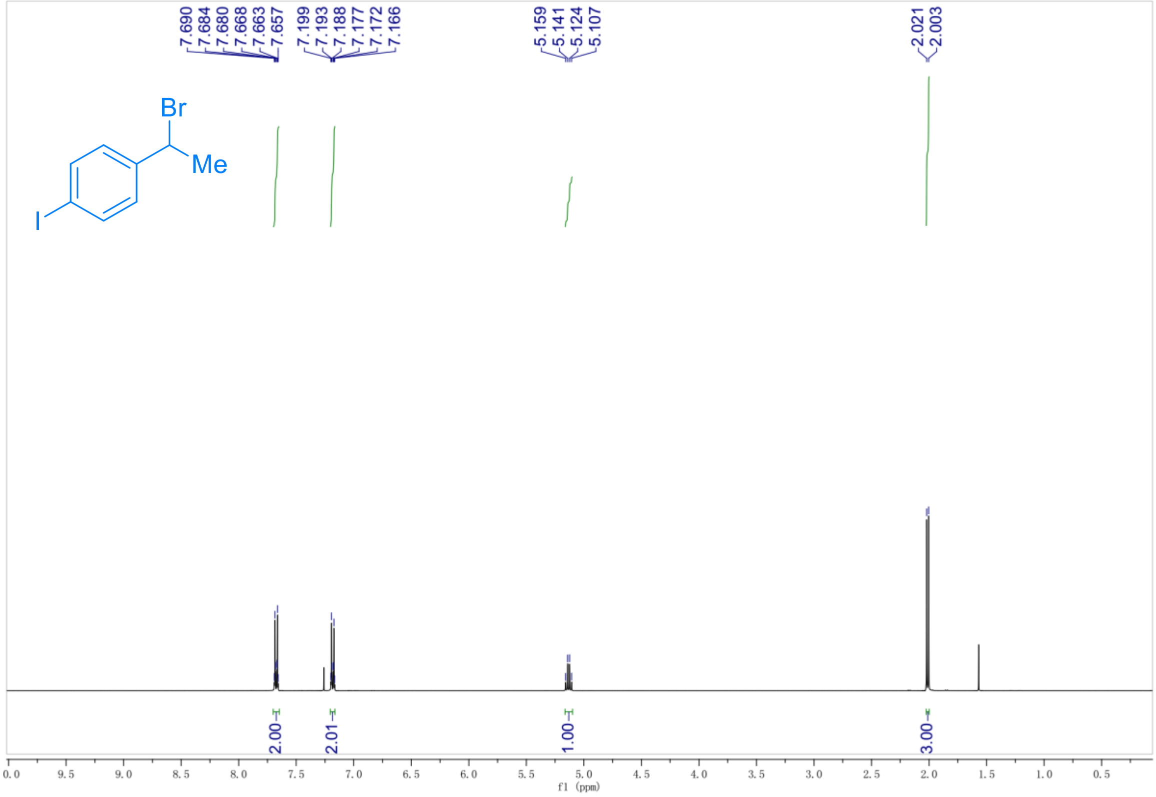

^{1}H NMR spectrum of compound **9a** in $CDCl_3$ (300 MHz).

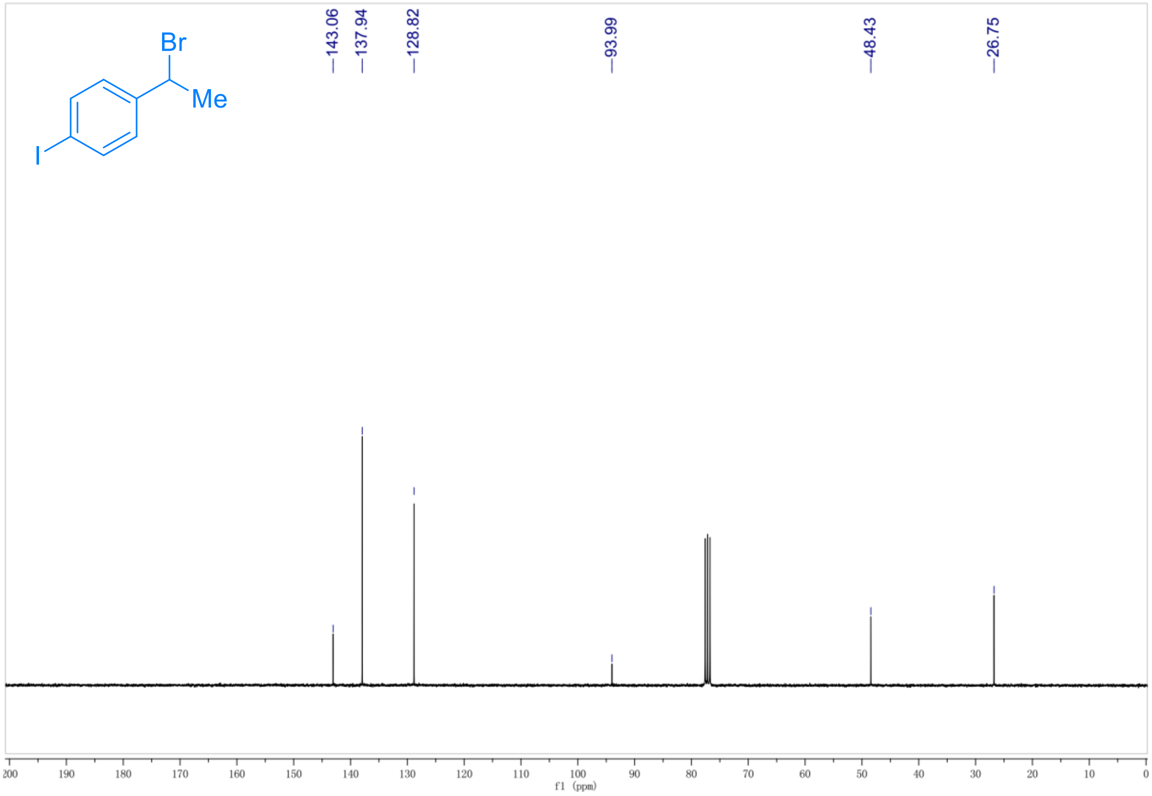

^{13}C{^{1}H} NMR spectrum of compound **9a** in $CDCl_3$ (75 MHz).

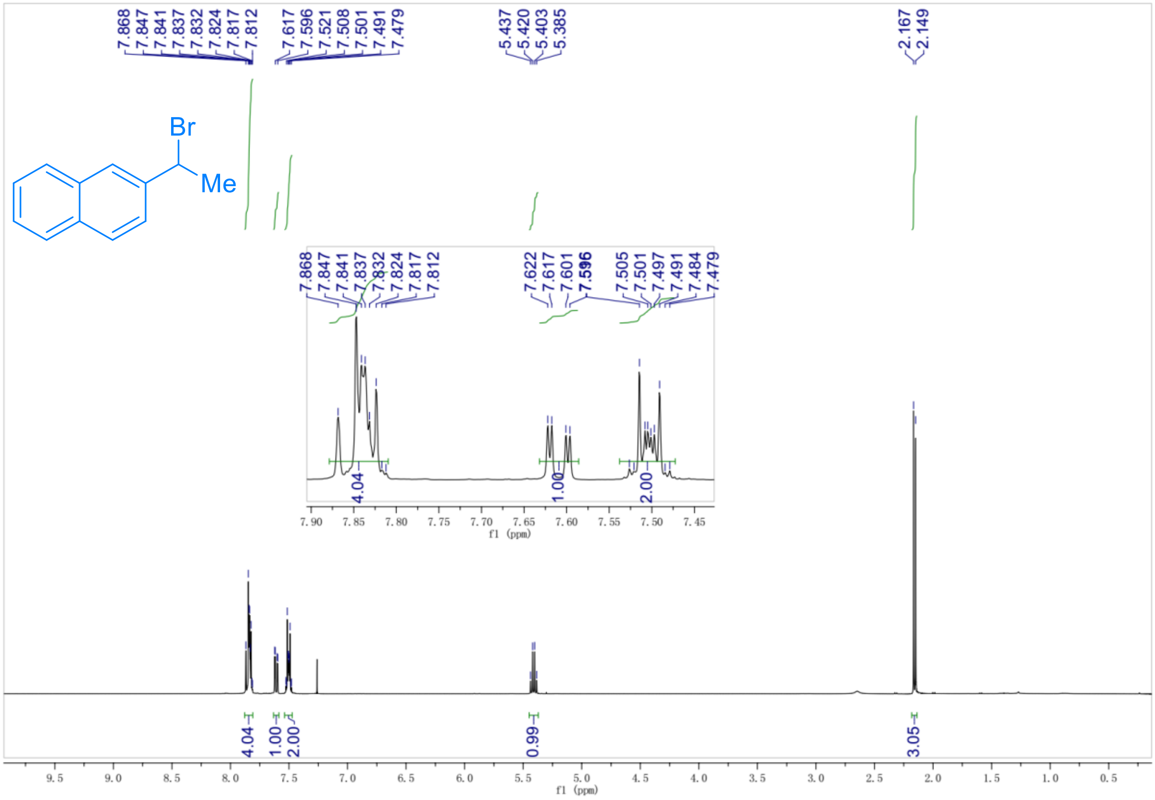

^{1}H NMR spectrum of compound **10a** in $CDCl_3$ (400 MHz).

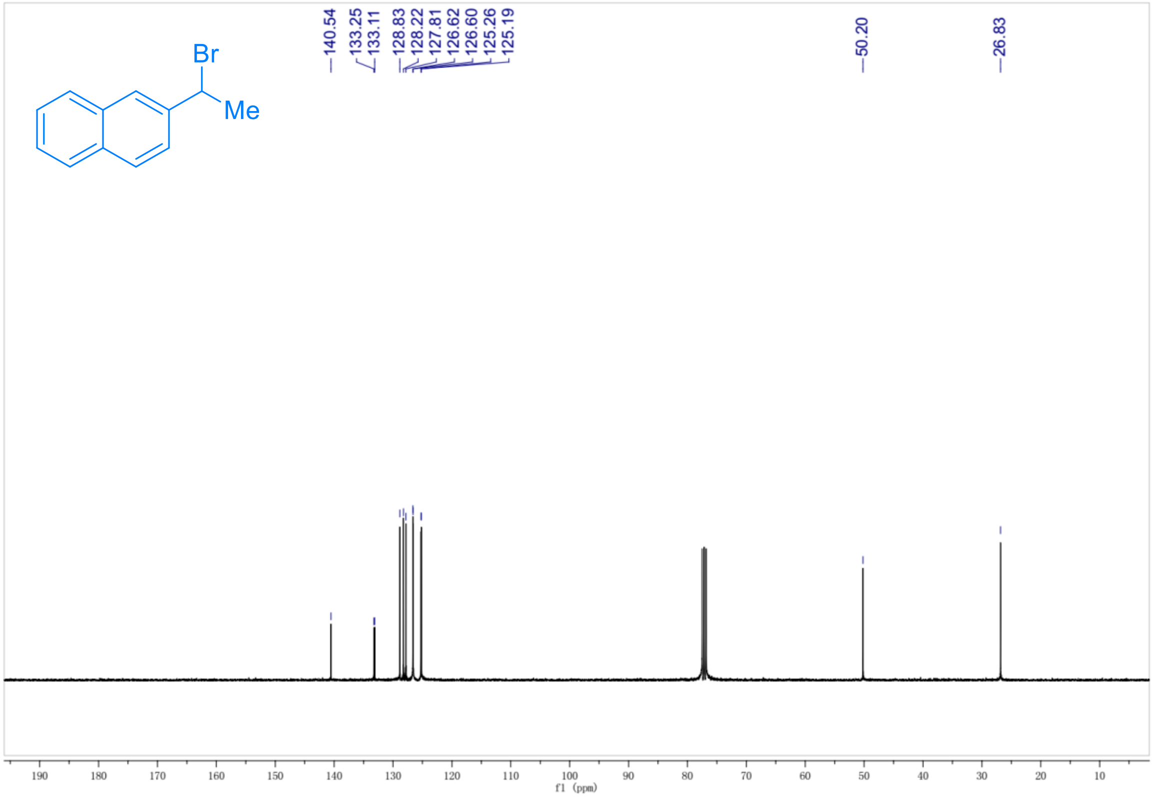

^{13}C{^{1}H} NMR spectrum of compound **10a** in $CDCl_3$ (100 MHz).

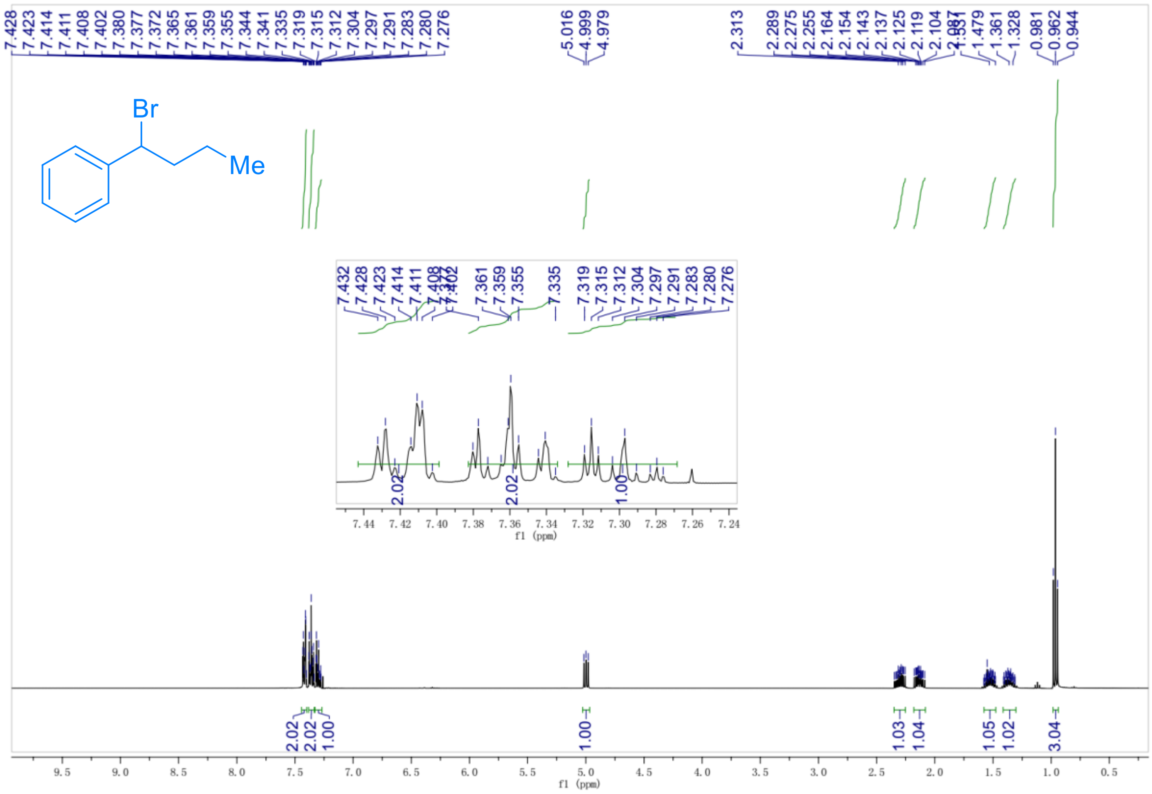

^{1}H NMR spectrum of compound **11a** in $CDCl_3$ (400 MHz).

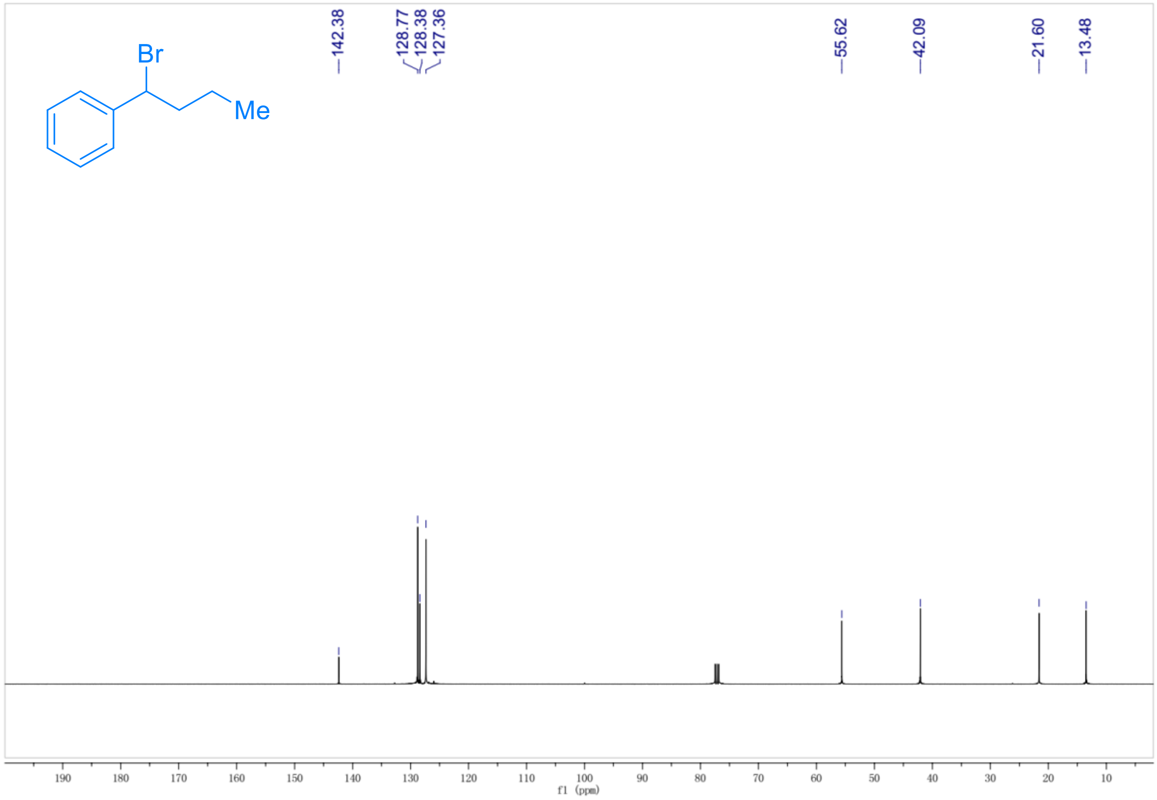

^{13}C{^{1}H} NMR spectrum of compound **11a** in $CDCl_3$ (100 MHz).

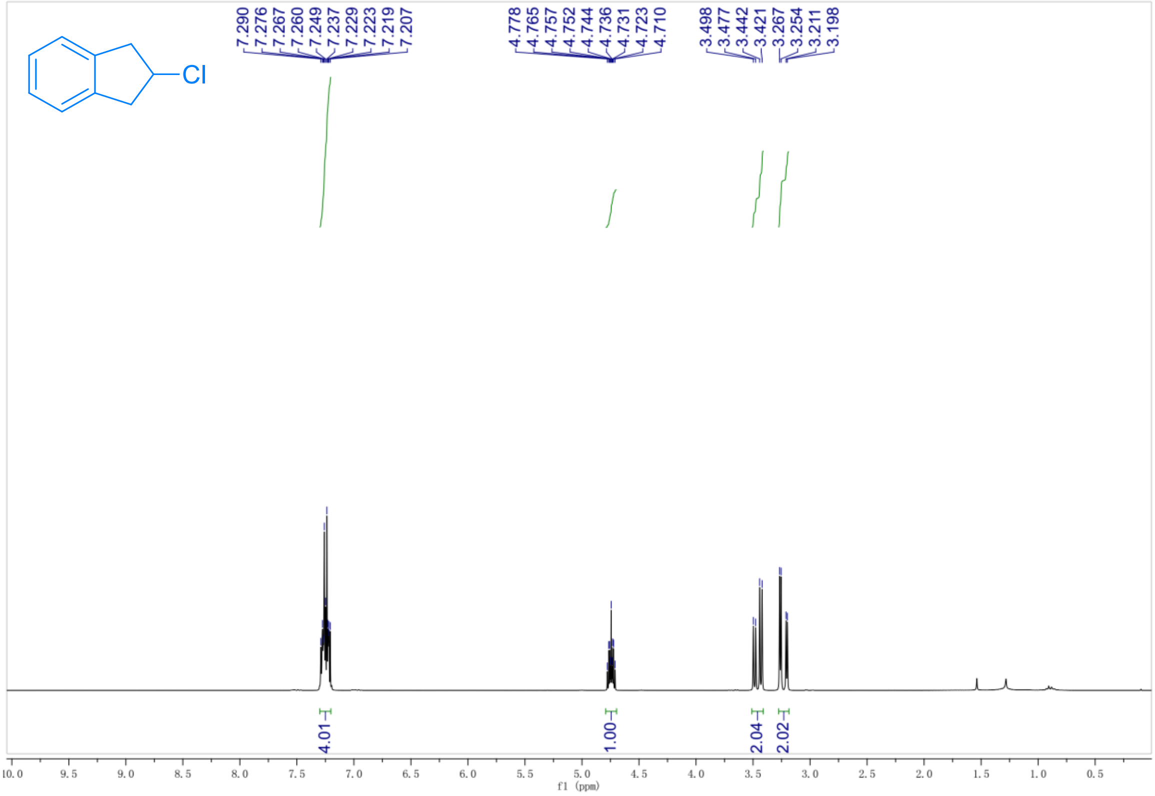

^{1}H NMR spectrum of compound **12a-1** in $CDCl_3$ (300 MHz).

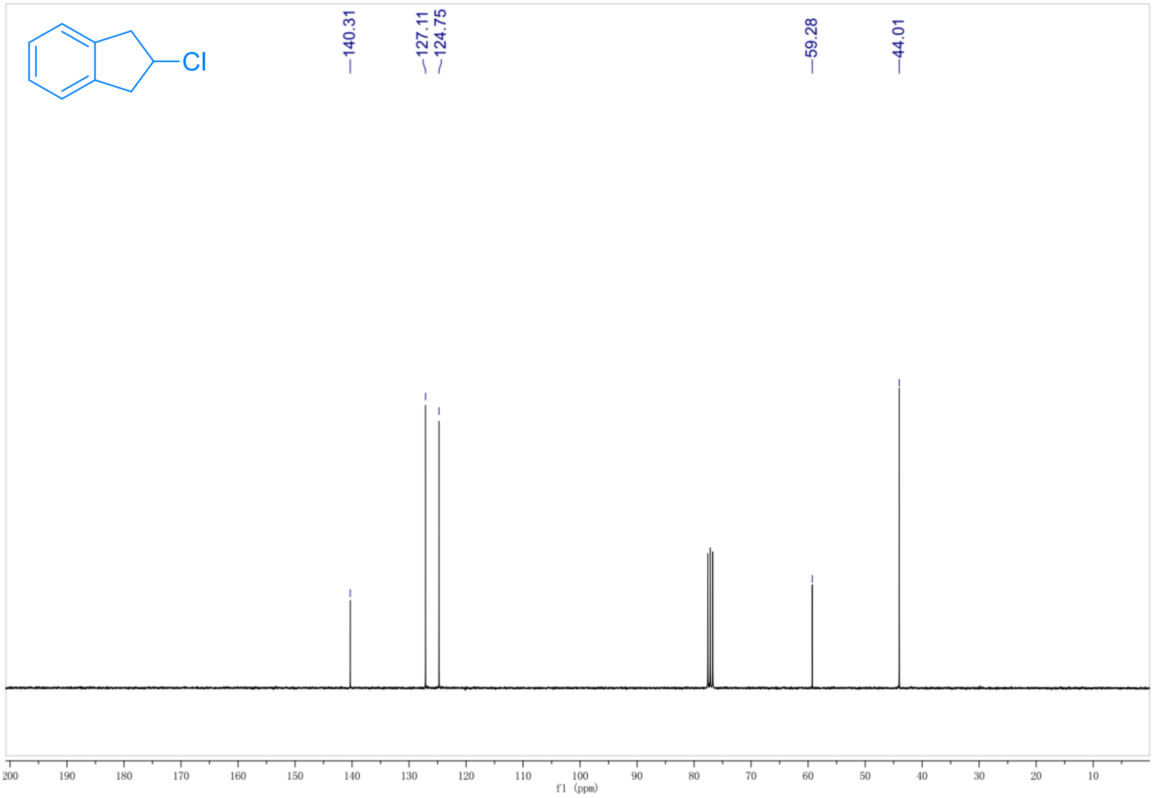

^{13}C{^{1}H} NMR spectrum of compound **12a-1** in $CDCl_3$ (75 MHz).

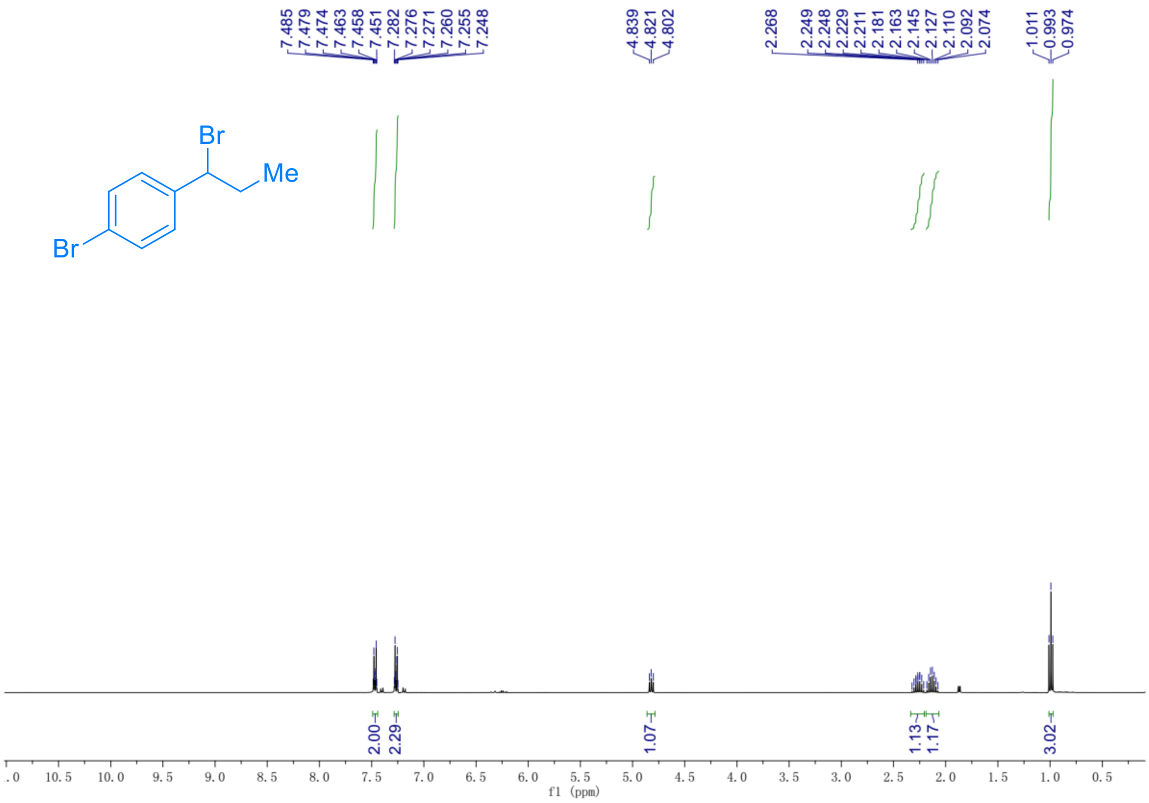

^{1}H NMR spectrum of compound **13a** in $CDCl_3$ (400 MHz).

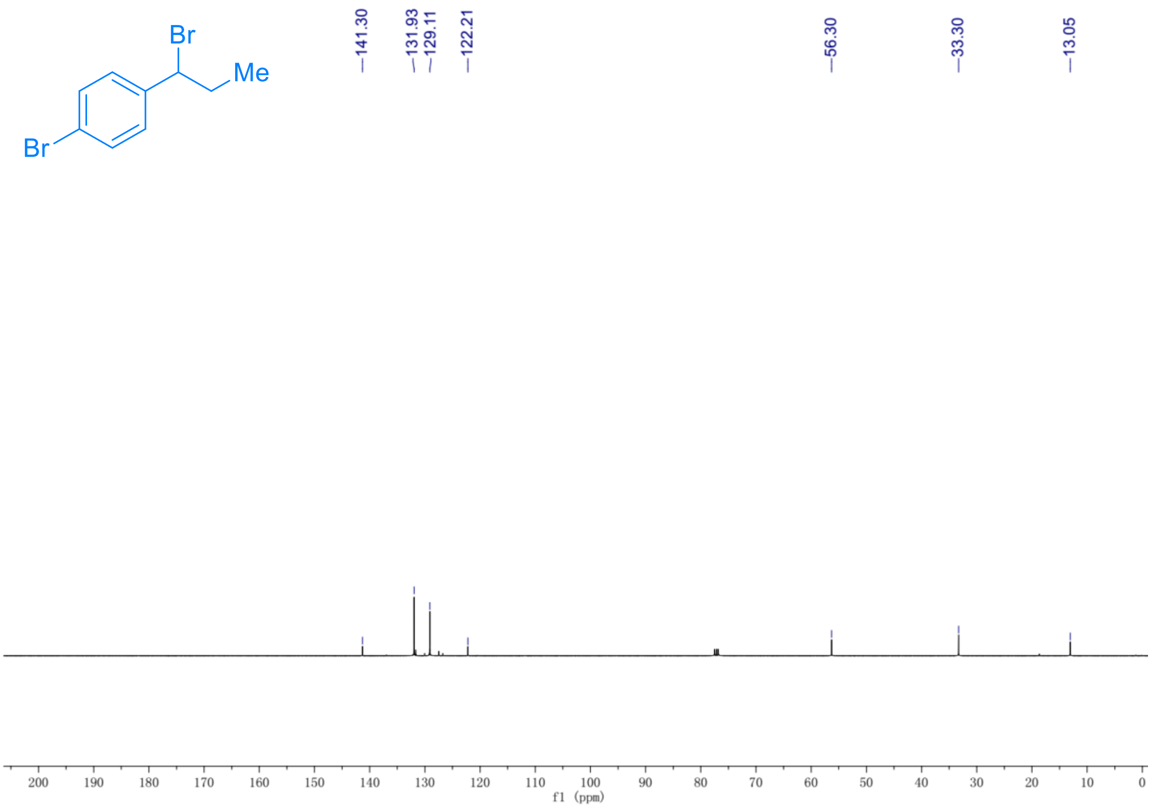

^{13}C{^{1}H} NMR spectrum of compound **13a** in $CDCl_3$ (100 MHz).

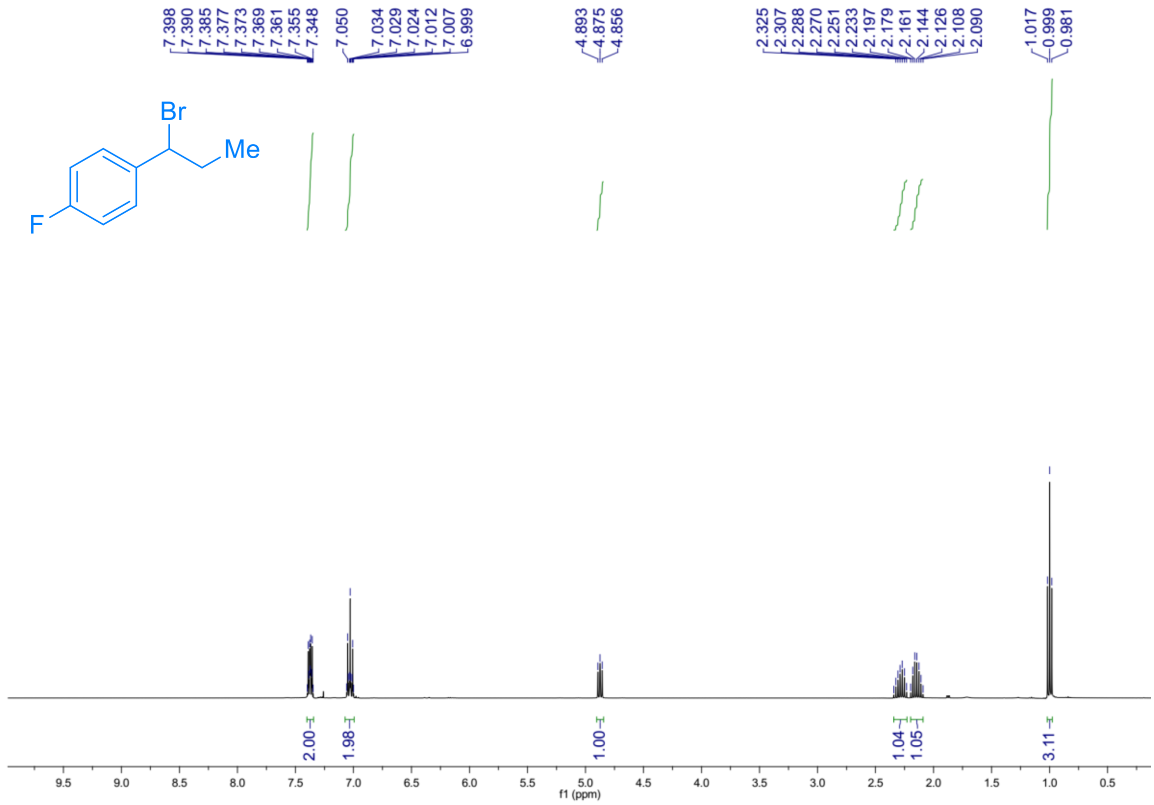

^{1}H NMR spectrum of compound **14a** in $CDCl_3$ (400 MHz).

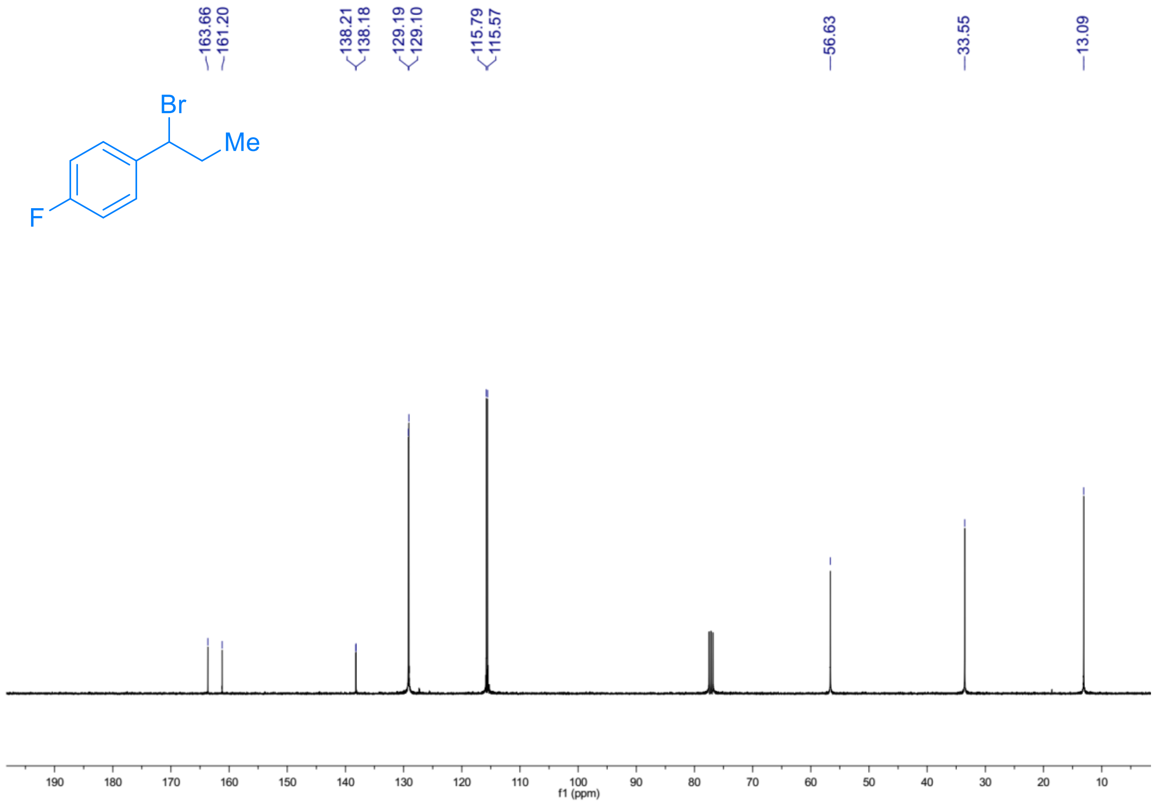

^{13}C{^{1}H} NMR spectrum of compound **14a** in $CDCl_3$ (100 MHz).

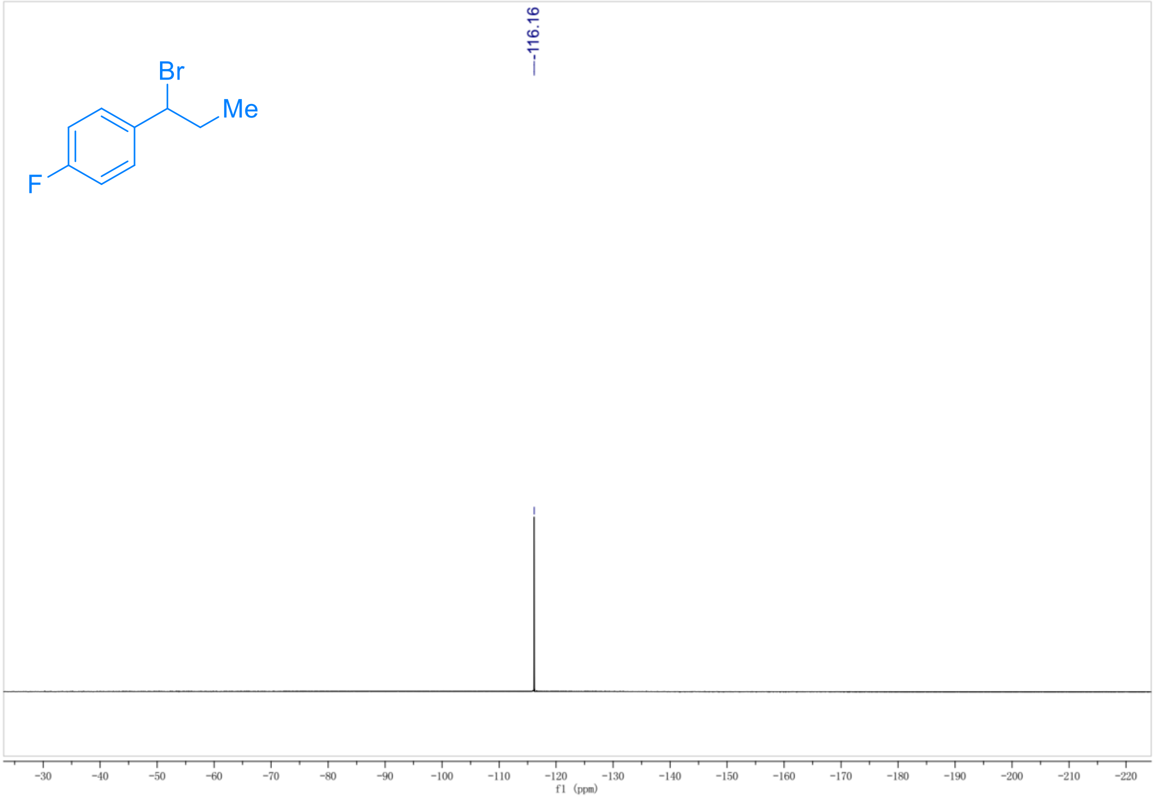

$^{19}F\{^{1}H\}$ NMR spectrum of compound **14a** in $CDCl_3$ (376 MHz).

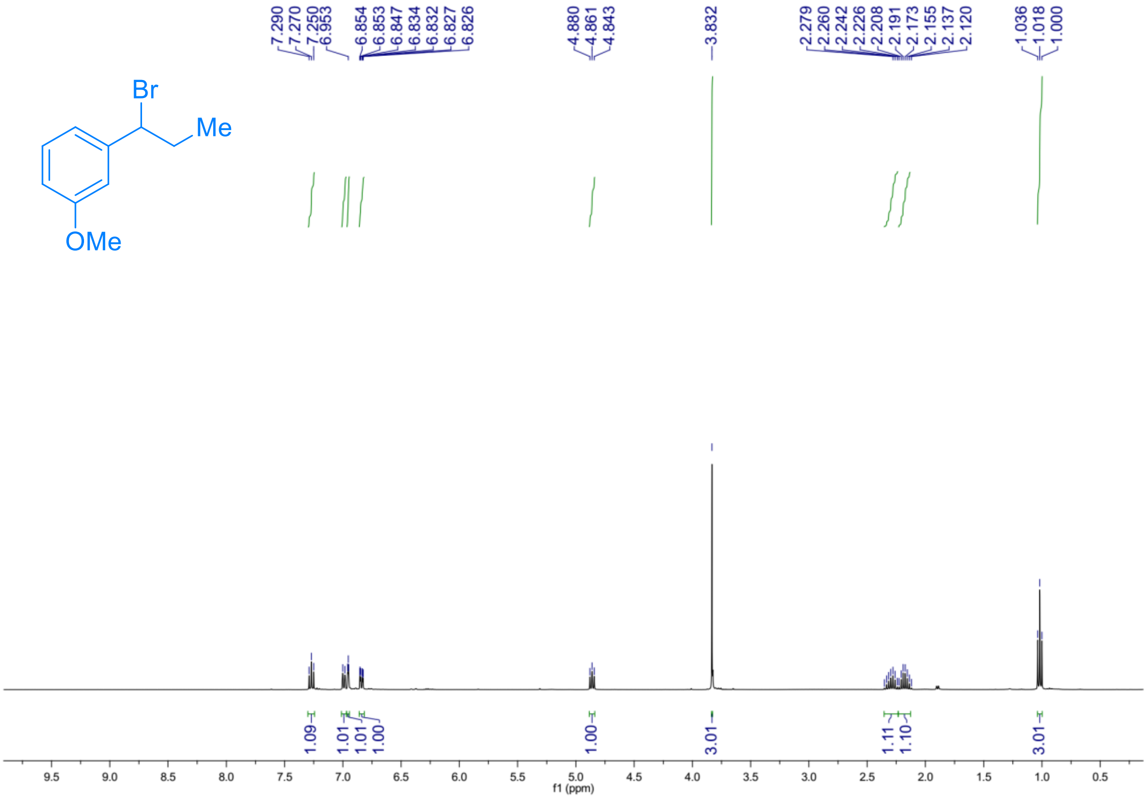

^{1}H NMR spectrum of compound **15a** in $CDCl_3$ (400 MHz).

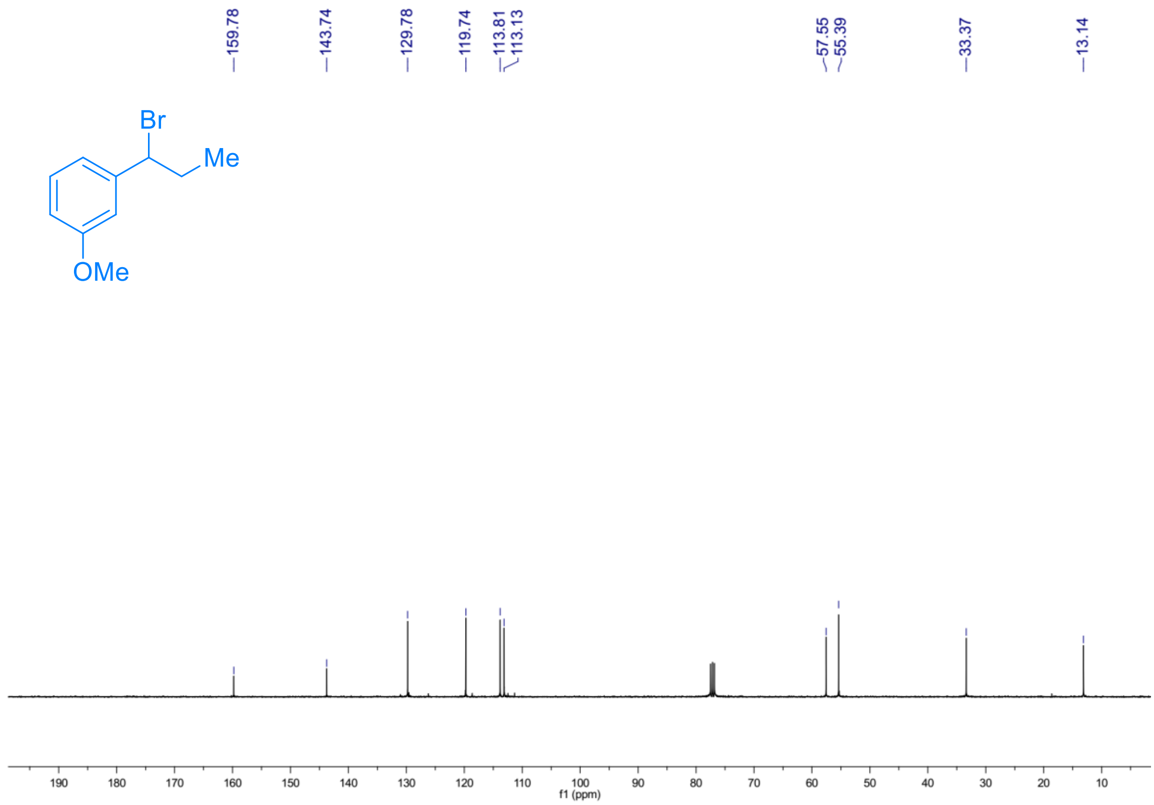

$^{13}C\{^{1}H\}$ NMR spectrum of compound **15a** in $CDCl_3$ (100 MHz).

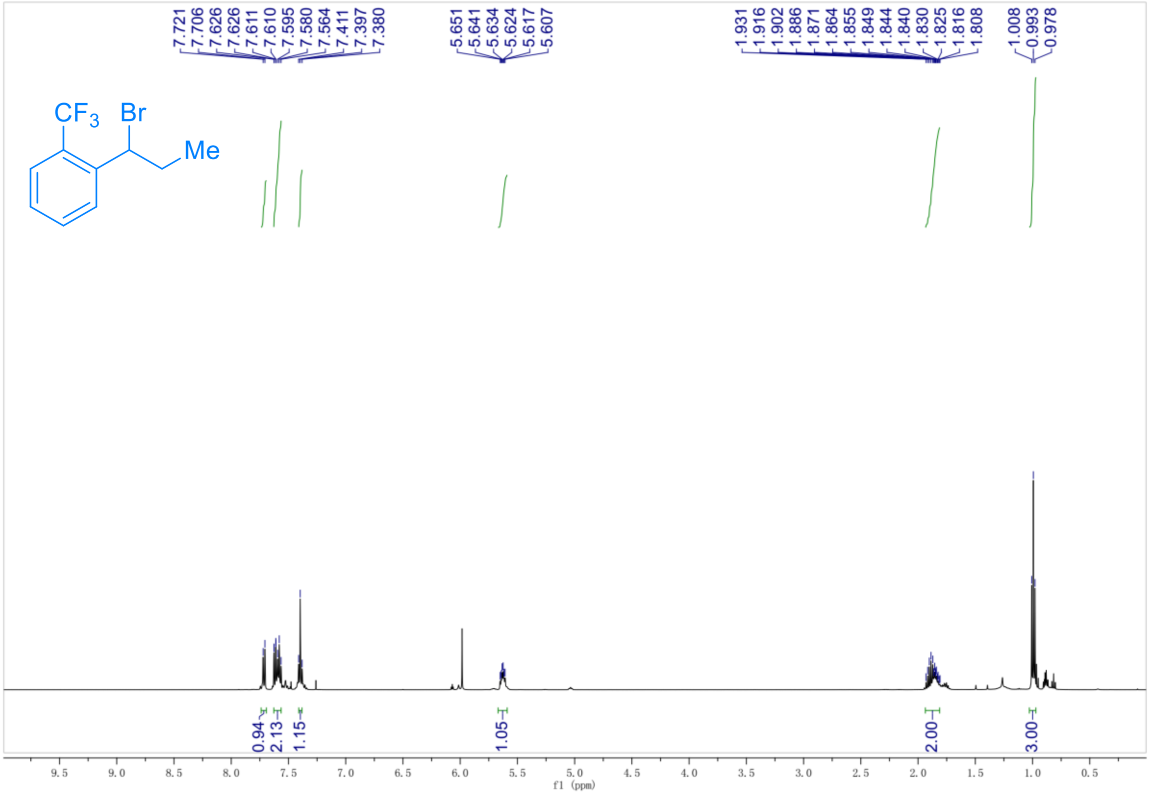

^{1}H NMR spectrum of compound **16a** in $CDCl_3$ (500 MHz).

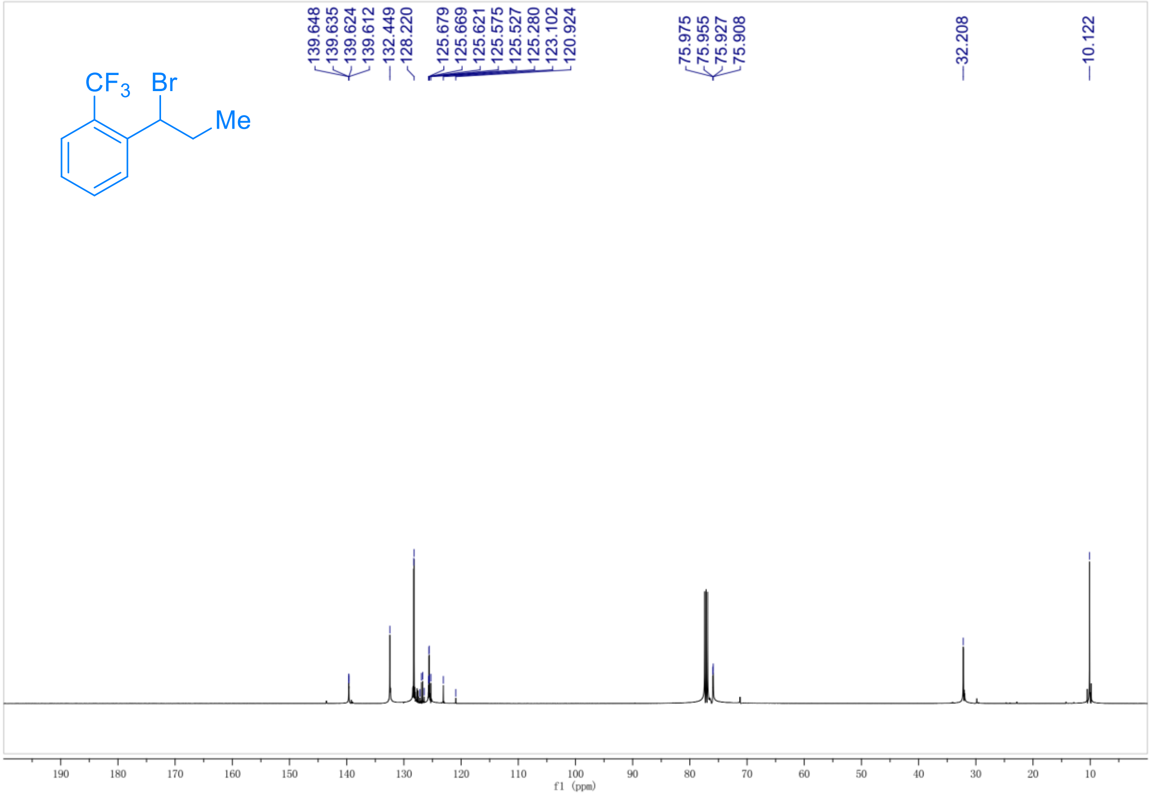

$^{13}C\{^1H\}$ NMR spectrum of compound **16a** in $CDCl_3$ (125 MHz).

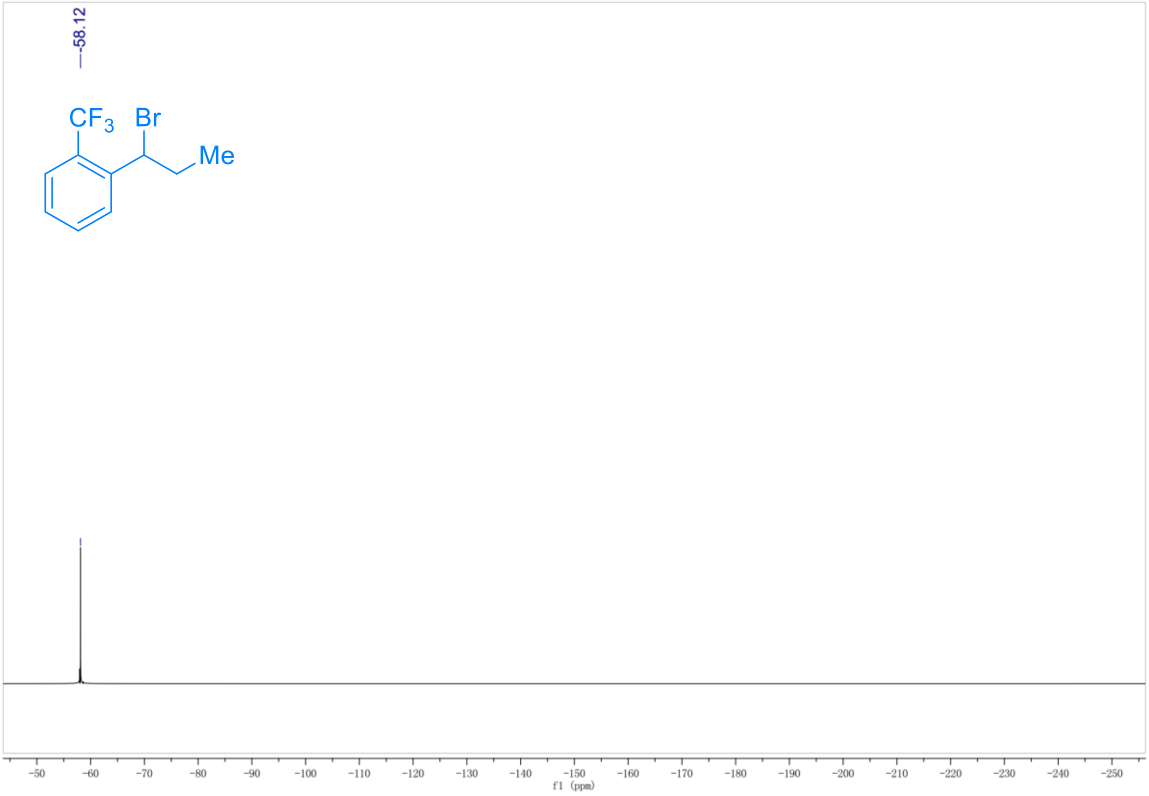

$^{19}F\{^1H\}$ NMR spectrum of compound **16a** in $CDCl_3$ (470 MHz).

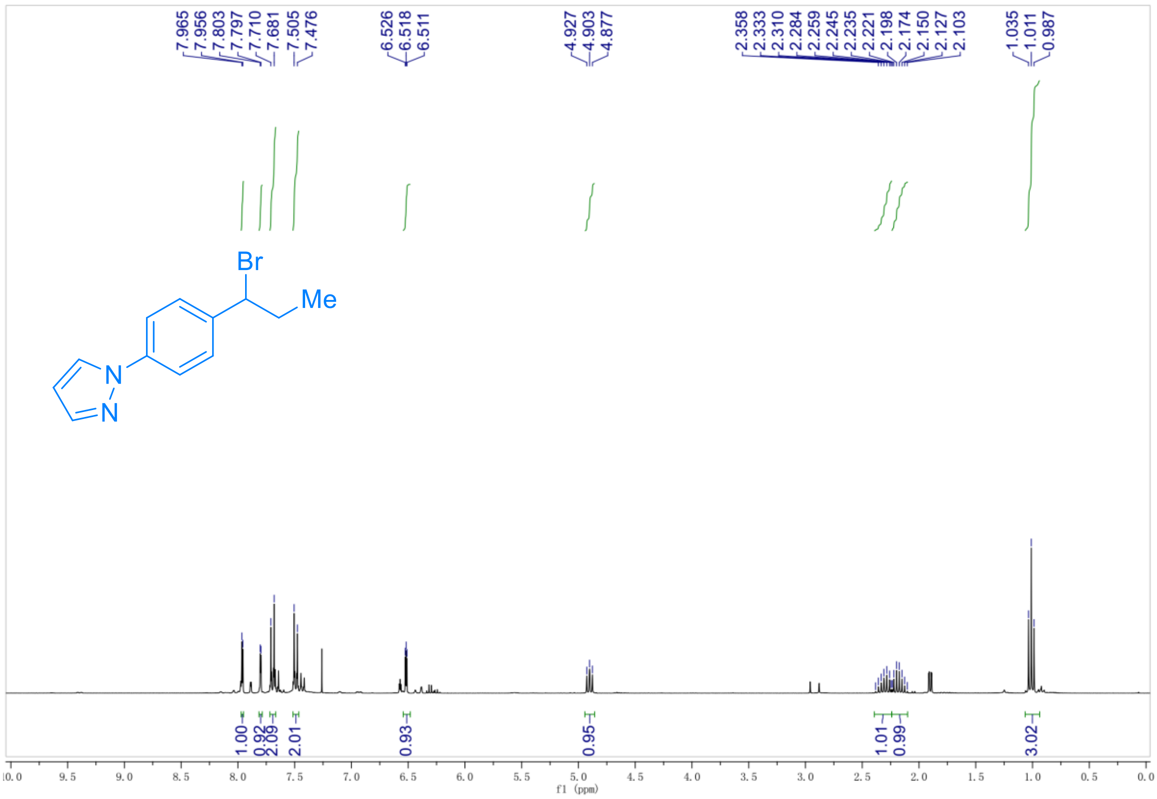

^{1}H NMR spectrum of compound **19a** in $CDCl_3$ (300 MHz).

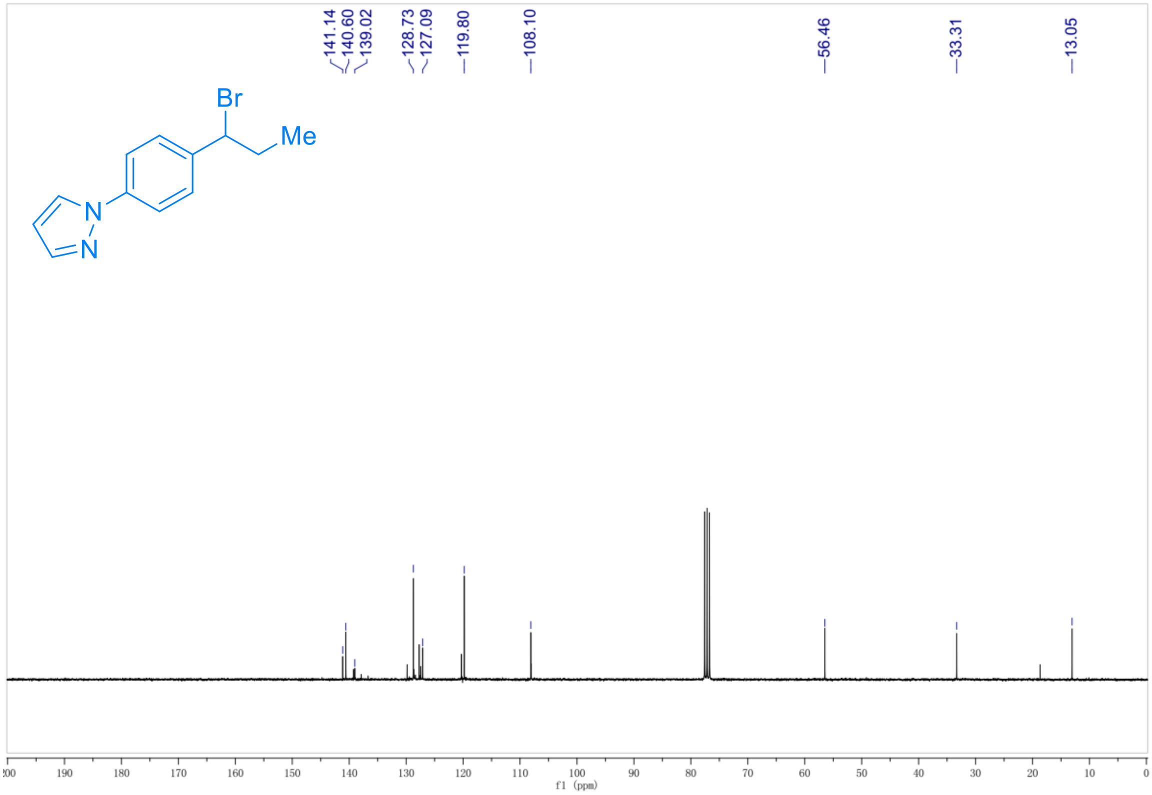

^{13}C{^{1}H} NMR spectrum of compound **19a** in $CDCl_3$ (75 MHz).

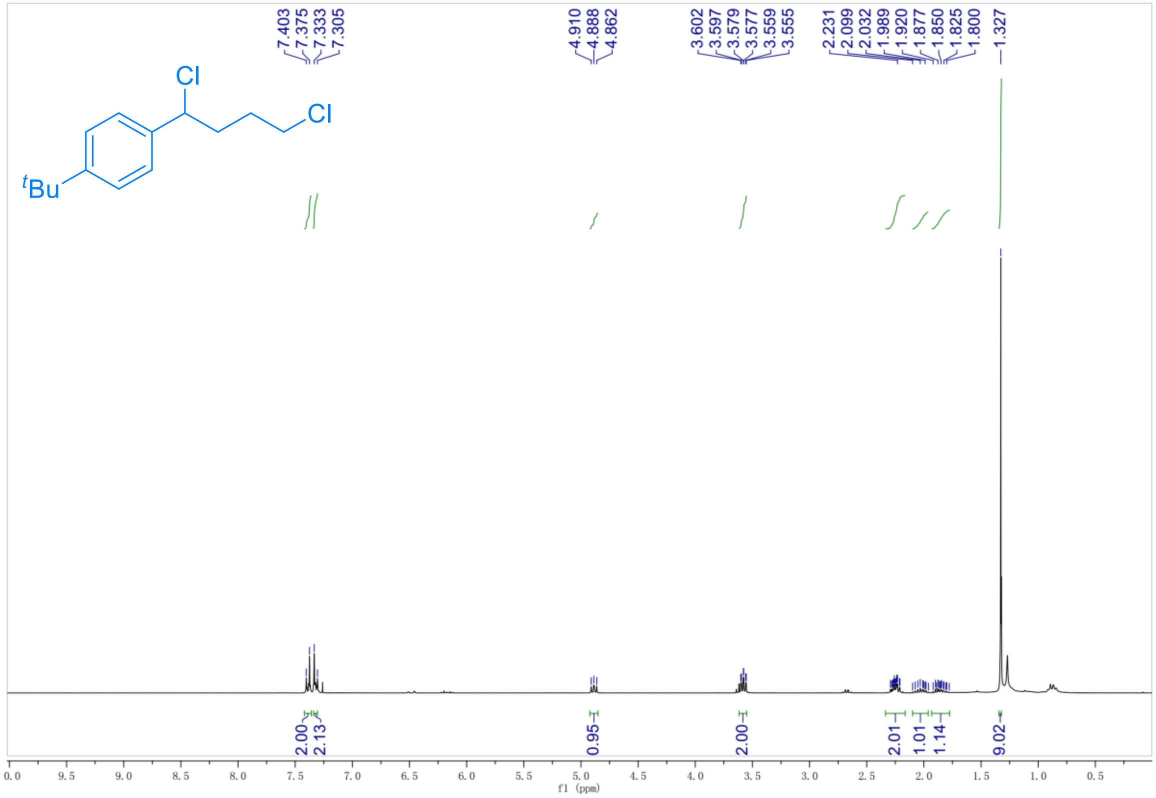

^{1}H NMR spectrum of compound **20a** in $CDCl_3$ (300 MHz).

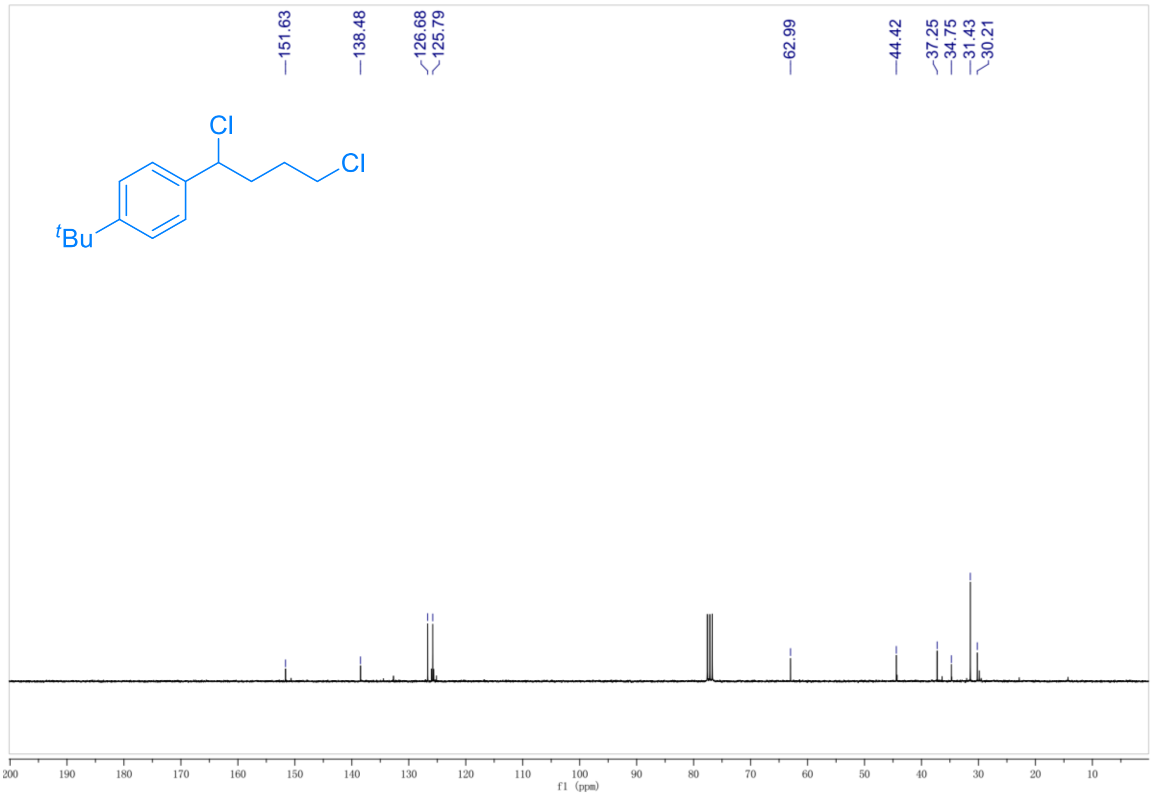

^{13}C{^{1}H} NMR spectrum of compound **20a** in $CDCl_3$ (75 MHz).

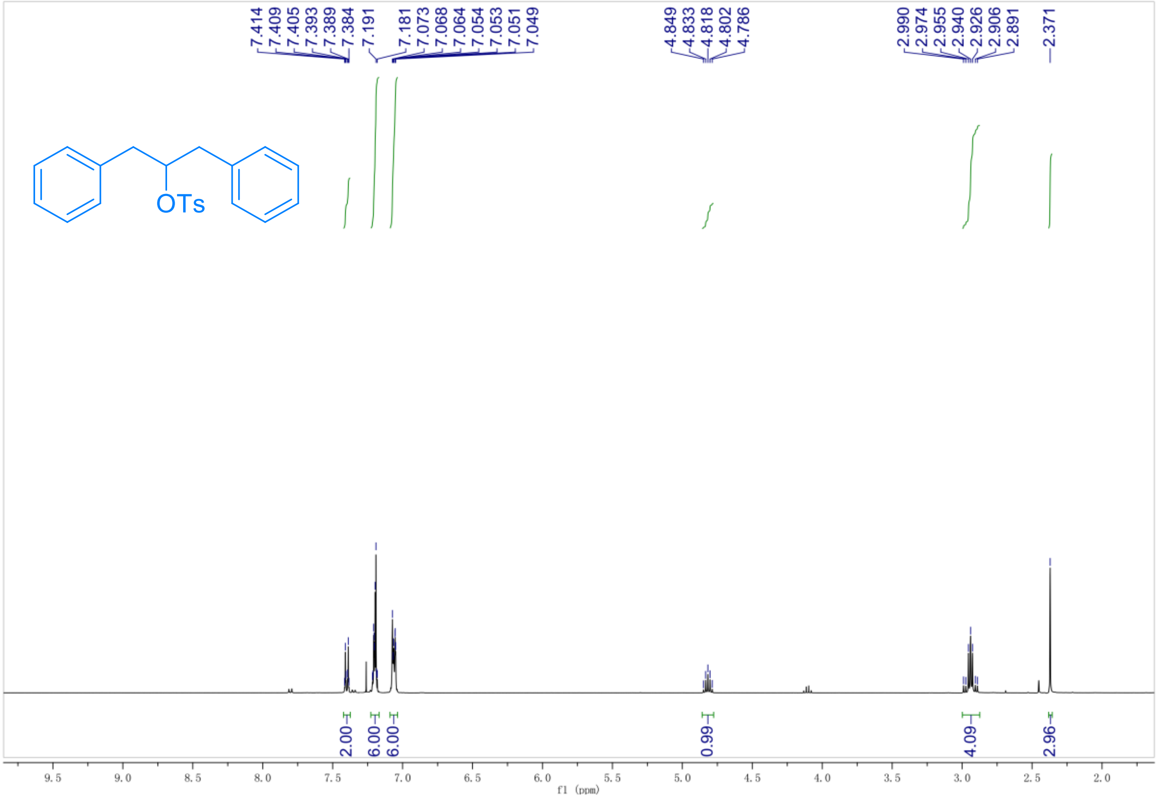

^{1}H NMR spectrum of compound **22a** in $CDCl_3$ (400 MHz).

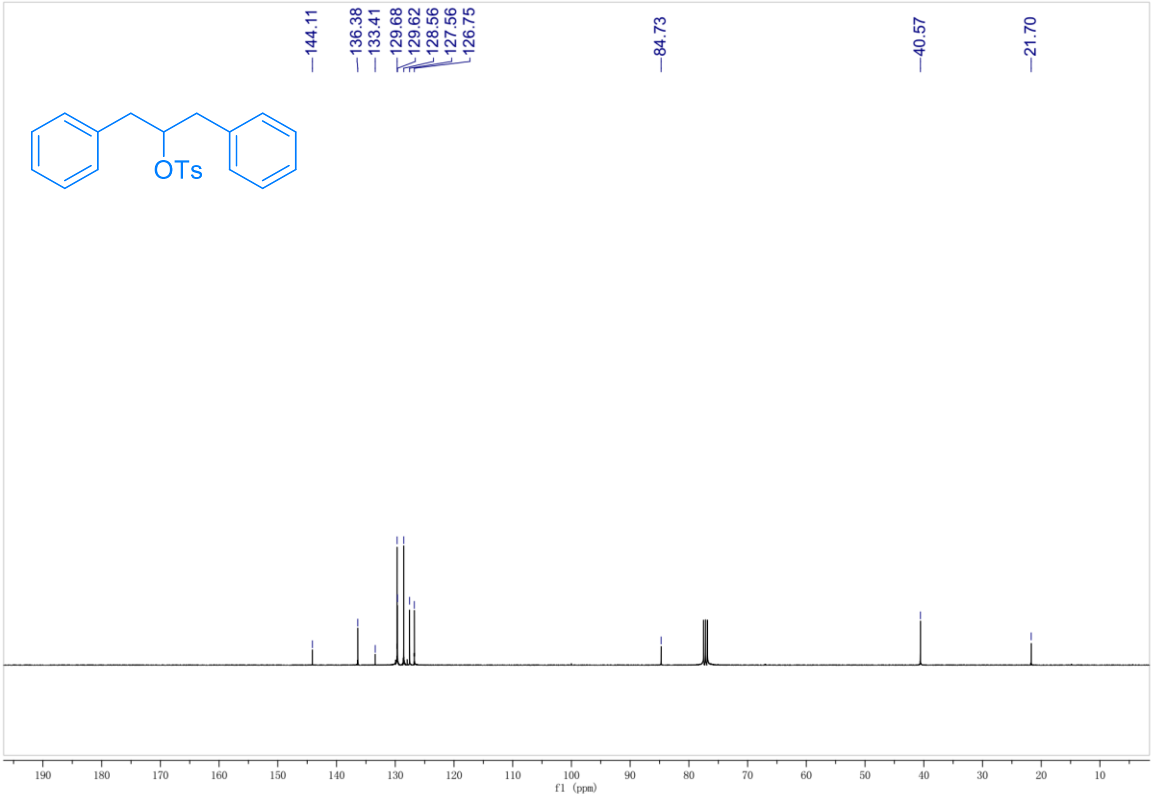

^{13}C{^{1}H} NMR spectrum of compound **22a** in $CDCl_3$ (100 MHz).

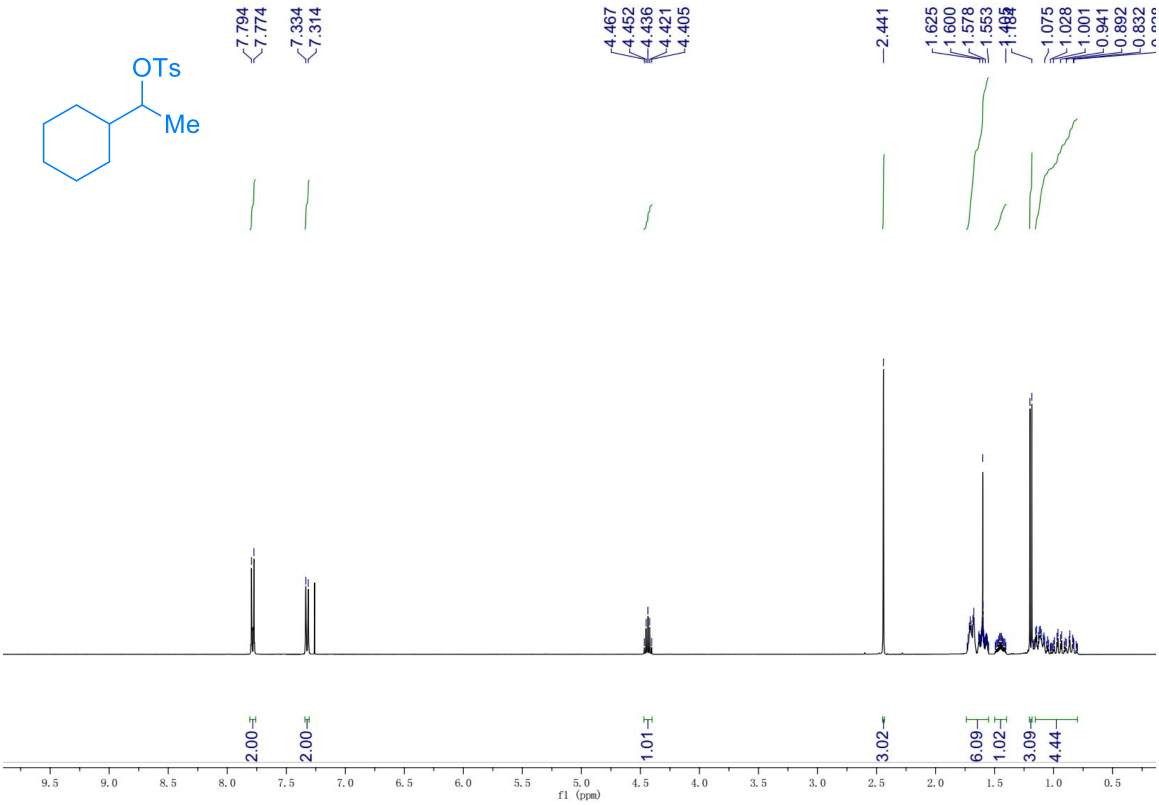

^{1}H NMR spectrum of compound **23a** in $CDCl_3$ (400 MHz).

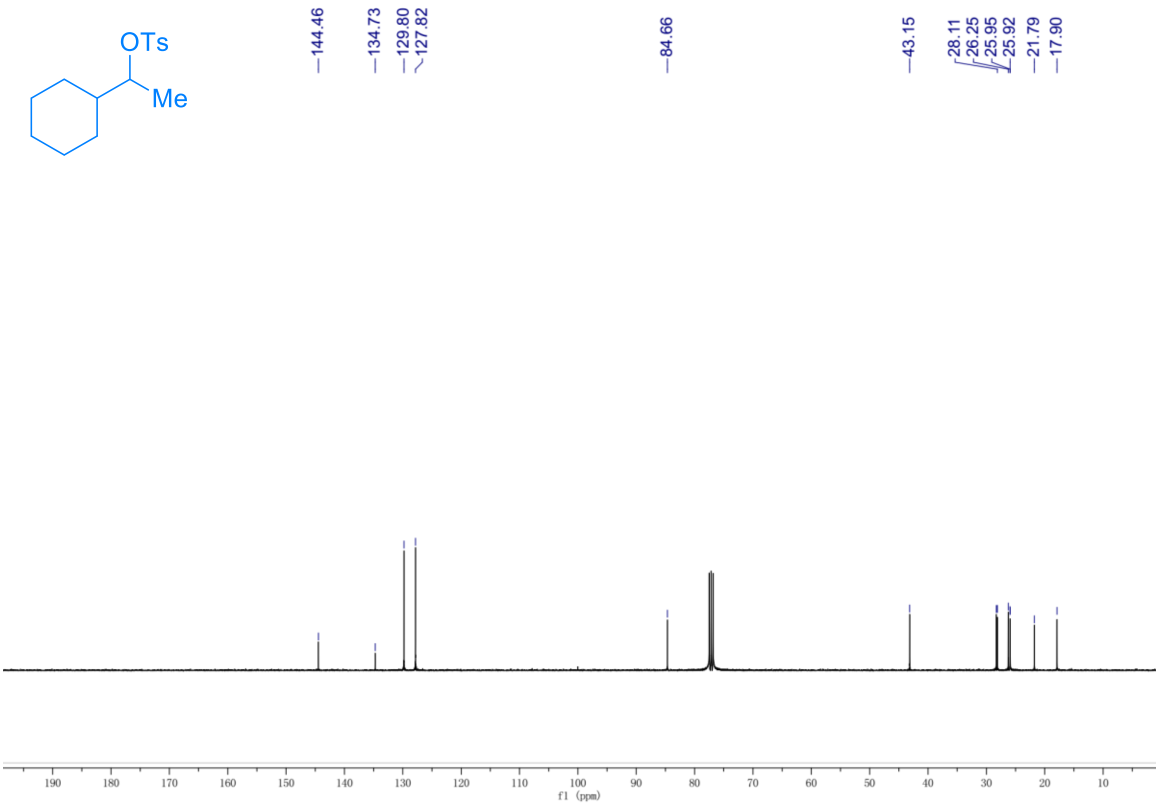

^{13}C{^{1}H} NMR spectrum of compound **23a** in $CDCl_3$ (100 MHz).

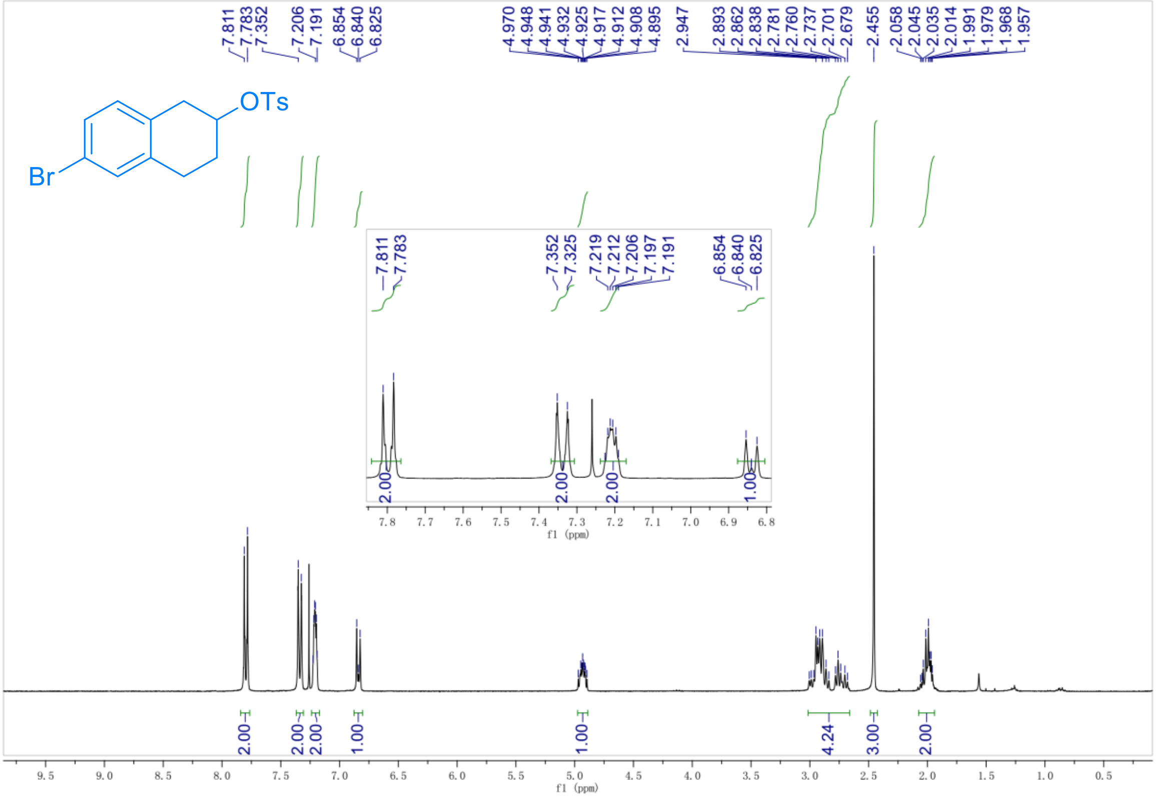

^{1}H NMR spectrum of compound **28a** in $CDCl_3$ (300 MHz).

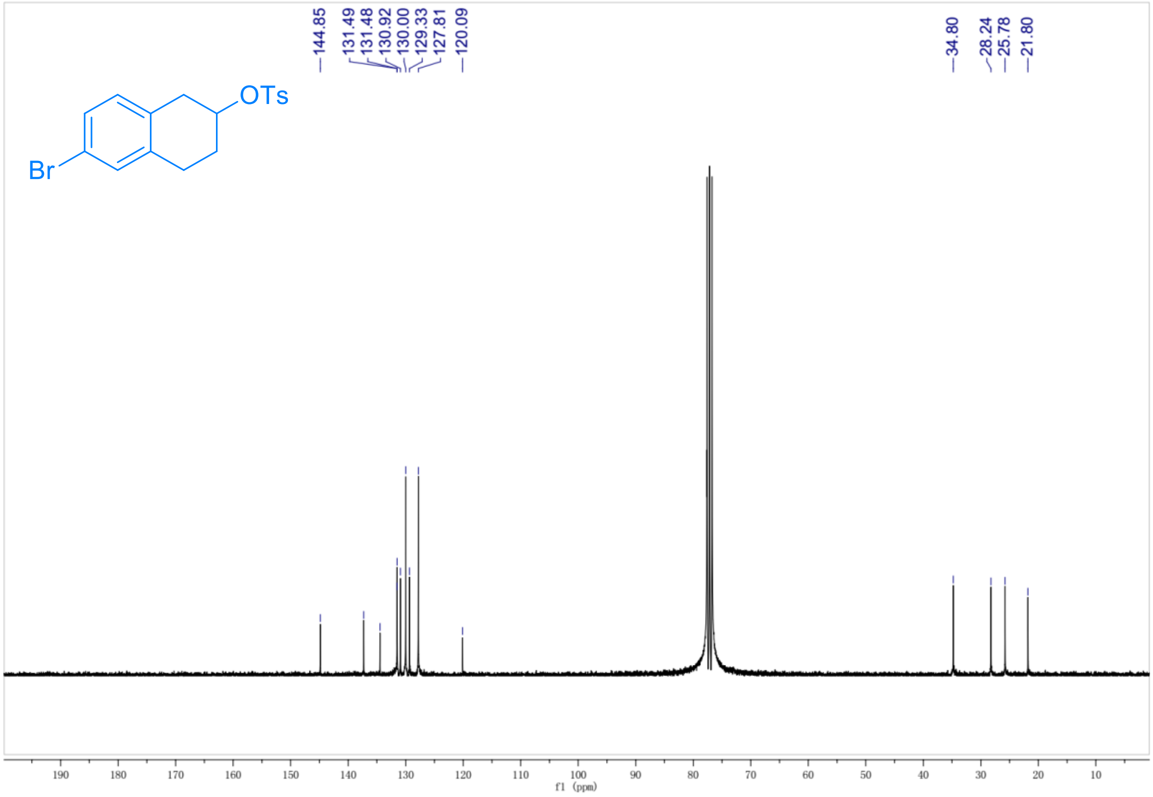

^{13}C{^{1}H} NMR spectrum of compound **28a** in $CDCl_3$ (75 MHz).

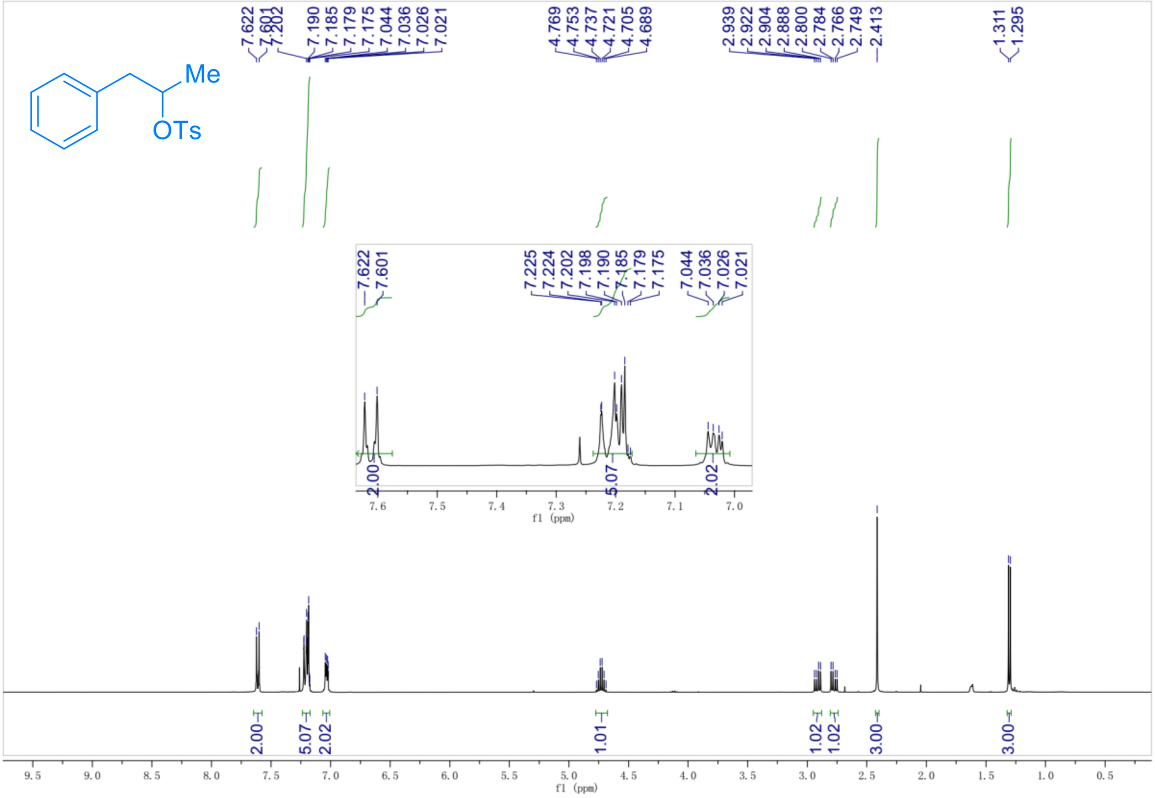

^{1}H NMR spectrum of compound **29a** in $CDCl_3$ (400 MHz).

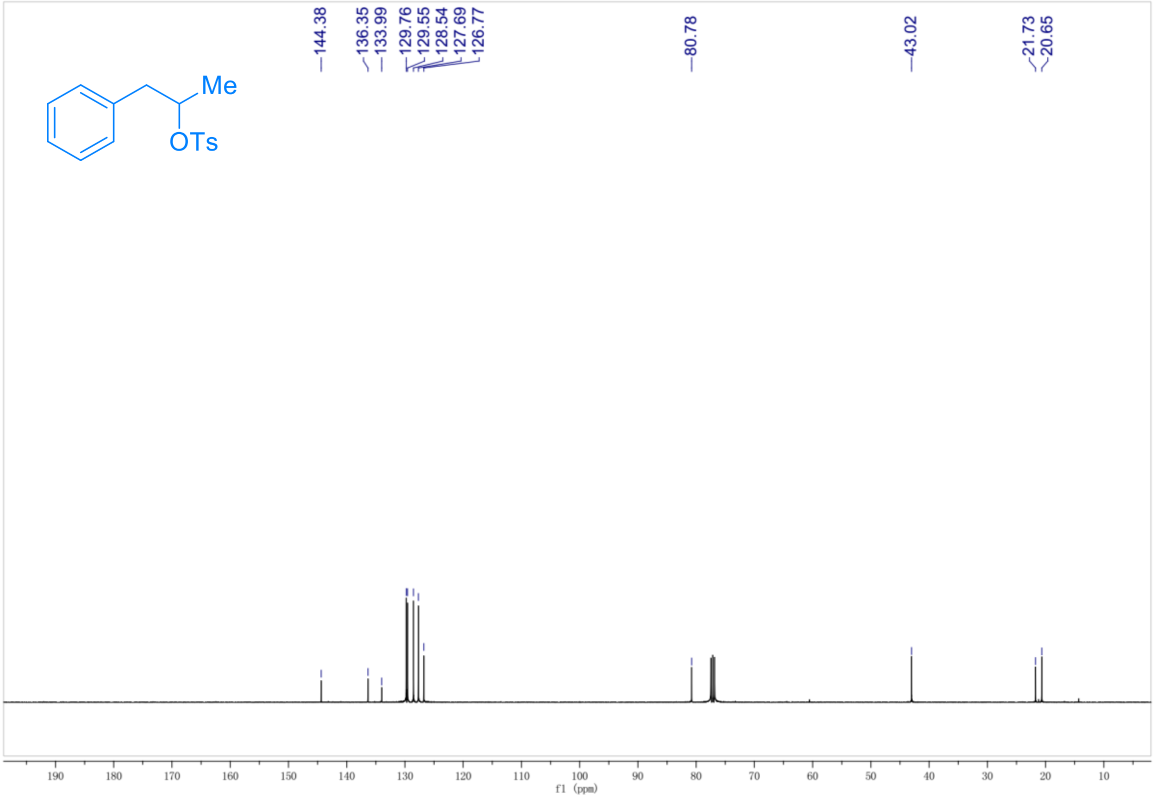

$^{13}C\{^{1}H\}$ NMR spectrum of compound **29a** in $CDCl_3$ (100 MHz).

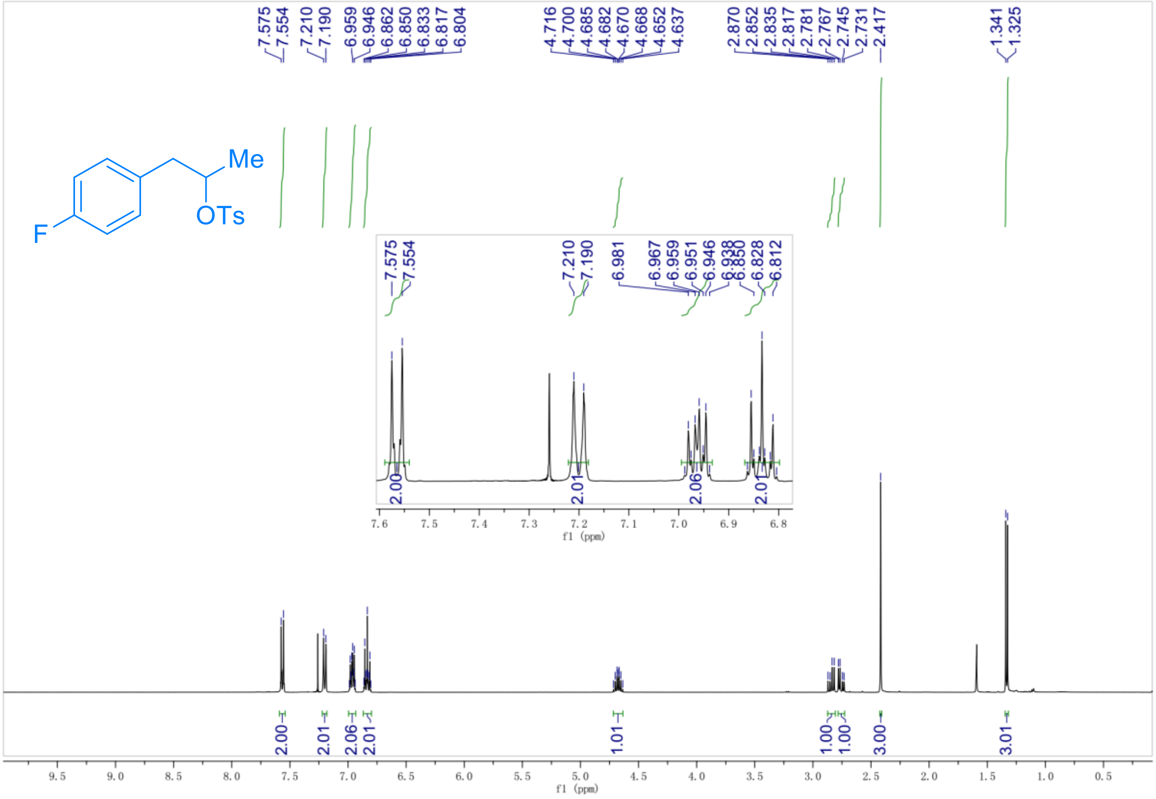

^{1}H NMR spectrum of compound **30a** in $CDCl_3$ (400 MHz).

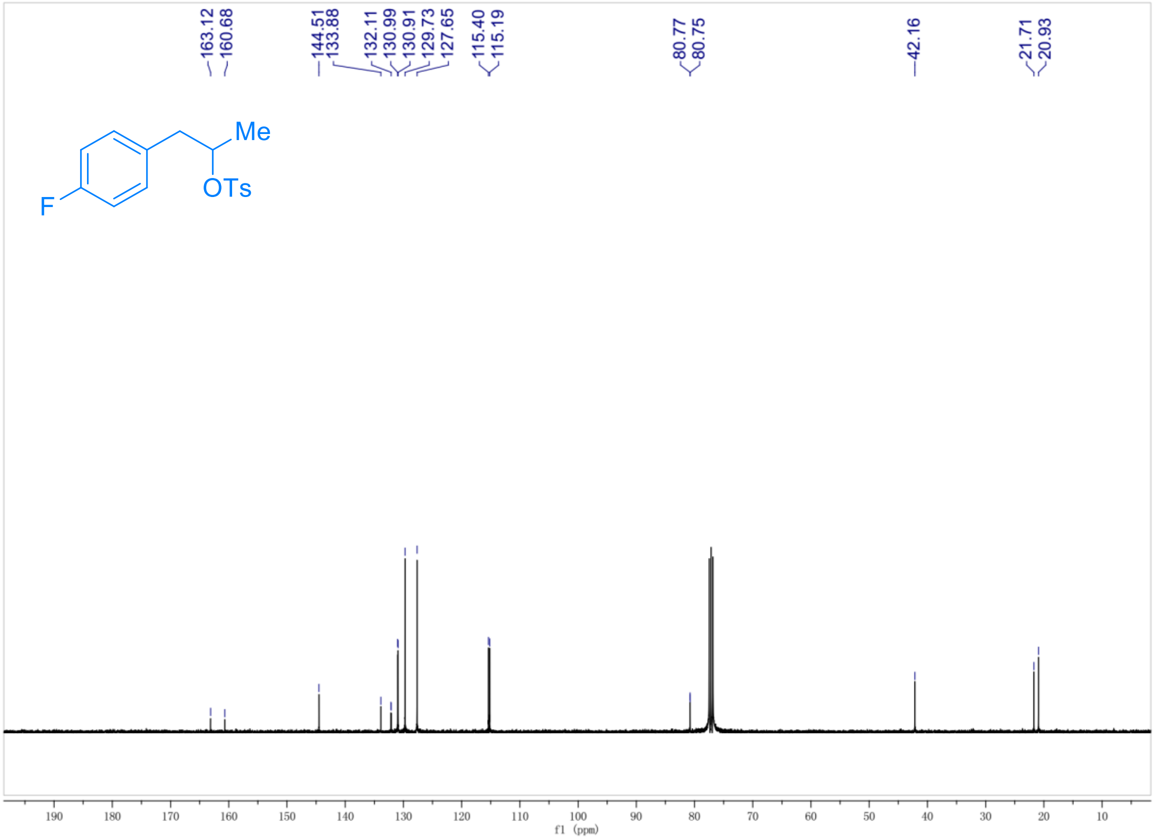

$^{13}C\{^{1}H\}$ NMR spectrum of compound **30a** in $CDCl_3$ (100 MHz).

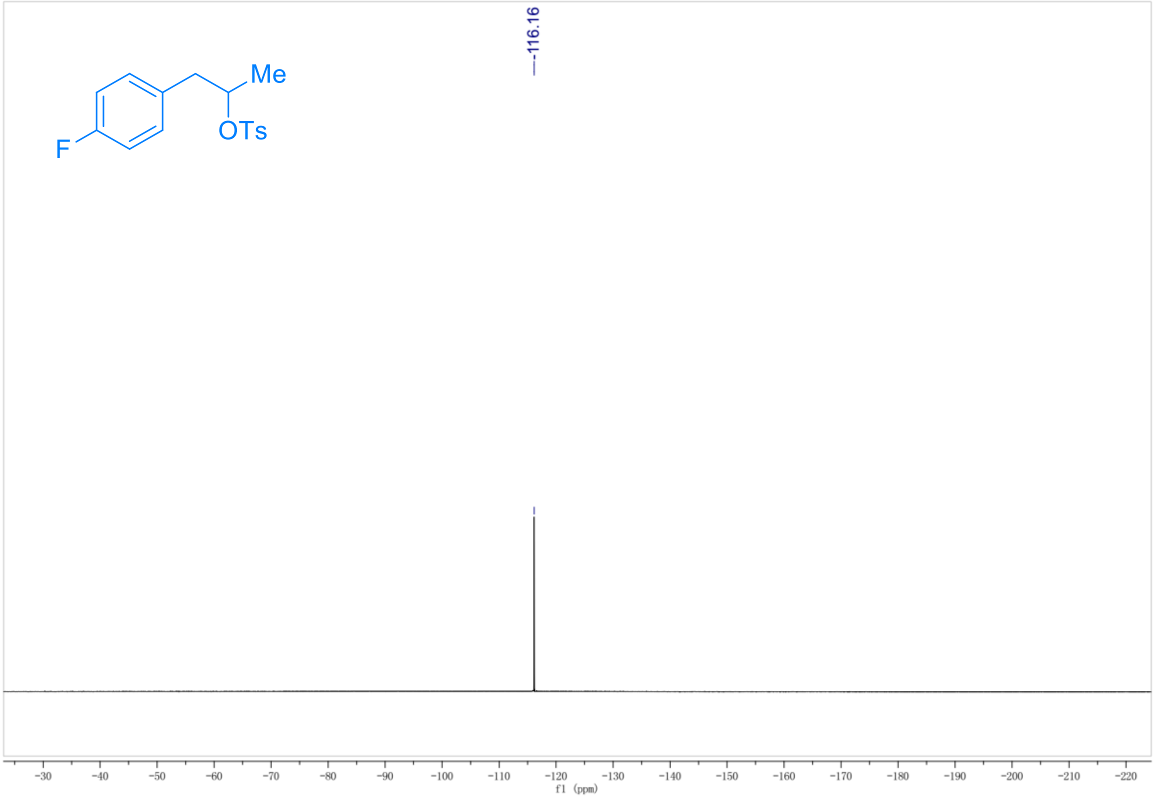

^{19}F{^{1}H} NMR spectrum of compound **30a** in $CDCl_3$ (376 MHz).

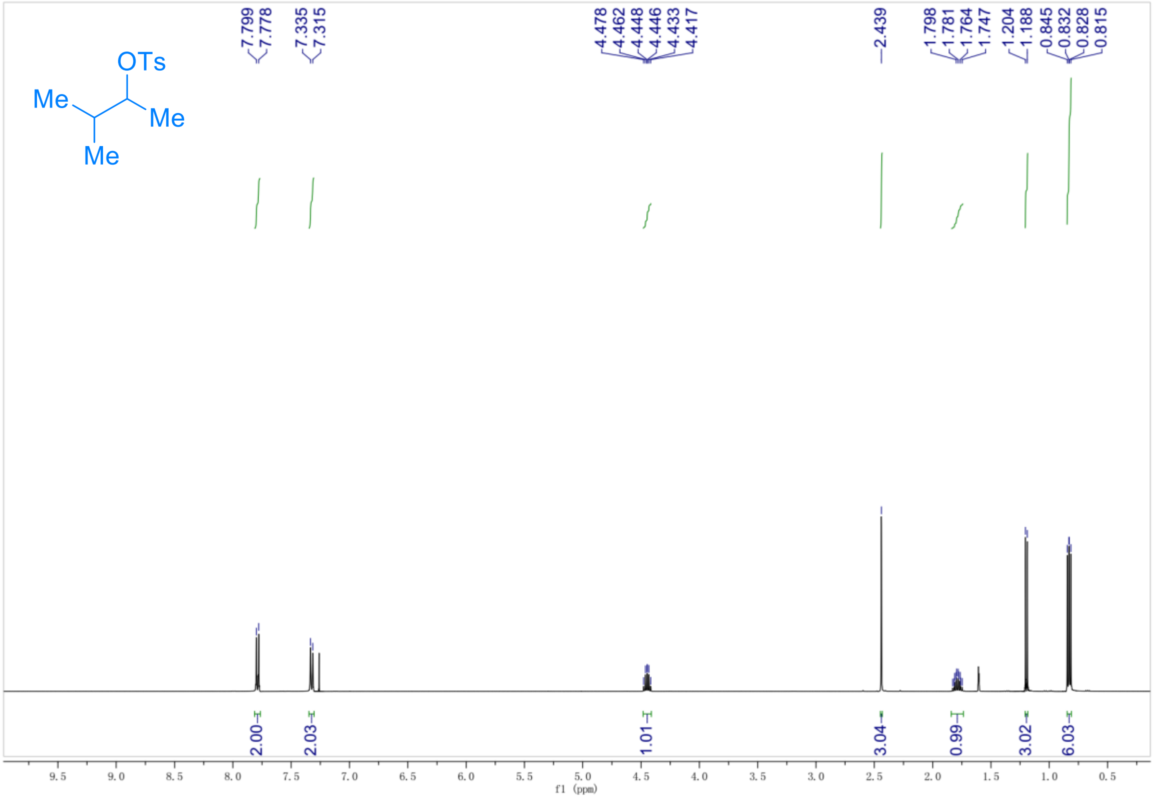

^{1}H NMR spectrum of compound **31a** in $CDCl_3$ (400 MHz).

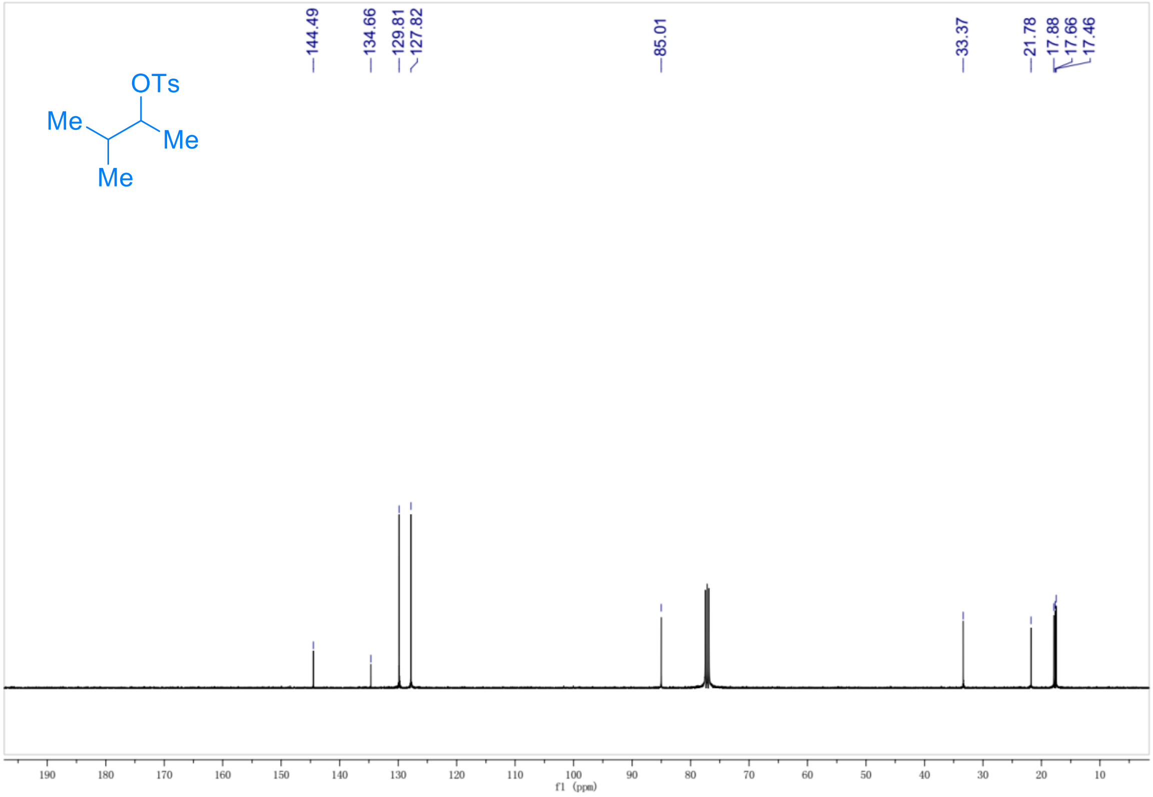

$^{13}C\{^1H\}$ NMR spectrum of compound **31a** in $CDCl_3$ (100 MHz).

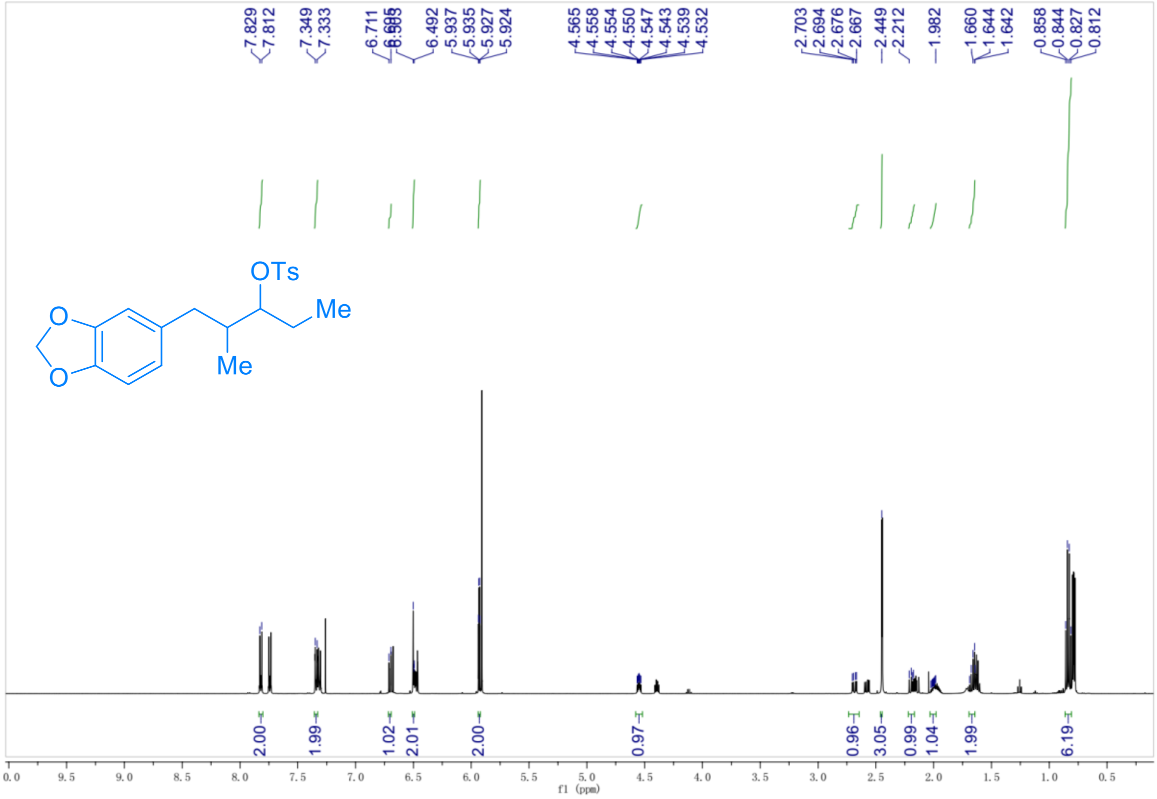

^1H NMR spectrum of compound **32a** in $CDCl_3$ (500 MHz).

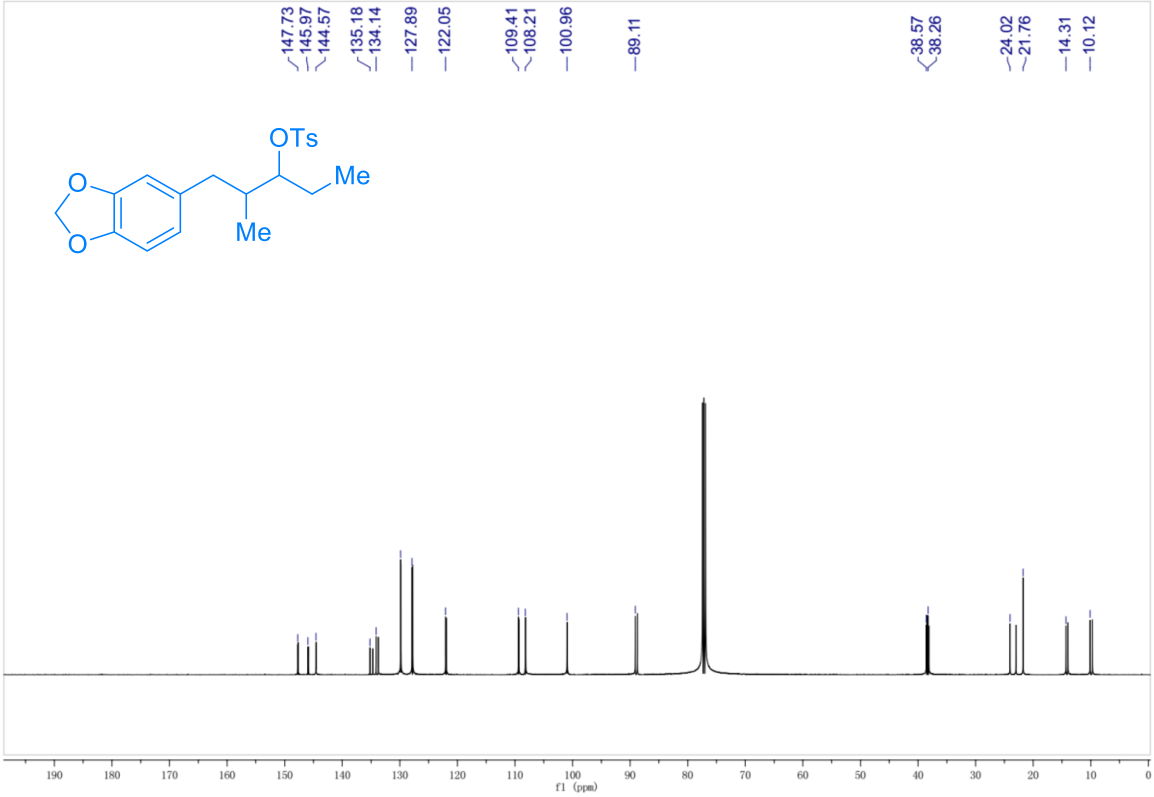

$^{13}C\{^{1}H\}$ NMR spectrum of compound **32a** in $CDCl_3$ (125 MHz).

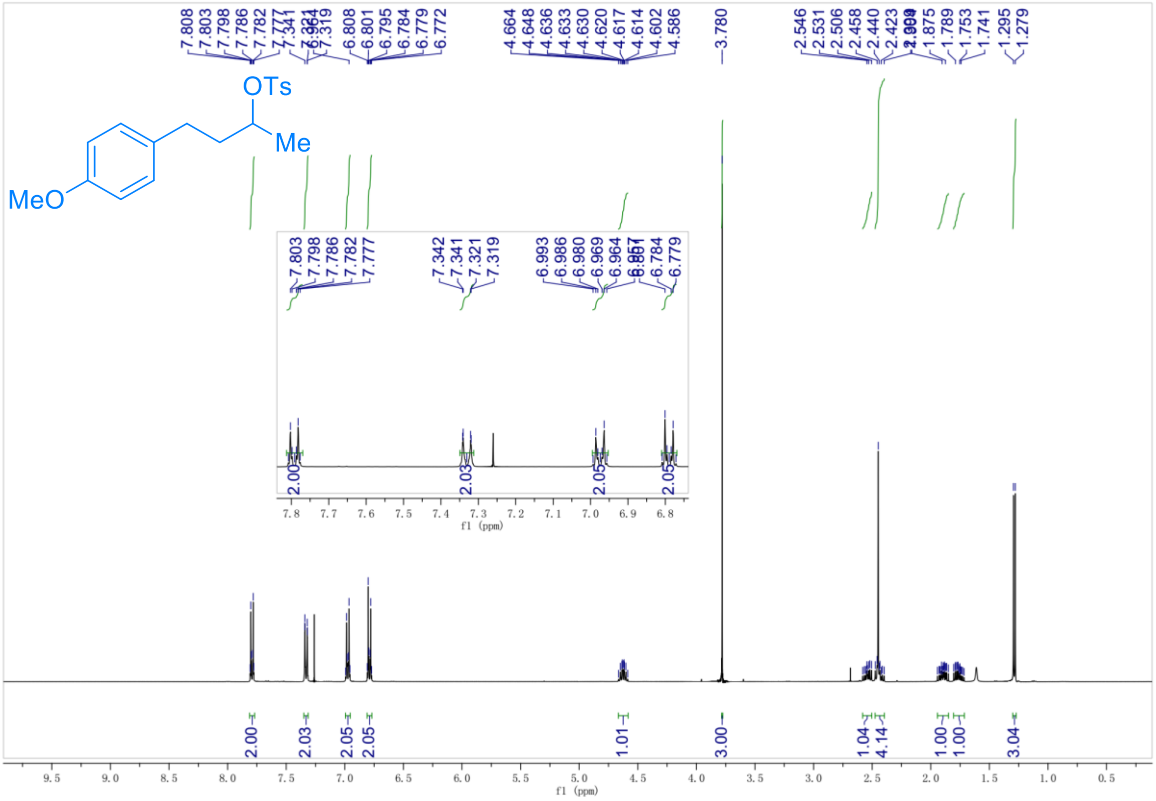

^{1}H NMR spectrum of compound **33a** in $CDCl_3$ (400 MHz).

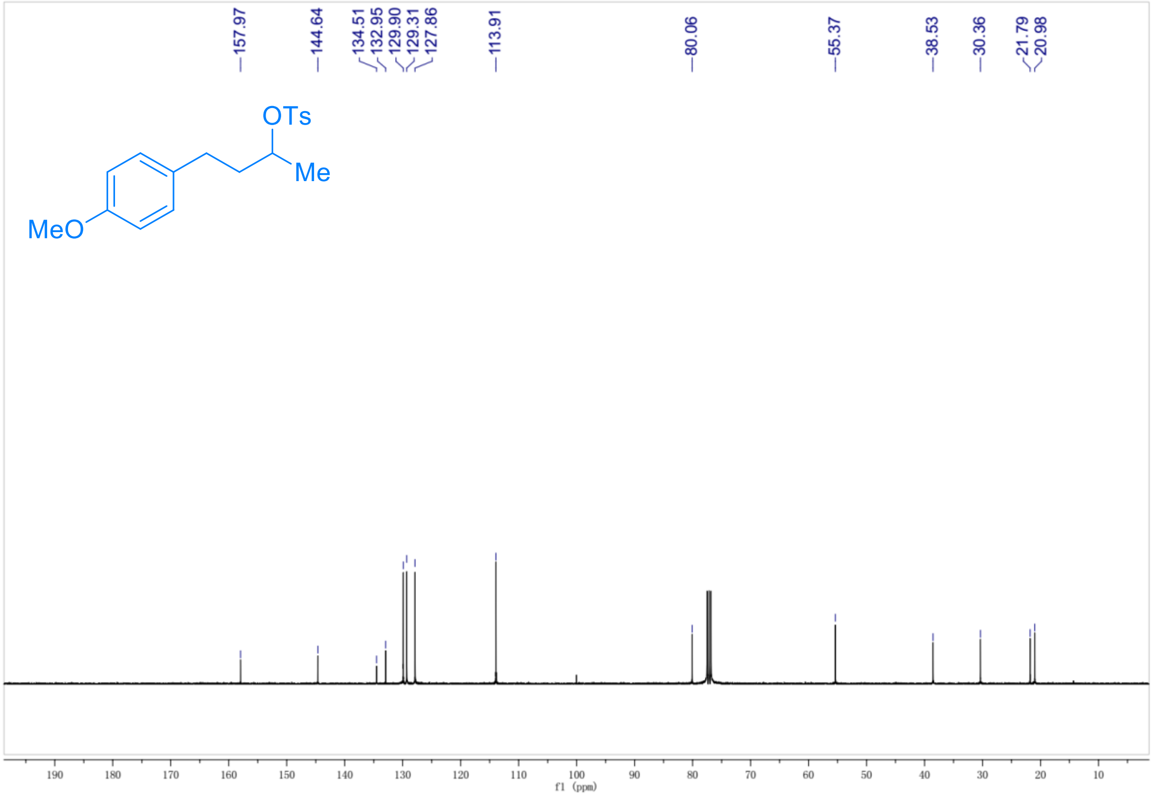

$^{13}C\{^{1}H\}$ NMR spectrum of compound **33a** in $CDCl_3$ (100 MHz).

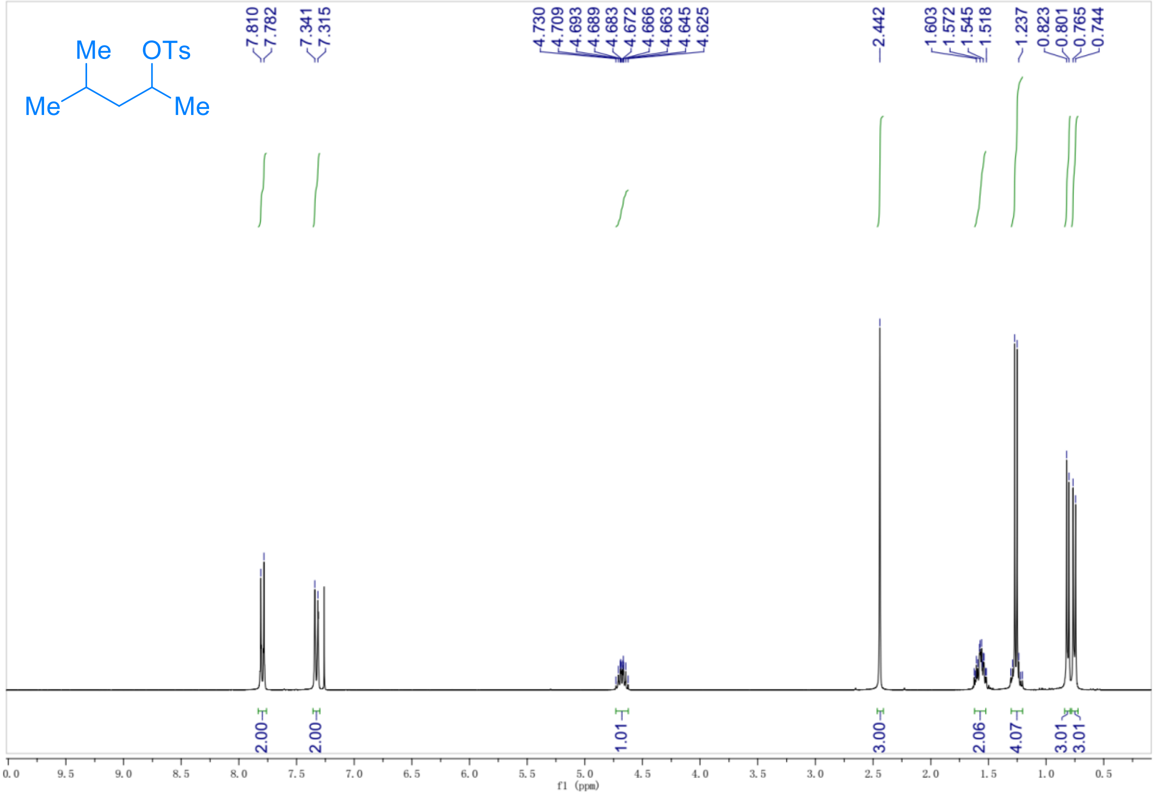

^{1}H NMR spectrum of compound **34a** in $CDCl_3$ (300 MHz).

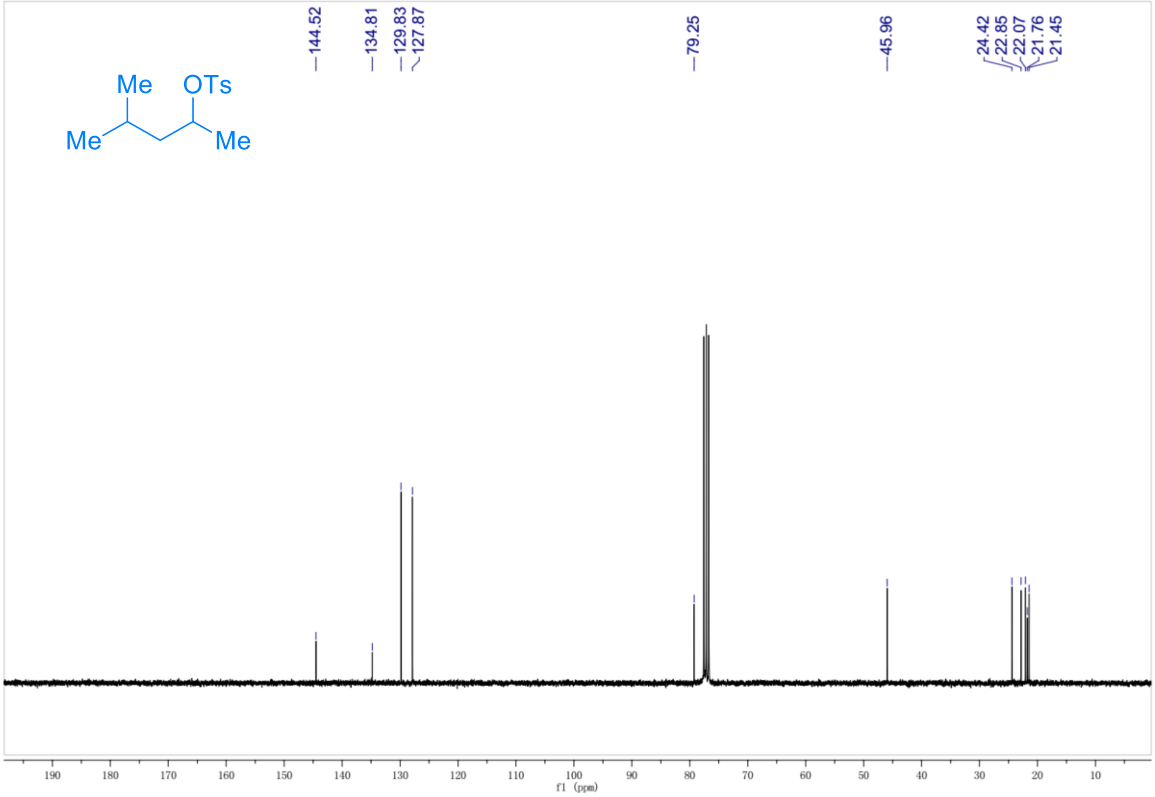

$^{13}C\{^{1}H\}$ NMR spectrum of compound **34a** in $CDCl_3$ (75 MHz).

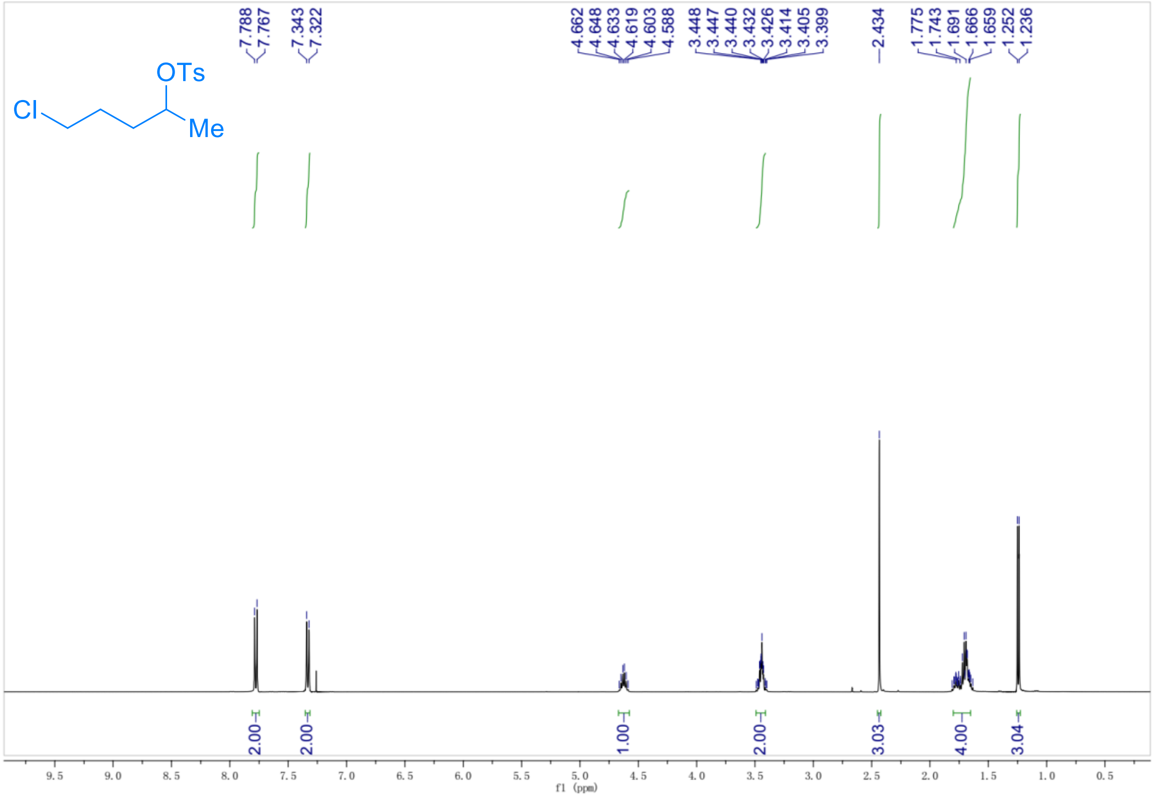

^{1}H NMR spectrum of compound **37a** in $CDCl_3$ (400 MHz).

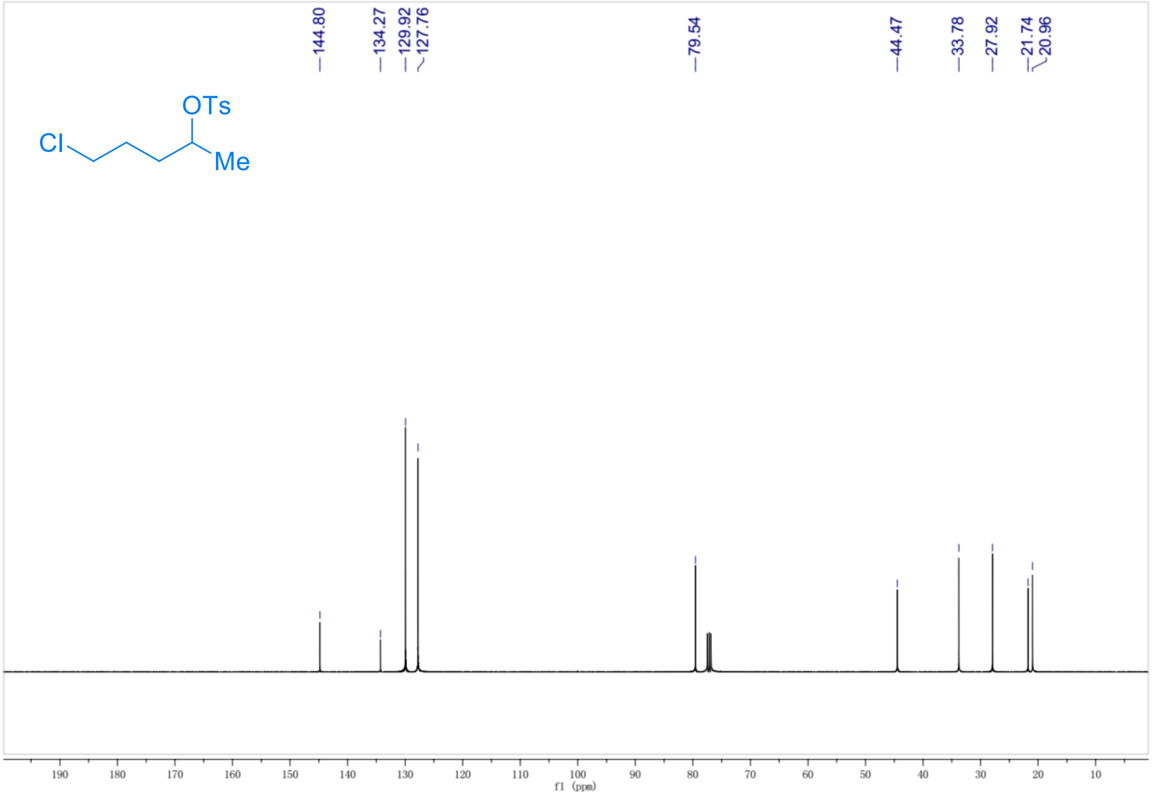

$^{13}C\{^{1}H\}$ NMR spectrum of compound **37a** in $CDCl_3$ (100 MHz).

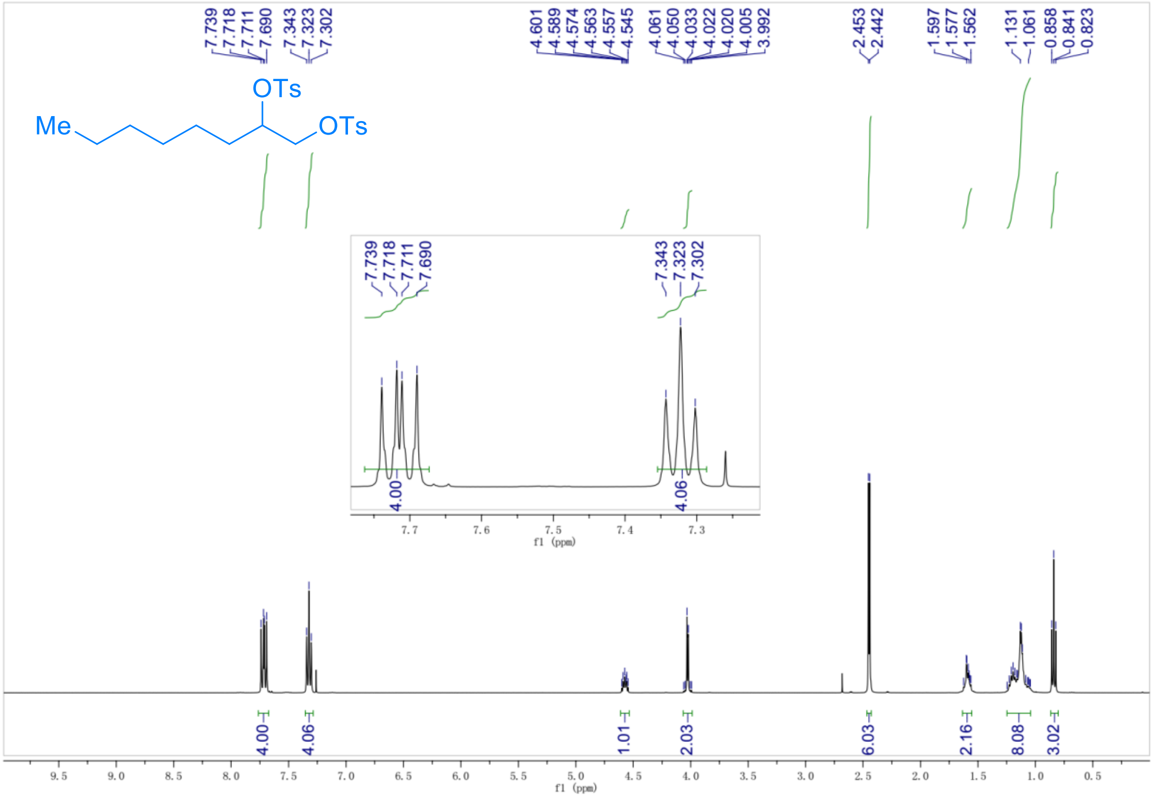

^{1}H NMR spectrum of compound **40a** in $CDCl_3$ (400 MHz).

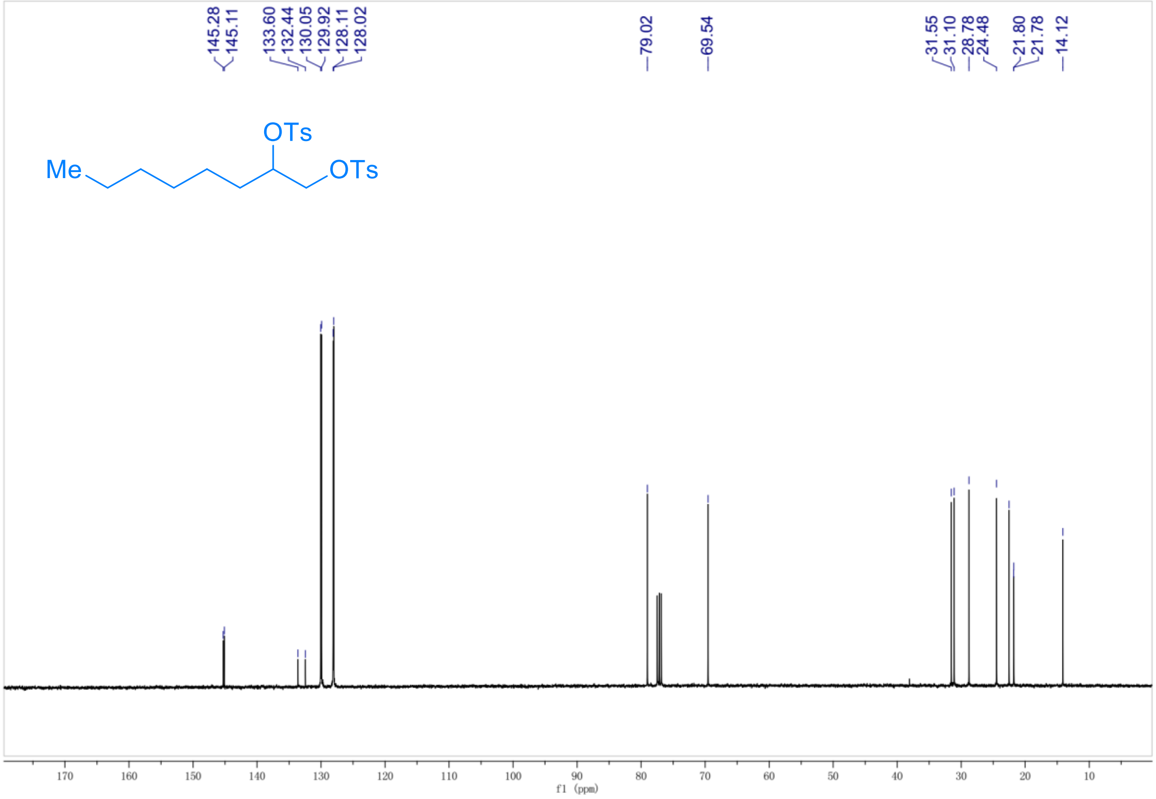

$^{13}C\{^1H\}$ NMR spectrum of compound **40a** in $CDCl_3$ (100 MHz).

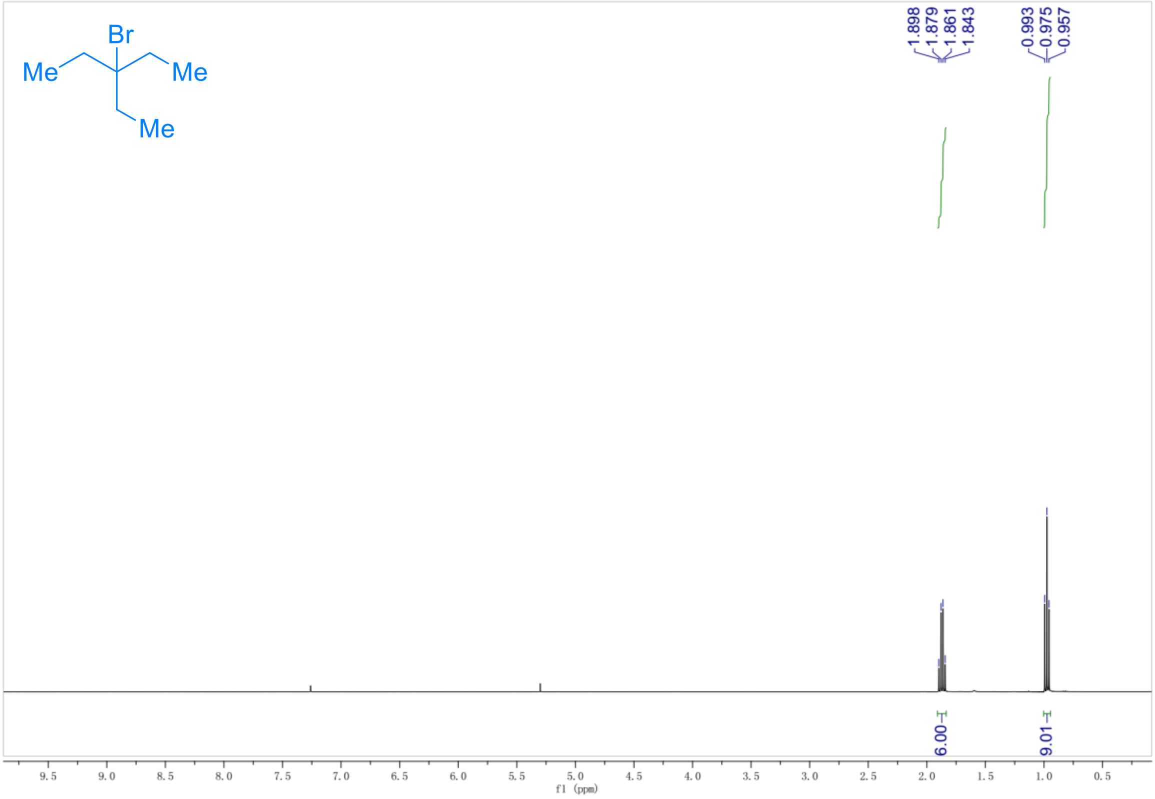

^1H NMR spectrum of compound **43a** in $CDCl_3$ (400 MHz).

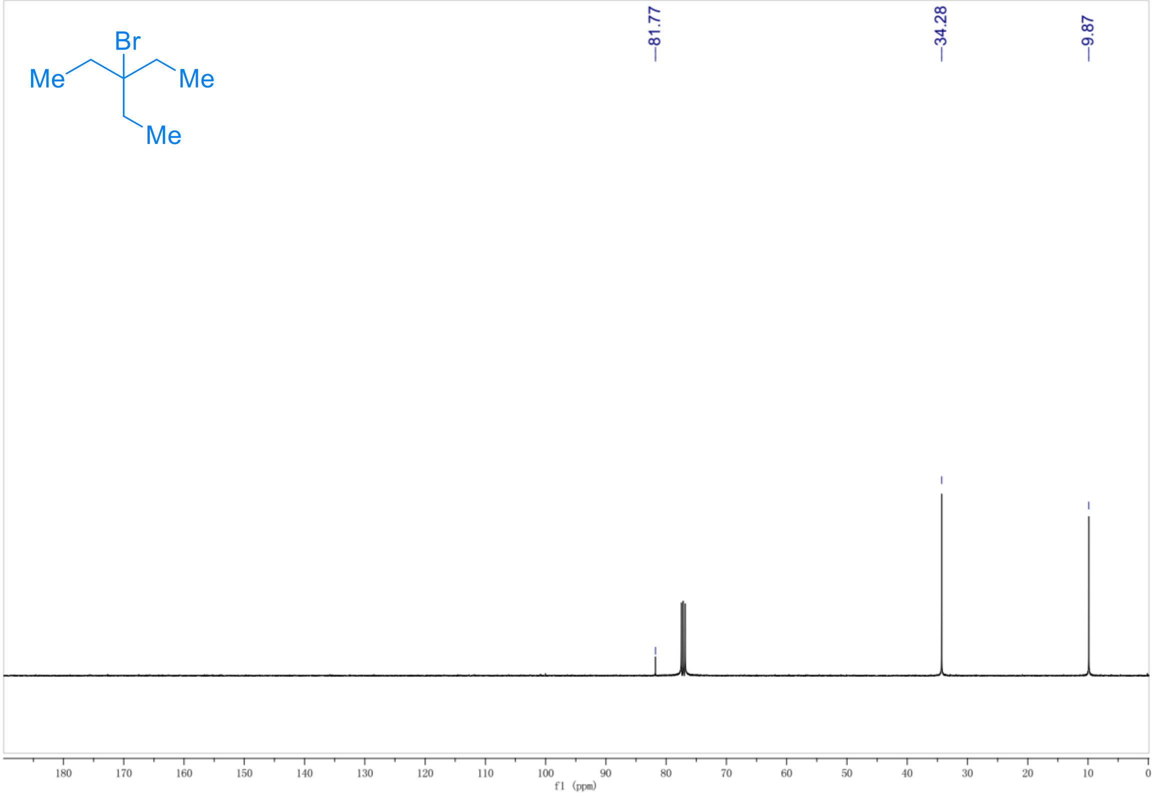

$^{13}C\{^{1}H\}$ NMR spectrum of compound **43a** in $CDCl_3$ (100 MHz).

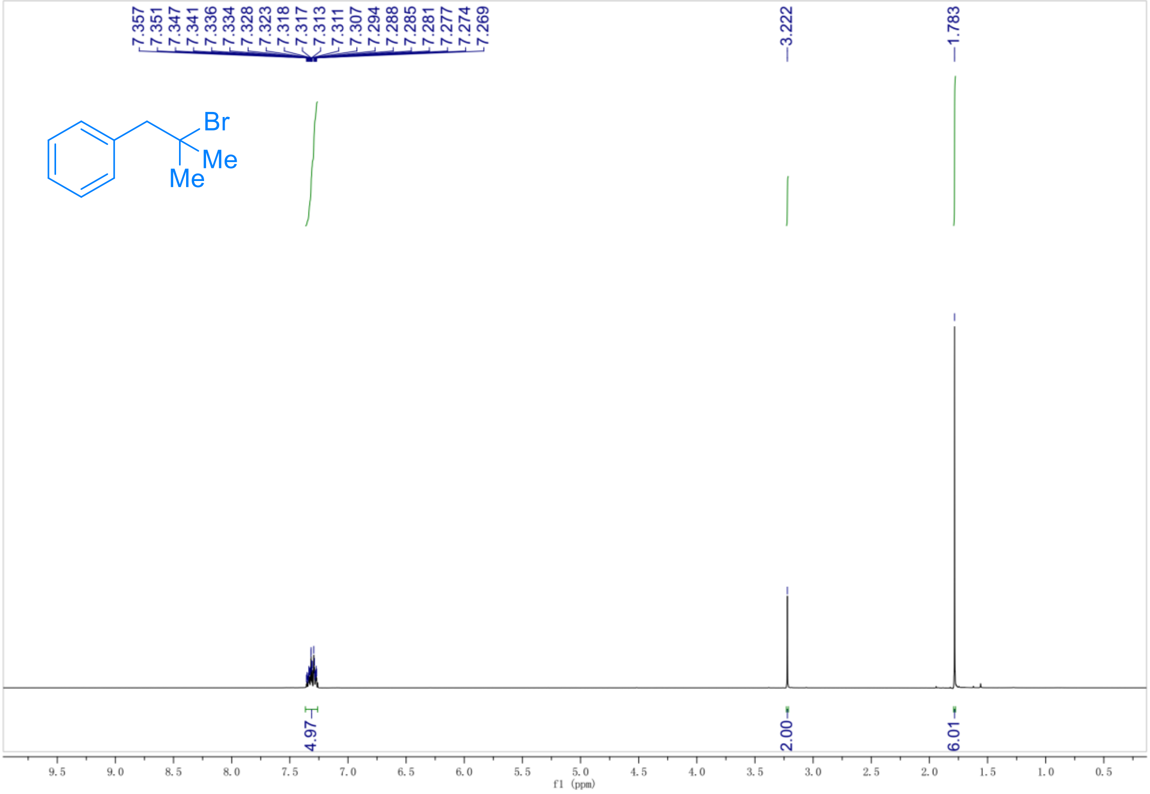

^{1}H NMR spectrum of compound **44a** in $CDCl_3$ (400 MHz).

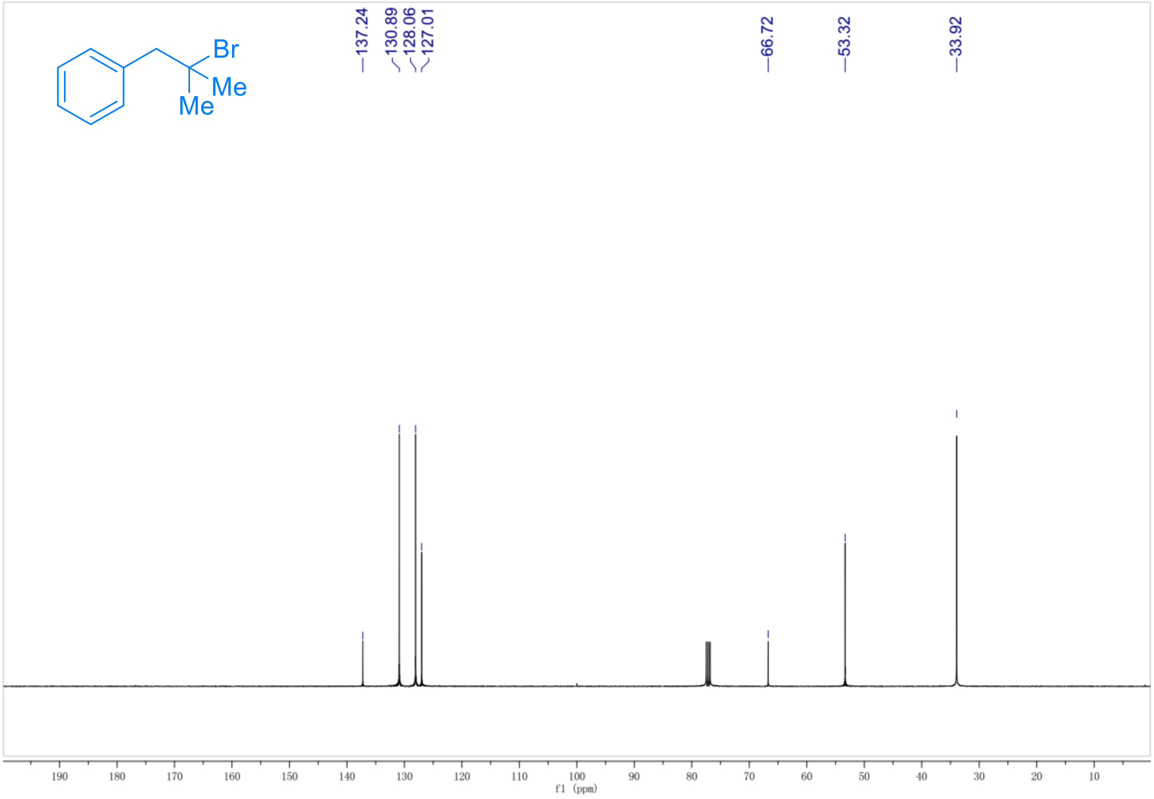

$^{13}C\{^{1}H\}$ NMR spectrum of compound **44a** in $CDCl_3$ (100 MHz).

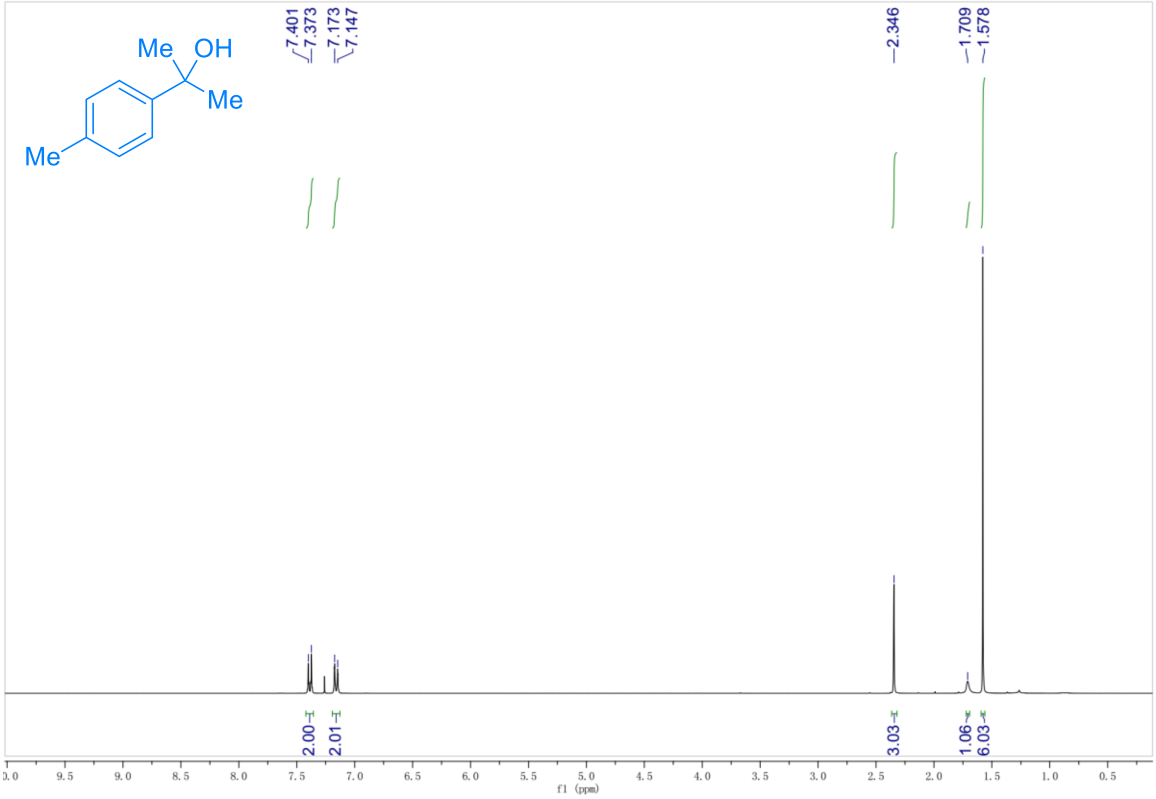

^{1}H NMR spectrum of compound **46a** in $CDCl_3$ (300 MHz).

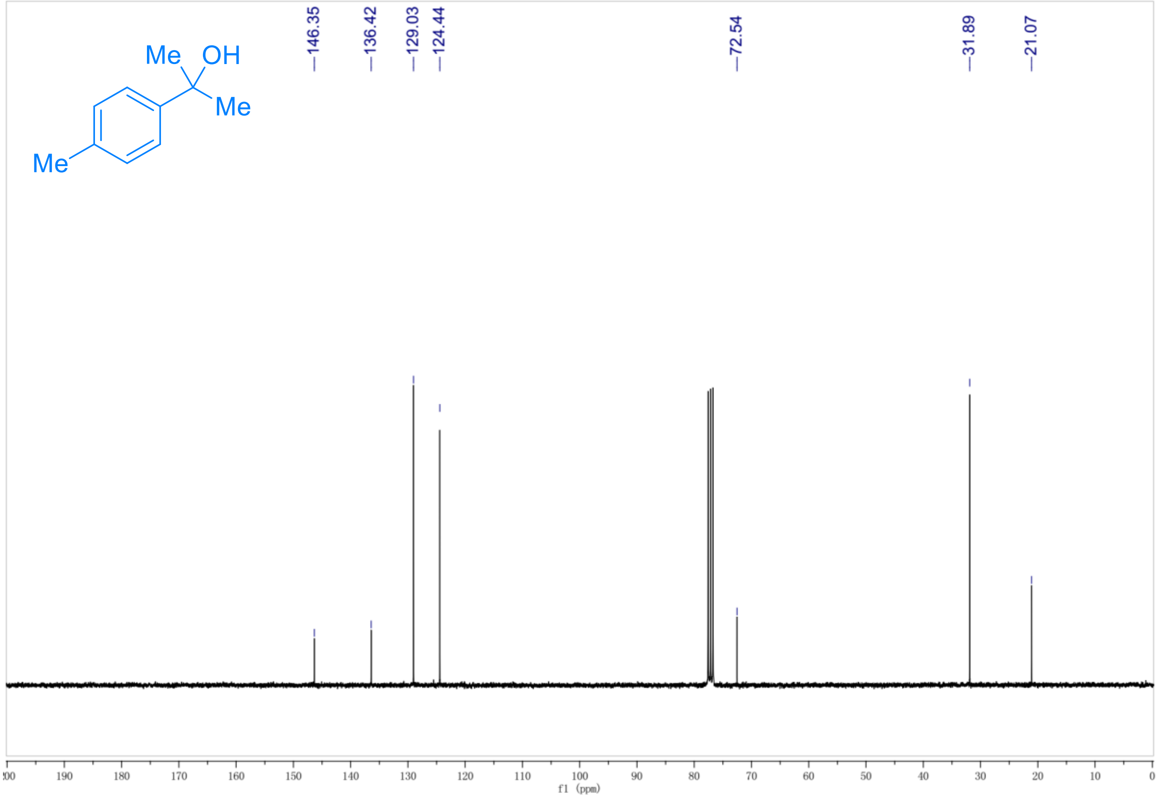

$^{13}C\{^{1}H\}$ NMR spectrum of compound **46a** in $CDCl_3$ (100 MHz).

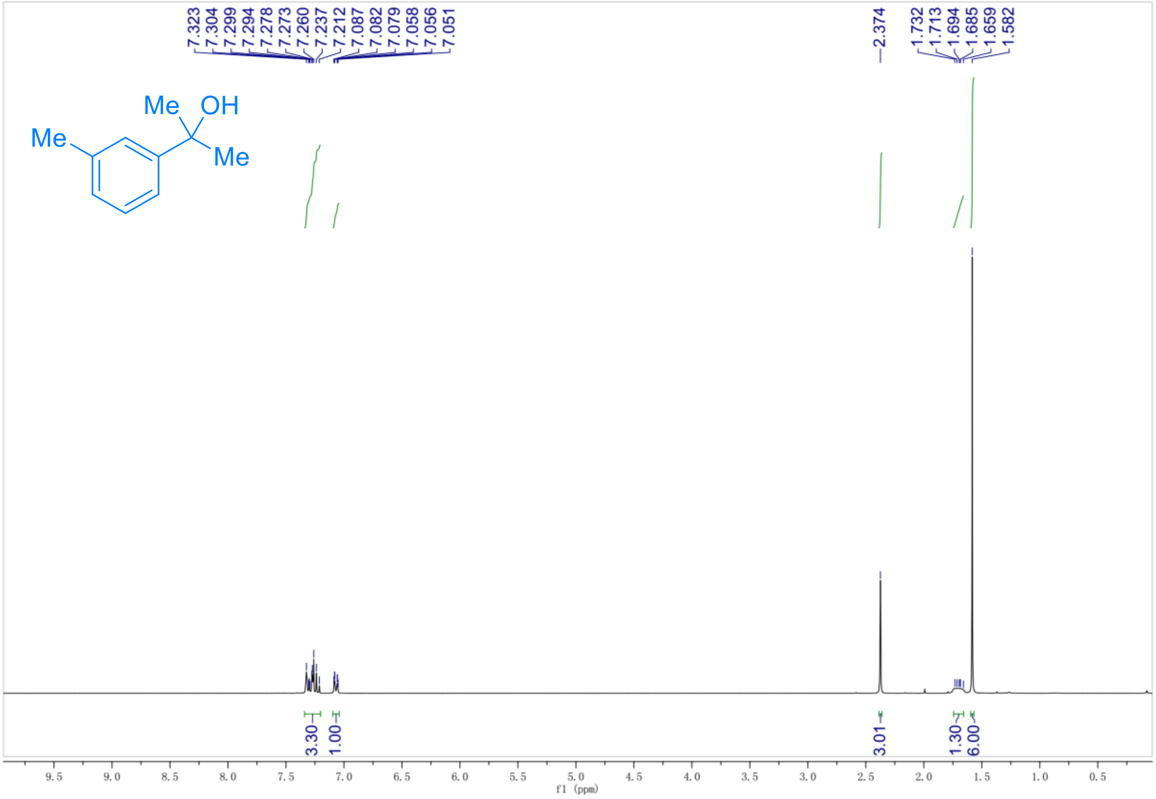

^{1}H NMR spectrum of compound **47a** in $CDCl_3$ (300 MHz).

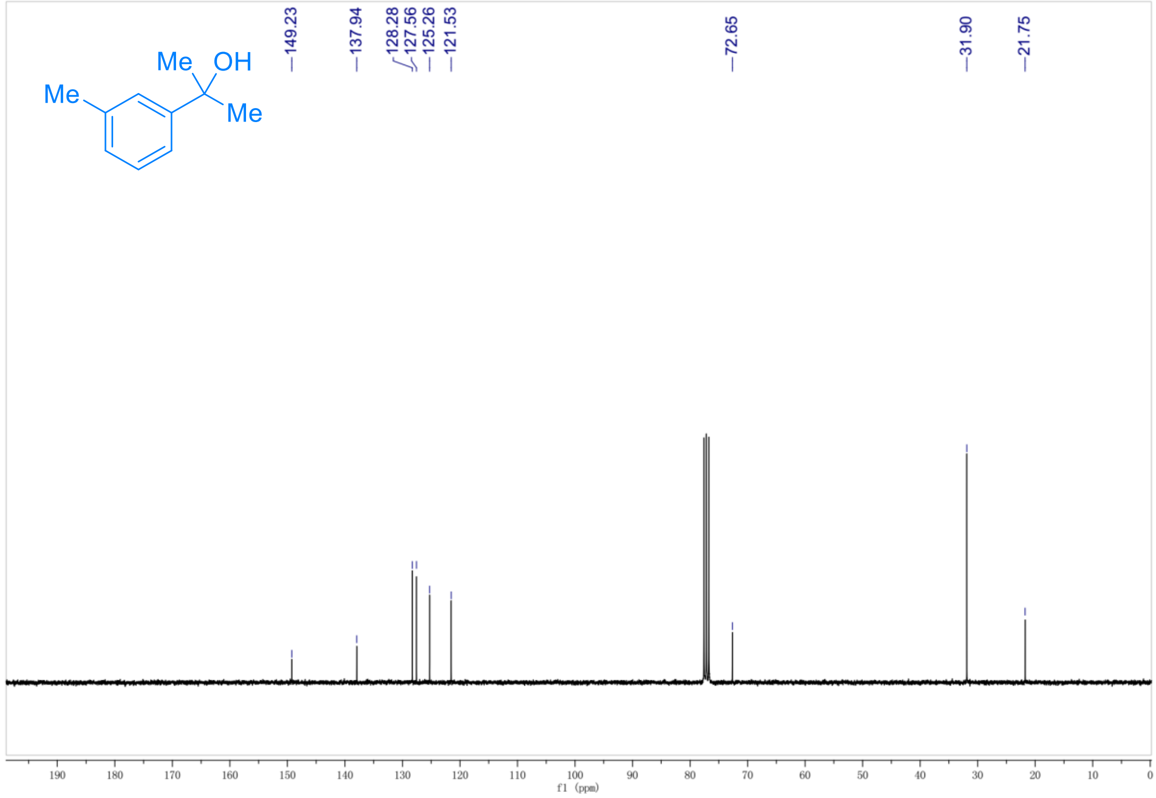

$^{13}C\{^{1}H\}$ NMR spectrum of compound **47a** in $CDCl_3$ (100 MHz).

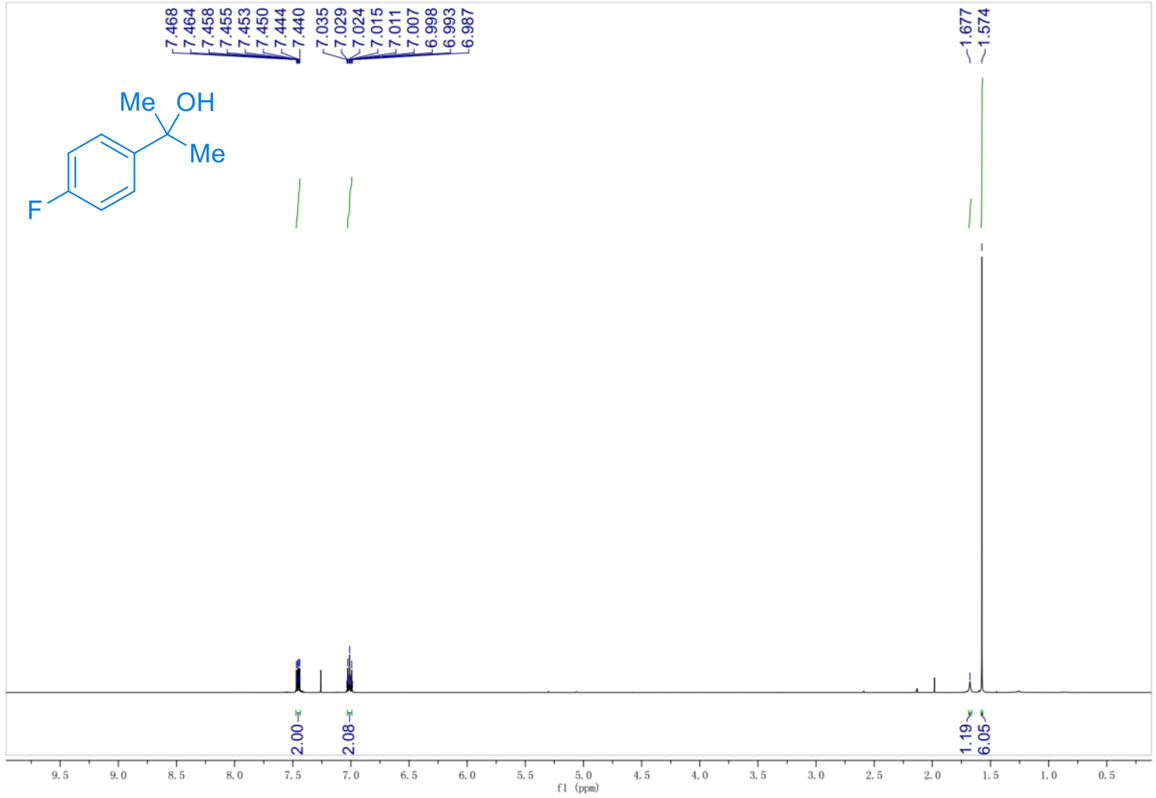

^{1}H NMR spectrum of compound **48a** in $CDCl_3$ (500 MHz).

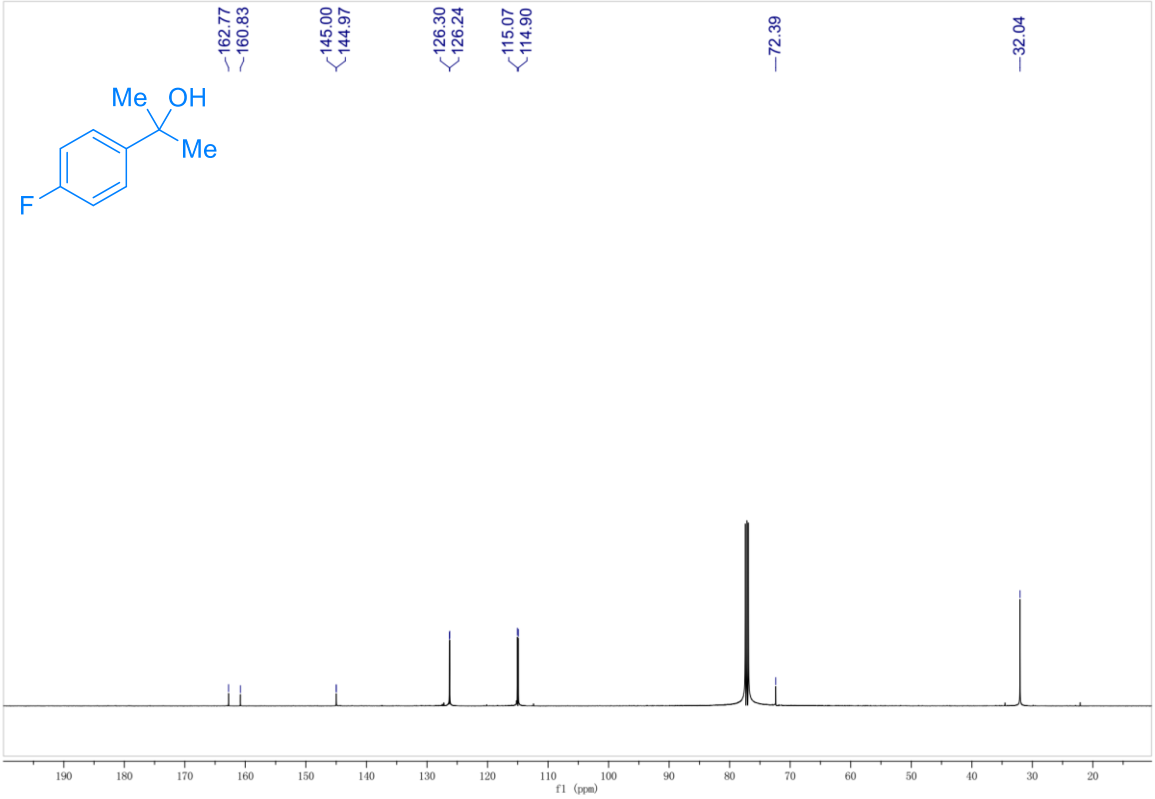

$^{13}C\{^{1}H\}$ NMR spectrum of compound **48a** in $CDCl_3$ (125 MHz).

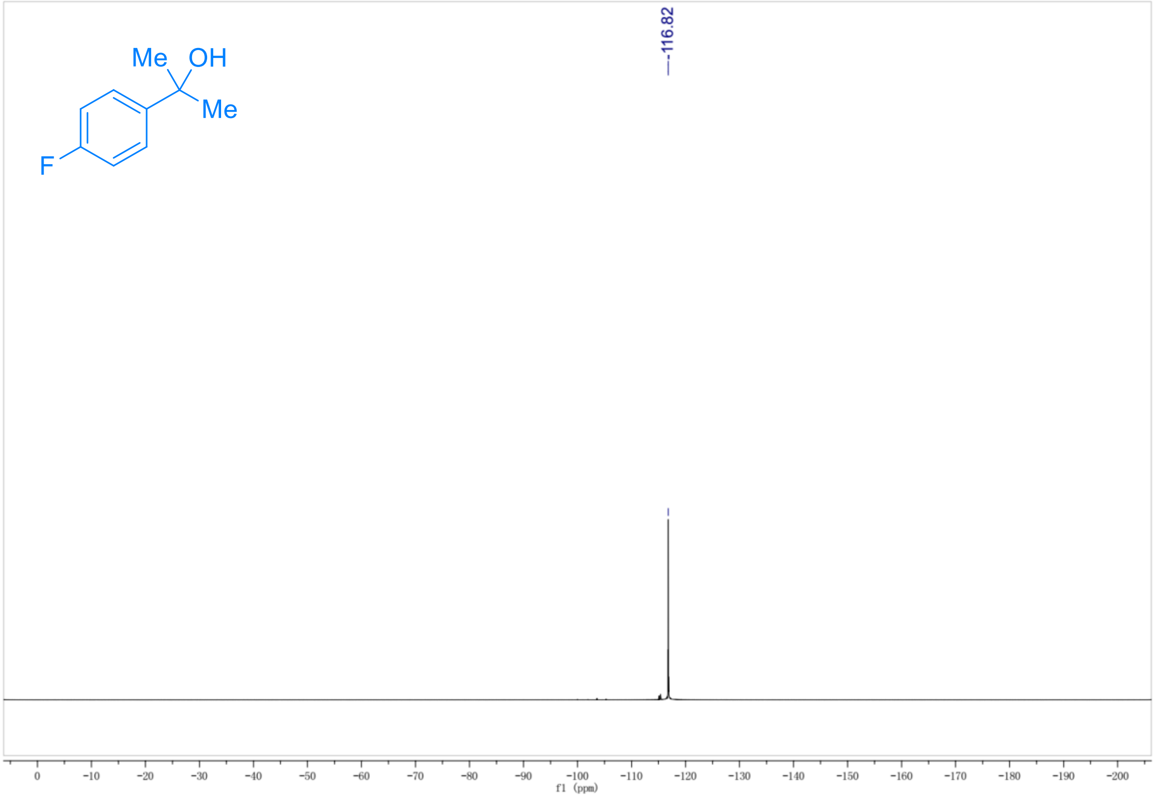

$^{19}F\{^{1}H\}$ NMR spectrum of compound **48a** in $CDCl_3$ (470 MHz).

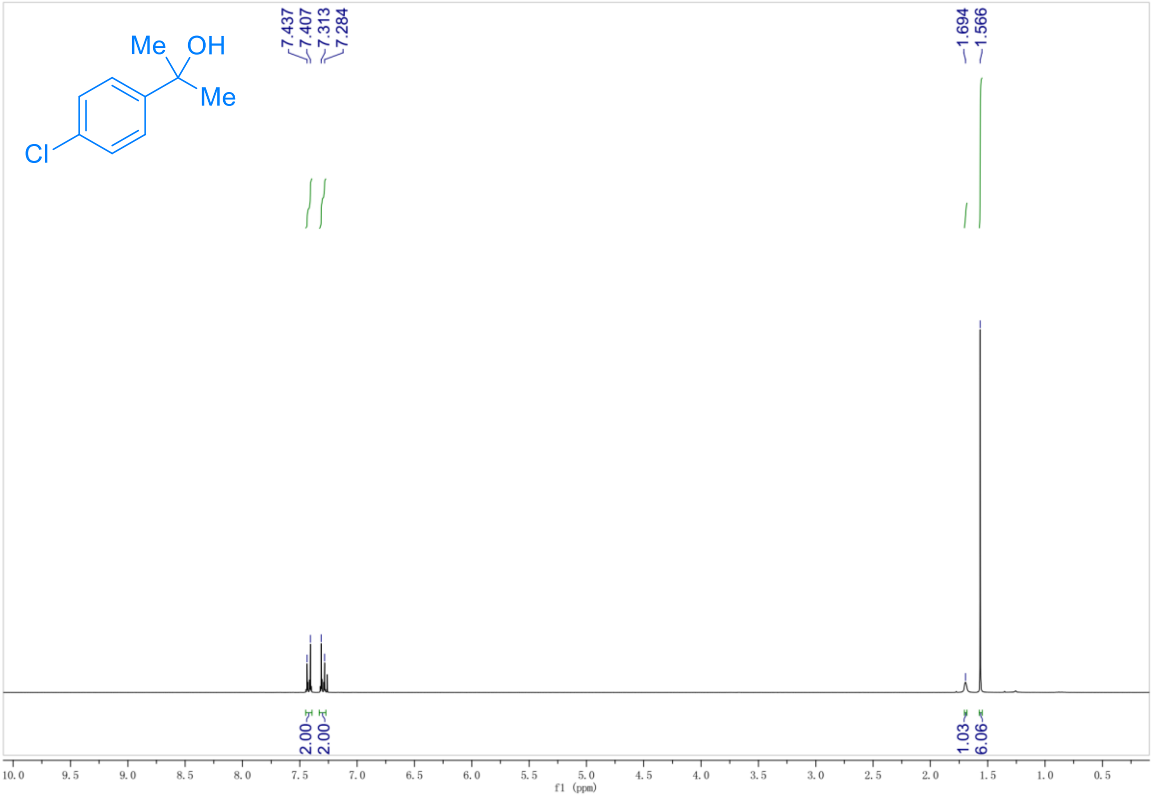

^{1}H NMR spectrum of compound **49a** in $CDCl_3$ (300 MHz).

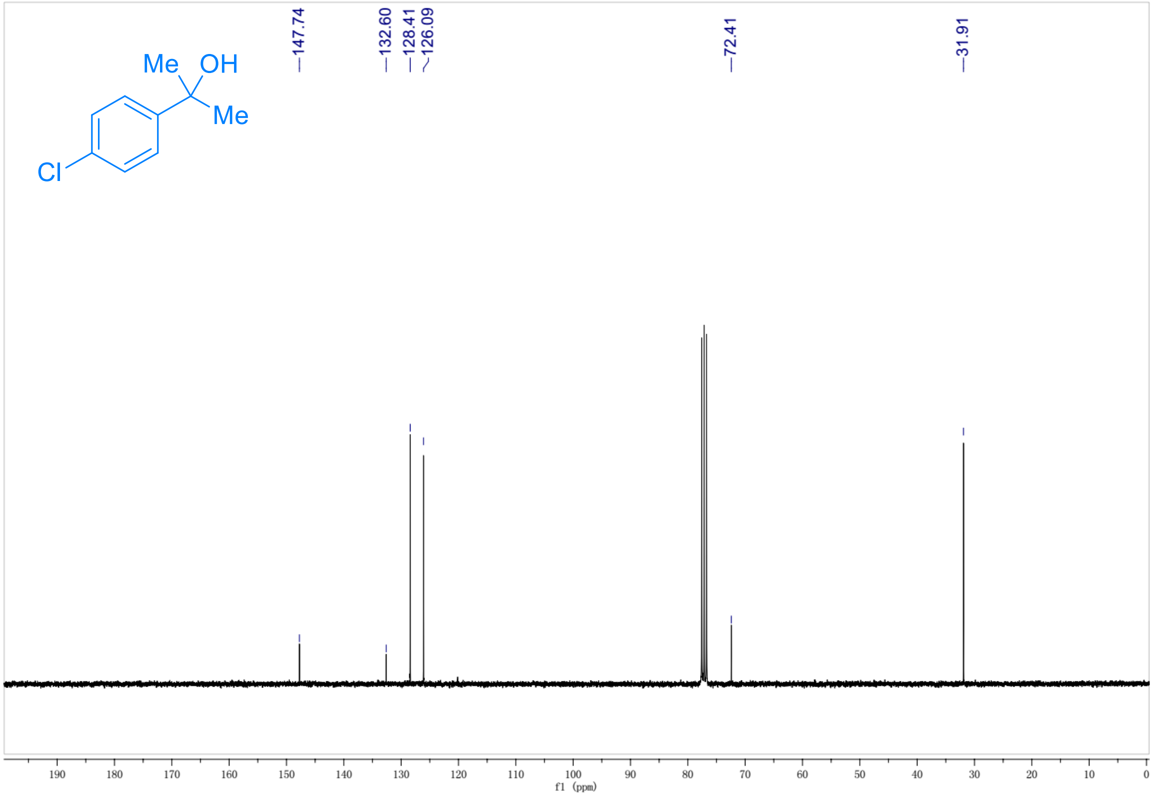

^{13}C{^{1}H} NMR spectrum of compound **49a** in $CDCl_3$ (75 MHz).

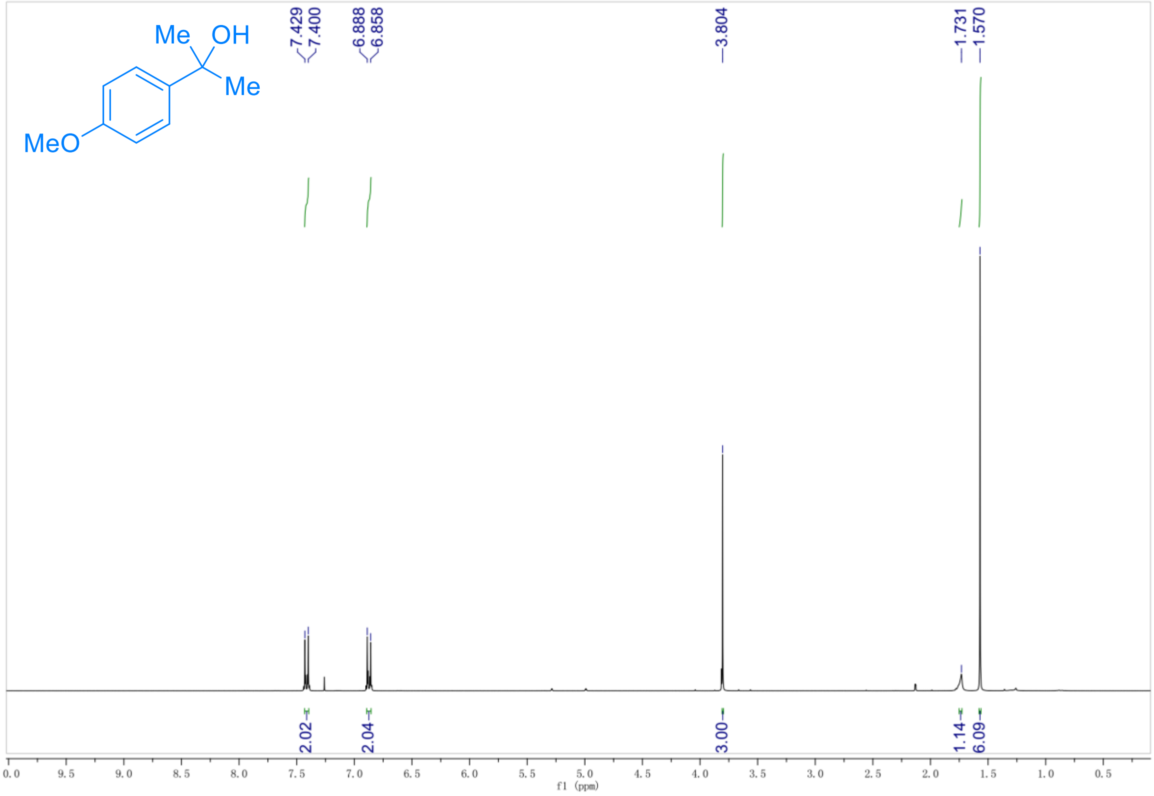

^{1}H NMR spectrum of compound **50a** in $CDCl_3$ (300 MHz).

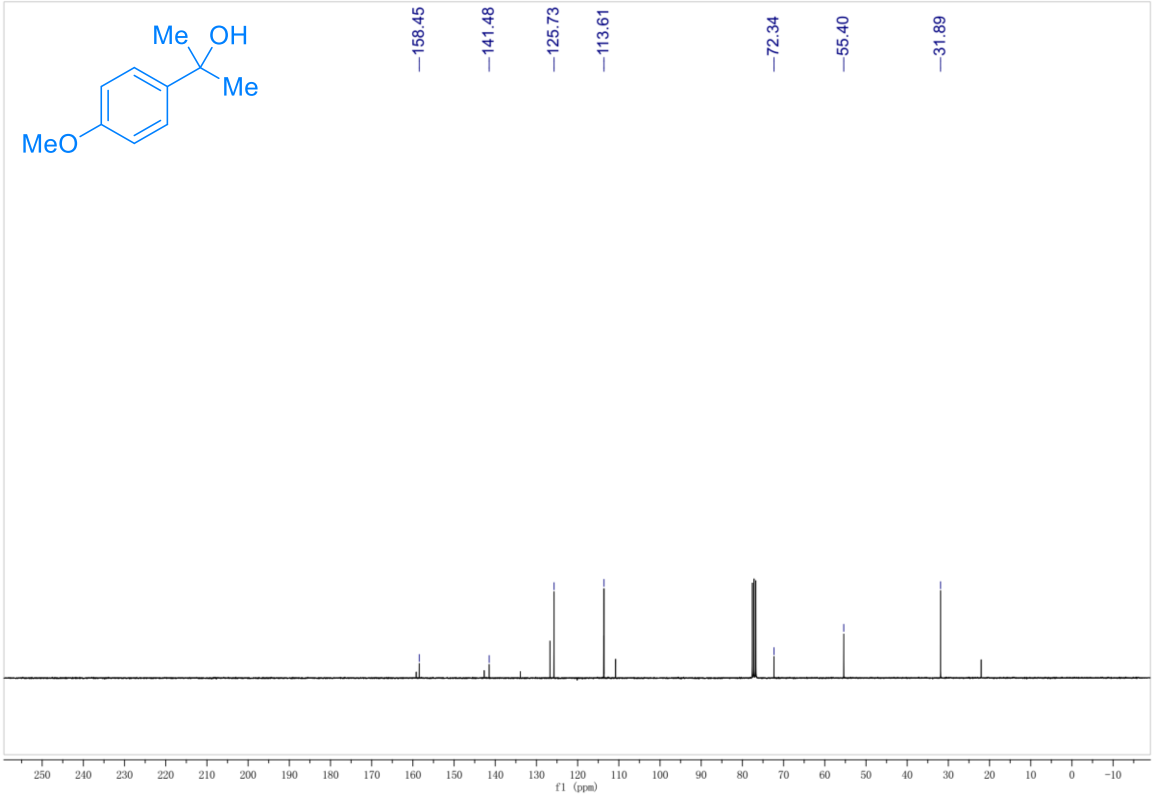

^{13}C{^{1}H} NMR spectrum of compound **50a** in $CDCl_3$ (75 MHz).

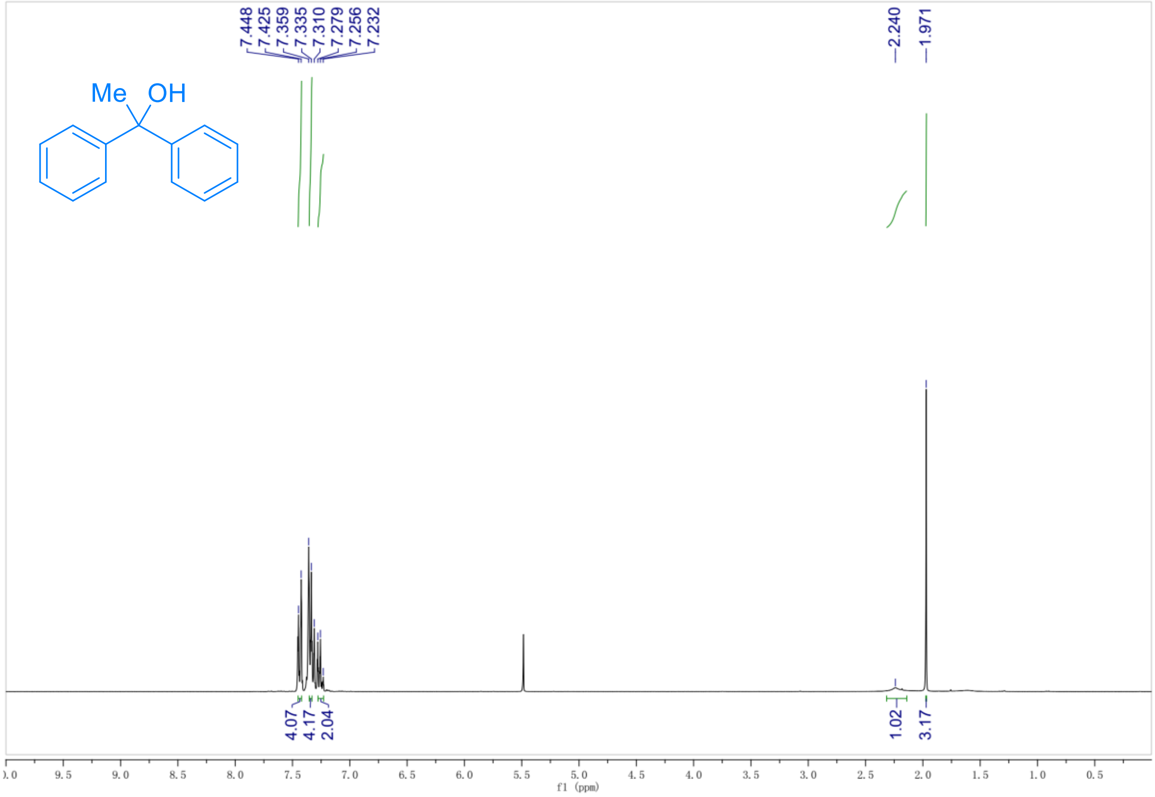

^{1}H NMR spectrum of compound **51a** in $CDCl_3$ (300 MHz).

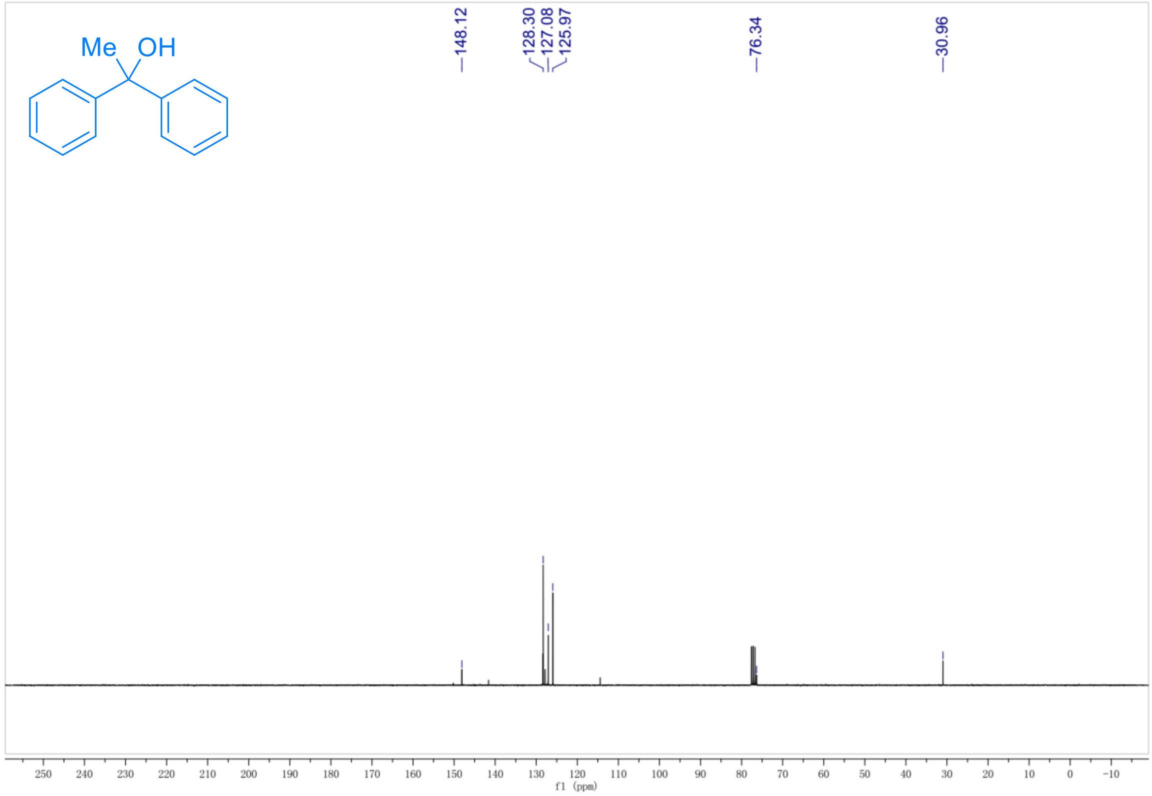

^{13}C{^{1}H} NMR spectrum of compound **51a** in $CDCl_3$ (75 MHz).

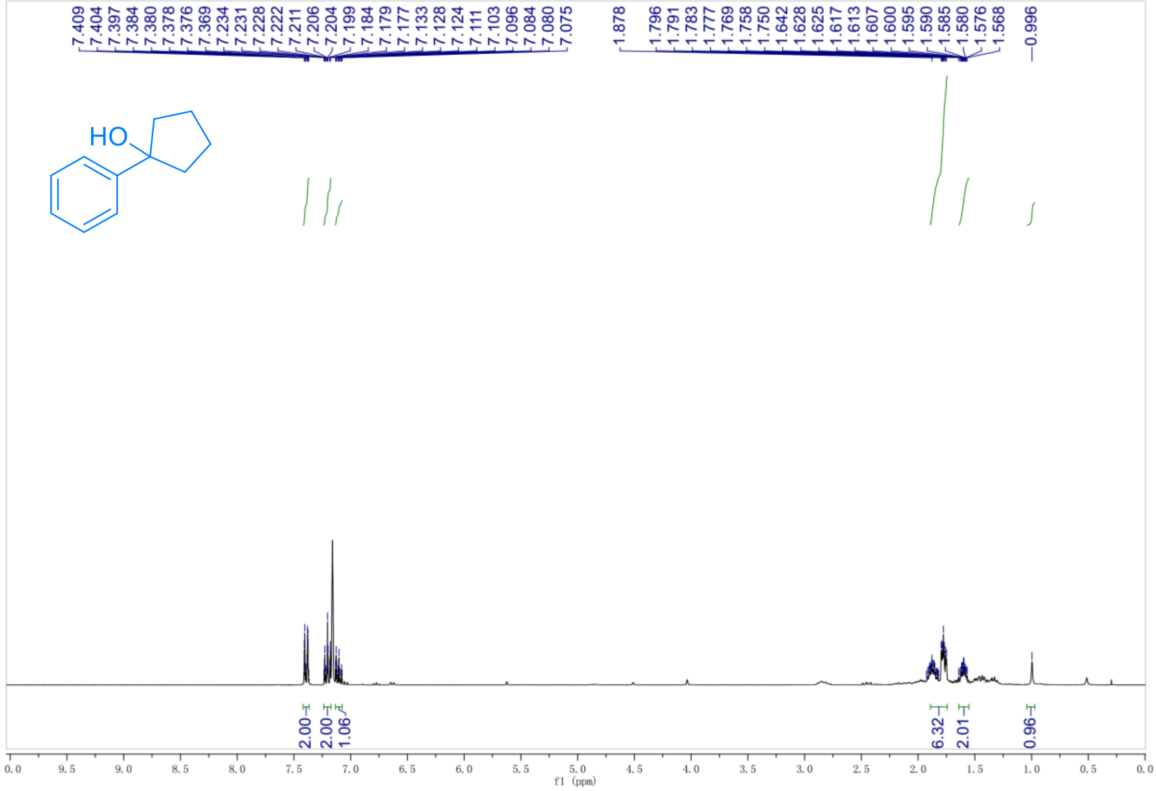

^{1}H NMR spectrum of compound **52a** in $CDCl_3$ (300 MHz).

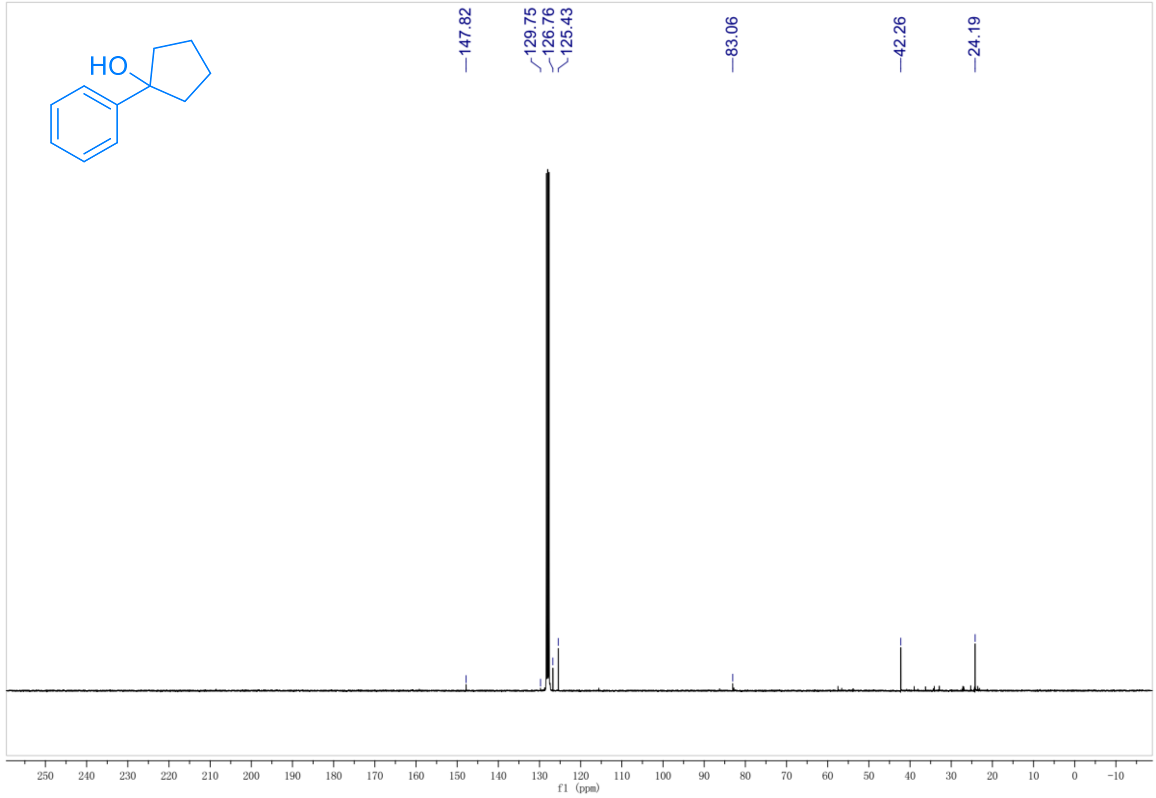

^{13}C{^{1}H} NMR spectrum of compound **52a** in $CDCl_3$ (75 MHz).

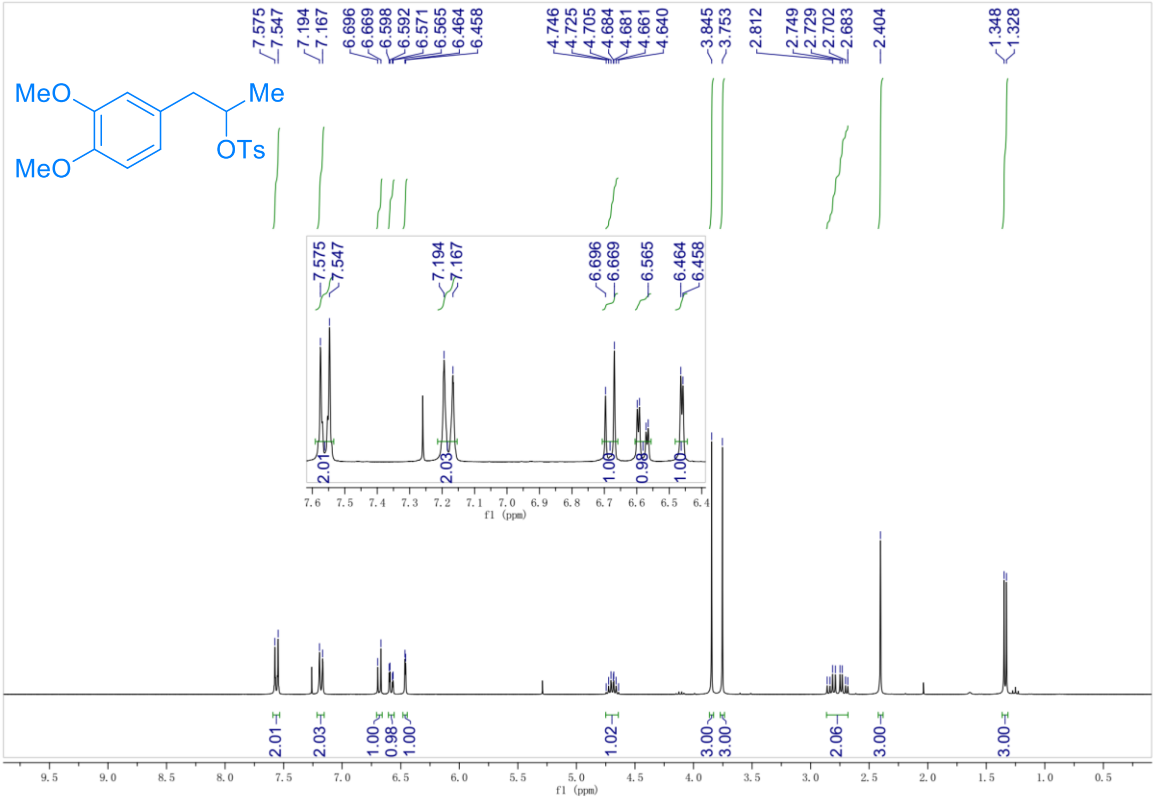

^{1}H NMR spectrum of compound **55a** in $CDCl_3$ (300 MHz).

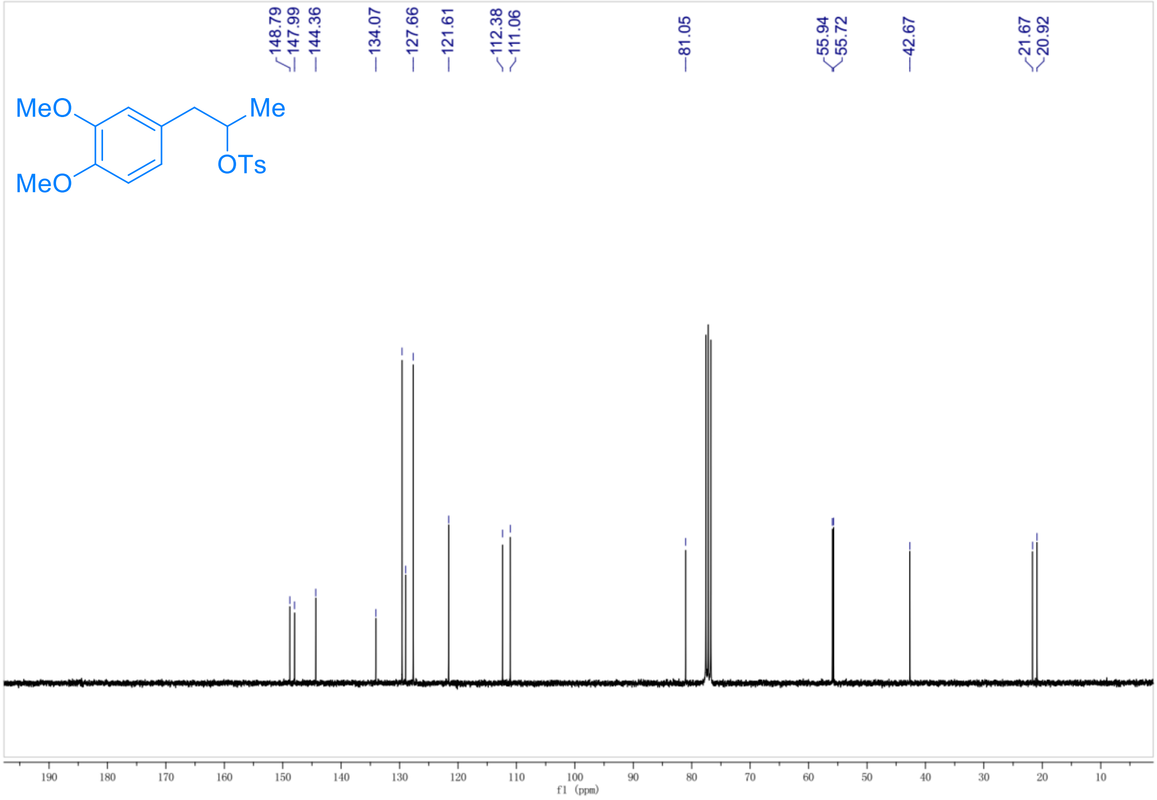

^{13}C{^{1}H} NMR spectrum of compound **55a** in $CDCl_3$ (100 MHz).

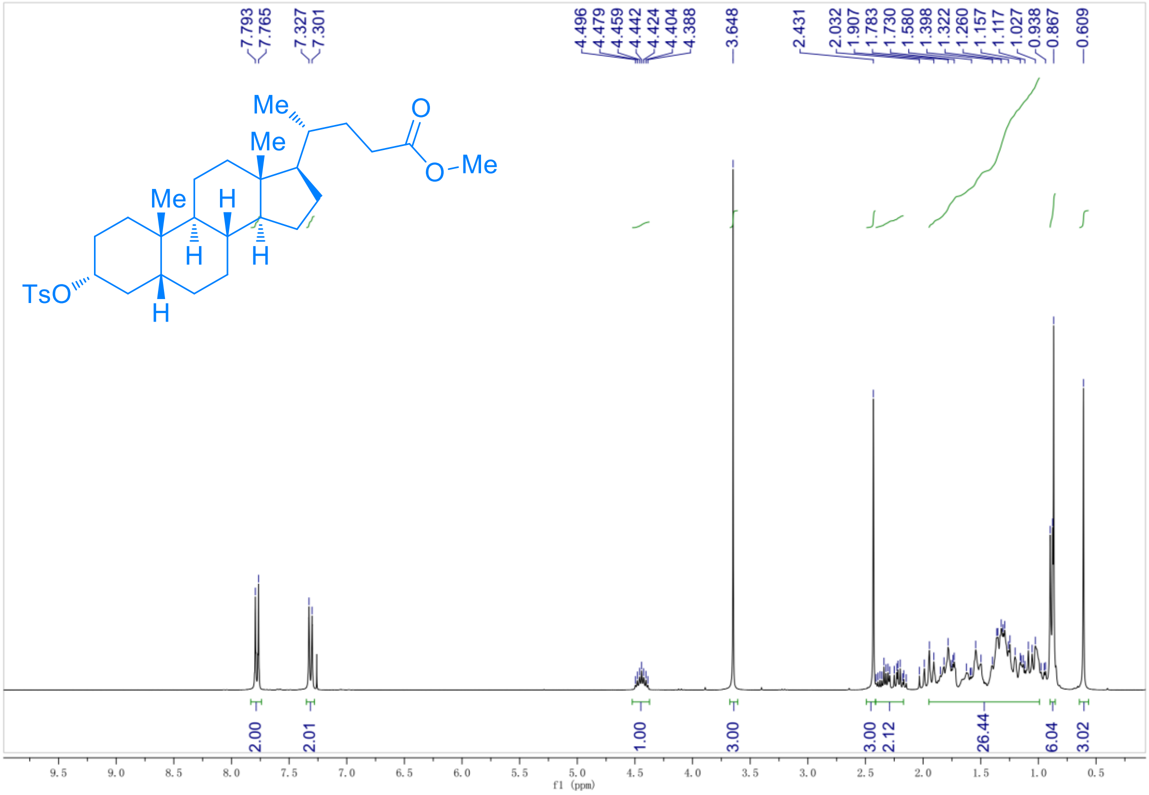

^{1}H NMR spectrum of compound **56a** in $CDCl_3$ (300 MHz).

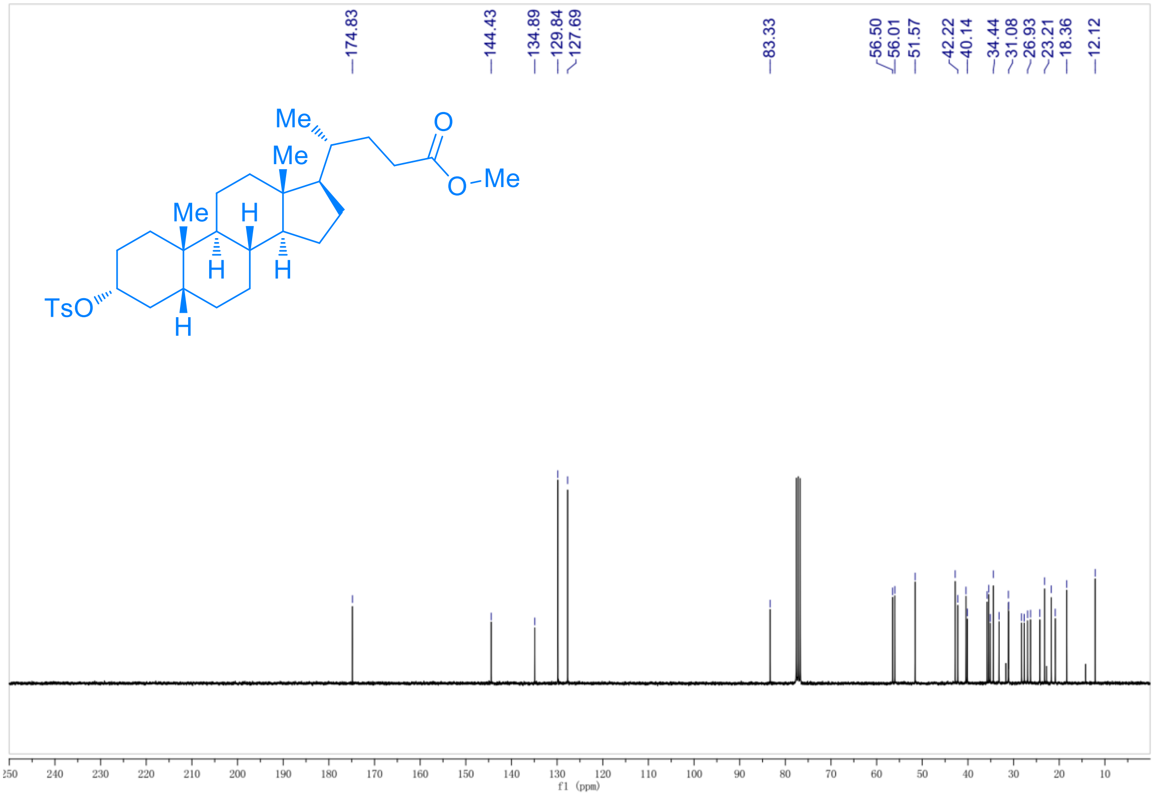

^{13}C{^{1}H} NMR spectrum of compound **56a** in $CDCl_3$ (75 MHz).

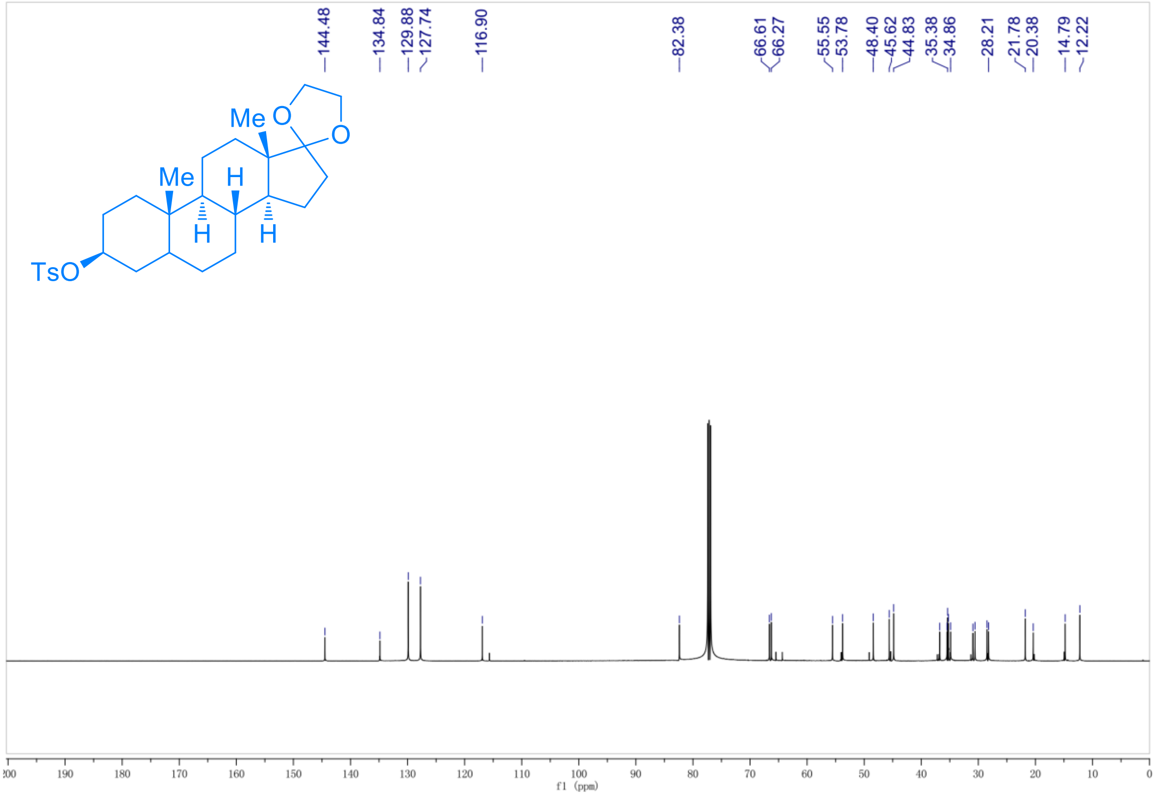

^{1}H NMR spectrum of compound **57a** in $CDCl_3$ (500 MHz).

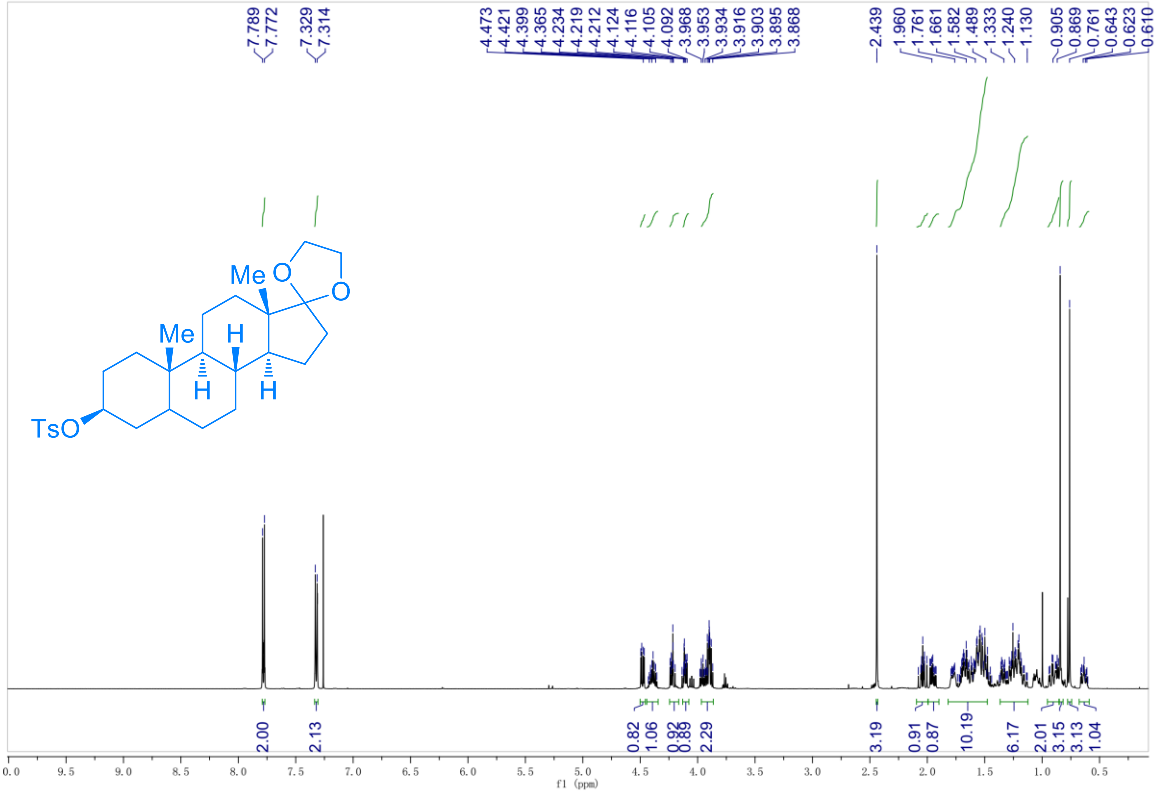

^{13}C{^{1}H} NMR spectrum of compound **57a** in $CDCl_3$ (125 MHz).

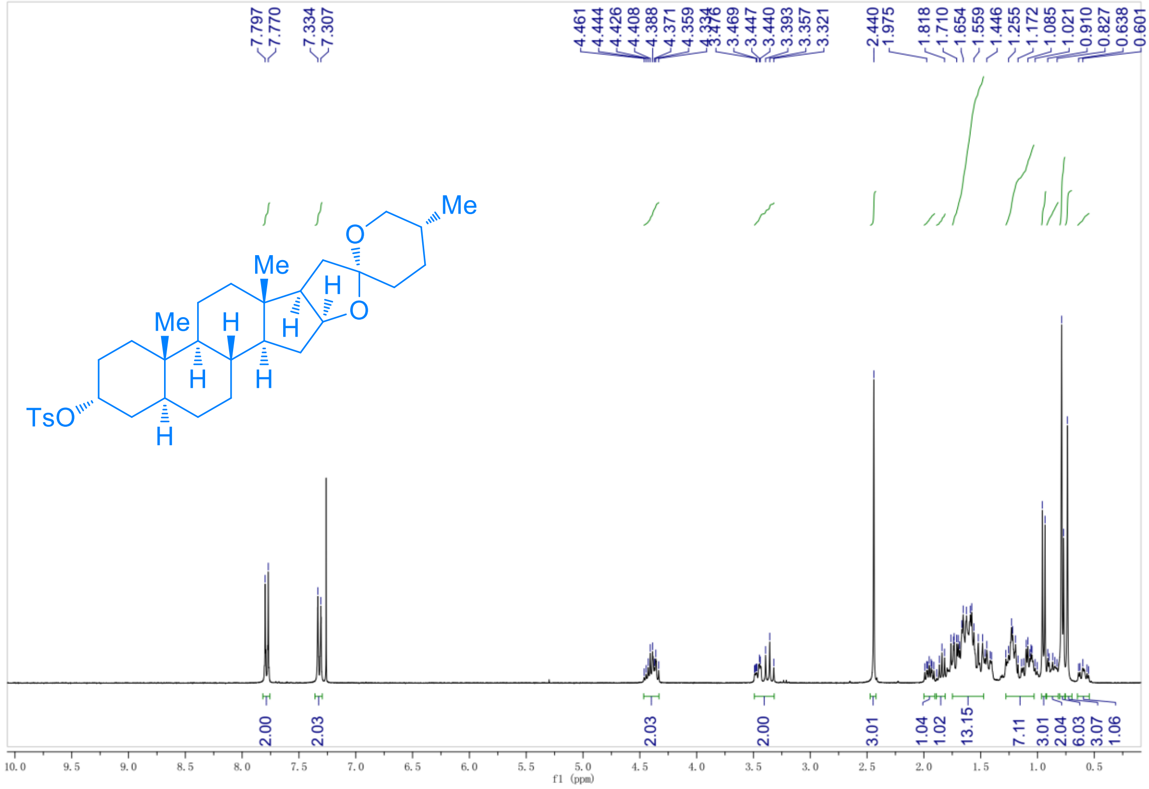

^{1}H NMR spectrum of compound **58a** in $CDCl_3$ (300 MHz).

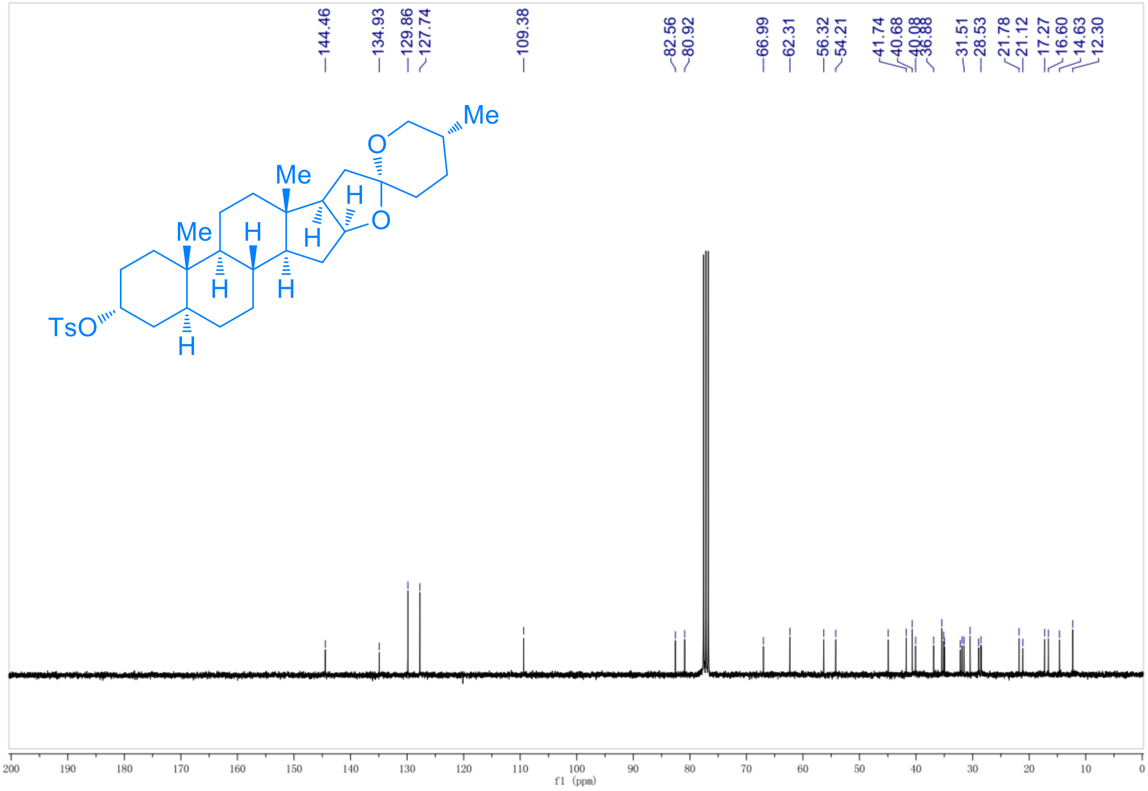

^{13}C{^{1}H} NMR spectrum of compound **58a** in $CDCl_3$ (75 MHz).

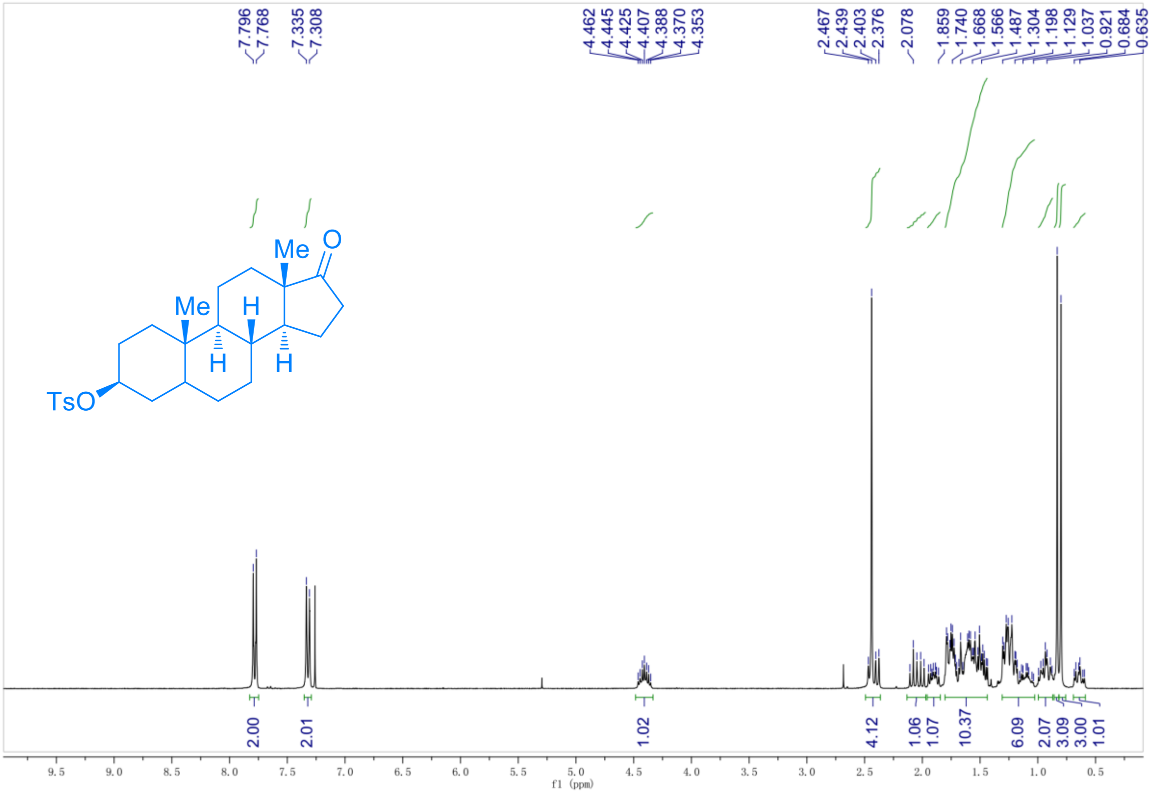

^{1}H NMR spectrum of compound **59a** in $CDCl_3$ (300 MHz).

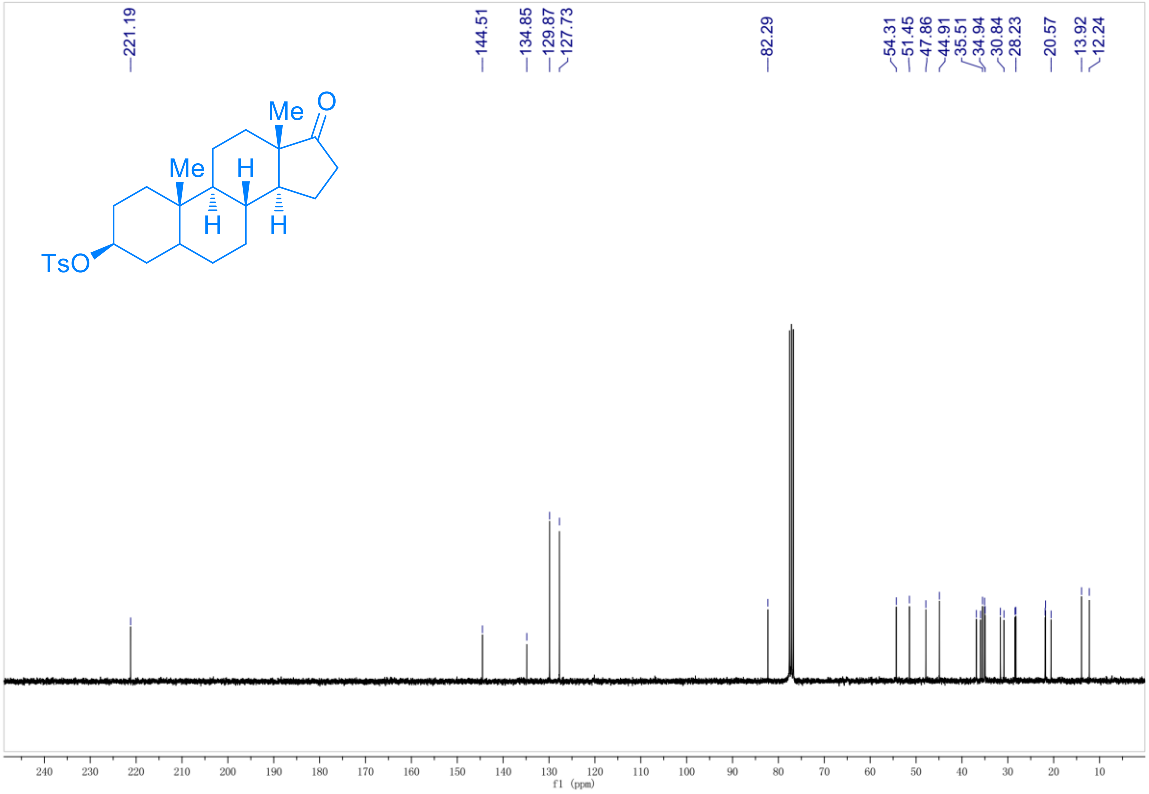

^{13}C{^{1}H} NMR spectrum of compound **59a** in $CDCl_3$ (75 MHz).

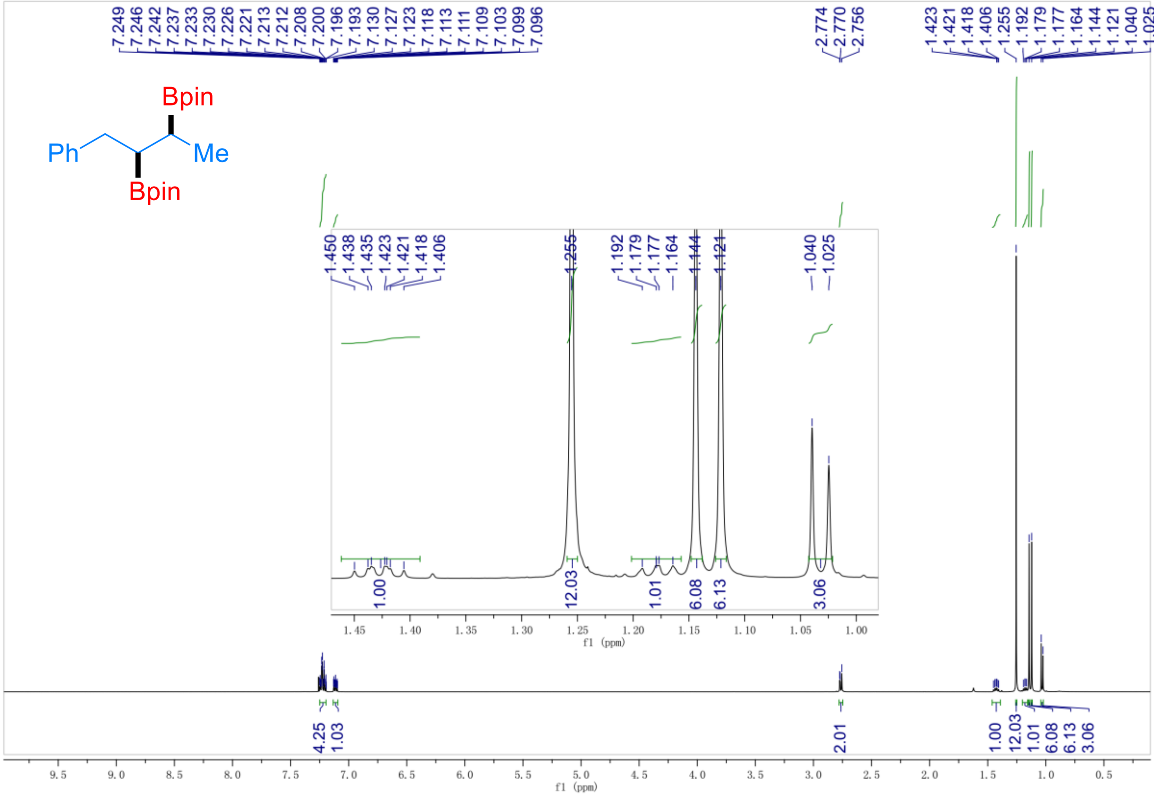

^{1}H NMR spectrum of compound **1b** in $CDCl_3$ (500 MHz).

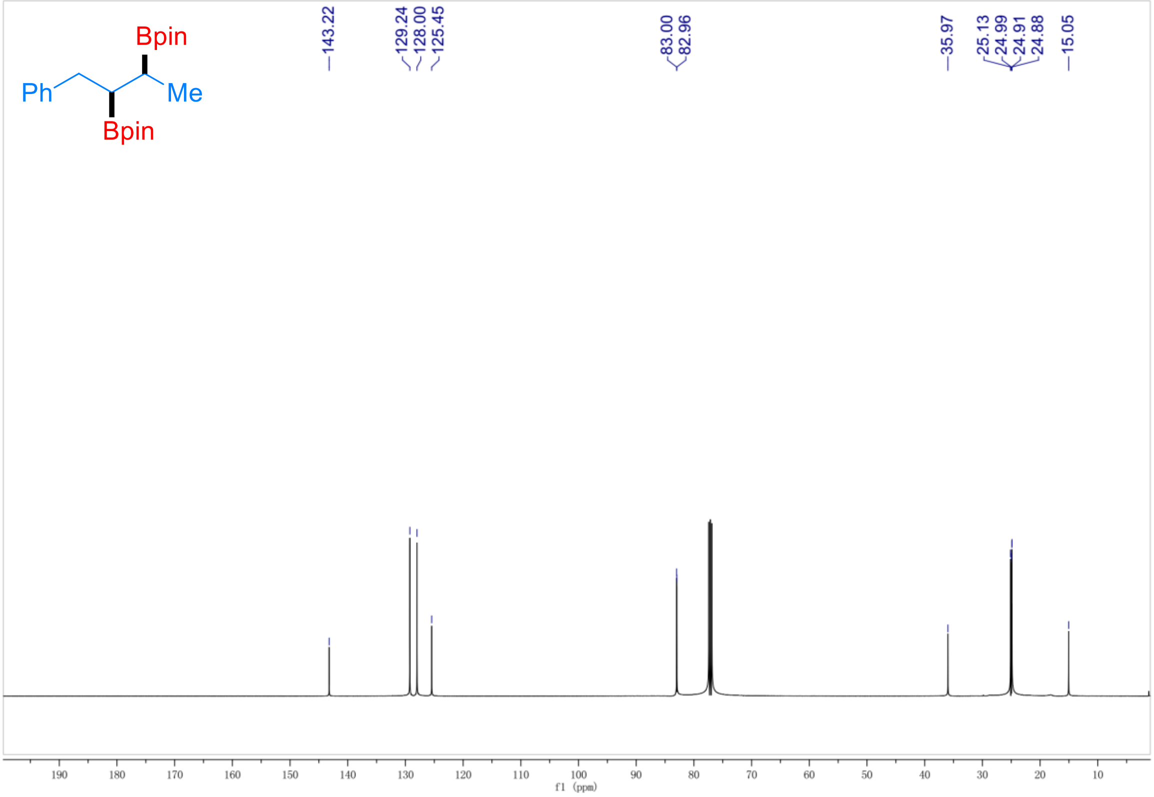

^{13}C{^{1}H} NMR spectrum of compound **1b** in $CDCl_3$ (125 MHz).

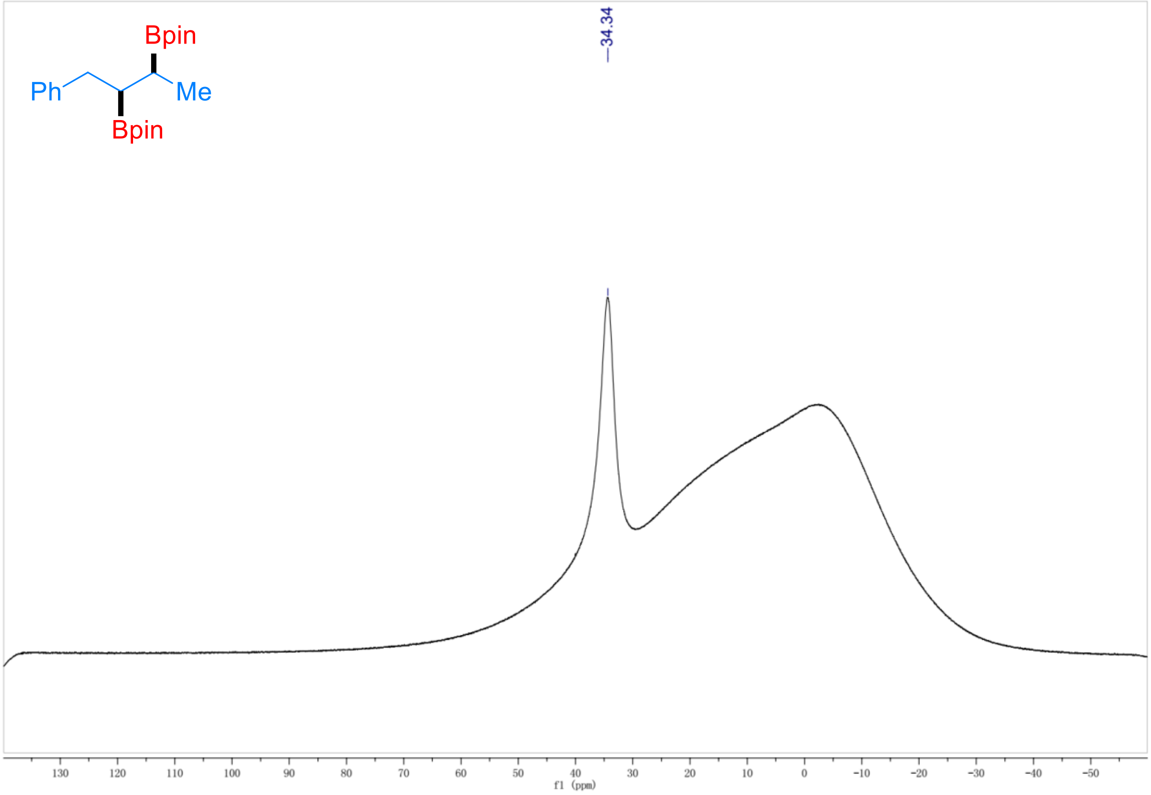

$^{11}B\{^{1}H\}$ NMR spectrum of compound **1b** in $CDCl_3$ (160 MHz).

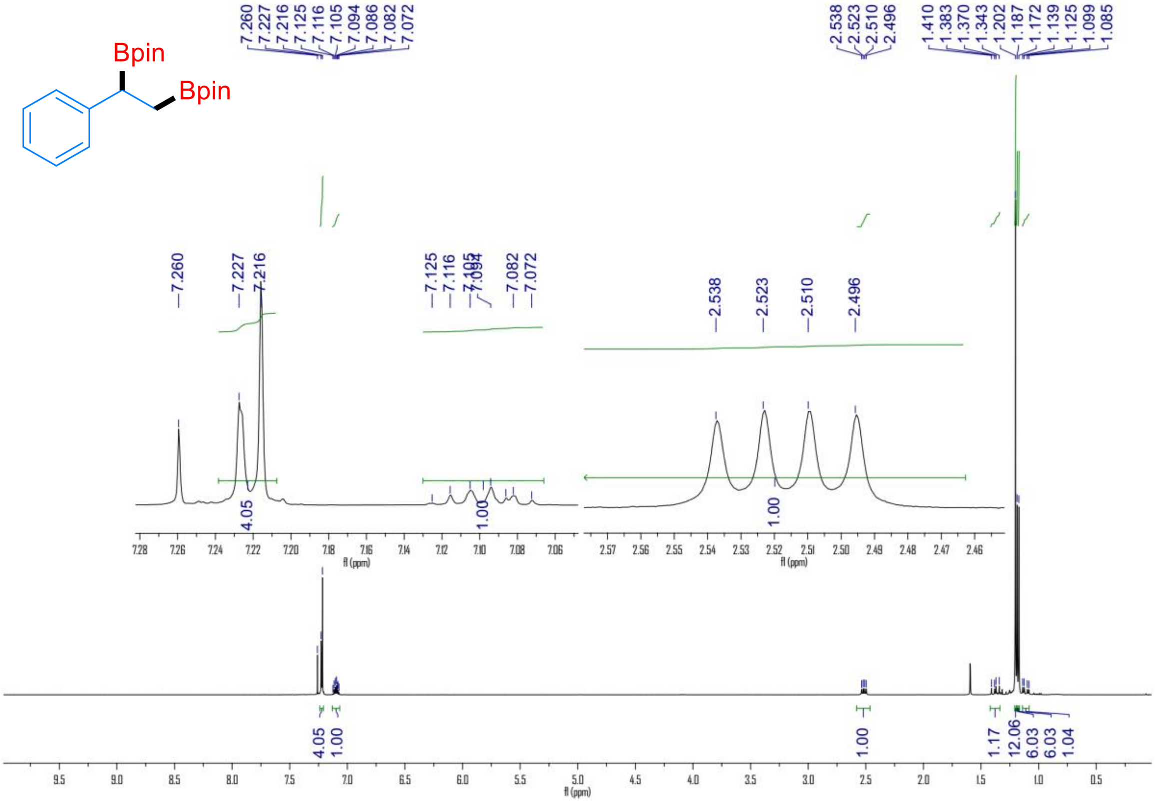

^{1}H NMR spectrum of compound **2b** in $CDCl_3$ (500 MHz).

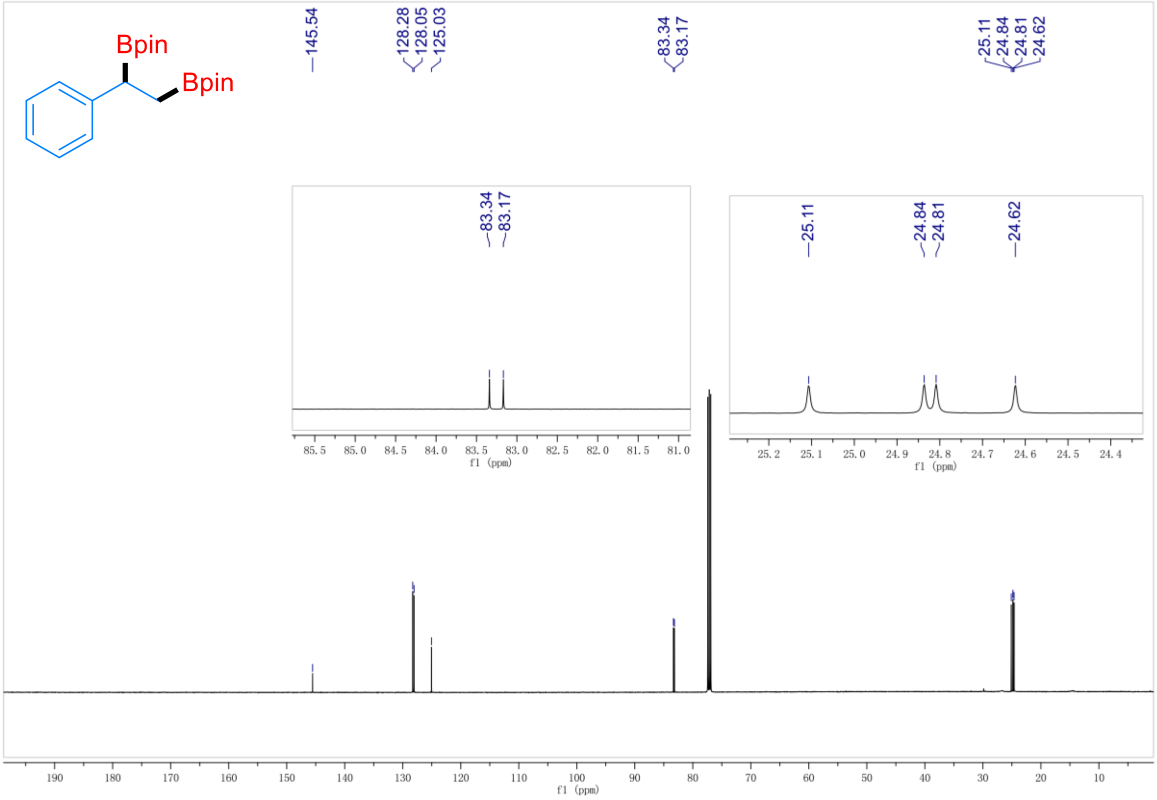

$^{13}C\{^{1}H\}$ NMR spectrum of compound **2b** in $CDCl_3$ (125 MHz).

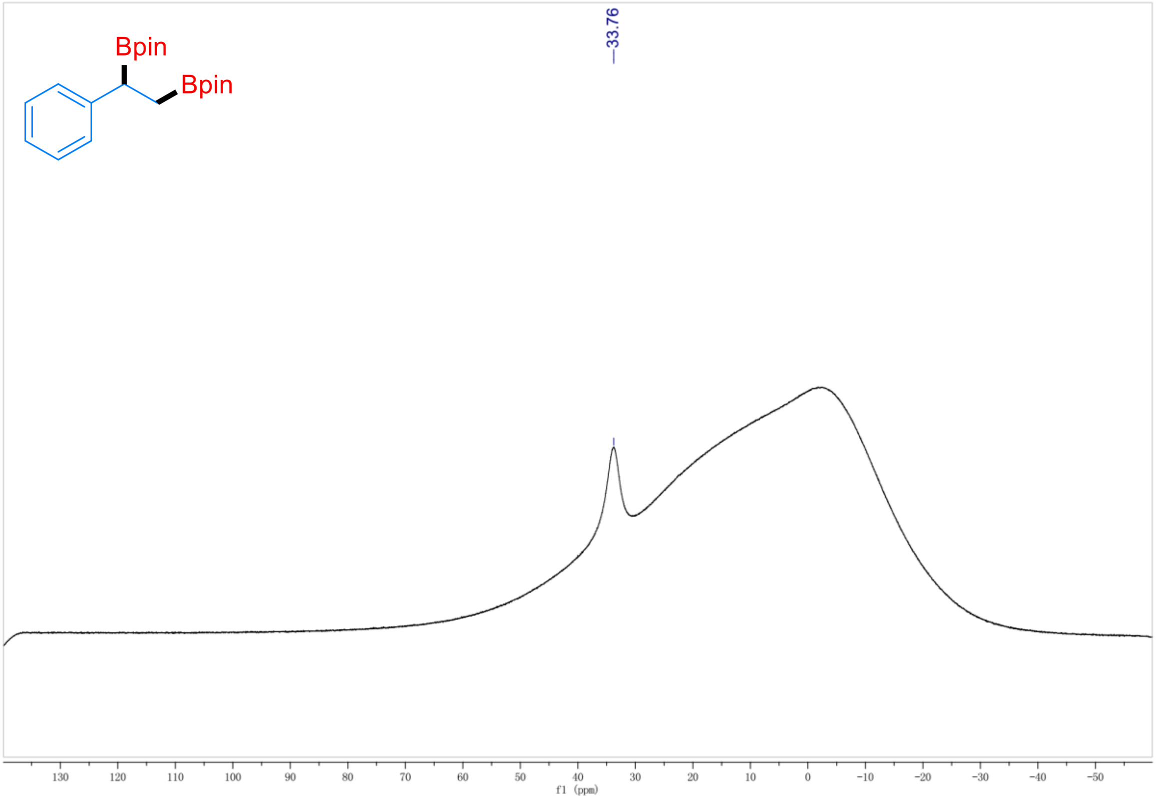

$^{11}B\{^{1}H\}$ NMR spectrum of compound **2b** in $CDCl_3$ (160 MHz).

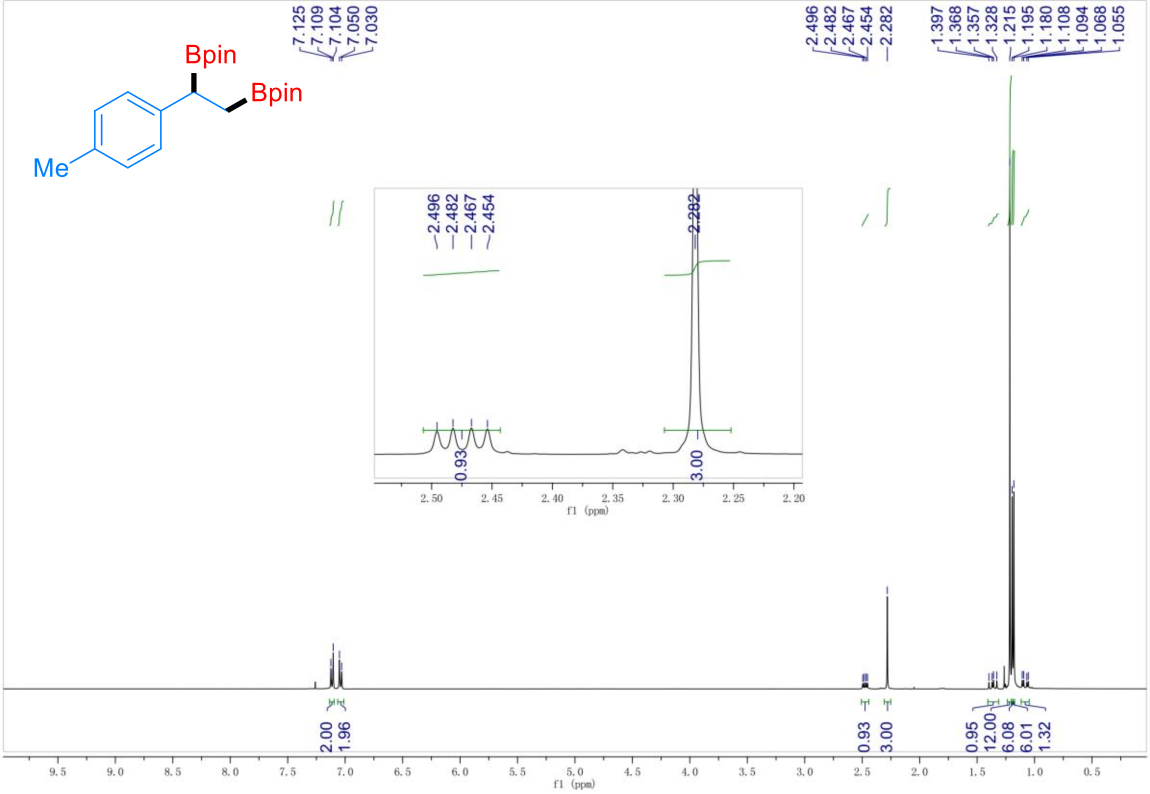

^{1}H NMR spectrum of compound **3b** in $CDCl_3$ (400 MHz).

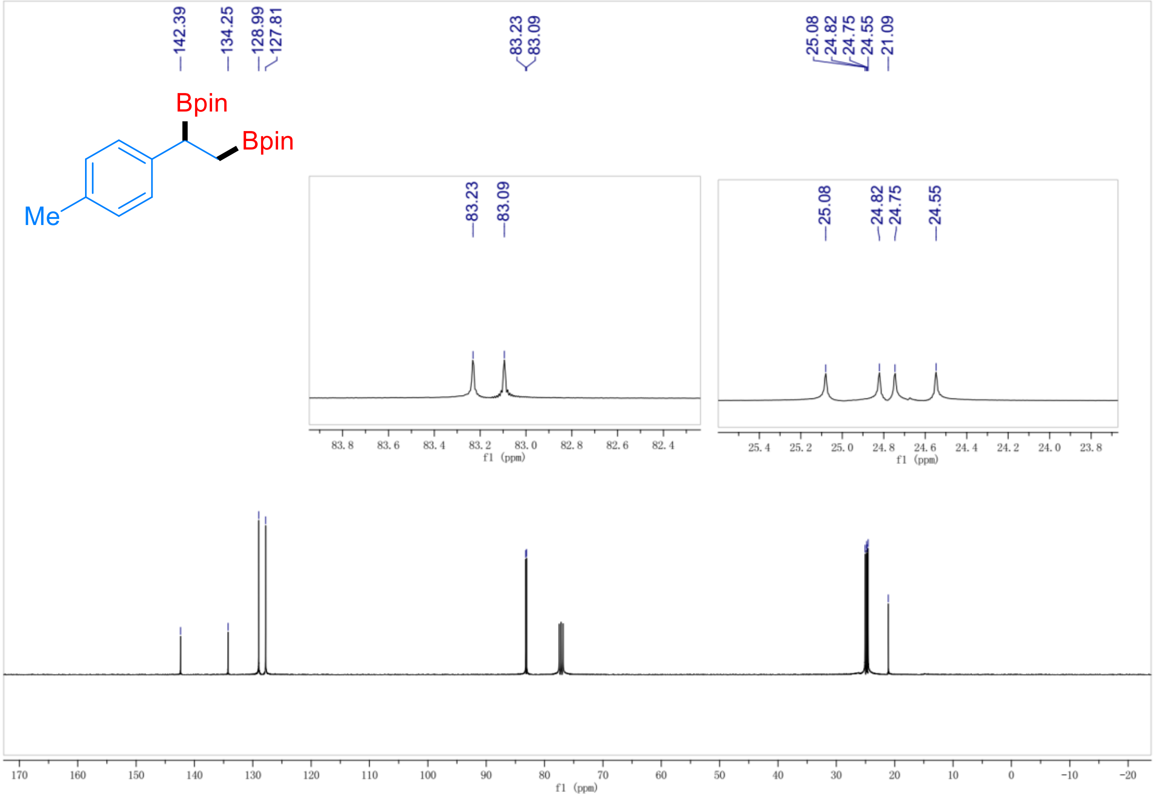

$^{13}C\{^{1}H\}$ NMR spectrum of compound **3b** in $CDCl_3$ (125 MHz).

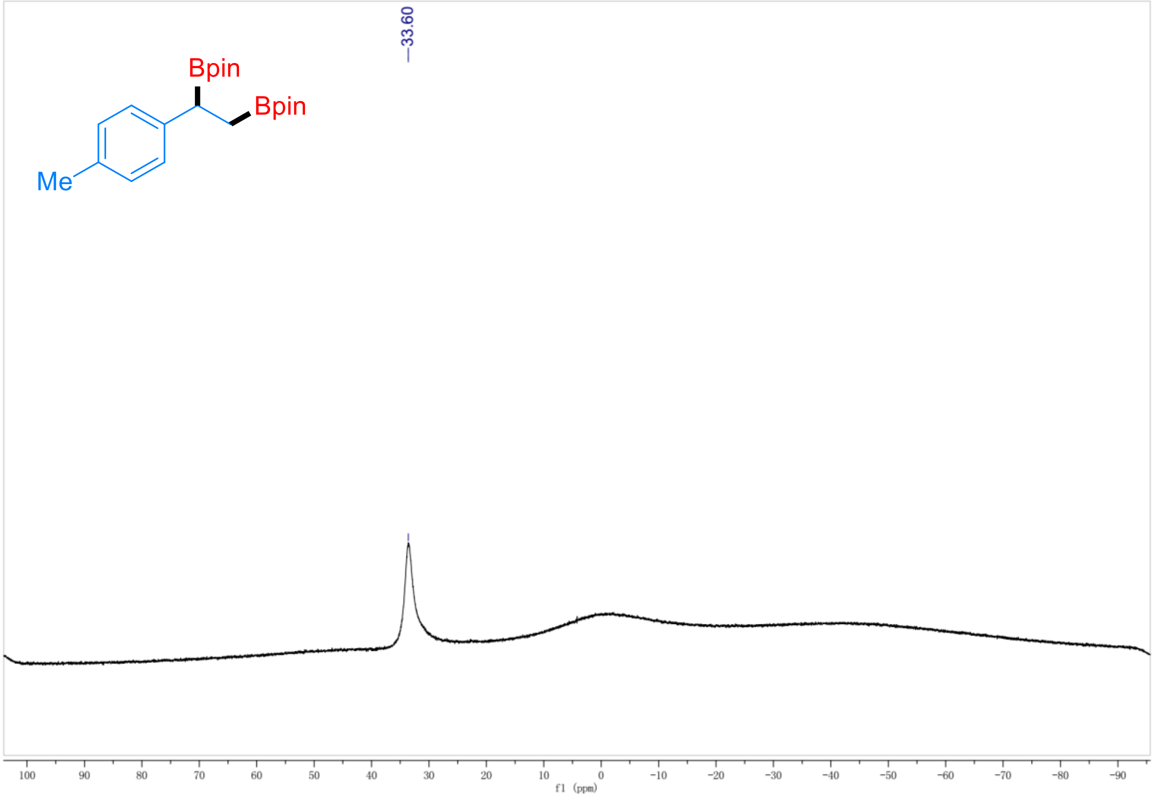

$^{11}B\{^{1}H\}$ NMR spectrum of compound **3b** in $CDCl_3$ (128 MHz).

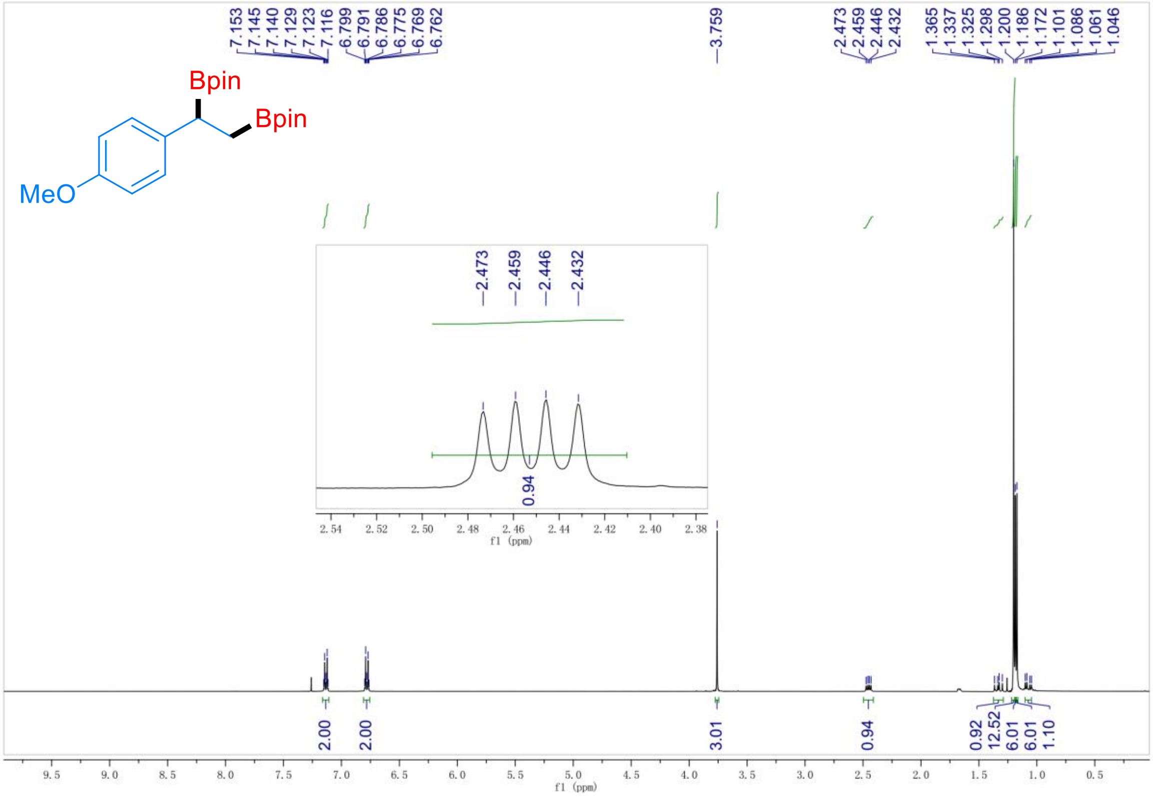

^{1}H NMR spectrum of compound **4b** in $CDCl_3$ (400 MHz).

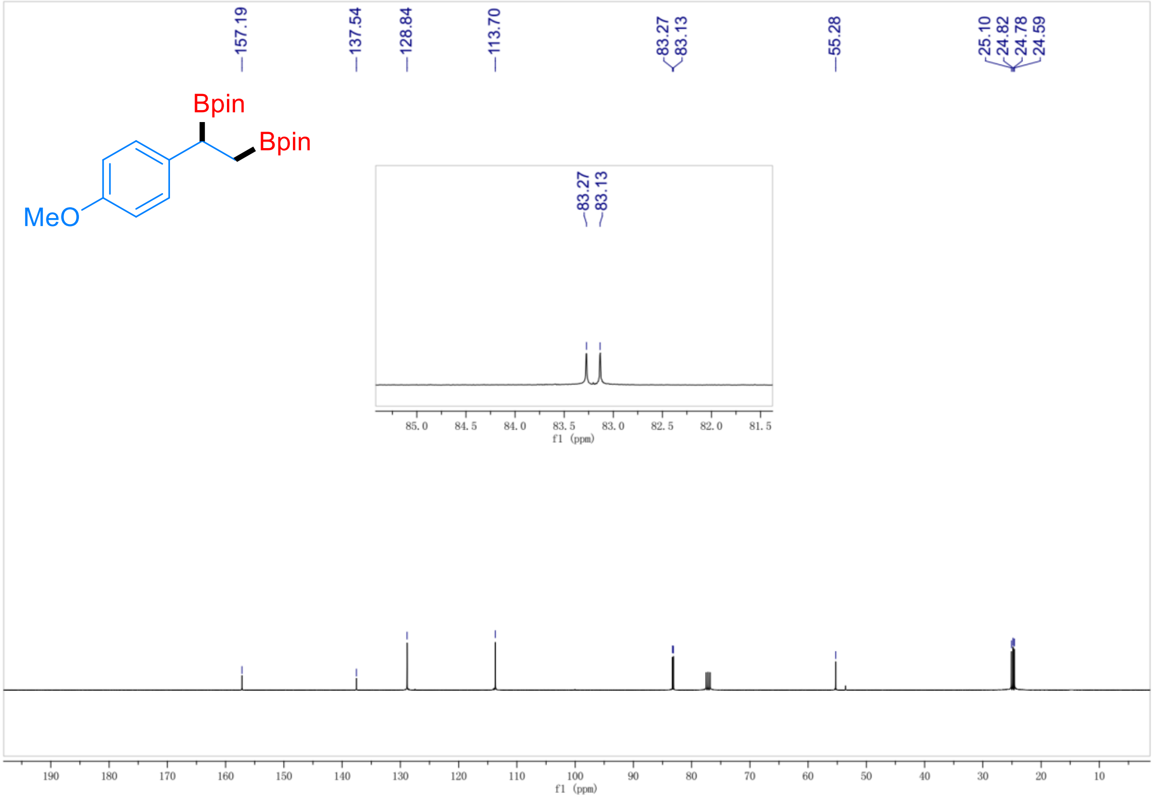

$^{13}C\{^{1}H\}$ NMR spectrum of compound **4b** in $CDCl_3$ (100 MHz).

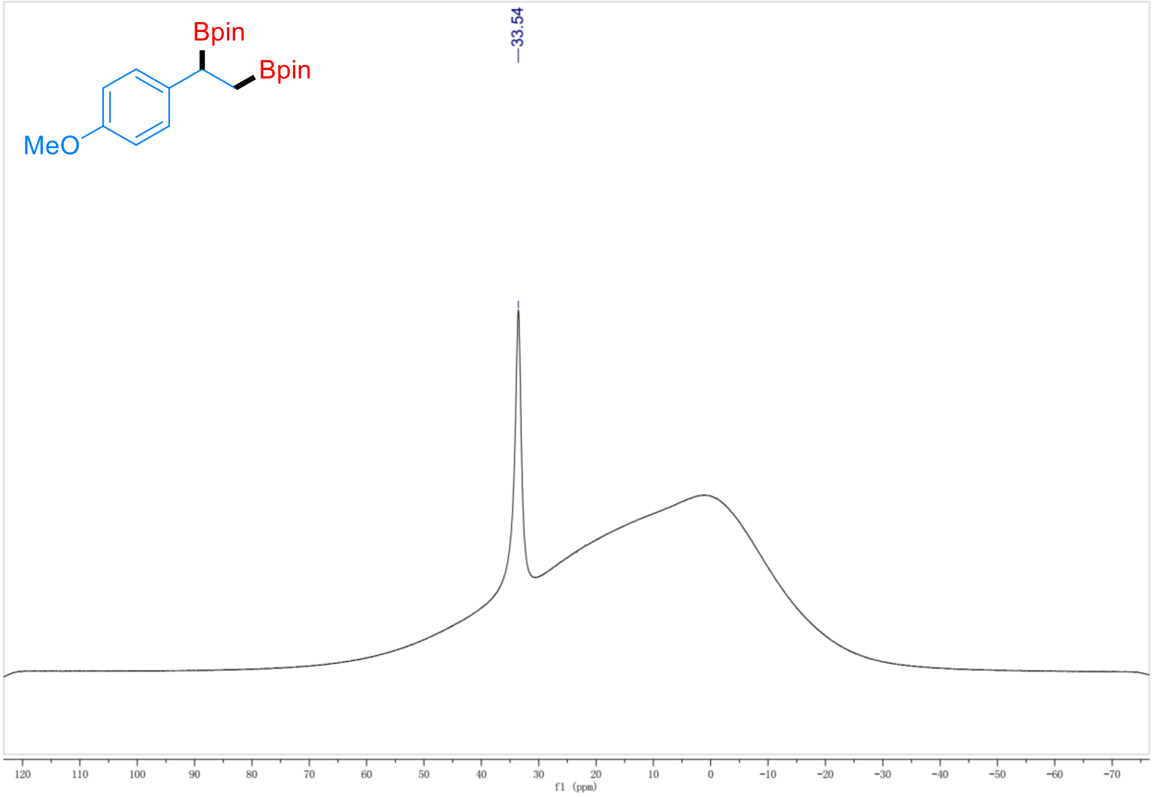

$^{11}B\{^{1}H\}$ NMR spectrum of compound **4b** in $CDCl_3$ (128 MHz).

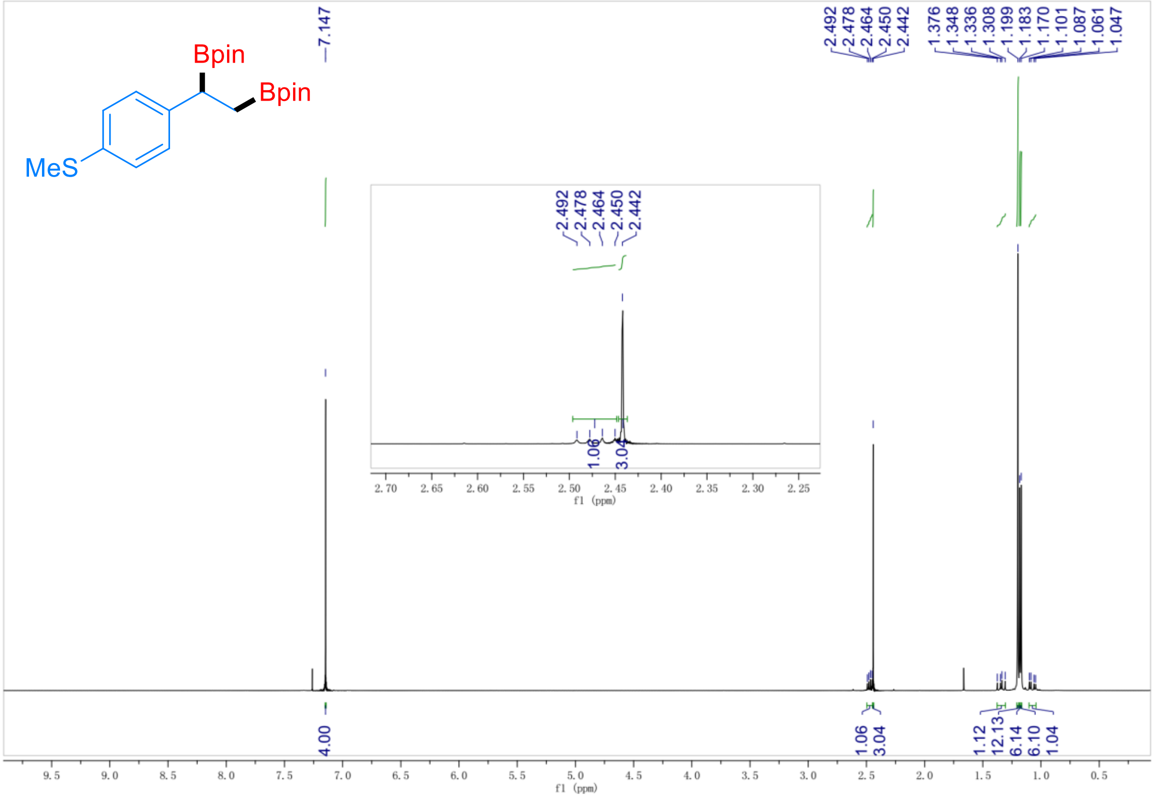

^{1}H NMR spectrum of compound **5b** in $CDCl_3$ (400 MHz).

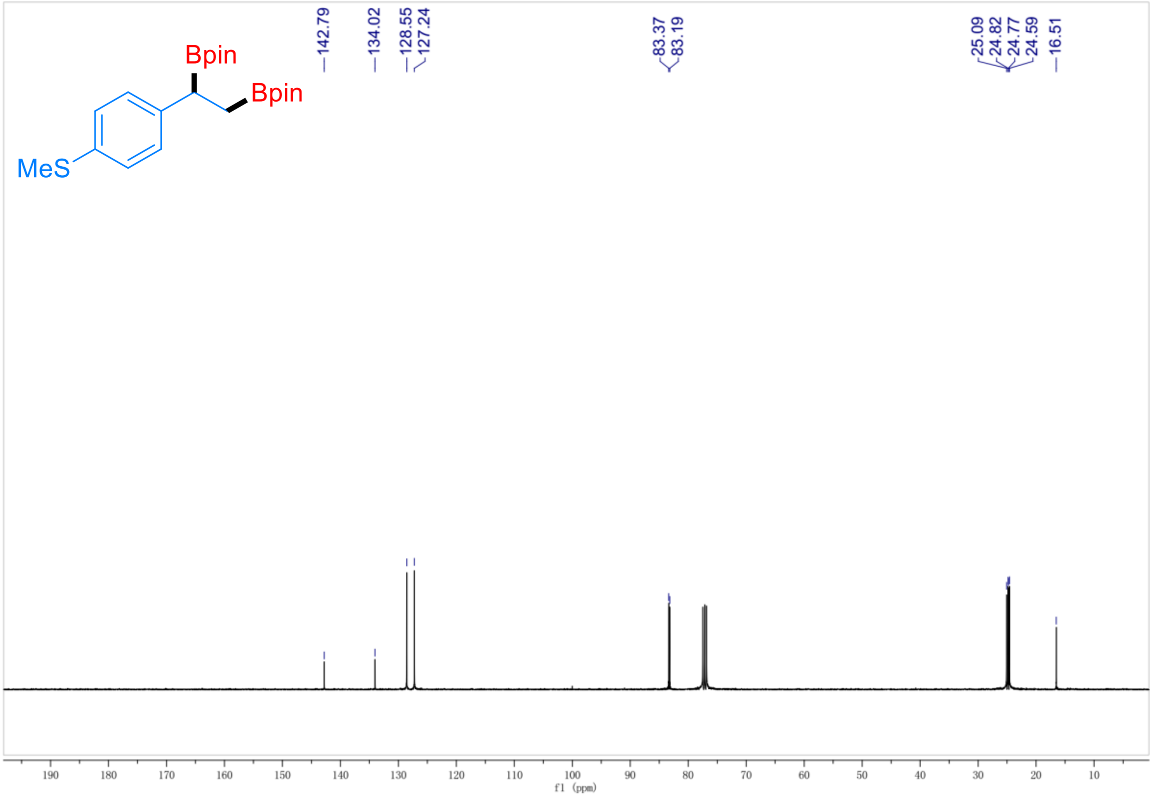

$^{13}C\{^{1}H\}$ NMR spectrum of compound **5b** in $CDCl_3$ (100 MHz).

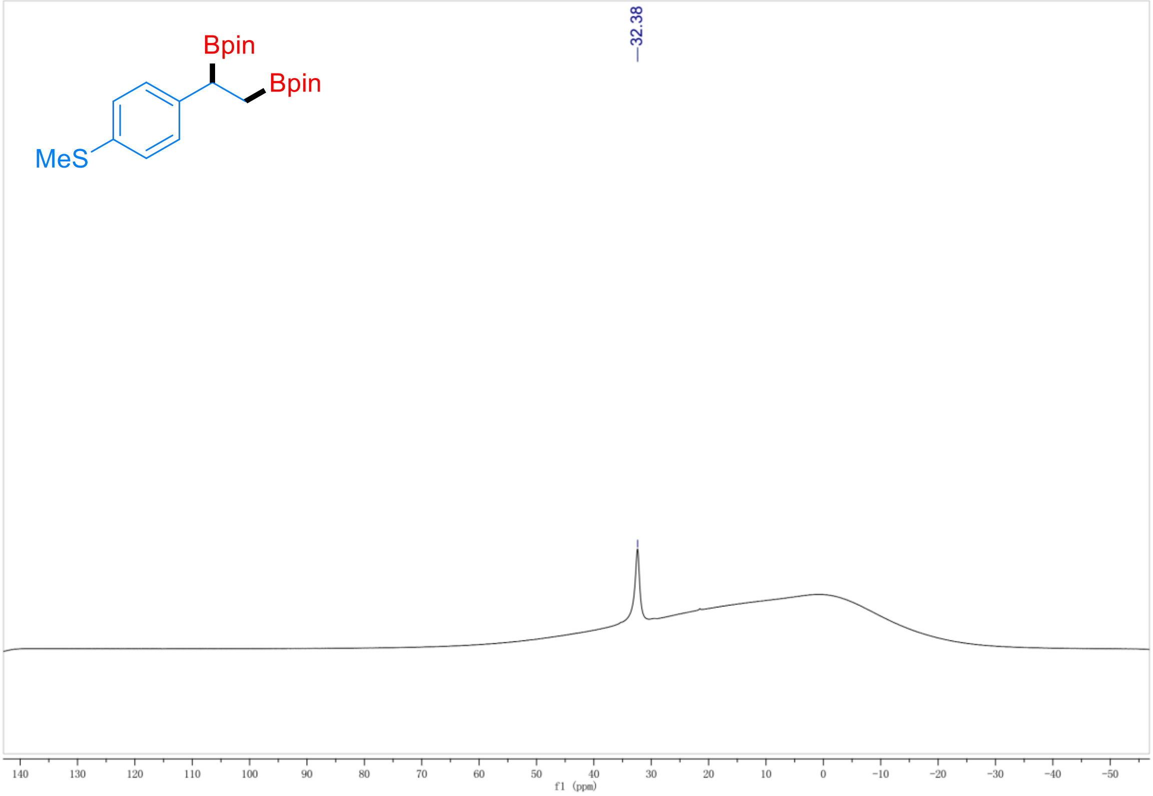

$^{11}B\{^{1}H\}$ NMR spectrum of compound **5b** in $CDCl_3$ (128 MHz).

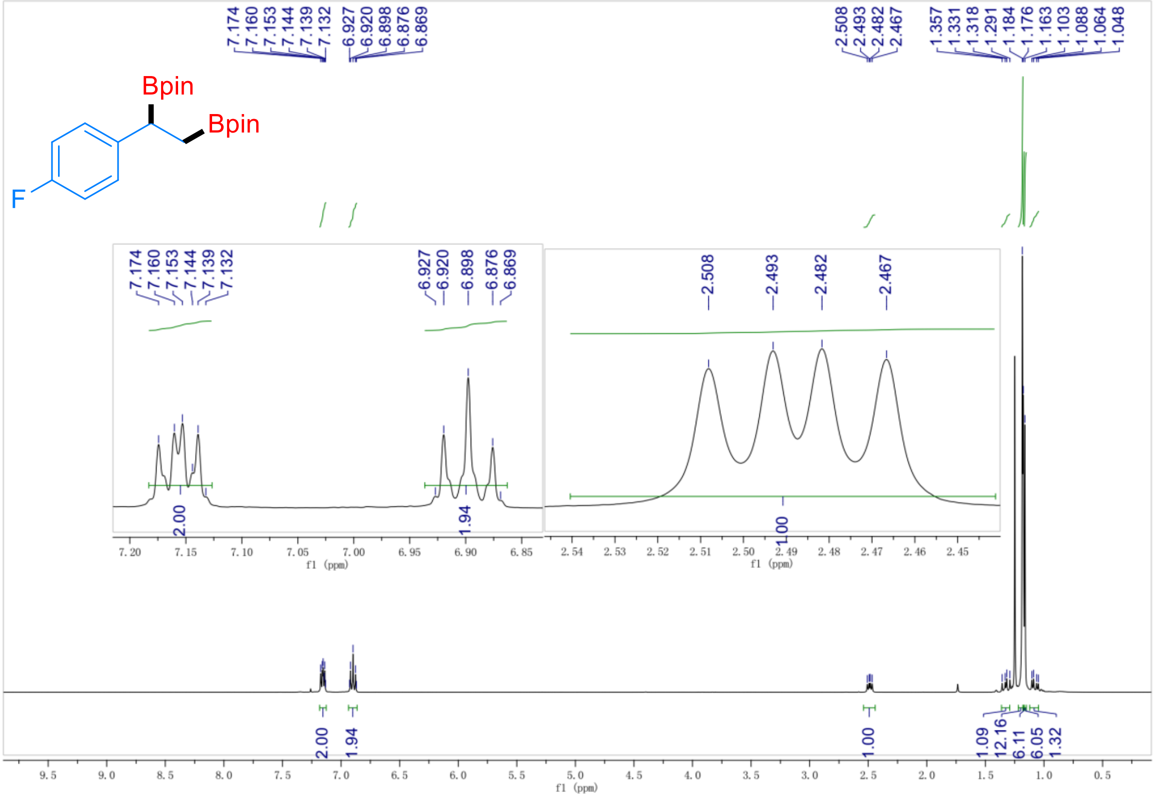

^{1}H NMR spectrum of compound **6b** in $CDCl_3$ (400 MHz).

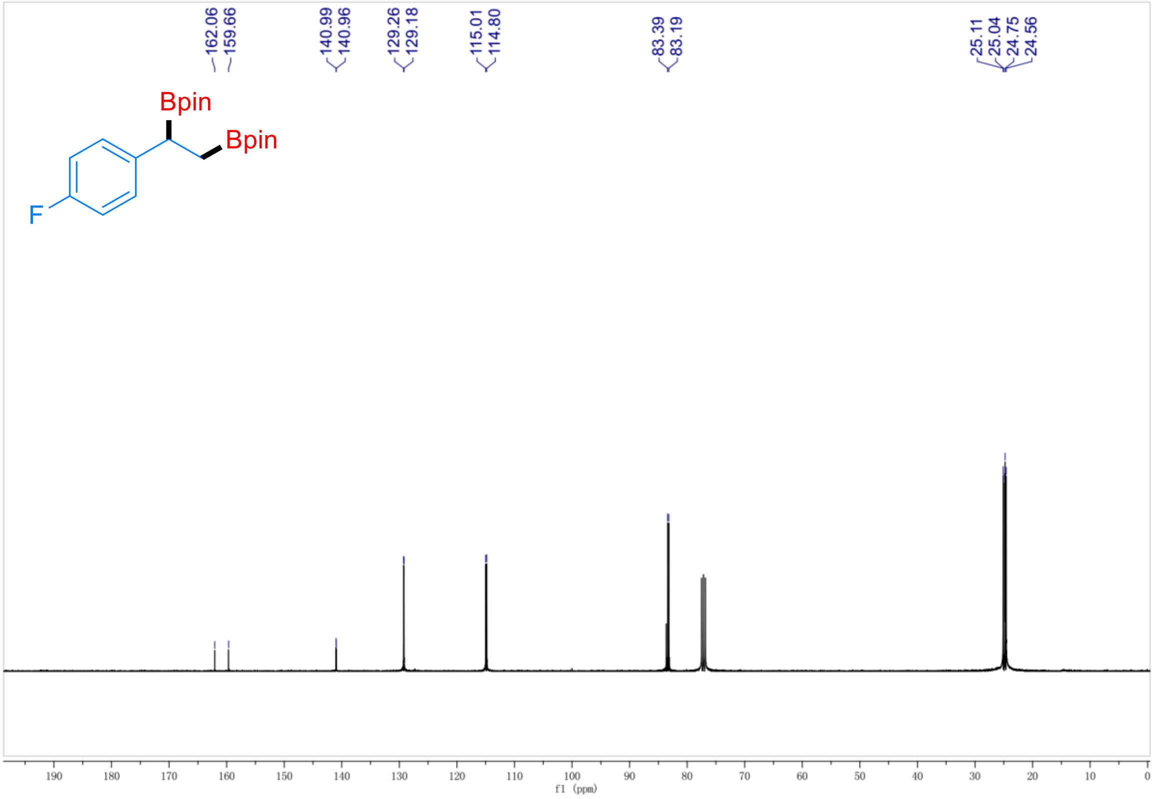

$^{13}C\{^{1}H\}$ NMR spectrum of compound **6b** in $CDCl_3$ (100 MHz).

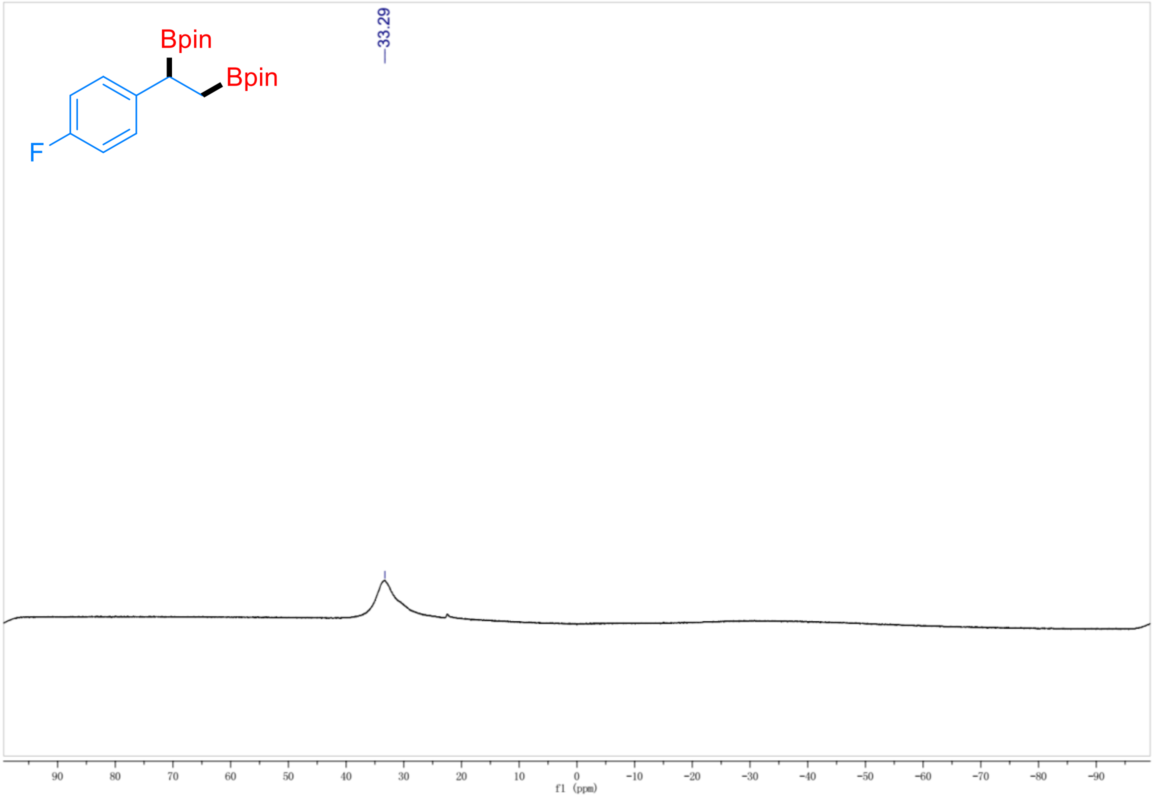

$^{11}B\{^{1}H\}$ NMR spectrum of compound **6b** in $CDCl_3$ (128 MHz).

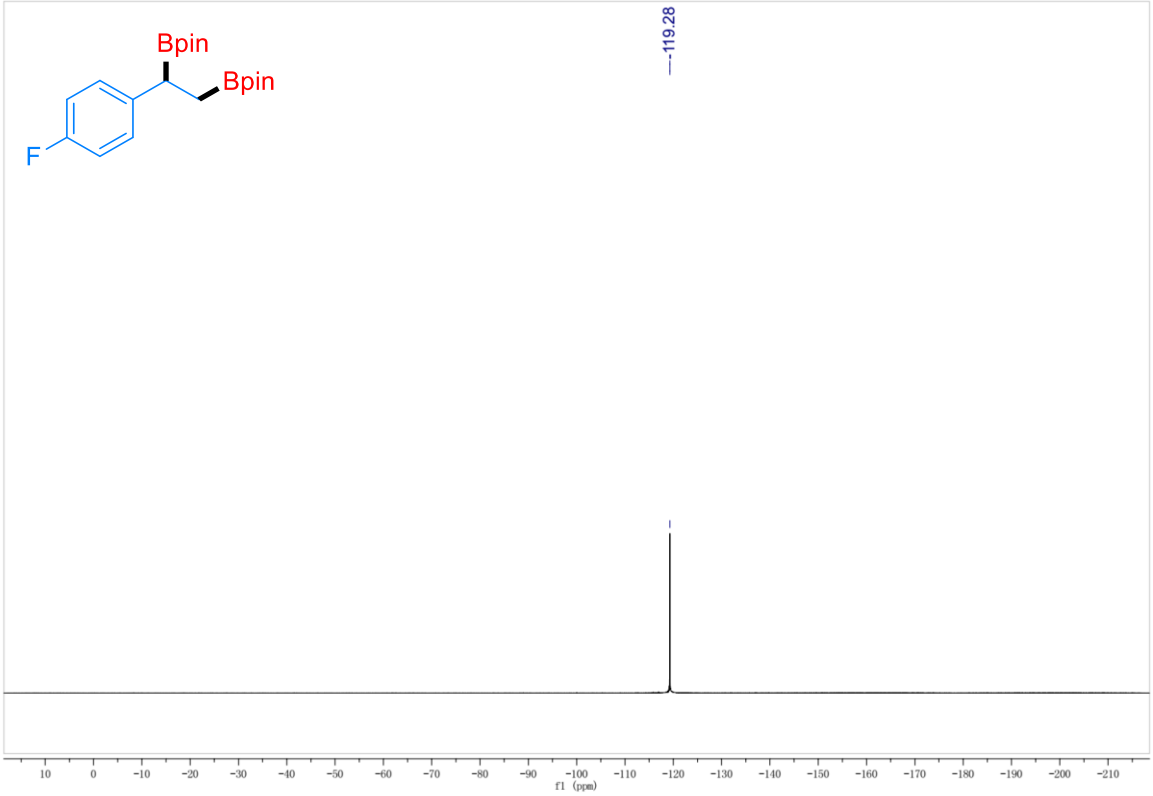

^{19}F{^{1}H} NMR spectrum of compound **6b** in $CDCl_3$ (376 MHz).

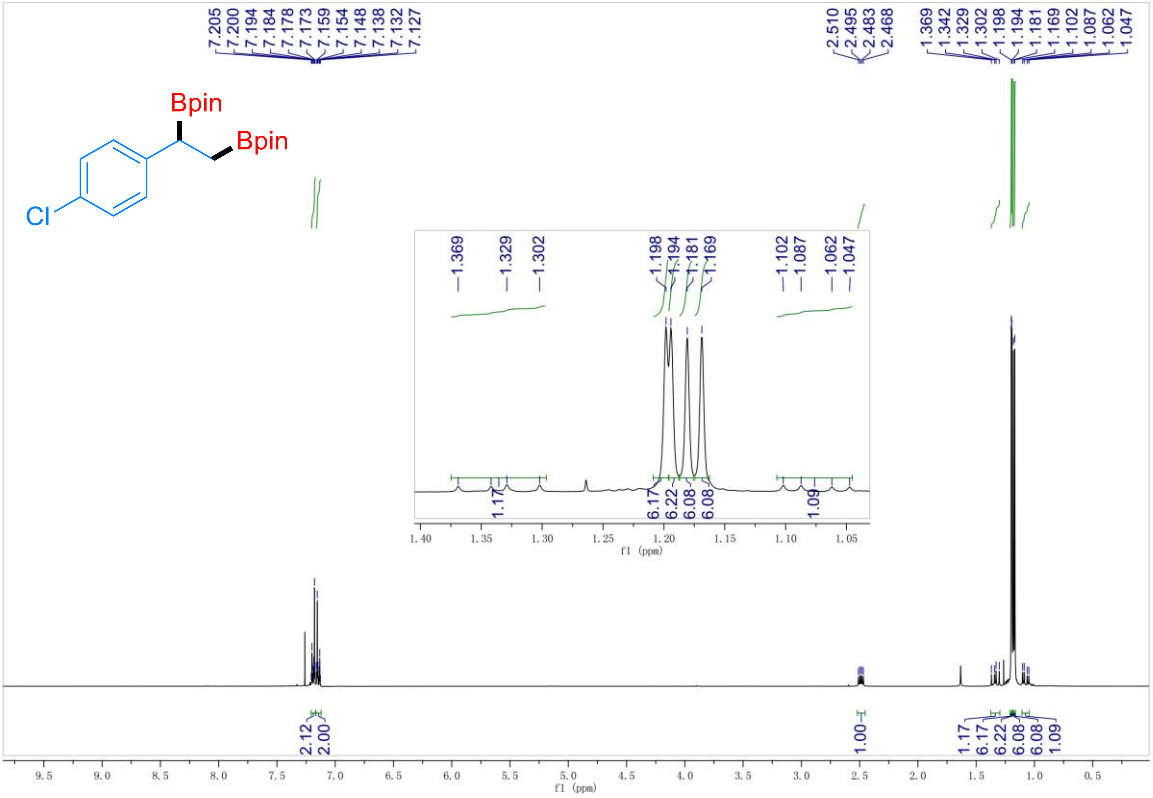

^{1}H NMR spectrum of compound **7b** in $CDCl_3$ (400 MHz).

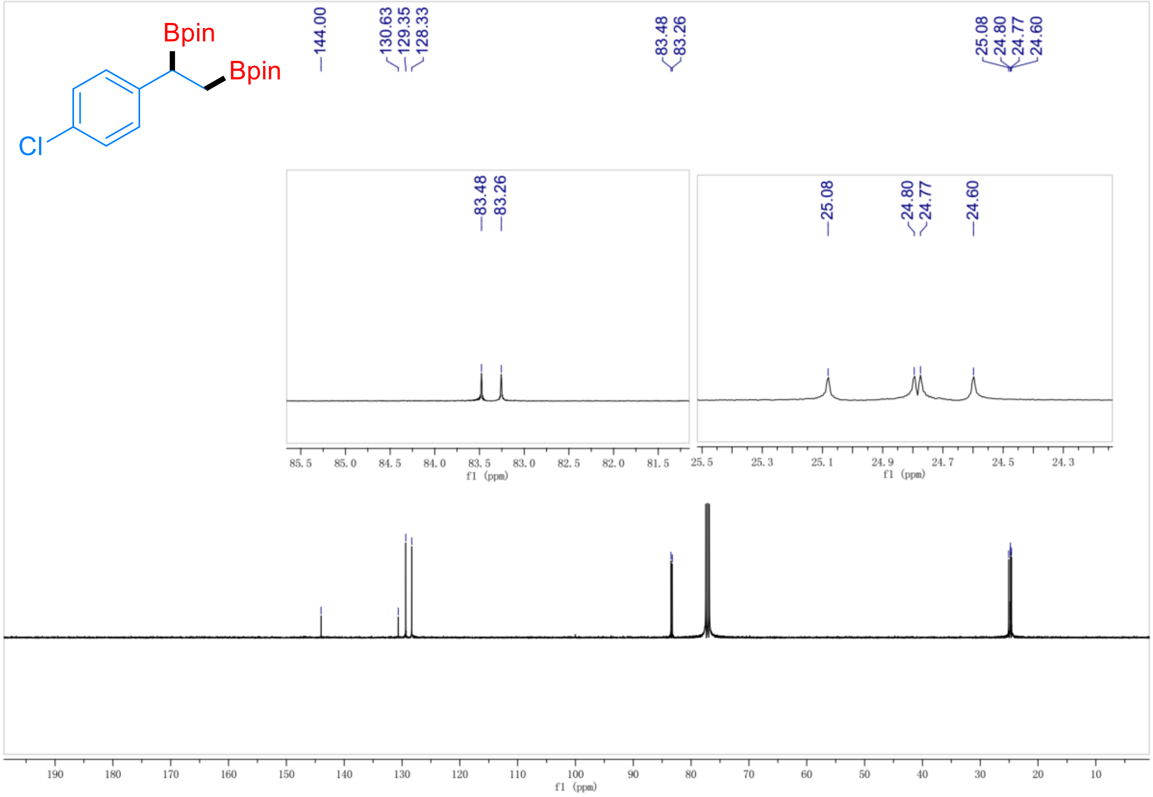

$^{13}C\{^1H\}$ NMR spectrum of compound **7b** in $CDCl_3$ (100 MHz).

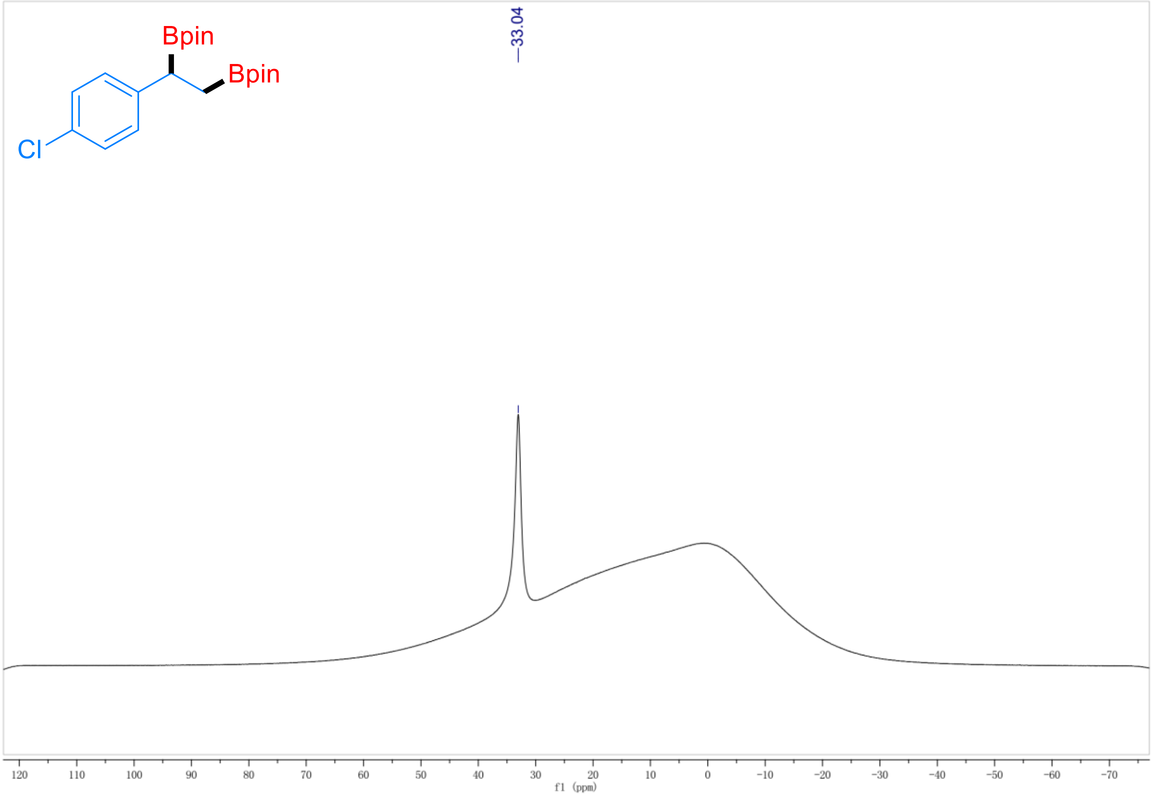

$^{11}B\{^1H\}$ NMR spectrum of compound **7b** in $CDCl_3$ (128 MHz).

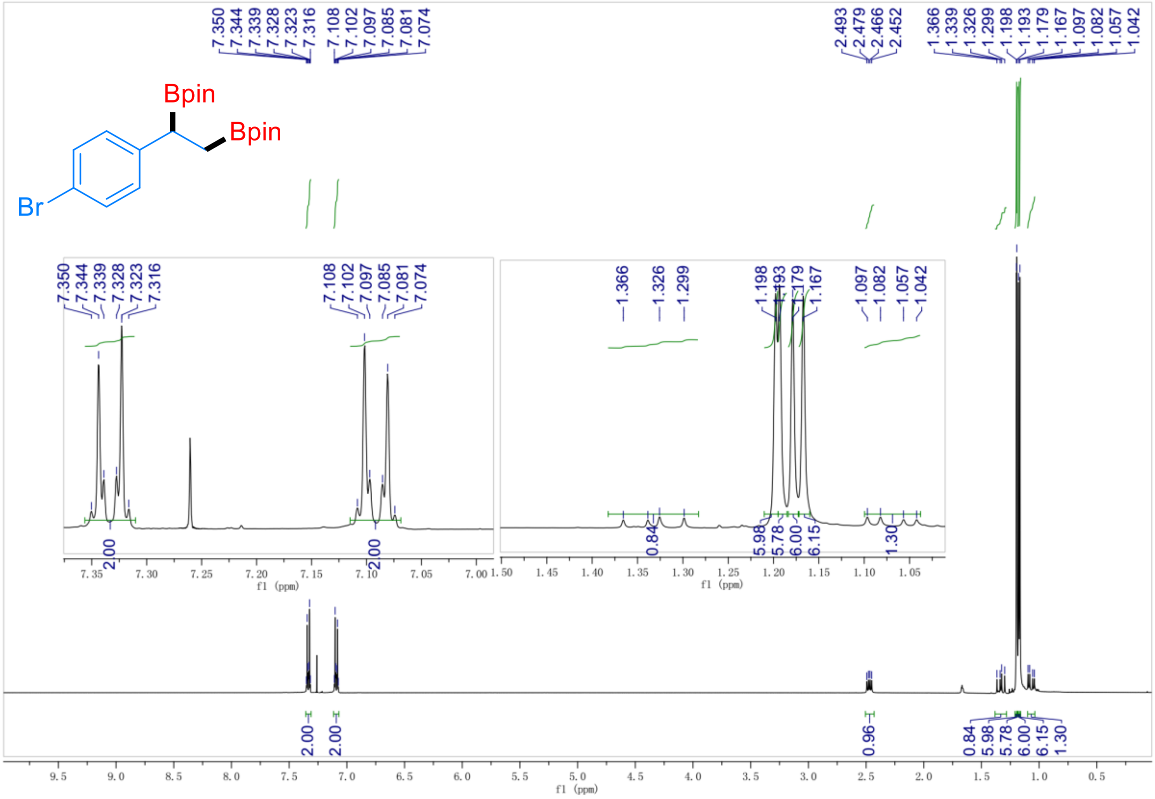

^{1}H NMR spectrum of compound **8b** in $CDCl_3$ (400 MHz).

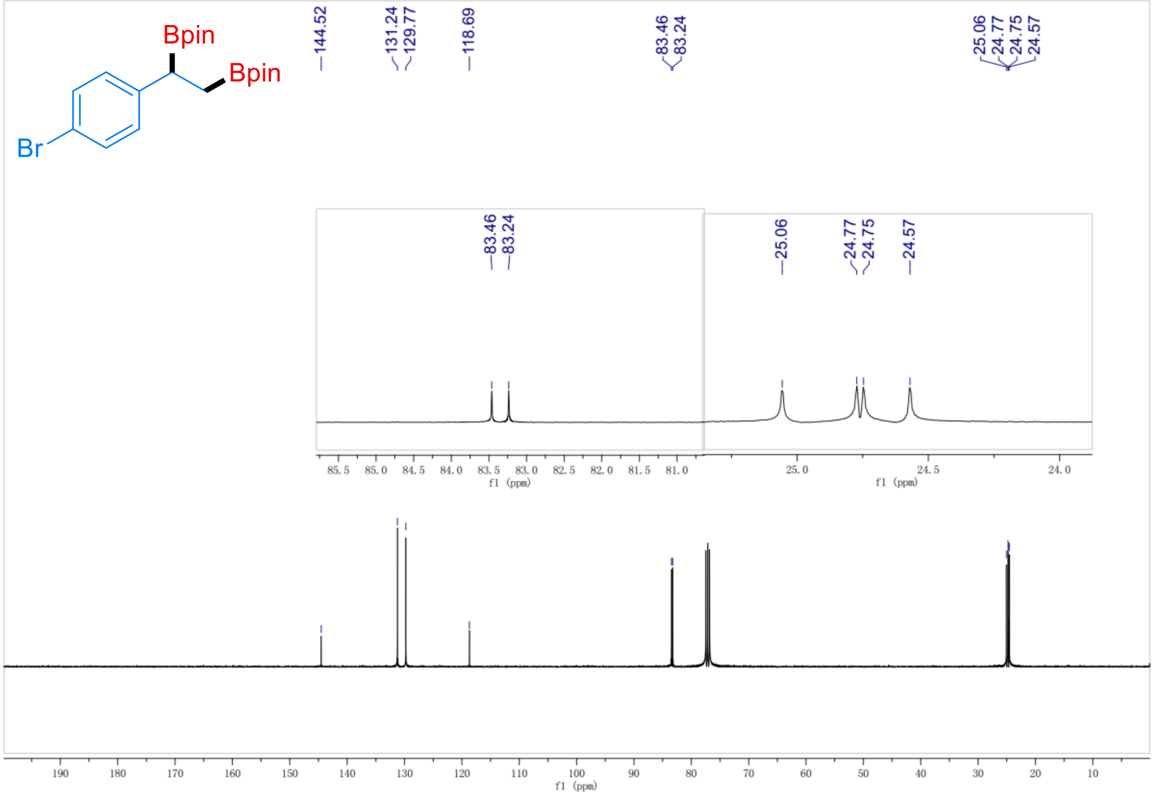

^{13}C{^{1}H} NMR spectrum of compound **8b** in $CDCl_3$ (100 MHz).

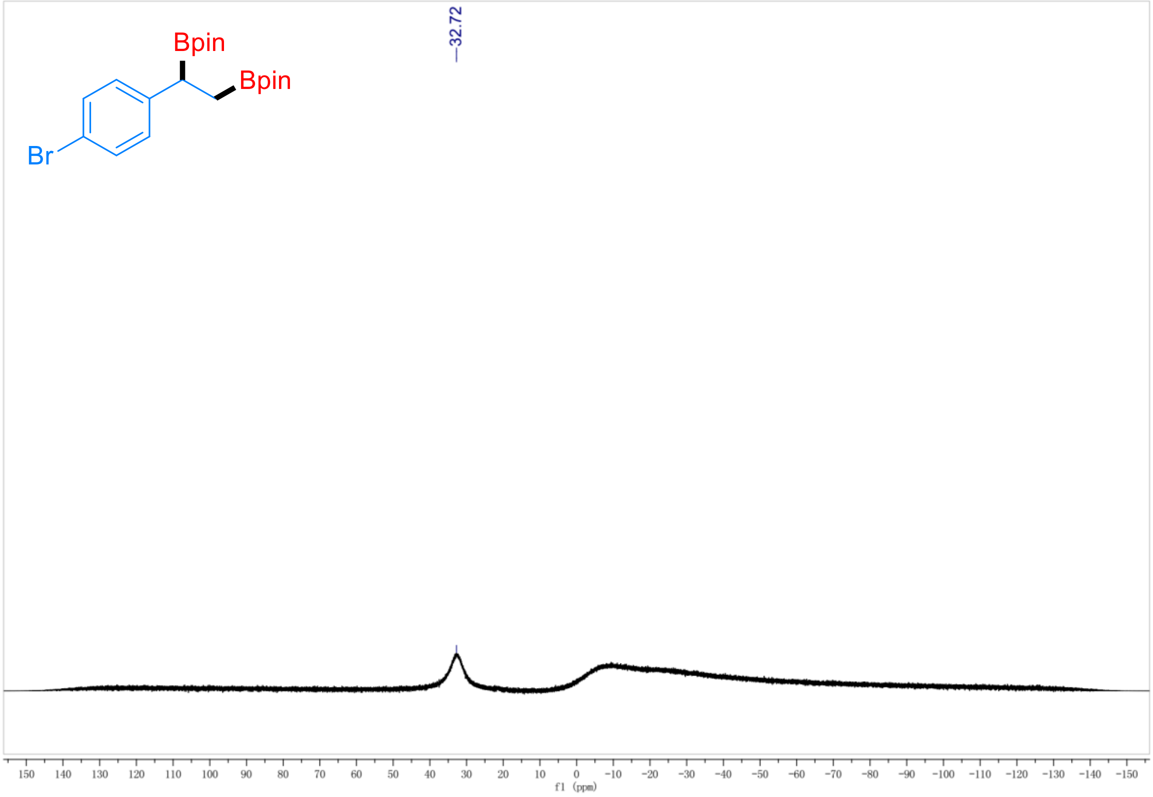

^{11}B{^{1}H} NMR spectrum of compound **8b** in $CDCl_3$ (128 MHz).

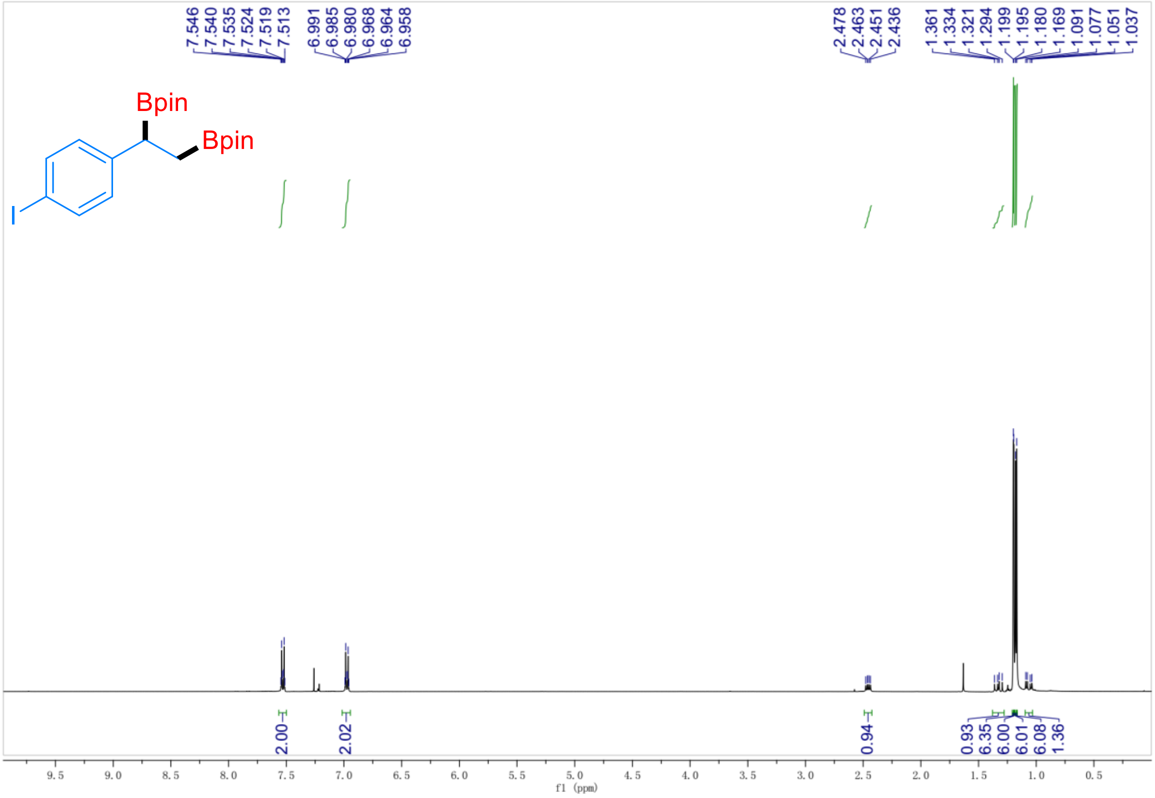

^{1}H NMR spectrum of compound **9b** in $CDCl_3$ (400 MHz).

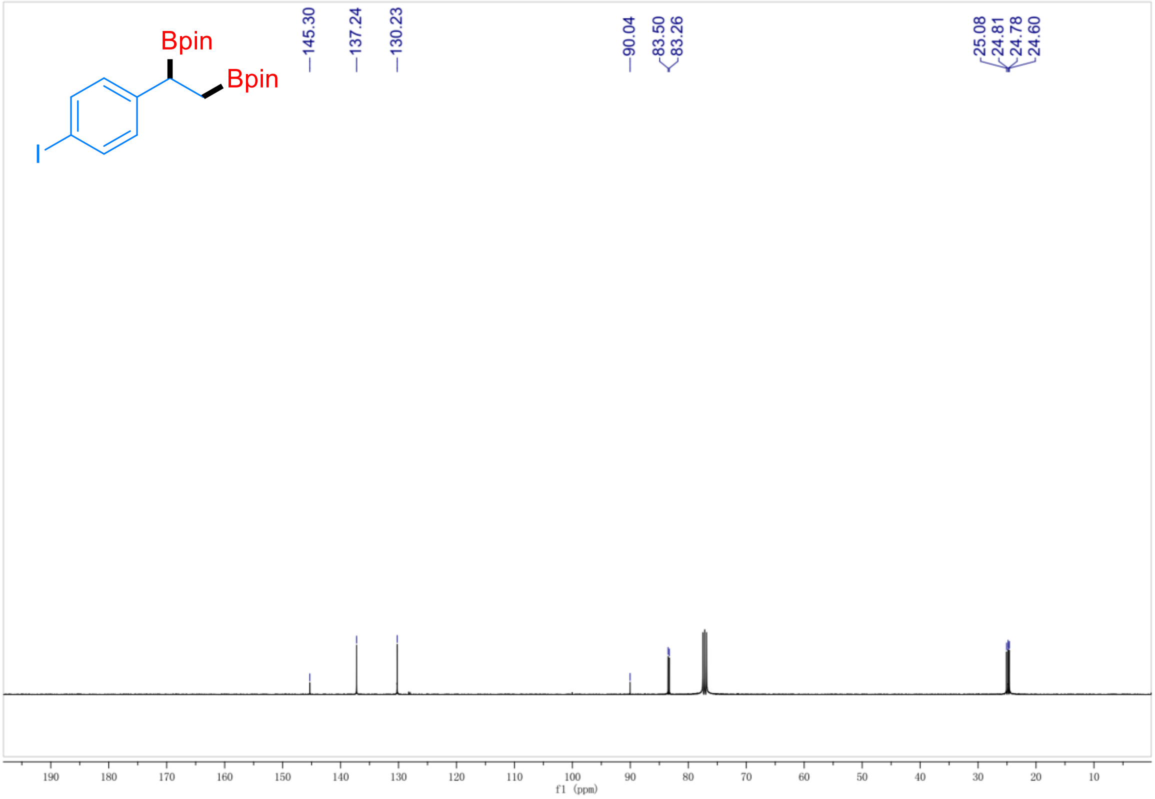

$^{13}C\{^{1}H\}$ NMR spectrum of compound **9b** in $CDCl_3$ (100 MHz).

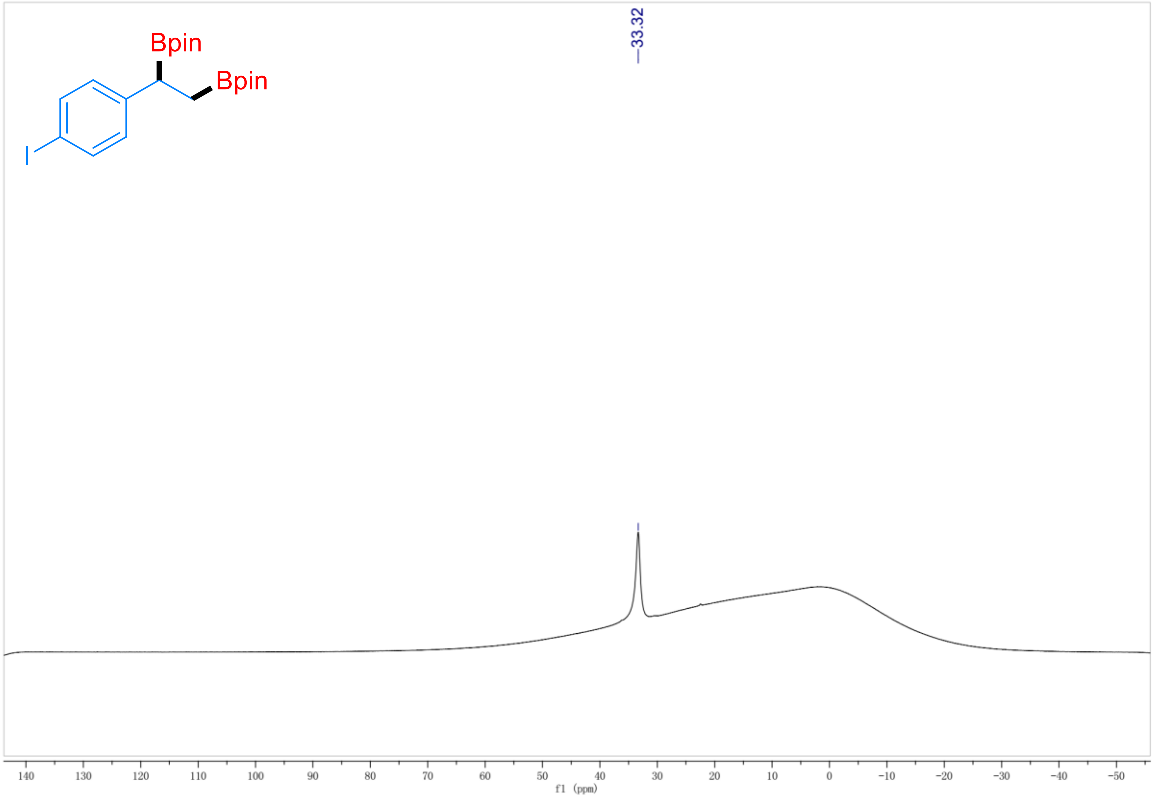

$^{11}B\{^{1}H\}$ NMR spectrum of compound **9b** in $CDCl_3$ (128 MHz).

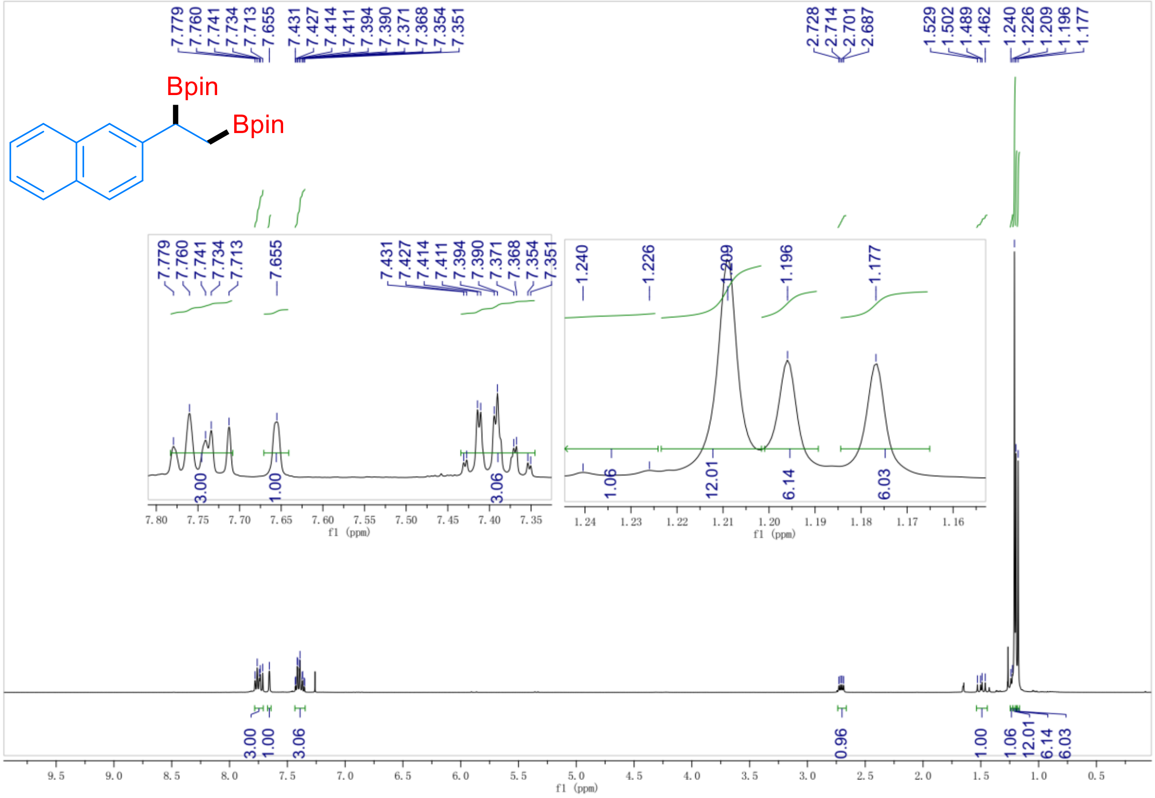

^{1}H NMR spectrum of compound **10b** in $CDCl_3$ (400 MHz).

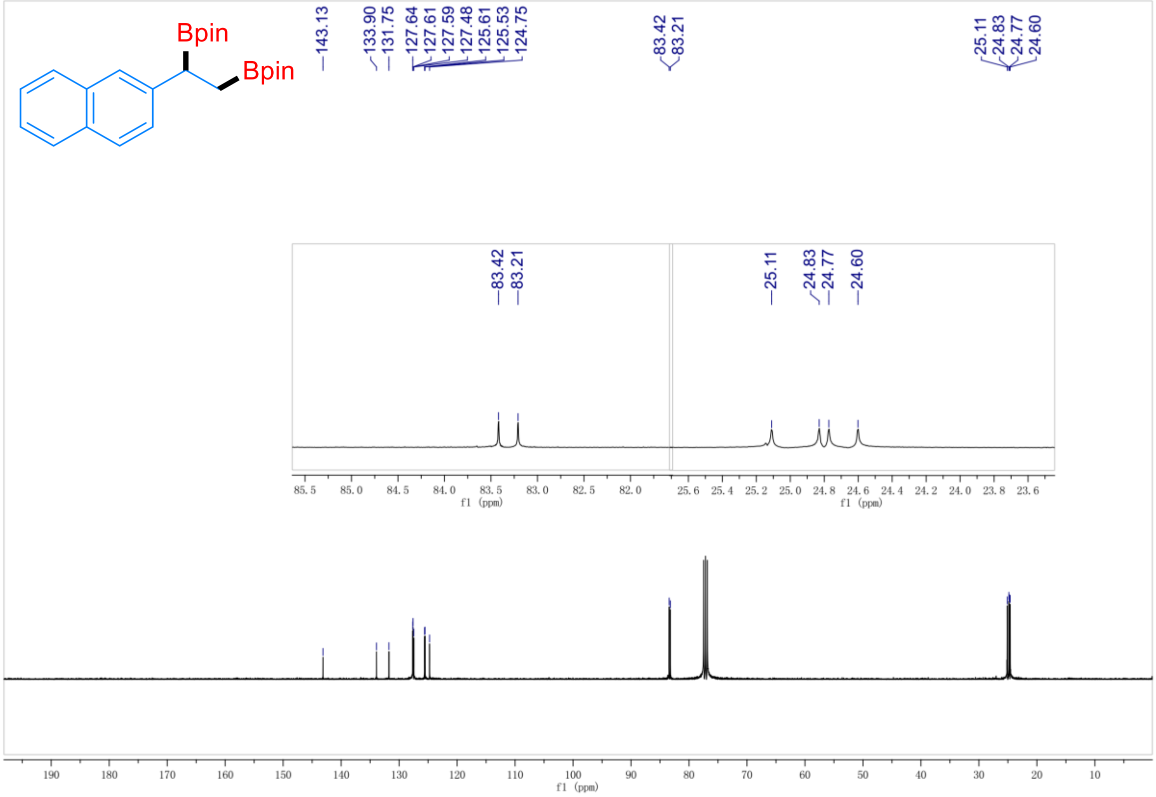

^{13}C{^{1}H} NMR spectrum of compound **10a** in $CDCl_3$ (100 MHz).

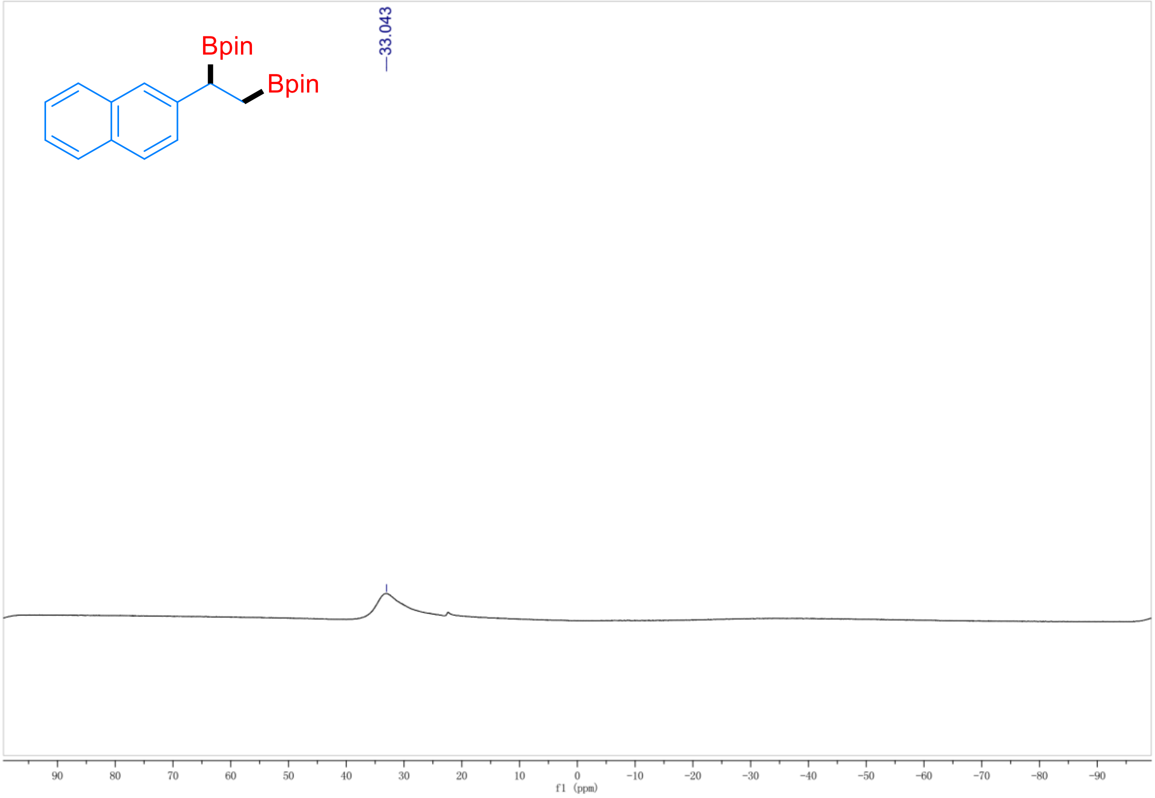

$^{11}B\{^{1}H\}$ NMR spectrum of compound **10b** in $CDCl_3$ (128 MHz).

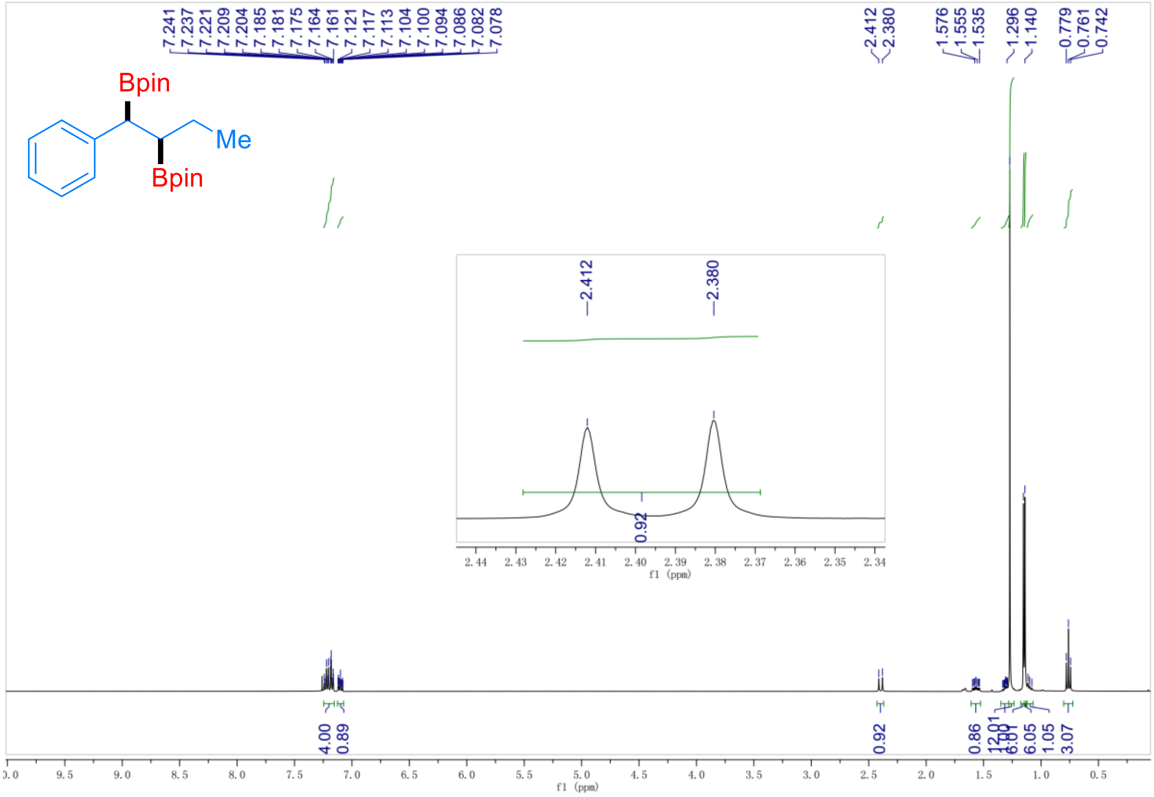

^{1}H NMR spectrum of compound **11b** in $CDCl_3$ (400 MHz).

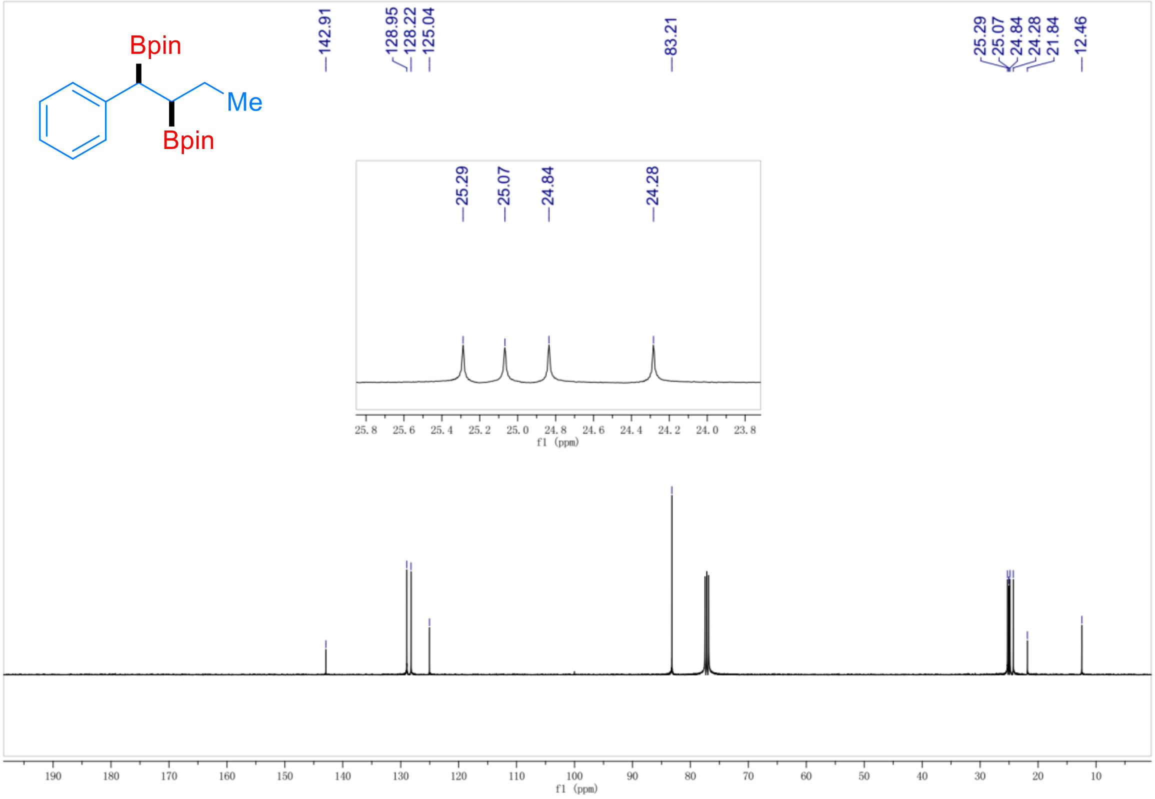

$^{13}C\{^1H\}$ NMR spectrum of compound **11a** in $CDCl_3$ (100 MHz).

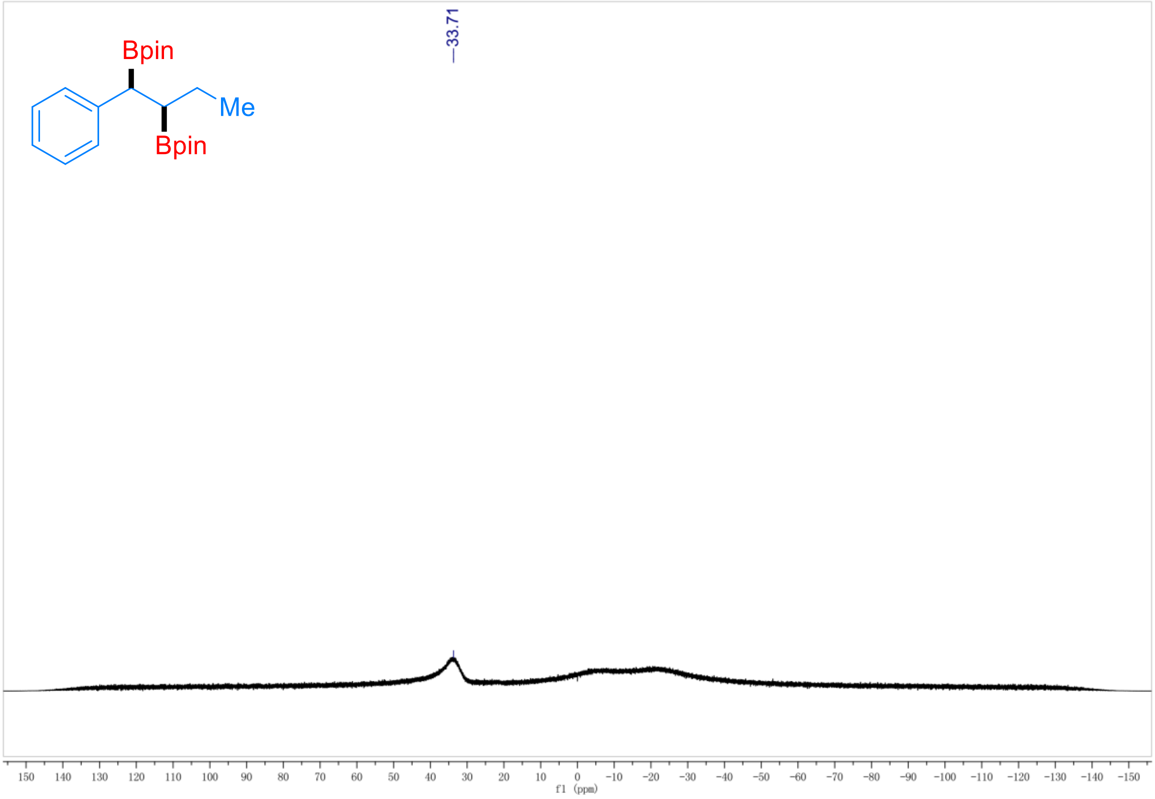

$^{11}B\{^1H\}$ NMR spectrum of compound **11b** in $CDCl_3$ (128 MHz).

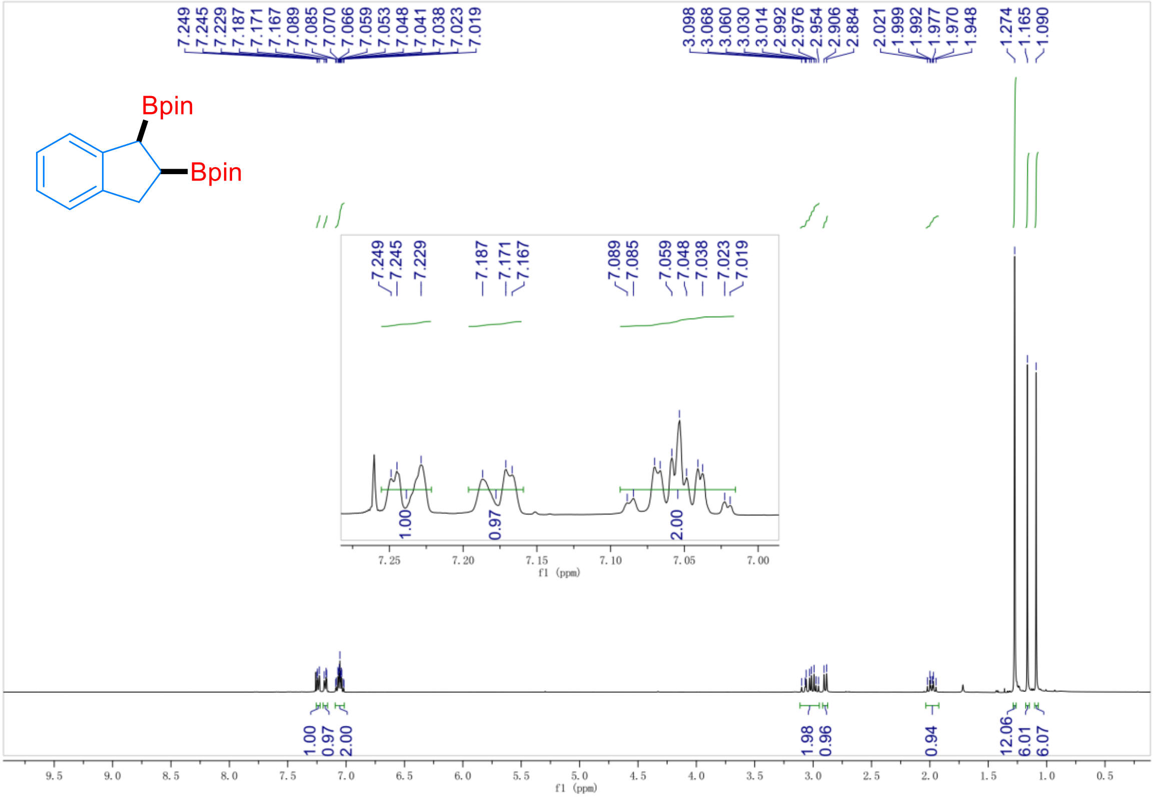

^{1}H NMR spectrum of compound **12b** in $CDCl_3$ (400 MHz).

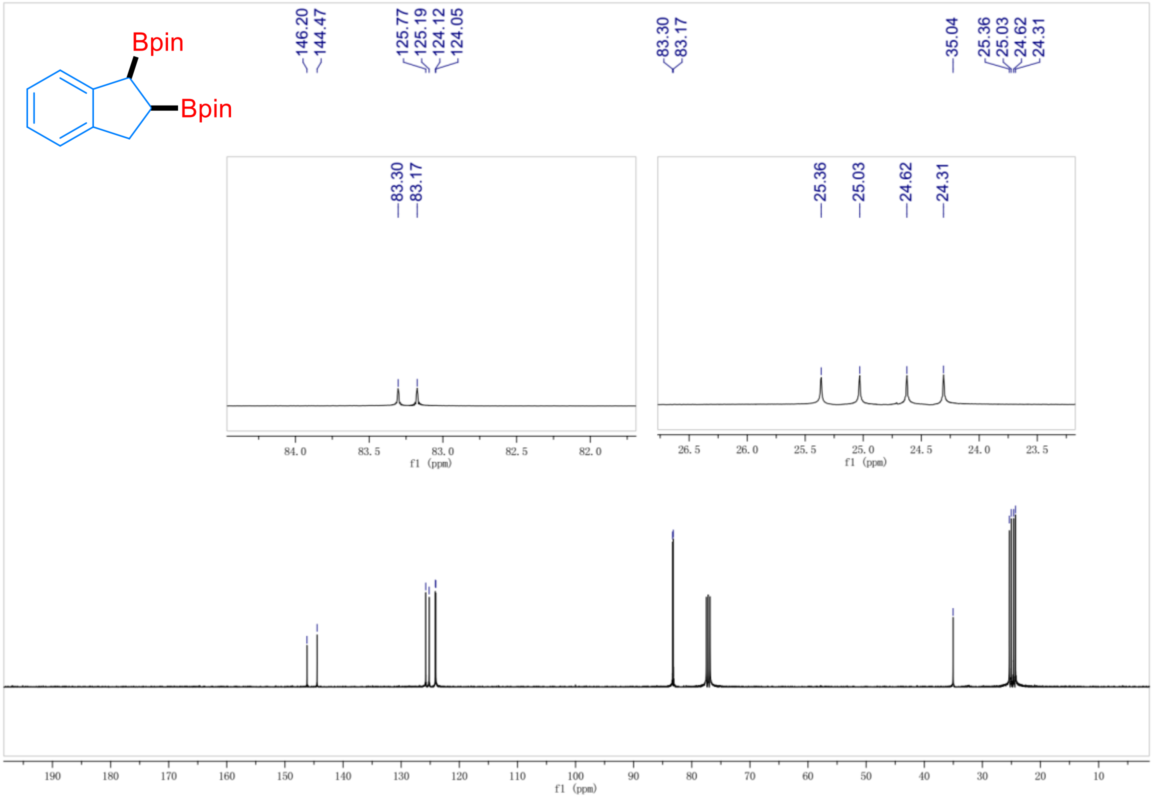

^{13}C{^{1}H} NMR spectrum of compound **12b** in $CDCl_3$ (100 MHz).

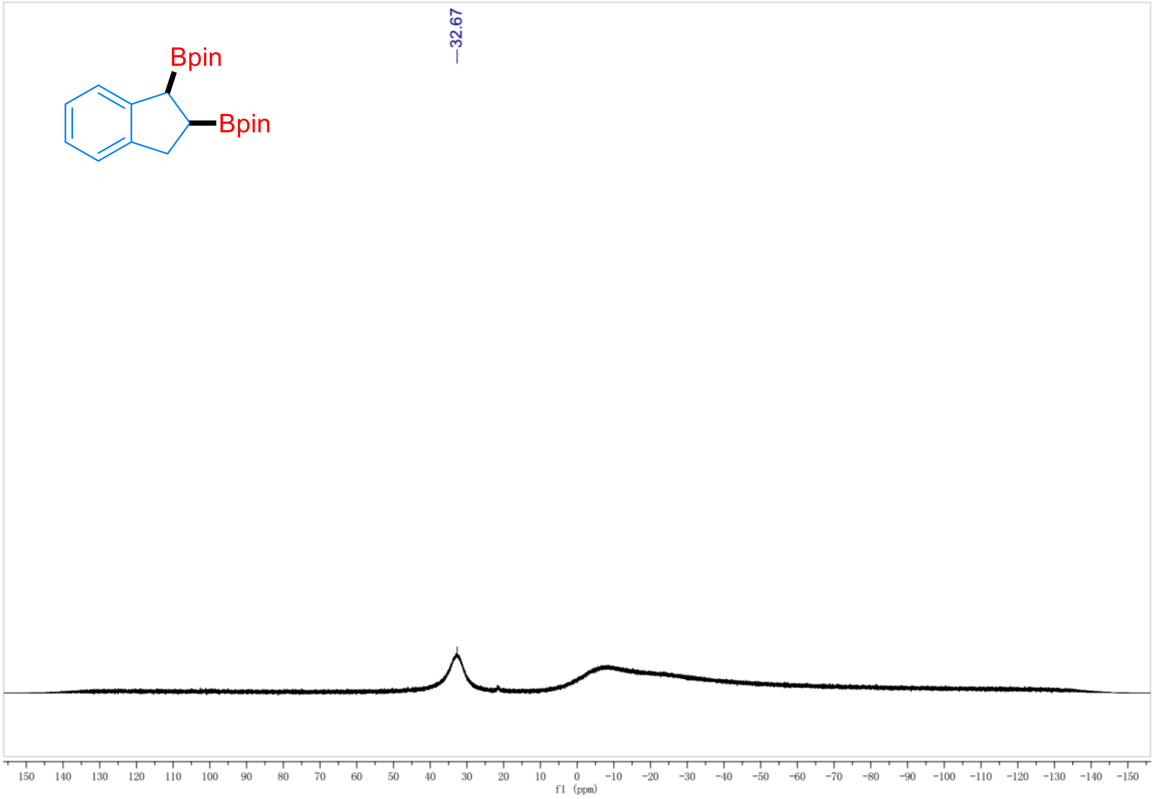

$^{11}B\{^1H\}$ NMR spectrum of compound **12b** in $CDCl_3$ (128 MHz).

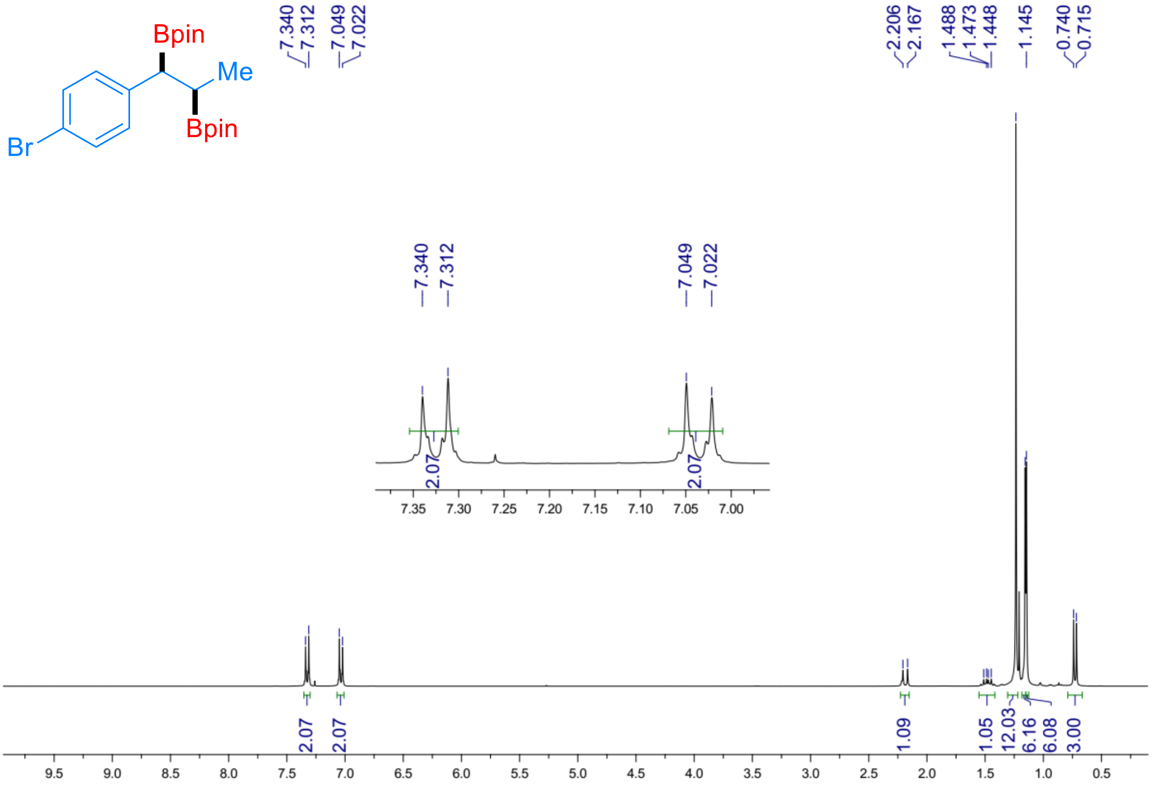

^1H NMR spectrum of compound **13b** in $CDCl_3$ (300 MHz).

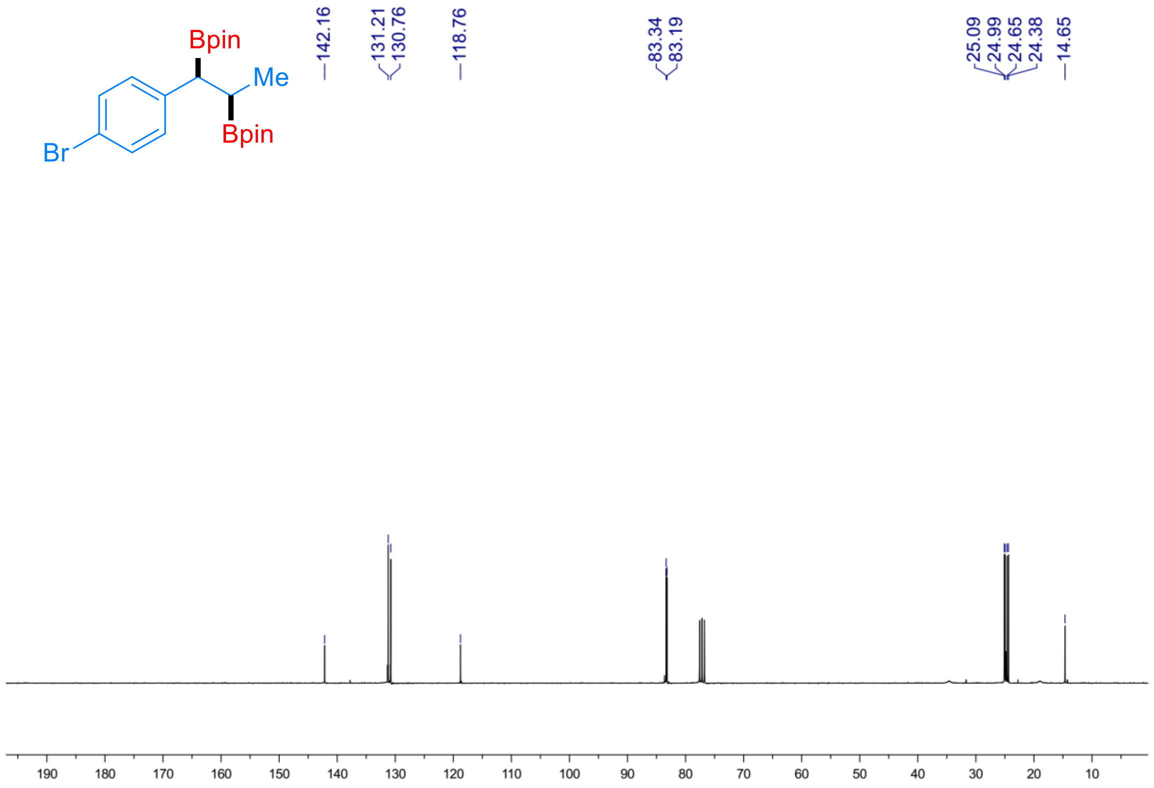

$^{13}C\{^{1}H\}$ NMR spectrum of compound **13b** in $CDCl_3$ (75 MHz).

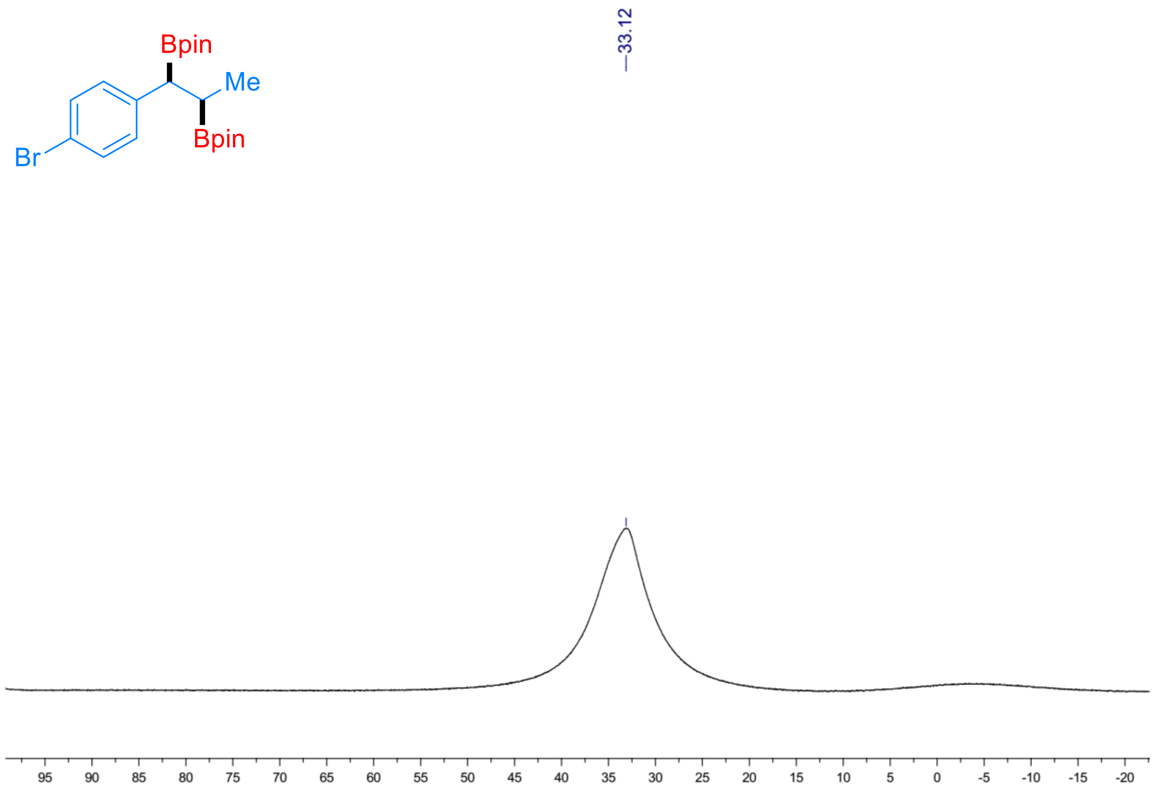

$^{11}B\{^{1}H\}$ NMR spectrum of compound **13b** in $CDCl_3$ (96 MHz).

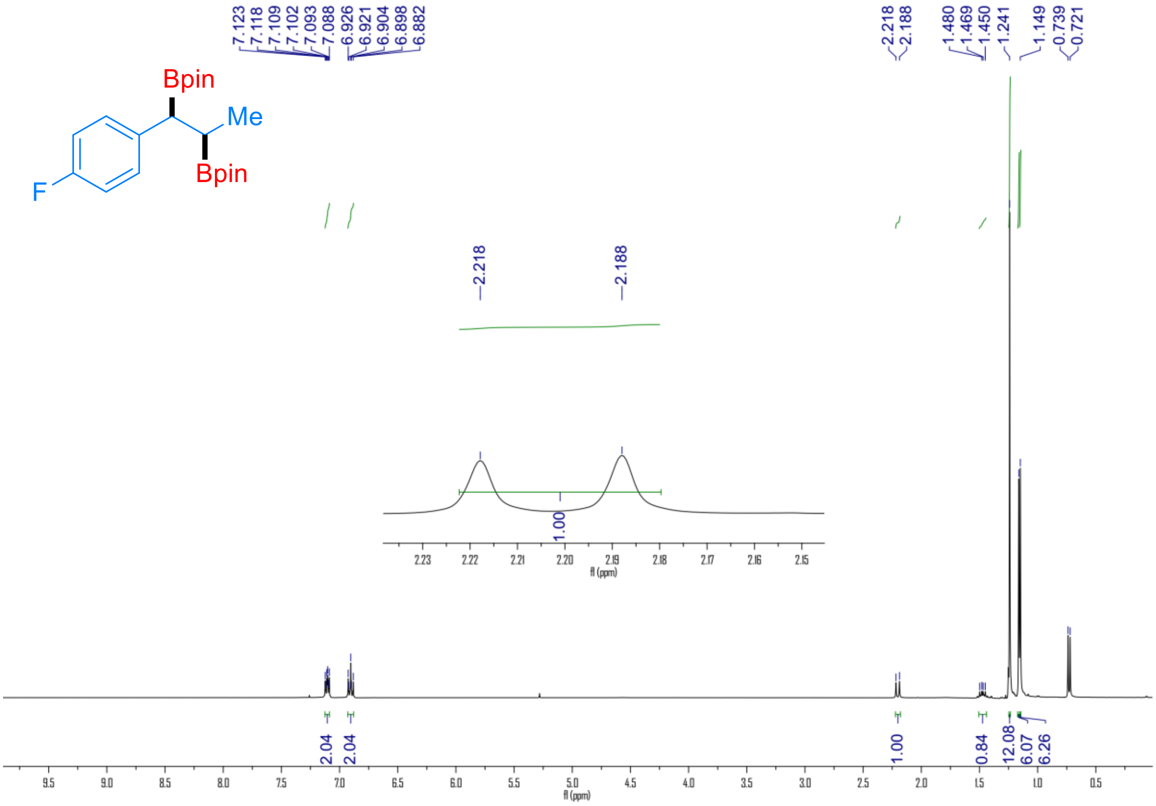

^{1}H NMR spectrum of compound **14b** in $CDCl_3$ (400 MHz).

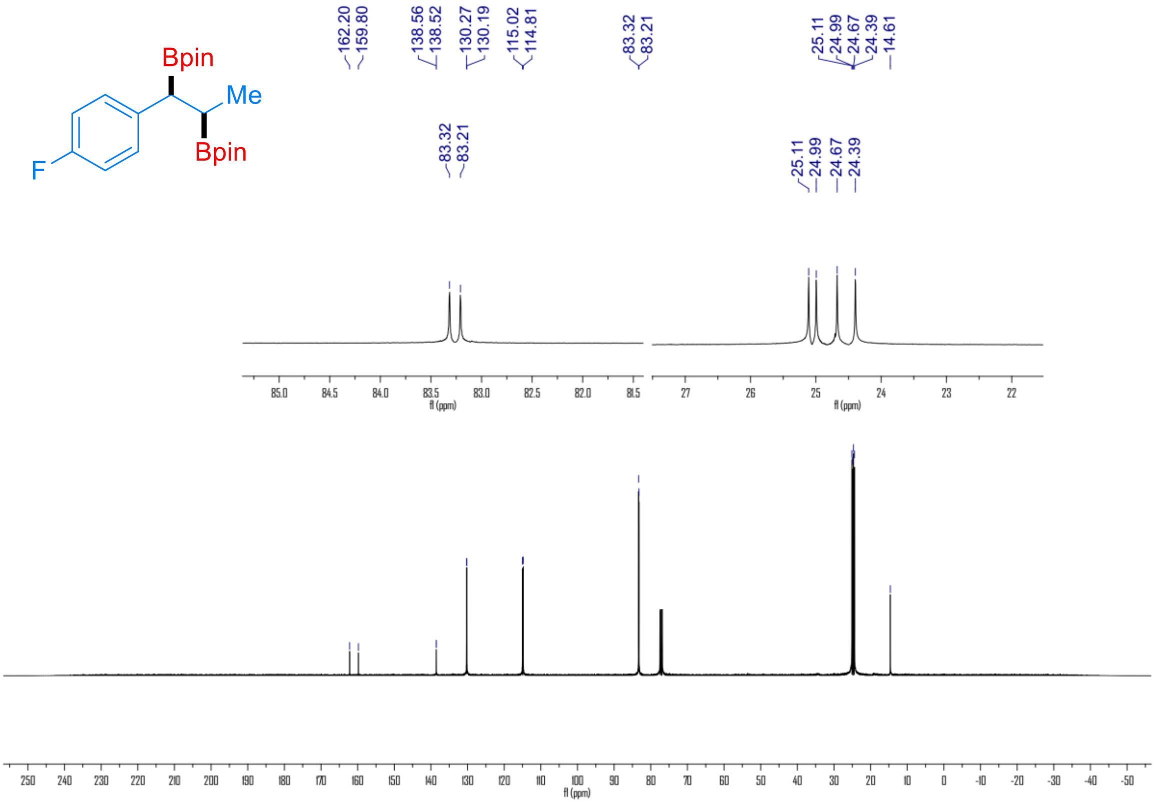

^{13}C{^{1}H} NMR spectrum of compound **14b** in $CDCl_3$ (100 MHz).

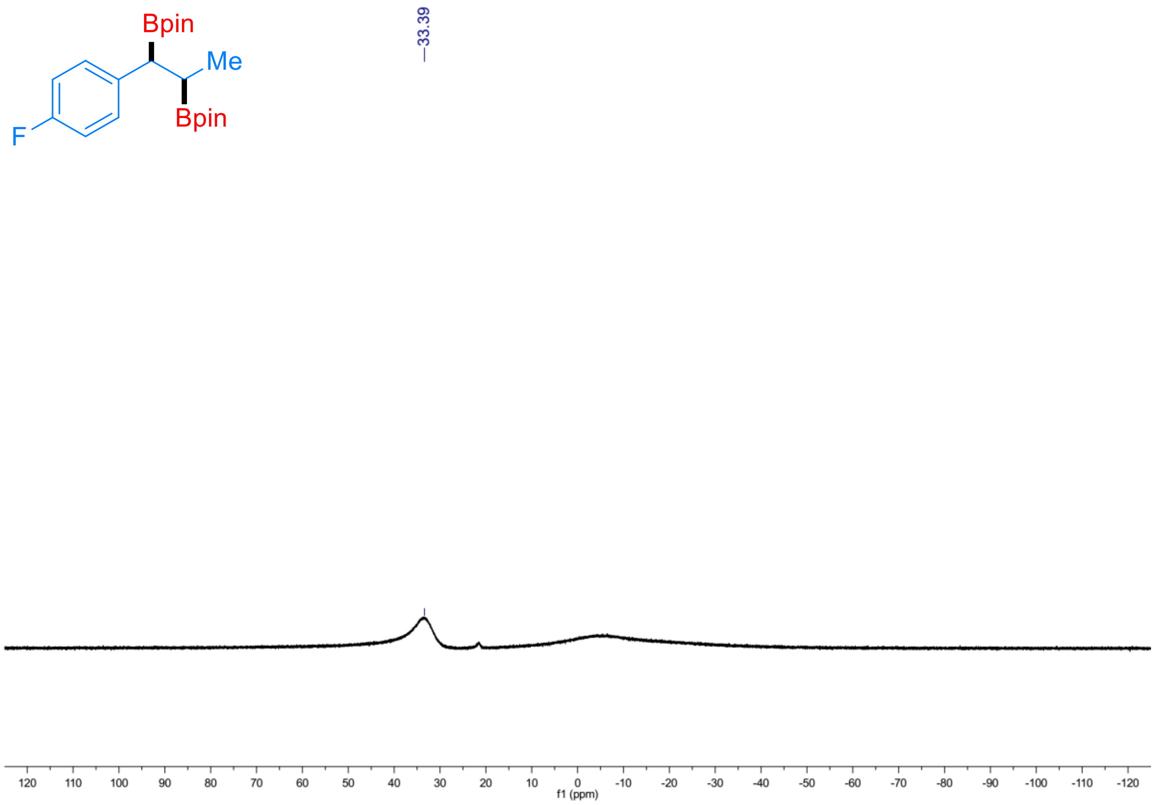

$^{11}B\{^{1}H\}$ NMR spectrum of compound **14b** in $CDCl_3$ (128 MHz).

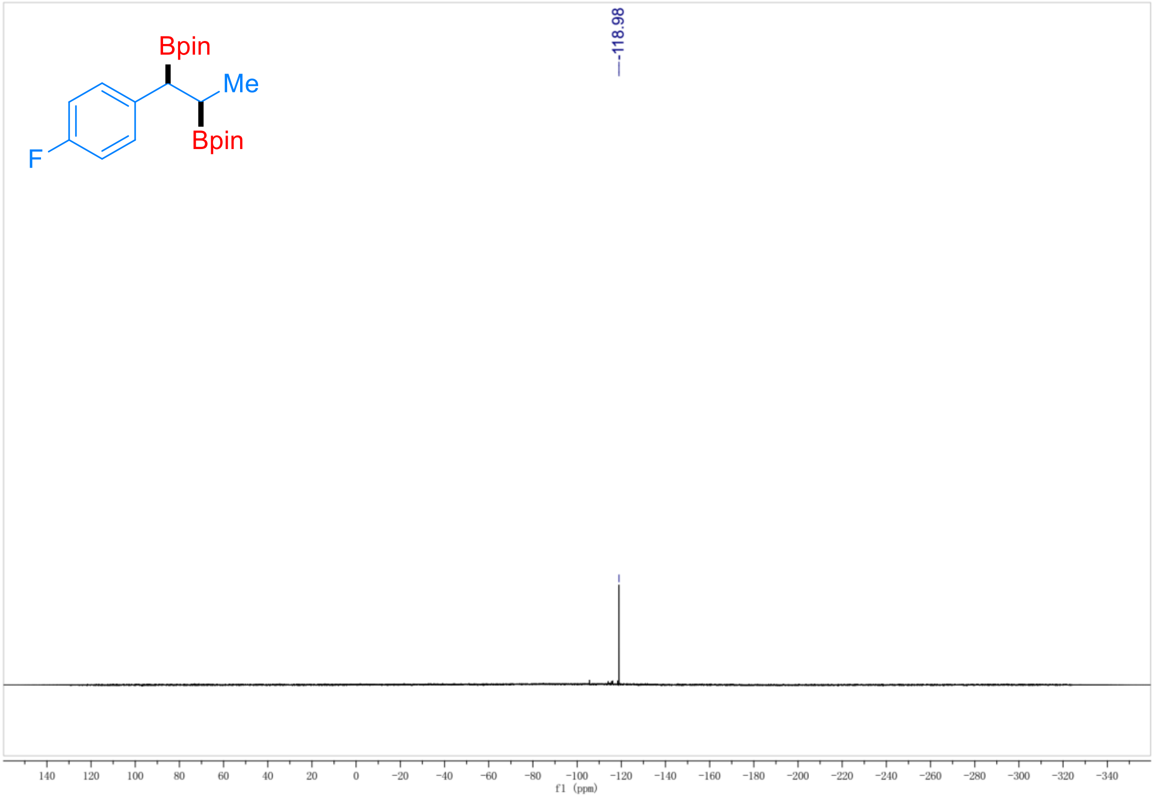

$^{19}F\{^{1}H\}$ NMR spectrum of compound **14b** in $CDCl_3$ (376 MHz).

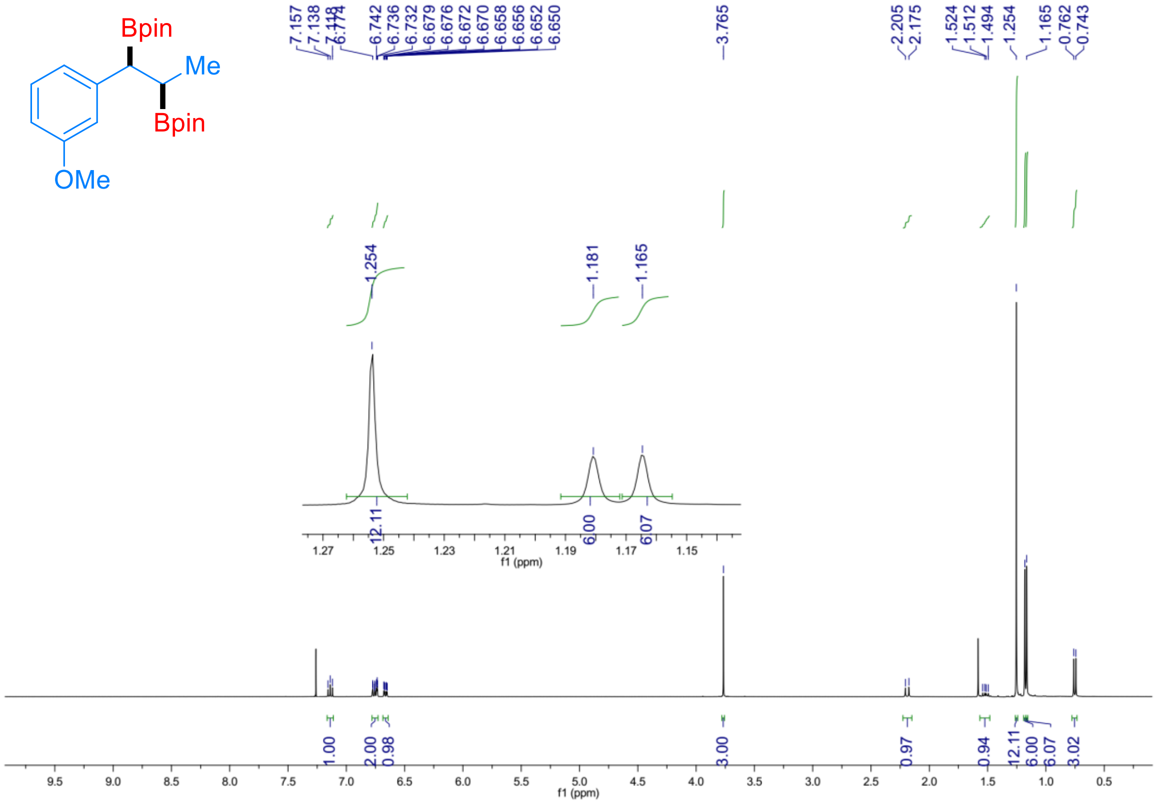

^{1}H NMR spectrum of compound **15b** in $CDCl_3$ (400 MHz).

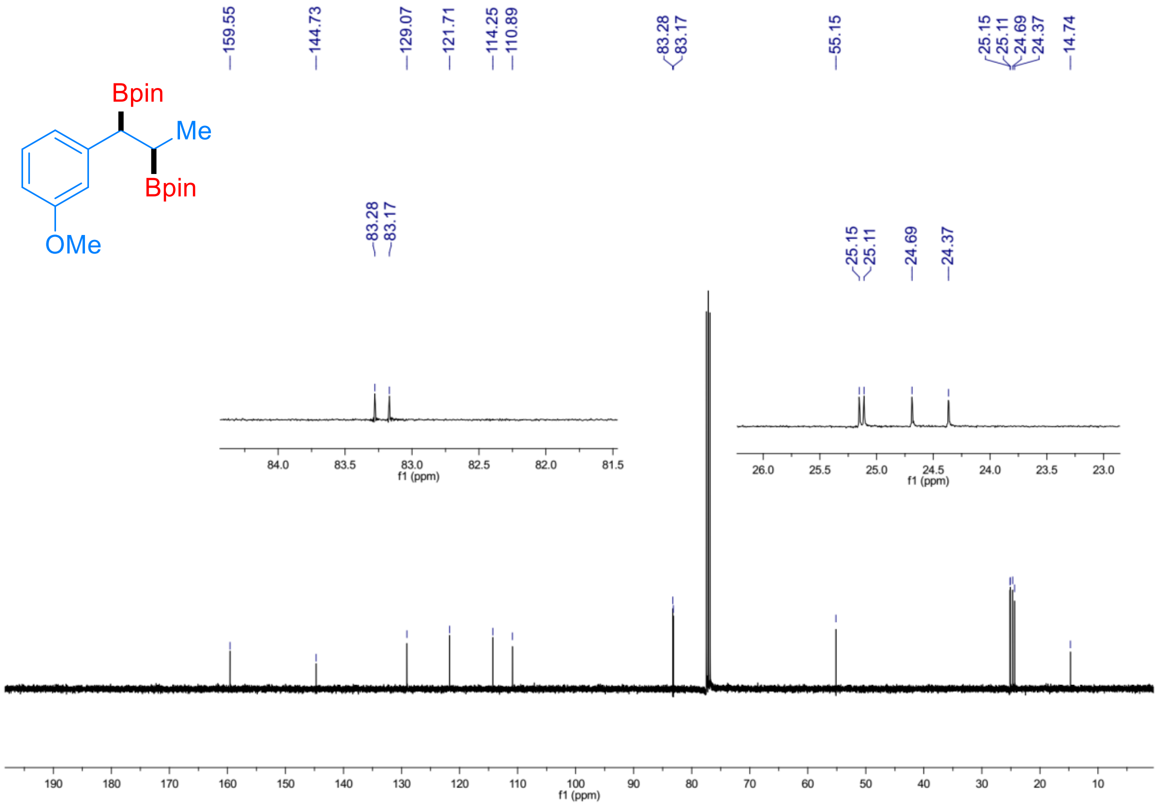

^{13}C{^{1}H} NMR spectrum of compound **15b** in $CDCl_3$ (100 MHz).

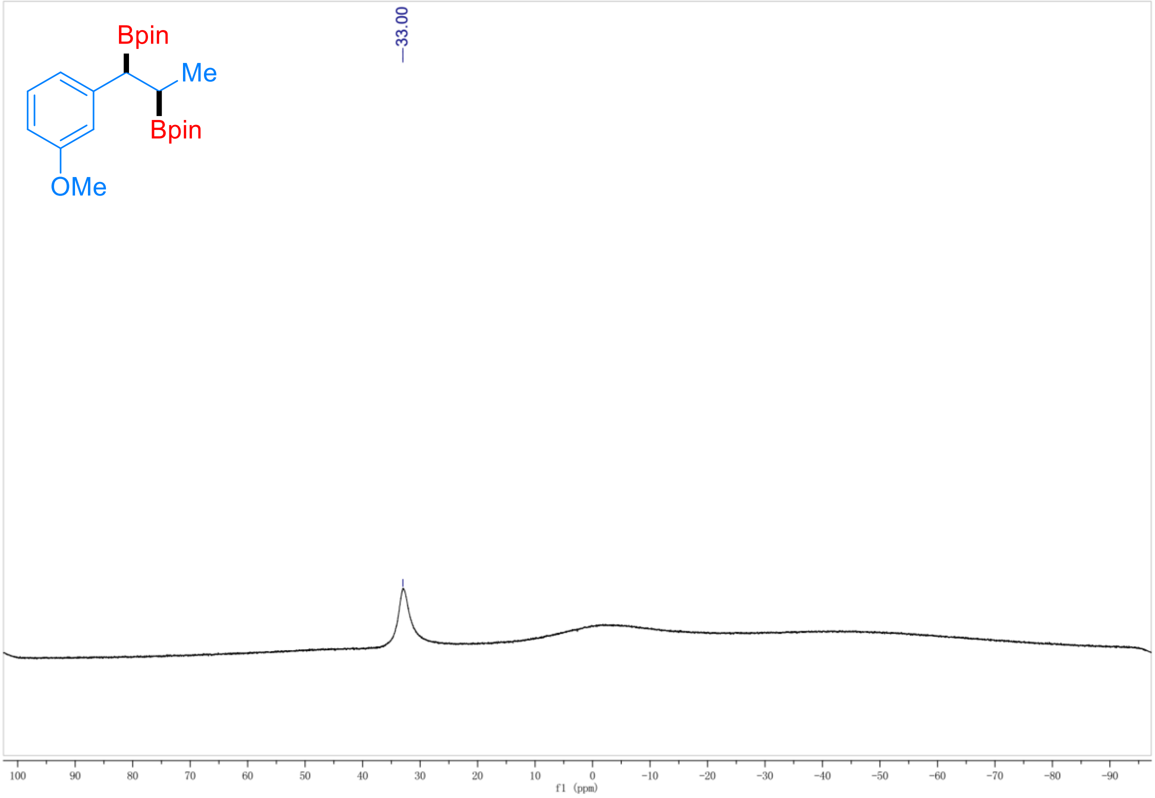

^{11}B{^{1}H} NMR spectrum of compound **15b** in $CDCl_3$ (128 MHz).

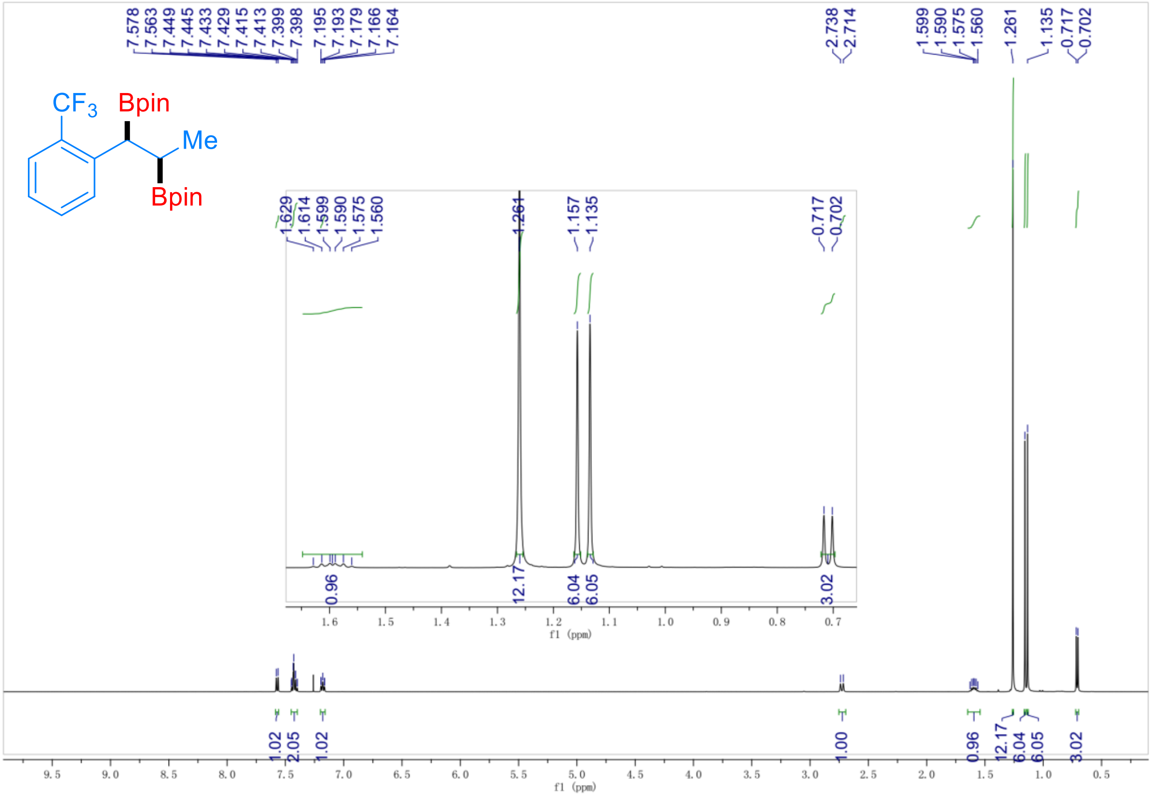

^{1}H NMR spectrum of compound **16b** in $CDCl_3$ (500 MHz).

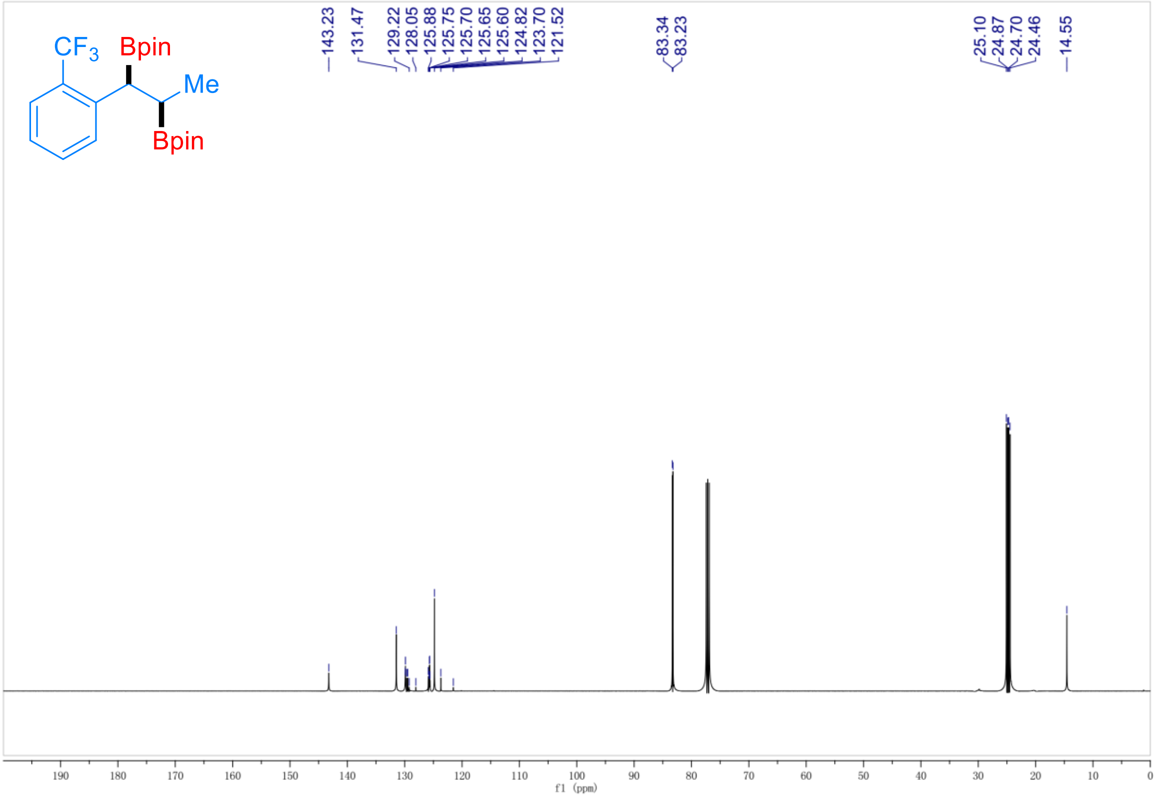

$^{13}C\{^{1}H\}$ NMR spectrum of compound **16b** in $CDCl_3$ (125 MHz).

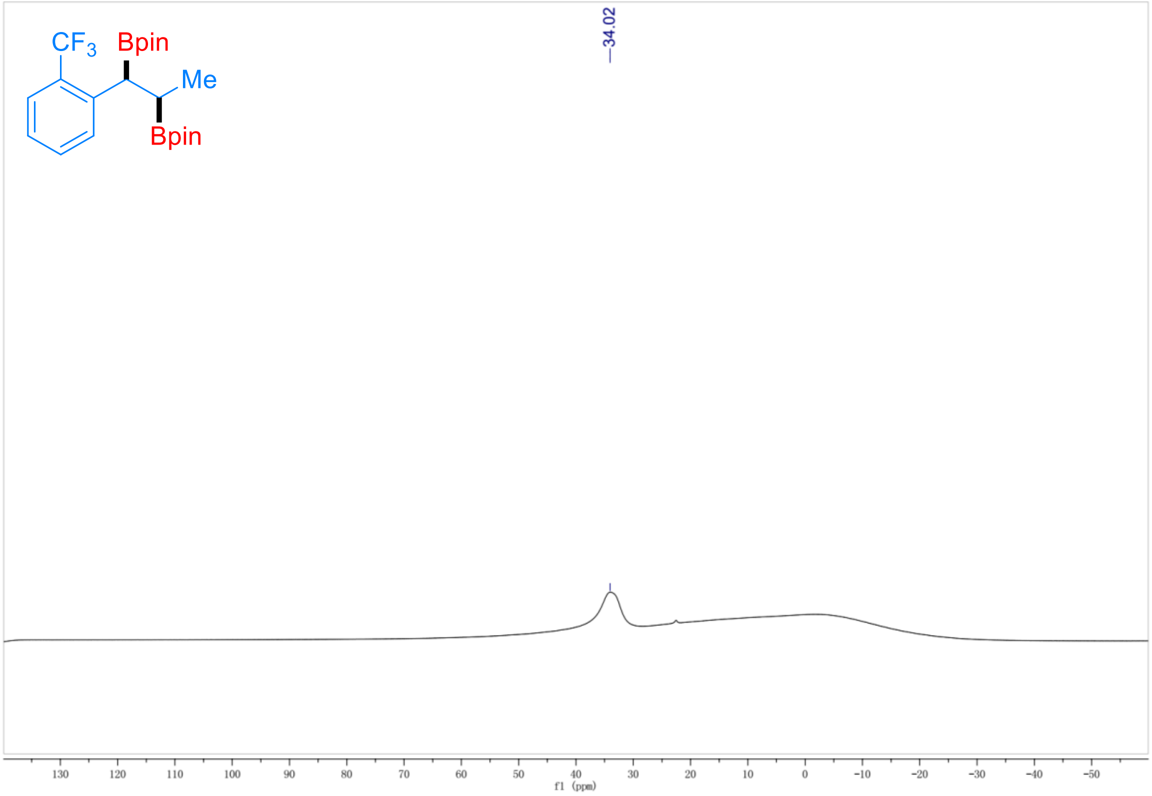

$^{11}B\{^{1}H\}$ NMR spectrum of compound **16b** in $CDCl_3$ (160 MHz).

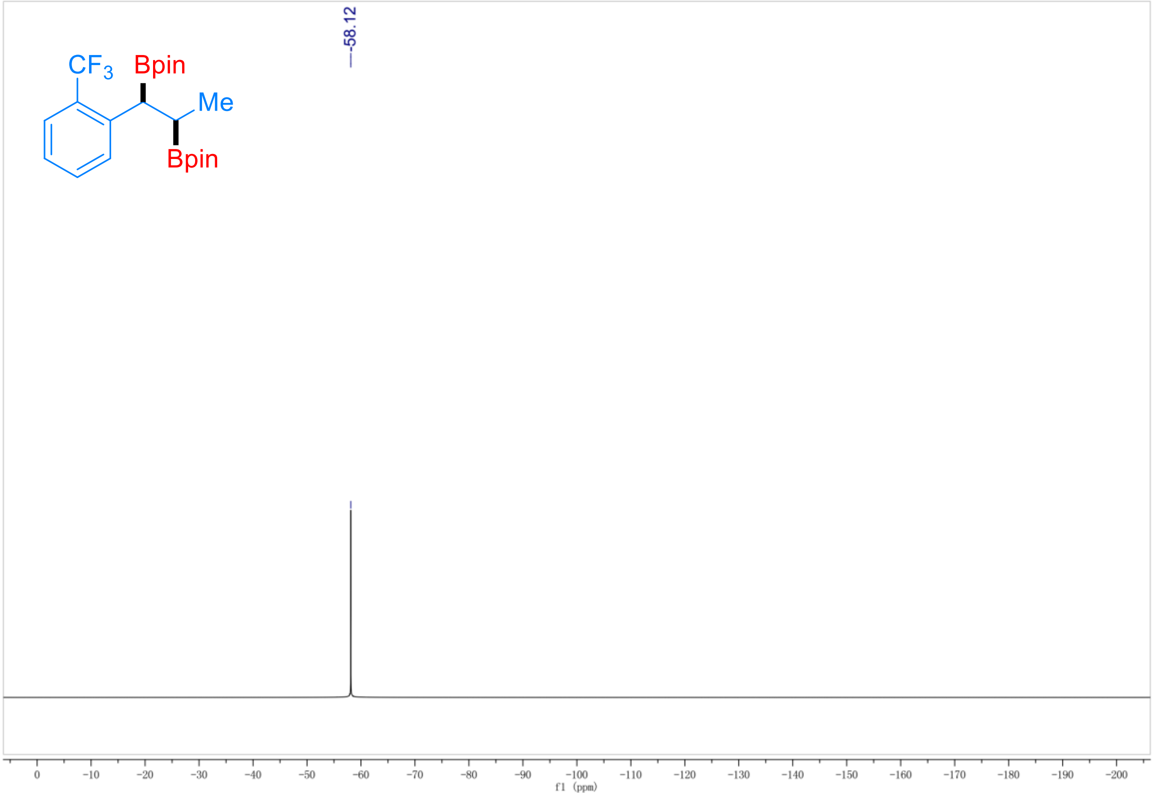

^{19}F{^{1}H} NMR spectrum of compound **16b** in $CDCl_3$ (470 MHz).

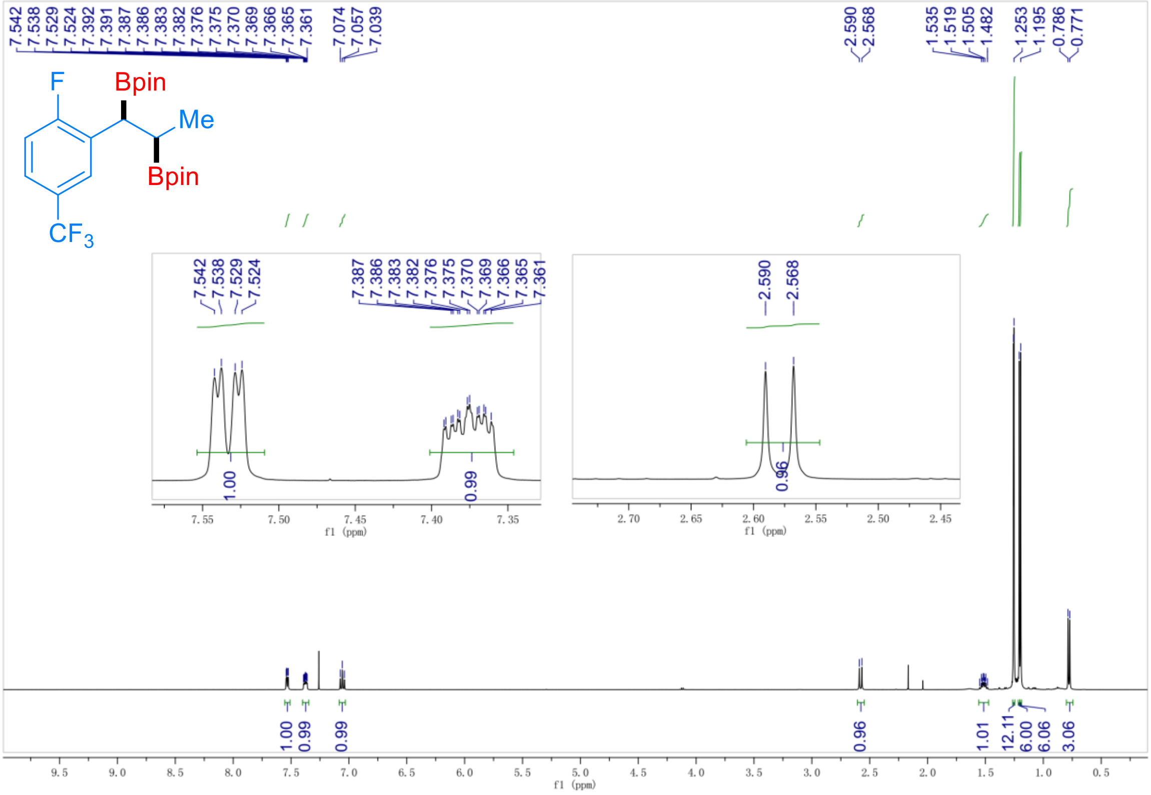

^{1}H NMR spectrum of compound **17b** in $CDCl_3$ (500 MHz).

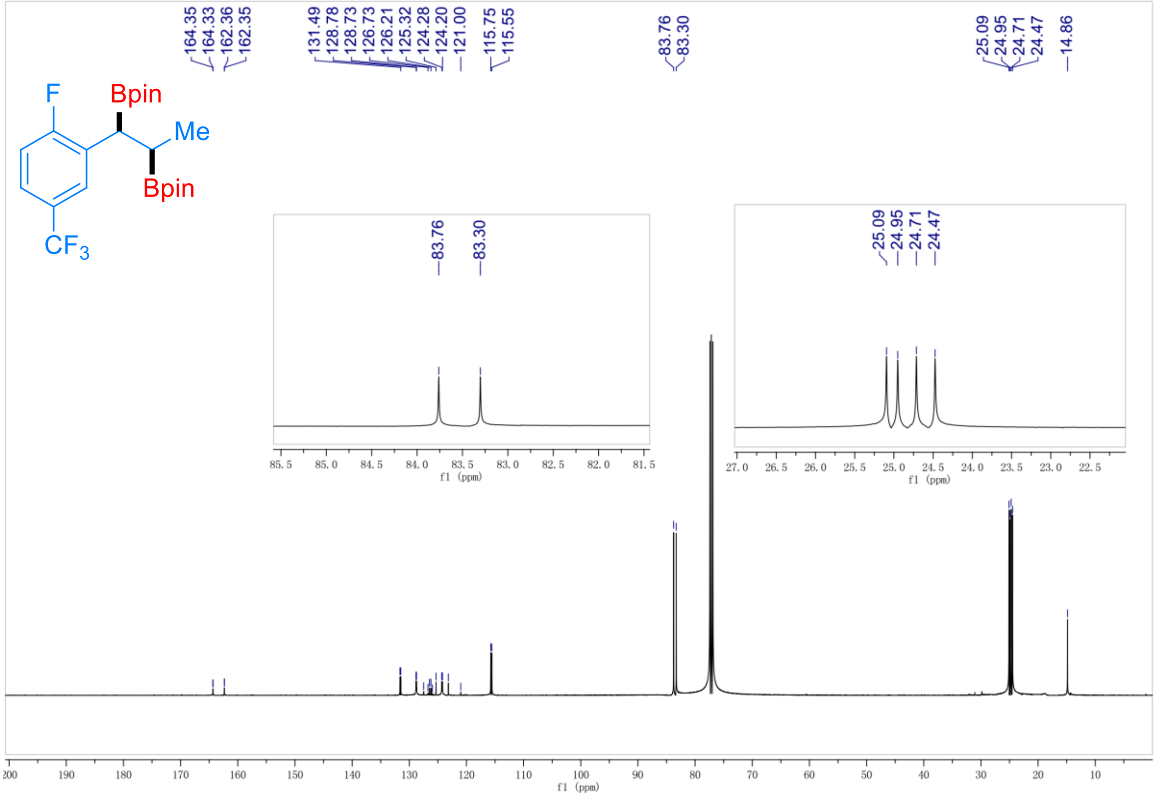

$^{13}C\{^1H\}$ NMR spectrum of compound **17b** in $CDCl_3$ (125 MHz).

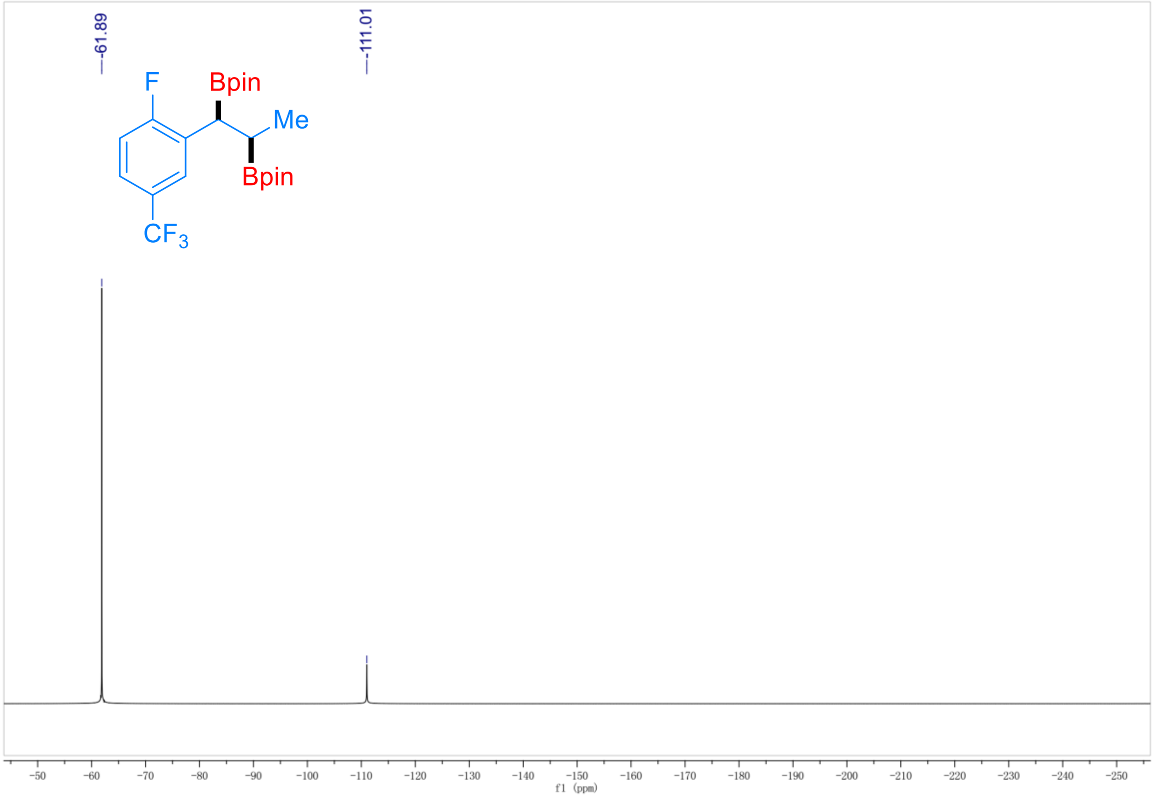

$^{19}F\{^1H\}$ NMR spectrum of compound **17b** in $CDCl_3$ (470 MHz).

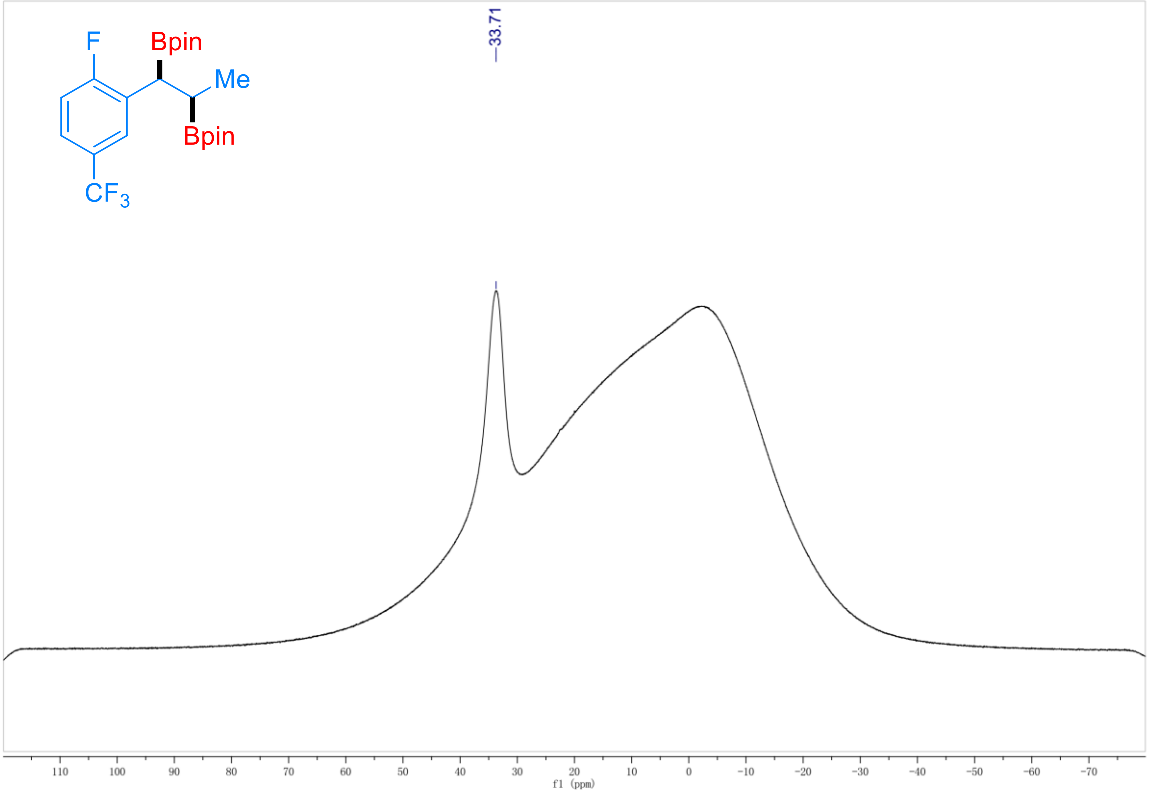

$^{11}B\{^{1}H\}$ NMR spectrum of compound **17b** in $CDCl_3$ (160 MHz).

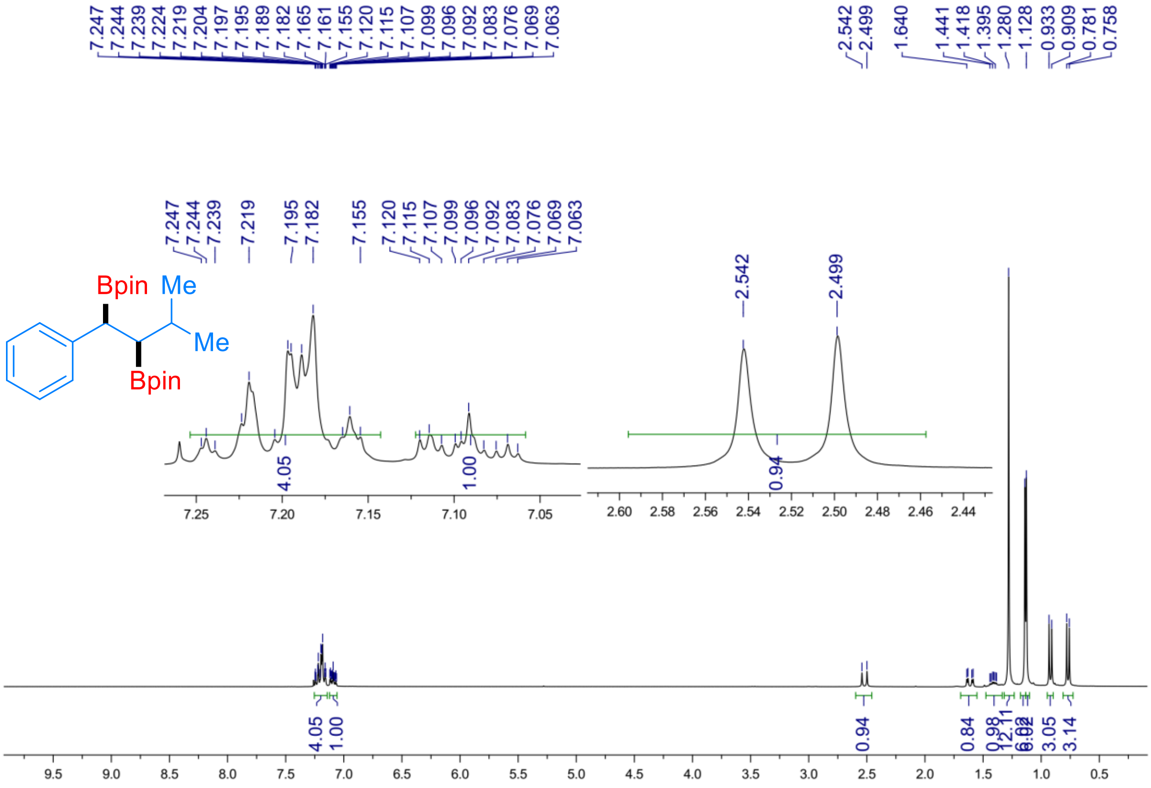

^{1}H NMR spectrum of compound **18b** in $CDCl_3$ (500 MHz).

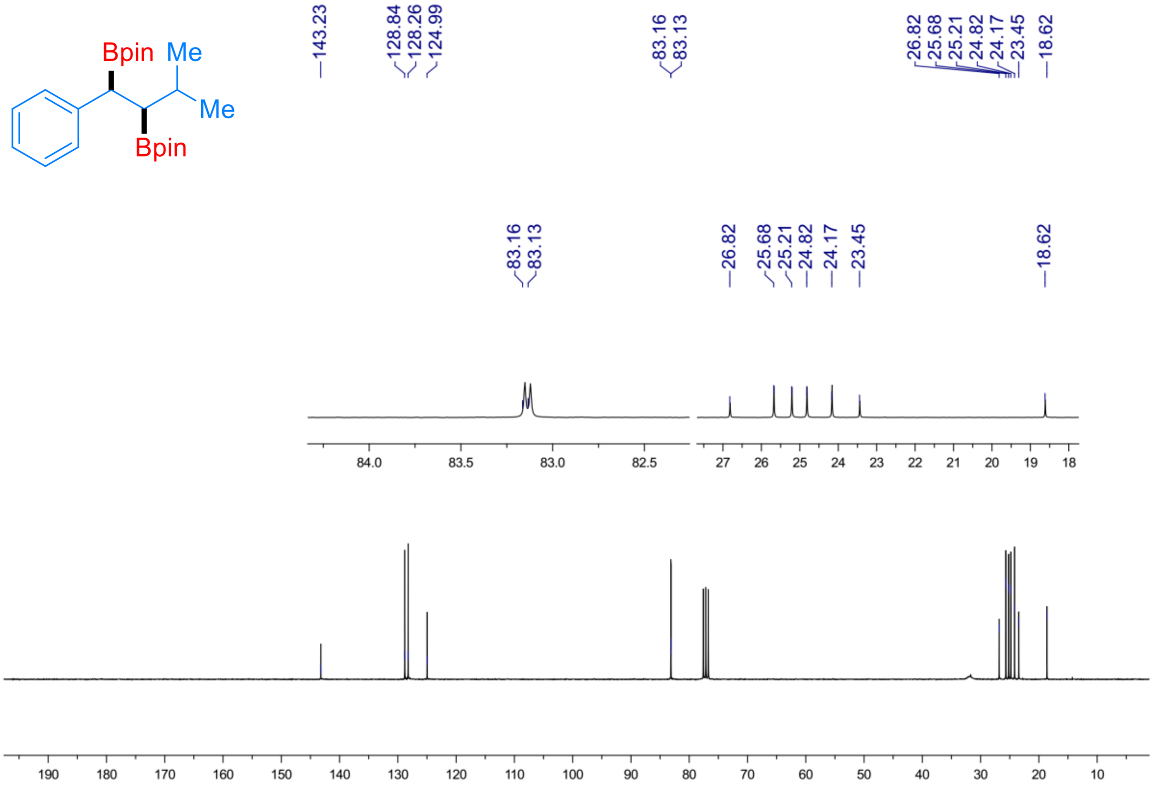

$^{13}C\{^{1}H\}$ NMR spectrum of compound **18b** in $CDCl_3$ (125 MHz).

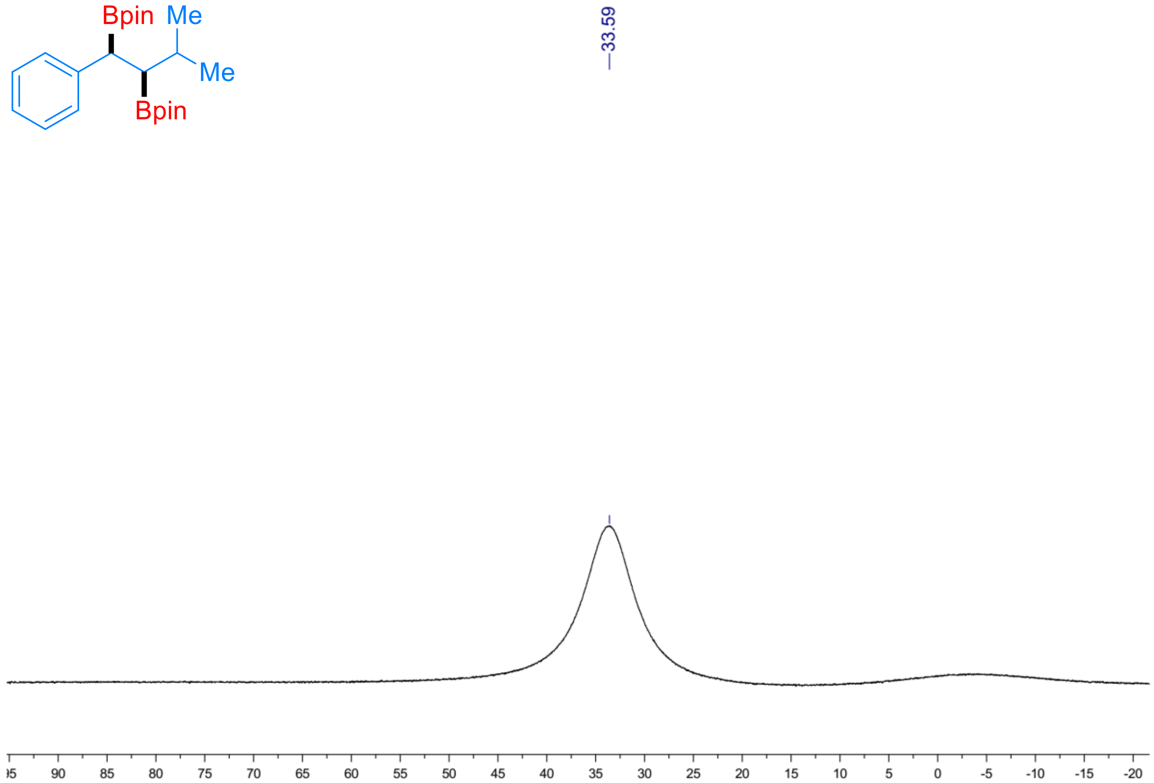

$^{11}B\{^{1}H\}$ NMR spectrum of compound **18b** in $CDCl_3$ (160 MHz).

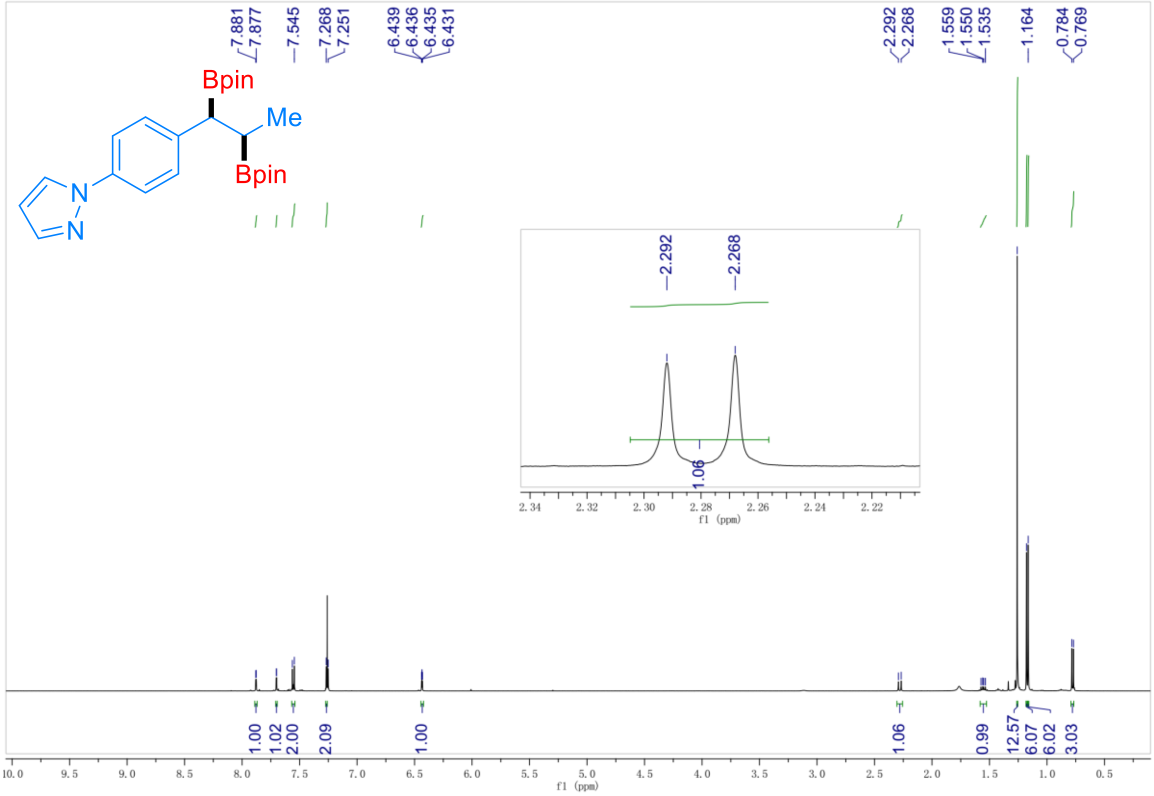

^{1}H NMR spectrum of compound **19b** in $CDCl_3$ (500 MHz).

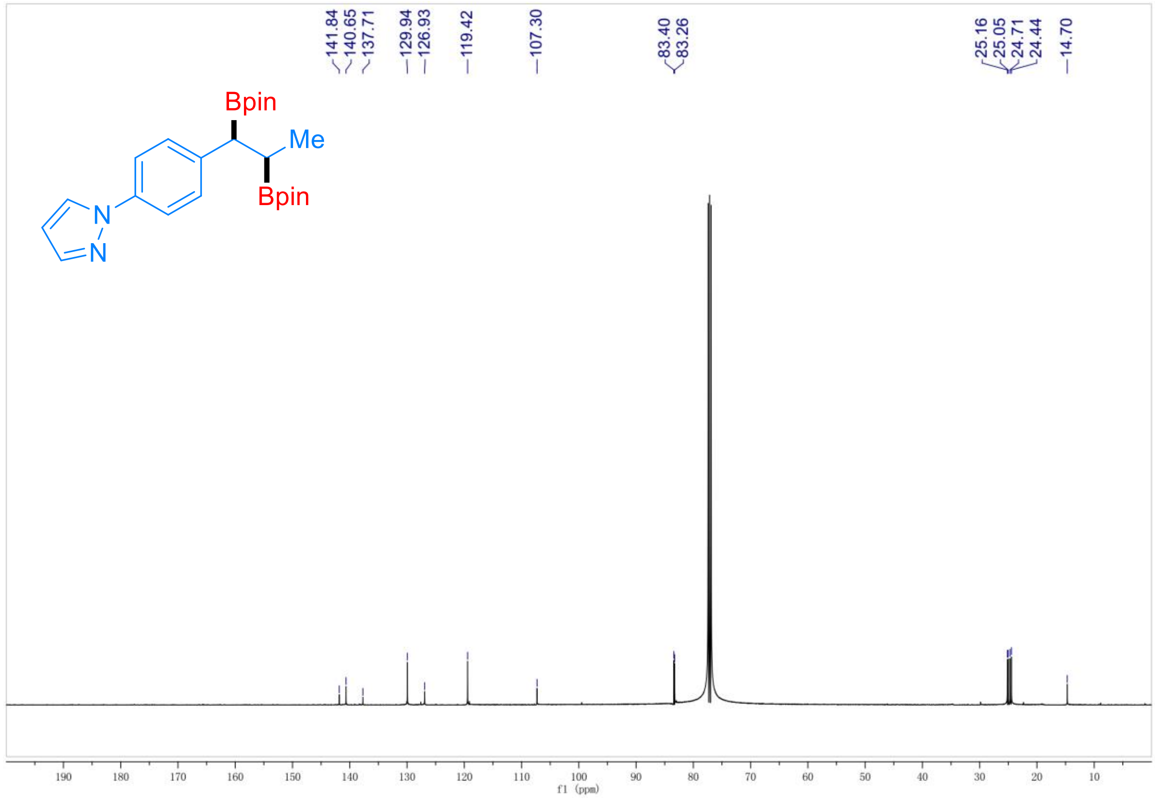

^{13}C{^{1}H} NMR spectrum of compound **19b** in $CDCl_3$ (125 MHz).

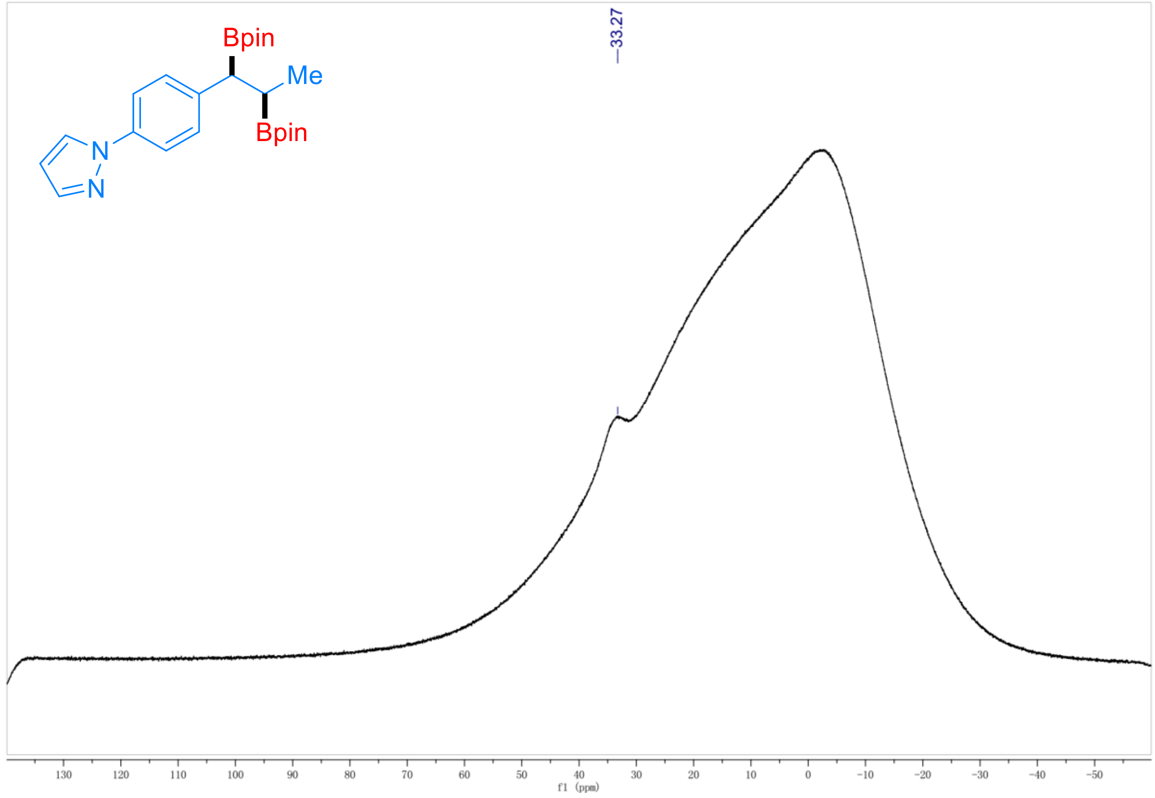

$^{11}B\{^{1}H\}$ NMR spectrum of compound **19b** in $CDCl_3$ (160 MHz).

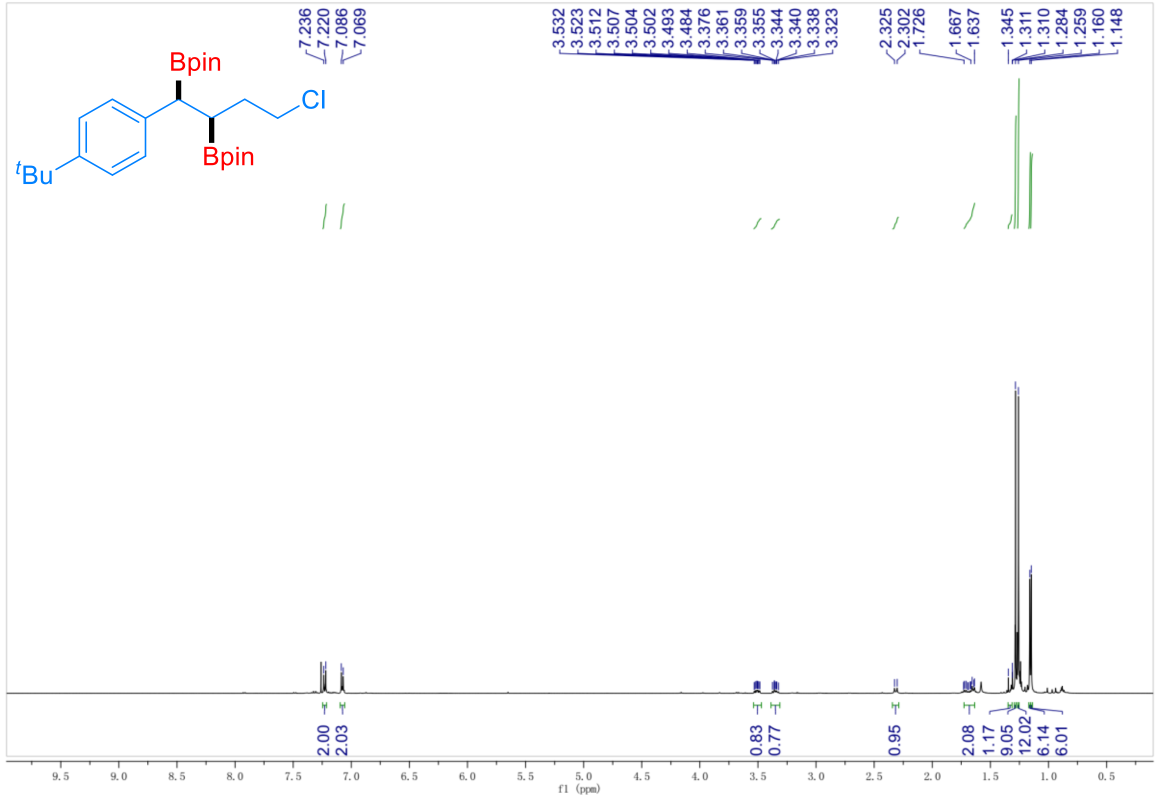

^{1}H NMR spectrum of compound **20b** in $CDCl_3$ (500 MHz).

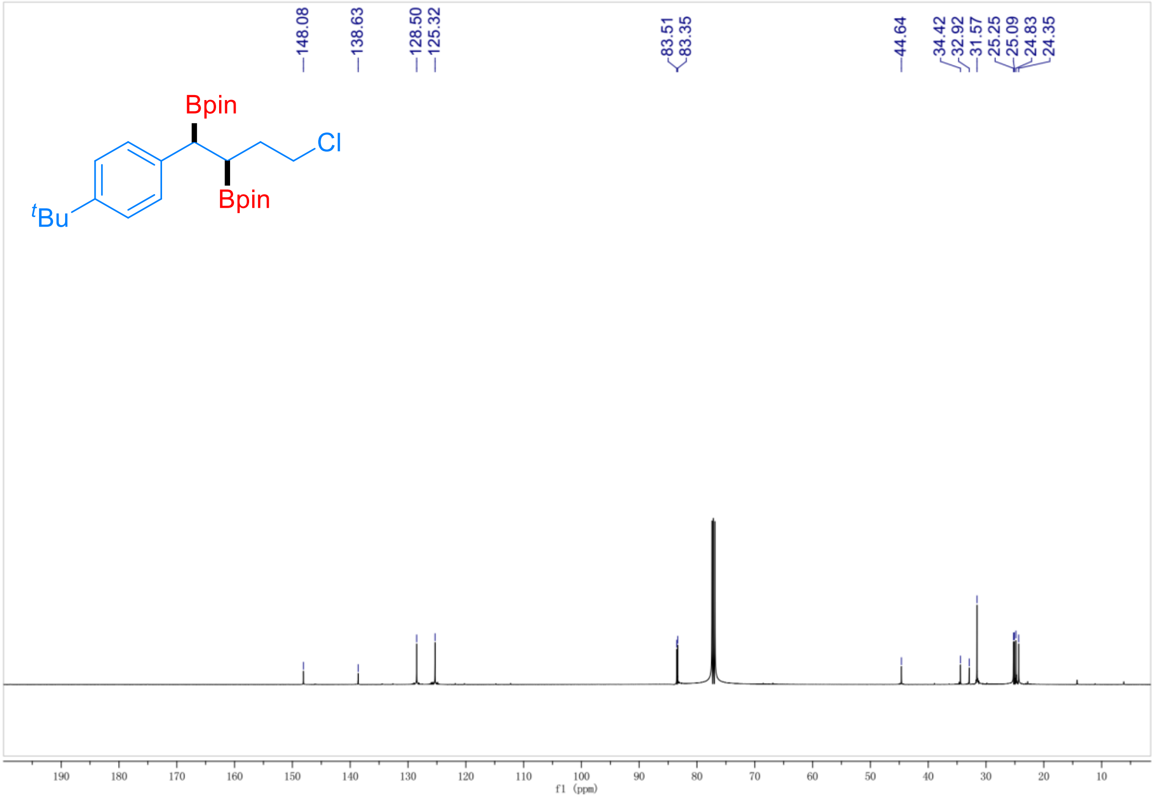

$^{13}C\{^{1}H\}$ NMR spectrum of compound **20b** in $CDCl_3$ (125 MHz).

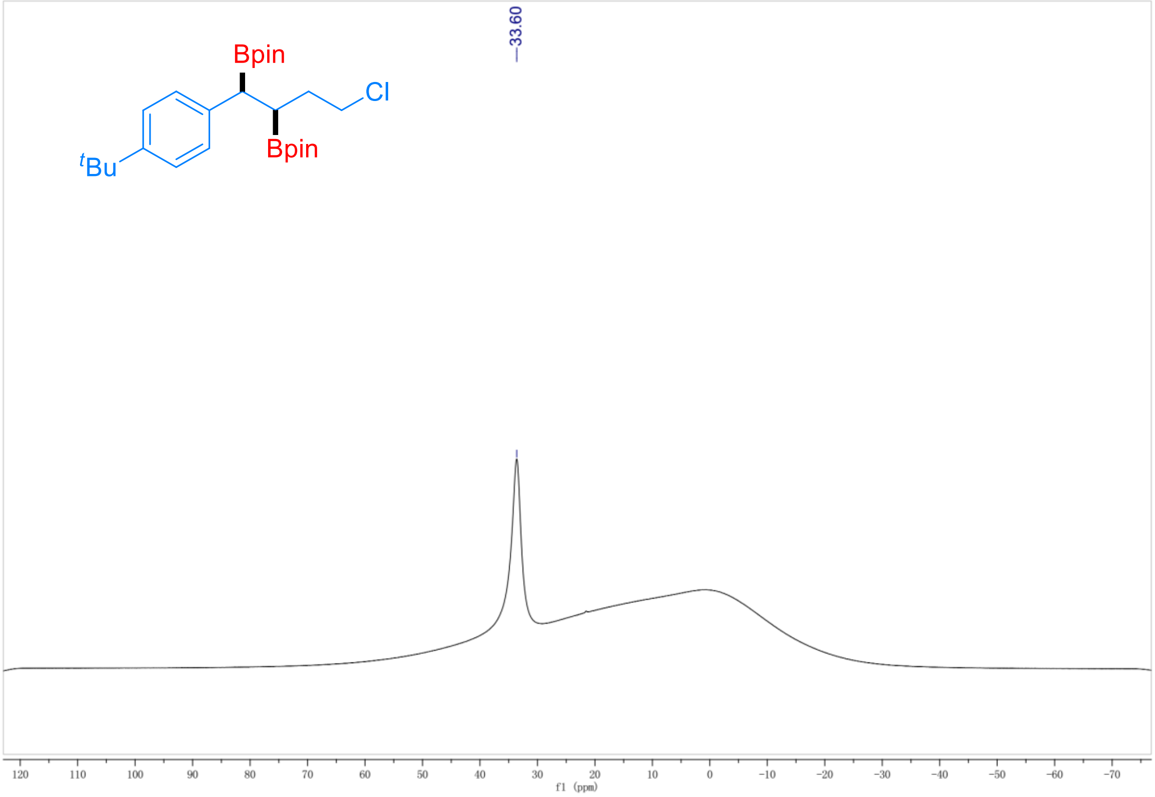

$^{11}B\{^{1}H\}$ NMR spectrum of compound **20b** in $CDCl_3$ (160 MHz).

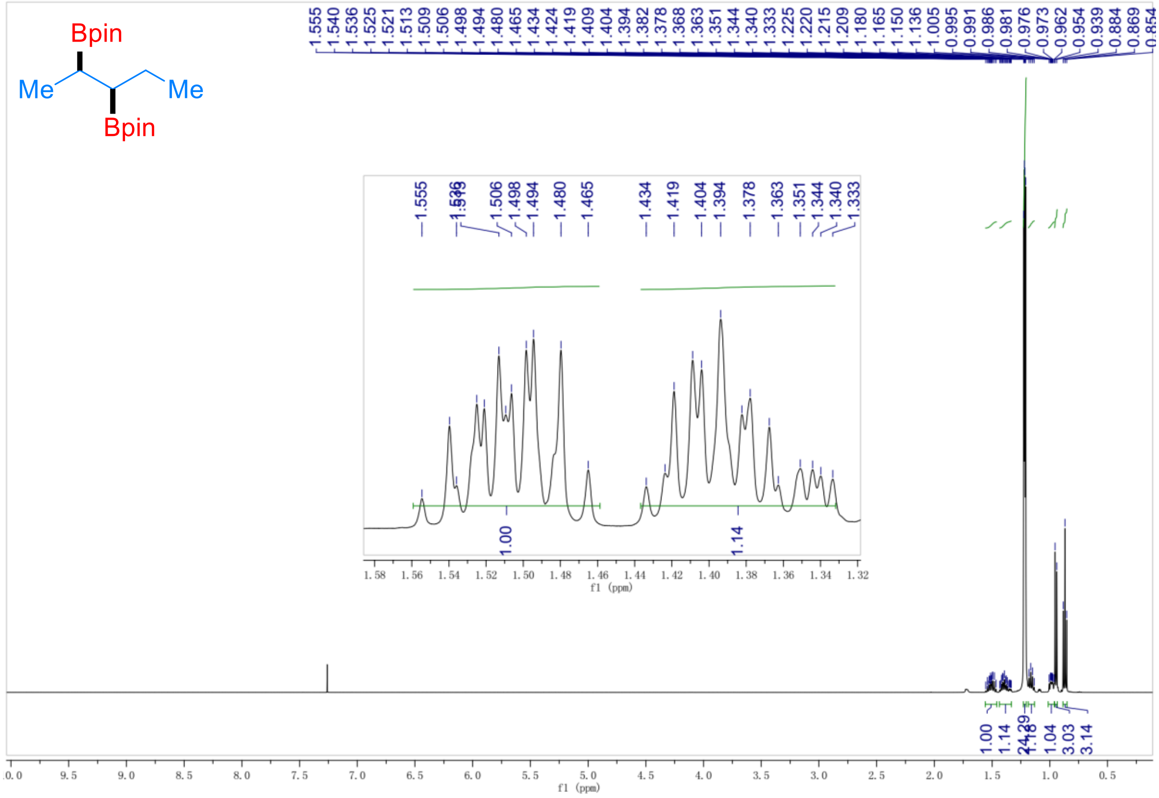

^{1}H NMR spectrum of compound **21b** in $CDCl_3$ (500 MHz).

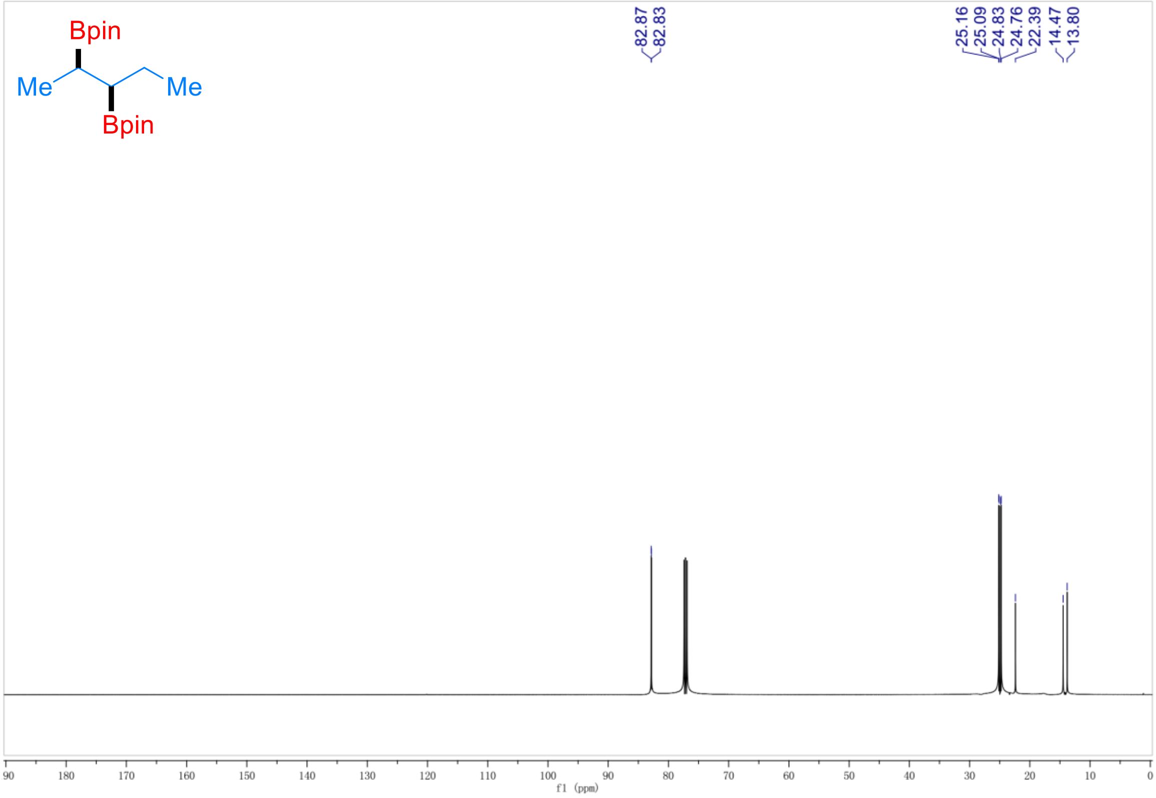

^{13}C{^{1}H} NMR spectrum of compound **21b** in $CDCl_3$ (125 MHz).

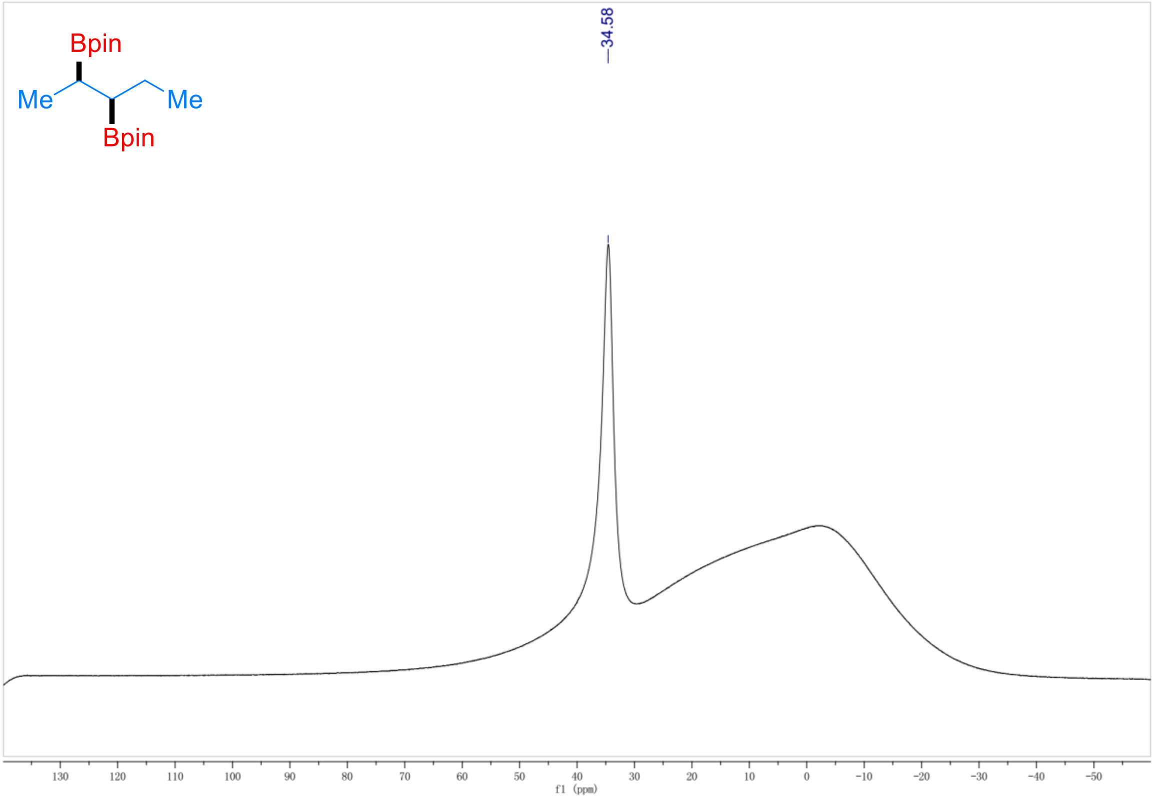

$^{11}B\{^{1}H\}$ NMR spectrum of compound **21b** in $CDCl_3$ (160 MHz).

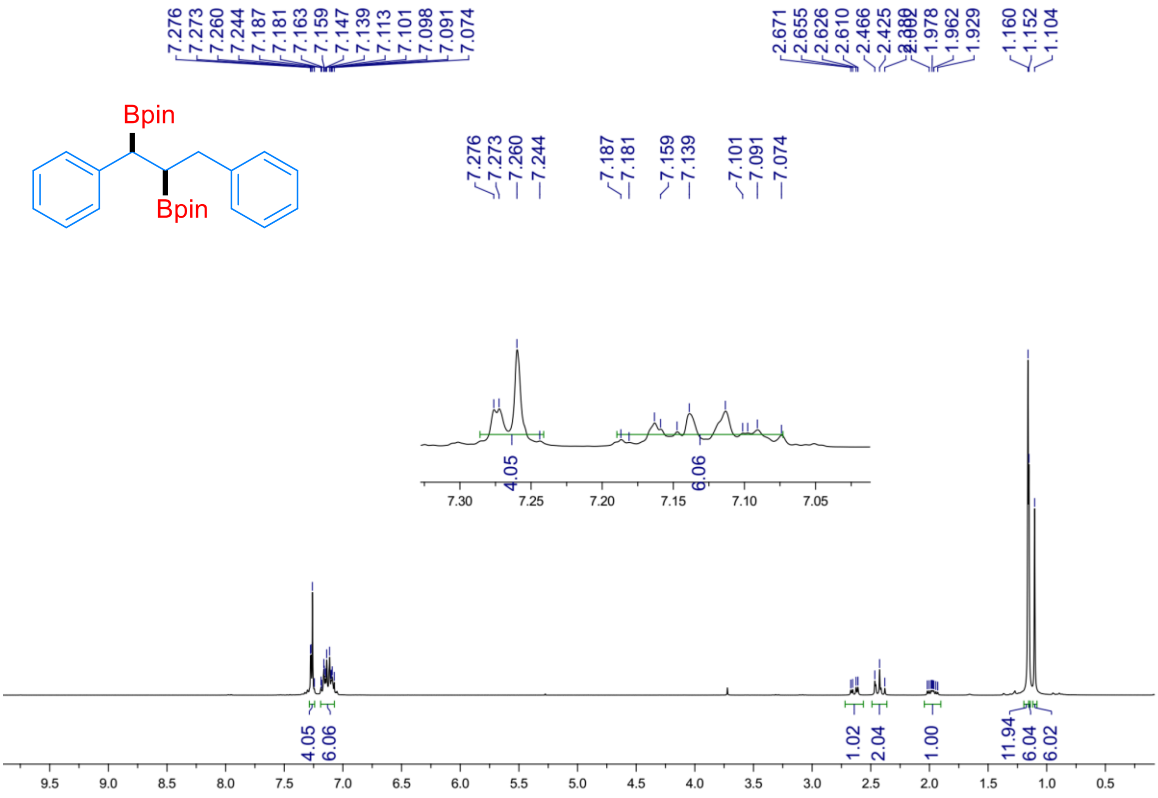

^{1}H NMR spectrum of compound **22b** in $CDCl_3$ (400 MHz).

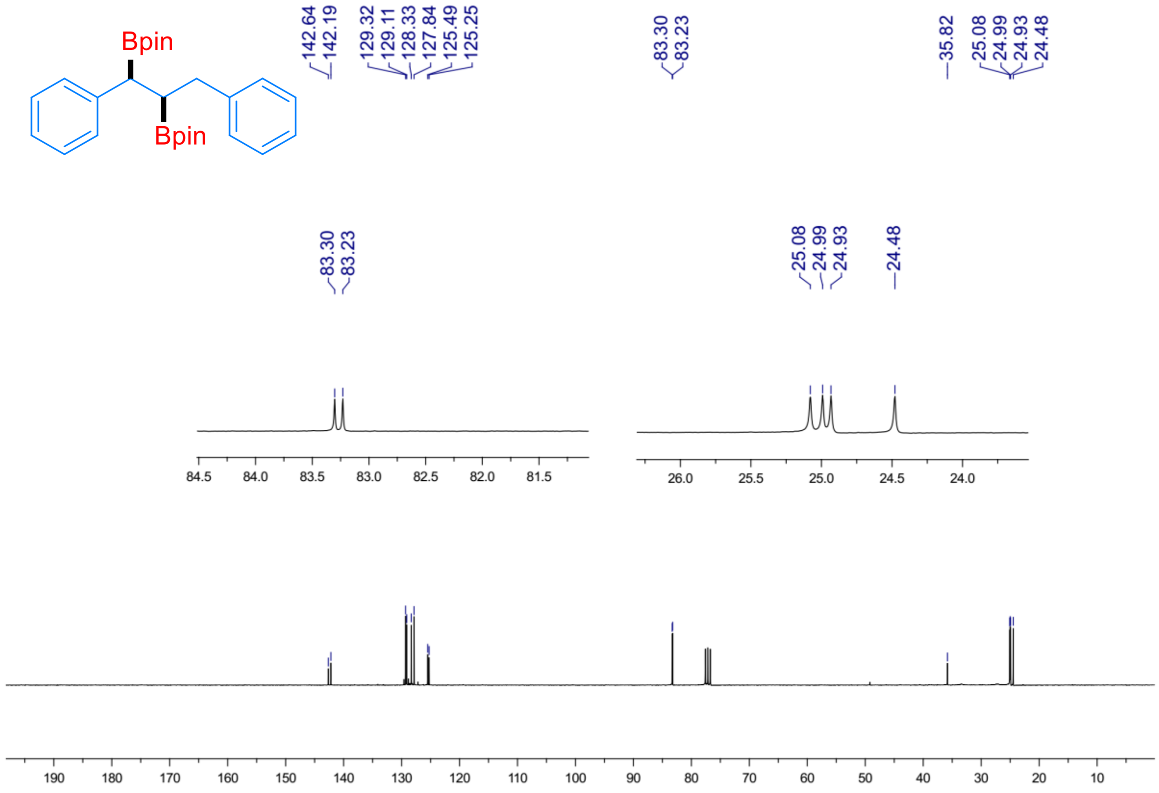

$^{13}C\{^{1}H\}$ NMR spectrum of compound **22b** in $CDCl_3$ (100 MHz).

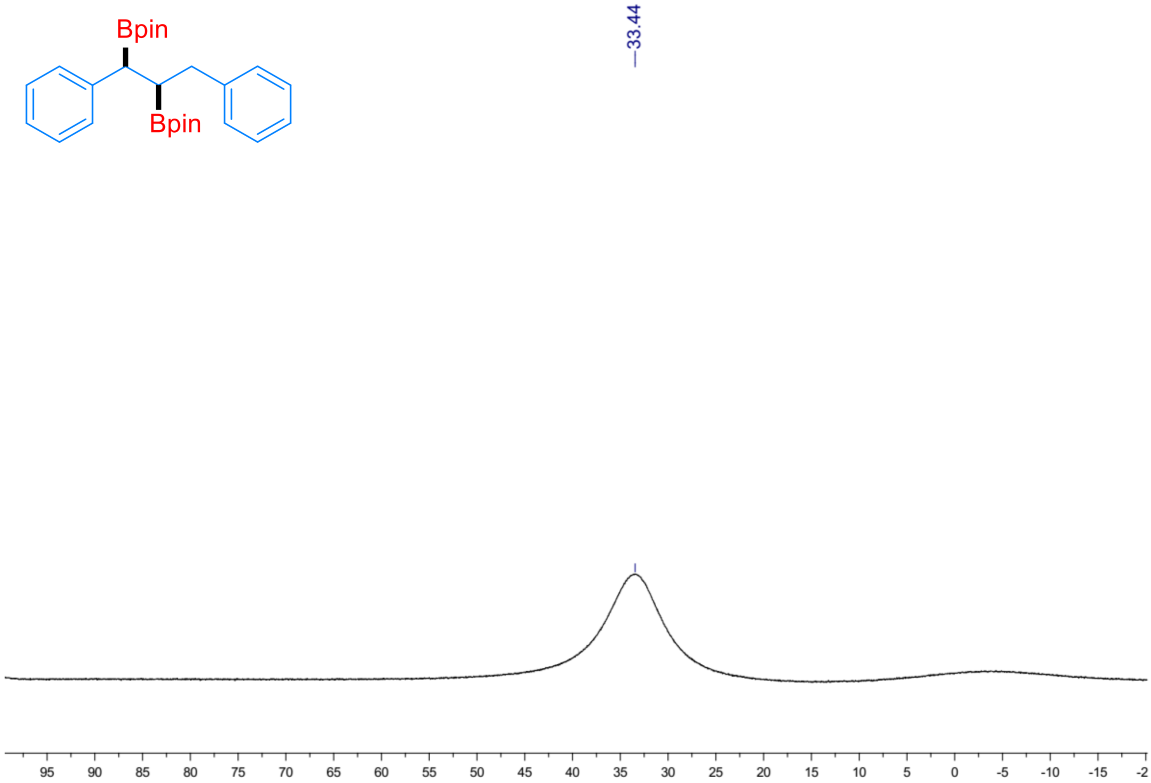

$^{11}B\{^{1}H\}$ NMR spectrum of compound **22b** in $CDCl_3$ (128 MHz).

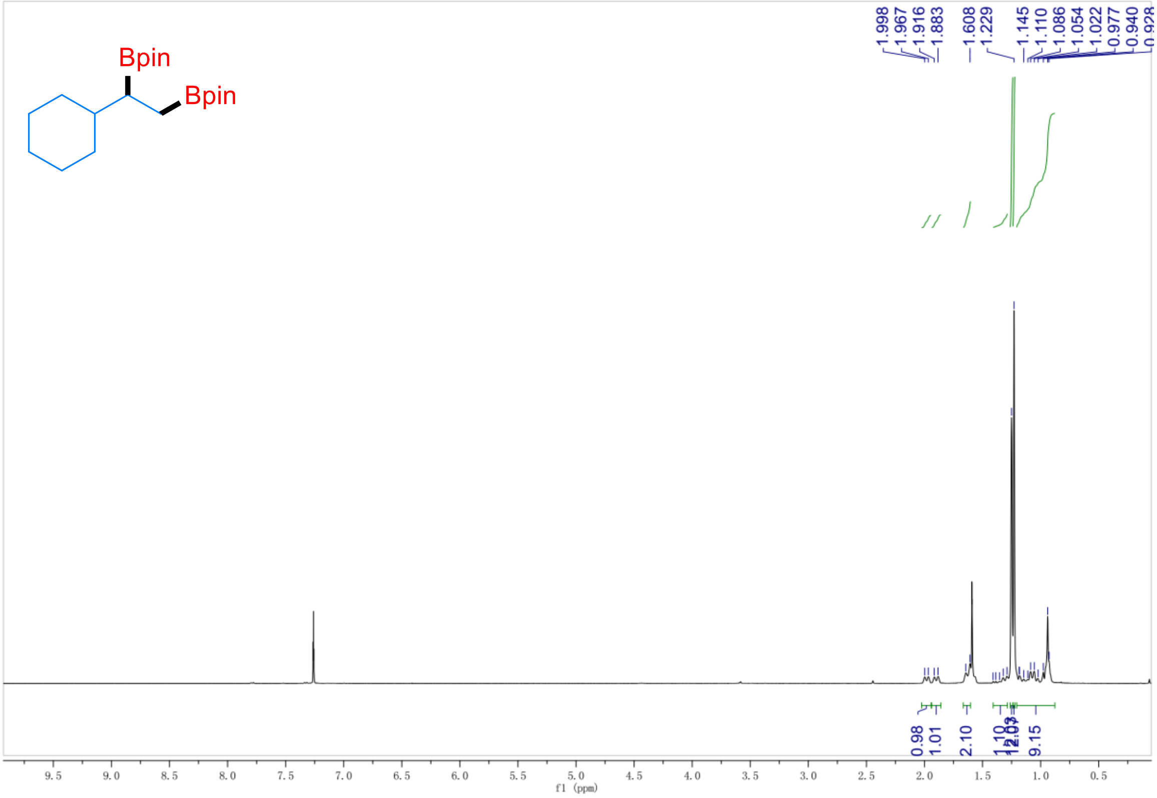

^{1}H NMR spectrum of compound **23b** in $CDCl_3$ (400 MHz).

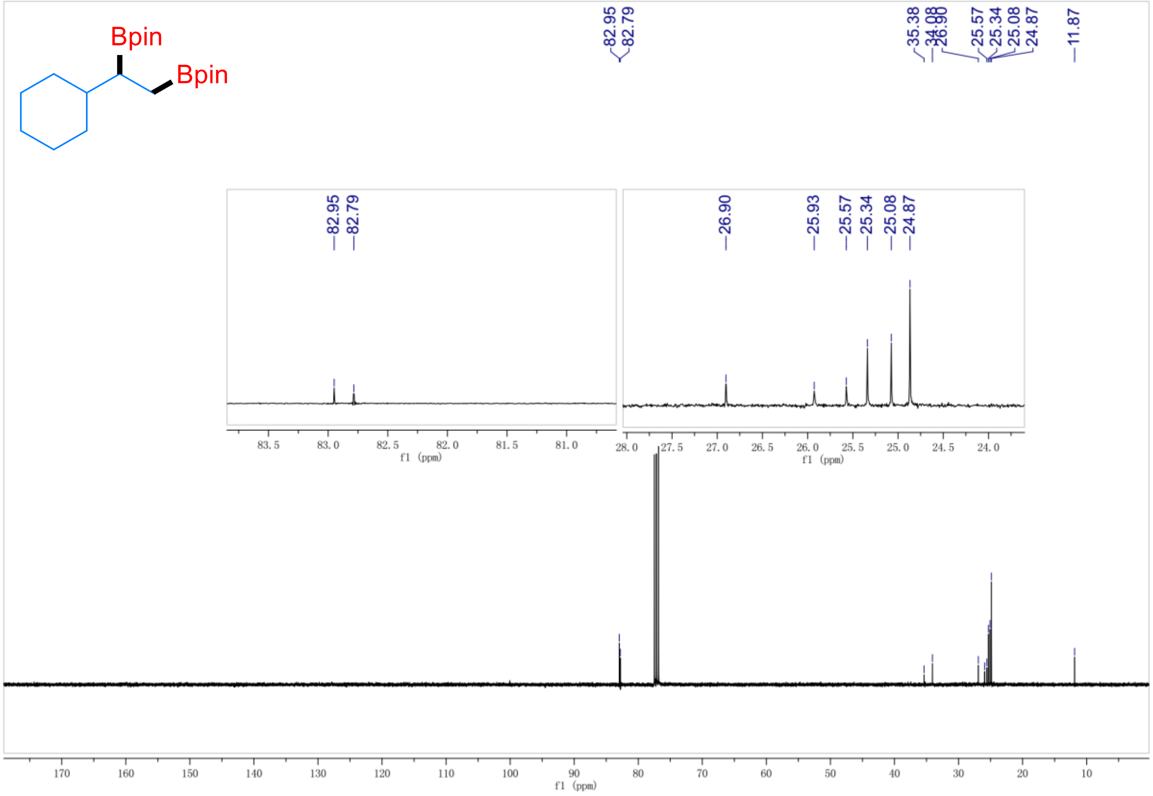

^{13}C{^{1}H} NMR spectrum of compound **23b** in $CDCl_3$ (100 MHz).

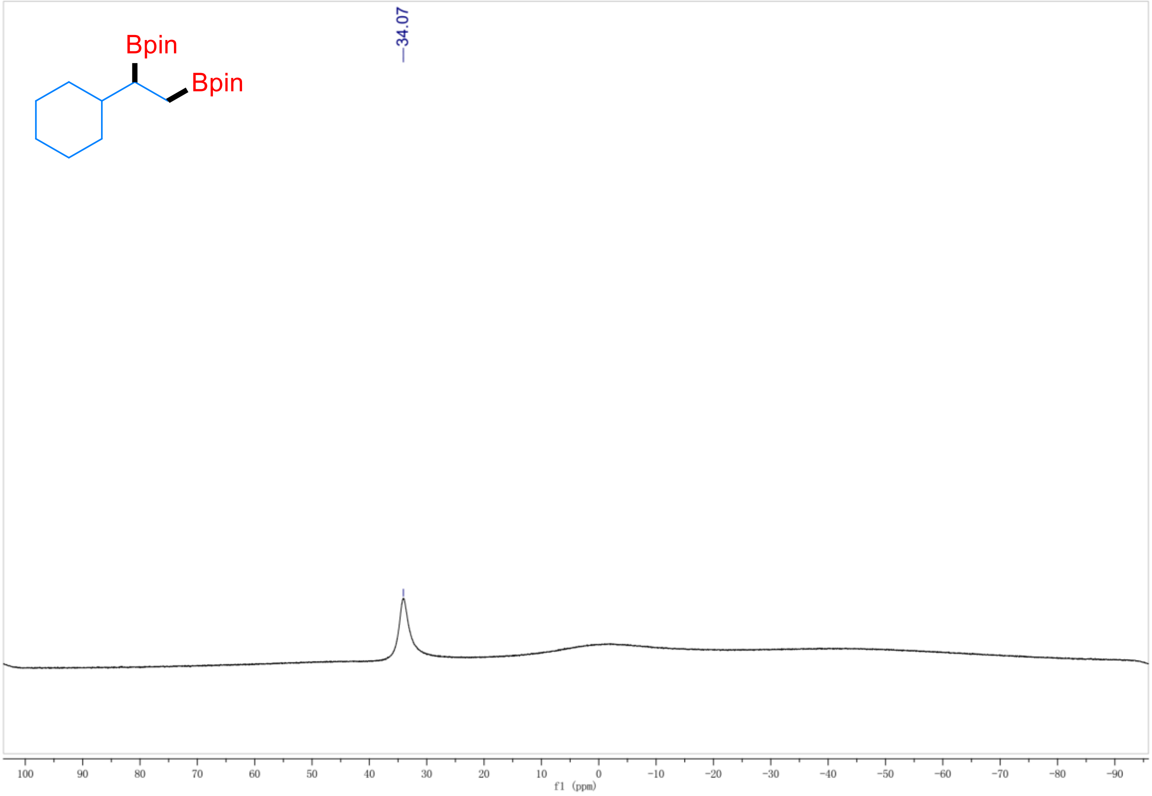

$^{11}B\{^{1}H\}$ NMR spectrum of compound **23b** in $CDCl_3$ (128 MHz).

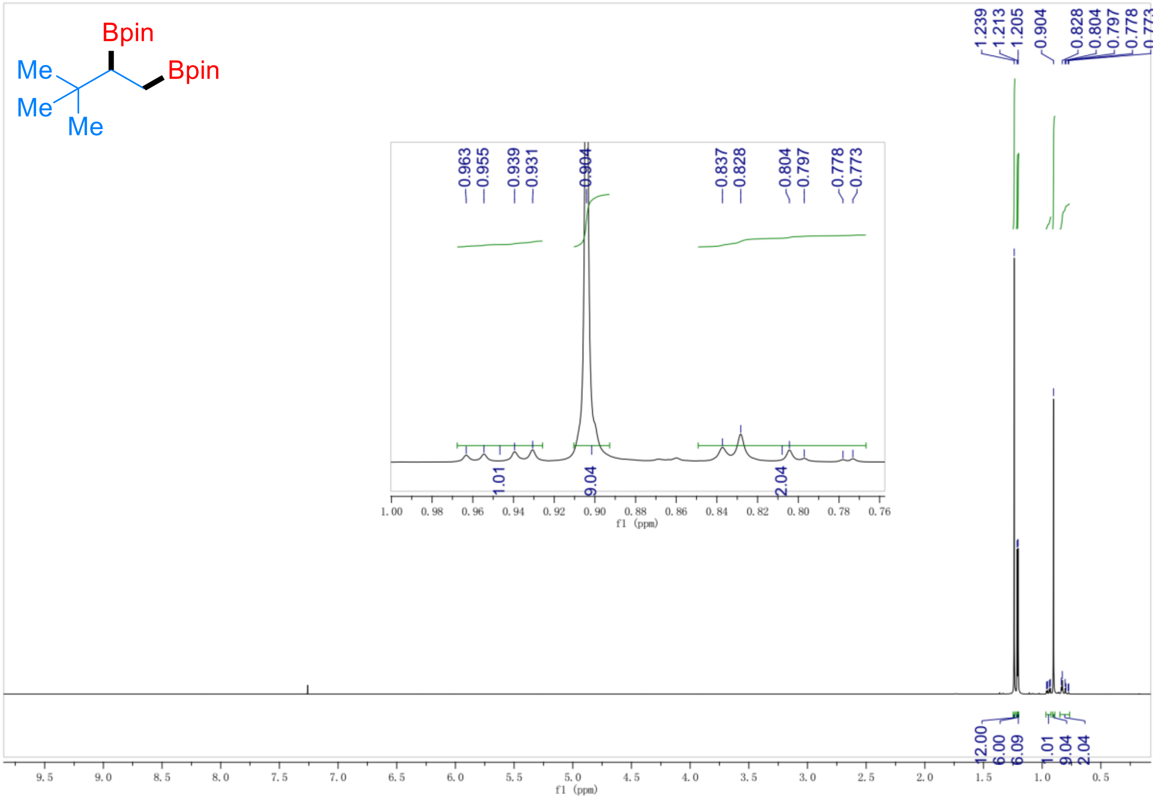

^{1}H NMR spectrum of compound **24b** in $CDCl_3$ (500 MHz).

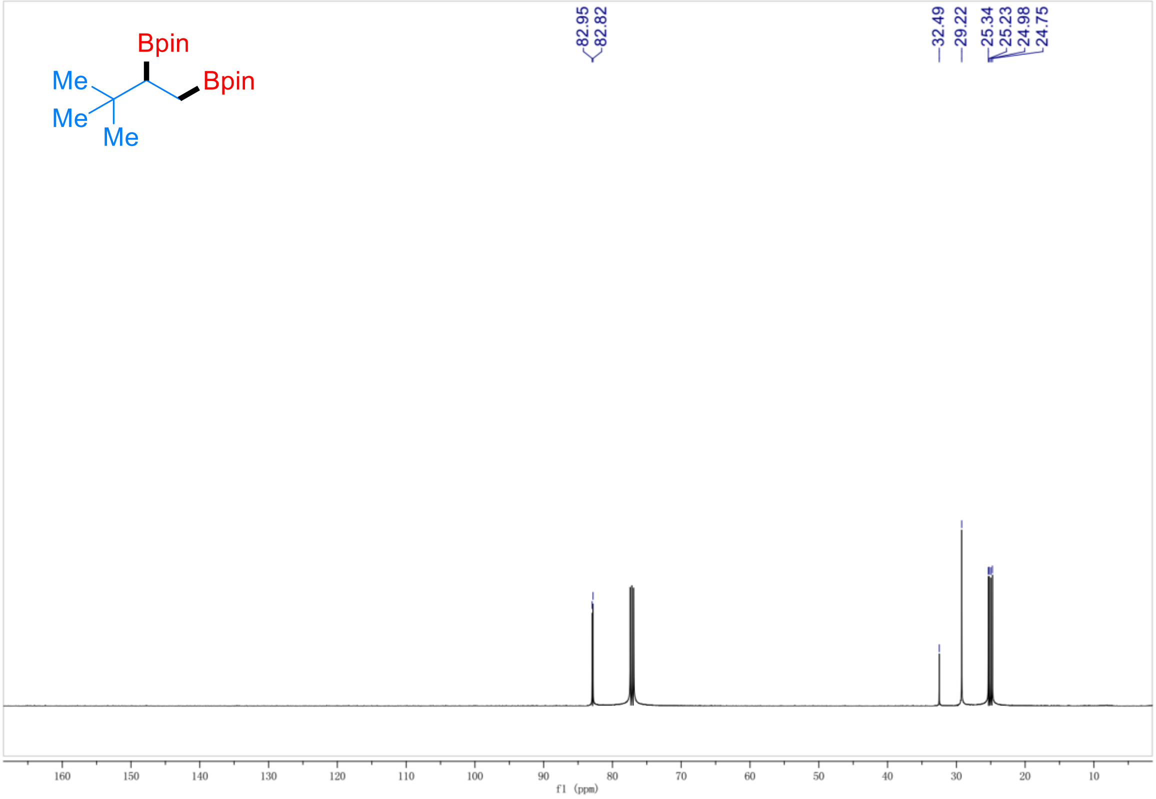

$^{13}C\{^{1}H\}$ NMR spectrum of compound **24b** in $CDCl_3$ (125 MHz).

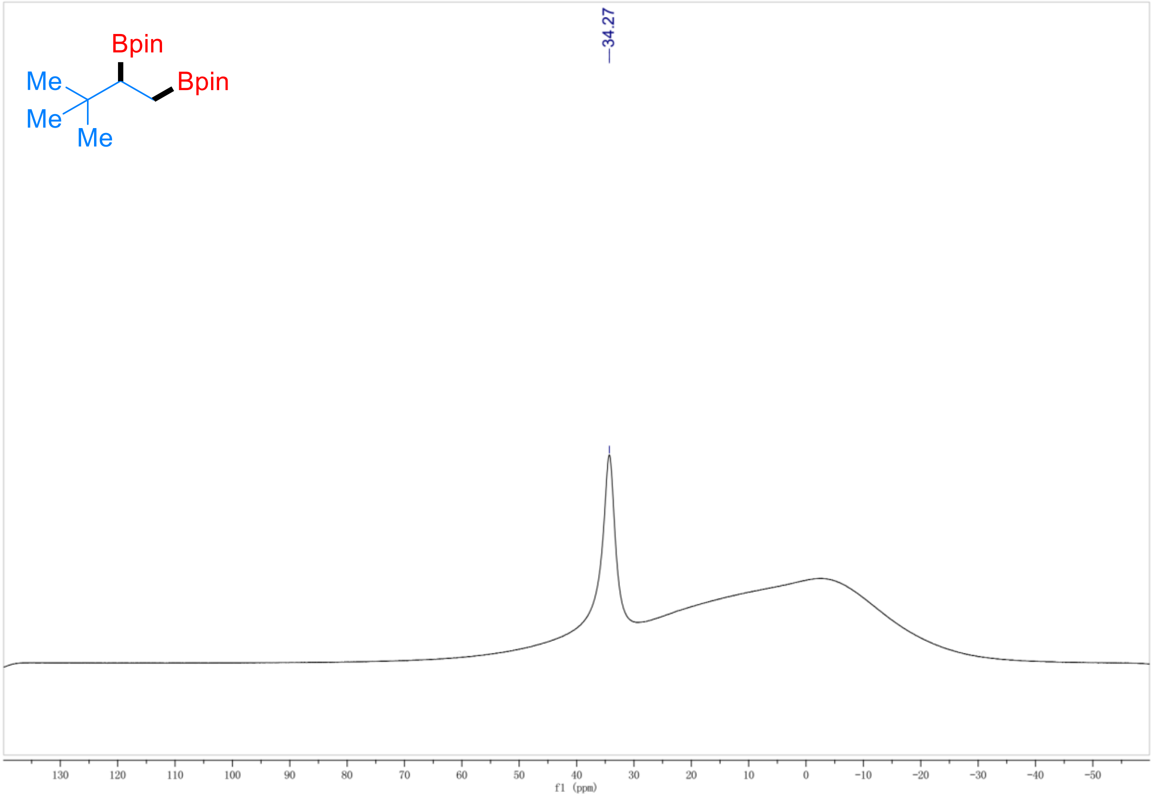

$^{11}B\{^{1}H\}$ NMR spectrum of compound **24b** in $CDCl_3$ (160 MHz).

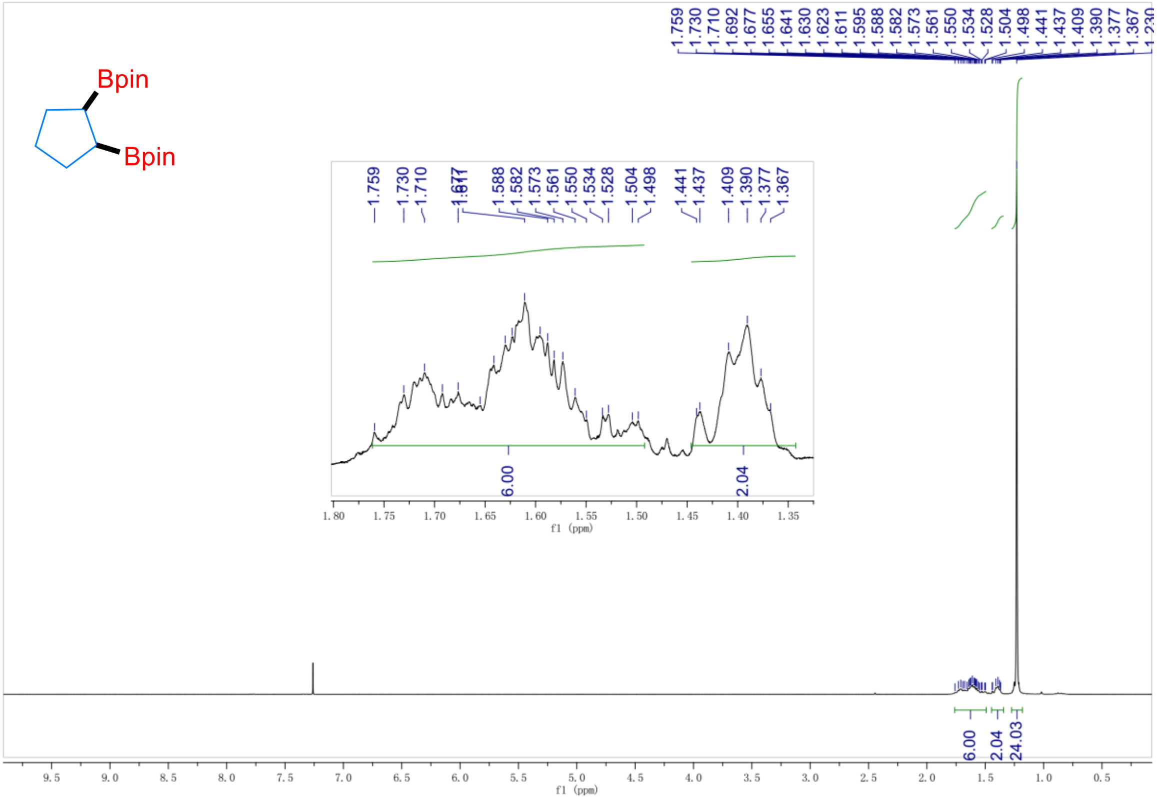

^{1}H NMR spectrum of compound **25b** in $CDCl_3$ (300 MHz).

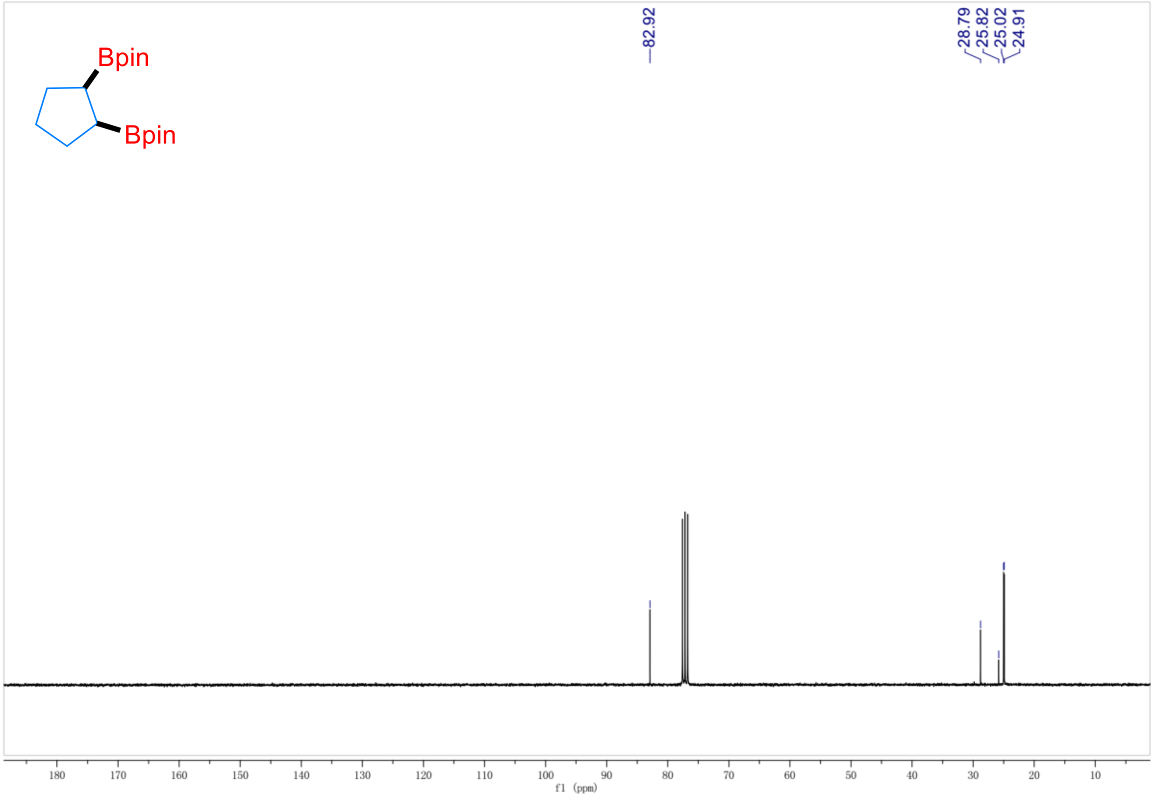

$^{13}C\{^{1}H\}$ NMR spectrum of compound **25b** in $CDCl_3$ (75 MHz).

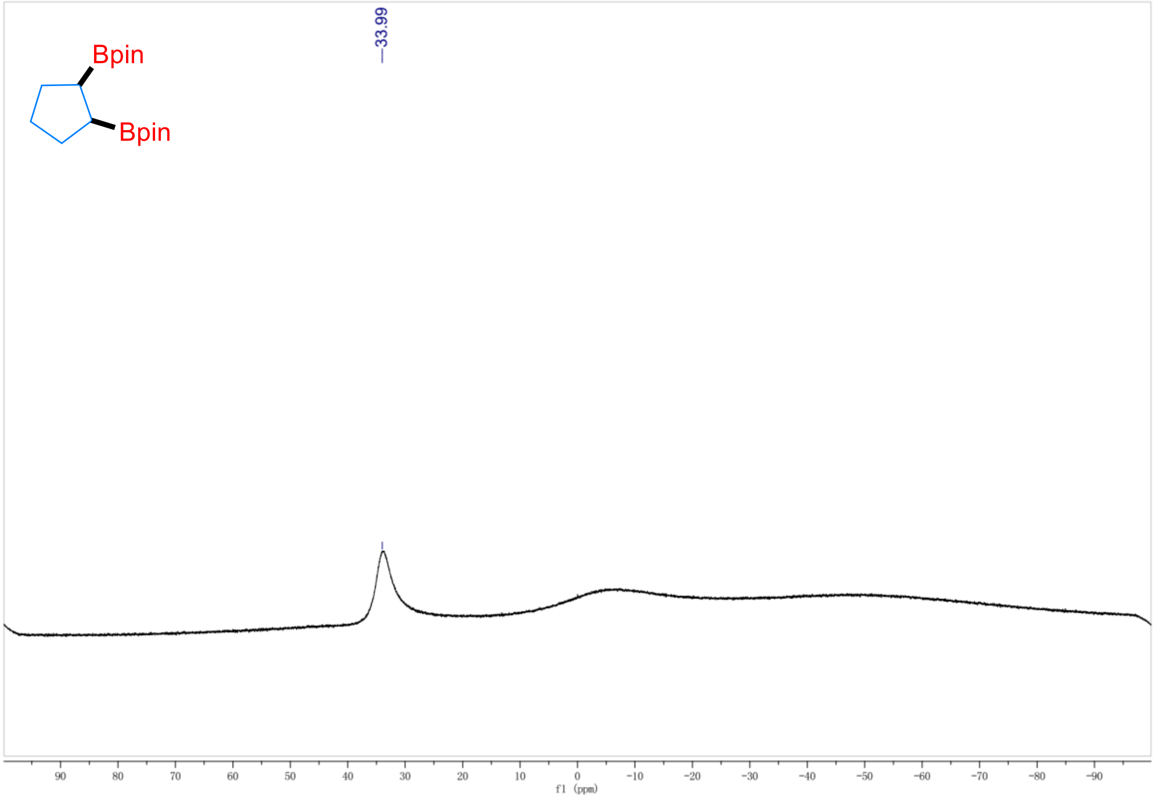

$^{11}B\{^{1}H\}$ NMR spectrum of compound **25b** in $CDCl_3$ (96 MHz).

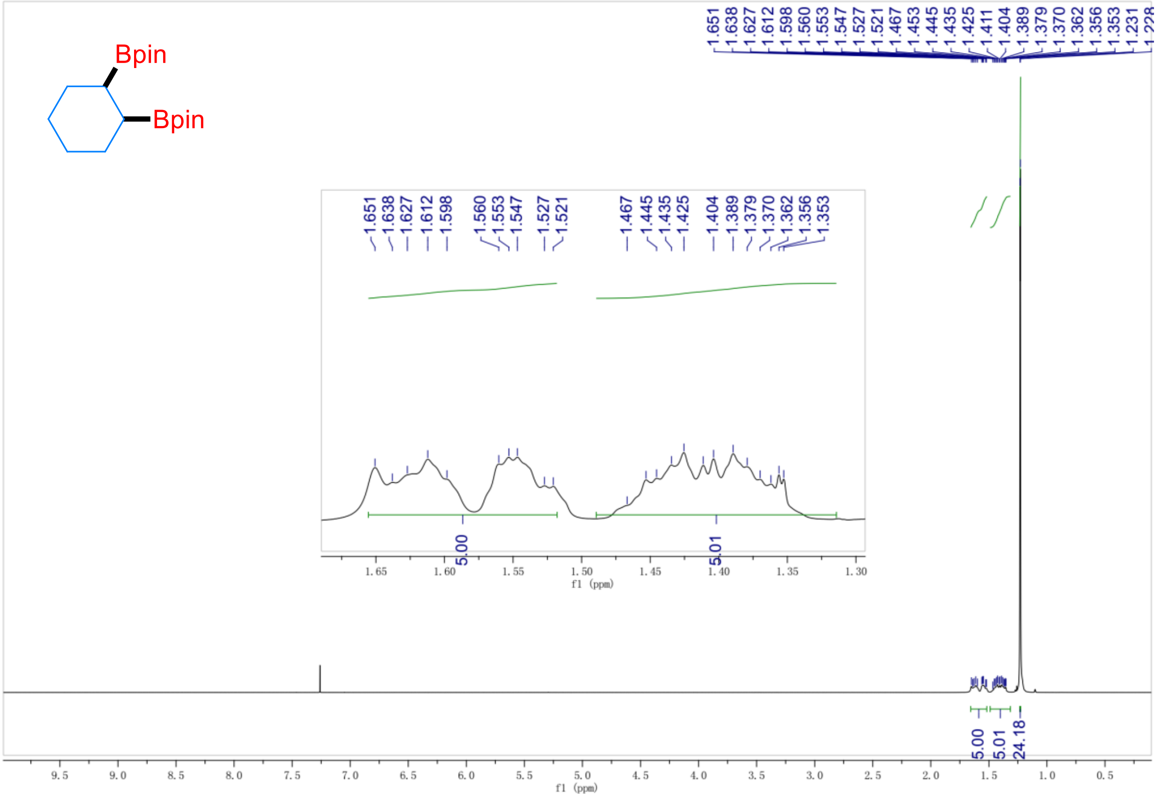

^{1}H NMR spectrum of compound **26b** in $CDCl_3$ (500 MHz).

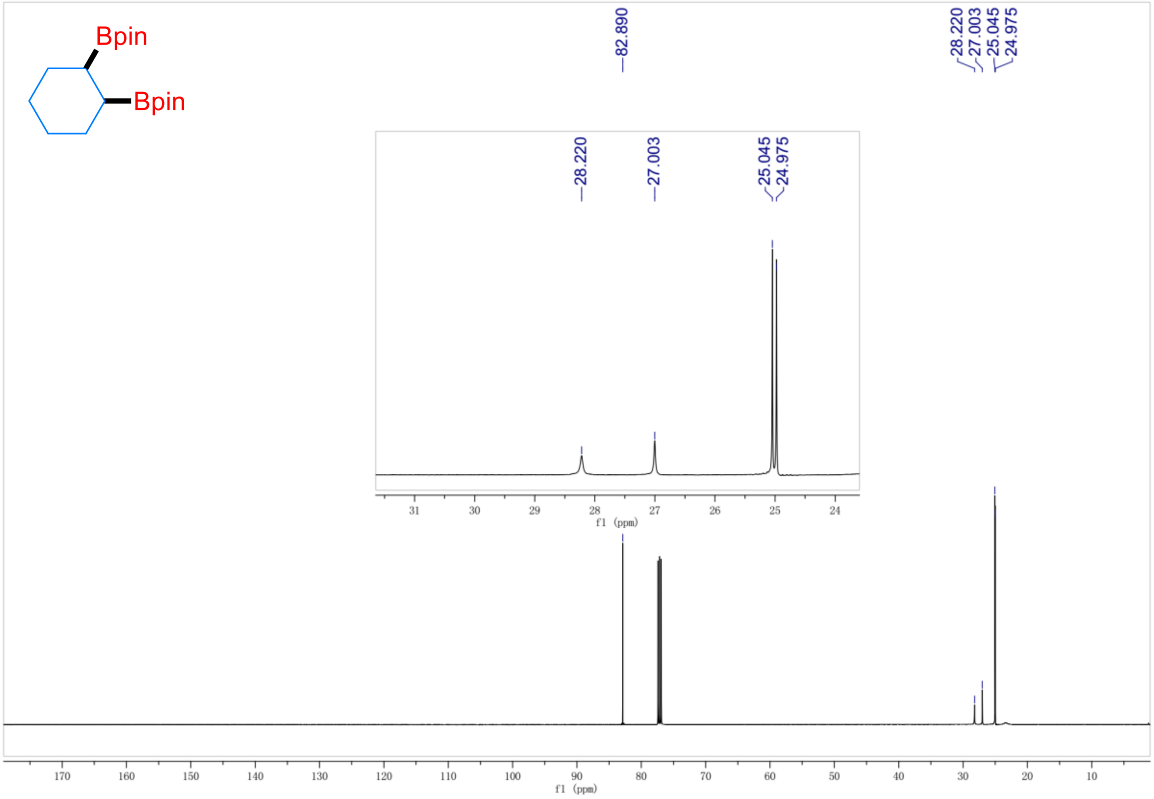

$^{13}C\{^1H\}$ NMR spectrum of compound **26b** in $CDCl_3$ (125 MHz).

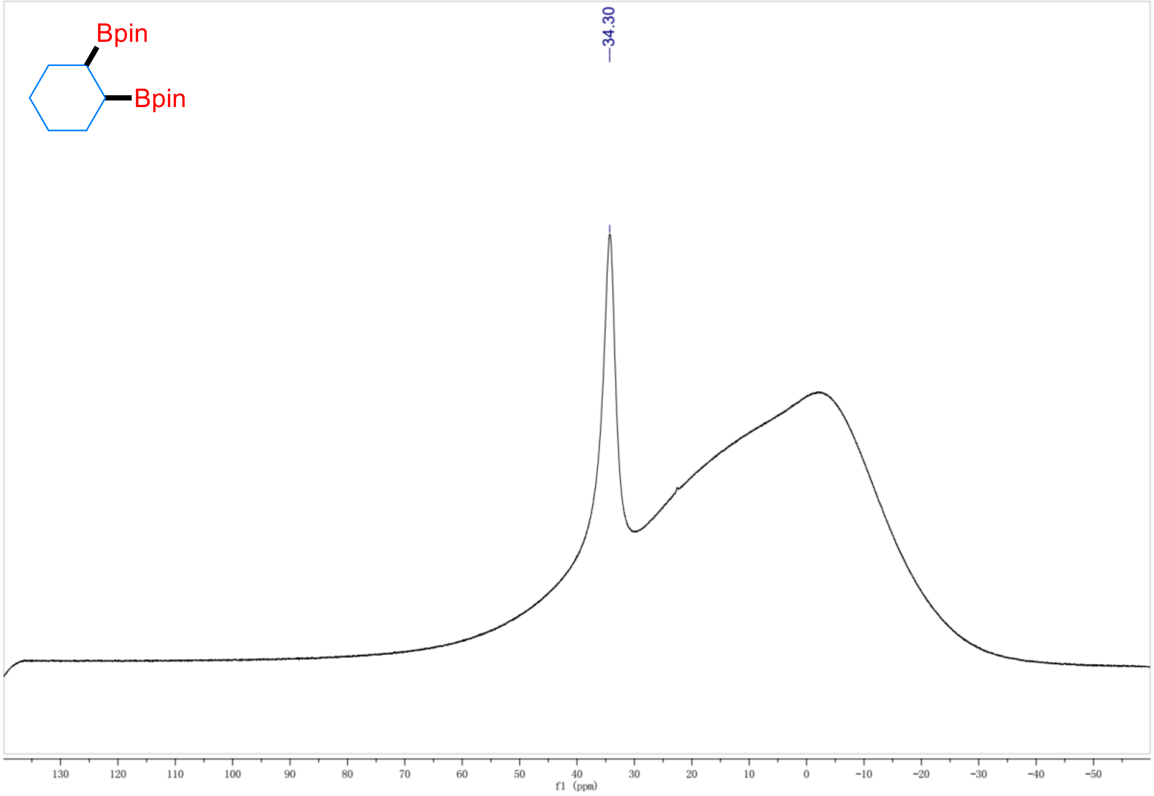

$^{11}B\{^1H\}$ NMR spectrum of compound **26b** in $CDCl_3$ (160 MHz).

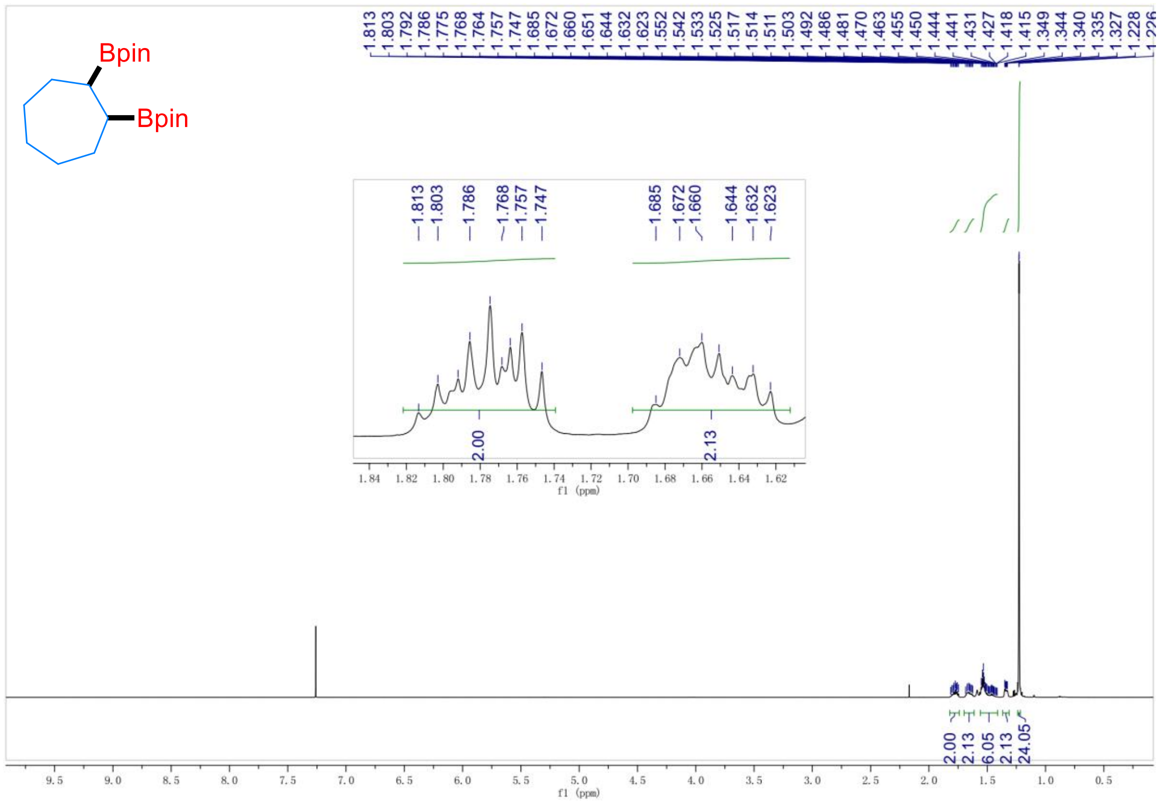

^{1}H NMR spectrum of compound **27b** in $CDCl_3$ (500 MHz).

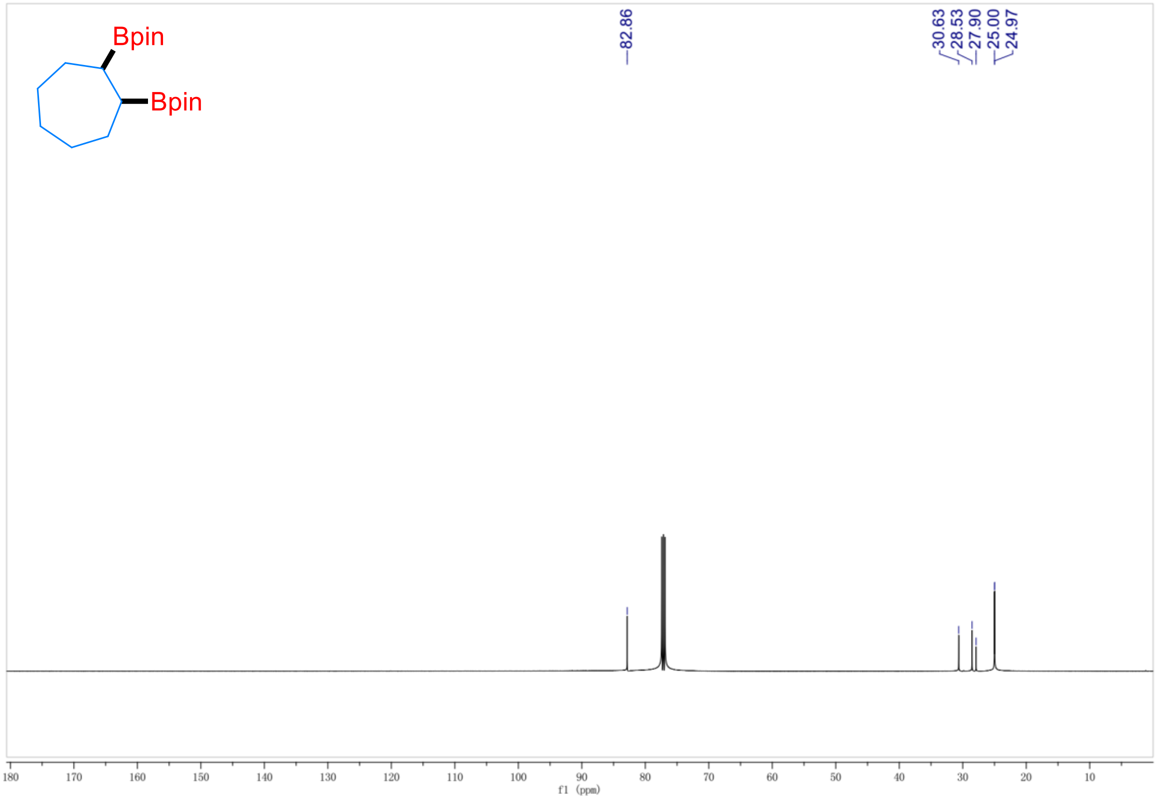

^{13}C{^{1}H} NMR spectrum of compound **27b** in $CDCl_3$ (125 MHz).

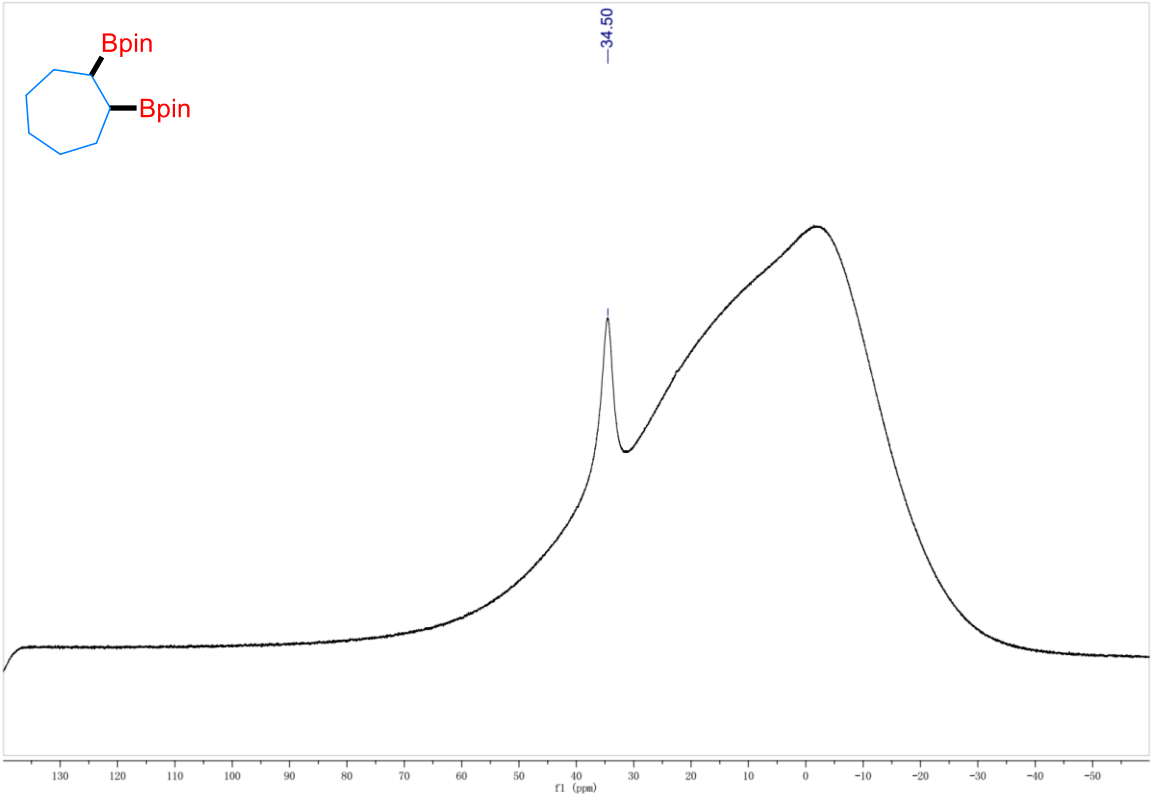

^{11}B{^{1}H} NMR spectrum of compound **27b** in $CDCl_3$ (160 MHz).

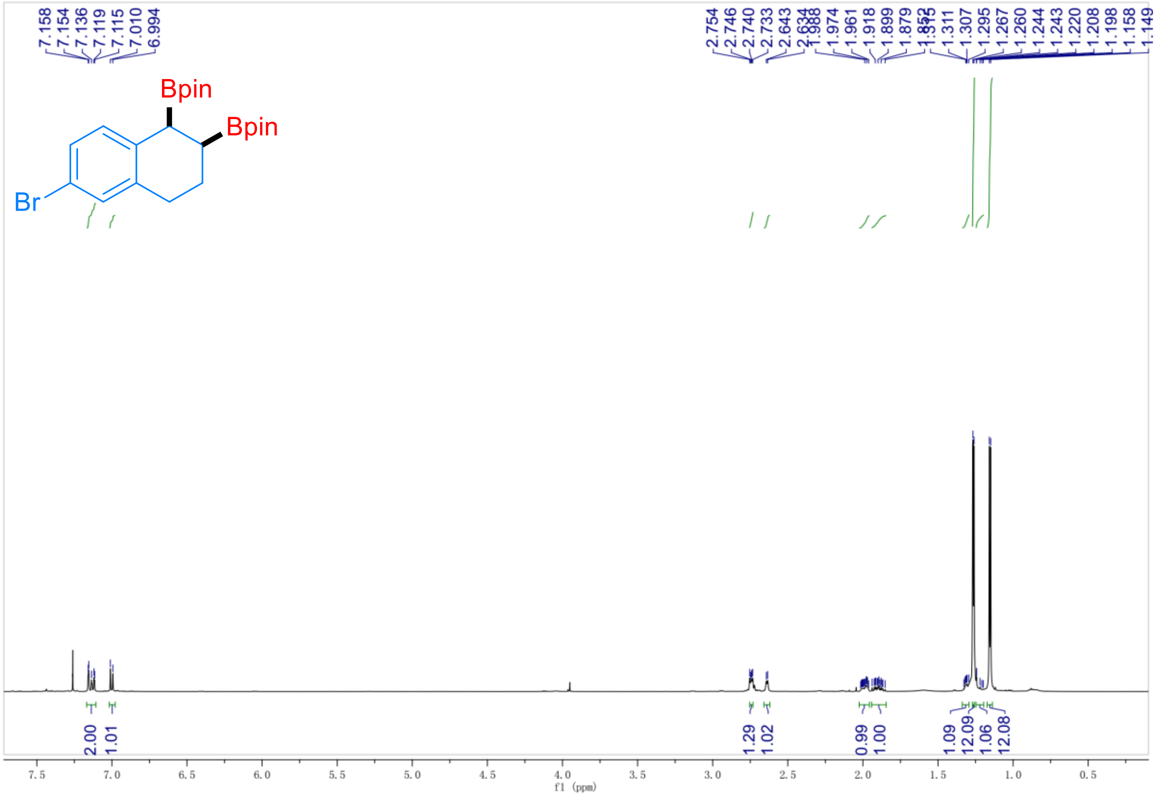

^{1}H NMR spectrum of compound **28b** in $CDCl_3$ (500 MHz).

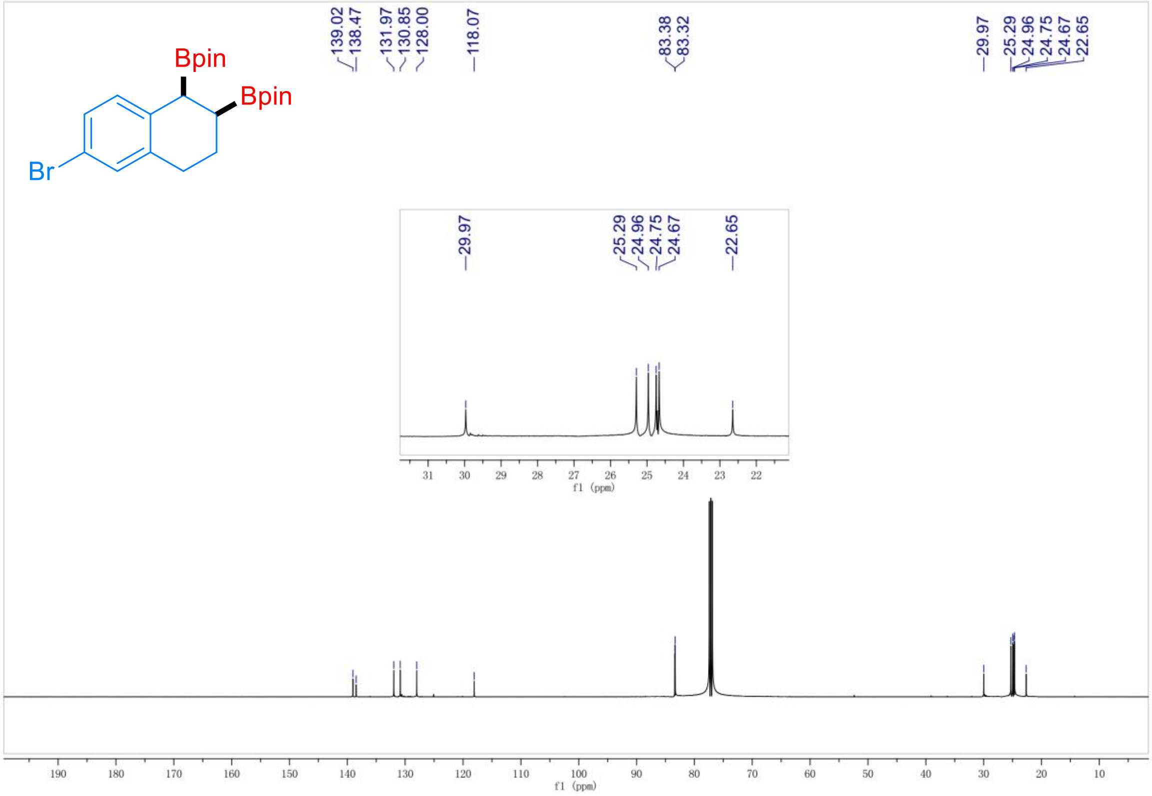

$^{13}C\{^1H\}$ NMR spectrum of compound **28b** in $CDCl_3$ (125 MHz).

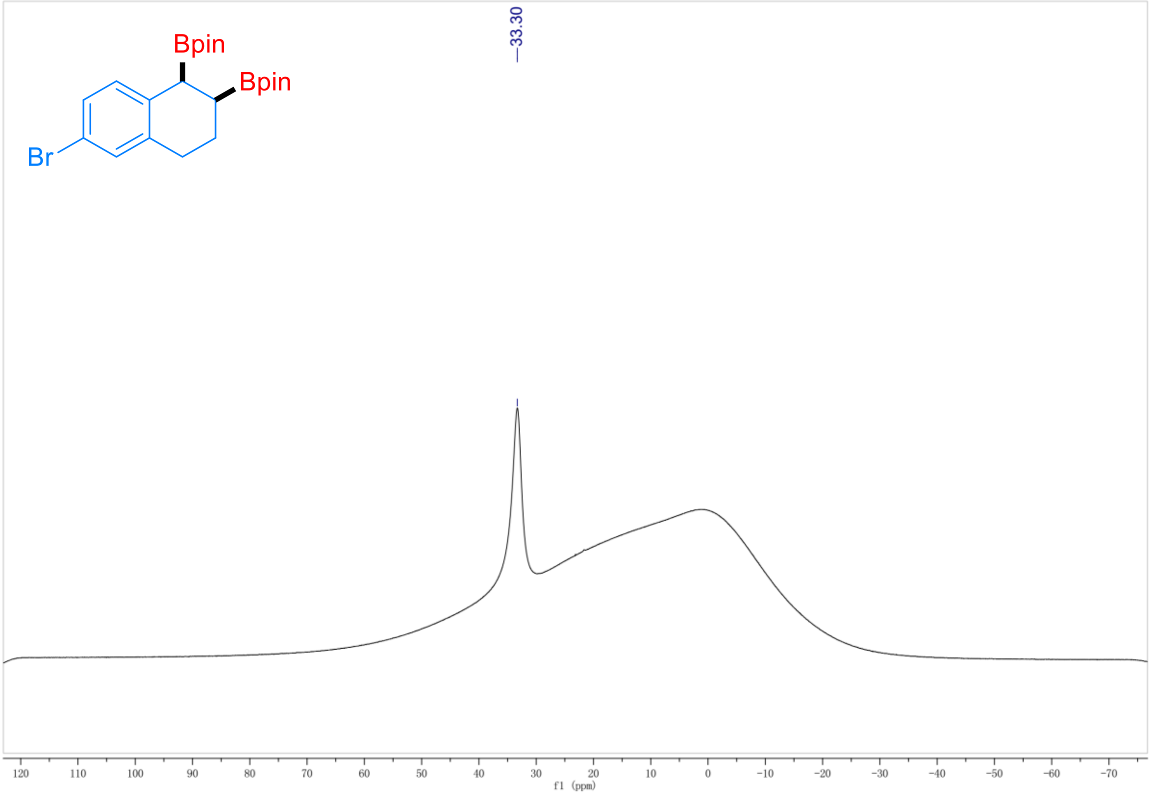

$^{11}B\{^1H\}$ NMR spectrum of compound **28b** in $CDCl_3$ (160 MHz).

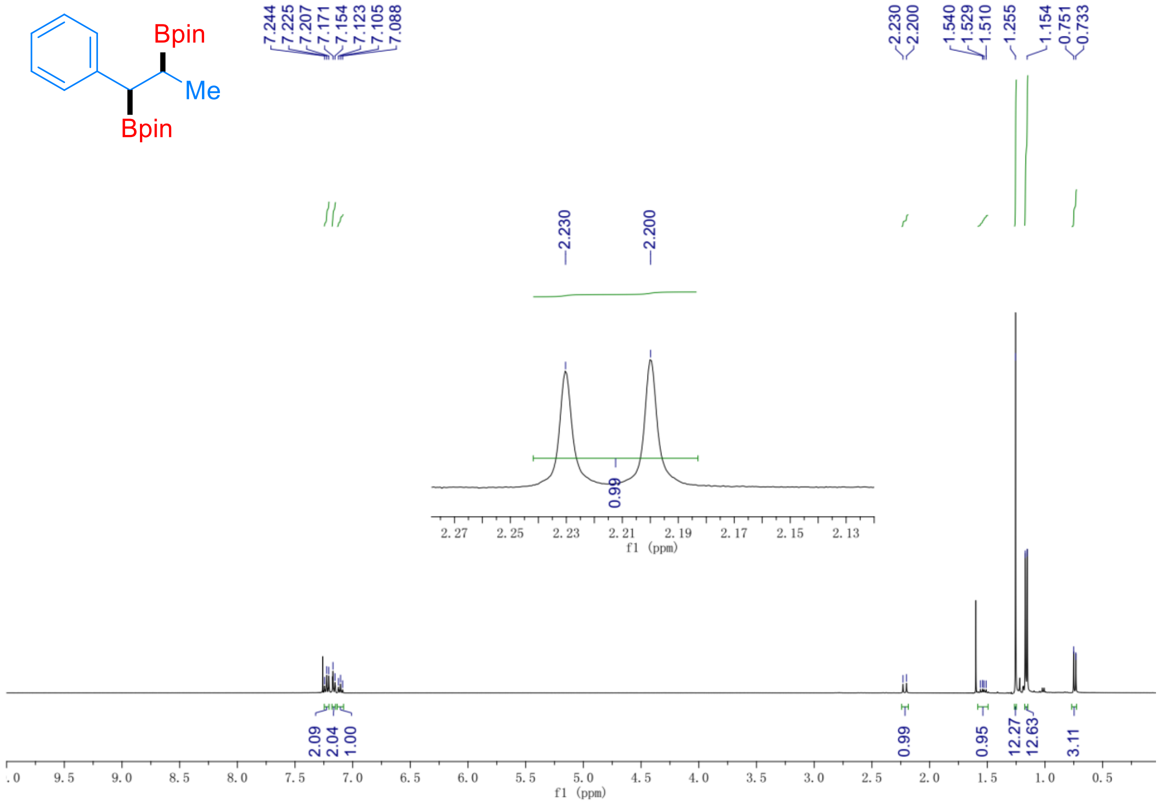

^{1}H NMR spectrum of compound **29b** in $CDCl_3$ (400 MHz).

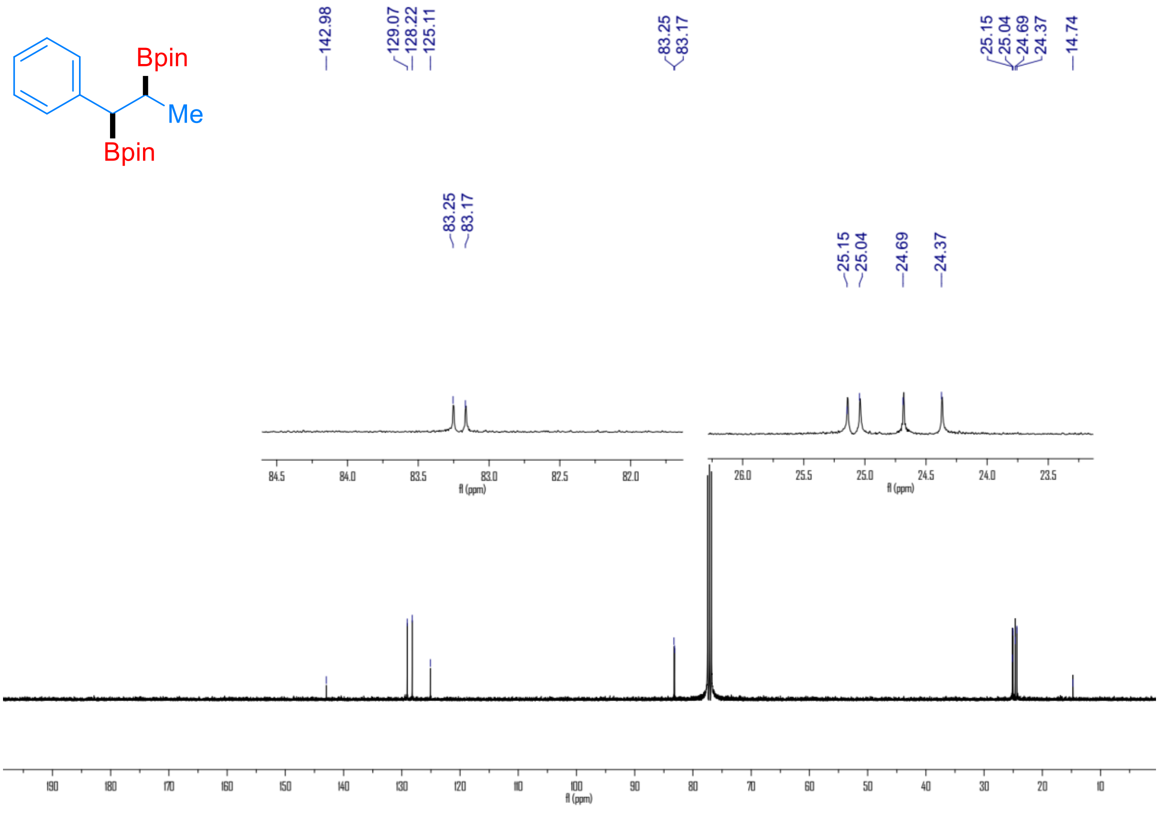

^{13}C{^{1}H} NMR spectrum of compound **29b** in $CDCl_3$ (100 MHz).

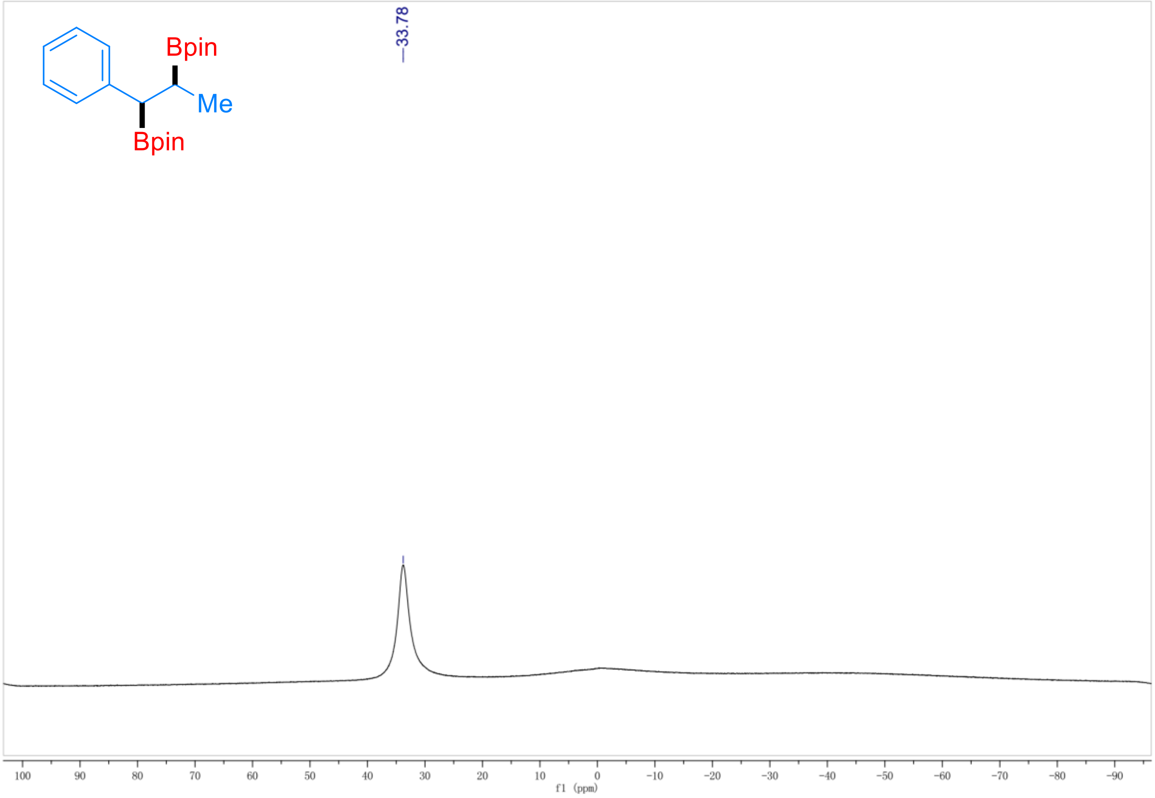

$^{11}B\{^{1}H\}$ NMR spectrum of compound **29b** in $CDCl_3$ (128 MHz).

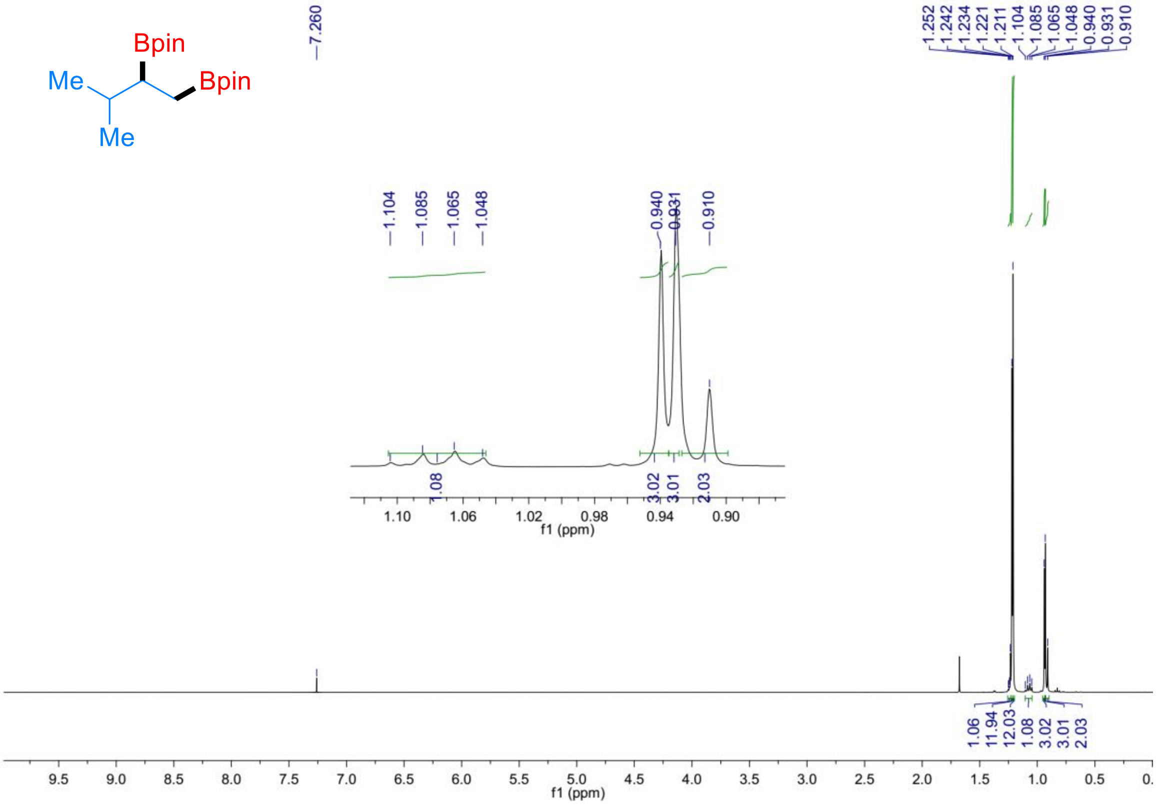

^{1}H NMR spectrum of compound **31b** in $CDCl_3$ (400 MHz).

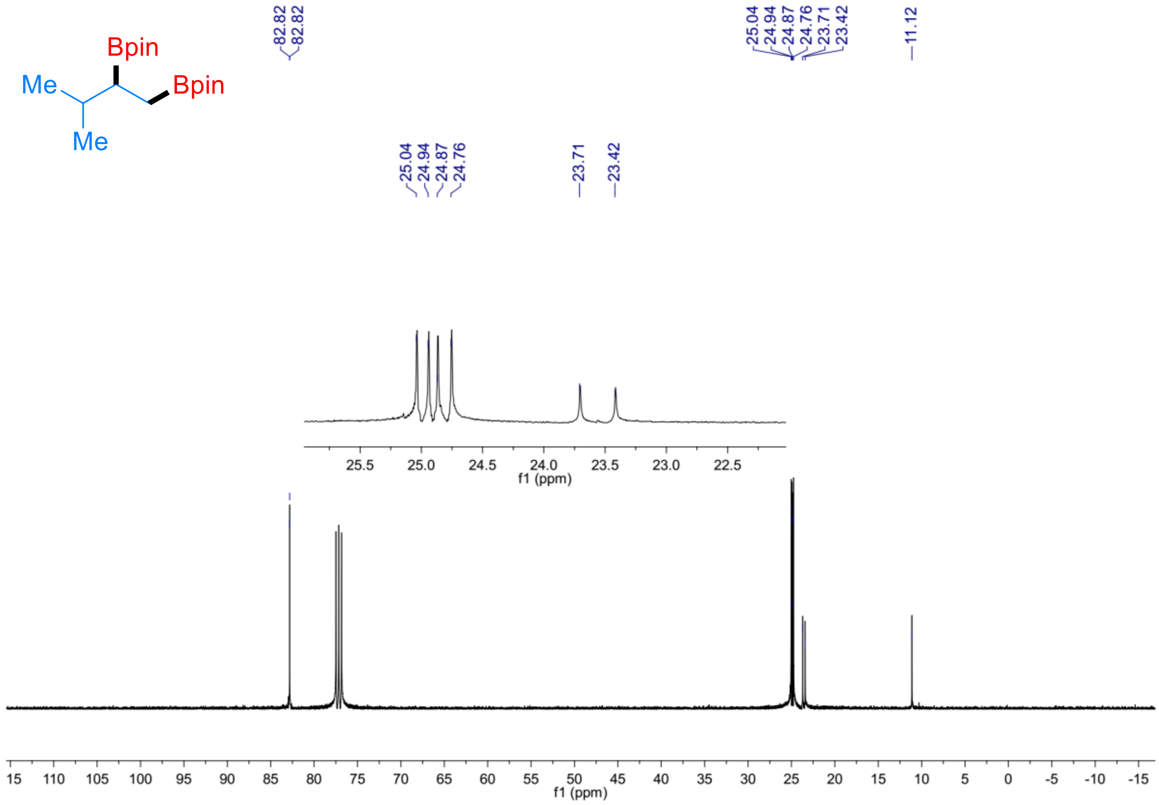

$^{13}C\{^{1}H\}$ NMR spectrum of compound **31b** in $CDCl_3$ (100 MHz).

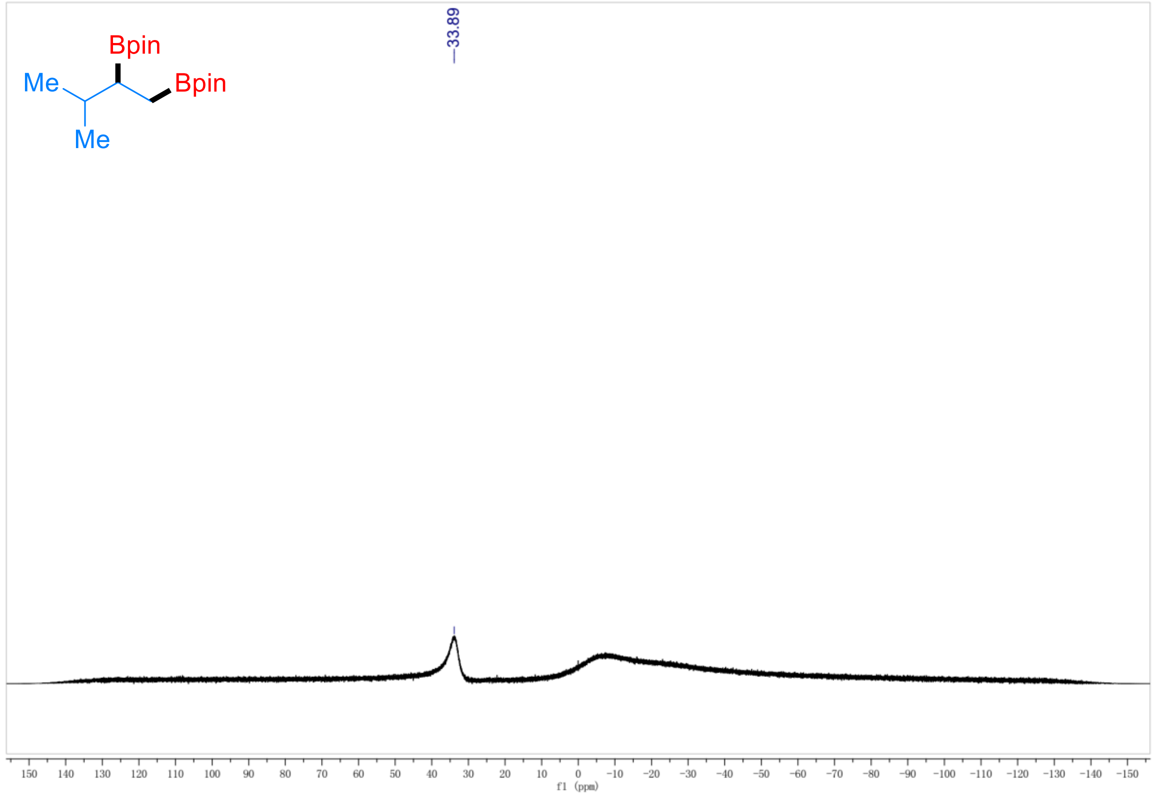

$^{11}B\{^{1}H\}$ NMR spectrum of compound **31b** in $CDCl_3$ (128 MHz).

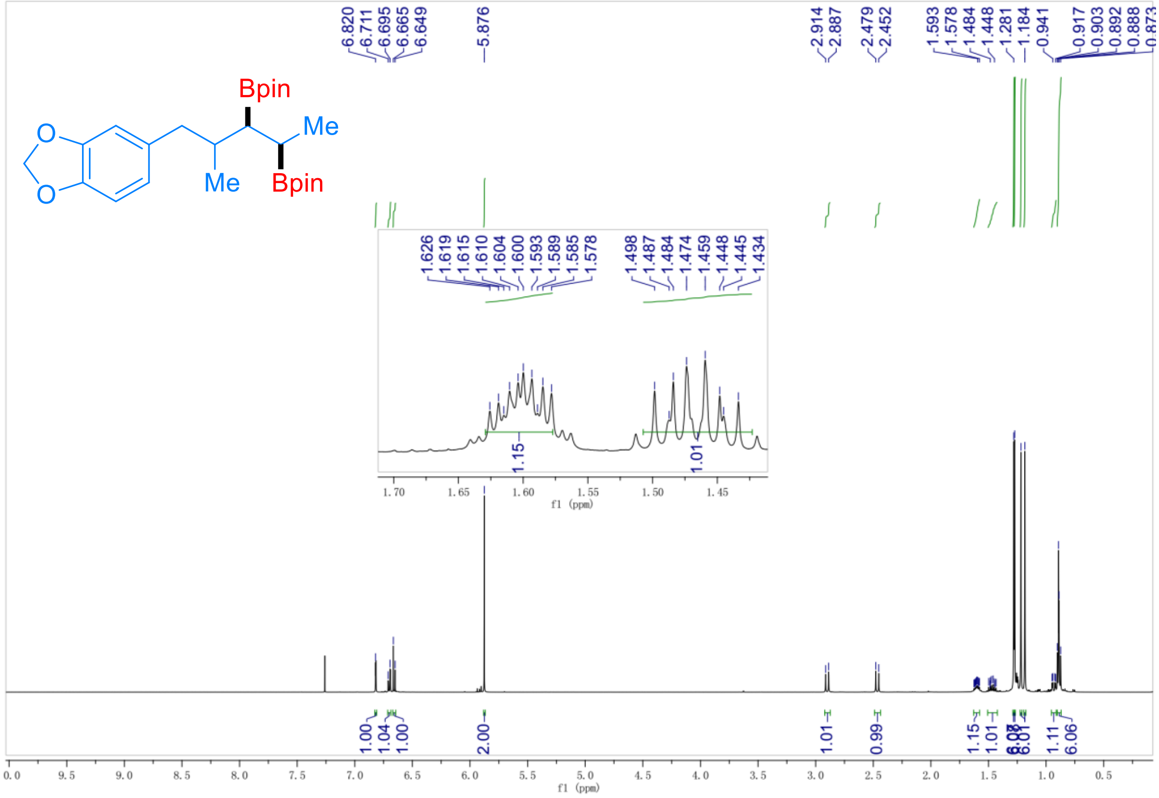

^{1}H NMR spectrum of compound **32b** in $CDCl_3$ (500 MHz).

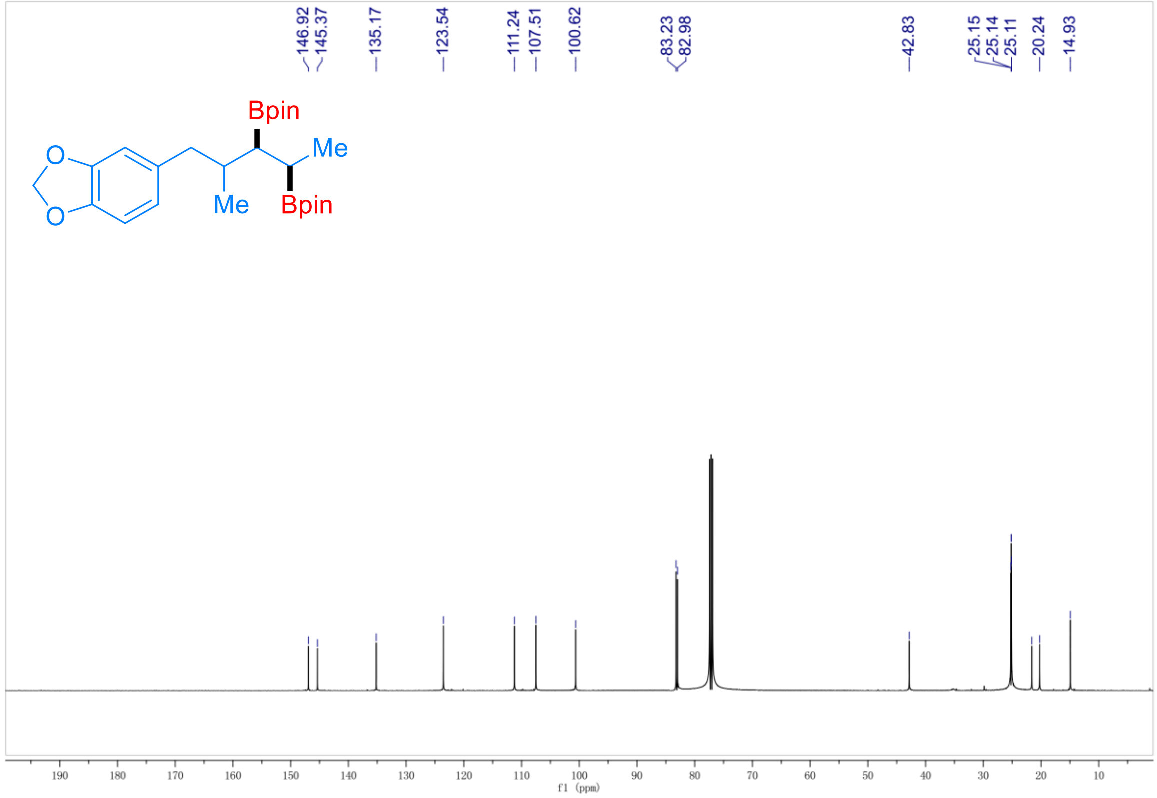

^{13}C{^{1}H} NMR spectrum of compound **32b** in $CDCl_3$ (125 MHz).

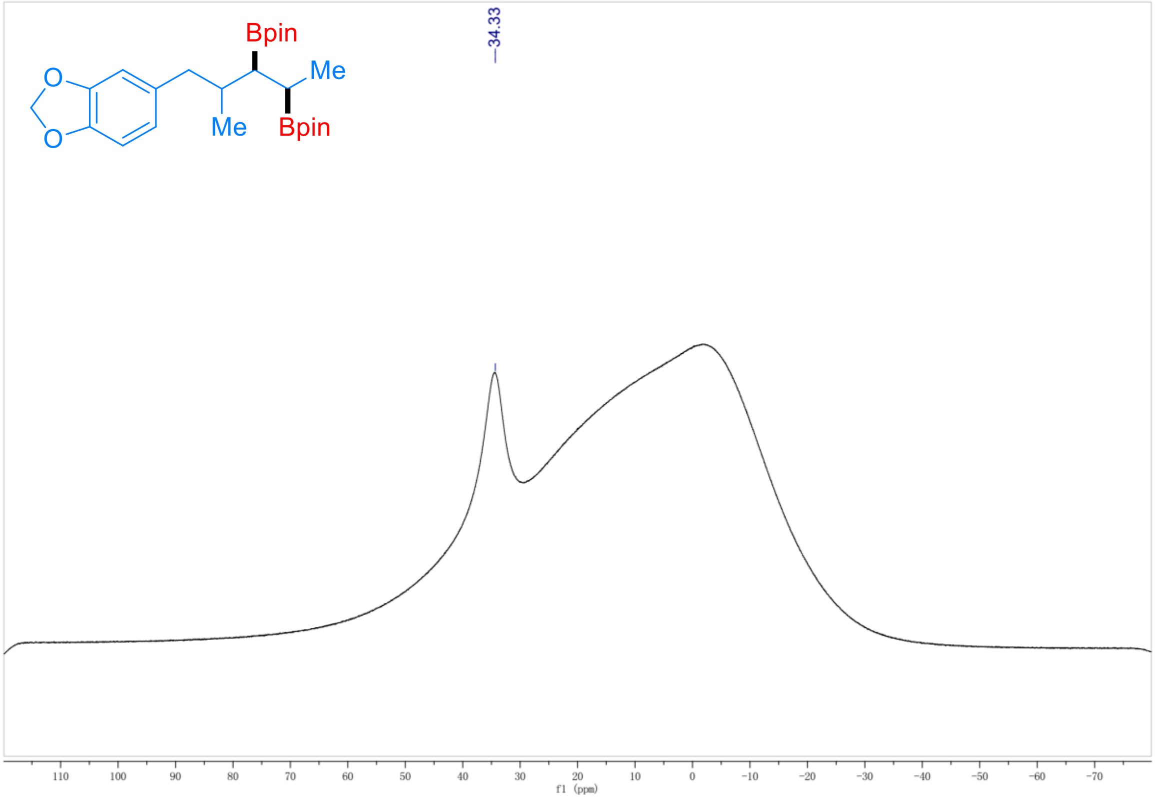

^{11}B{^{1}H} NMR spectrum of compound **32b** in $CDCl_3$ (160 MHz).

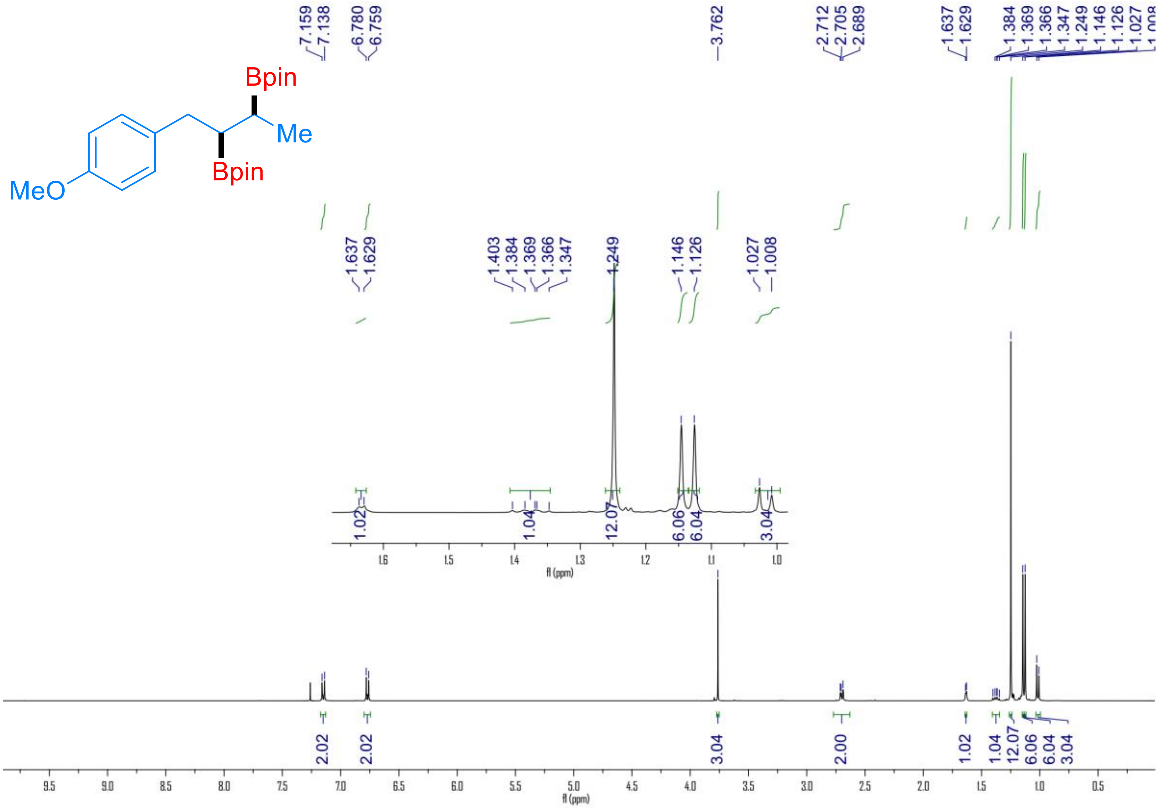

^{1}H NMR spectrum of compound **33b** in $CDCl_3$ (400 MHz).

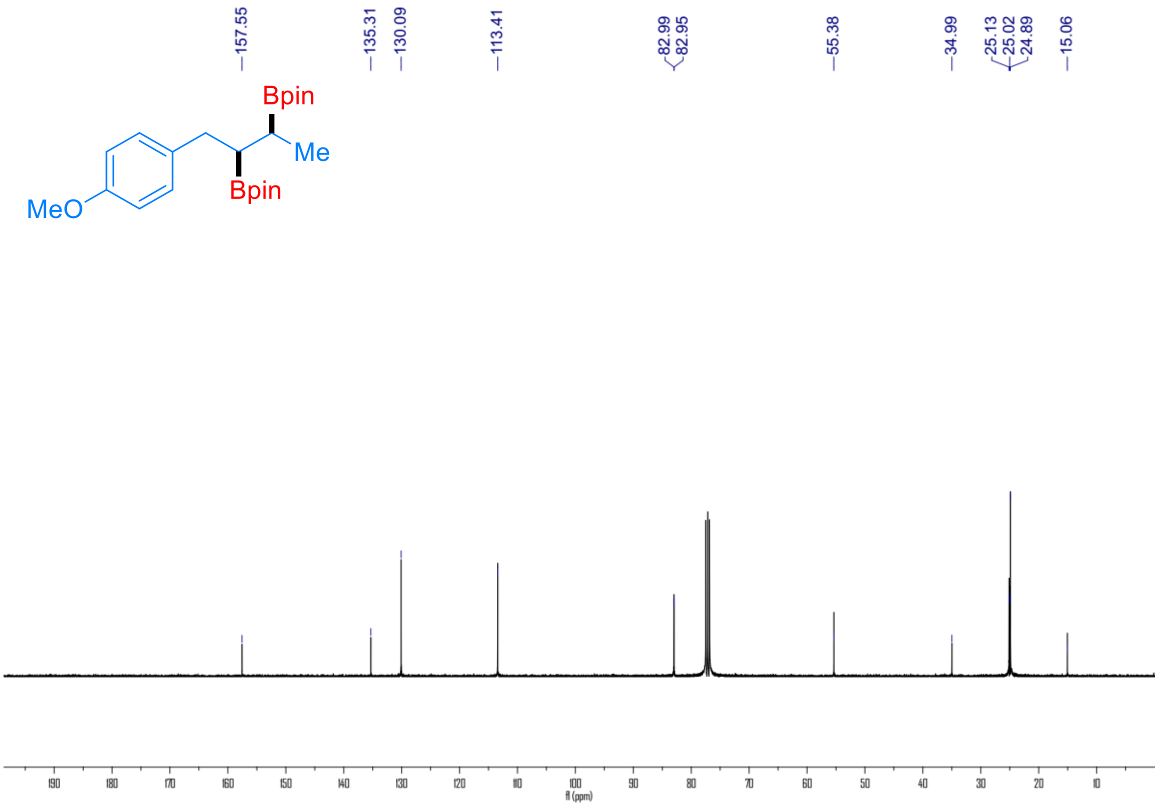

$^{13}C\{^1H\}$ NMR spectrum of compound **33b** in $CDCl_3$ (100 MHz).

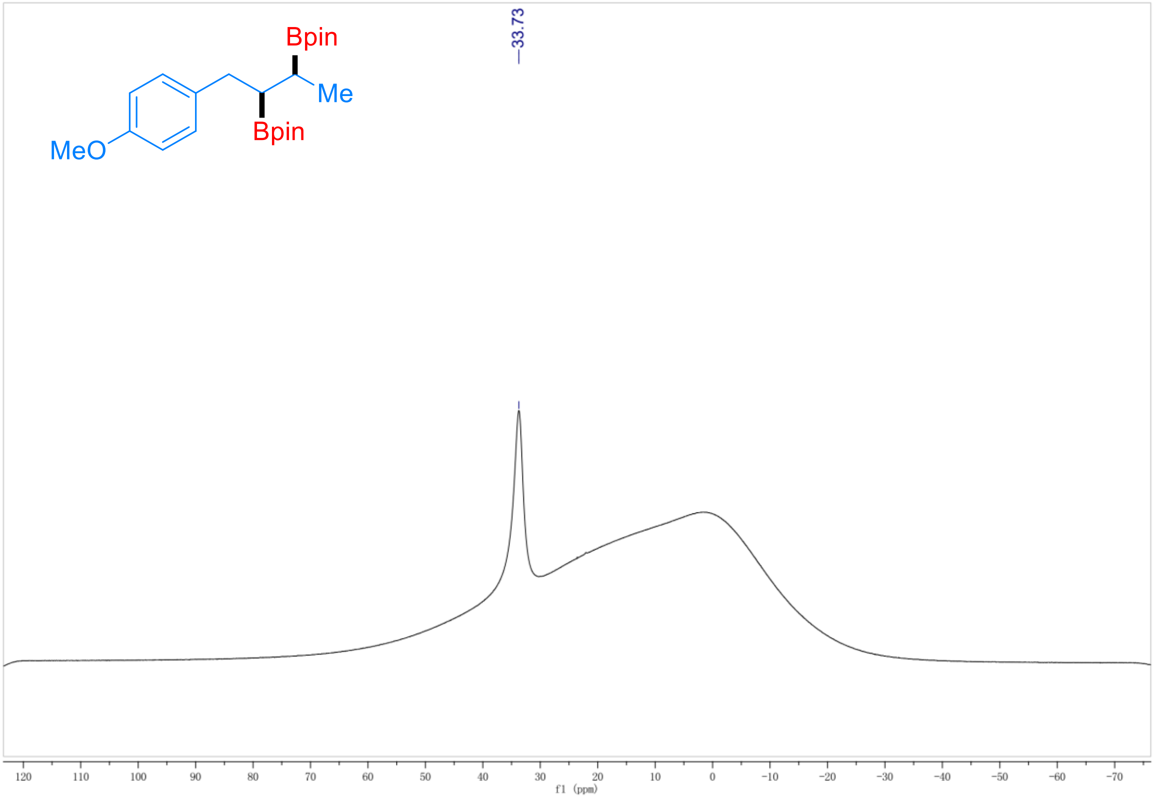

$^{11}B\{^1H\}$ NMR spectrum of compound **33b** in $CDCl_3$ (128 MHz).

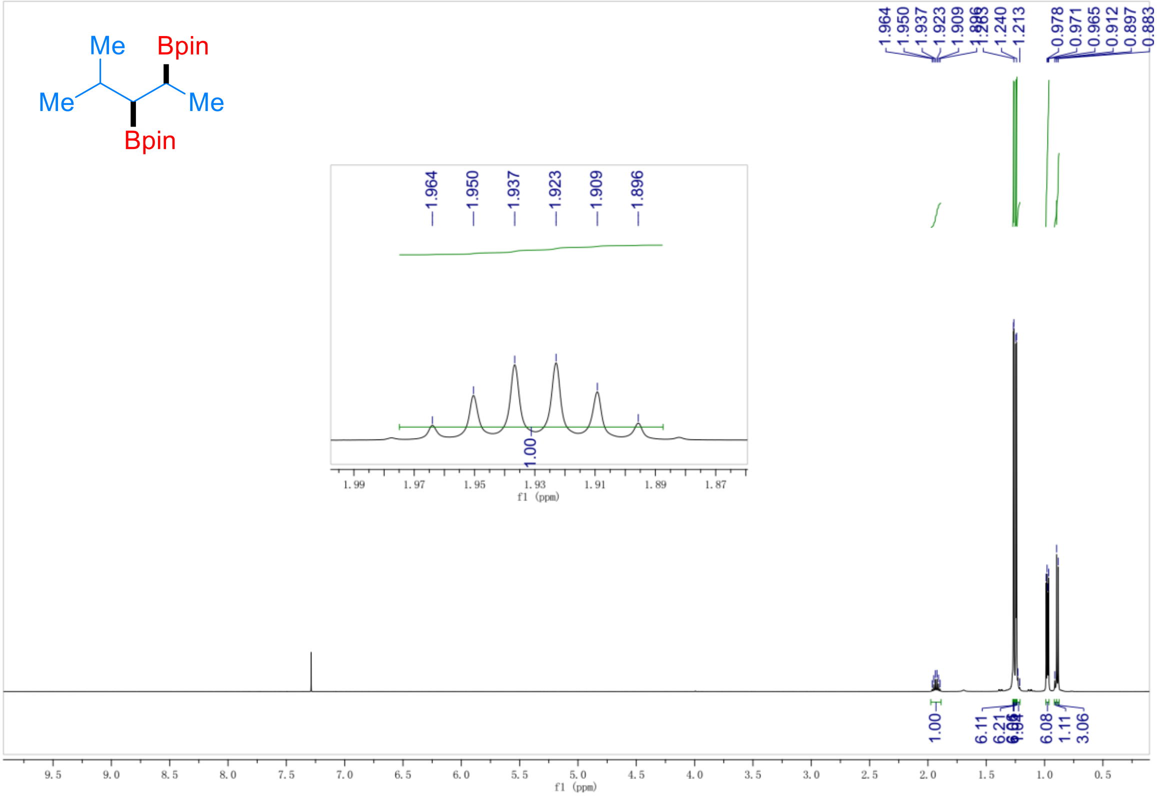

^{1}H NMR spectrum of compound **34b** in $CDCl_3$ (400 MHz).

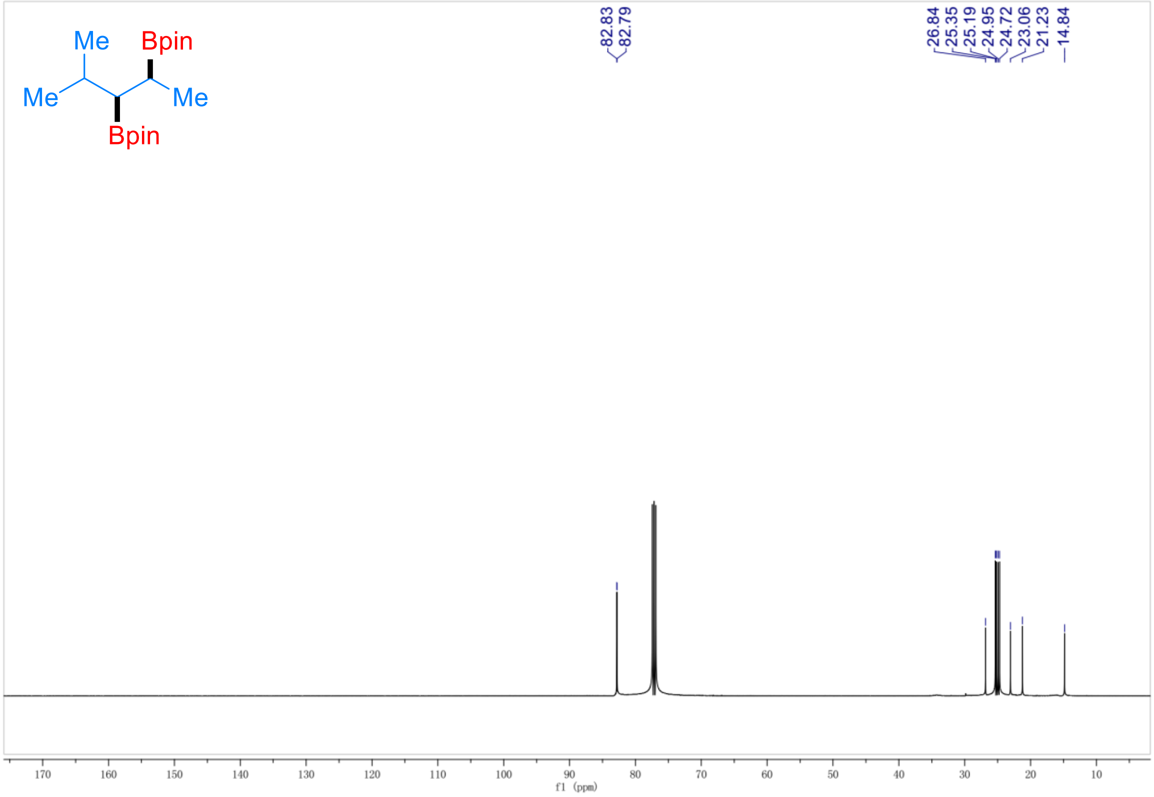

^{13}C{^{1}H} NMR spectrum of compound **34b** in $CDCl_3$ (100 MHz).

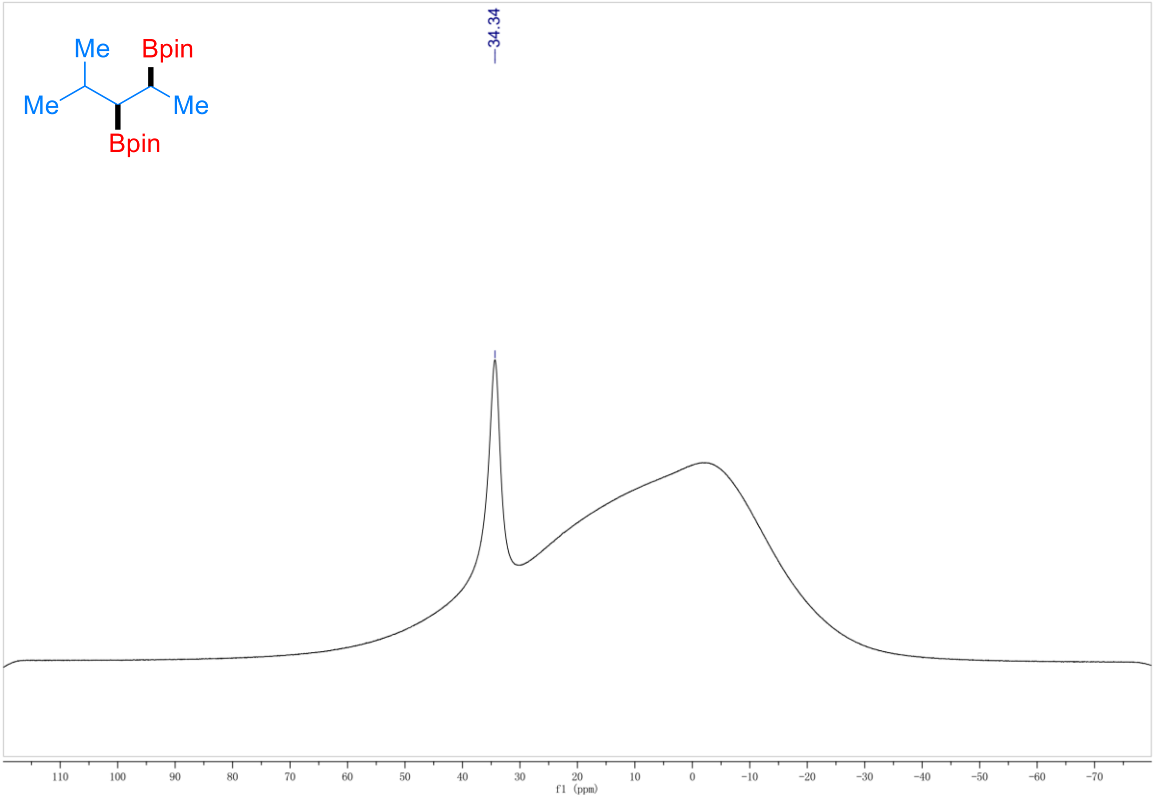

$^{11}B\{^{1}H\}$ NMR spectrum of compound **34b** in $CDCl_3$ (128 MHz).

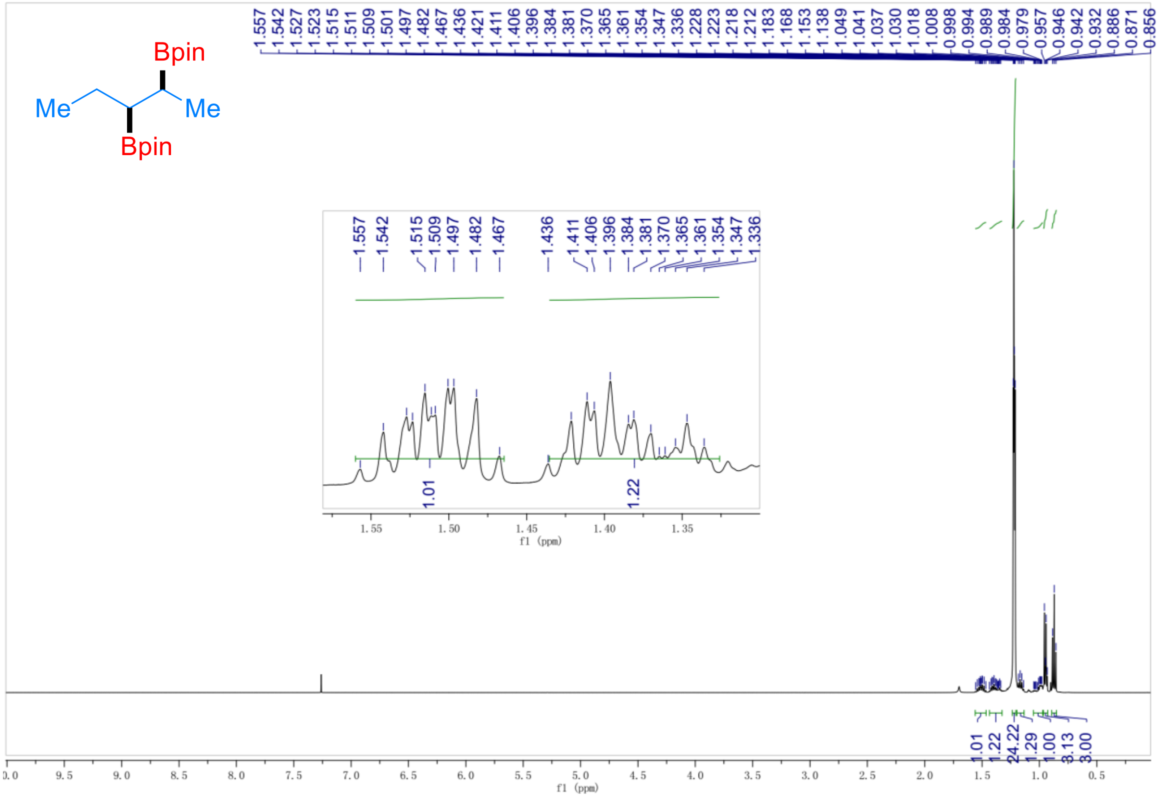

^{1}H NMR spectrum of compound **35b** in $CDCl_3$ (500 MHz).

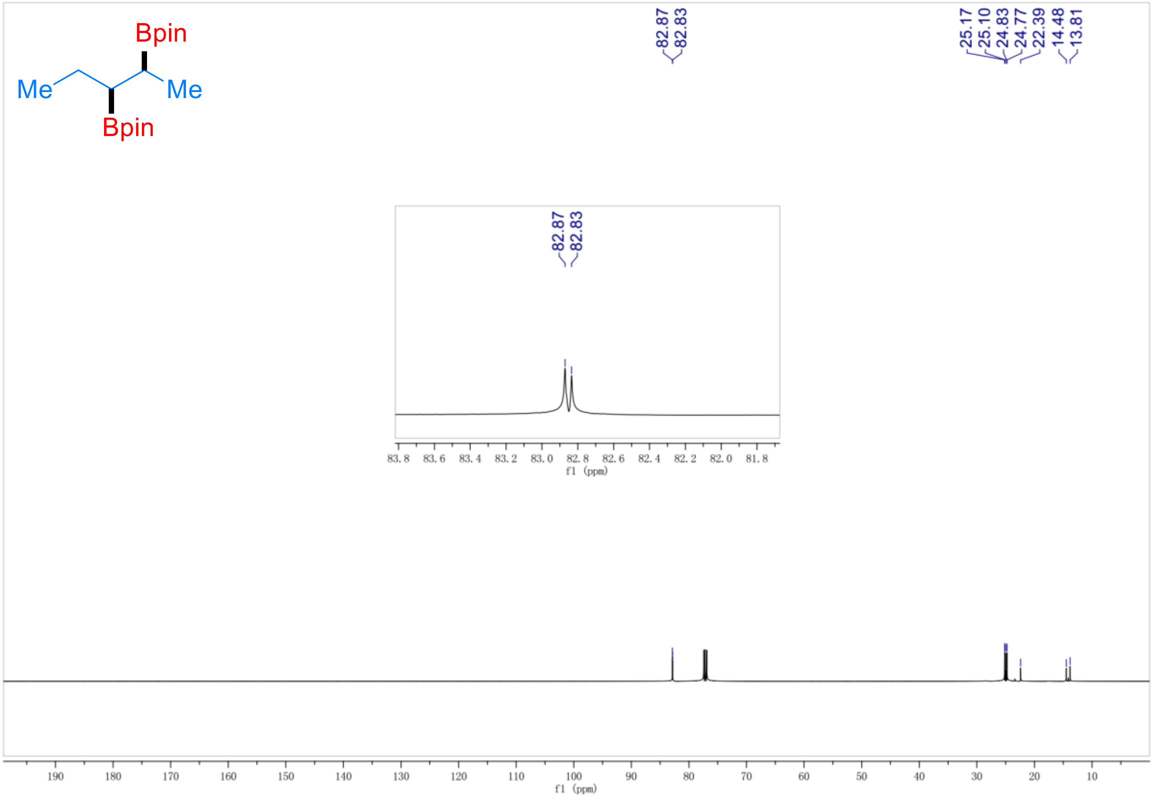

$^{13}C\{^{1}H\}$ NMR spectrum of compound **35b** in $CDCl_3$ (125 MHz).

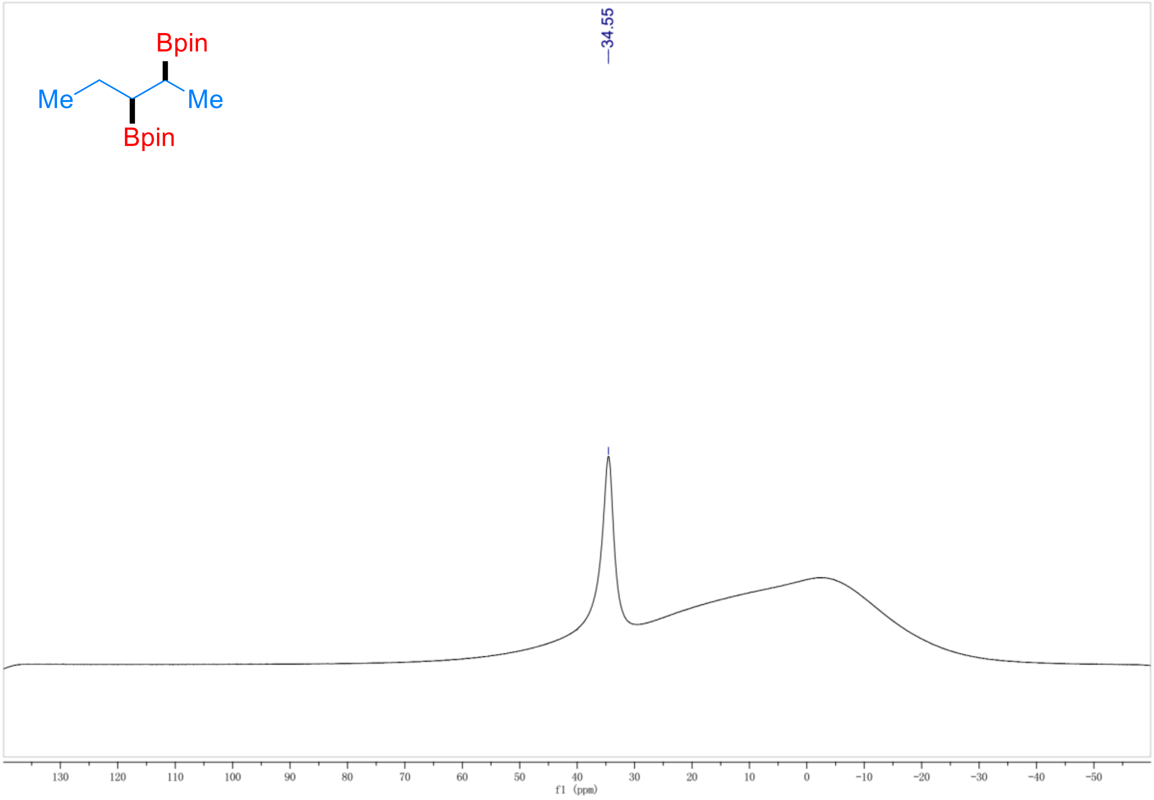

$^{11}B\{^{1}H\}$ NMR spectrum of compound **35b** in $CDCl_3$ (160 MHz).

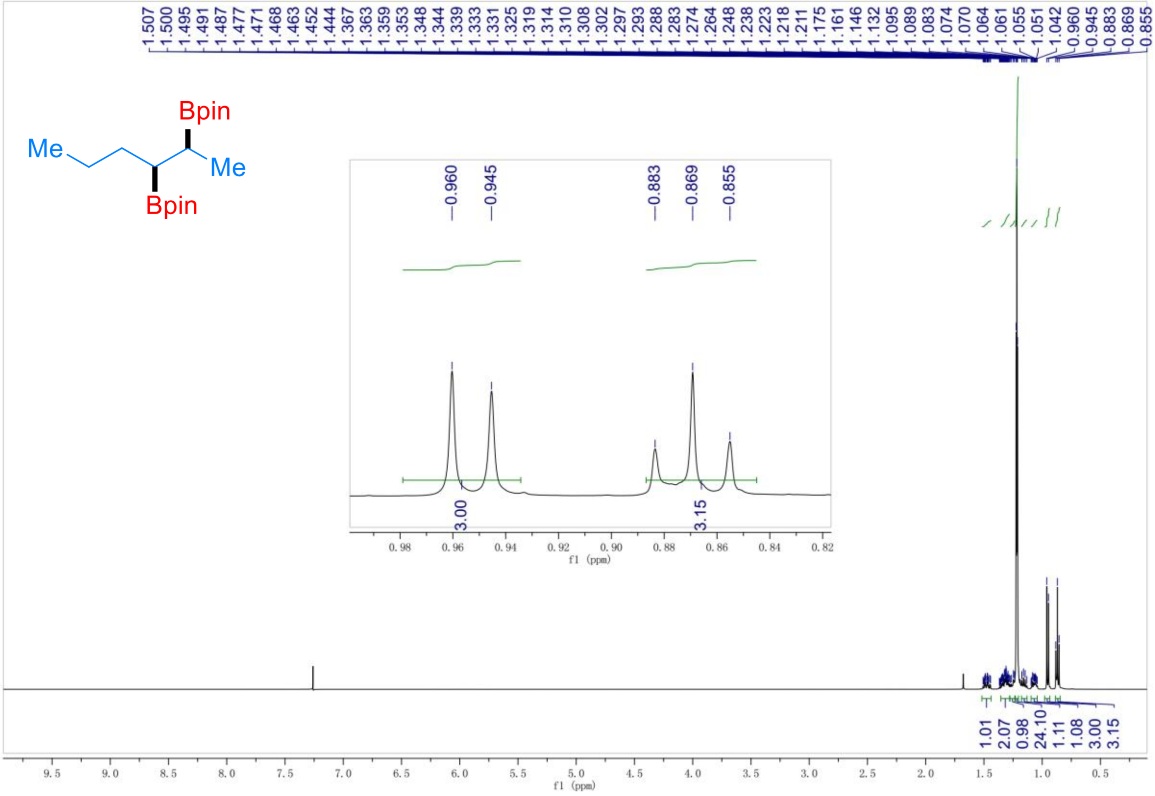

^{1}H NMR spectrum of compound **36b** in $CDCl_3$ (500 MHz).

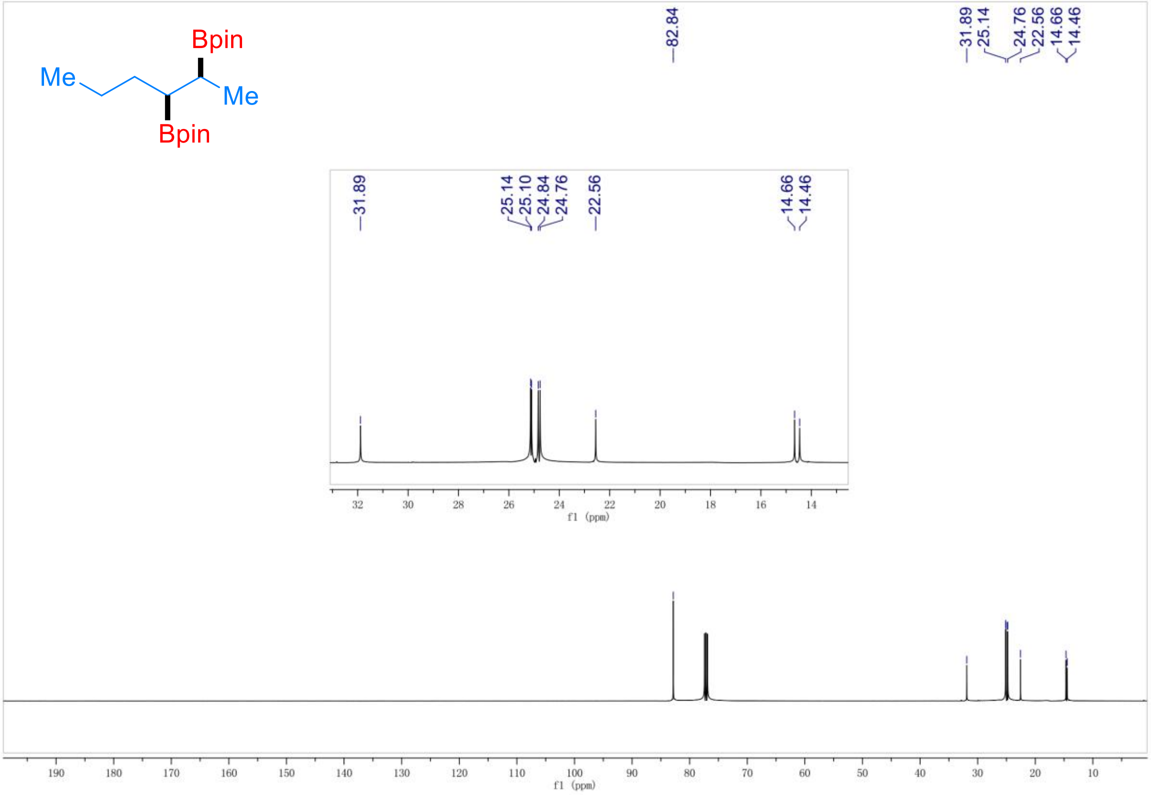

^{13}C{^{1}H} NMR spectrum of compound **36b** in $CDCl_3$ (125 MHz).

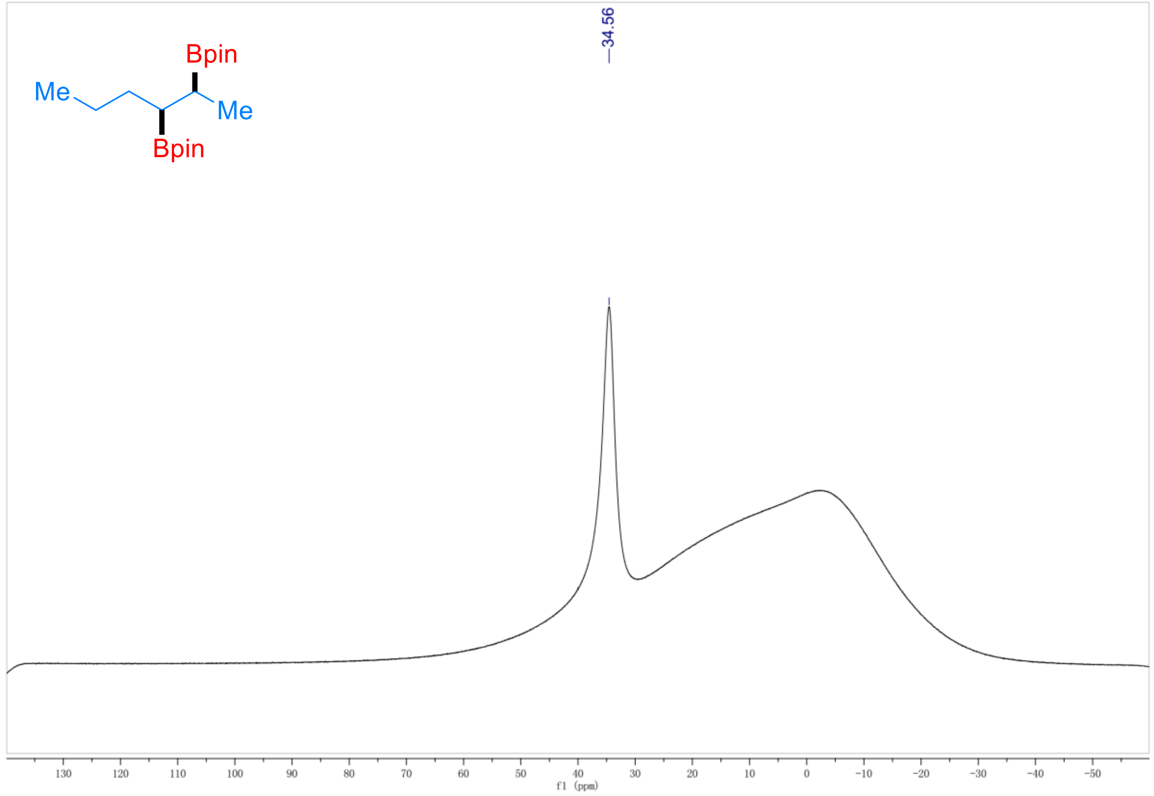

^{11}B{^{1}H} NMR spectrum of compound **36b** in $CDCl_3$ (160 MHz).

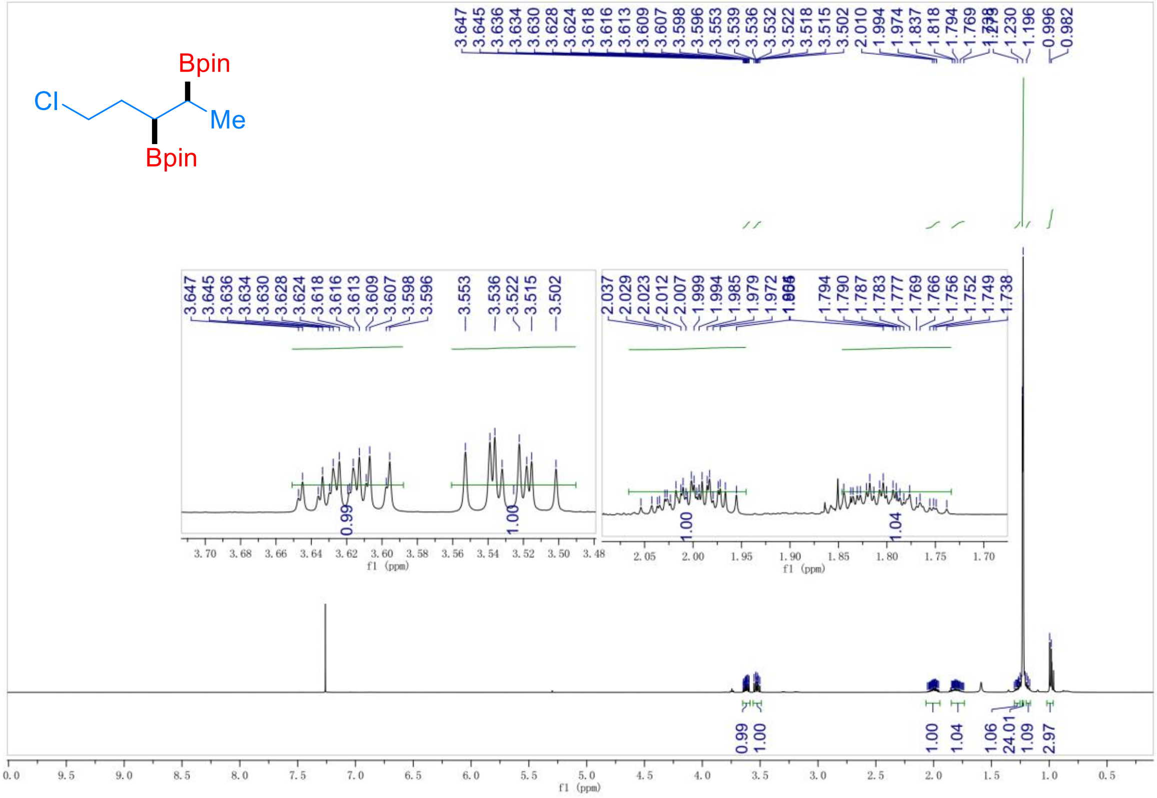

^{1}H NMR spectrum of compound **37b** in $CDCl_3$ (500 MHz).

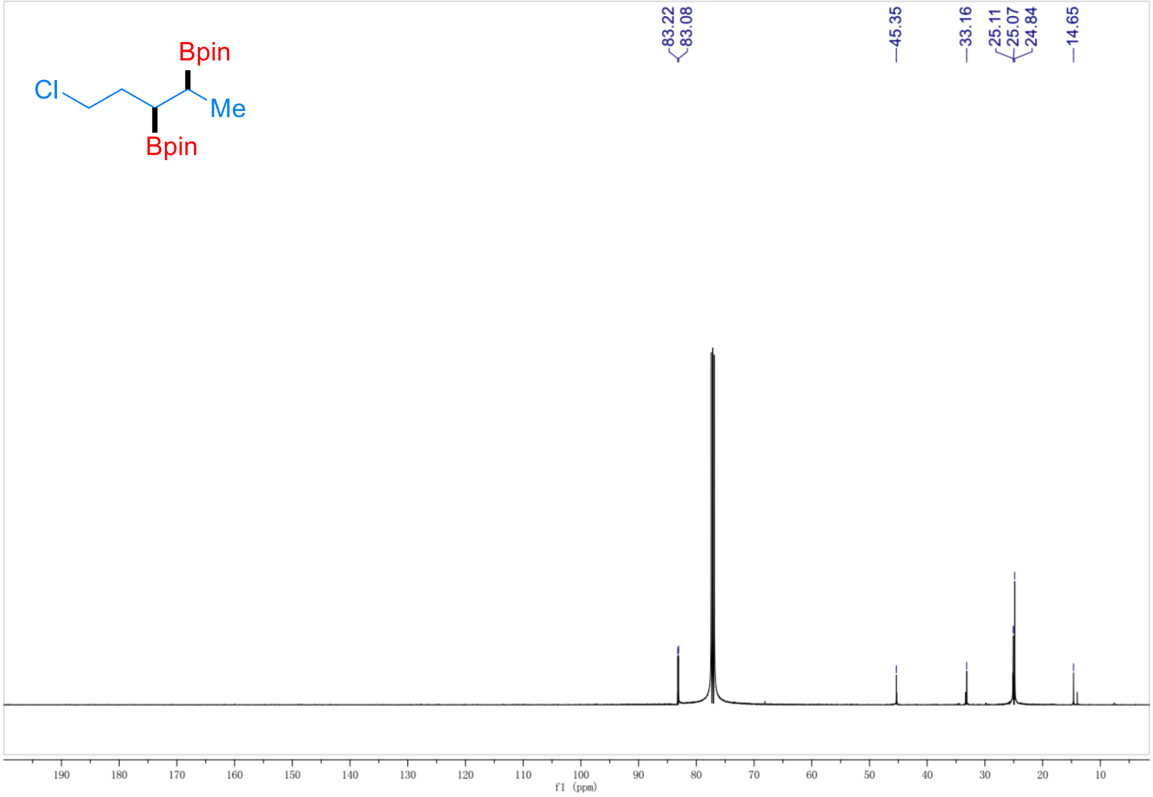

$^{13}C\{^{1}H\}$ NMR spectrum of compound **37b** in $CDCl_3$ (125 MHz).

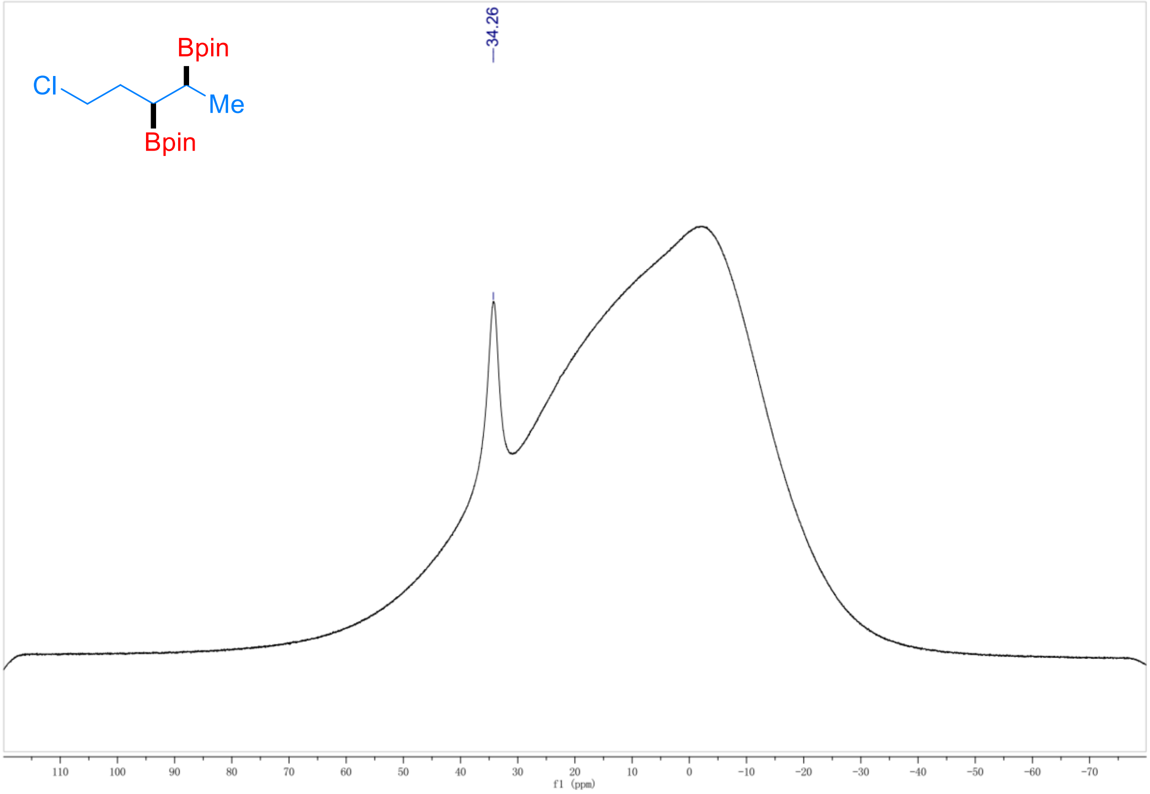

$^{11}B\{^{1}H\}$ NMR spectrum of compound **37b** in $CDCl_3$ (160 MHz).

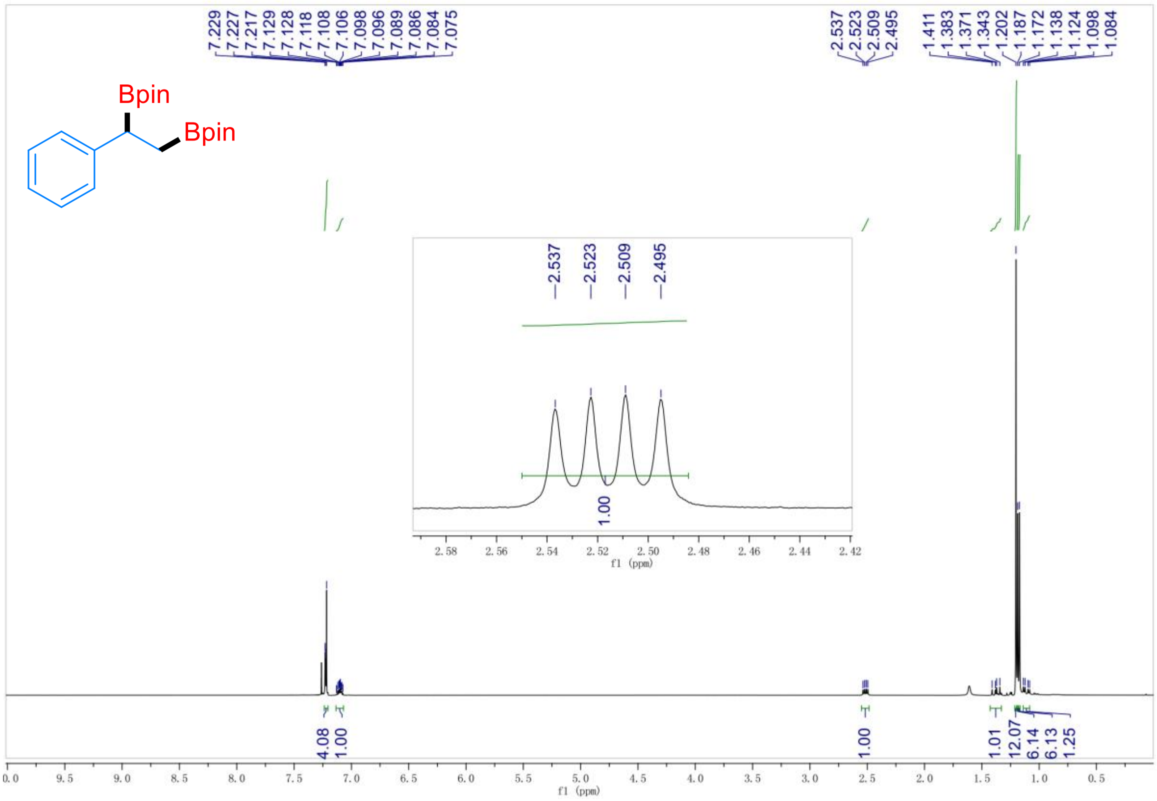

^{1}H NMR spectrum of compound **38b** in $CDCl_3$ (400 MHz).

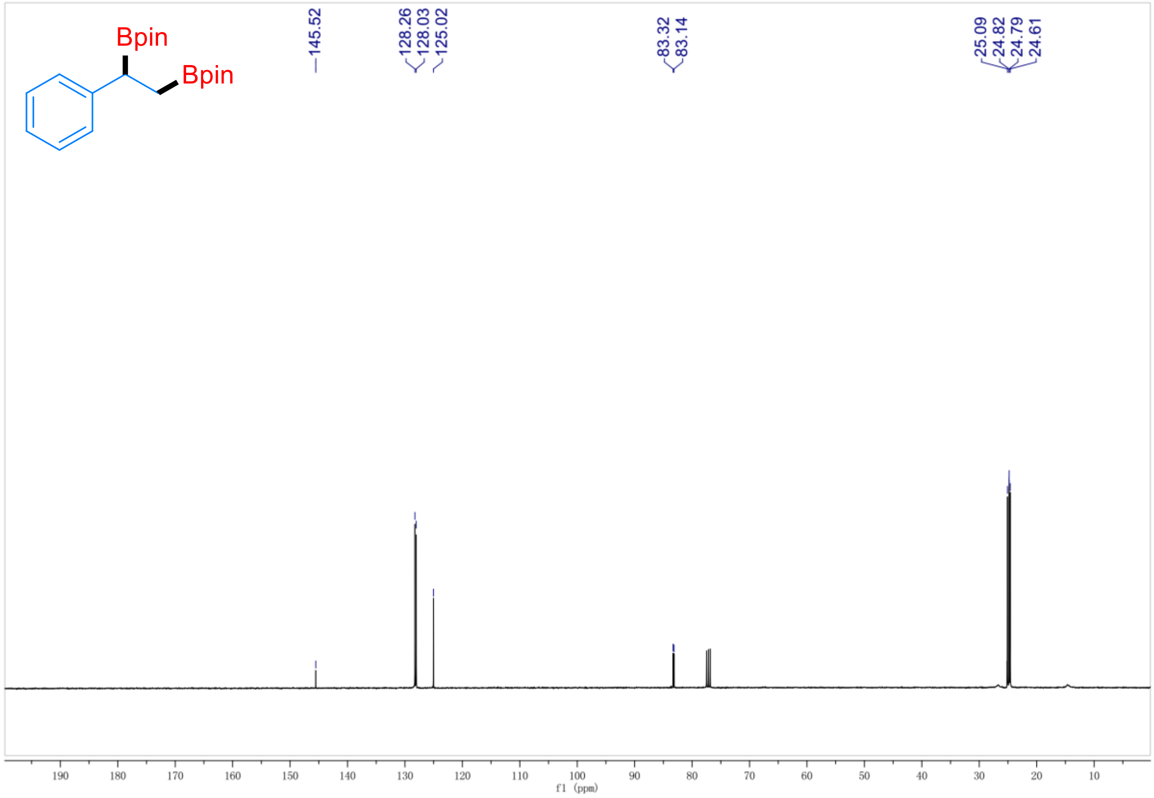

^{13}C{^{1}H} NMR spectrum of compound **38b** in $CDCl_3$ (100 MHz).

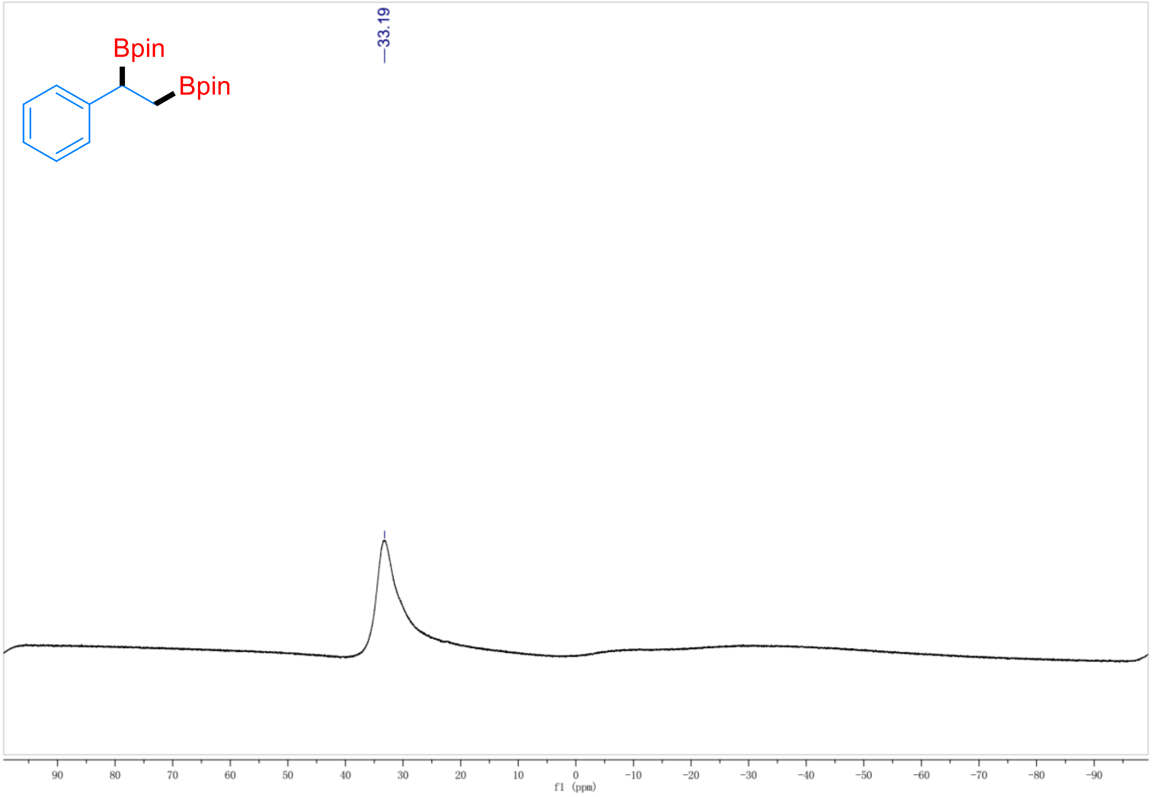

$^{11}B\{^{1}H\}$ NMR spectrum of compound **38b** in $CDCl_3$ (128 MHz).

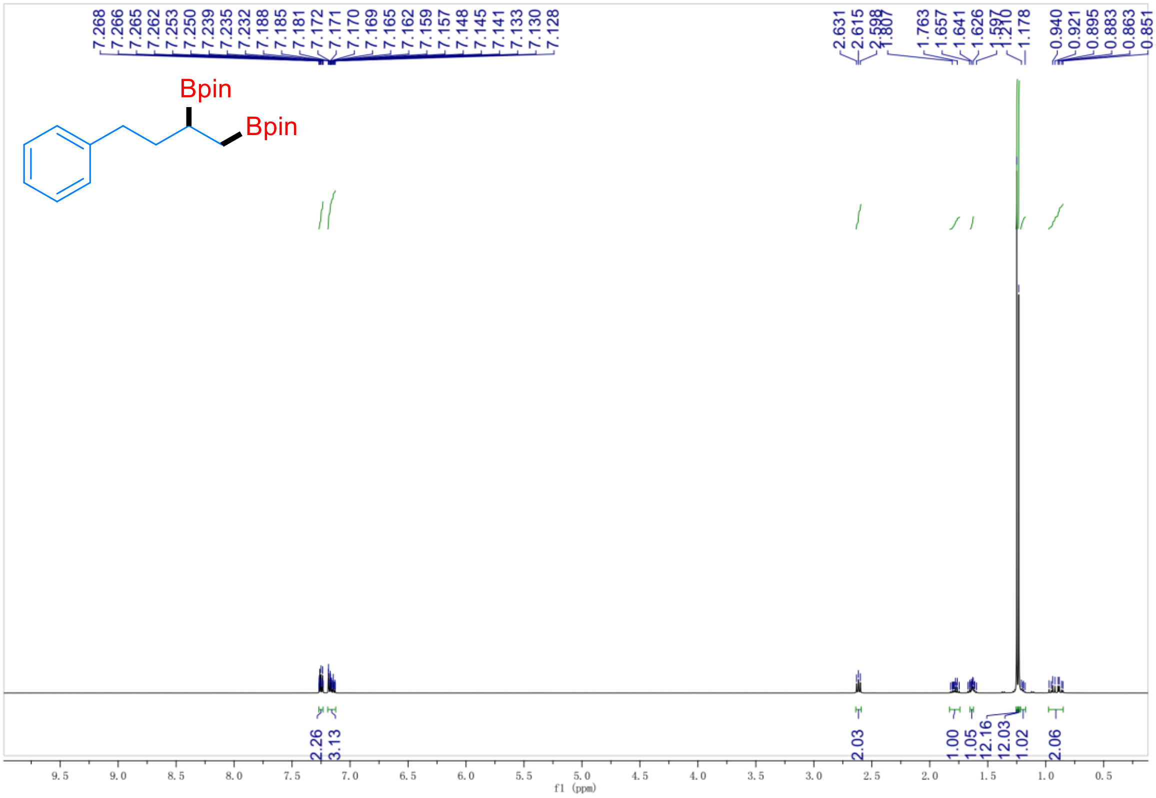

^{1}H NMR spectrum of compound **39b** in $CDCl_3$ (500 MHz).

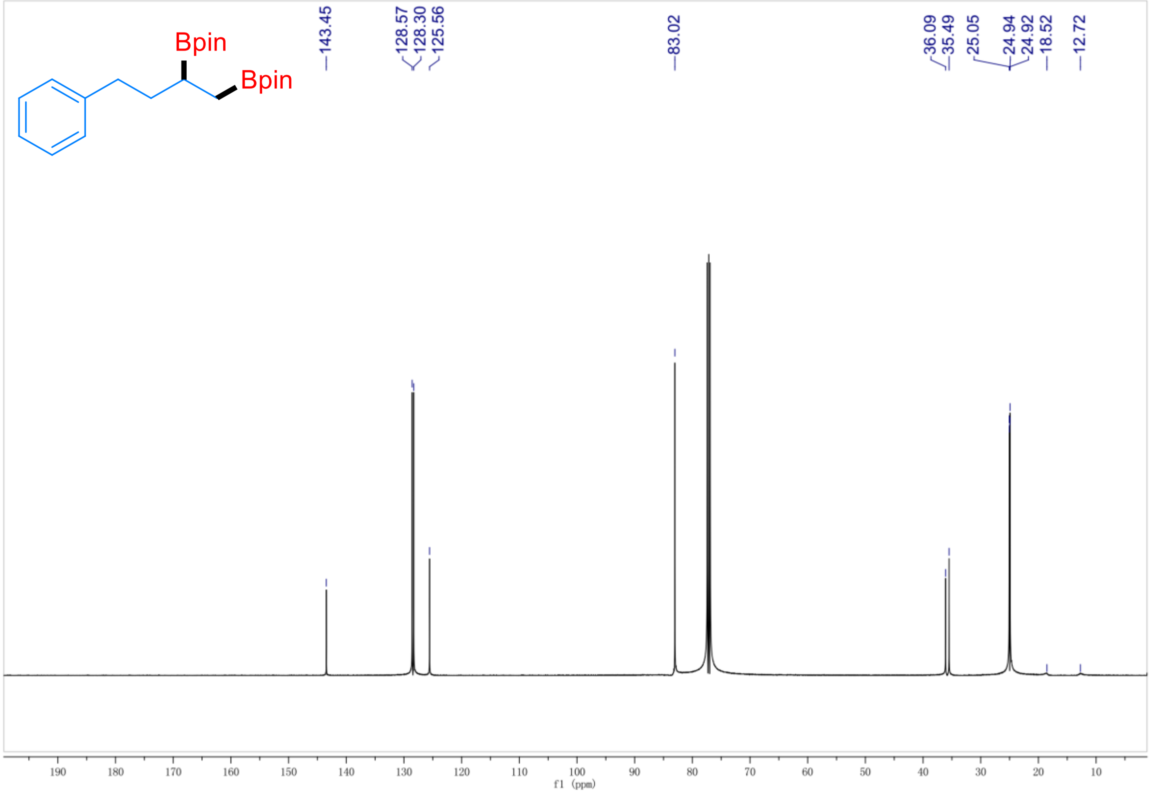

$^{13}C\{^1H\}$ NMR spectrum of compound **39b** in $CDCl_3$ (125 MHz).

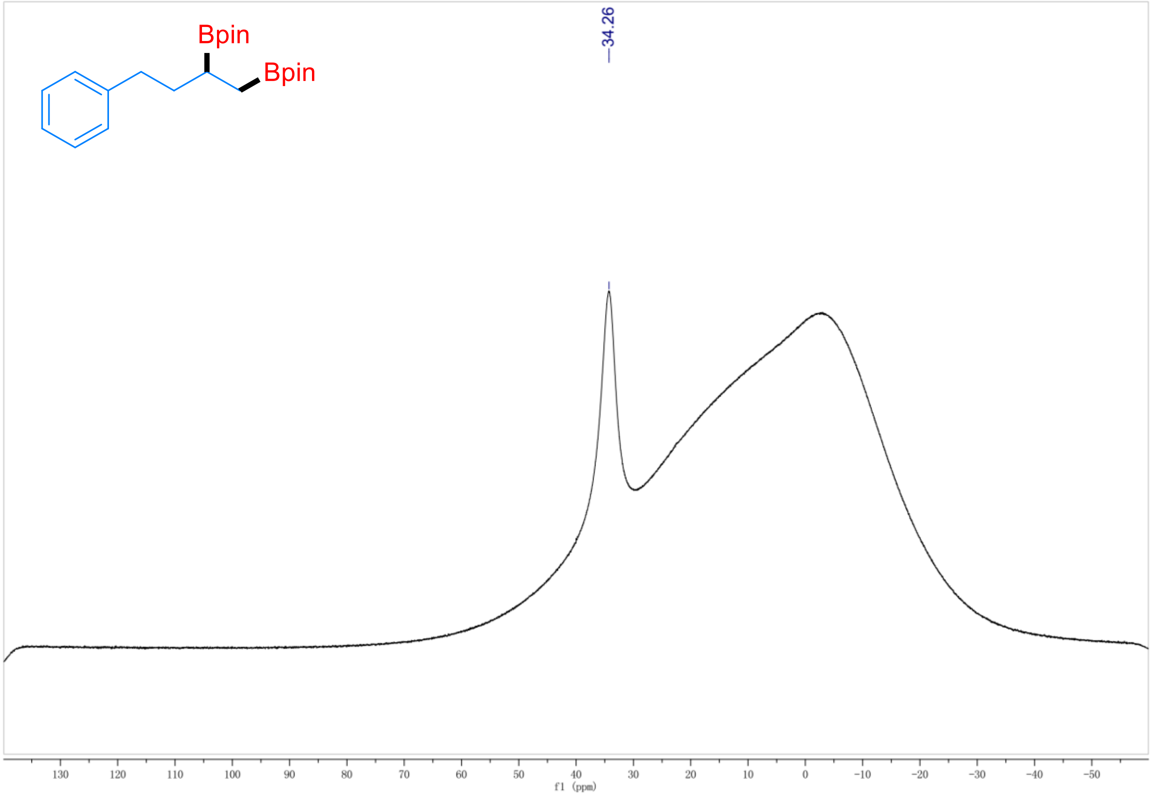

$^{11}B\{^1H\}$ NMR spectrum of compound **39b** in $CDCl_3$ (160 MHz).

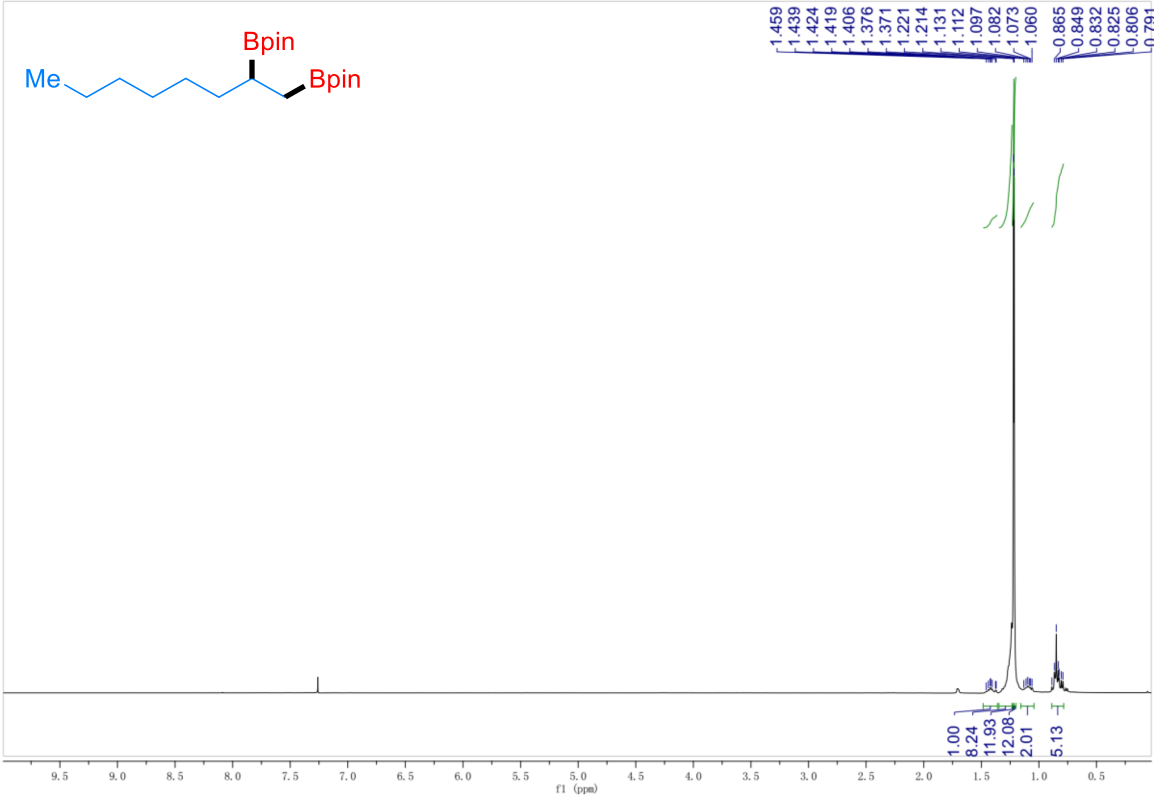

^{1}H NMR spectrum of compound **40b** in $CDCl_3$ (400 MHz).

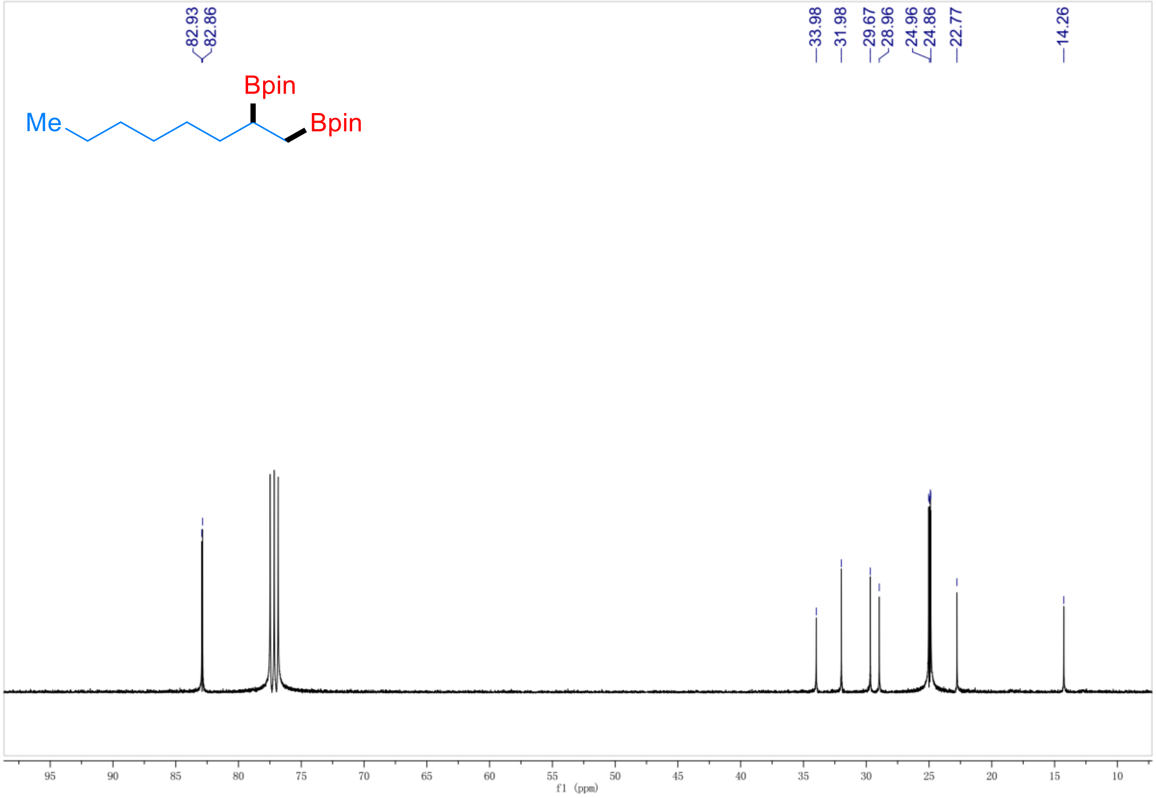

^{13}C{^{1}H} NMR spectrum of compound **40b** in $CDCl_3$ (100 MHz).

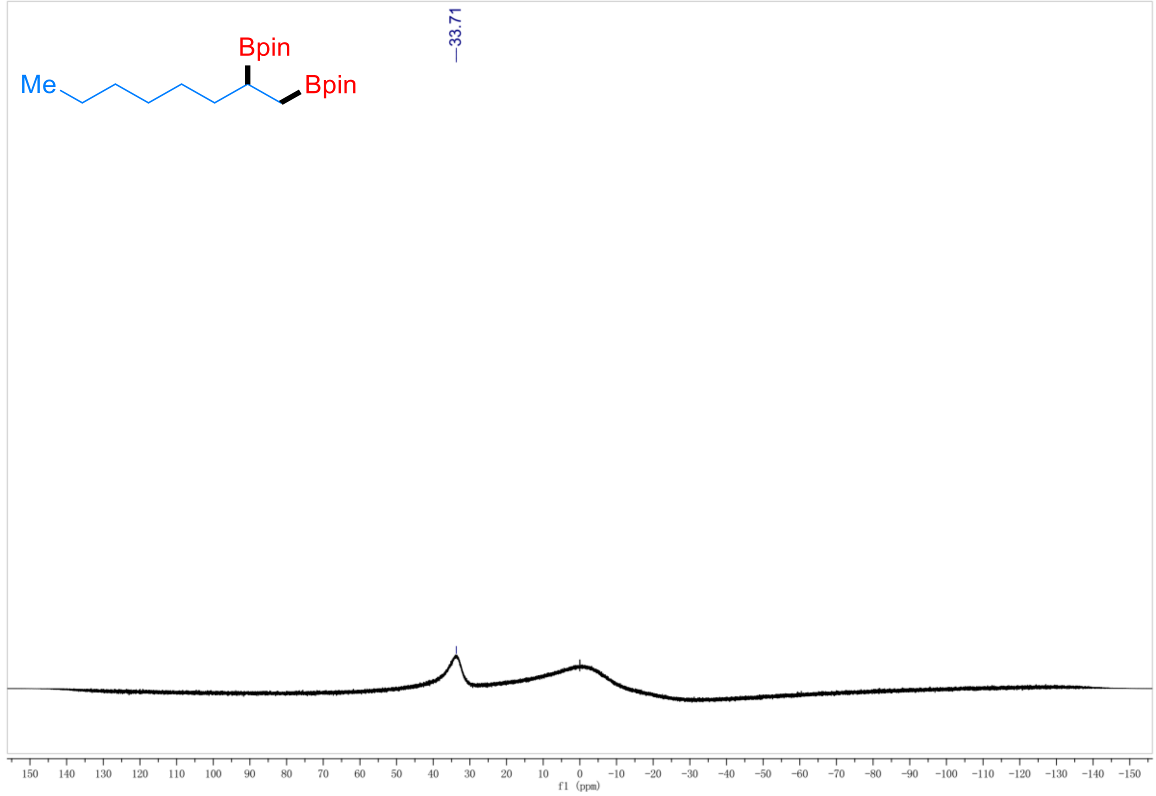

$^{11}B\{^1H\}$ NMR spectrum of compound **40b** in $CDCl_3$ (128 MHz).

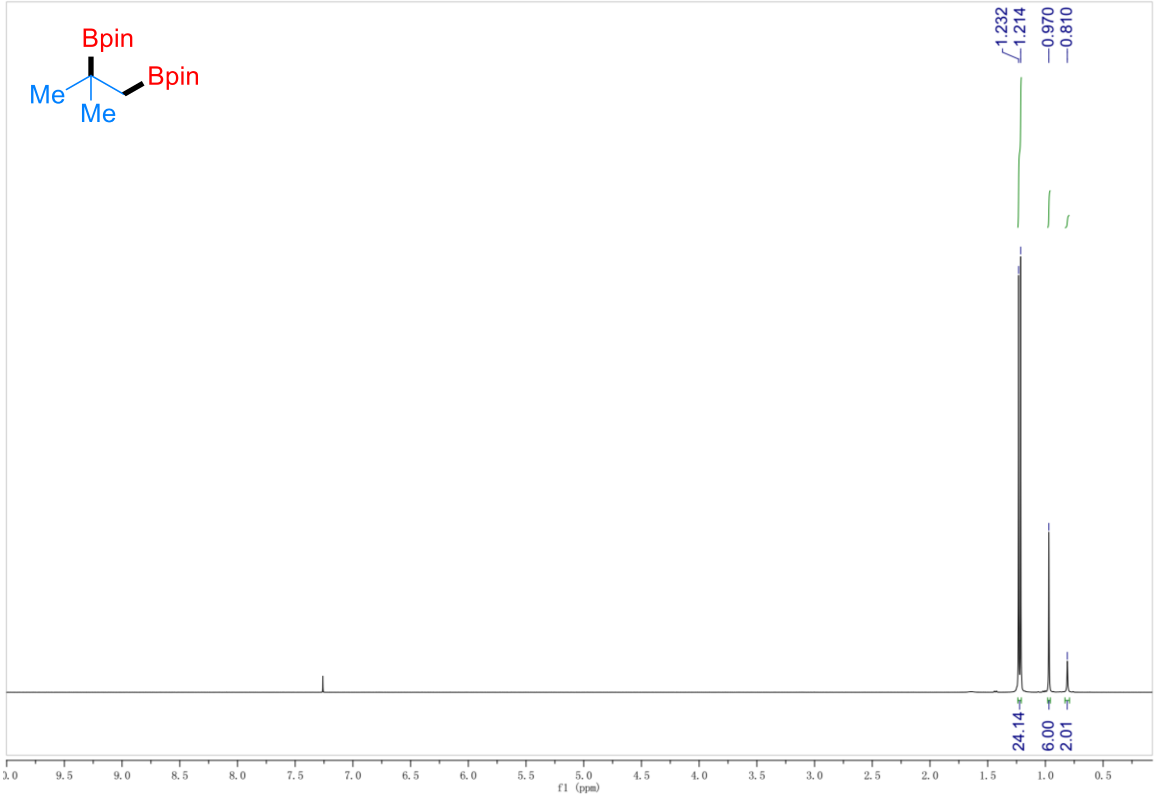

^1H NMR spectrum of compound **41b** in $CDCl_3$ (400 MHz).

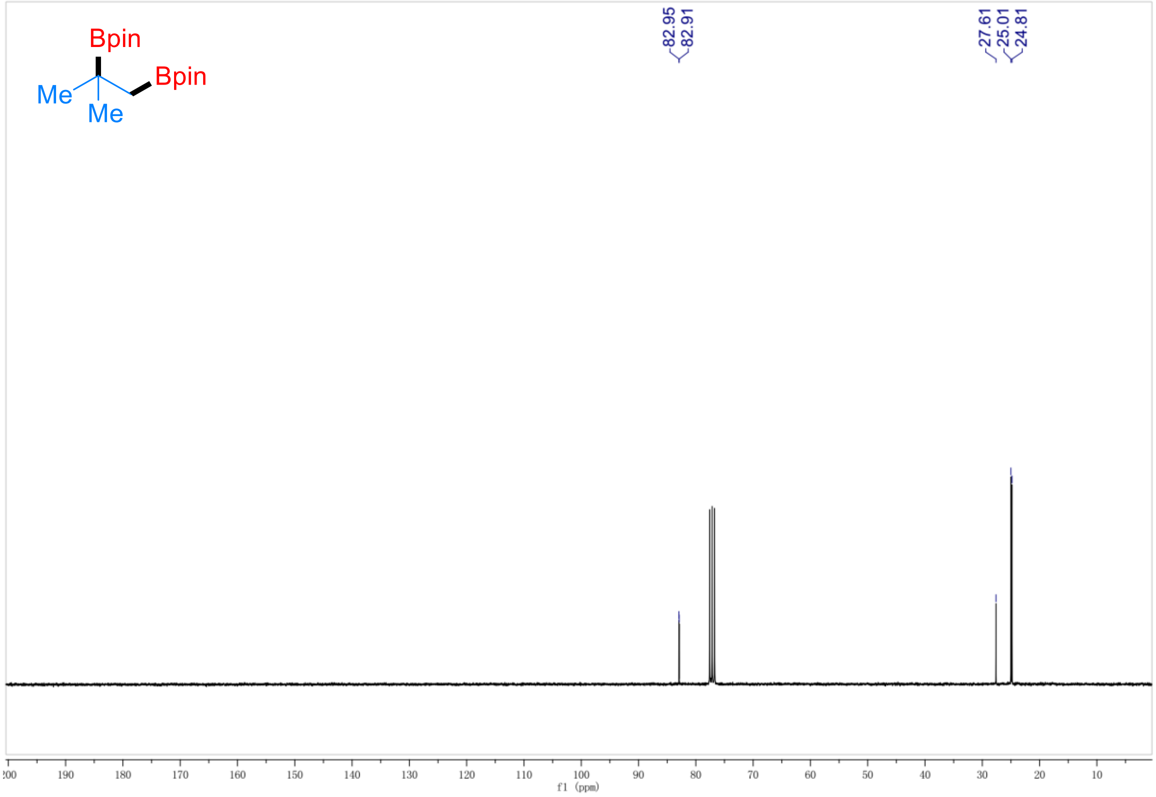

$^{13}C\{^{1}H\}$ NMR spectrum of compound **41b** in $CDCl_3$ (100 MHz).

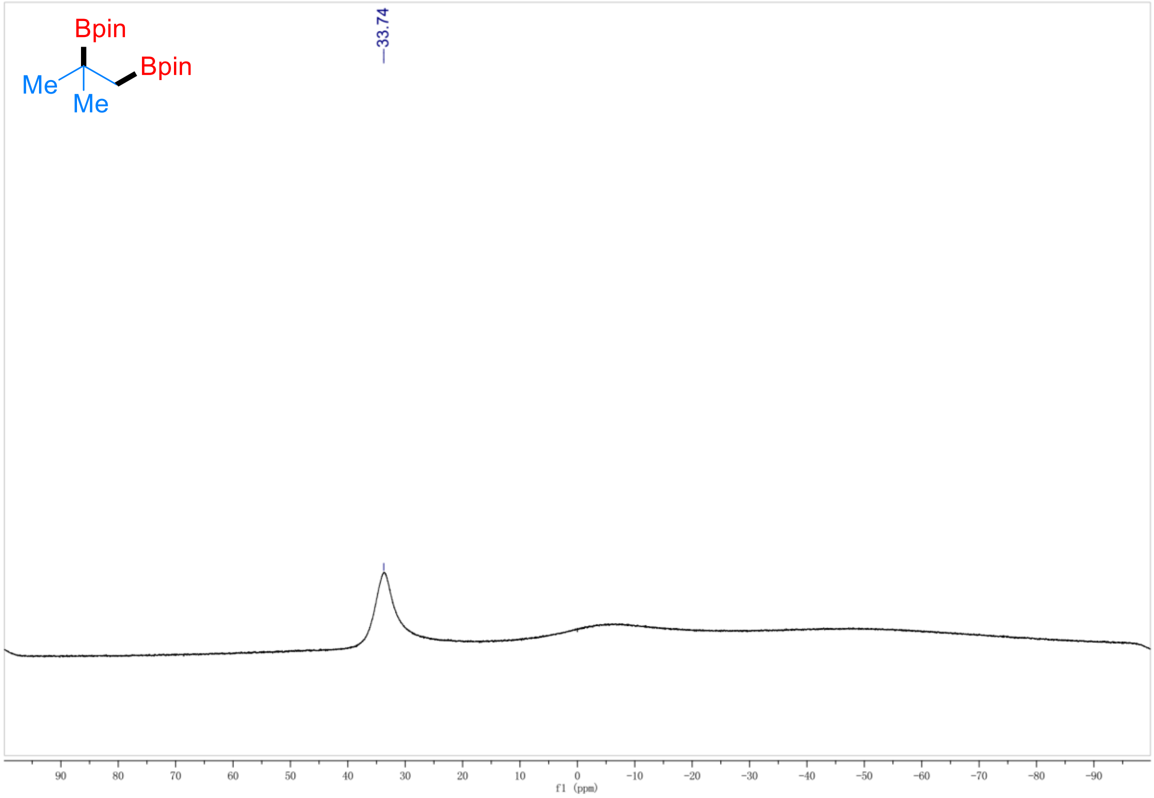

$^{11}B\{^{1}H\}$ NMR spectrum of compound **41b** in $CDCl_3$ (128 MHz).

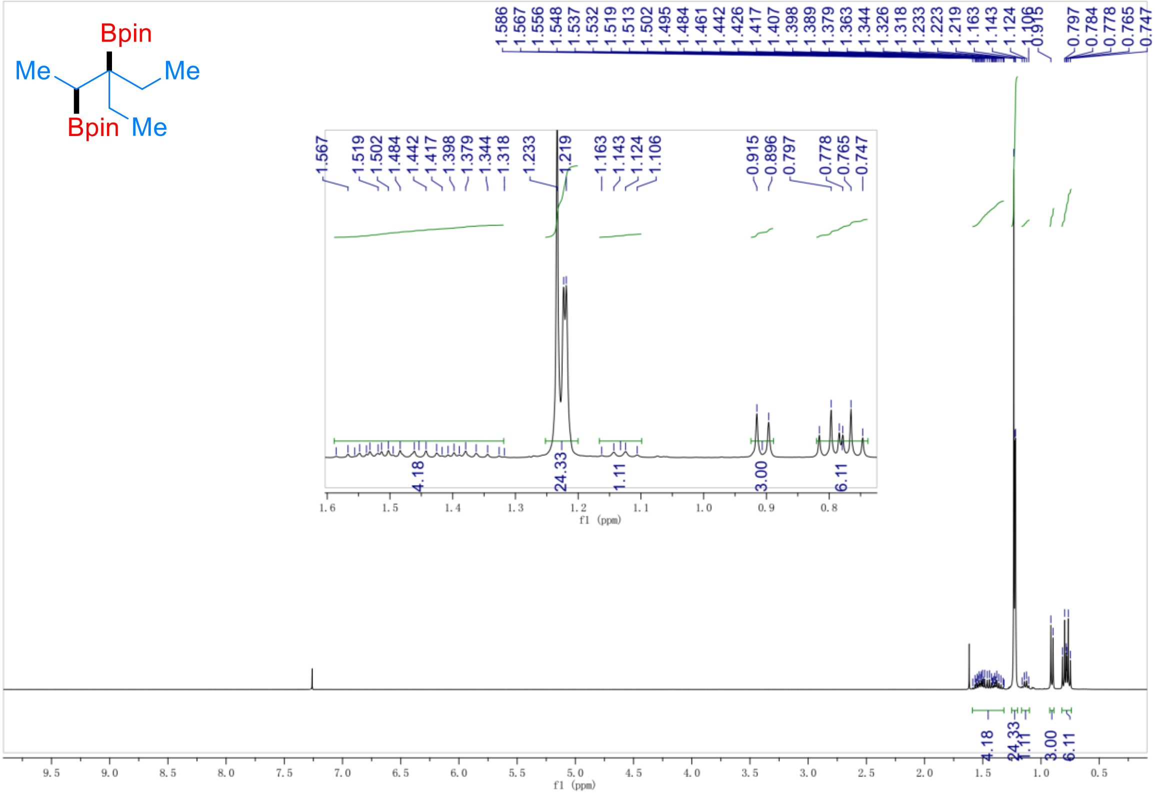

^{1}H NMR spectrum of compound **43b** in $CDCl_3$ (400 MHz).

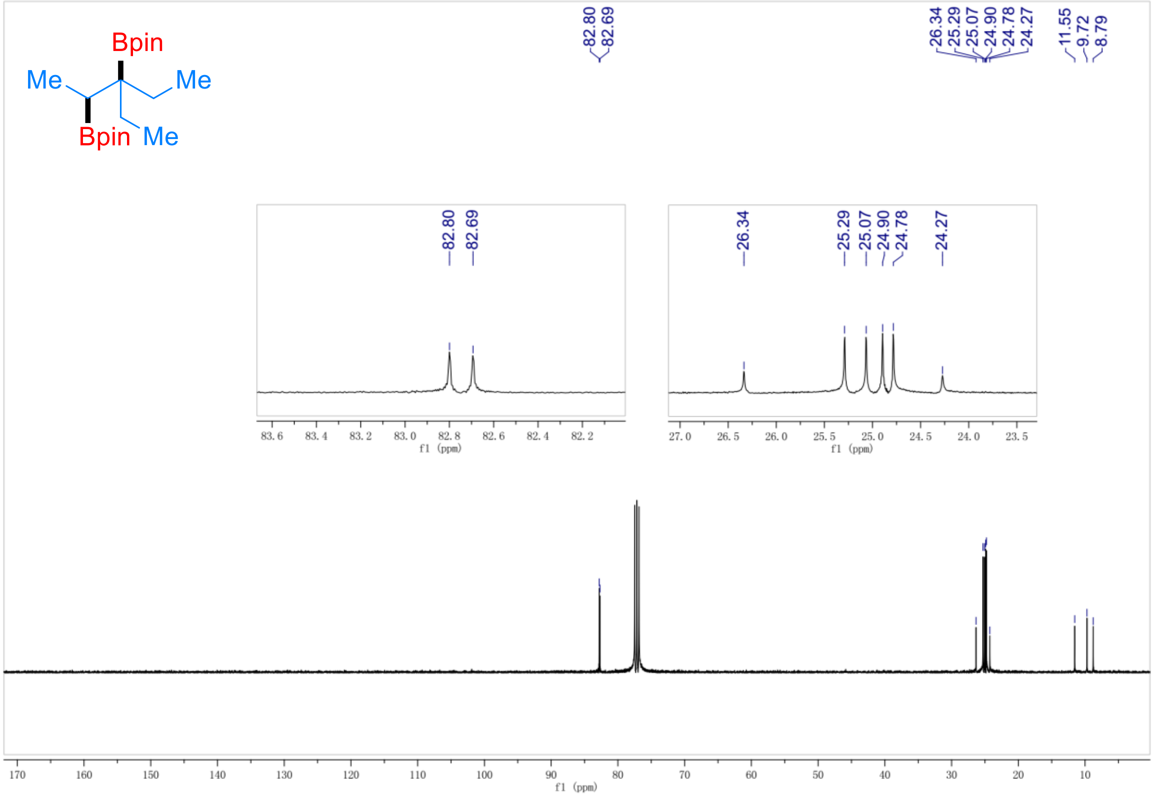

^{13}C{^{1}H} NMR spectrum of compound **43b** in $CDCl_3$ (100 MHz).

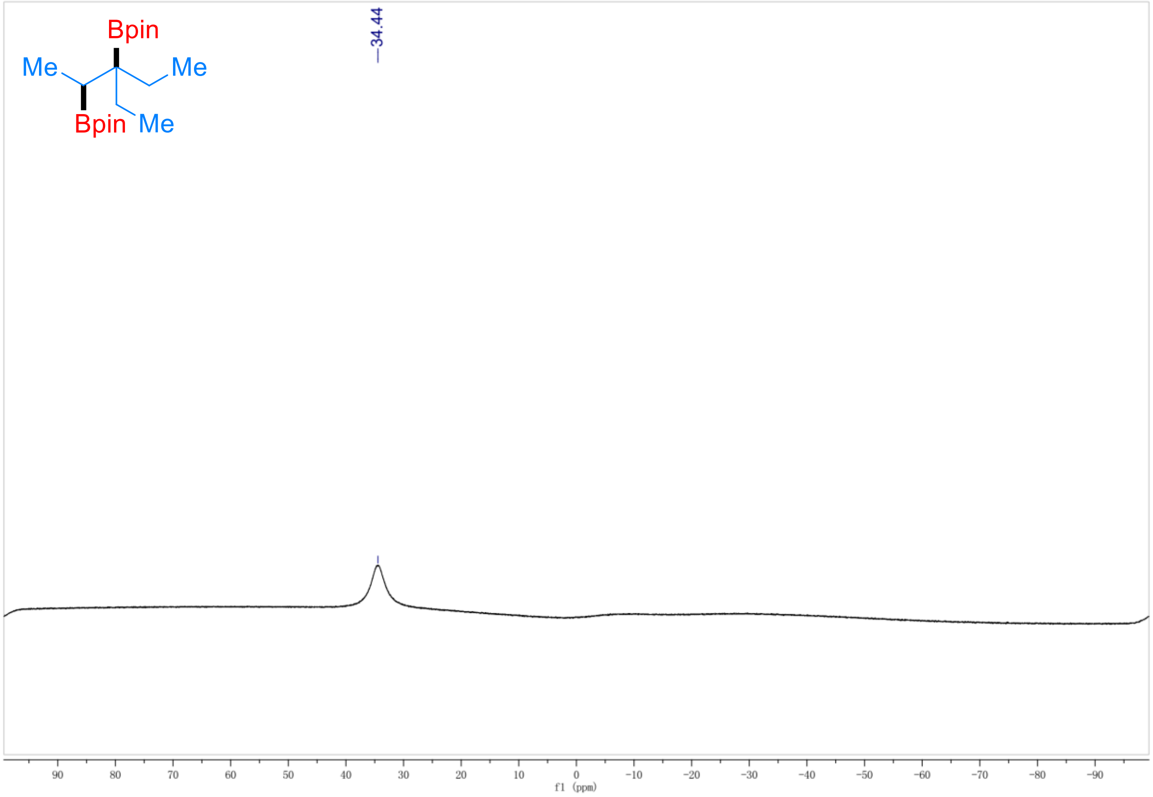

$^{11}B\{^1H\}$ NMR spectrum of compound **43b** in $CDCl_3$ (128 MHz).

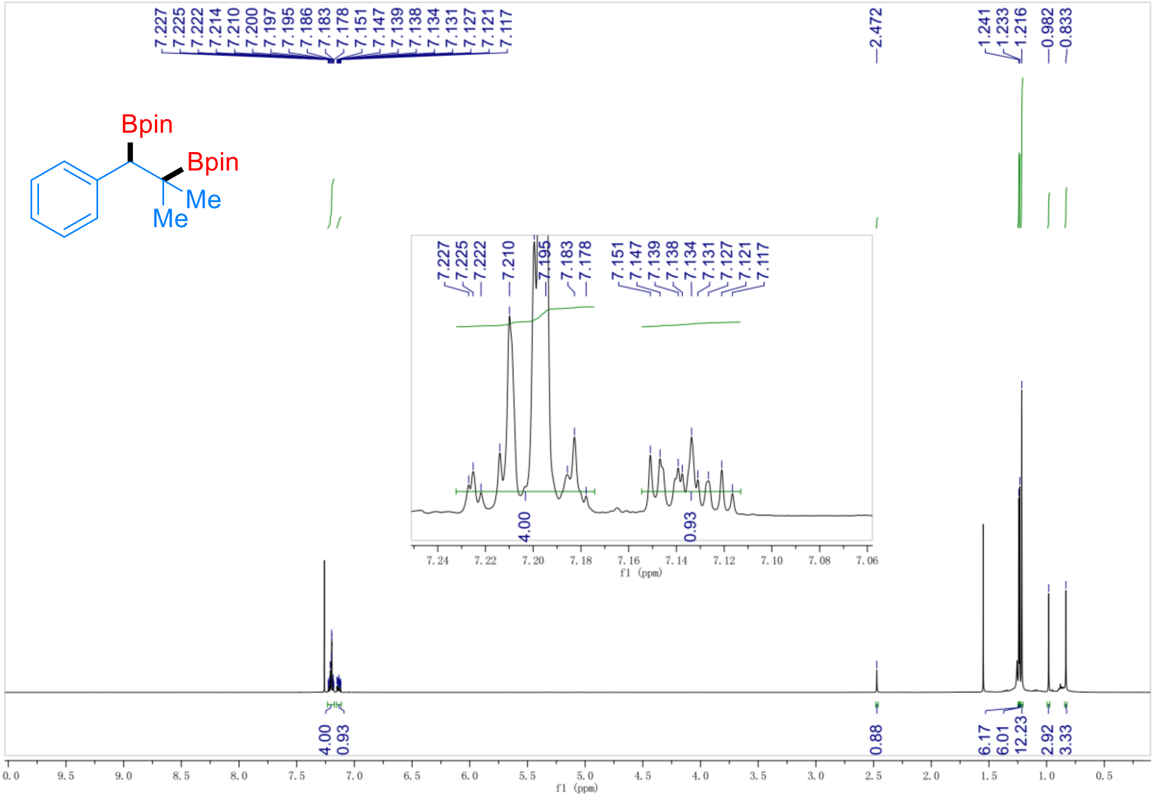

^1H NMR spectrum of compound **44b** in $CDCl_3$ (500 MHz).

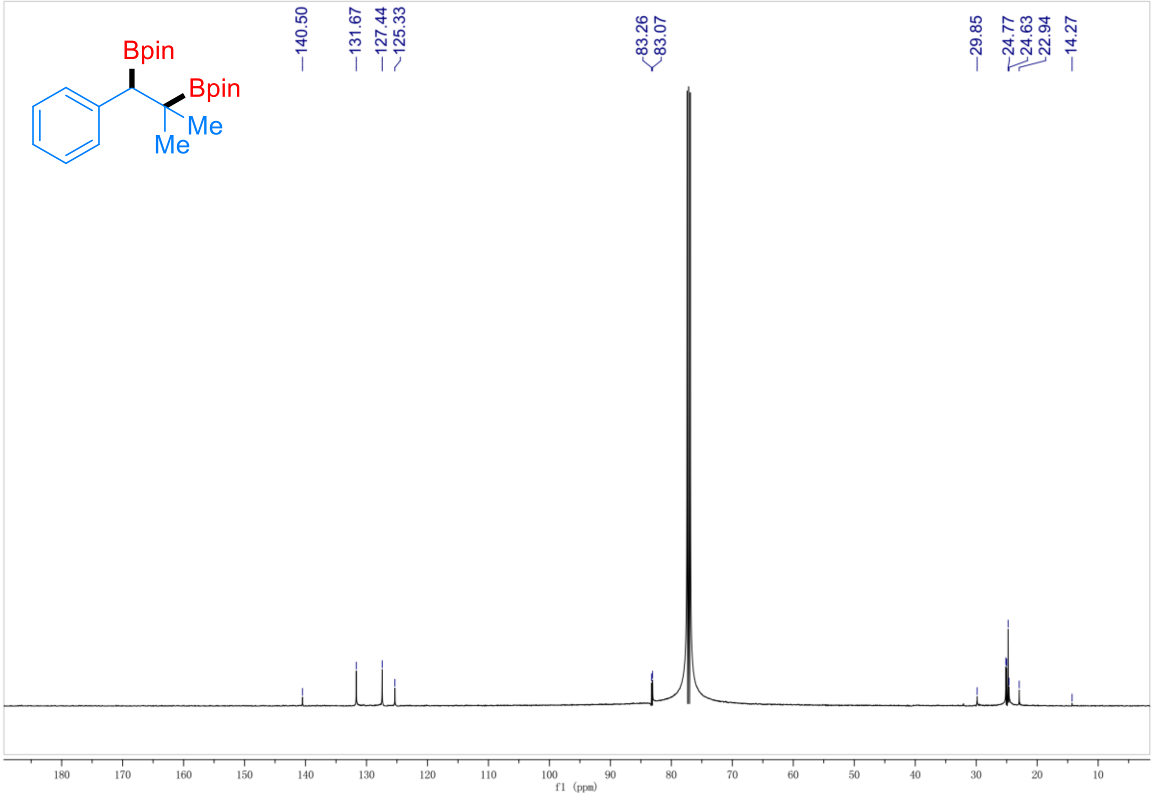

$^{13}C\{^{1}H\}$ NMR spectrum of compound **44b** in $CDCl_3$ (125 MHz).

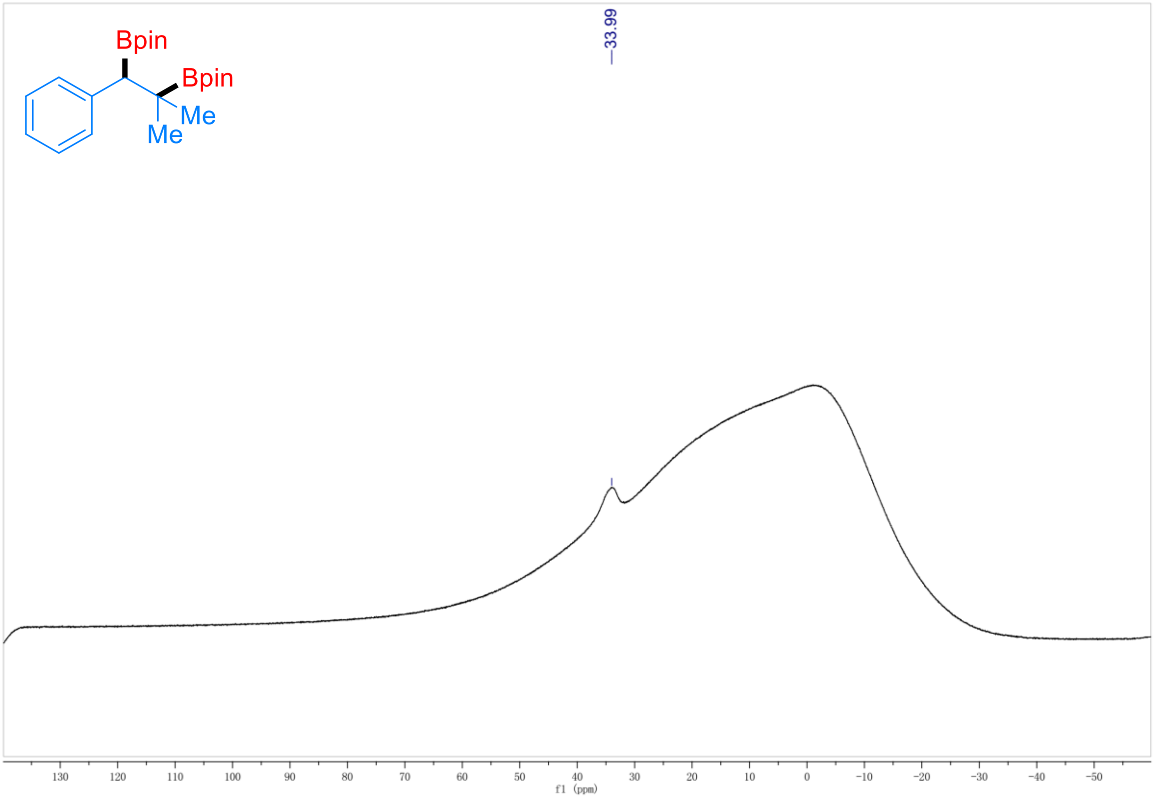

$^{11}B\{^{1}H\}$ NMR spectrum of compound **44b** in $CDCl_3$ (160 MHz).

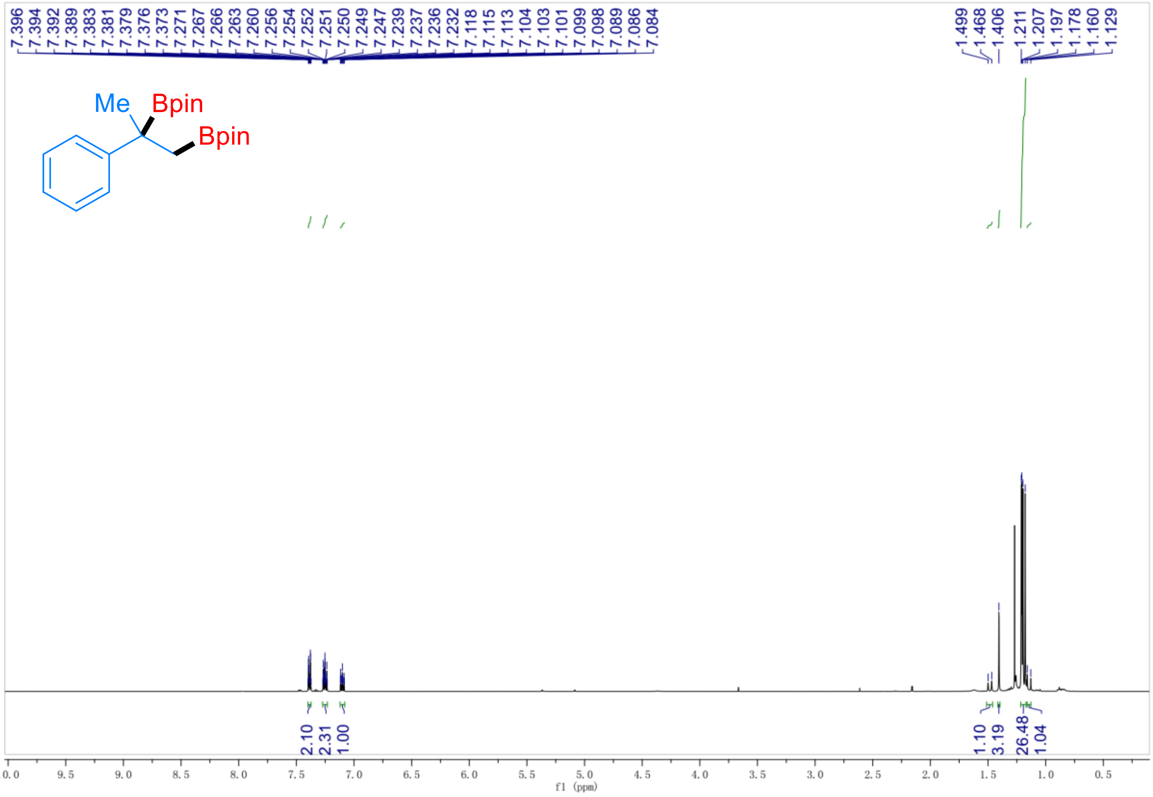

^{1}H NMR spectrum of compound **45b** in $CDCl_3$ (500 MHz).

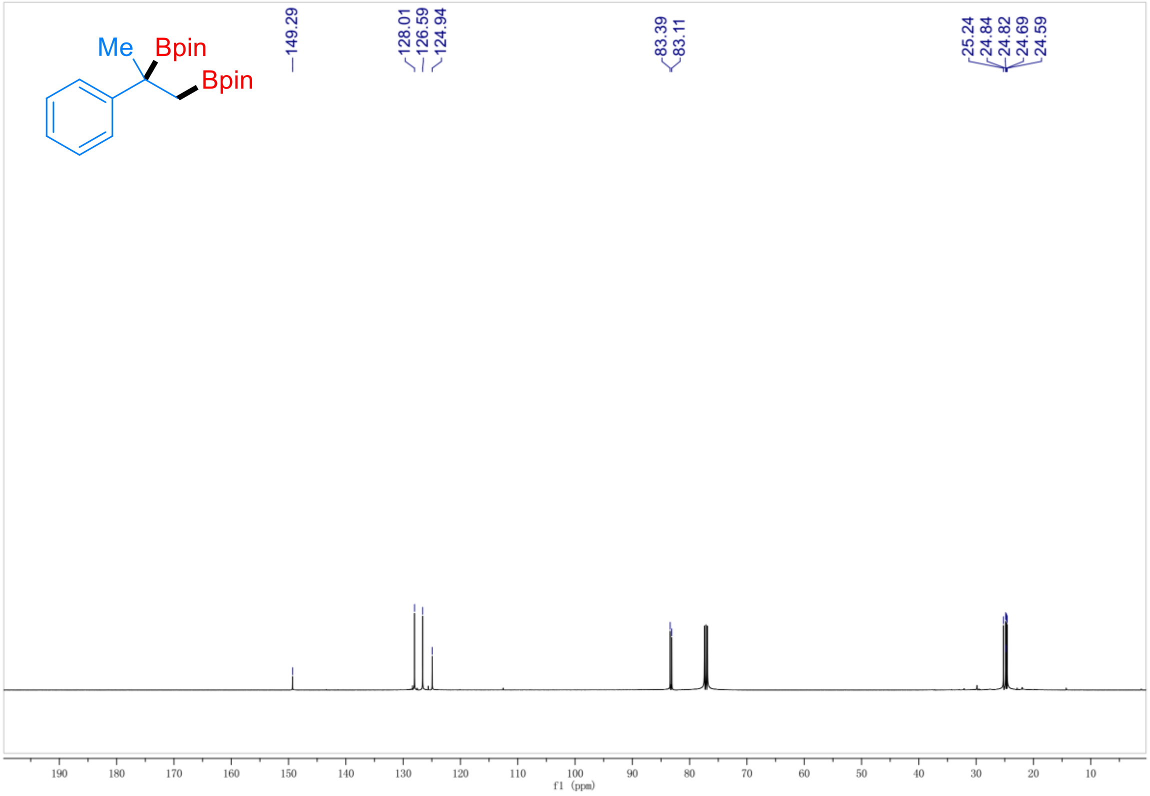

^{13}C{^{1}H} NMR spectrum of compound **45b** in $CDCl_3$ (125 MHz).

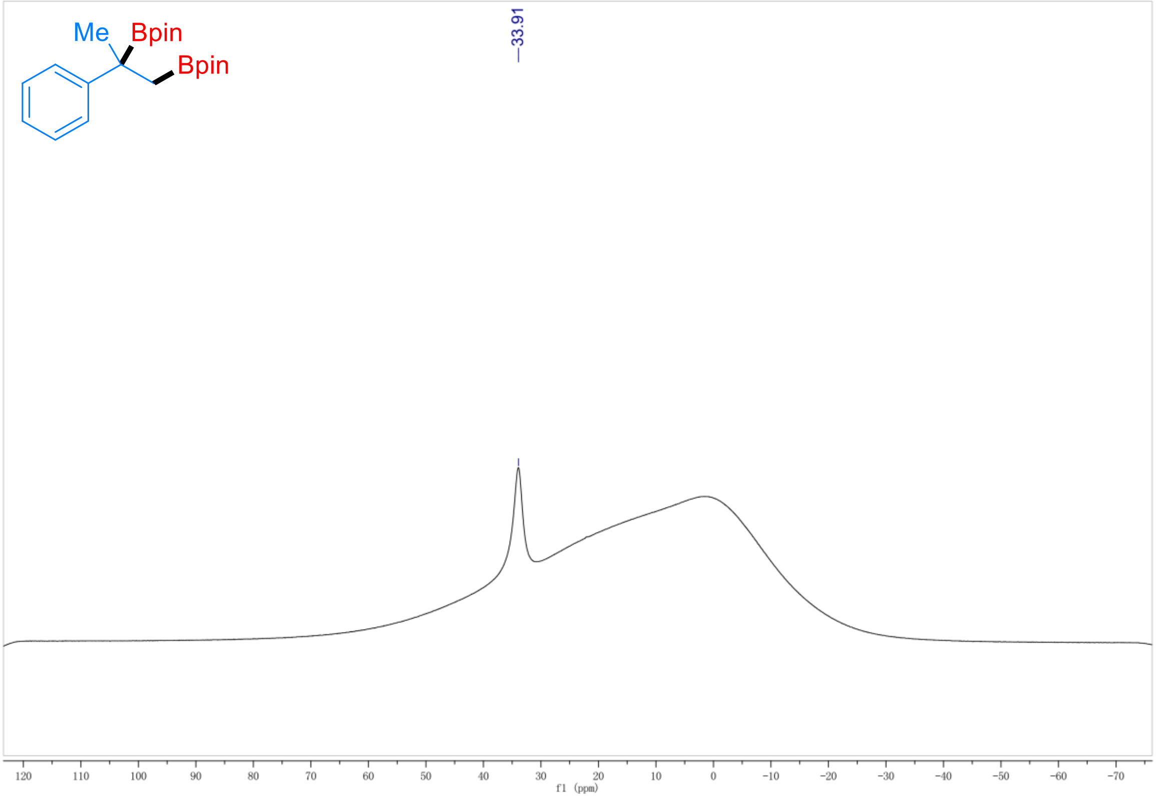

$^{11}B\{^1H\}$ NMR spectrum of compound **45b** in $CDCl_3$ (160 MHz).

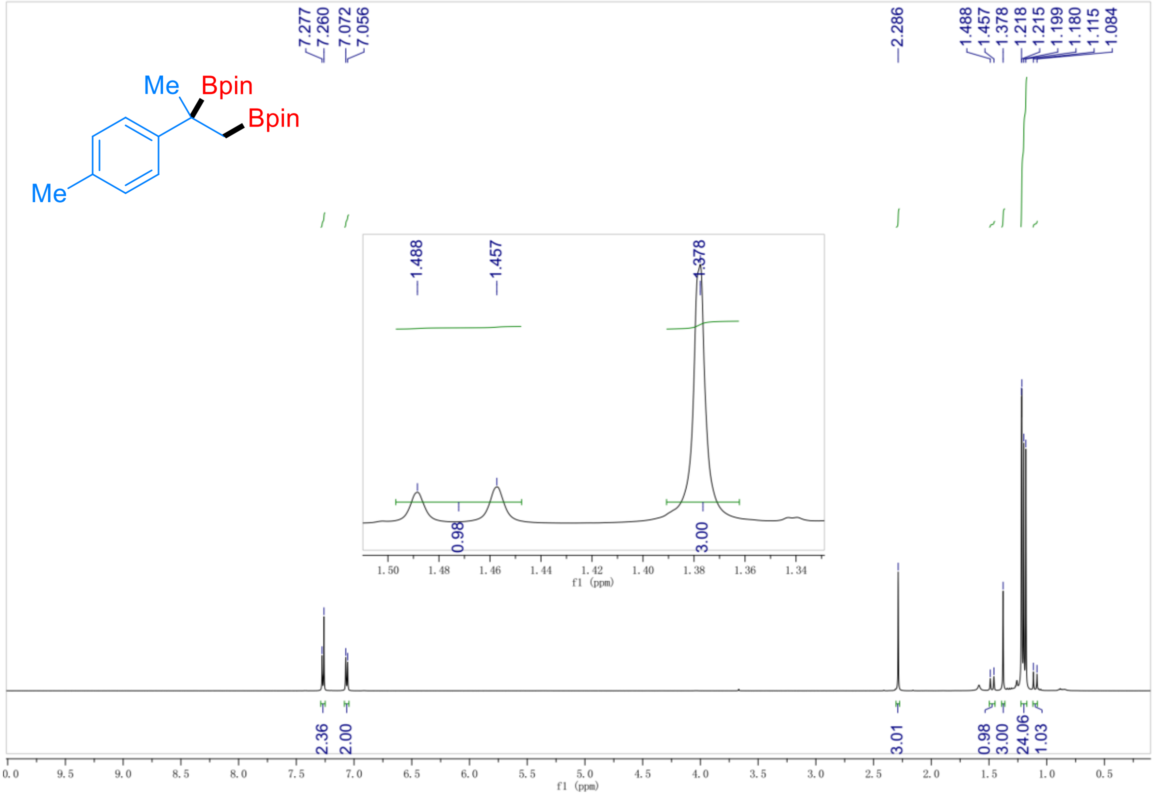

^1H NMR spectrum of compound **46b** in $CDCl_3$ (500 MHz).

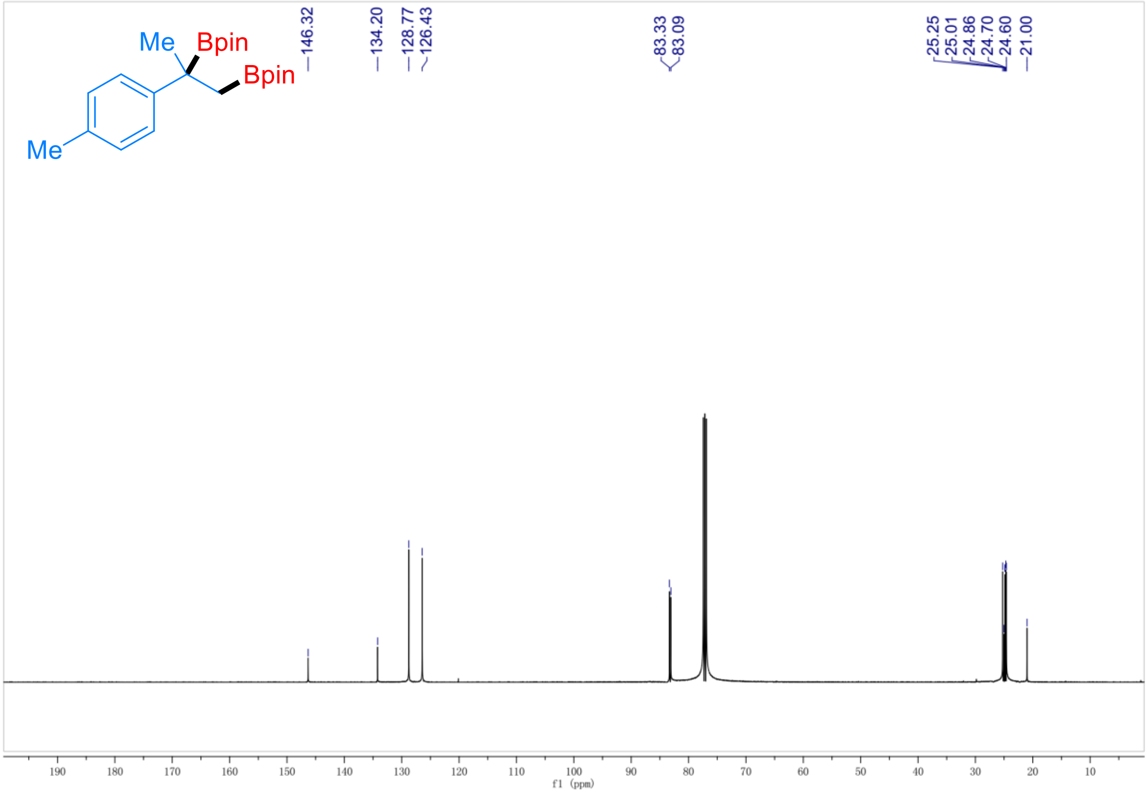

$^{13}C\{^1H\}$ NMR spectrum of compound **46b** in $CDCl_3$ (125 MHz).

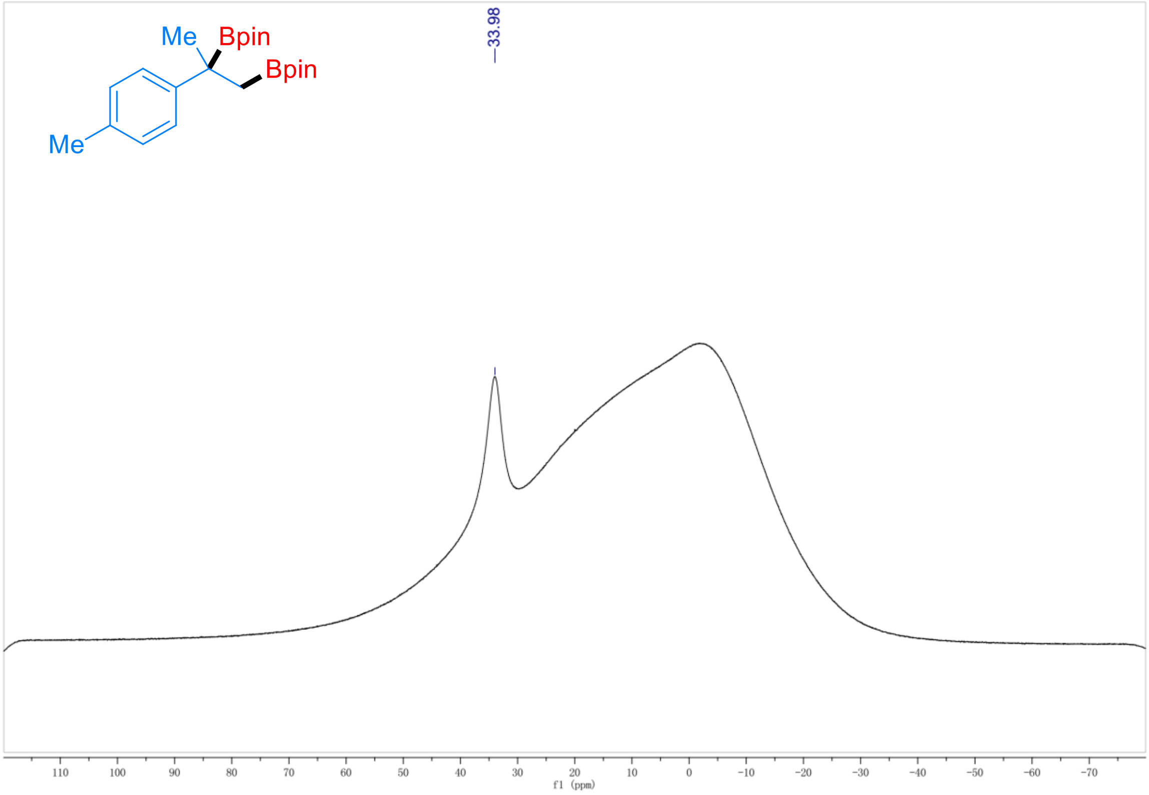

$^{11}B\{^1H\}$ NMR spectrum of compound **46b** in $CDCl_3$ (160 MHz).

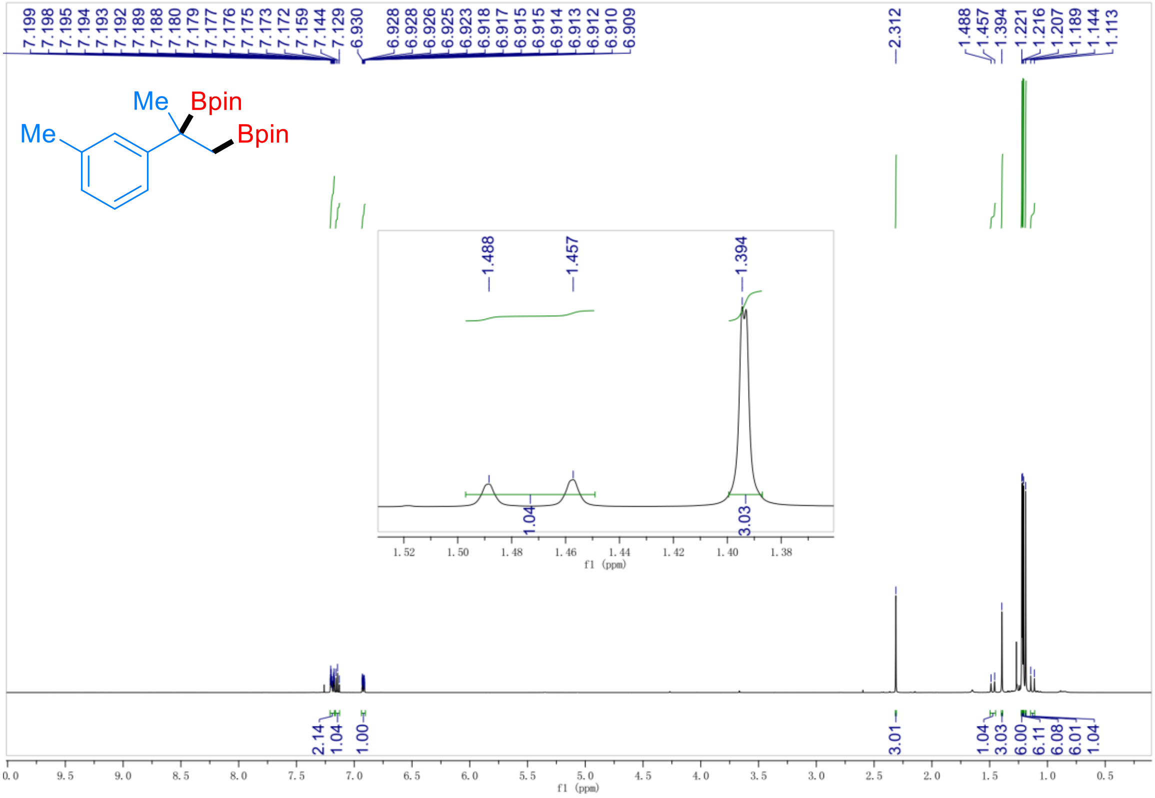

^{1}H NMR spectrum of compound **47b** in $CDCl_3$ (500 MHz).

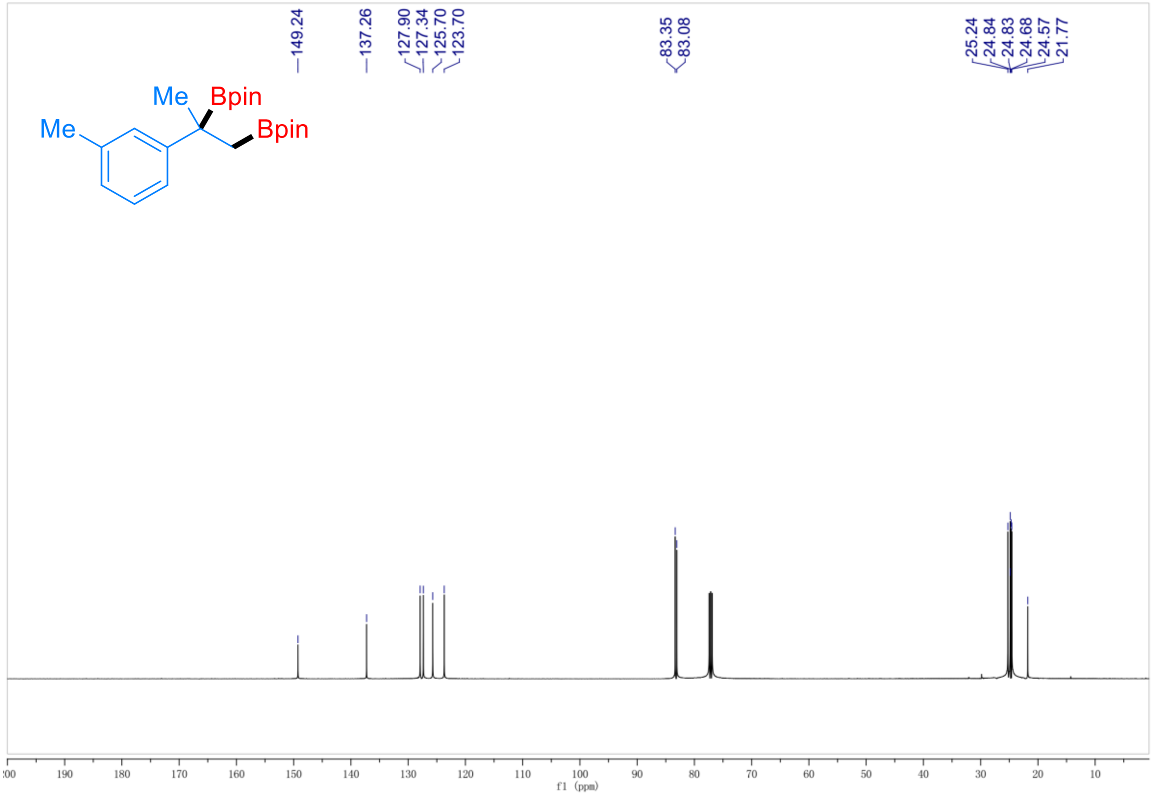

$^{13}C\{^{1}H\}$ NMR spectrum of compound **47b** in $CDCl_3$ (125 MHz).

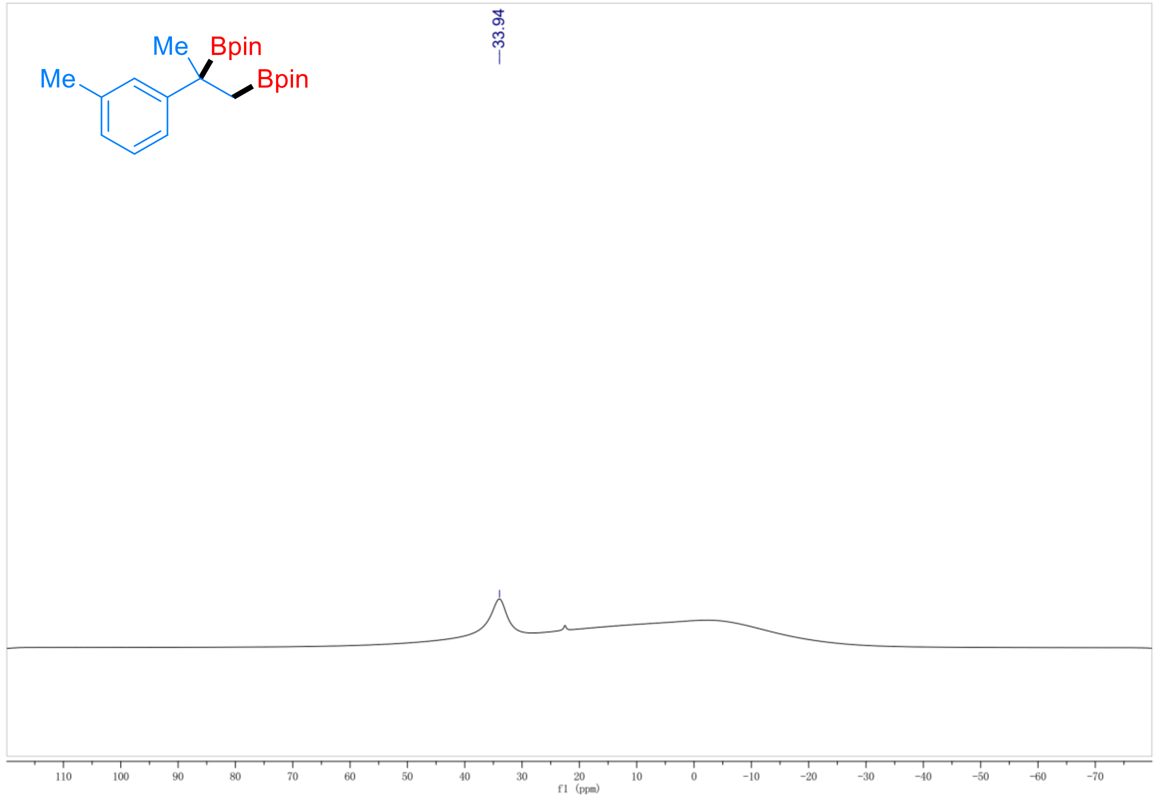

$^{11}B\{^{1}H\}$ NMR spectrum of compound **47b** in $CDCl_3$ (160 MHz).

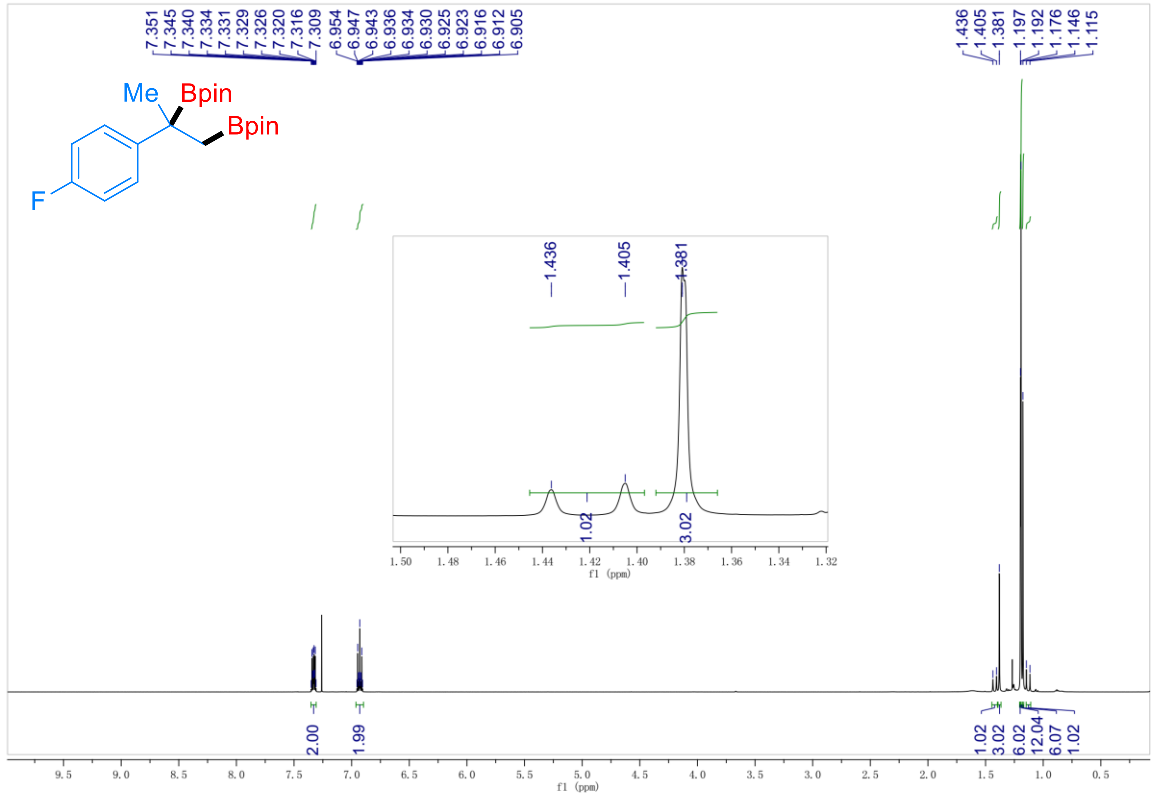

^{1}H NMR spectrum of compound **48b** in $CDCl_3$ (500 MHz).

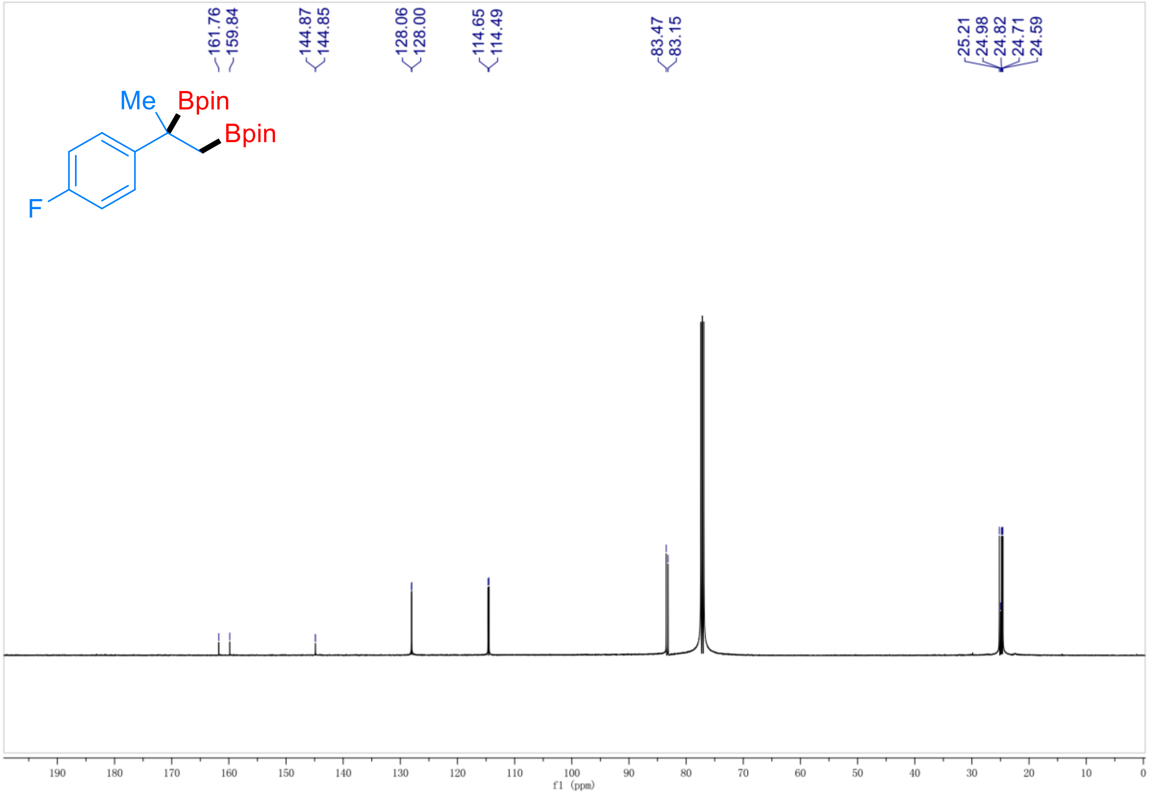

$^{13}C\{^{1}H\}$ NMR spectrum of compound **48b** in $CDCl_3$ (125 MHz).

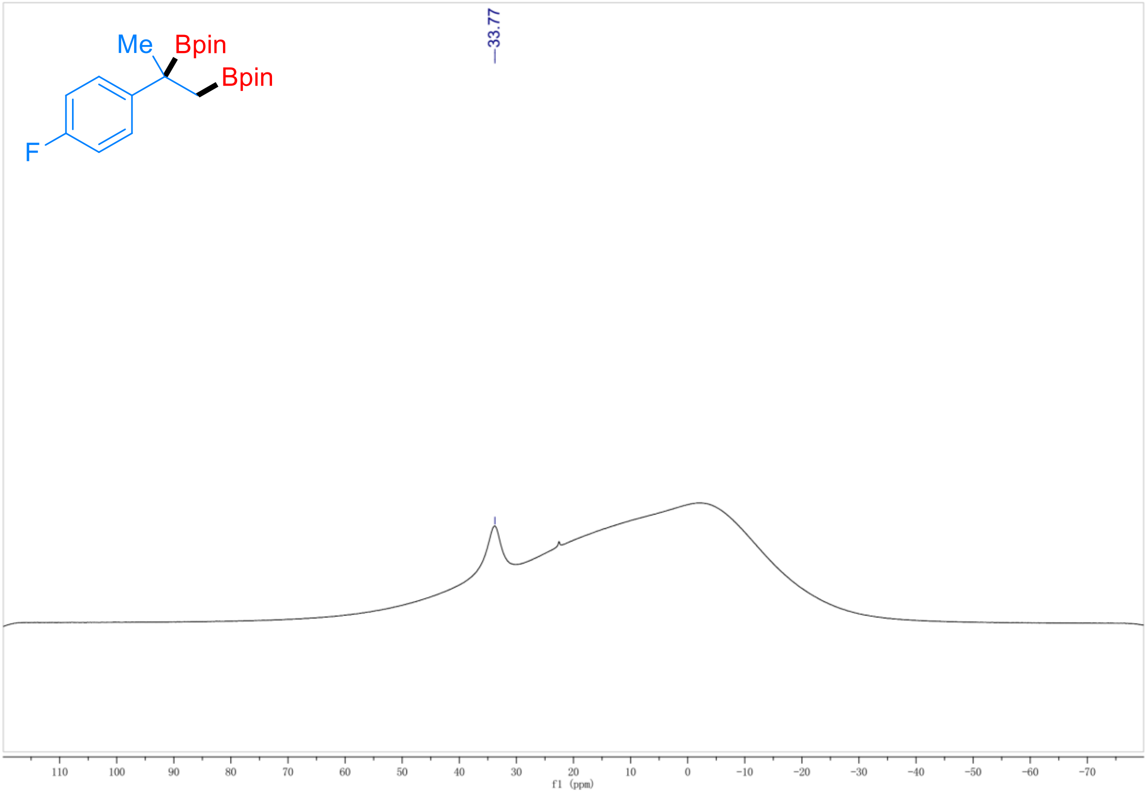

$^{11}B\{^{1}H\}$ NMR spectrum of compound **48b** in $CDCl_3$ (160 MHz).

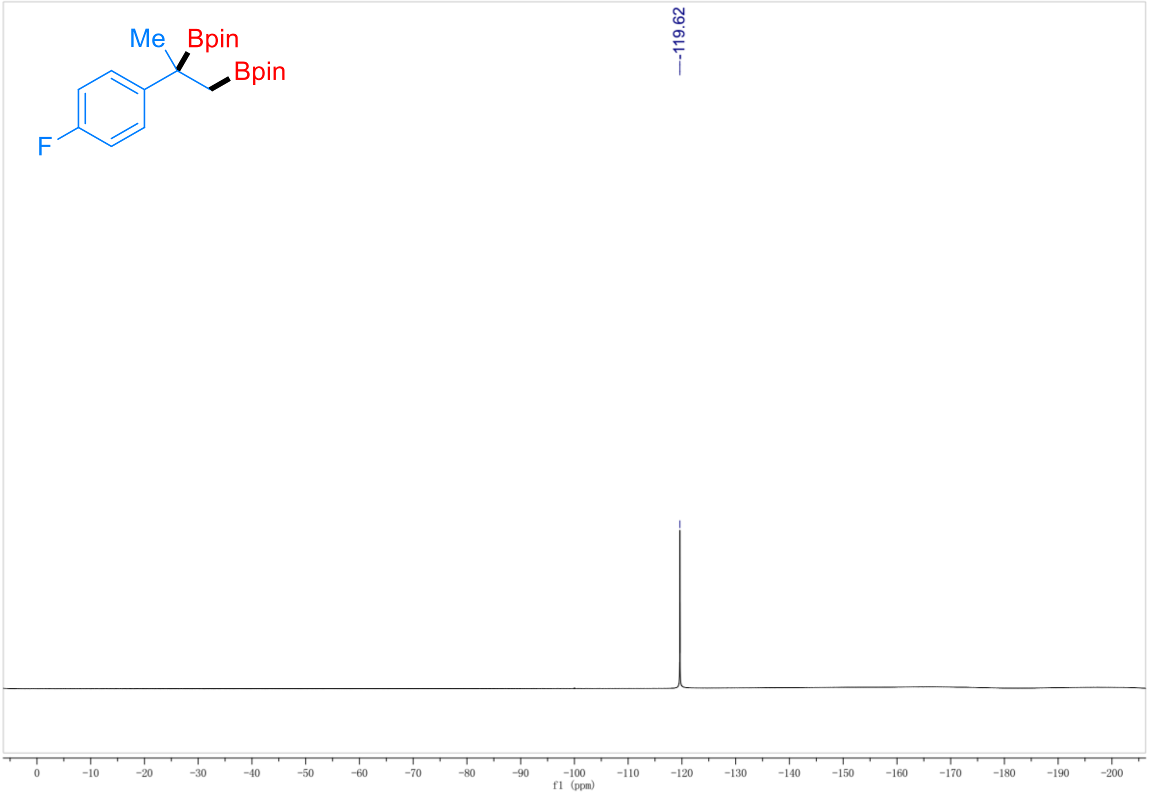

$^{19}F\{^{1}H\}$ NMR spectrum of compound **48a** in $CDCl_3$ (470 MHz).

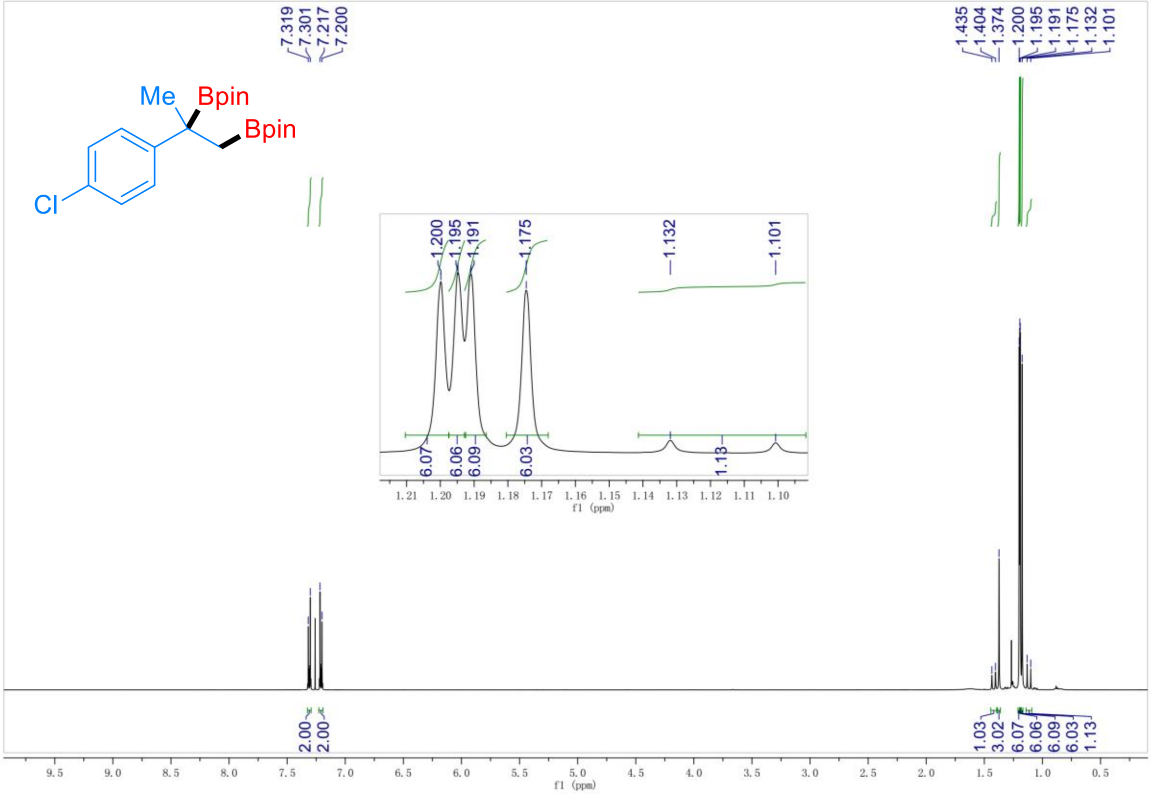

^{1}H NMR spectrum of compound **49b** in $CDCl_3$ (500 MHz).

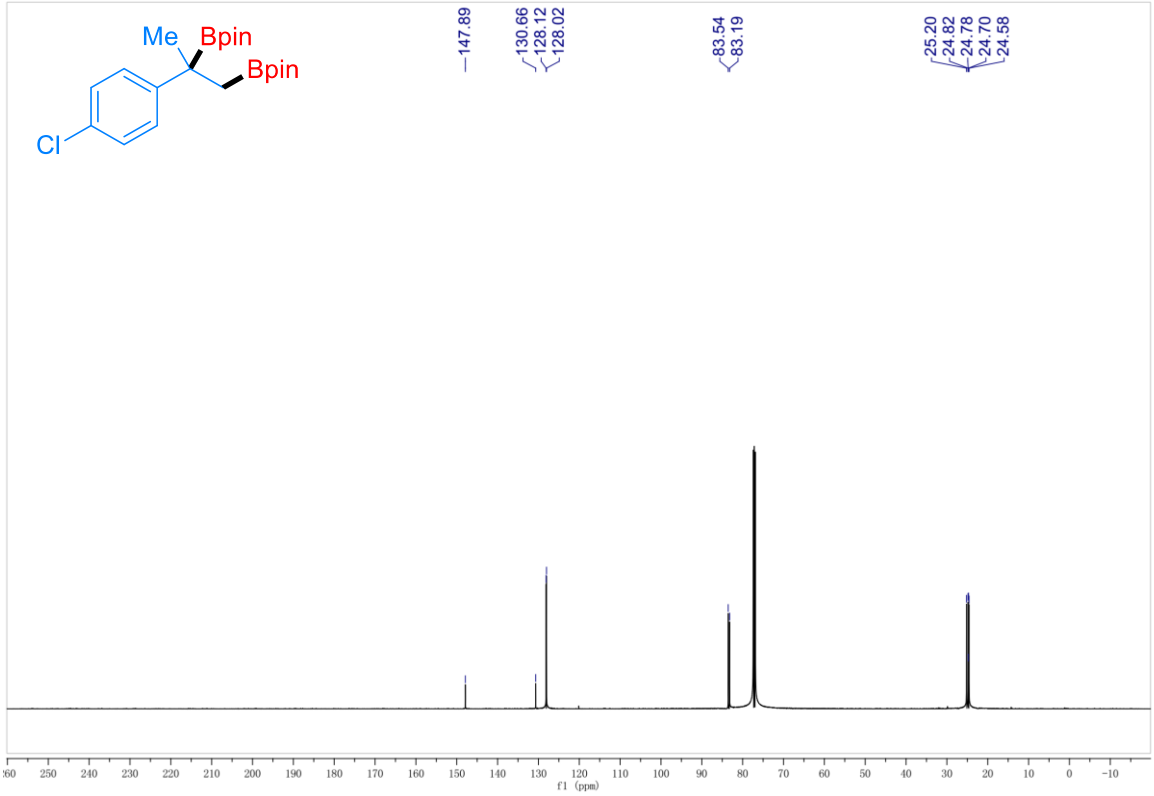

$^{13}C\{^1H\}$ NMR spectrum of compound **49b** in $CDCl_3$ (125 MHz).

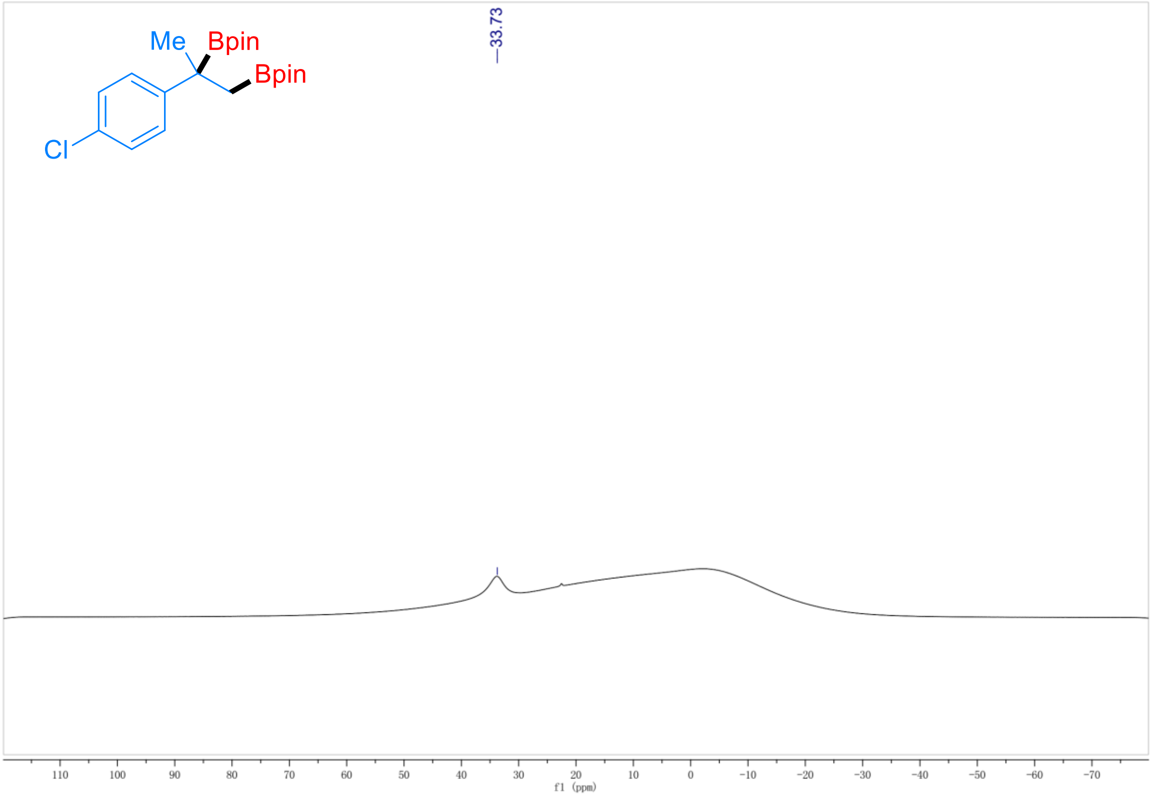

$^{11}B\{^1H\}$ NMR spectrum of compound **49b** in $CDCl_3$ (160 MHz).

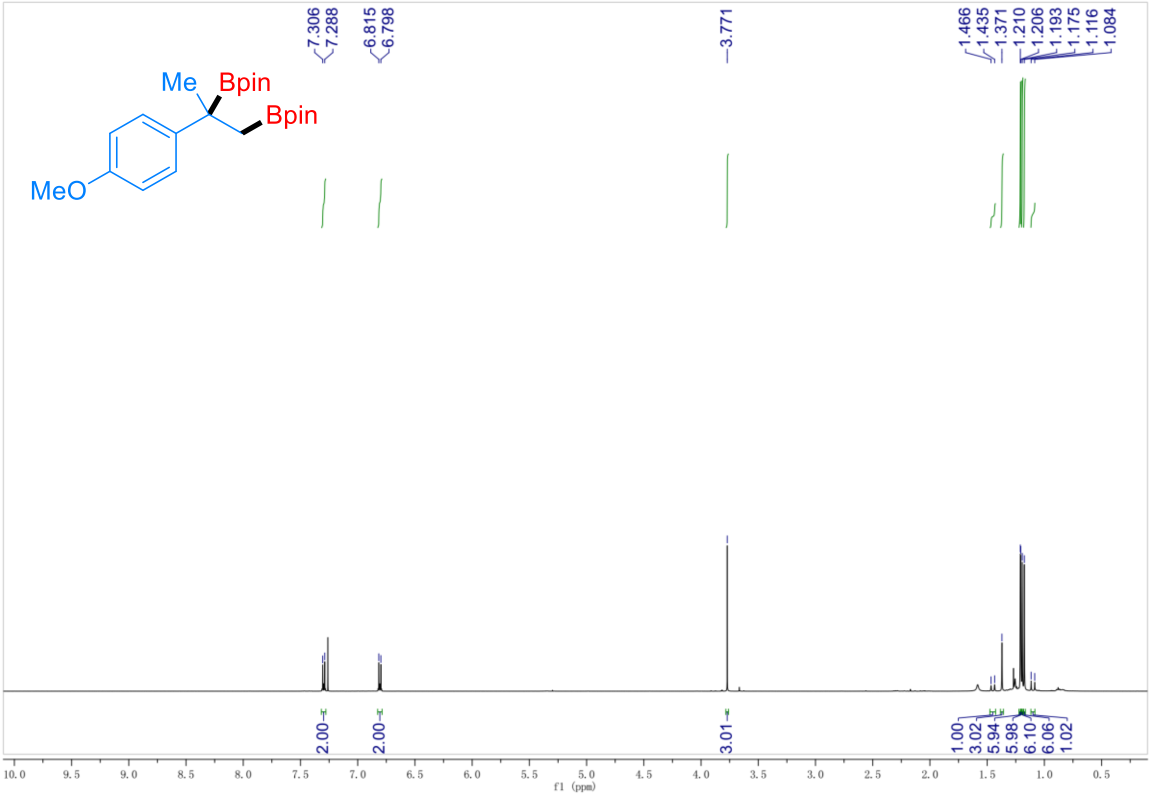

^{1}H NMR spectrum of compound **50b** in $CDCl_3$ (500 MHz).

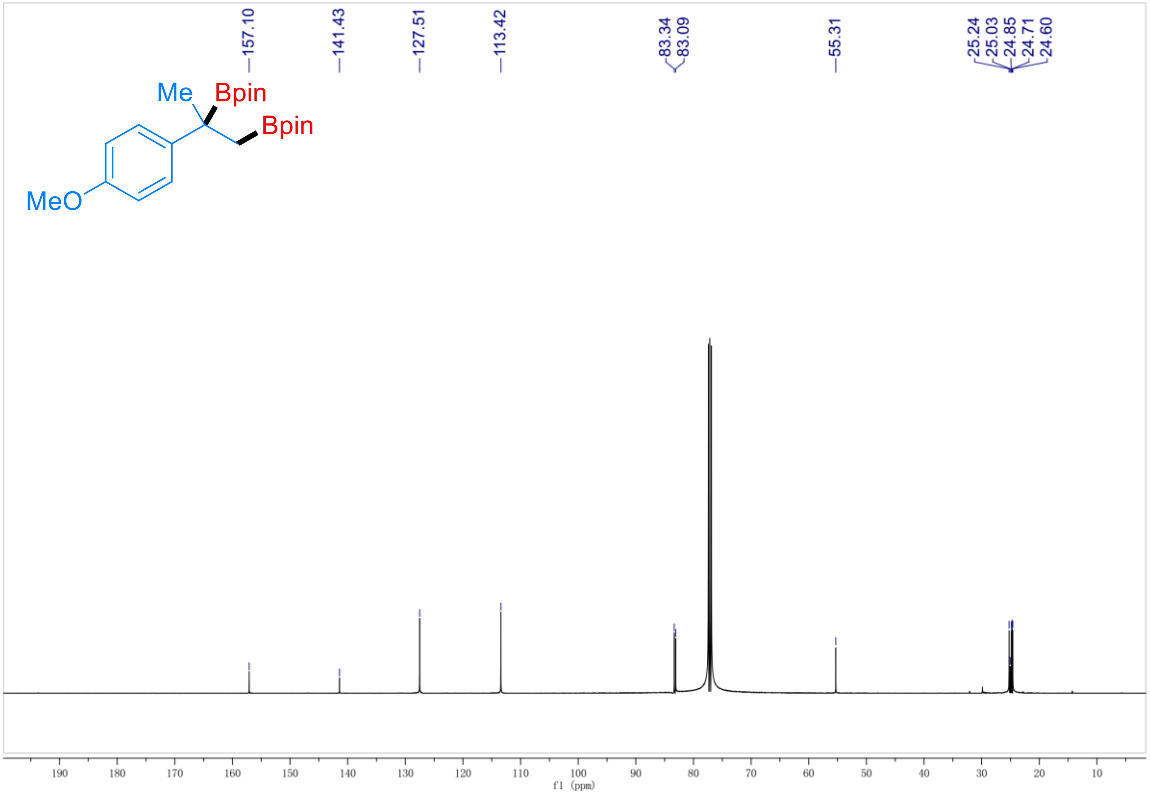

^{13}C{^{1}H} NMR spectrum of compound **50b** in $CDCl_3$ (125 MHz).

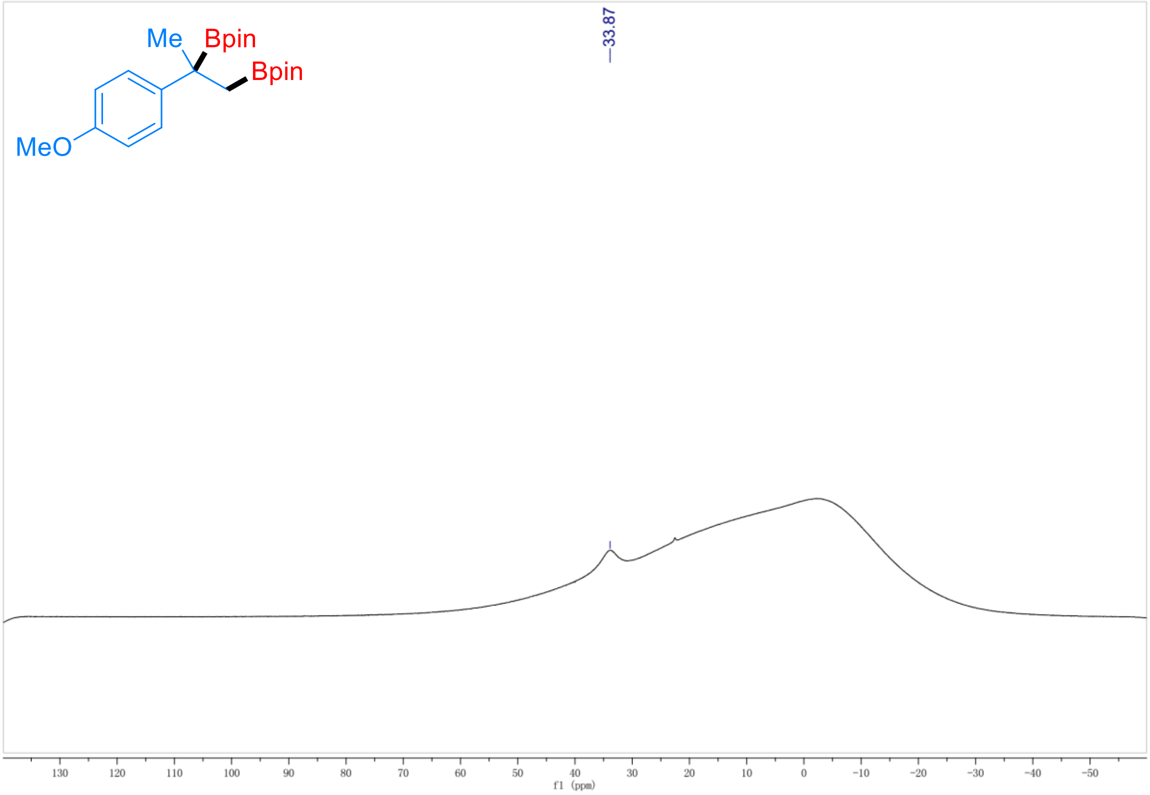

$^{11}B\{^{1}H\}$ NMR spectrum of compound **50b** in $CDCl_3$ (160 MHz).

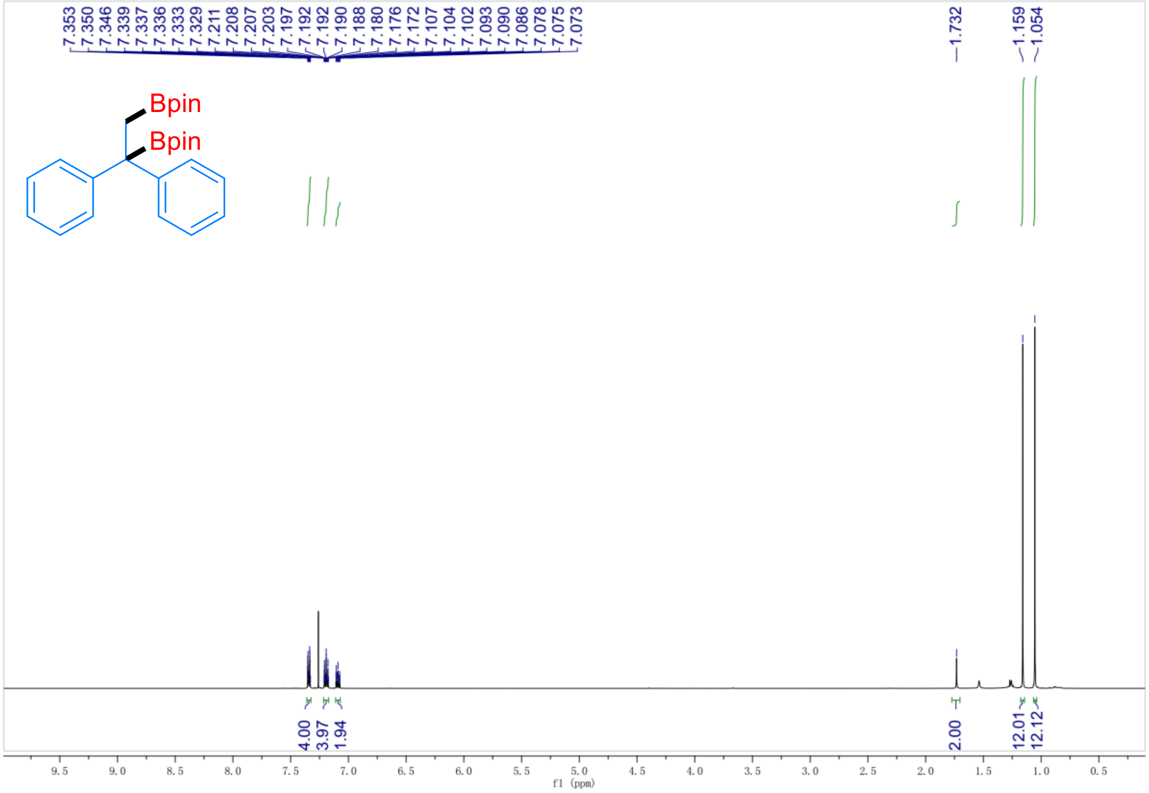

^{1}H NMR spectrum of compound **51b** in $CDCl_3$ (500 MHz).

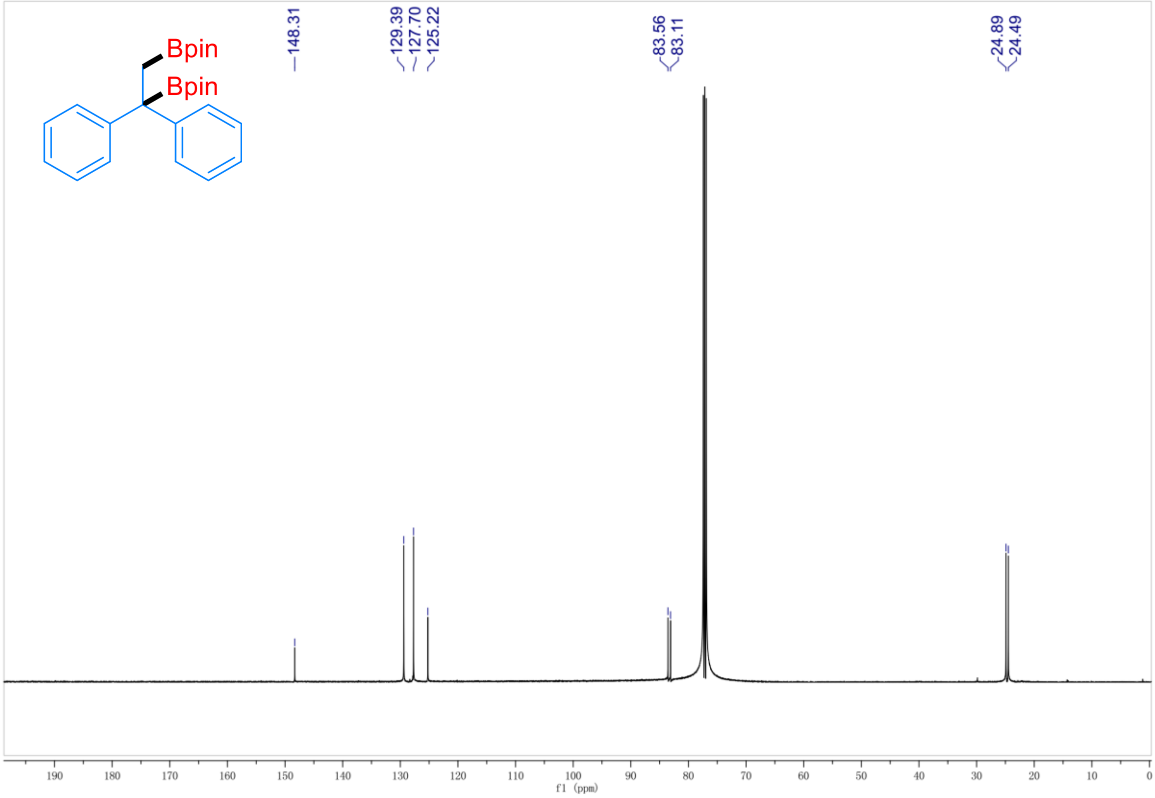

$^{13}C\{^{1}H\}$ NMR spectrum of compound **51b** in $CDCl_3$ (125 MHz).

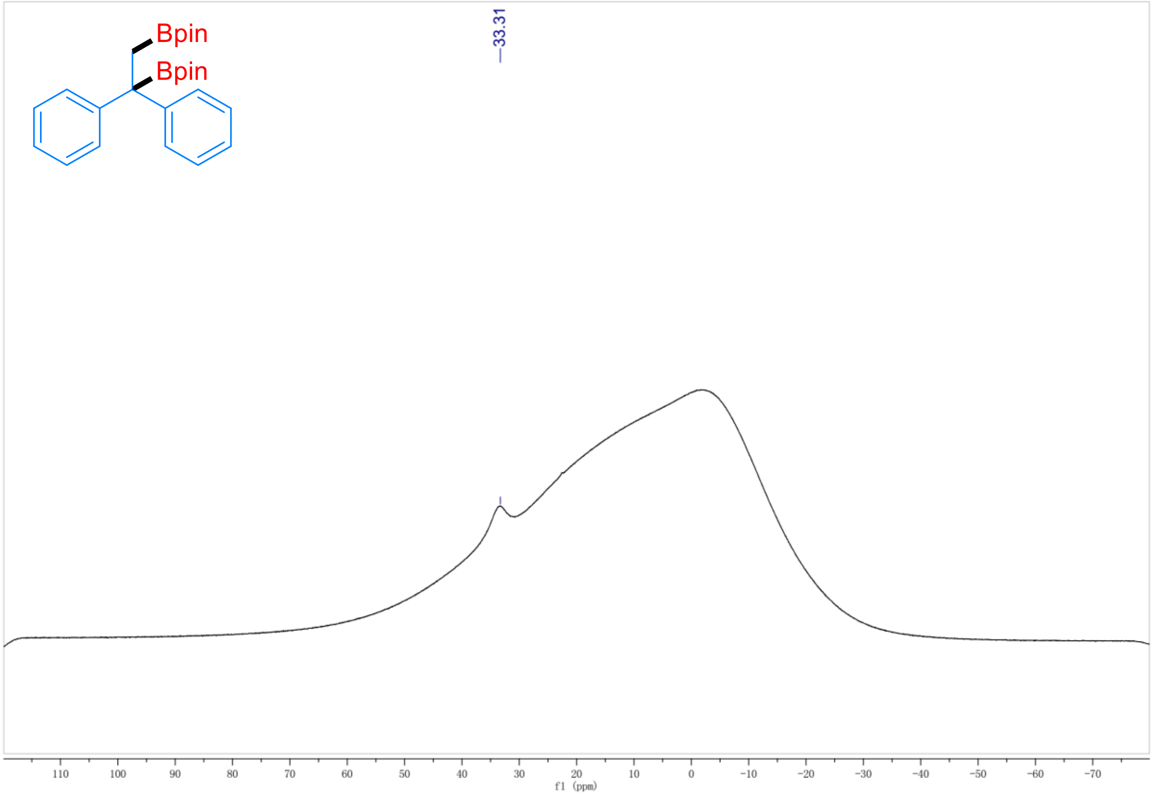

$^{11}B\{^{1}H\}$ NMR spectrum of compound **51b** in $CDCl_3$ (160 MHz).

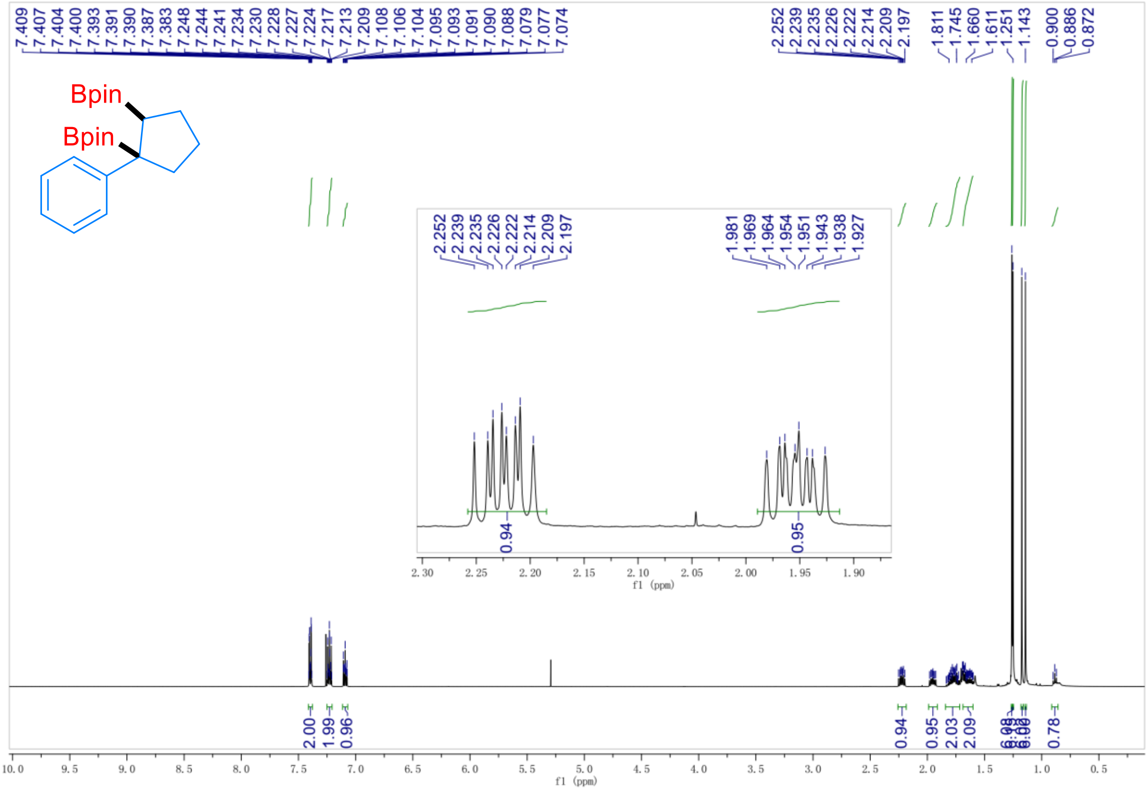

^{1}H NMR spectrum of compound **52b** in $CDCl_3$ (500 MHz).

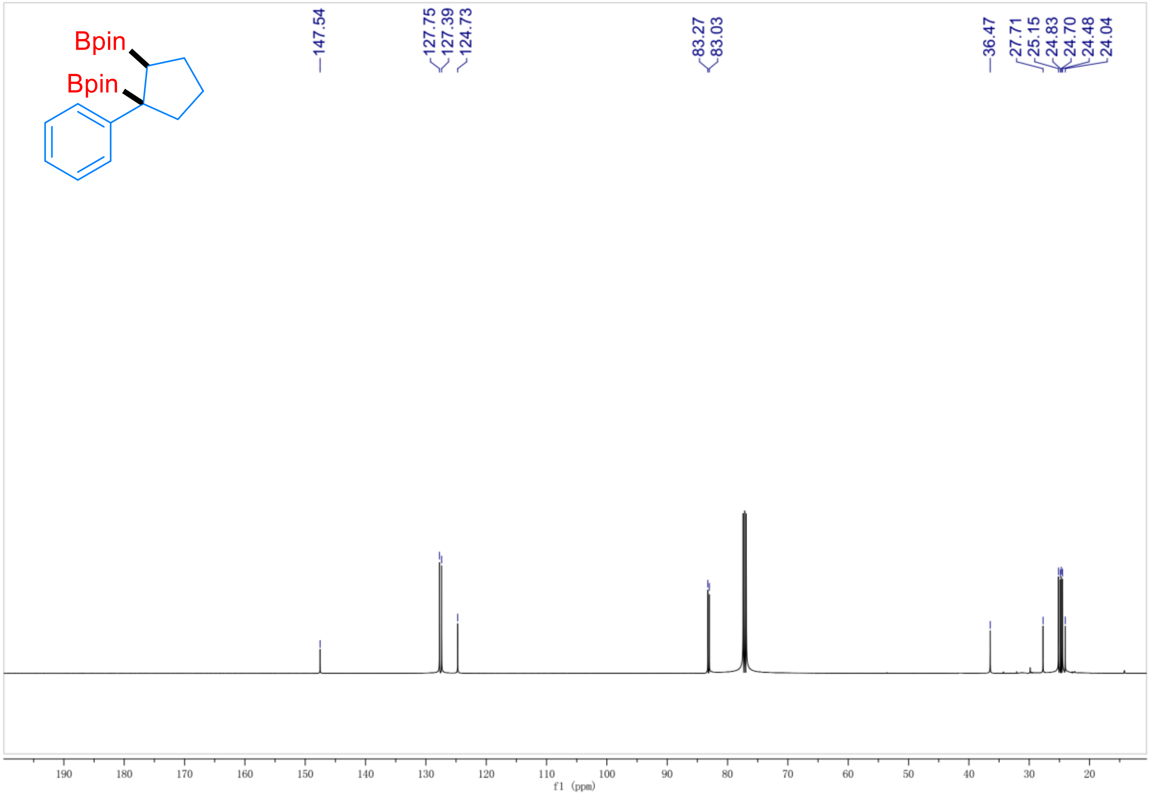

^{13}C{^{1}H} NMR spectrum of compound **52b** in $CDCl_3$ (125 MHz).

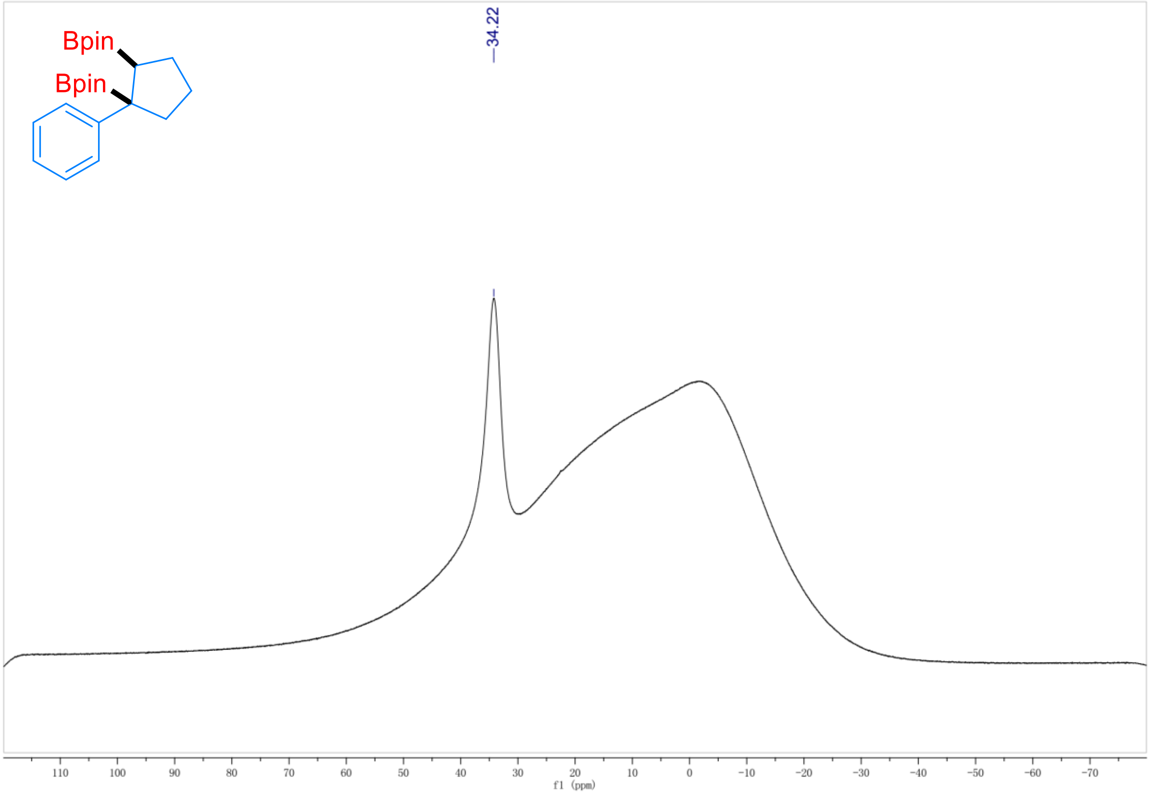

$^{11}B\{^{1}H\}$ NMR spectrum of compound **52b** in $CDCl_3$ (160 MHz).

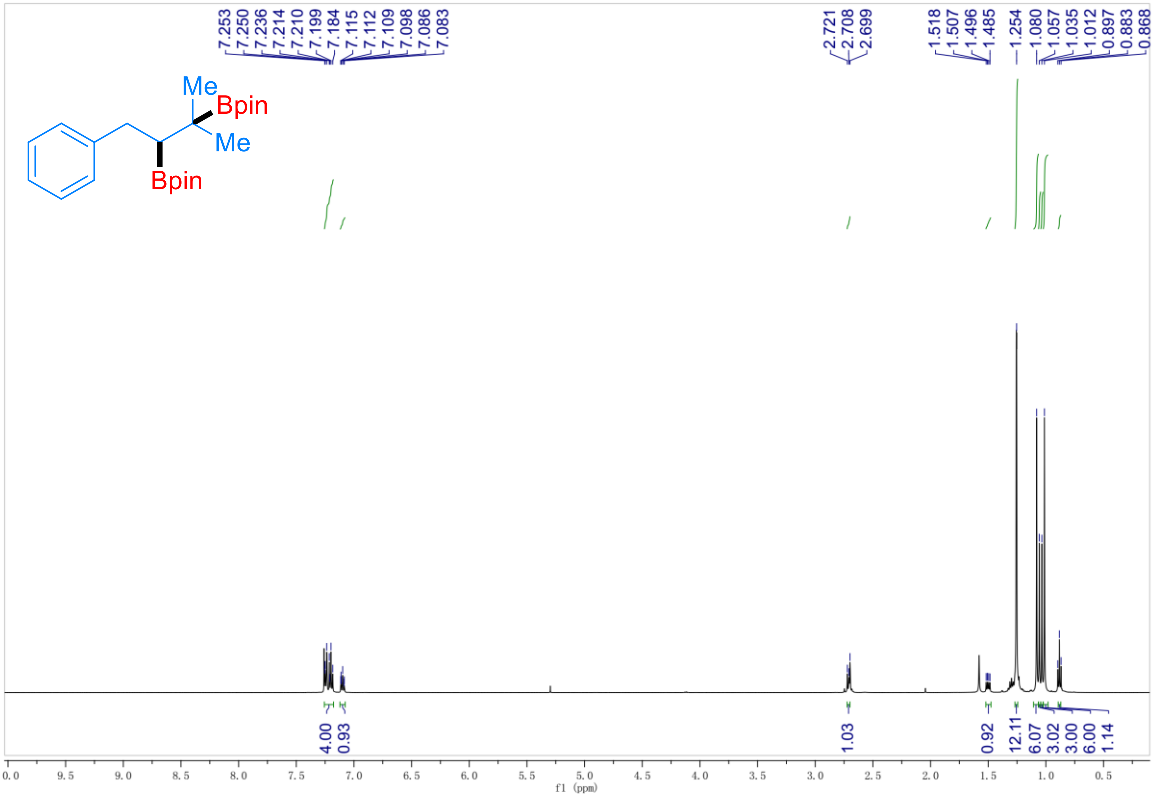

^{1}H NMR spectrum of compound **53b** in $CDCl_3$ (500 MHz).

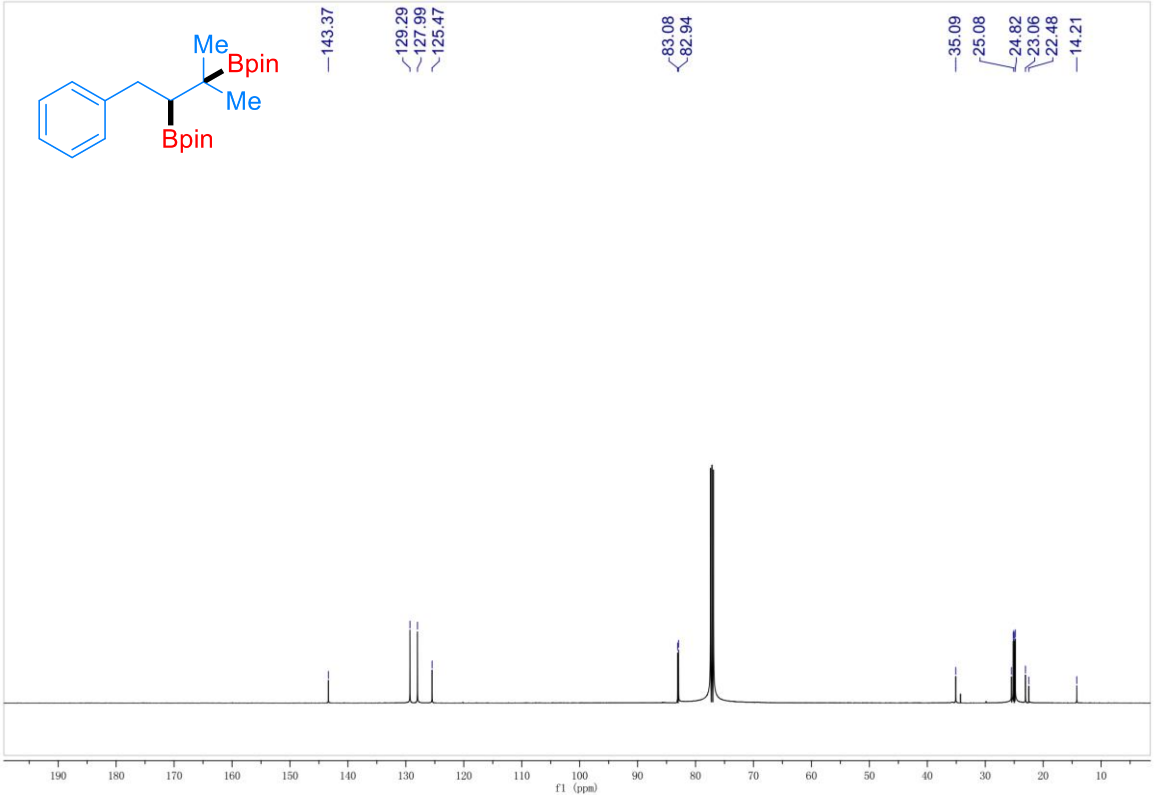

$^{13}C\{^1H\}$ NMR spectrum of compound **53b** in $CDCl_3$ (125 MHz).

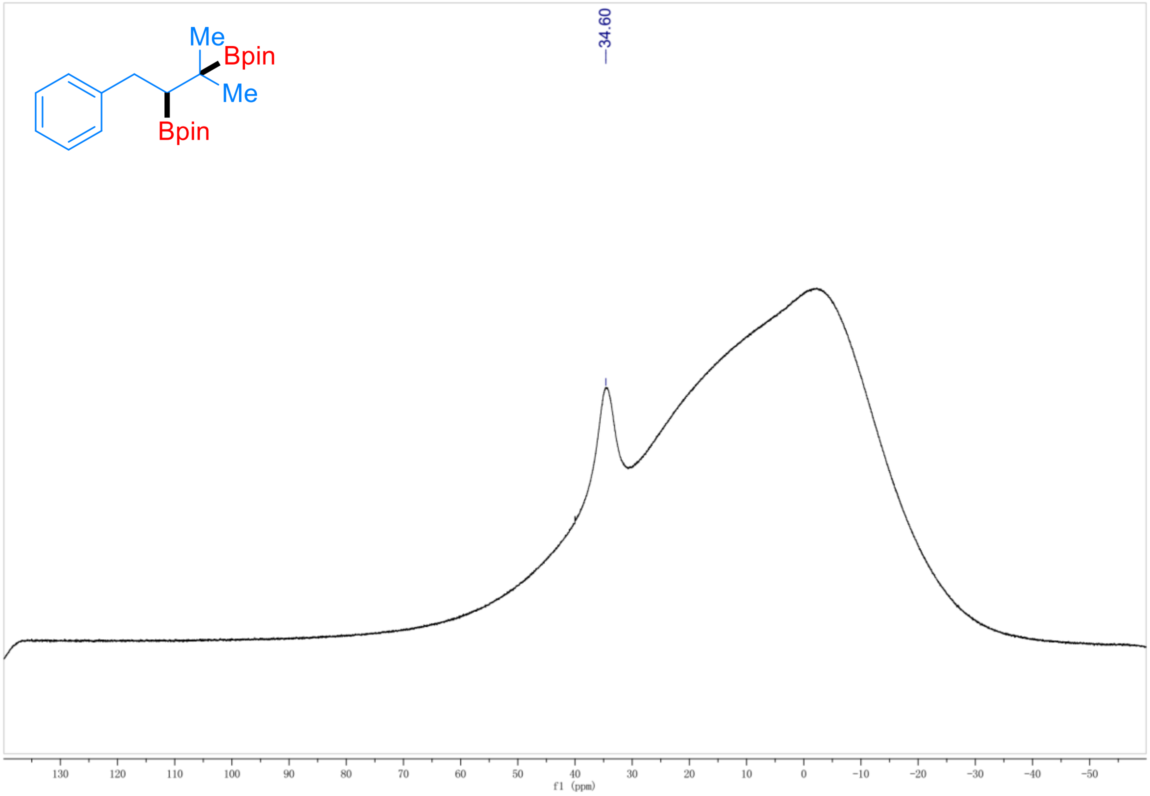

$^{11}B\{^1H\}$ NMR spectrum of compound **53b** in $CDCl_3$ (160 MHz).

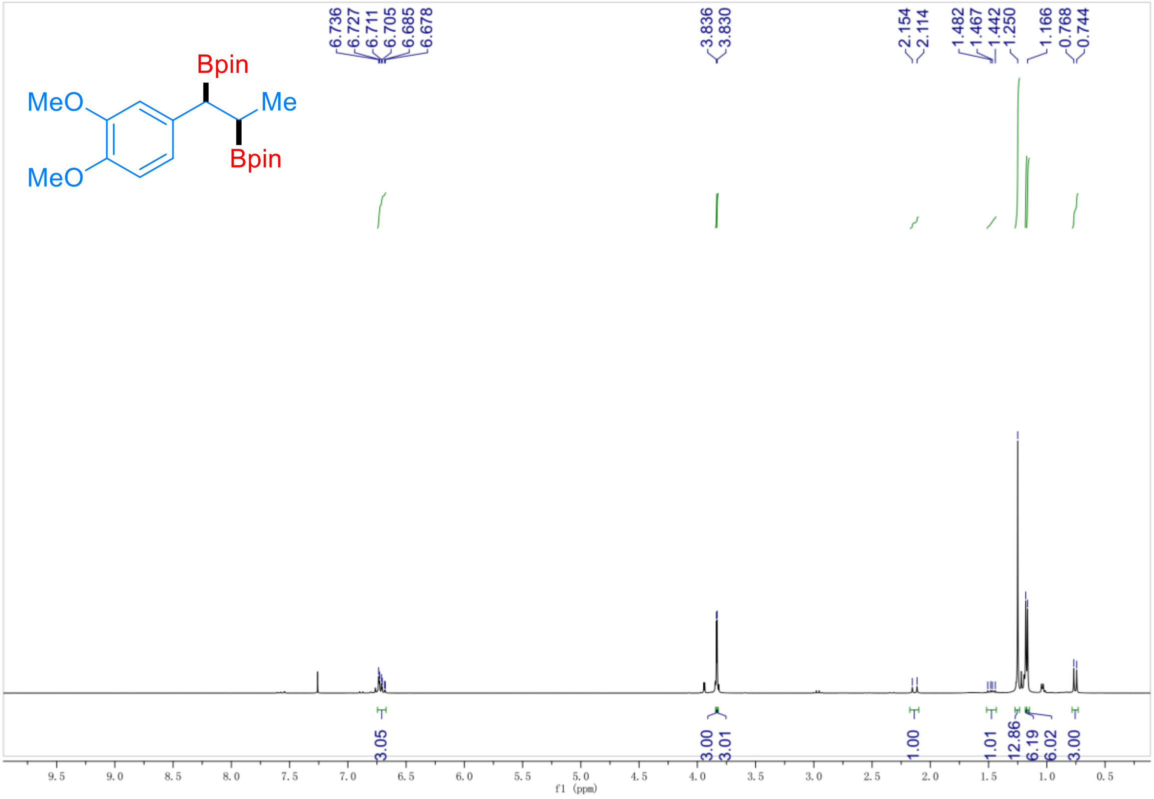

^{1}H NMR spectrum of compound **55b** in $CDCl_3$ (300 MHz).

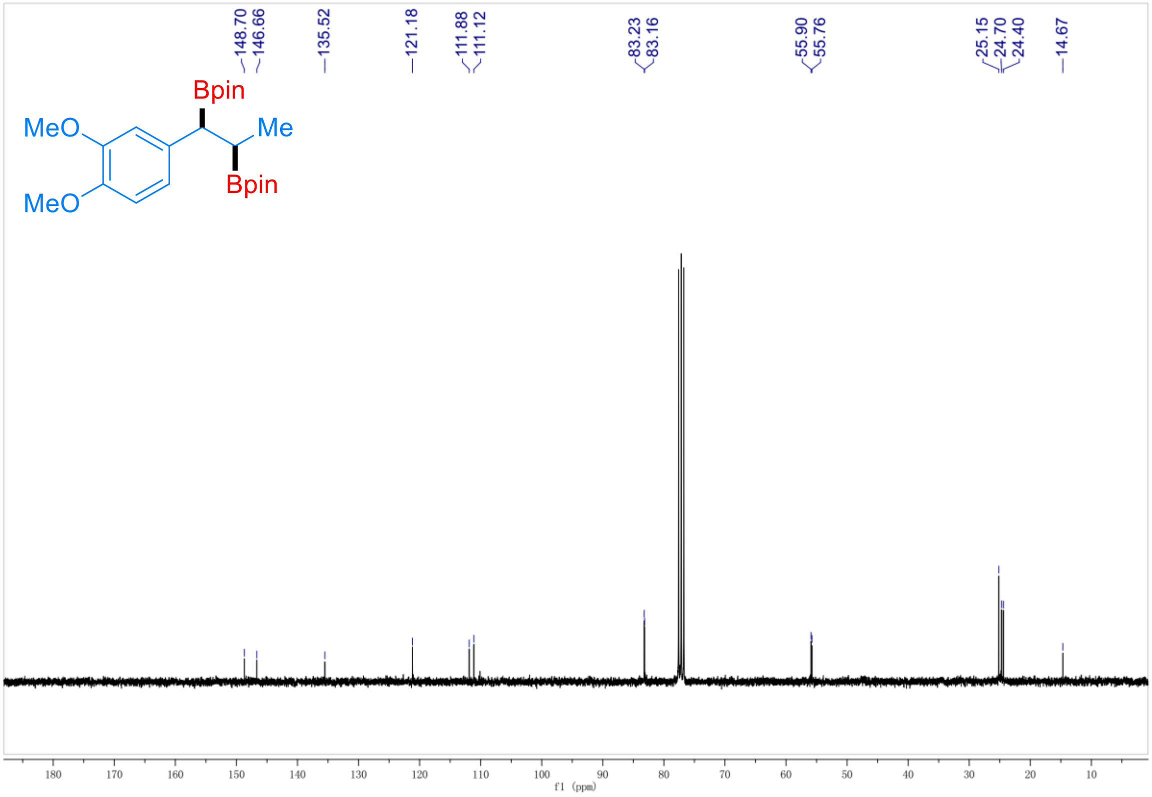

^{13}C{^{1}H} NMR spectrum of compound **55b** in $CDCl_3$ (75 MHz).

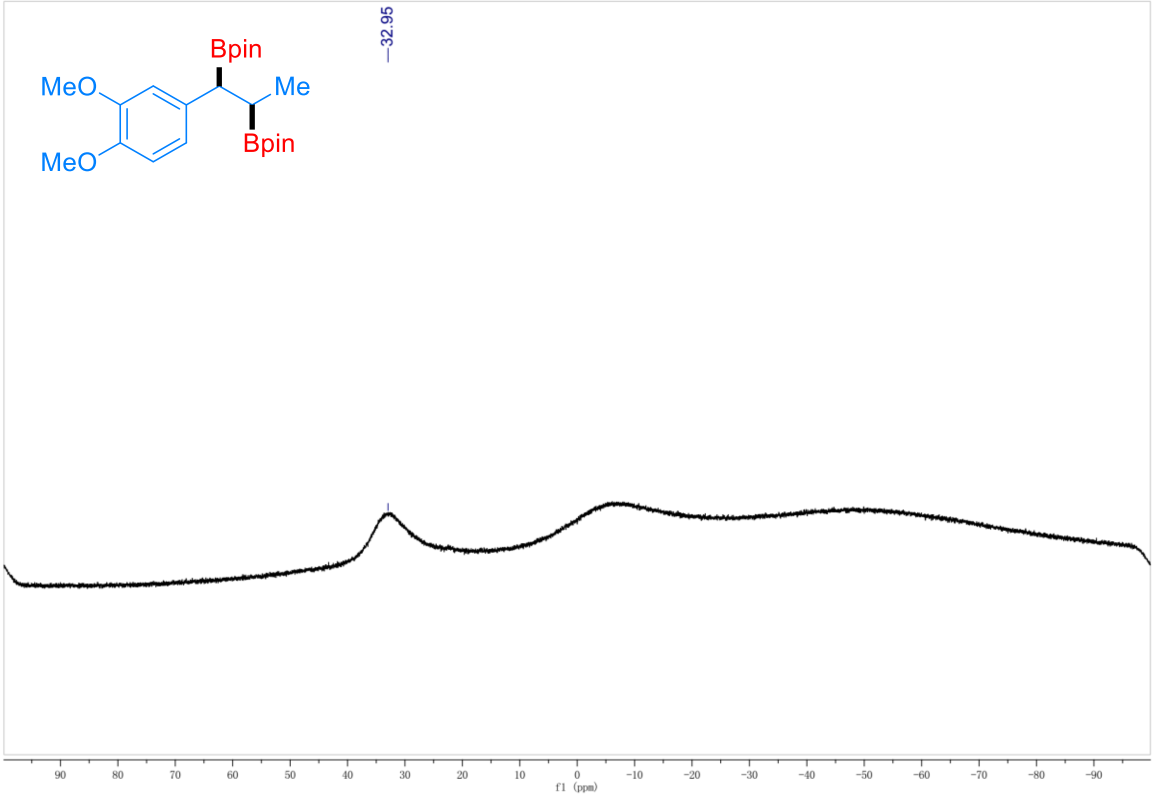

^{11}B{^{1}H} NMR spectrum of compound **55b** in $CDCl_3$ (96 MHz).

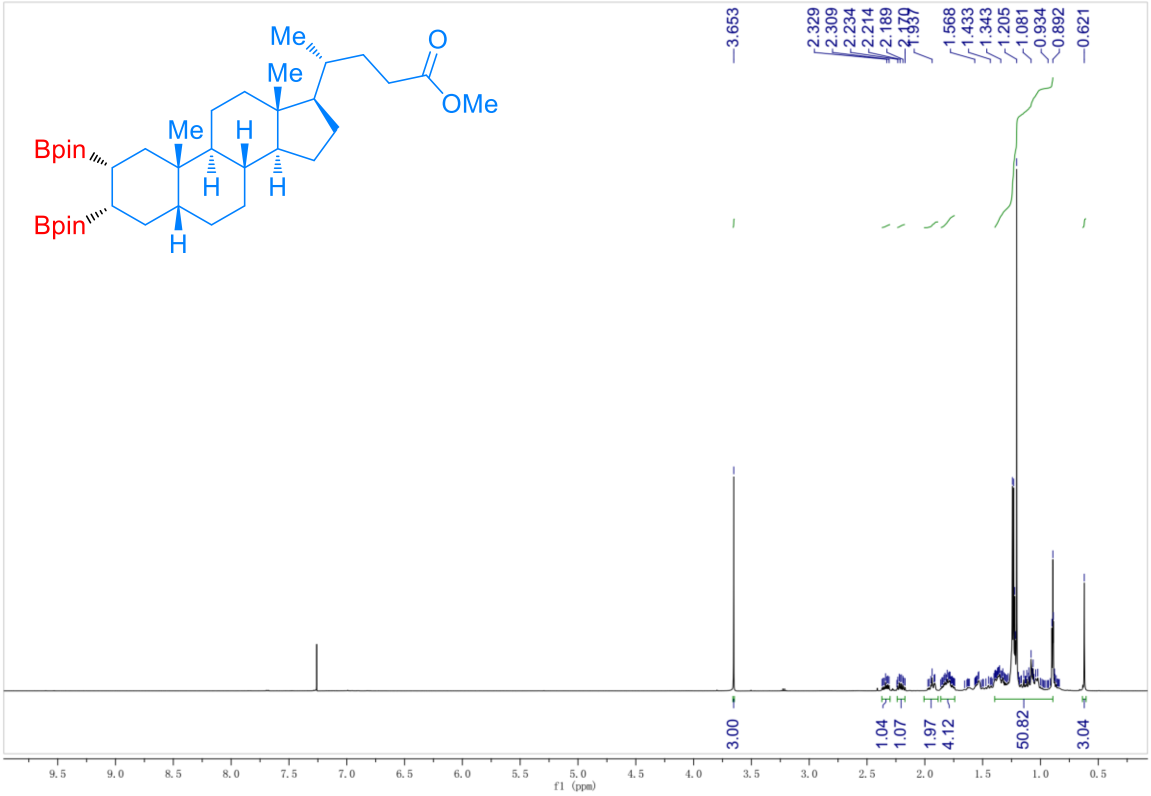

^{1}H NMR spectrum of compound **56b** in $CDCl_3$ (500 MHz).

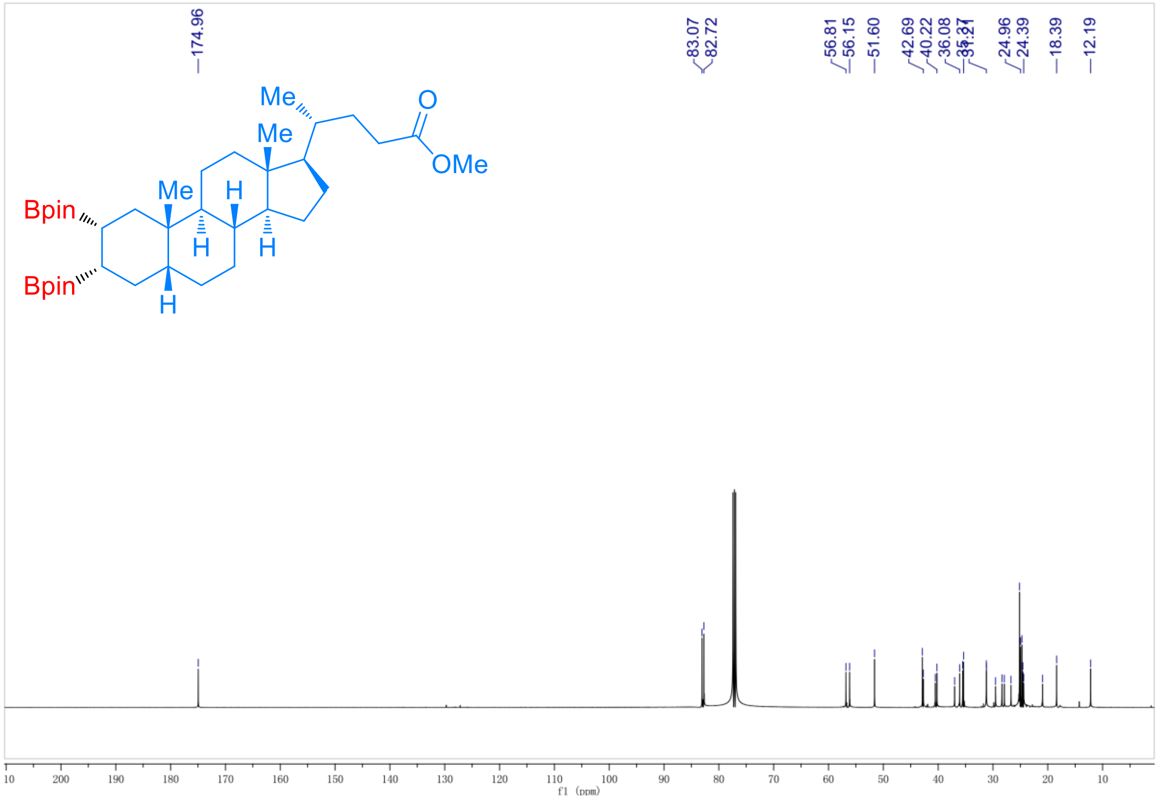

$^{13}C\{^{1}H\}$ NMR spectrum of compound **56b** in $CDCl_3$ (125 MHz).

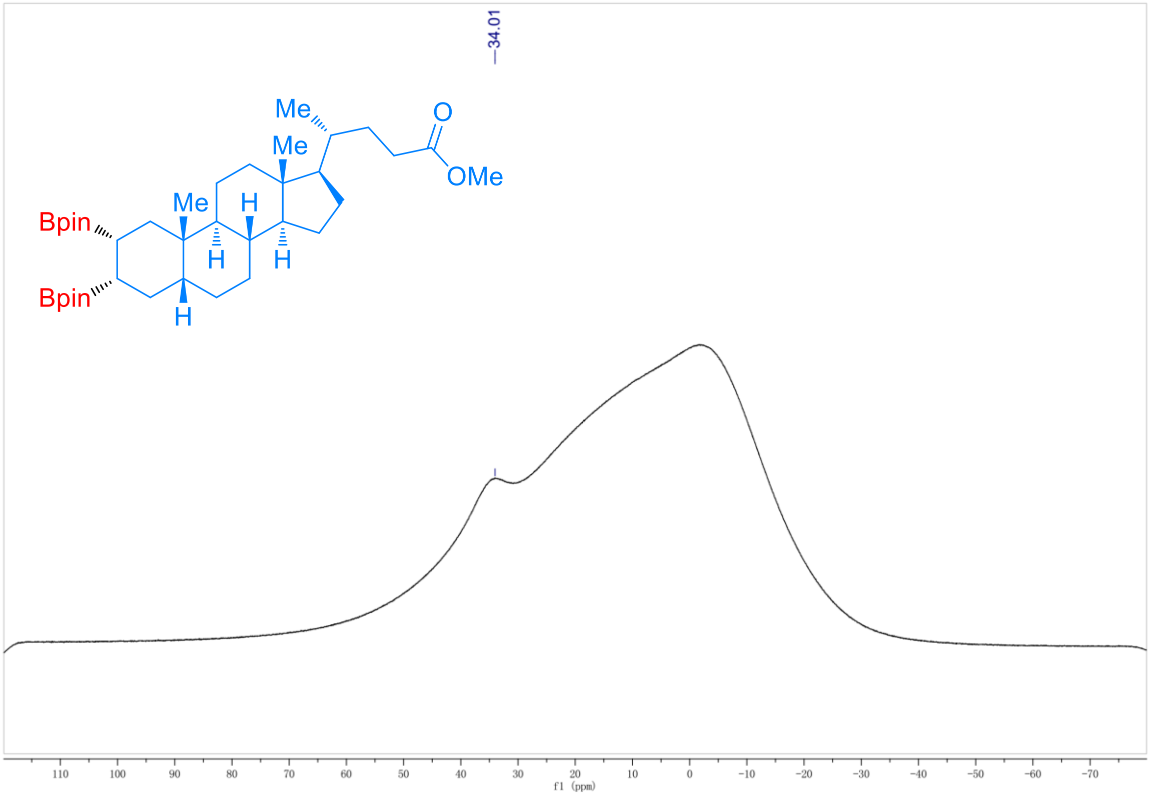

$^{11}B\{^{1}H\}$ NMR spectrum of compound **56b** in $CDCl_3$ (160 MHz).

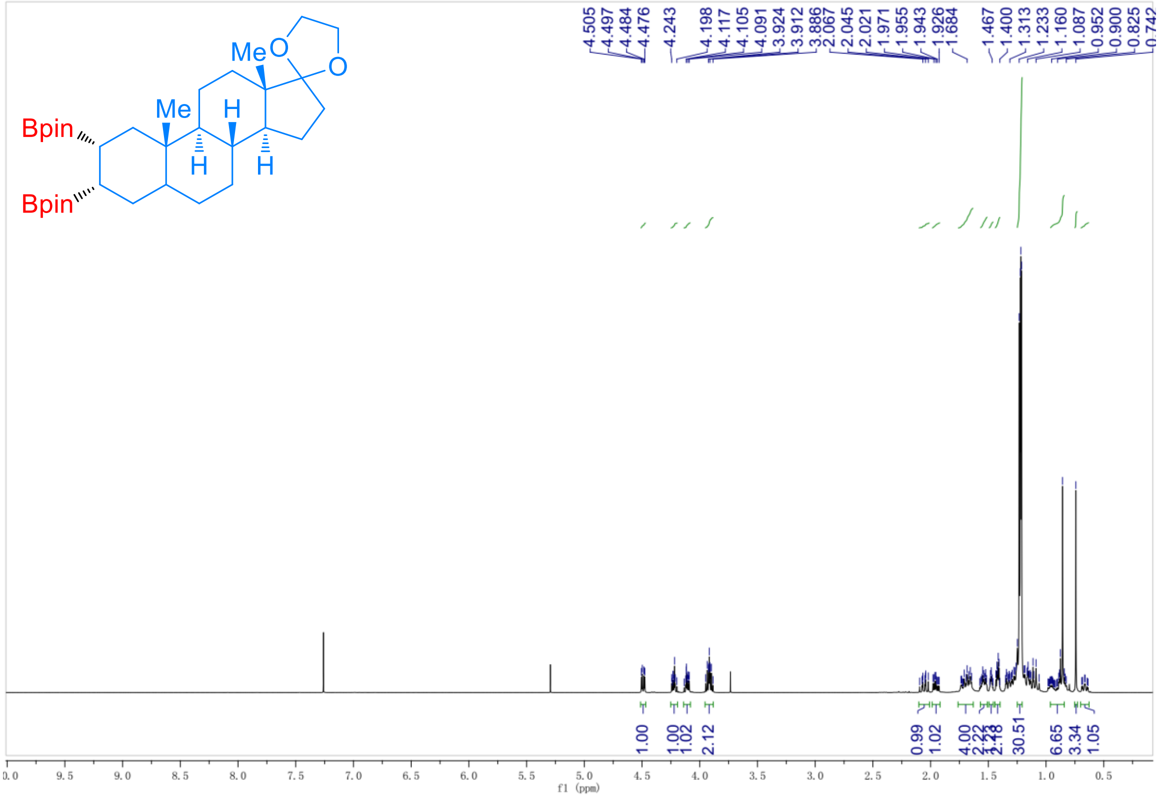

^{1}H NMR spectrum of compound **57b** in $CDCl_3$ (500 MHz).

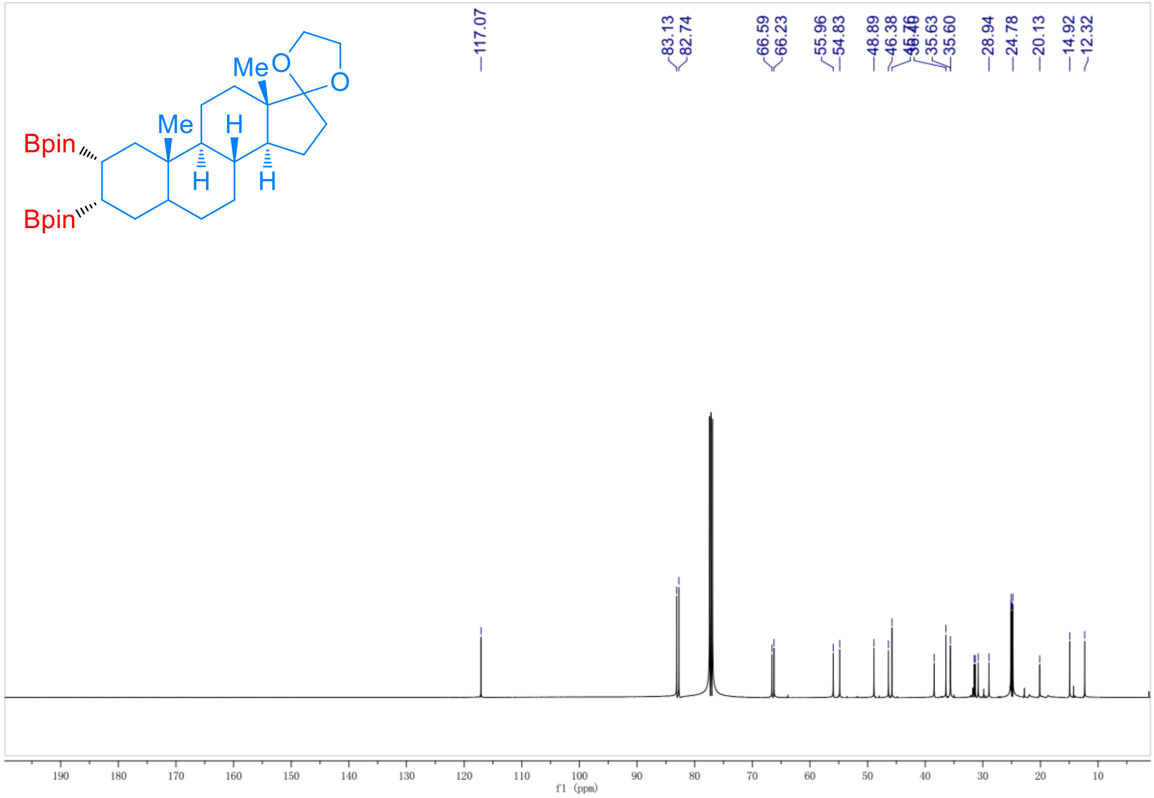

^{13}C{^{1}H} NMR spectrum of compound **57b** in $CDCl_3$ (125 MHz).

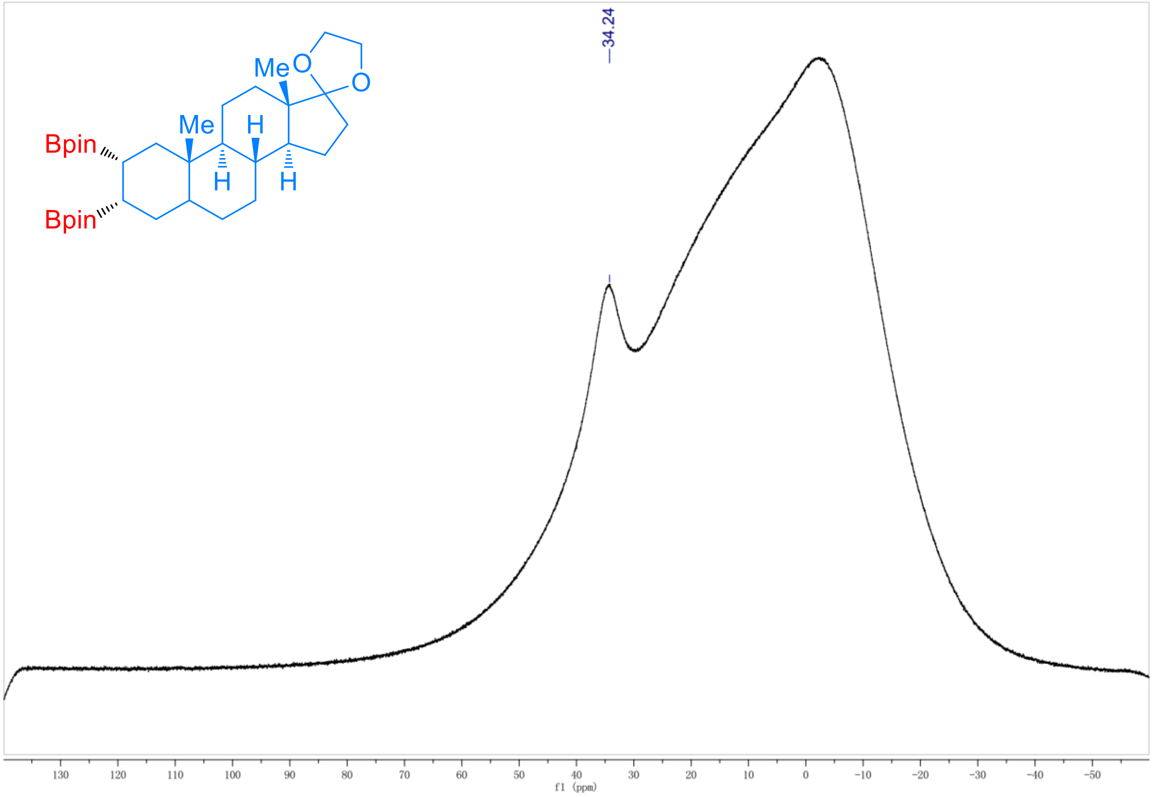

11B{1H} NMR spectrum of compound **57b** in $CDCl_3$ (128 MHz).

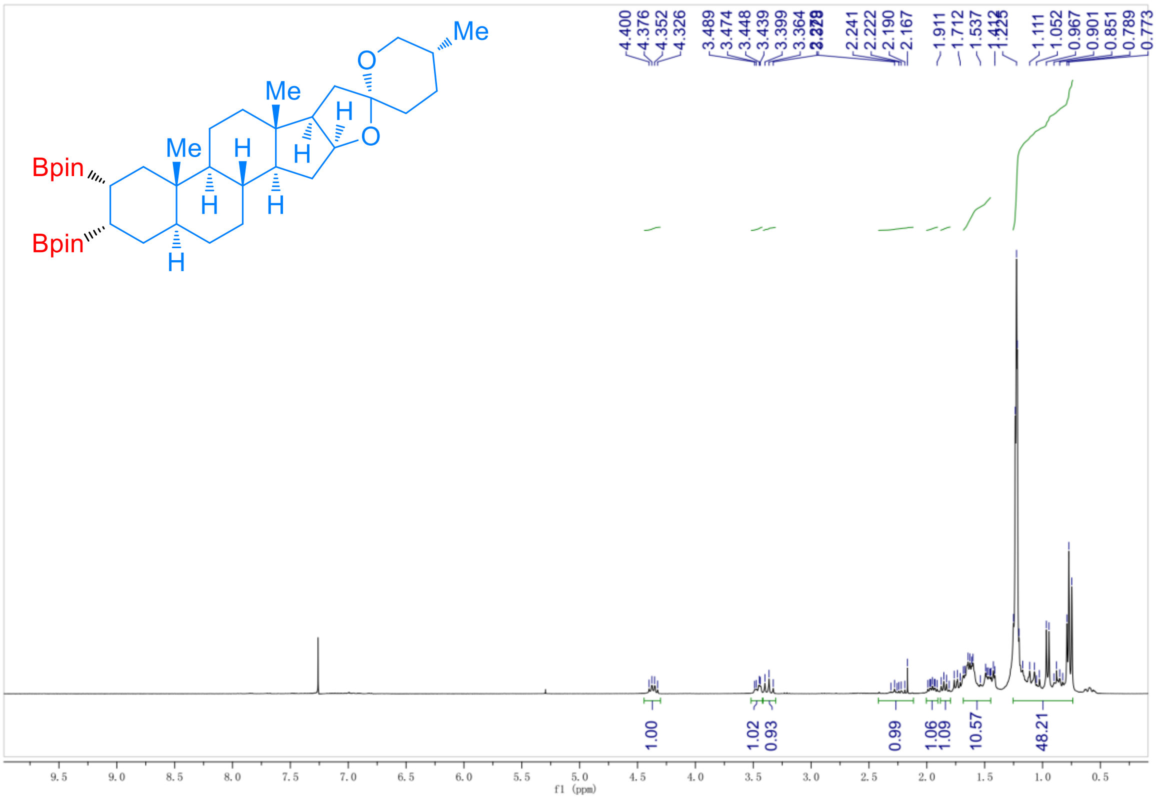

1H NMR spectrum of compound **58b** in $CDCl_3$ (400 MHz).

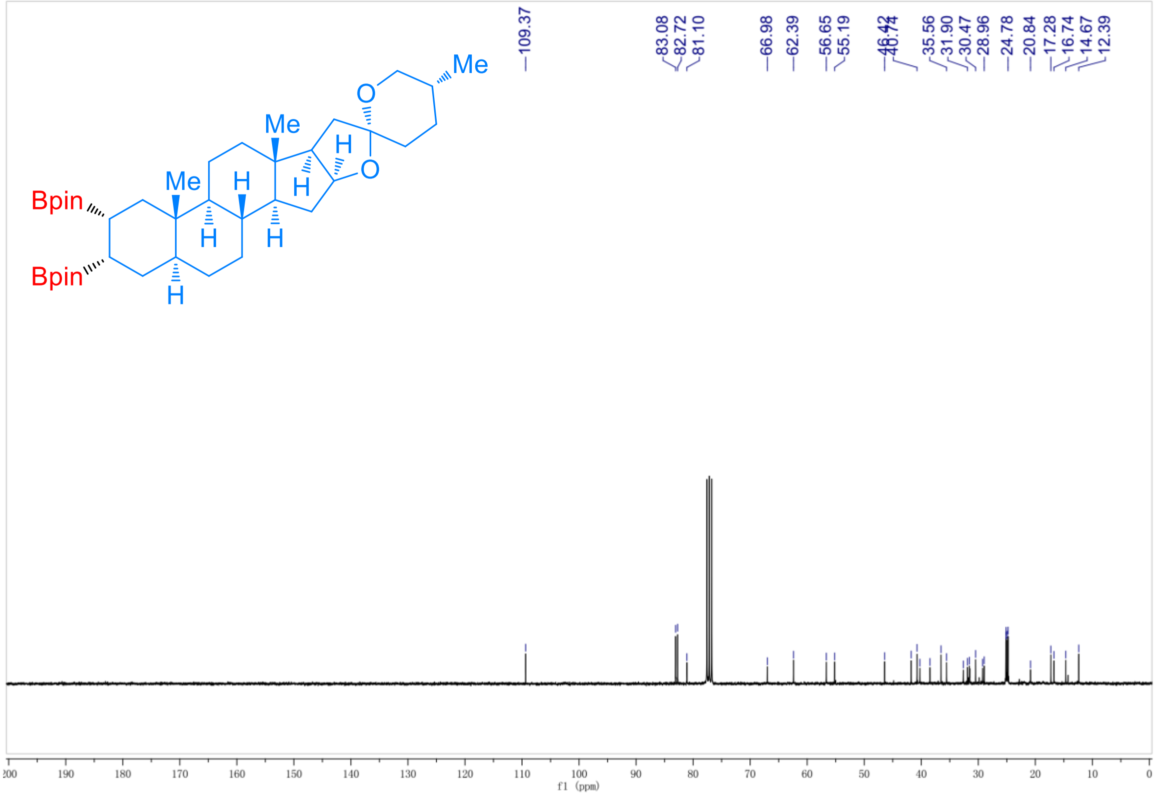

$^{13}C\{^{1}H\}$ NMR spectrum of compound **58b** in $CDCl_3$ (100 MHz).

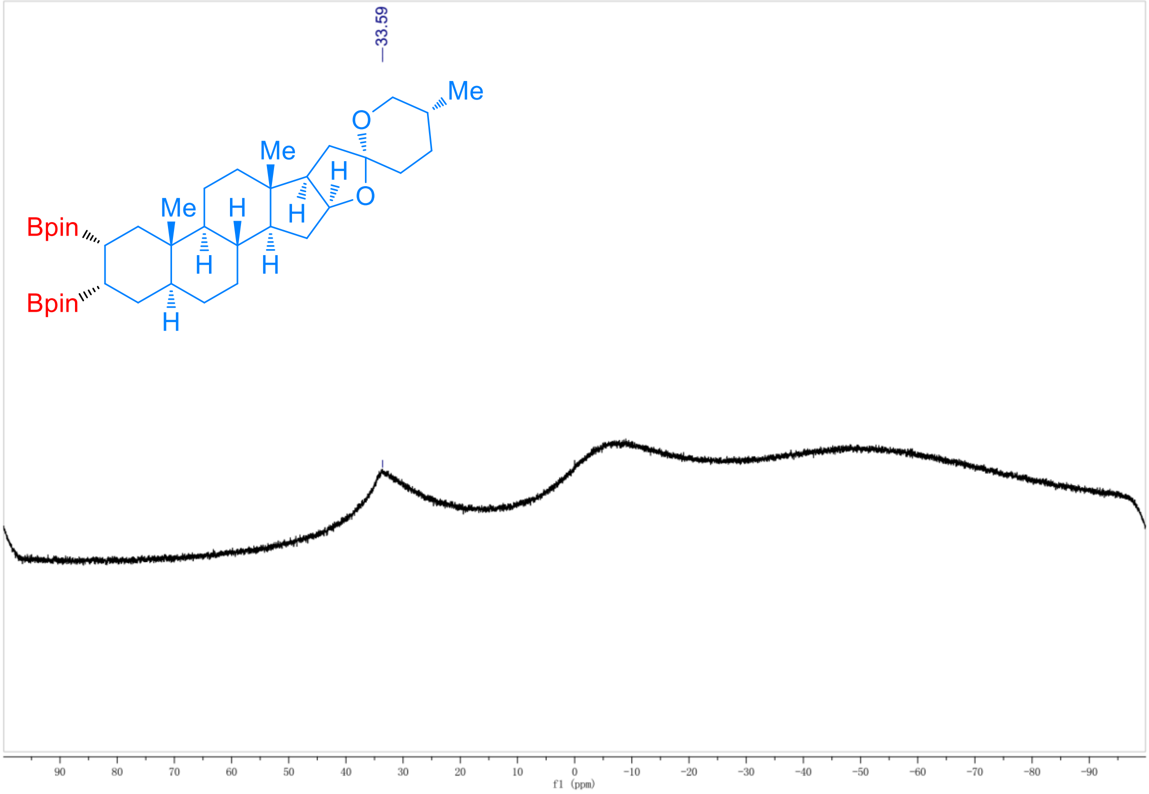

$^{11}B\{^{1}H\}$ NMR spectrum of compound **58b** in $CDCl_3$ (128 MHz).

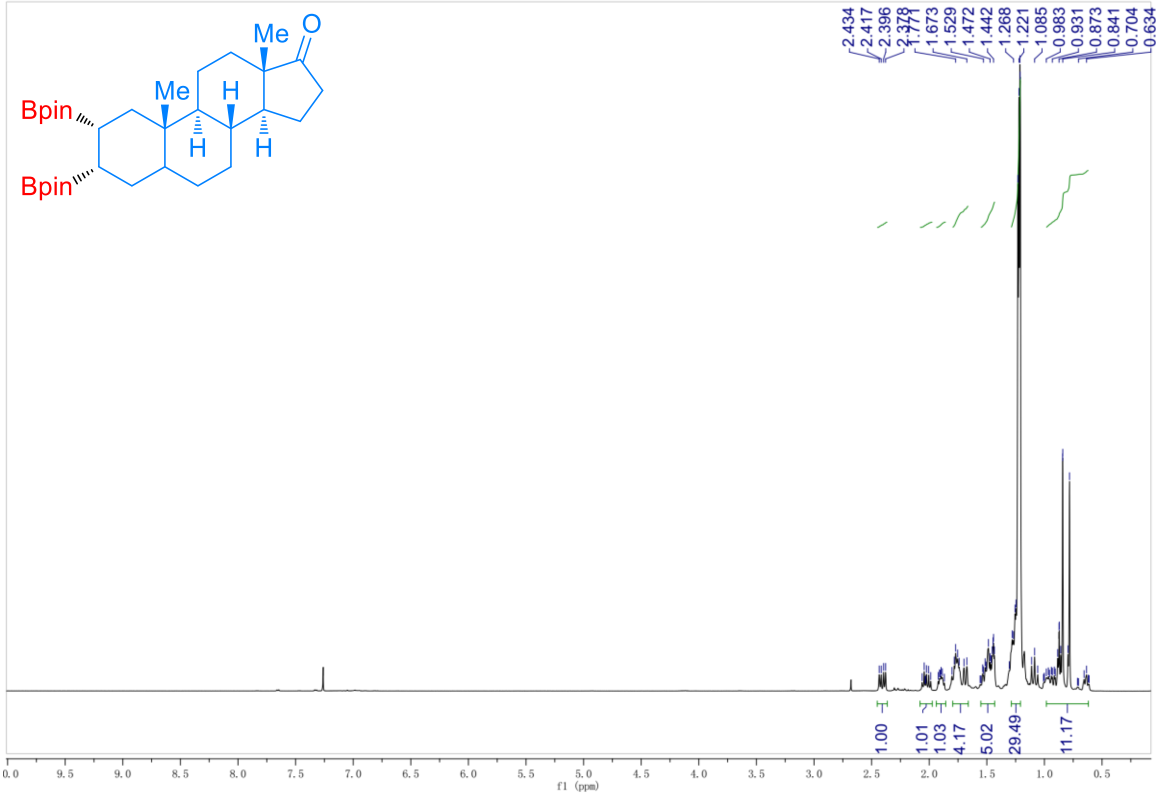

^{1}H NMR spectrum of compound **59b** in $CDCl_3$ (500 MHz).

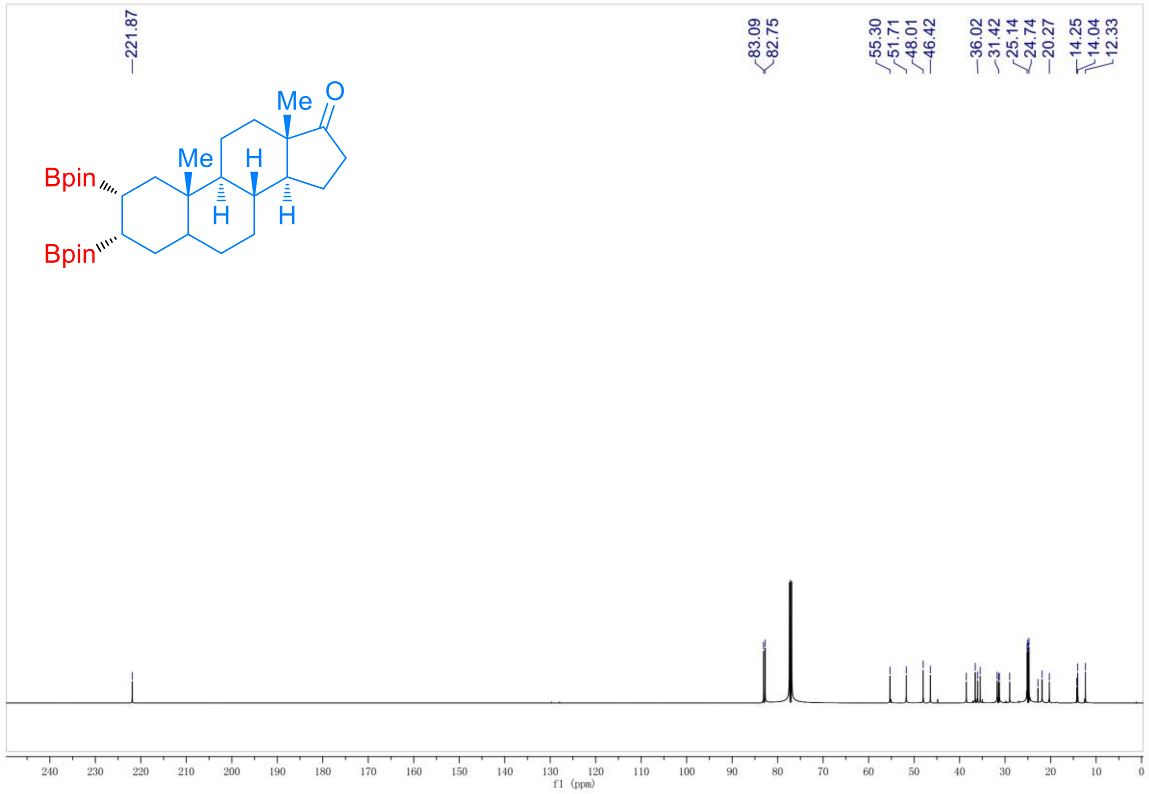

^{13}C{^{1}H} NMR spectrum of compound **59b** in $CDCl_3$ (125 MHz).

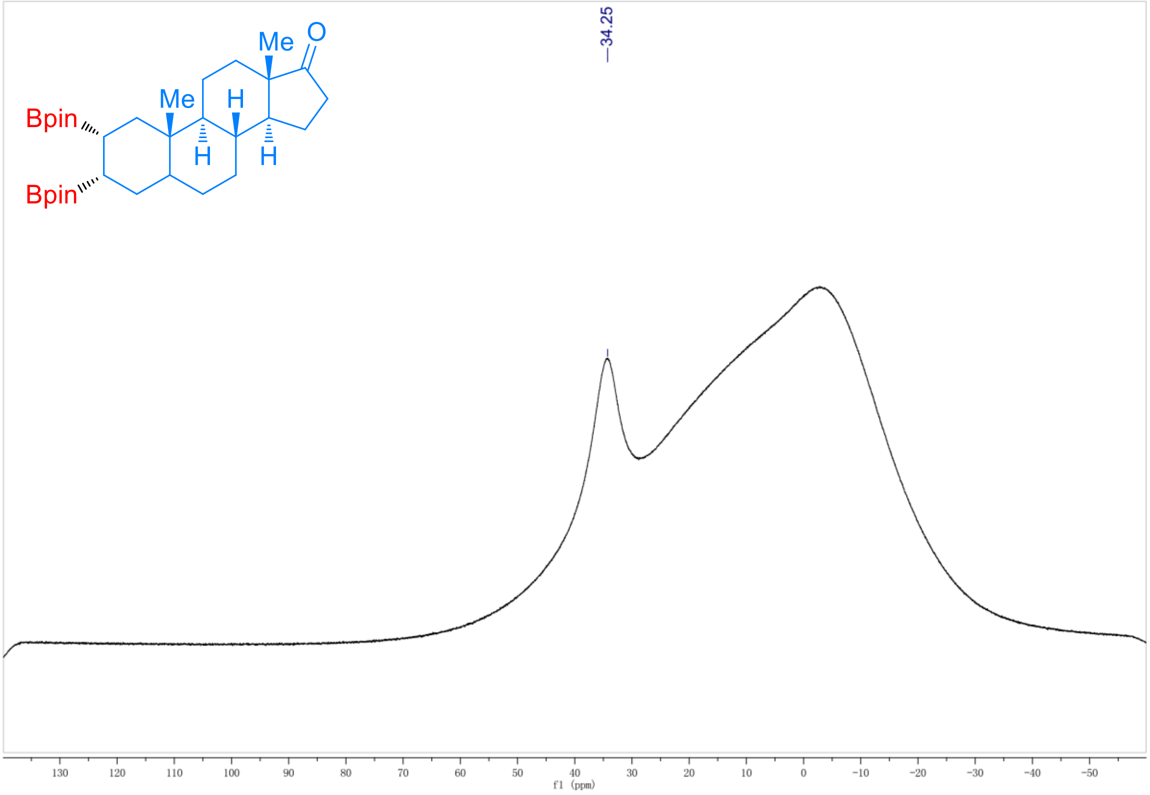

^{11}B{^{1}H} NMR spectrum of compound **59b** in $CDCl_3$ (160 MHz).

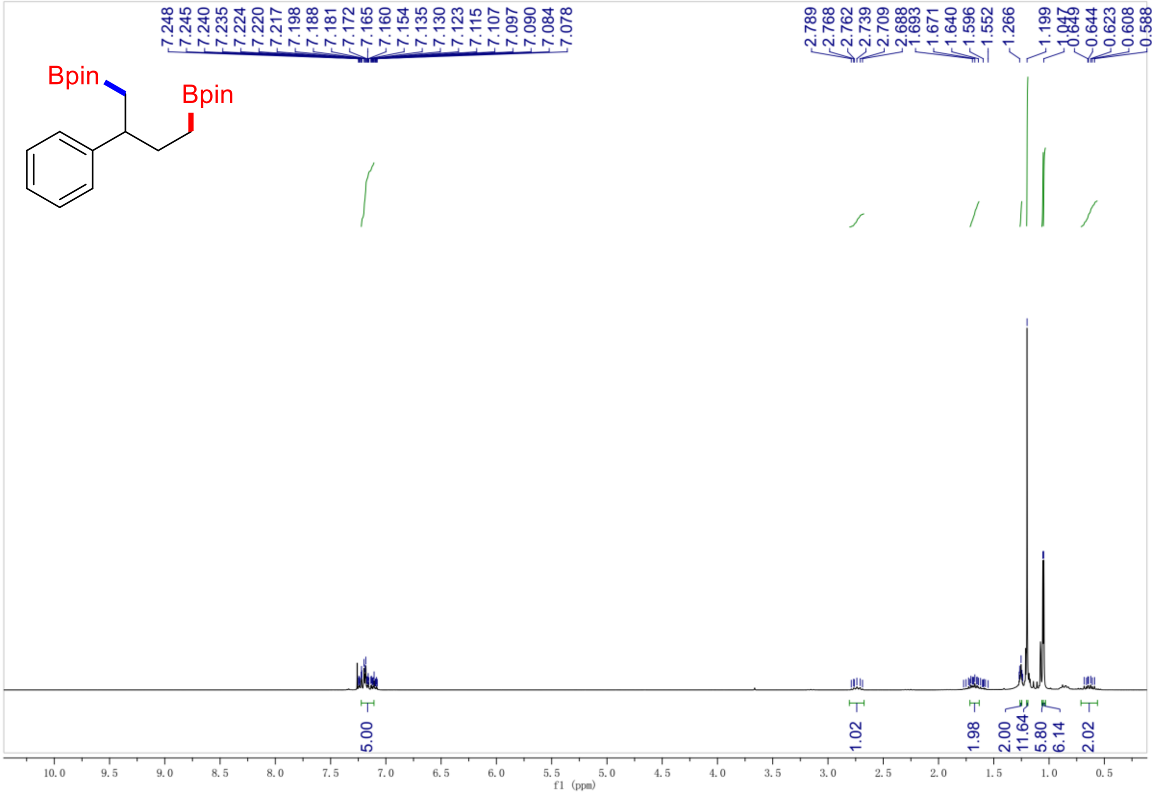

^{1}H NMR spectrum of compound **60** in $CDCl_3$ (300 MHz).

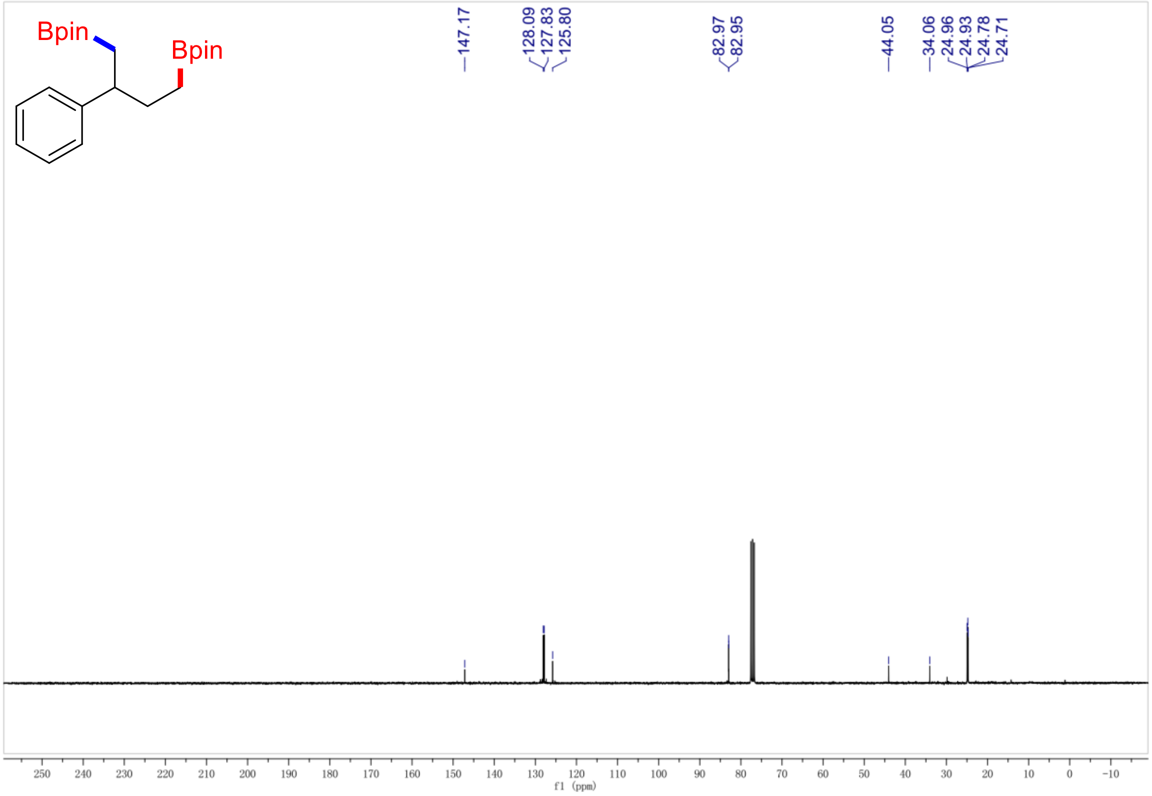

$^{13}C\{^{1}H\}$ NMR spectrum of compound **60** in $CDCl_3$ (75 MHz).

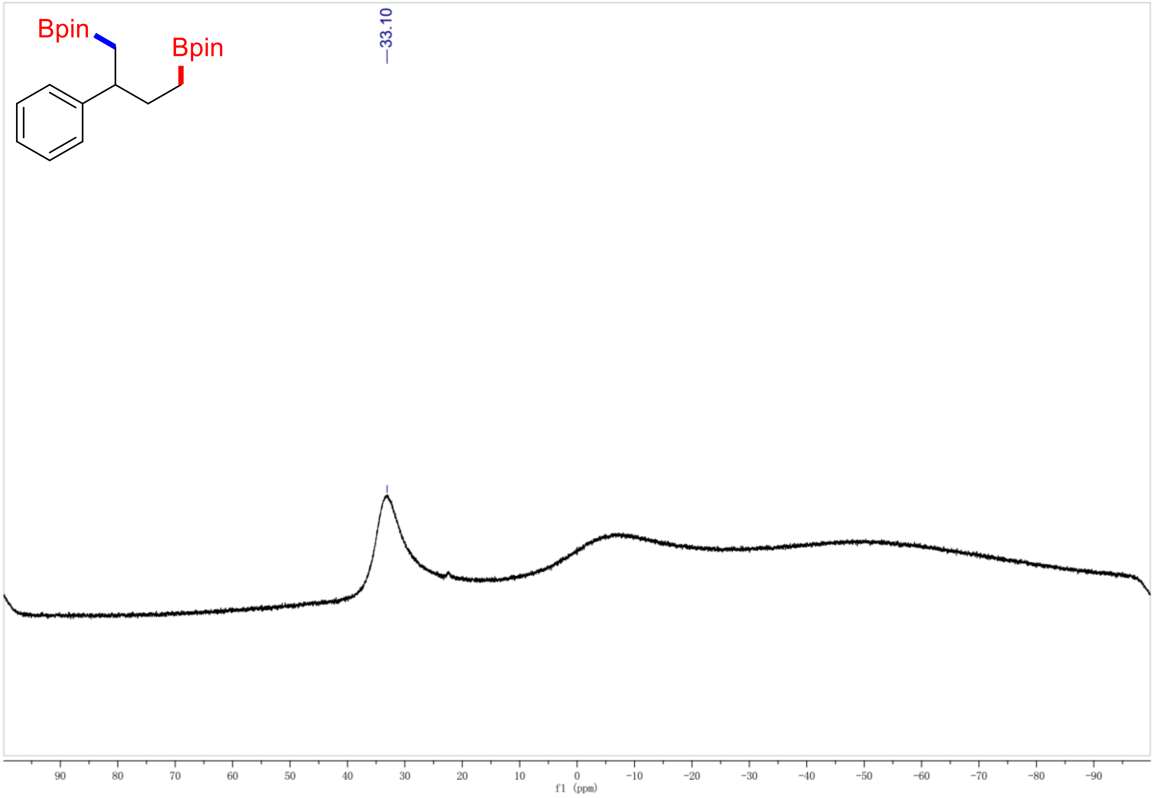

$^{11}B\{^{1}H\}$ NMR spectrum of compound **60** in $CDCl_3$ (96 MHz).

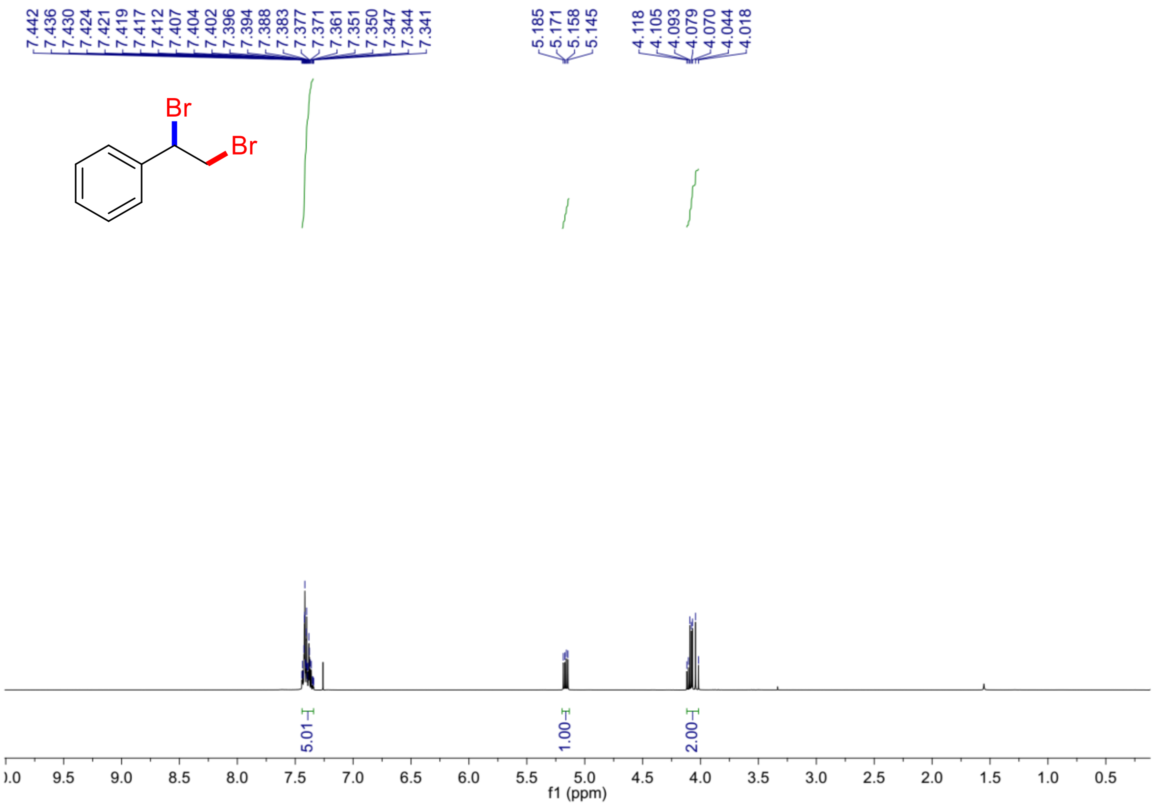

^{1}H NMR spectrum of compound **61** in $CDCl_3$ (400 MHz).

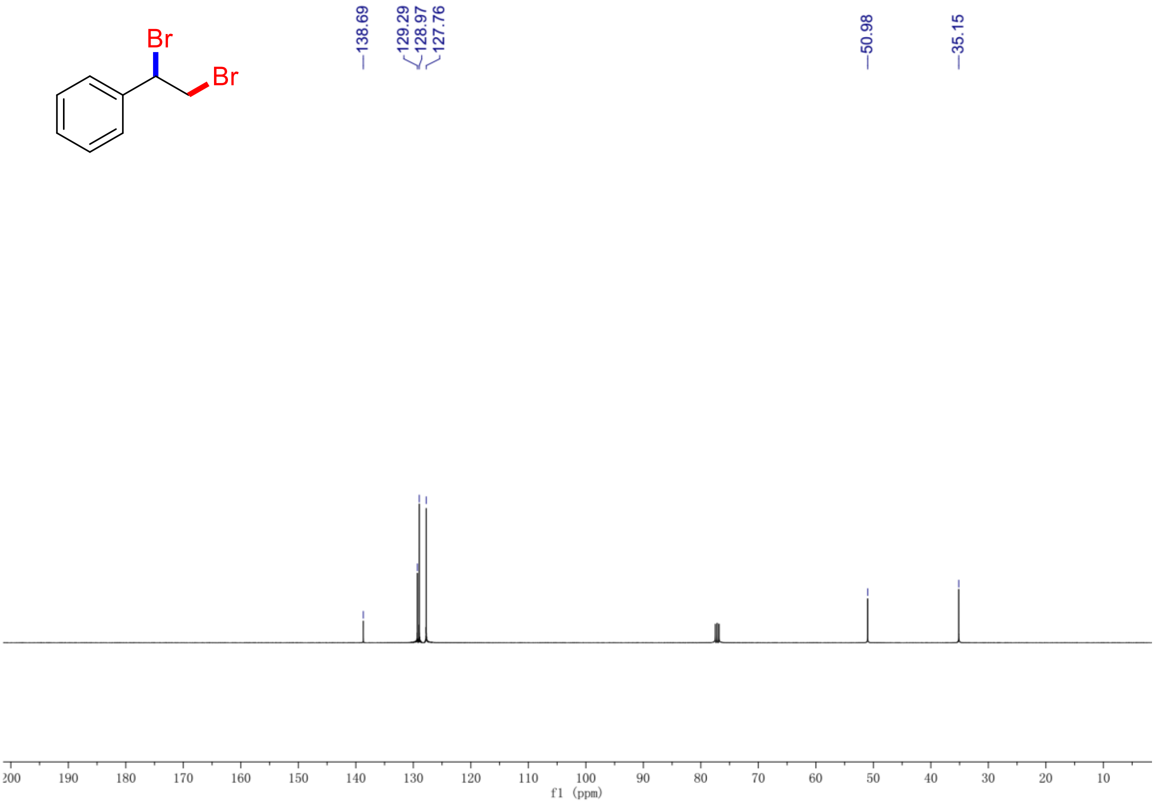

^{13}C{^{1}H} NMR spectrum of compound **61** in $CDCl_3$ (100 MHz).

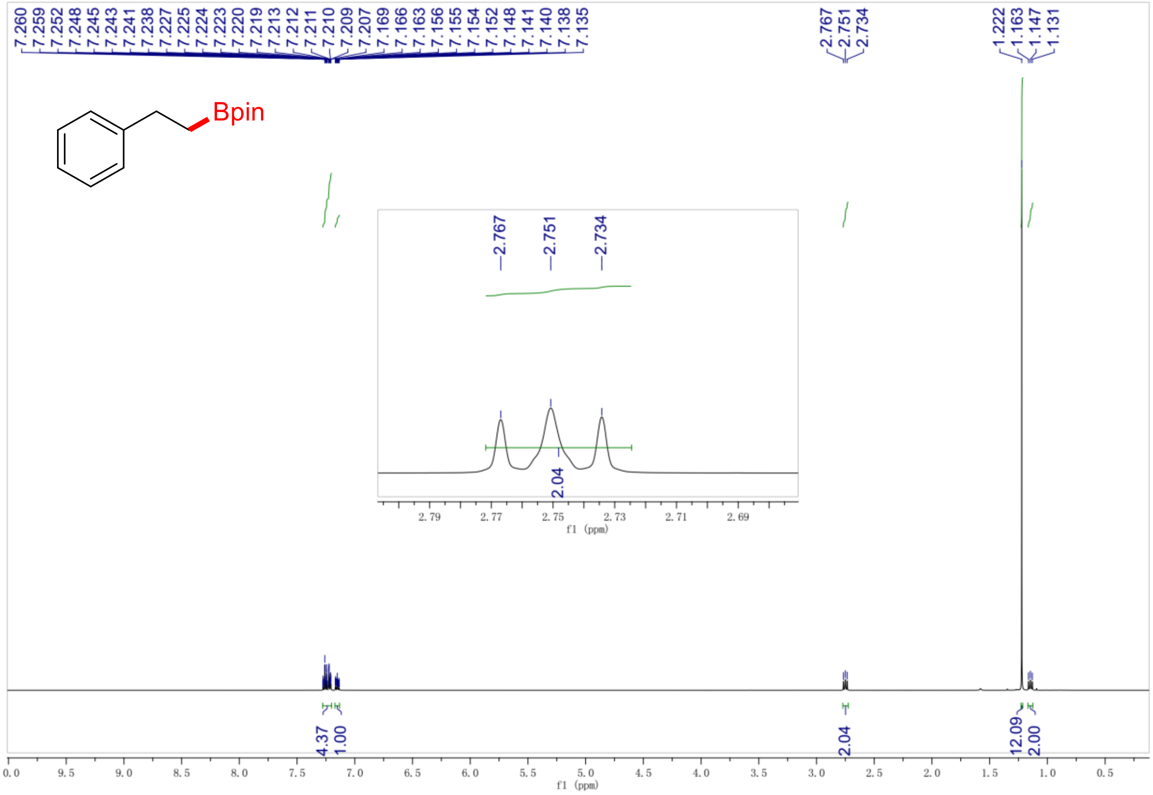

^{1}H NMR spectrum of compound **62** in $CDCl_3$ (500 MHz).

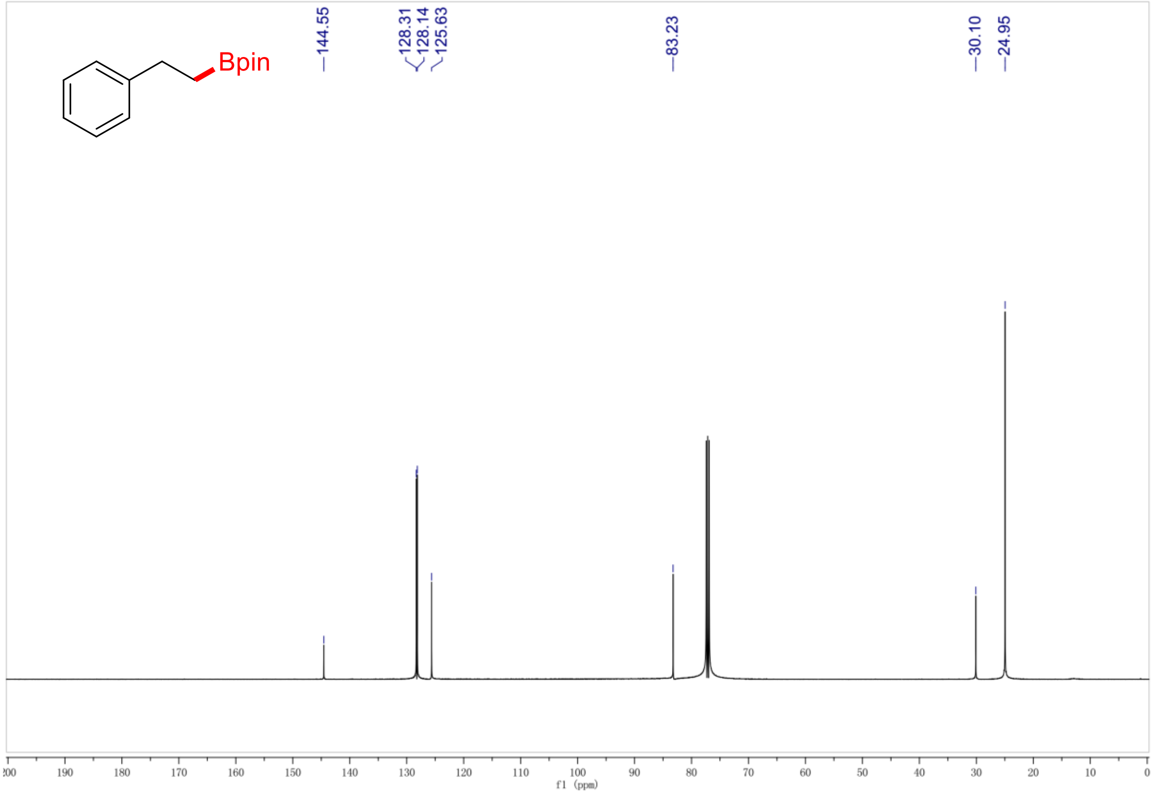

^{13}C{^{1}H} NMR spectrum of compound **62** in $CDCl_3$ (125 MHz).

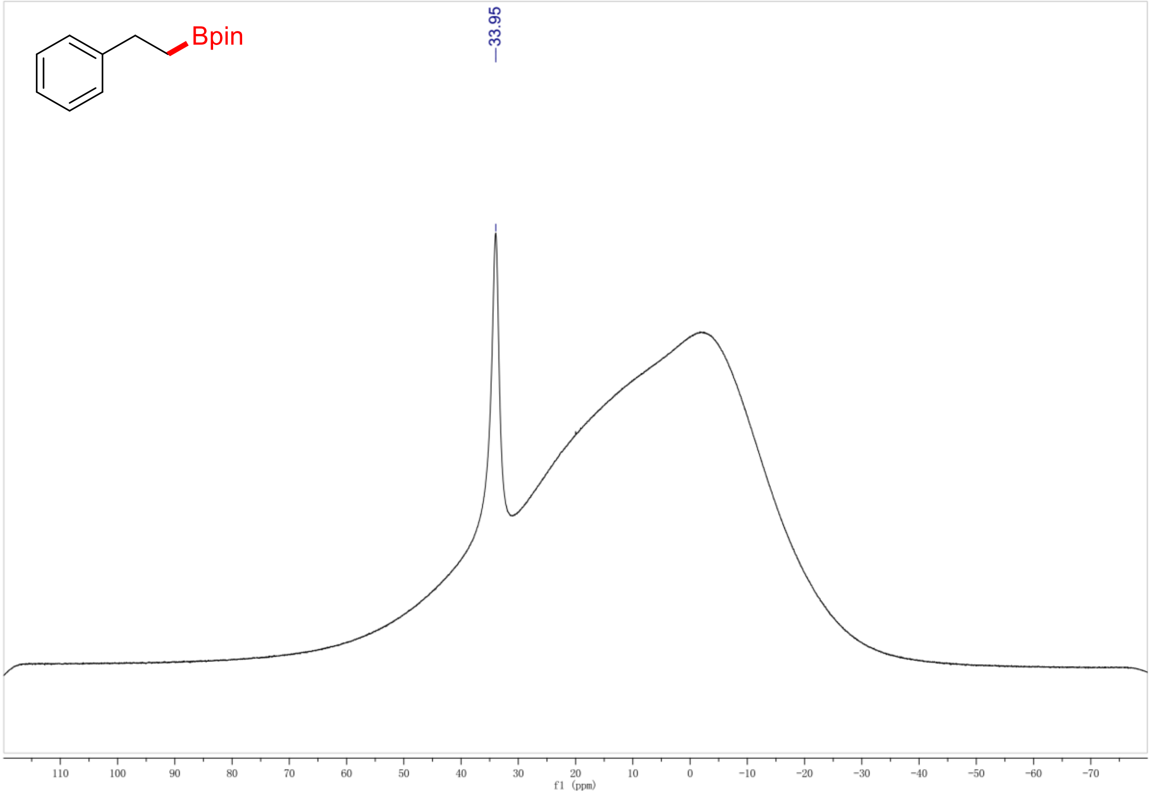

$^{11}B\{^{1}H\}$ NMR spectrum of compound **62** in $CDCl_3$ (160 MHz).

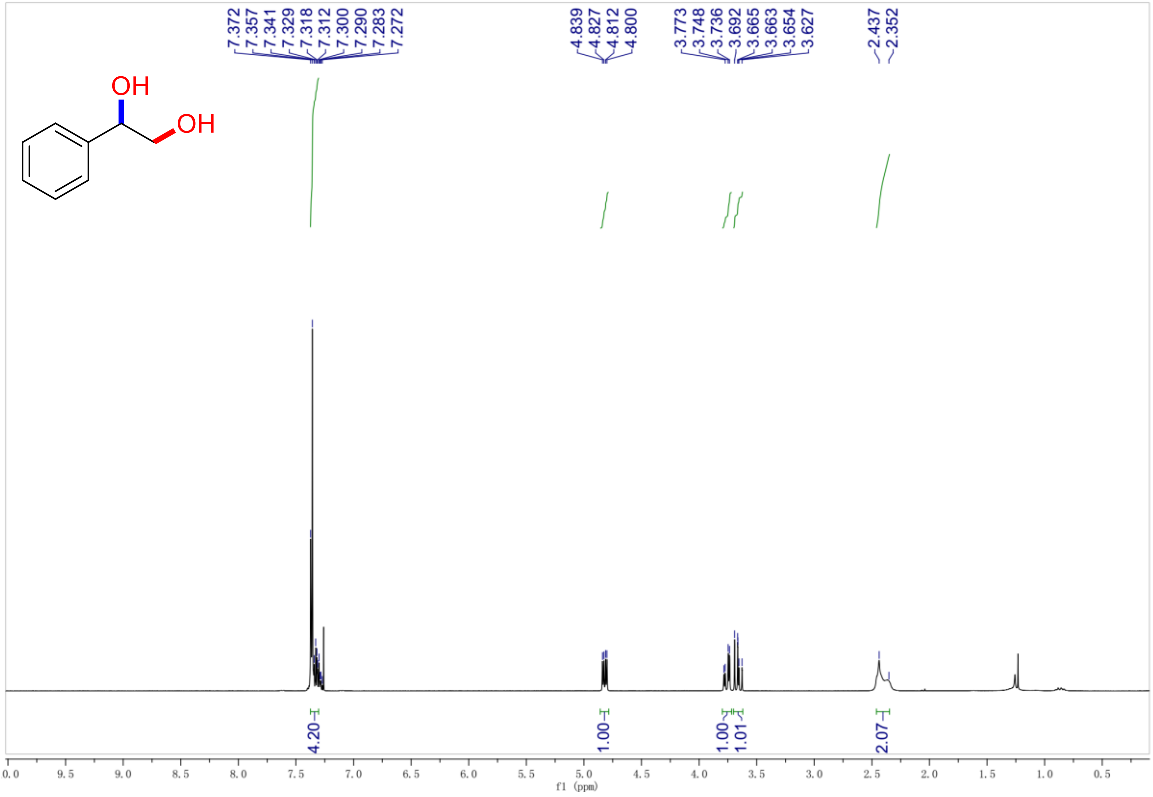

^{1}H NMR spectrum of compound **63** in $CDCl_3$ (300 MHz).

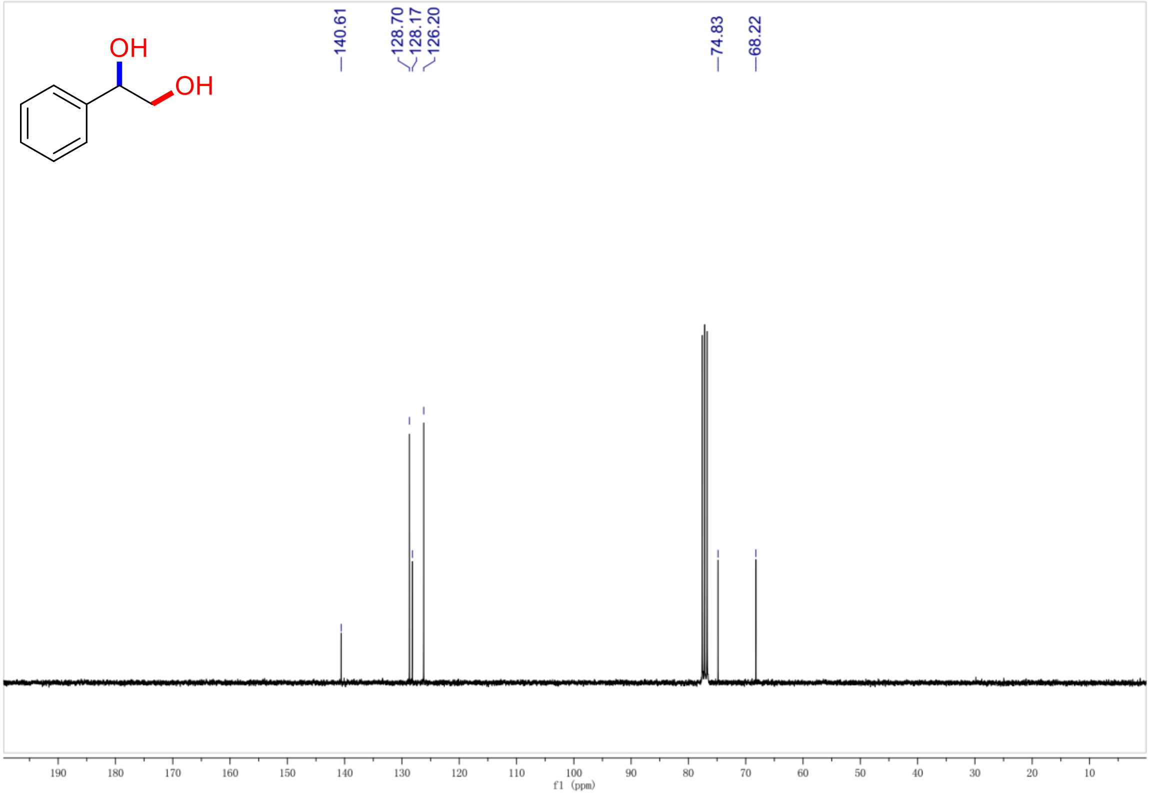

$^{13}C\{^{1}H\}$ NMR spectrum of compound **63** in $CDCl_3$ (75 MHz).

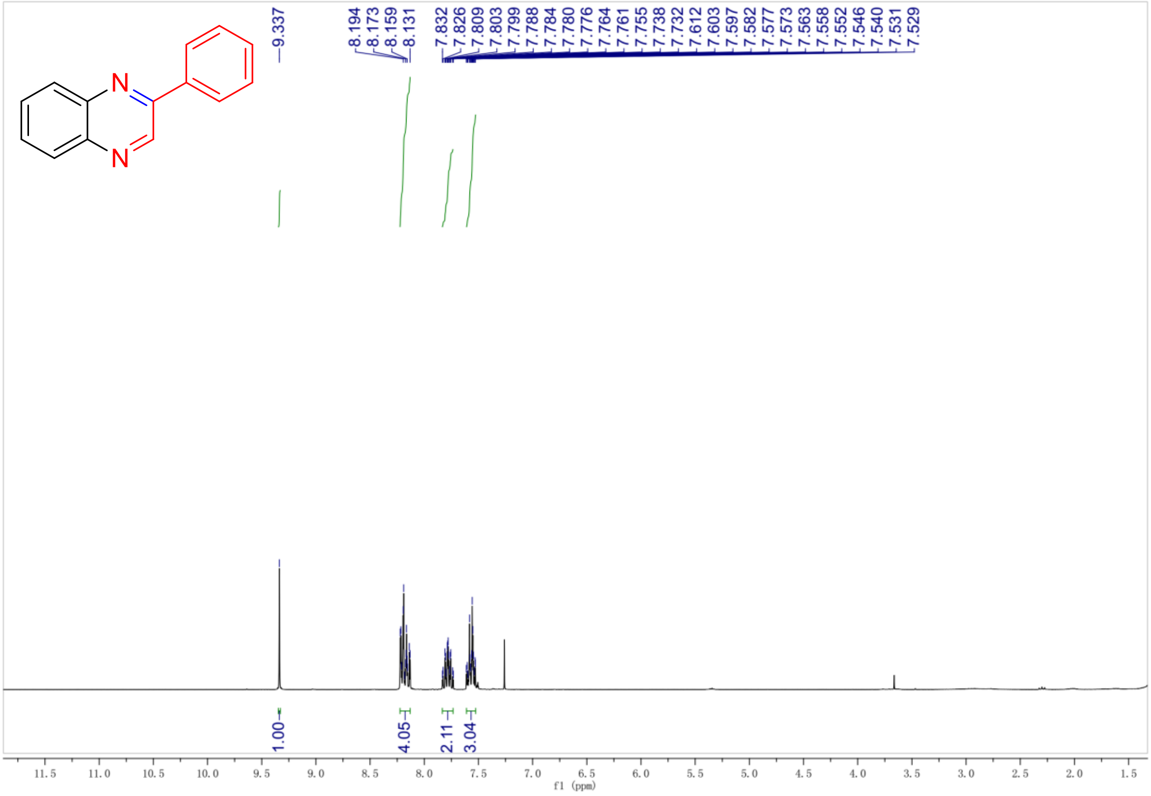

^{1}H NMR spectrum of compound **64** in $CDCl_3$ (300 MHz).

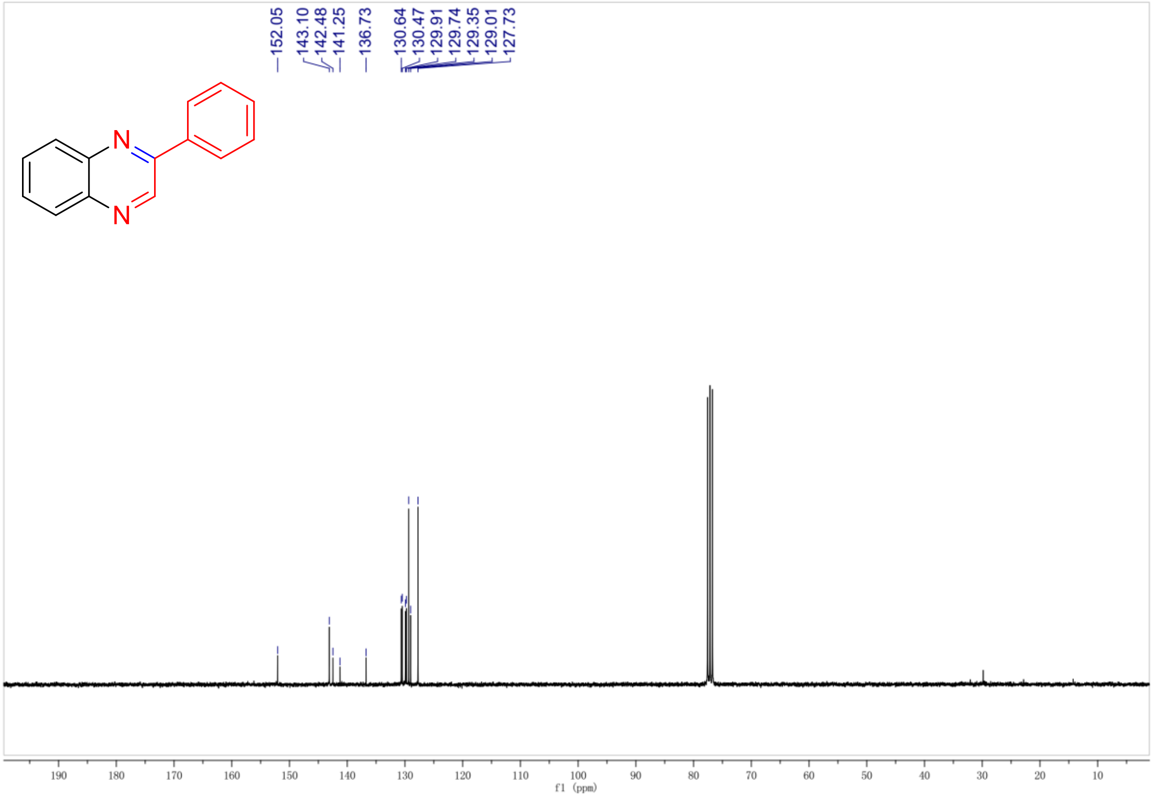

$^{13}C\{^{1}H\}$ NMR spectrum of compound **64** in $CDCl_3$ (75 MHz).

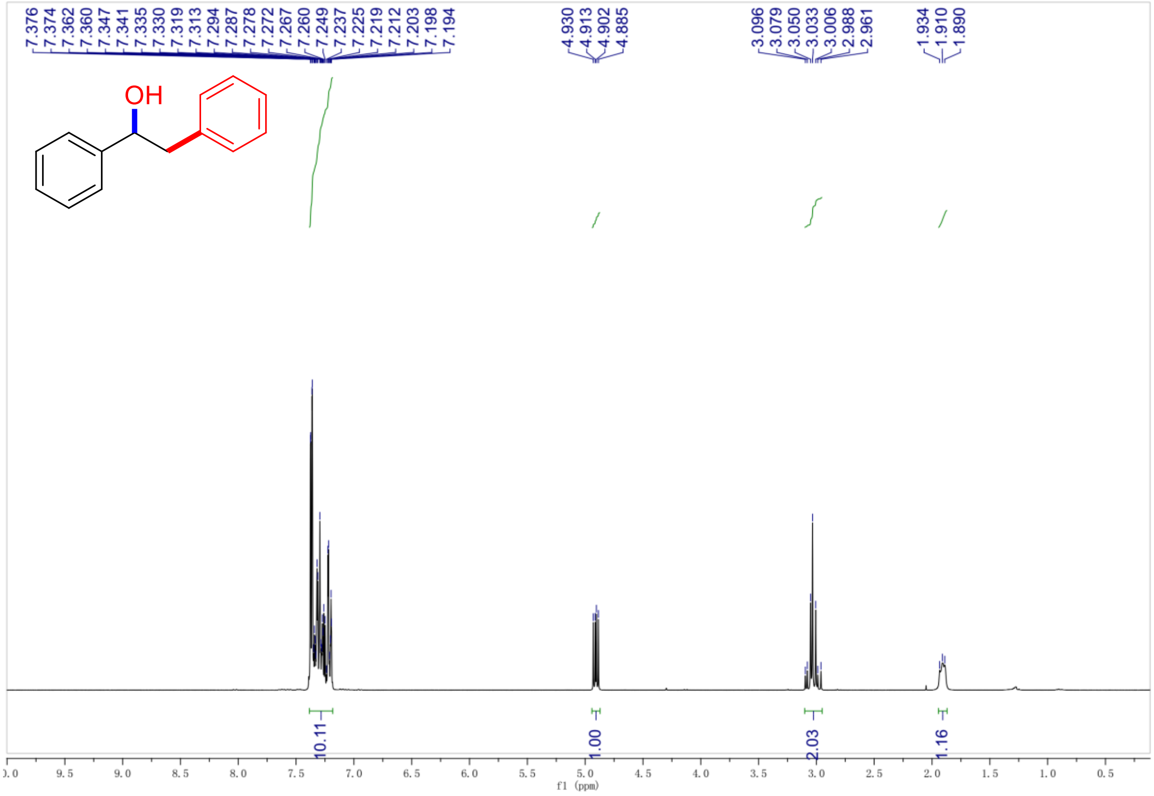

^{1}H NMR spectrum of compound **65** in $CDCl_3$ (300 MHz).

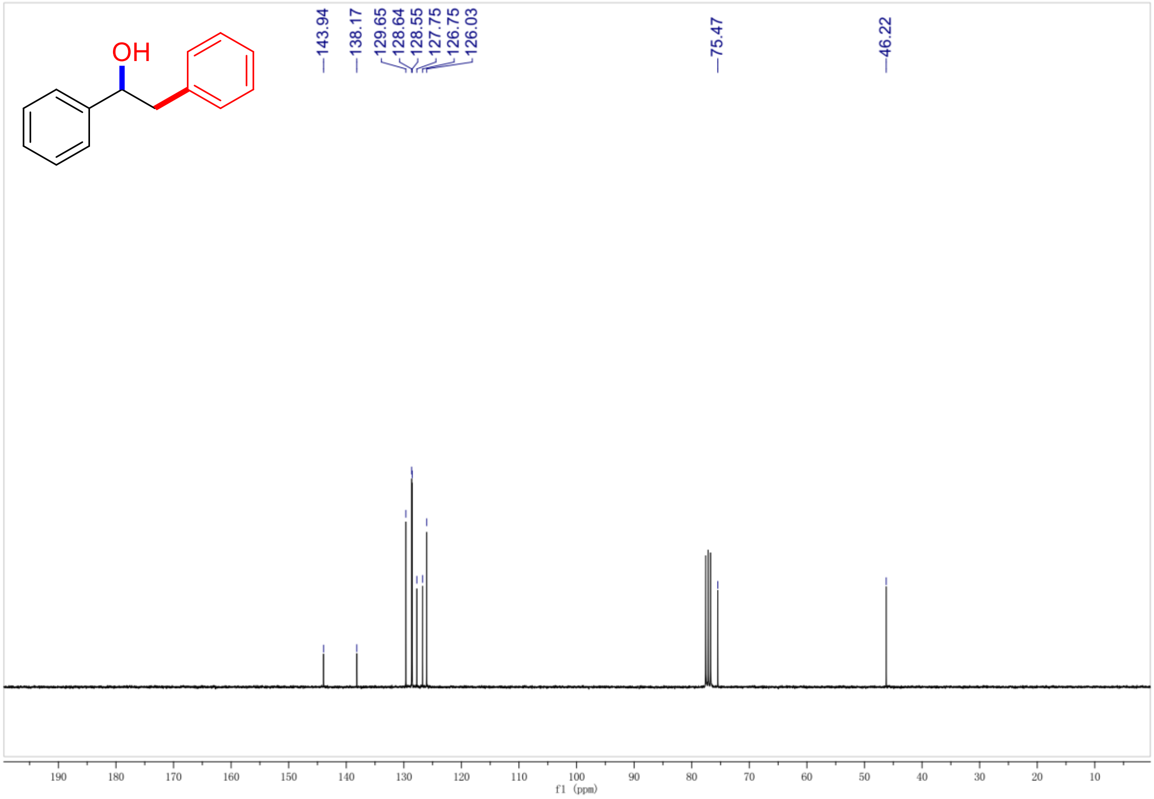

$^{13}C\{^{1}H\}$ NMR spectrum of compound **65** in $CDCl_3$ (75 MHz).

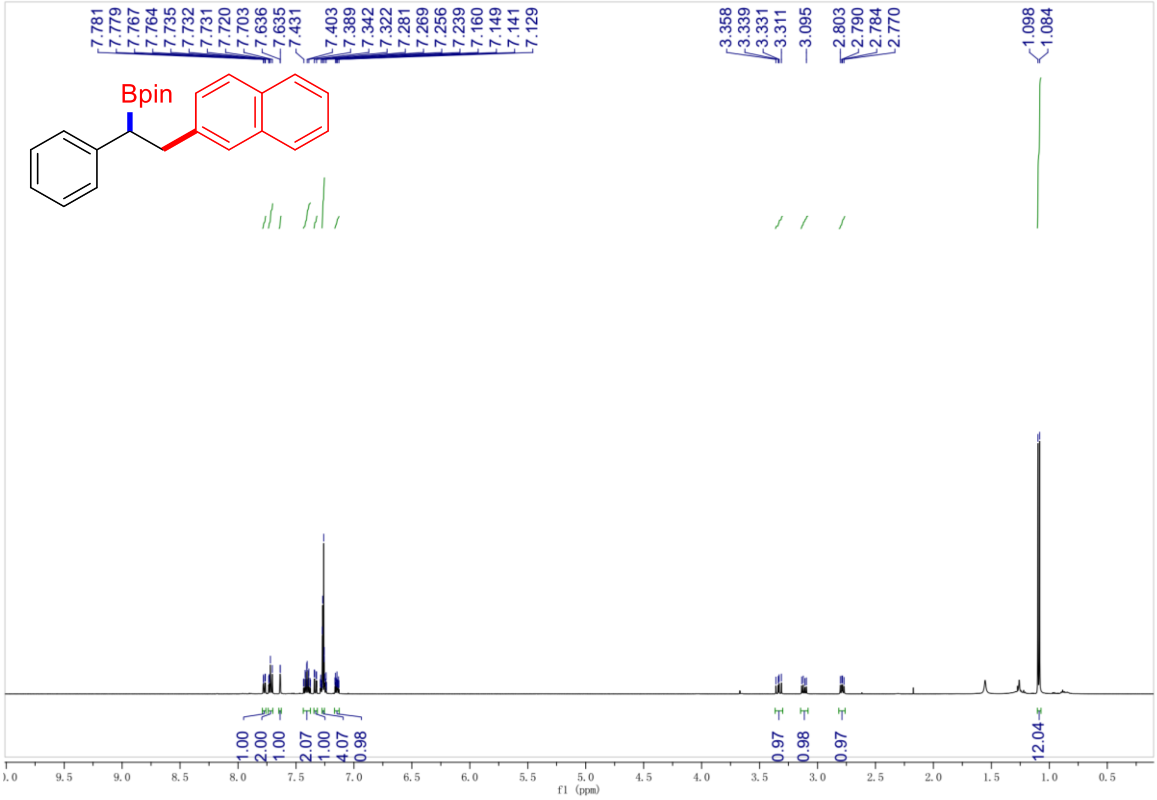

^{1}H NMR spectrum of compound **66** in $CDCl_3$ (500 MHz).

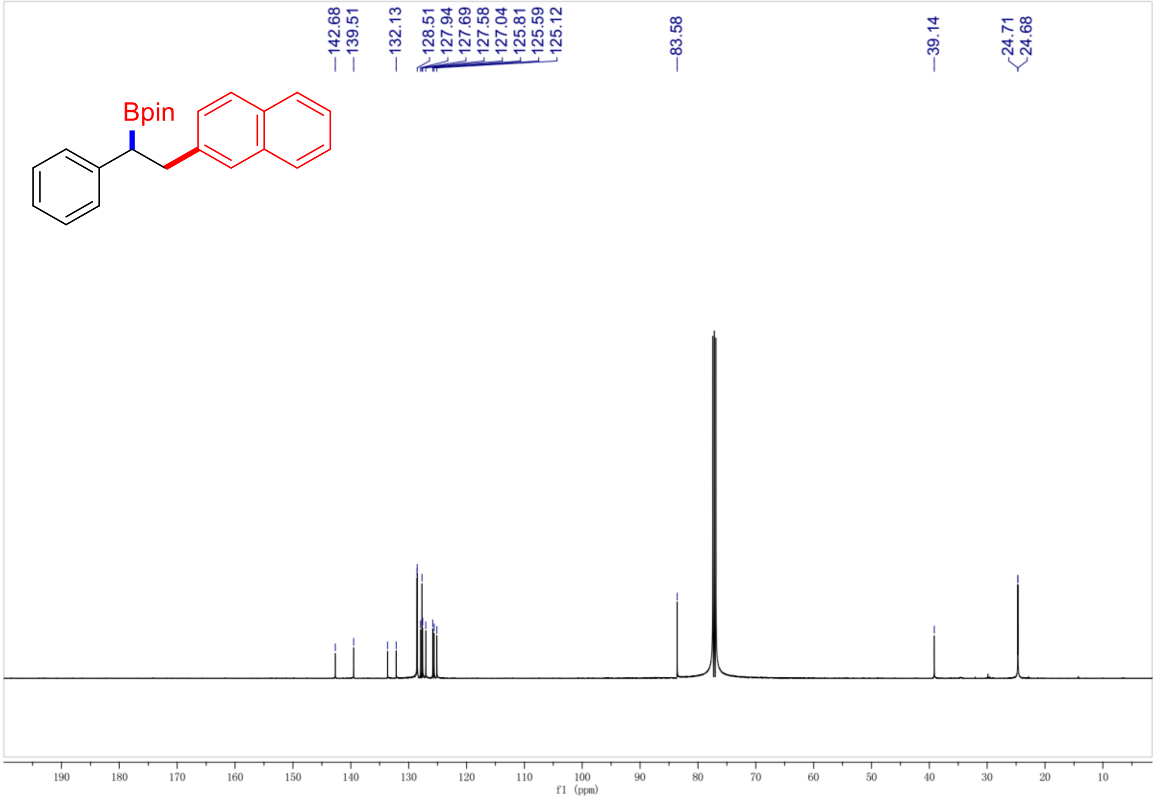

$^{13}C\{^{1}H\}$ NMR spectrum of compound **66** in $CDCl_3$ (125 MHz).

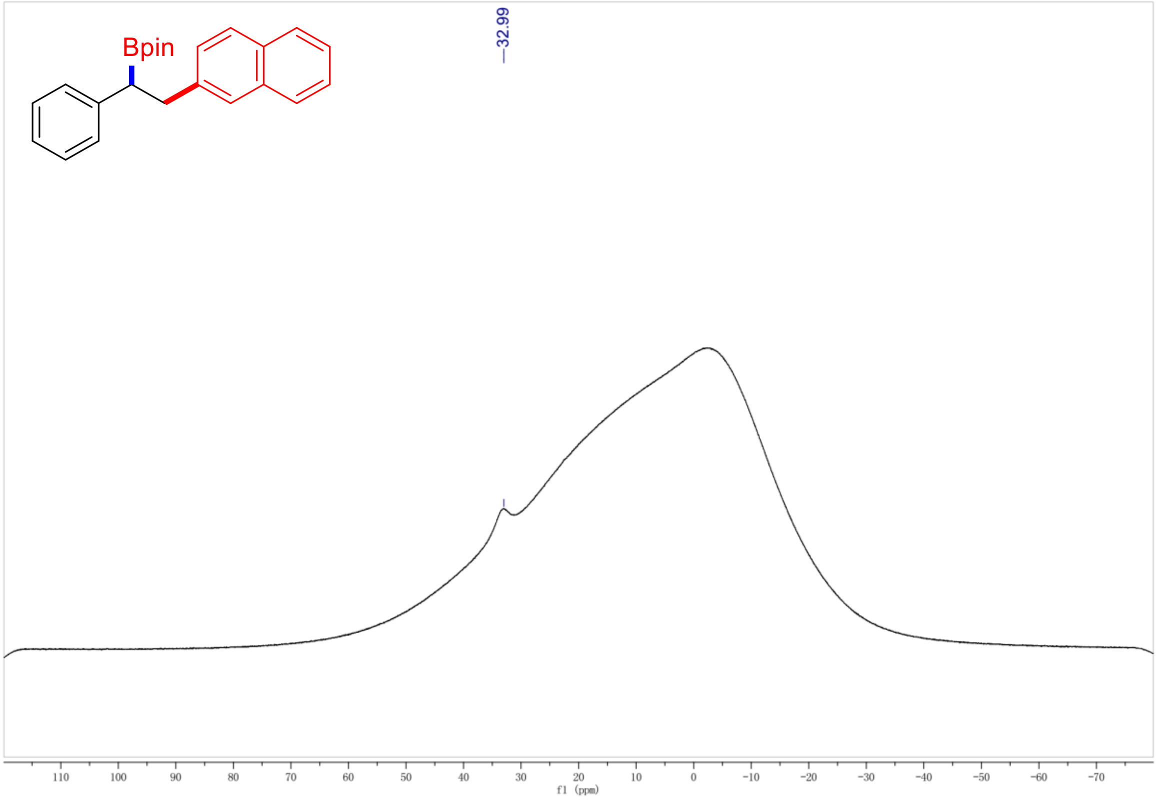

$^{11}B\{^{1}H\}$ NMR spectrum of compound **66** in $CDCl_3$ (160 MHz).

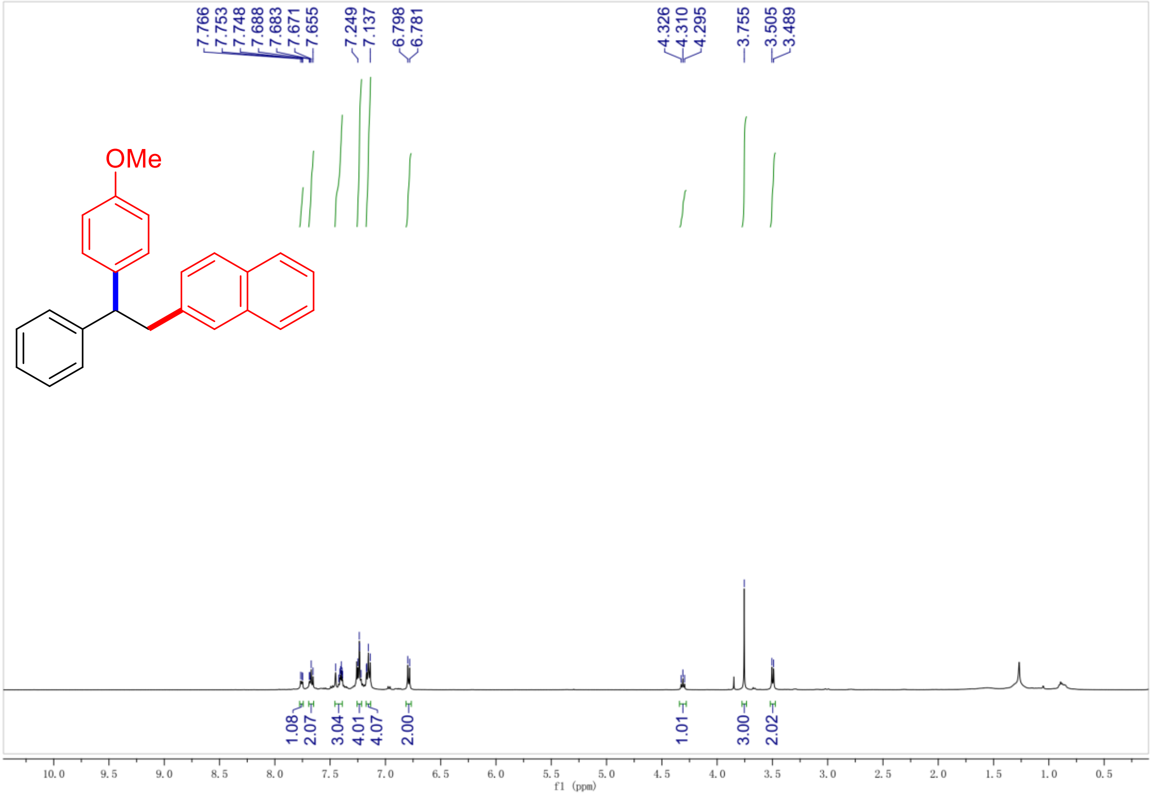

^{1}H NMR spectrum of compound **67** in $CDCl_3$ (500 MHz).

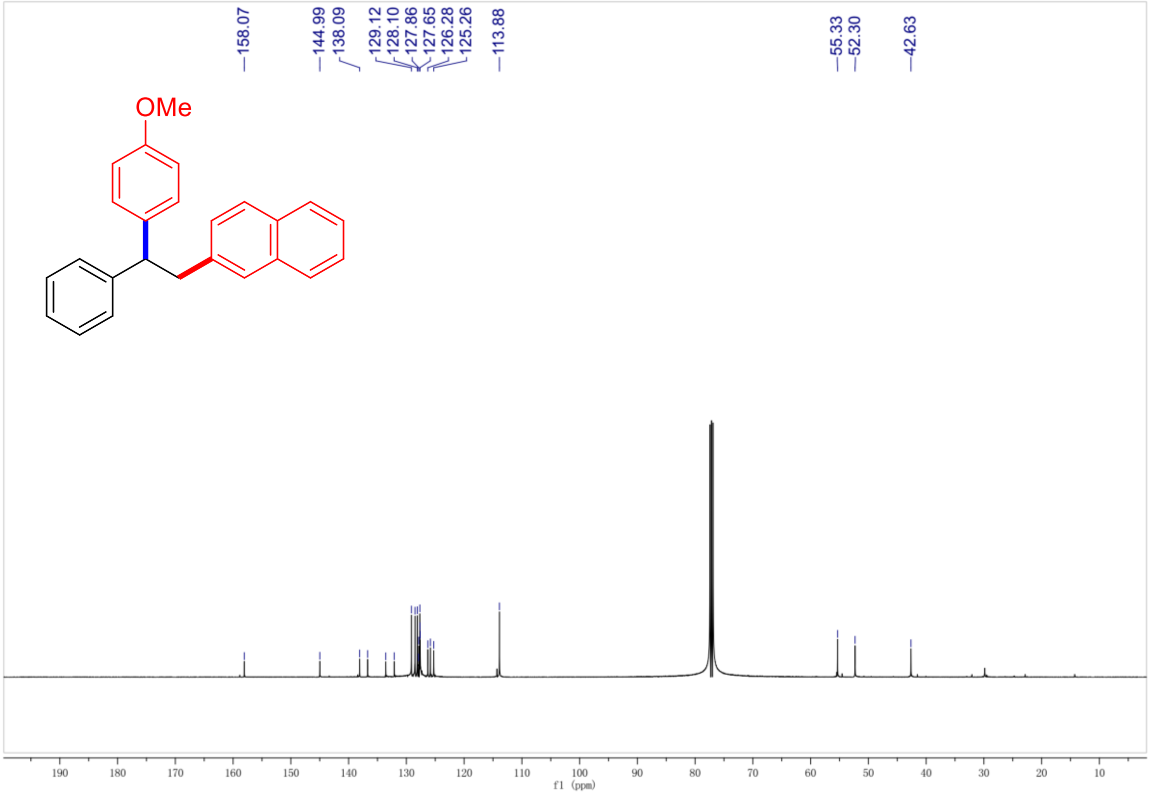

^{13}C{^{1}H} NMR spectrum of compound **67** in $CDCl_3$ (125 MHz).

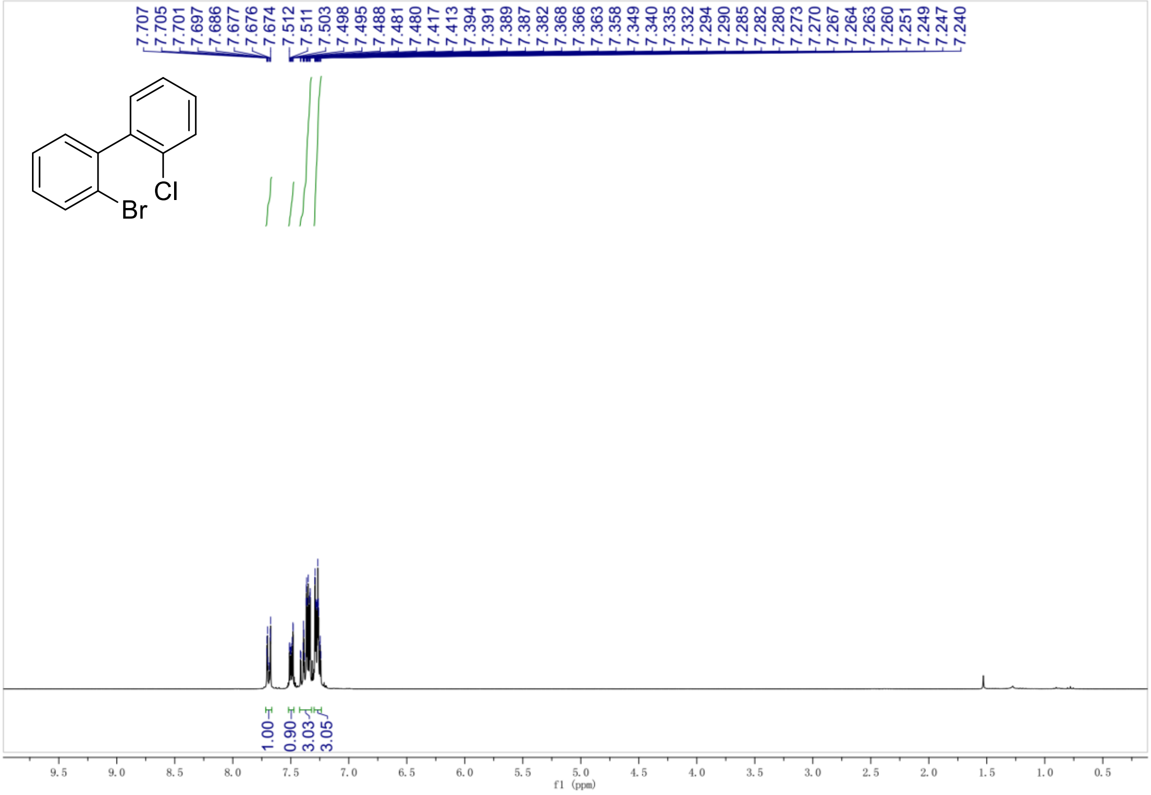

^{1}H NMR spectrum of **2-Bromo-2'-chloro biaryl** in $CDCl_3$ (300 MHz).

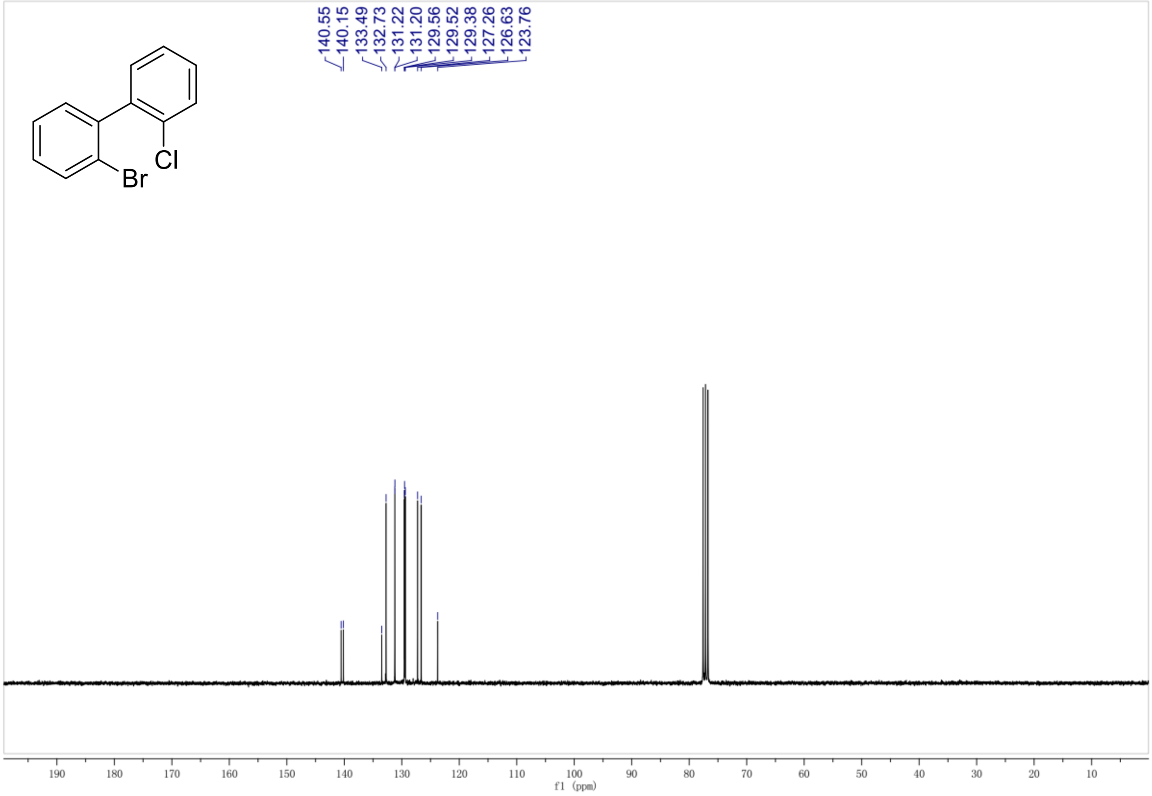

^{13}C{^{1}H} NMR spectrum of **2-Bromo-2'-chloro biaryl** in $CDCl_3$ (75 MHz).

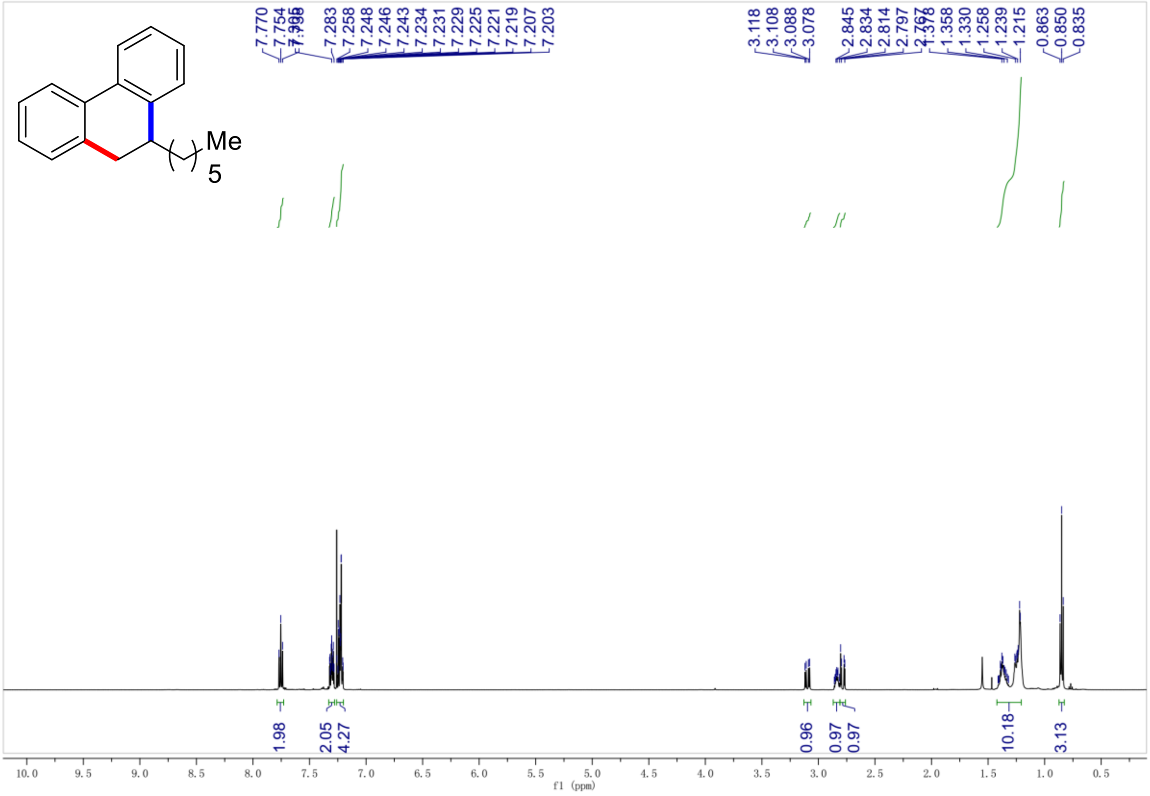

^{1}H NMR spectrum of compound **68** in $CDCl_3$ (500 MHz).

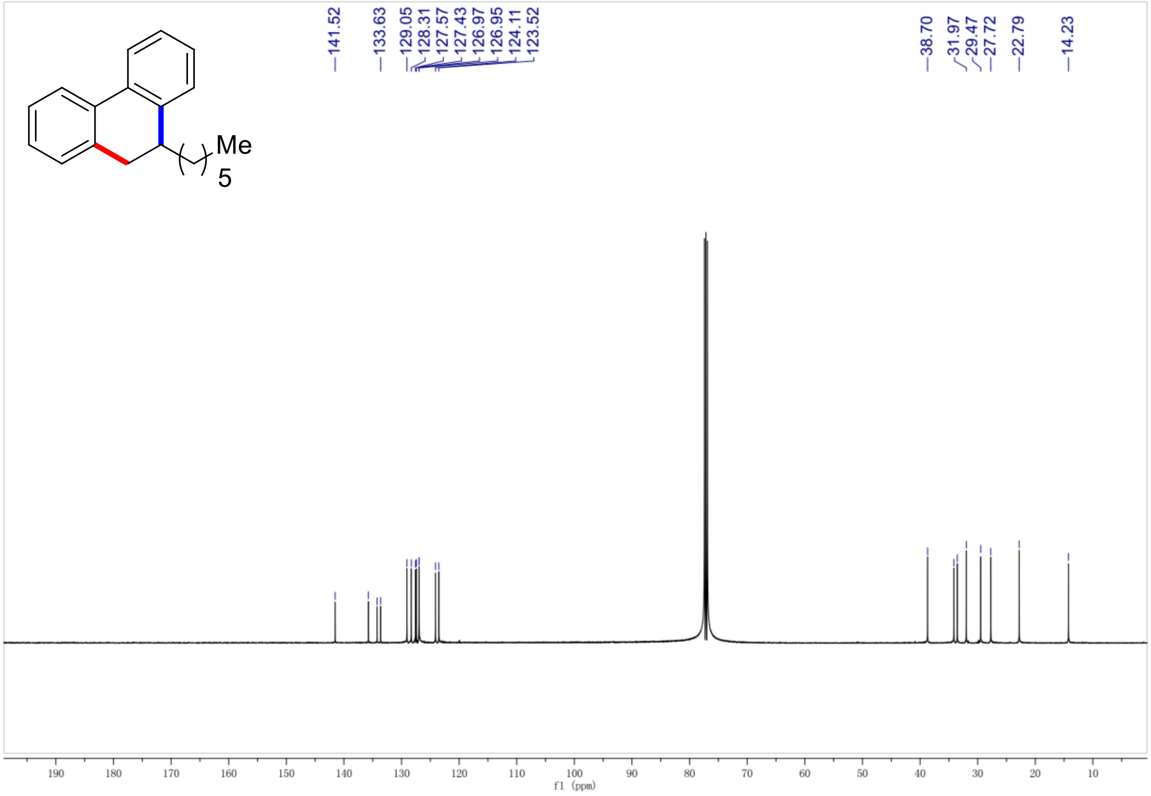

^{13}C{^{1}H} NMR spectrum of compound **68** in $CDCl_3$ (125 MHz).

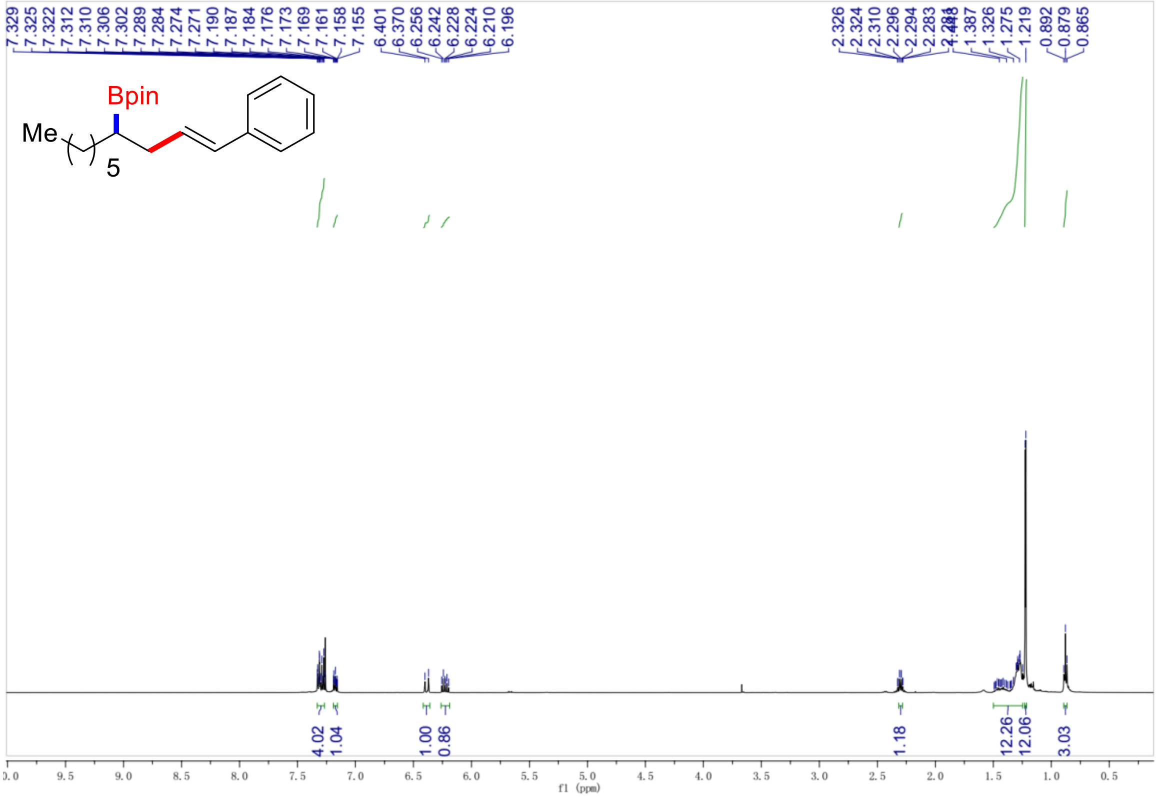

^{1}H NMR spectrum of compound **69** in $CDCl_3$ (500 MHz).

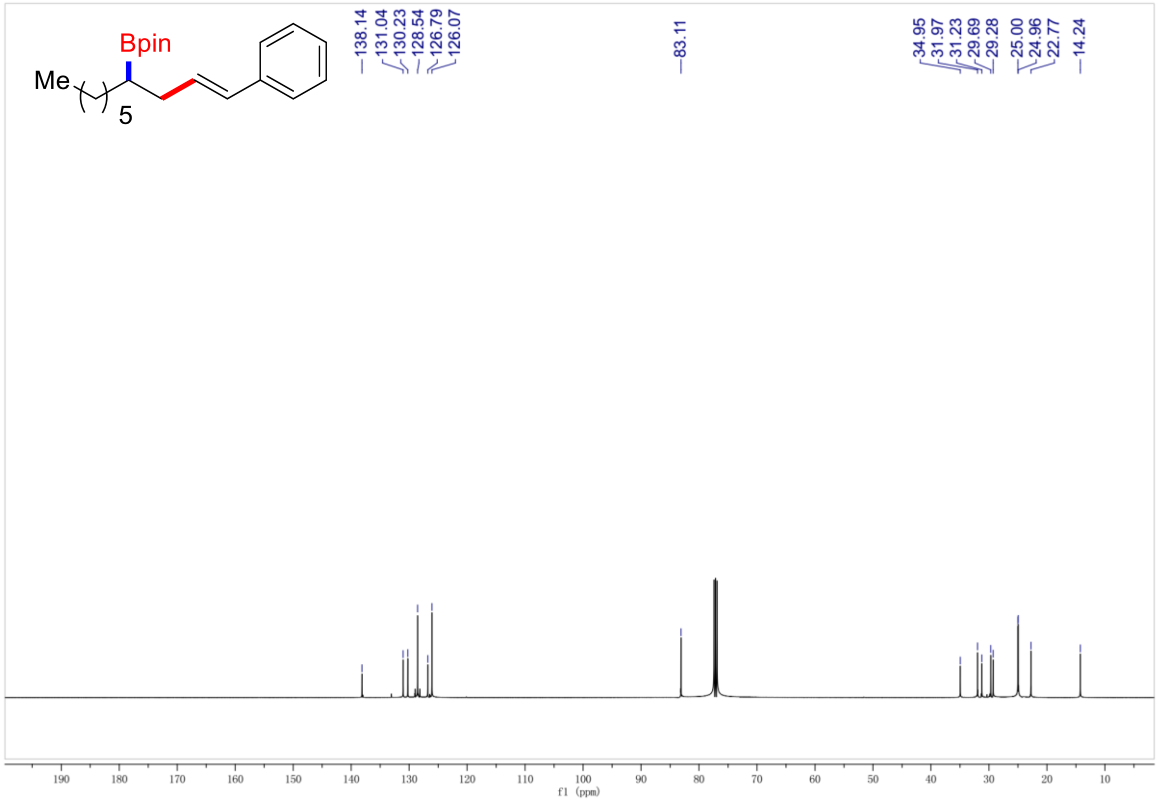

^{13}C{^{1}H} NMR spectrum of compound **69** in $CDCl_3$ (125 MHz).

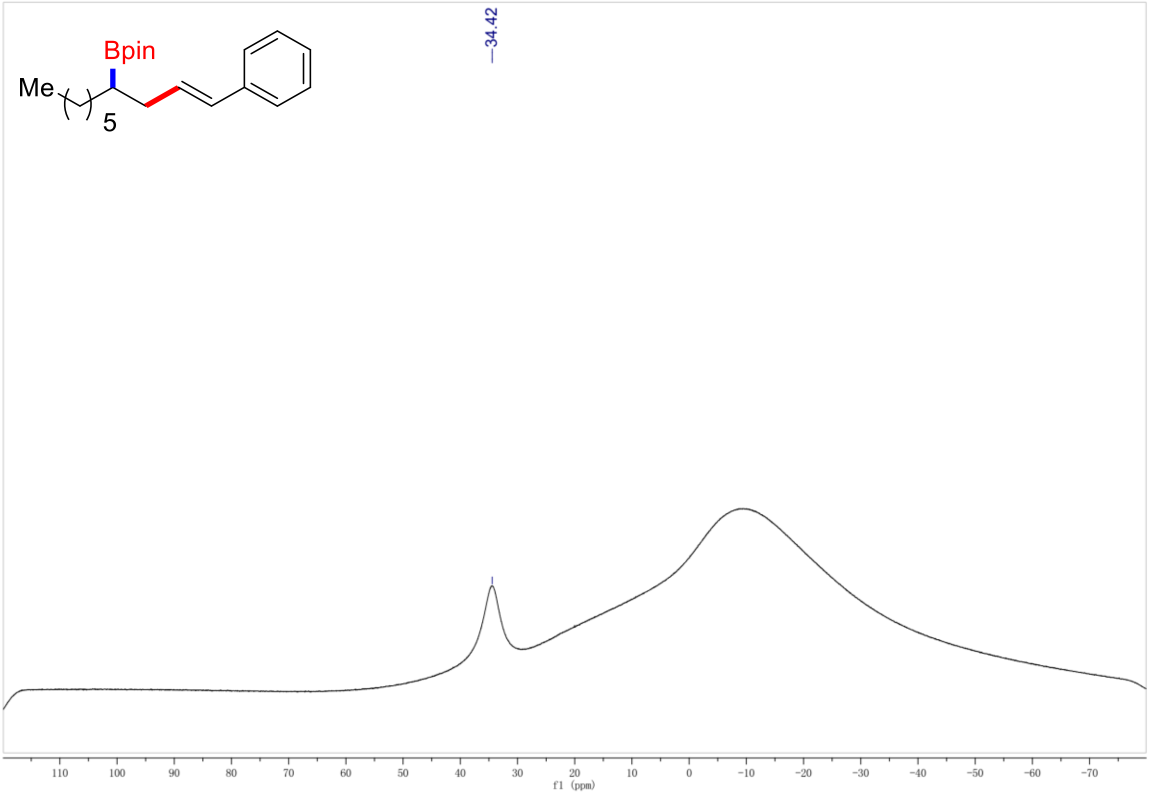

$^{11}B\{^1H\}$ NMR spectrum of compound **69** in $CDCl_3$ (160 MHz).

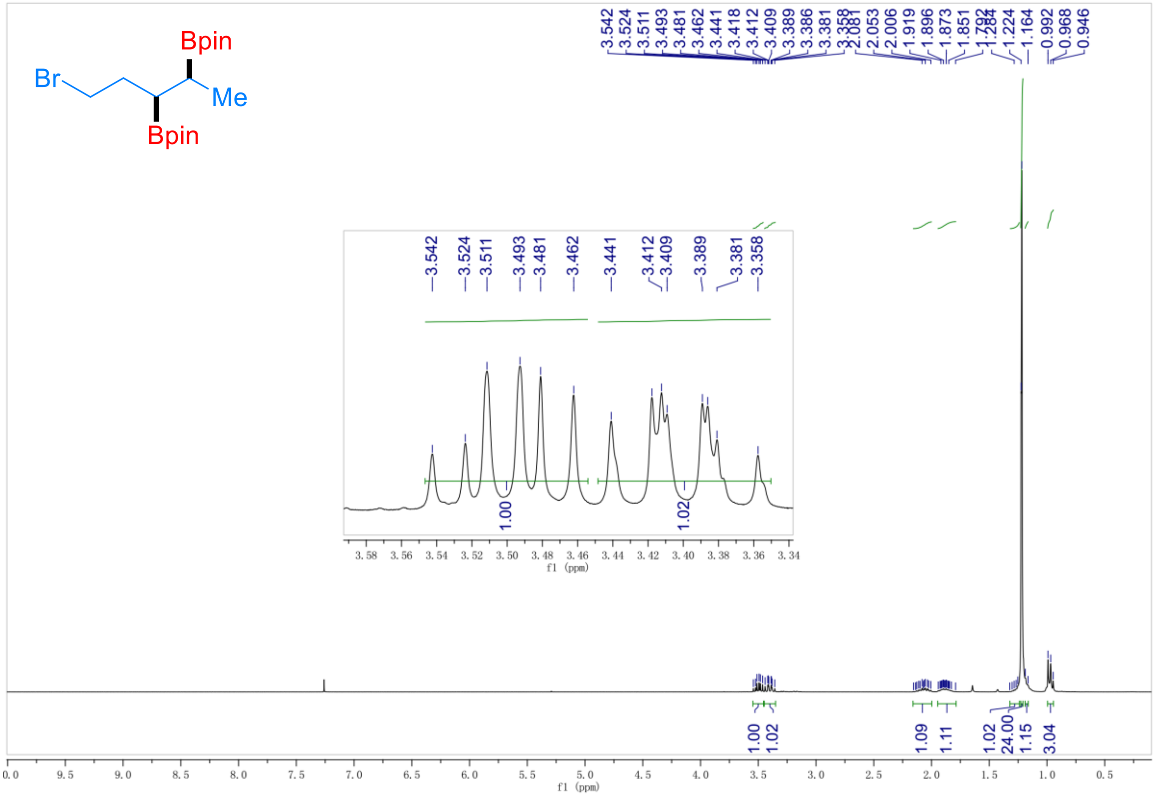

^1H NMR spectrum of compound **70b** in $CDCl_3$ (400 MHz).

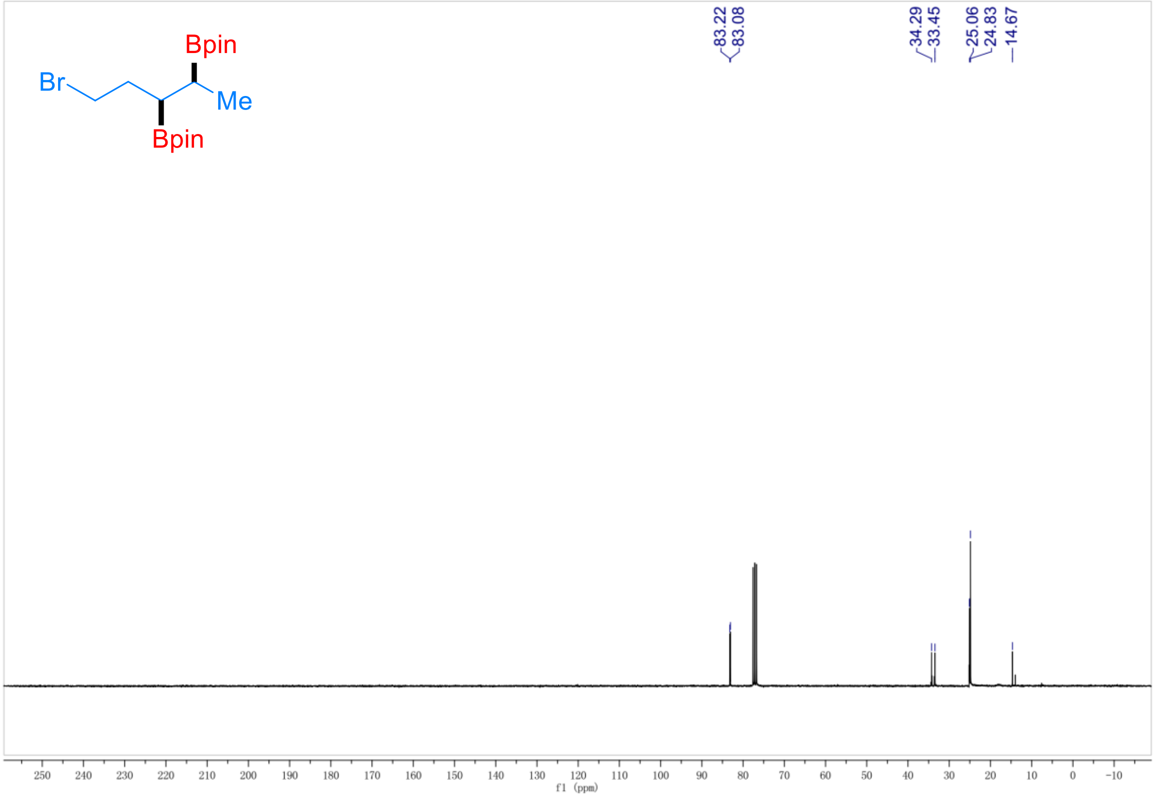

$^{13}C\{^{1}H\}$ NMR spectrum of compound **70b** in $CDCl_3$ (100 MHz).

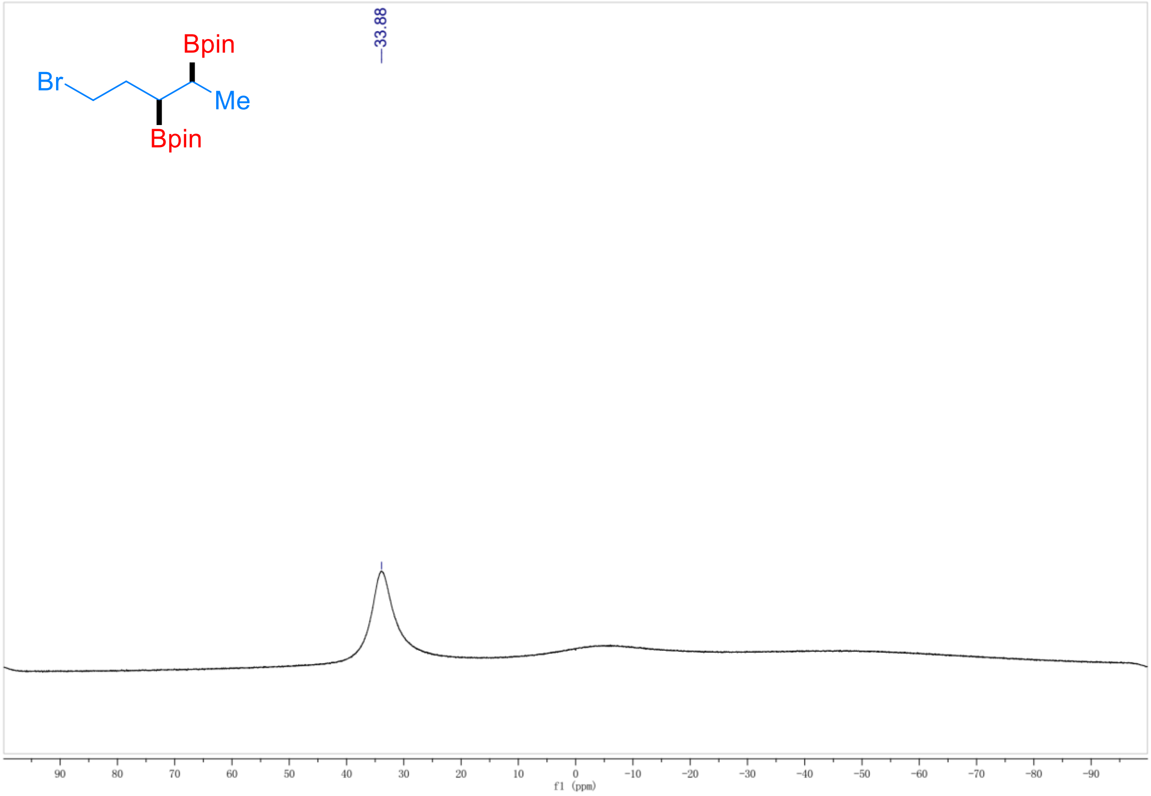

$^{11}B\{^{1}H\}$ NMR spectrum of compound **70b** in $CDCl_3$ (128 MHz).

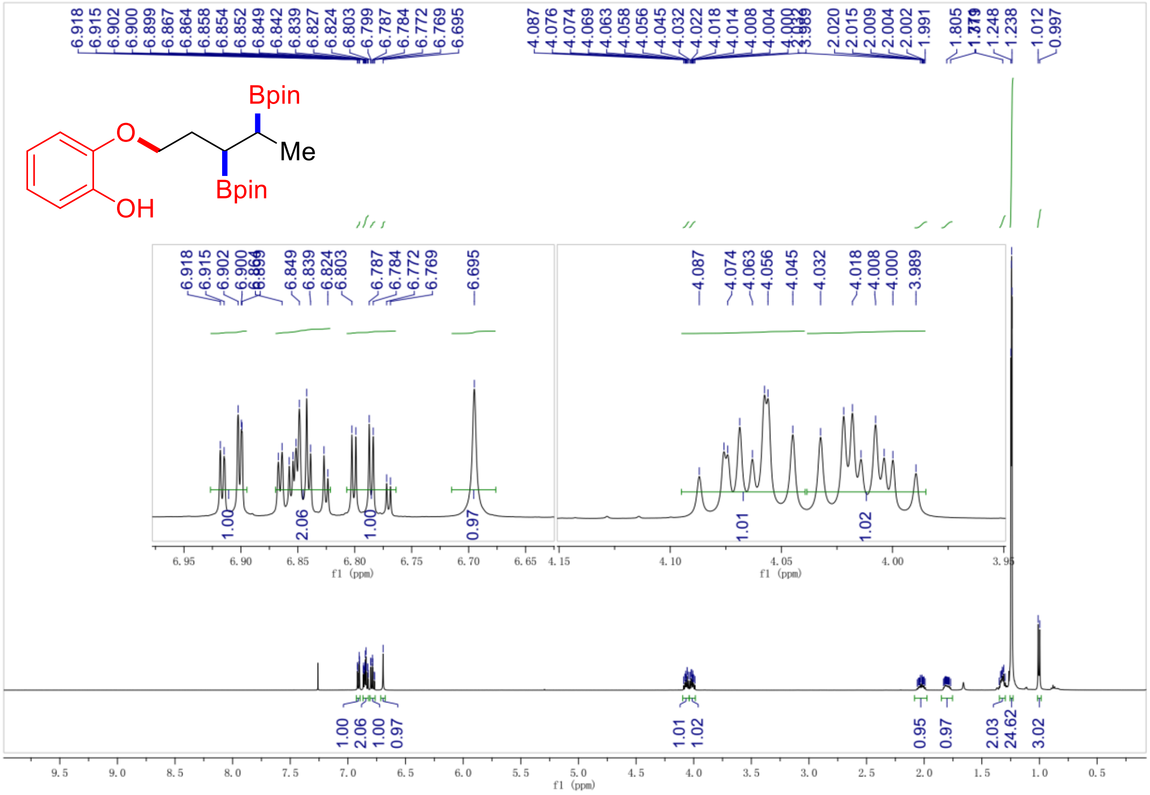

^{1}H NMR spectrum of compound **70b-1** in $CDCl_3$ (500 MHz).

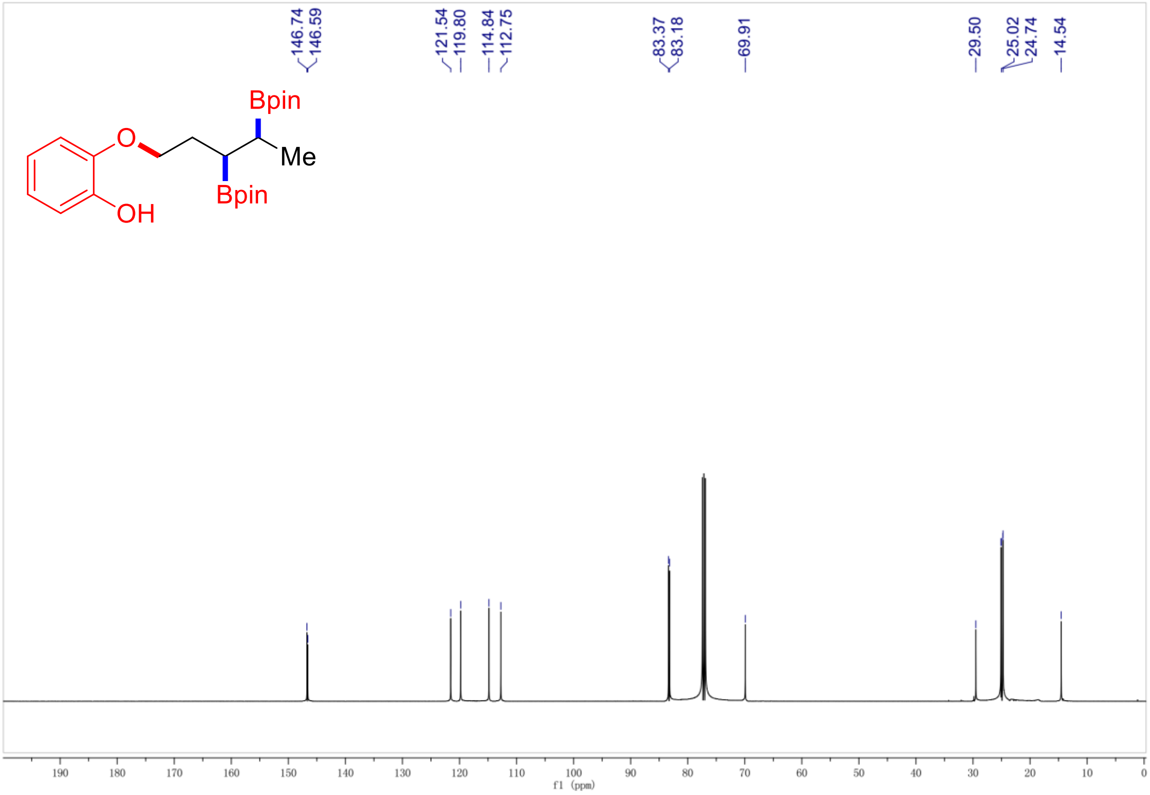

^{13}C{^{1}H} NMR spectrum of compound **70b-1** in $CDCl_3$ (125 MHz).

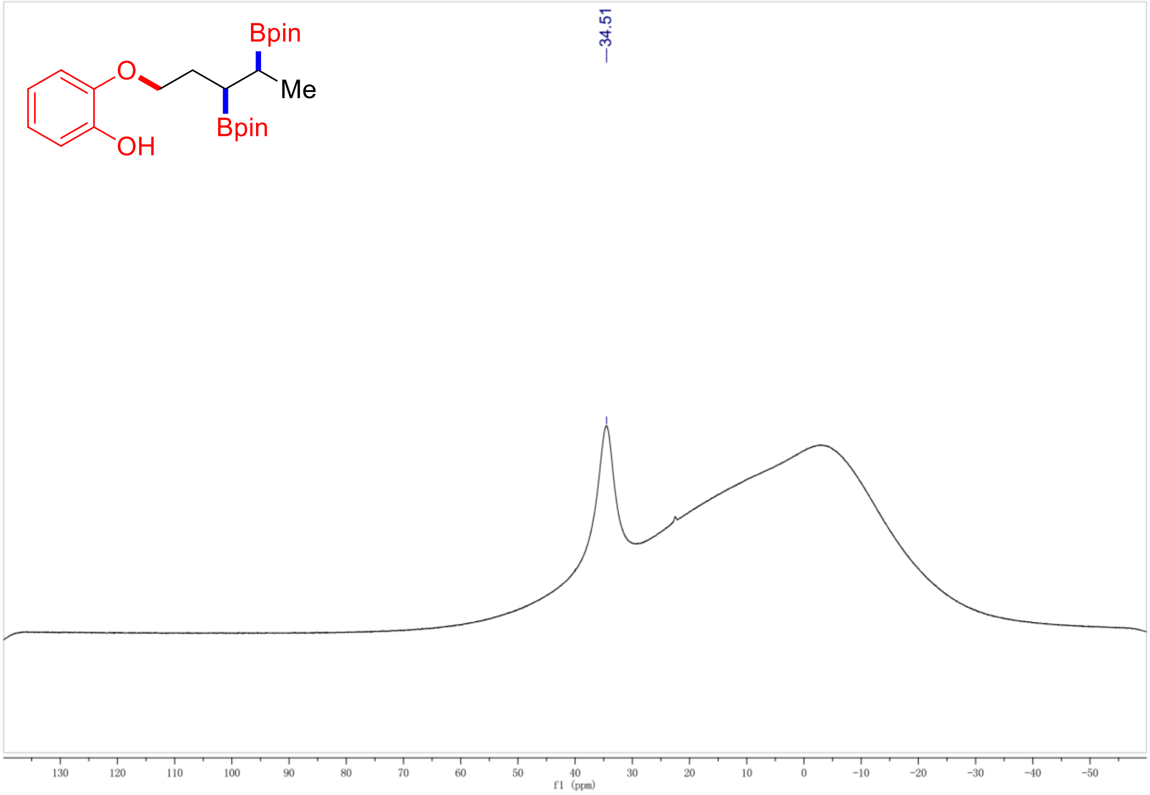

$^{11}B\{^{1}H\}$ NMR spectrum of compound **70b-1** in $CDCl_3$ (160 MHz).

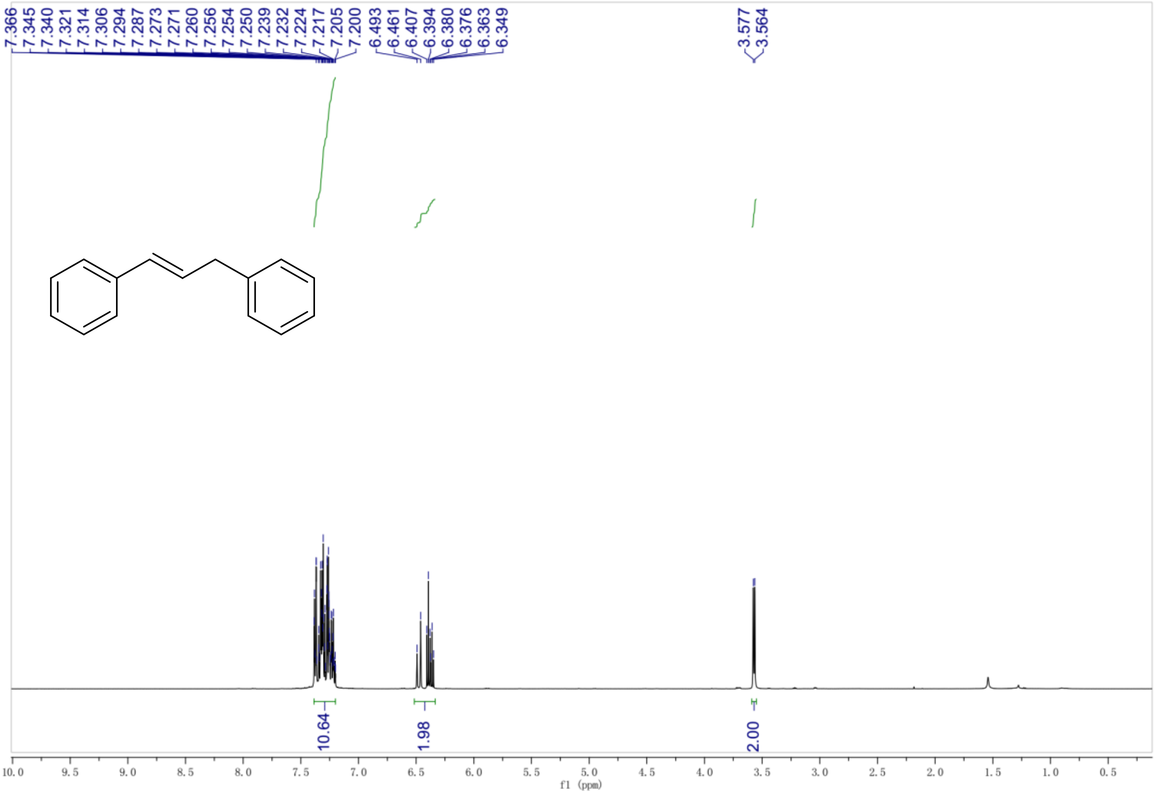

^{1}H NMR spectrum of compound **22c** in $CDCl_3$ (500 MHz).

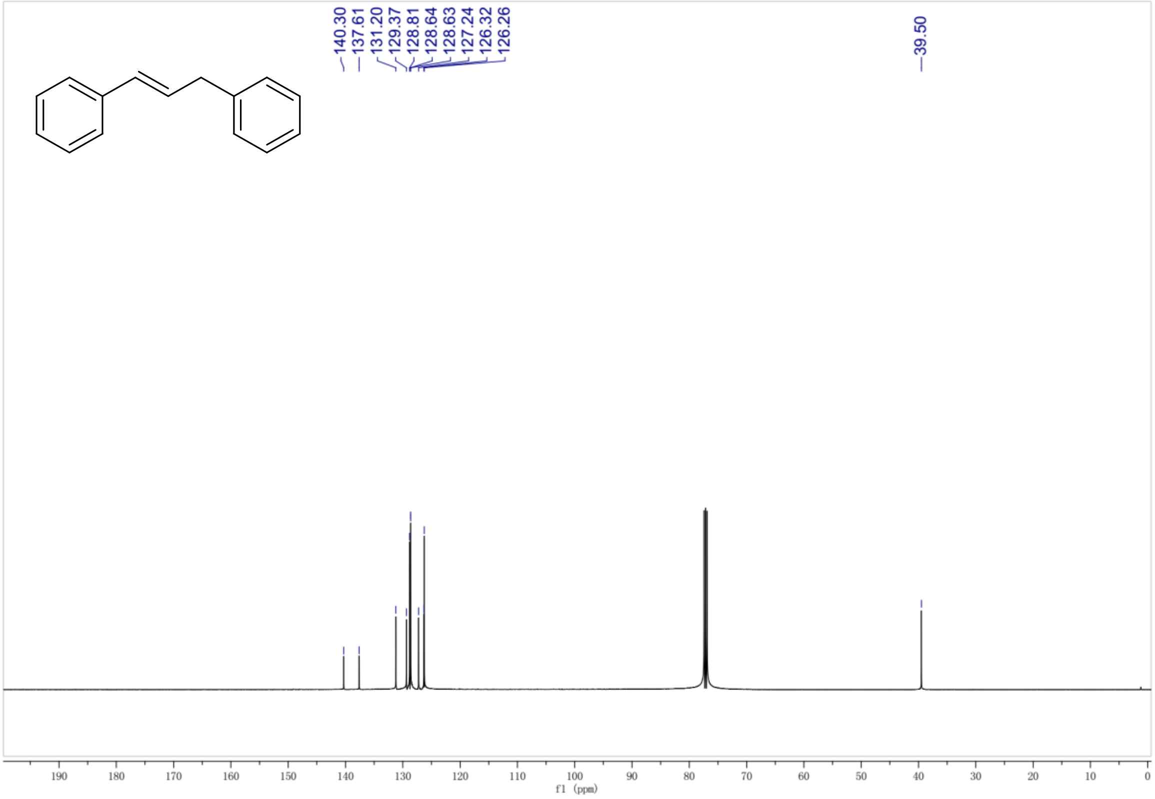

$^{13}C\{^{1}H\}$ NMR spectrum of compound **22c** in $CDCl_3$ (125 MHz).
